# Exocytosis and protein secretion in *Trypanosoma*

**DOI:** 10.1186/1471-2180-10-20

**Published:** 2010-01-26

**Authors:** Anne Geiger, Christophe Hirtz, Thierry Bécue, Eric Bellard, Delphine Centeno, Daniel Gargani, Michel Rossignol, Gérard Cuny, Jean-Benoit Peltier

**Affiliations:** 1 UMR 177, IRD-CIRAD, CIRAD TA A-17/G, Campus International de Baillarguet, 34398 Montpellier Cedex 5, France; 2 INRA, UR1199, LPF; 2, place Pierre Viala - Bât. 13; 34060 Montpellier Cedex 01, France; 3 UFR Odontologie, EA 4203, 545 Avenue du Pr Viala, 34193 Montpellier cedex 5, France; 4 UMR, Biologie et Génétique des interactions Plante-Parasite, INRA, CIRAD, SUPAGRO, TA A54/K, Campus international de Baillarguet, 34398 Montpellier cedex 5, France

## Abstract

**Background:**

Human African trypanosomiasis is a lethal disease caused by the extracellular parasite *Trypanosoma brucei*. The proteins secreted by *T. brucei *inhibit the maturation of dendritic cells and their ability to induce lymphocytic allogenic responses. To better understand the pathogenic process, we combined different approaches to characterize these secreted proteins.

**Results:**

Overall, 444 proteins were identified using mass spectrometry, the largest parasite secretome described to date. Functional analysis of these proteins revealed a strong bias toward folding and degradation processes and to a lesser extent toward nucleotide metabolism. These features were shared by different strains of *T. brucei*, but distinguished the secretome from published *T. brucei *whole proteome or glycosome. In addition, several proteins had not been previously described in *Trypanosoma *and some constitute novel potential therapeutic targets or diagnostic markers. Interestingly, a high proportion of these secreted proteins are known to have alternative roles once secreted. Furthermore, bioinformatic analysis showed that a significant proportion of proteins in the secretome lack transit peptide and are probably not secreted through the classical sorting pathway. Membrane vesicles from secretion buffer and infested rat serum were purified on sucrose gradient and electron microscopy pictures have shown 50- to 100-nm vesicles budding from the coated plasma membrane. Mass spectrometry confirmed the presence of *Trypanosoma *proteins in these microvesicles, showing that an active exocytosis might occur beyond the flagellar pocket.

**Conclusions:**

This study brings out several unexpected features of the secreted proteins and opens novel perspectives concerning the survival strategy of *Trypanosoma *as well as possible ways to control the disease. In addition, concordant lines of evidence support the original hypothesis of the involvement of microvesicle-like bodies in the survival strategy allowing *Trypanosoma *to exchange proteins at least between parasites and/or to manipulate the host immune system.

## Background

The *Trypanosomatidae *family comprises genera that infect many kinds of eukaryotes: insects, fish, amphibians, reptiles, birds, mammals, and even plants. In the *Trypanosoma *genus, three species are pathogenic for humans (*Trypanosoma brucei, T. cruzi*, and *T. evansi*). Human African trypanosomiasis (HAT, or sleeping sickness) is caused by *T. brucei *and transmitted by tsetse flies (*Glossina *sp.). In contrast to most other insect-transmitted parasites, *T. brucei *spends its entire cycle as an extracellular parasite. To thwart the host immune system, the parasite has developed population survival strategies. Through antigenic variation, trypanosomes shield their plasma membrane with a continually switching densely packed layer of 5 × 10^6 ^dimers of variant surface glycoprotein (VSG), which constitutes a surface coat. This coat is indeed composed of a single protein, but the parasite genome has a repertoire of about 2,000 different potential VSG genes that are expressed in a mutually exclusive manner. The coat also prevents antibodies from gaining access to necessarily invariant surface molecules [1-3].

HAT is lethal when untreated and is a threat for over 60 million people living in sub-Saharan countries [4,5]. Treatment of the disease is difficult and expensive and has potentially life-threatening side effects [6,7]. Since today there is no prophylactic chemotherapy, specific, low-cost, and sensitive methods for the early diagnosis of the parasite in human blood samples are needed, as well as novel therapeutic targets for fighting the parasite. A class of particularly interesting proteins are the expressed/secreted proteins (ESPs), which are specifically secreted by parasites. Several ESPs are involved in various aspects of the pathogenesis [8-10]. In addition, we have previously shown that the secretome of *T. brucei *inhibits the maturation of dendritic cells and their ability to induce lymphocytic allogenic responses [11]. As the majority of ESPs of the secretome remain unknown, we used a proteomics-based approach to analyze the entire secretome of the parasite.

In this study, we compared three different *T. brucei gambiense *strains, identified over 440 proteins and determined their protomeric status. We isolated microvesicles from the secretion medium and showed in microscopy the budding of these microvesicles at the parasite surface before and after incubation in the secretion medium. Moreover, microvesicles were also isolated directly from infected rat serum and the proteome of these microvesicles was similar to the secretome. This extended overview demonstrates that ESPs play an unexpected major role in the trypanosome survival strategy via these microvesicles and highlights a number of potential therapeutic strategies to control the disease.

## Results

**Figure 1:**
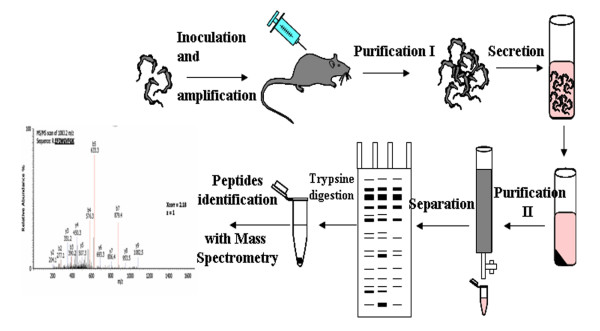
**General purification procedure**. Trypanosomes were intraperitoneally injected into rats. When their multiplication reached the logarithmic growth stage, parasites were purified from blood by chromatography and resuspended in secretion medium. After 2 h, parasites were removed by centrifugation and secreted proteins (ESPs) were purified through chromatography. ESPs were separated on polyacrylamide gel electrophoresis (PAGE), stained before mass spectrometry (MS/MS) analysis.

Parasites amplified from rats were incubated in a secretion medium mimicking blood but containing no proteins. A set of soluble proteins (secretome) was recovered after incubation and submitted to proteomic analysis (Figure 1). No protein was obtained after incubation in the secretion medium when the parasites were omitted.

### Characterization of the secretome of *T. brucei gambiense*

#### 1- Comparison of different *T. brucei *strains reveals potential strain markers

**Figure 2:**
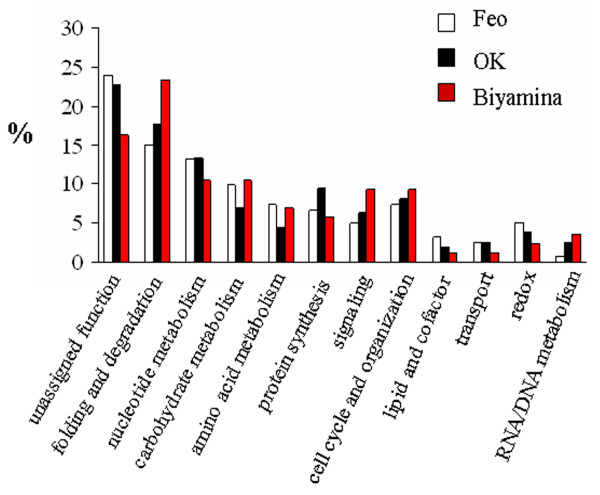
**Classification of *T. brucei gambiense *proteins from 3 different strains into functional categories**. Proteins from the different strains (Feo, OK, Biyamina) were classified into 12 functional categories according to the hierarchical, nonredundant classification system developed for MapMan [13]. On the x-axis, the categories are indicated. The y-axis shows the percentage of each category for each strain.

**Figure 3:**
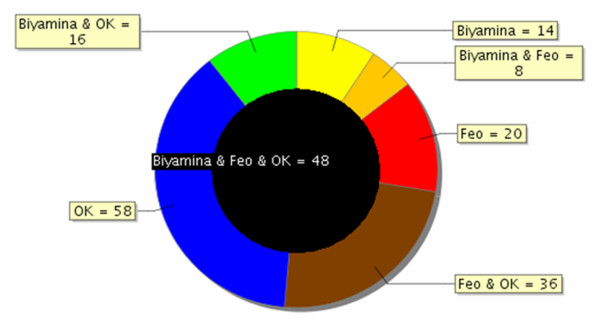
**Overlap between secretomes of 3 different *T. brucei gambiense *strains**. Proteins found in the analysis of 3 different *T. brucei *strain secretomes separated on 1D-PAGE were compared. The black circle in the middle represents proteins common to the 3 strains (48 proteins). Biyamina and OK have 16 proteins in common; 14 proteins are specific to the Biyamina secretome.

*T. brucei gambiense *is divided into two groups [12]: the Feo and OK strains are two strains belonging to group I, while Biyamina belongs to the less homogeneous group II. All three strains were found to secrete complex sets of proteins ranging from 7 to 150 kDa. Reproducibility of the protein profiles has been controlled in several independent experiments (from trypanosome production, protein secretion process to electrophoretic runs); in addition, SDS-PAGE controls on secretion samples taken after a 2-h secretion showed the same profiles as those performed on samples taken after a 30-min stimulation (data not shown). After extensive sampling of all 1D gel lanes, 356 proteins (112 for Feo, 158 for OK, and 86 for Biyamina strains) were identified by LC-ESI MS/MS (liquid chromatography-electrospray tandem mass spectrometry) (additional file 1, Table S1) and grouped into 12 main functional classes according to the nonredundant classification system developed for MapMan [13]. No rat proteins were identified when specified database searches were done with Mascot. A summary of the functions of ESPs is shown in Figure 2. For all strains, about 50% of the proteins belonged to three major categories: protein folding and degradation, nucleotide metabolism, and unassigned functions. Forty percent of the remaining proteins fell into one of five categories: carbohydrate metabolism, amino acid metabolism, protein synthesis, signaling, and cell cycle and cell organization. In spite of a globally similar functional classification, the contribution of proteins involved in signaling and protein synthesis was quite different between the three strains. In addition, some proteins were specifically identified by one strain (Figure 3) and are therefore potential candidates for strain discrimination and/or to understand their pathogenicity. Other than proteins with no known function, these markers included specific isoforms of adenylate kinase and lysophospholipase in Feo, a dihydrolipoyl dehydrogenase in Biyamina, and a specific isoform of adenine phosphoribosyltransferase and a calpain-like cysteine peptidase, as well as a tryparedoxin for the OK strain.

#### 2- Secreted proteins form stable complexes

**Figure 4:**
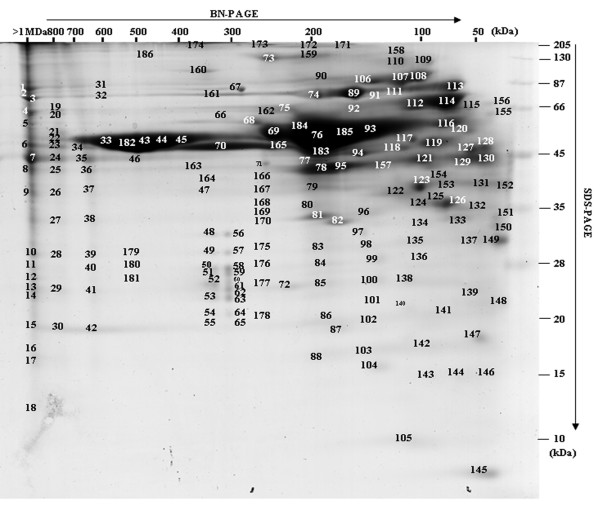
**BN-PAGE separation of the *T. brucei gambiense *secretome (OK strain)**. Proteins were separated by native gel electrophoresis (BN-PAGE) and stained with coomassie brilliant blue. Coomassie-stained protein spots (186) were excised, digested with trypsin, and identified by MS/MS. 382 proteins were identified and the associated data (accession numbers, molecular masses and MS/MS data) are presented in additional file 2, Table S2.

To further understand the secretome and its interaction network, protein complexes were separated using two-dimensional BN-SDS-PAGE (blue native-sodium dodecyl sulfate-polyacrylamide gel electrophoresis) [14]. With this method, proteins focusing on a virtual vertical lane are potentially part of the same complex, whereas proteins not in a complex are focused at the same molecular weight (MW) in both dimensions and located at the extreme right on the gel (Figure 4). Gels have been carried out two times giving similar protein profiles. A total of 382 nonredundant proteins were identified by MS/MS (additional file 2, Table S2). Functional classification led to a similar distribution as above (see Figure 2). Figure 4 highlights the importance of a small number of protein spots (<20) that accounted for more than 80% of the total amount of secreted proteins. These proteins included not only the well-known and abundant VSGs (spots 33, 182, 43), but also enzymes involved in nucleotide and amino acid metabolism (spots 76, 123, 126), chaperones (spots 114, 113, 89, 107), and proteases (spots 165, 114), thus defining a major role for defense and nutrition to the secretome. Some monomeric proteins were observed, including for instance two *14-3-3 *proteins (spot 149), calreticulin (spot 127-130), and ubiquitin (spot 145), as well as proteins showing more than one protomeric stage, such as the hsp83 (heat shock protein) chaperone that was in the monomeric state for roughly one half (spot 113) and acted as a homodimer for the other half (spot 89). However, most secreted proteins were detected as homo- or heteroligomers. Two typical examples were the TCP-1 complex and the aminopeptidase M17. The TCP-1 complex is a chaperone complex of eight distinct subunit species (α, β, γ, δ, ε, η, θ and ζ)We identified the TCP-1 complex in spots 44 and 45 corresponding to a native mass between 400 and 450 kDa (expected size: 440 kDa). Aminopeptidase M17 (50 kDa) has been reported to form a homohexameric structure [15], and we found this enzyme (spot 165) with a native mass of approximately 250 kDa.

Another striking feature concerned the proteasome, which we identified in two forms (spots 48-55 and 56-65) in the secretome. The 20S proteasome is a 28-mer composed of two stacked heptameric rings of proteolytically active beta subunits, surmounted at each end by another heptameric ring of structural alpha subunits. Seven alpha and seven beta paralogs exist in the *T. brucei *genome and all of the 14 different subunits were identified in both lanes, except alpha3 in the highest MW complex. The 20S core is regulated by additional 19S or 11S complexes. In *T. brucei*, a form of the 20S proteasome showing enhanced peptidase activity was previously described, and a 26-kDa protein, PA26 (26-kDa proteasome activator protein), was proposed to correspond to the 11S activator known in mammals [16,17]. We identified PA26 in both complexes. Because of the sizes of the two proteasome complexes (300-350 kDa) and the average size of the alpha and beta subunits (~25 kDa), the two forms of the proteasome complex identified here probably contain a single ring of alpha and beta subunits. Moreover, from the size of the highest MW complex and the apparent stoichiometry between PA26 and the other subunits in the complex, the highest MW complex may represent the activated form of the complex. Finally, it should be pointed out that the 19S and 20S subunits were also identified in the unresolved part of the gel (spots 1-18), corresponding to complexes above 1000 kDa, and they could reveal a minor form of the 26S proteasome that has not been identified in *T. brucei *to date.

#### 3- Secreted proteins correspond to a specific subset of the trypanosome proteome

A few proteomic data sets were recently published for members of the *Trypanosomatidae *family, including the total proteome of *T. brucei *in the procyclic (insect) stage [18], that of the glycosome, a specialized compartment similar to peroxisomes and involved in sugar metabolism [19], and the secretome of the promastigote *Leishmania donovani*, where authors were able to distinguish between actively secreted proteins and cell-associated proteins [20].

To cross-correlate between the secretome and proteome data sets, we first searched for *Leishmania *orthologs in *T. brucei *using BLAST (Basic Local Alignment Search Tool) analysis. 281 out of the 358 *Leishmania *secretome entries were found to have an ortholog in *Trypanosoma *(additional file 3, Table S3), including 115 actively secreted proteins and 166 cell-associated proteins. Interestingly, a high proportion (61%) of the former was present in our *Trypanosoma *secretome, suggesting a close relationship between the actively secreted proteins in *Leishmania *and the *Trypanosoma *secreted proteins.

**Figure 5:**
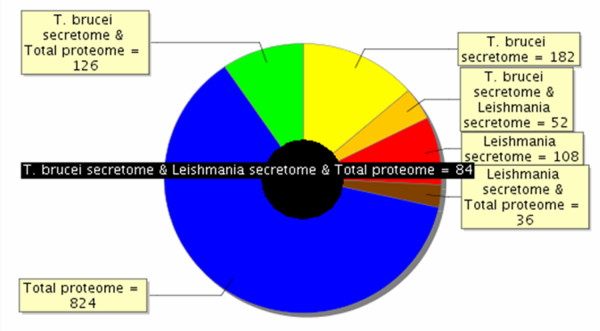
**Overlap between *Trypanosoma *total proteome and the *T. brucei gambiense *and *L. donovanii *secretome**. Proteins identified in 3 different compartments (*T. brucei *total proteome, *T. brucei gambiense *secretome, and *L. donovanii *secretome) were compared as to determine part of the proteins that were either specific to each compartment or common to different compartments. So, the black circle in the middle shows that 84 proteins are common to *T. brucei *total proteome, *T. brucei gambiense *secretome, and *L. donovanii *secretome. Among the other proteins of the *T. brucei gambiense *secretome, for example, 182 (41%) were specific to this compartment, whereas 52 were common with *L. donovanii *secretome, and 126 with the total proteome; out of the proteins identified in the total *T. brucei *proteome, 824 were specific to this compartment.

In contrast, only 8% of the *Trypanosoma *secretome was shared with the glycosome proteome (additional file 4, Table S4). We also compared the trypanosome total proteome (additional file 5, Table S5) and the secretomes from *Trypanosoma *and *Leishmania*. Figure 5 shows that 41% and 39%, respectively, of the trypanosome and *Leishmania *secretomes were not shared with any of the other proteomes. Simultaneously, secretome proteins shared with the *Trypanosoma *total proteome amounted to 47% and 43% for *Trypanosoma *and *Leishmania*, respectively, indicating that a major part of these secretomes resulted from an active secretion process.

**Figure 6:**
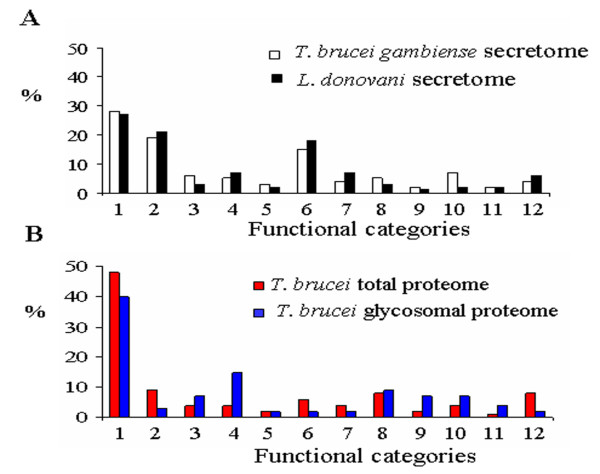
**Secretomes of *T. brucei gambiense *and *L. donovanii *share functional homology**. Functional categories from *T. brucei gambiense *and *L. donovanii *secretomes were compared (A). Proteins from *T. brucei *total proteome and glycosome were also classified into functional categories (B). On the x-axis, the categories are the following: 1. unassigned function, 2. folding and degradation, 3. nucleotide metabolism, 4. carbohydrate metabolism, 5. amino acid metabolism, 6. protein synthesis, 7. signaling, 8. cell cycle and organization, 9. lipid and cofactor, 10. transport, 11. redox, and 12. RNA/DNA metabolism. The y-axis shows the percentage of each category for each proteome/secretome.

Finally, these different proteomes were compared at the functional level (Figure 6; additional files 1, 2, 3, 4 and 5, Tables S1-S5). Interestingly, the two secretomes showed large similarities with almost the same proportion of proteins involved in folding and degradation and protein synthesis or with unassigned function. In contrast, the comparison between secretomes and glycosome functional categories showed major differences, the glycosome proteome displaying an expected bias toward sugar (15%) and lipid metabolism (7%) and, more surprisingly, toward nucleotide metabolism (7%). Also, the total proteome differed from all sub-proteomes by a deeper investment in cell organization and RNA/DNA metabolism. Almost half of the total proteome is functionally unassigned.

In summary, comparison of both the protein accessions and the functional categories similarly demonstrated features specific to the different compartments, and a close relationship between the secretome of *Trypanosoma *and *Leishmania*.

### How are *Trypanosoma *proteins secreted?

#### 1- Secreted proteins do not contain a transit peptide

If trypanosomes use the classical secretion pathway, most secreted proteins should carry an N-terminal extension (transit peptide). SignalP is currently the most popular software for predicting the presence of a N-terminal transit peptide and the associated cleavage site [21]. We performed a genome-wide screen of the *Trypanosoma *proteome using SignalP and identified 1445 proteins as predicted to contain a transit peptide (see additional file 6, Table S6), 61% without any known function. Of the remaining 561 proteins, many were known to be secreted or located at the plasma membrane, including 128 VSGs, 16 invariant surface proteins (ISG), 15 procyclin surface proteins, 14 bloodstream stage alanine-rich surface proteins (BARPs), 36 receptors for adenylate cyclase (GRESAGs), 28 transporters, 13 cysteine peptidases/clan CA/family C1 and family C2, seven transialidases, and many enzymes involved in lipid modification, glycosylation, and GPI (Glycosylphosphatidylinositol) anchoring. To focus specifically on the secreted proteins, i.e., proteins with no transmembrane span, we further assessed the occurrence of such domains using the transmembrane predictor TMHMM (transmembrane protein topology with a Hidden Markov Model) [22]. 660 proteins were simultaneously predicted to contain a transit peptide by SignalP and not to contain transmembrane domains by TMHMM. Quite unexpectedly, only 30 out of the 444 secretome proteins experimentally identified in this work belonged to the predicted secretome.

Although not secreted by the classical secretory pathway, proteins devoid of an N-terminal signal peptide may still be secreted. We used the SecretomeP software [23] to predict such proteins in the *Trypanosoma *genome (additional file 6, Table S6). Depending on the selected threshold score, different proportions of known proteins and proteins having unassigned functions were computed. A score between 0.8 and 0.9 was taken as a convenient trade-off between sensitivity and specificity, so that enough known proteins were identified for validation and avoiding obvious false predictions. In this way, 583 proteins were predicted as secreted, 79% of which had unassigned functions. Of the remaining 125 proteins, 18 transporters were found, as well as three procyclins. However, only 13 proteins from this set were found to match our experimental data.

Thus, taken together, less than 20% of the secreted proteins from our data set were predicted to have a transit peptide (SignalP) and no transmembrane domain (TMHMM) or to be secreted via the nonclassical pathway (SecretomeP), suggesting that most *Trypanosoma *secreted proteins purified so far are secreted by a novel mechanism.

#### 2-Possible exocytosis of microvesicles

**Figure 7:**
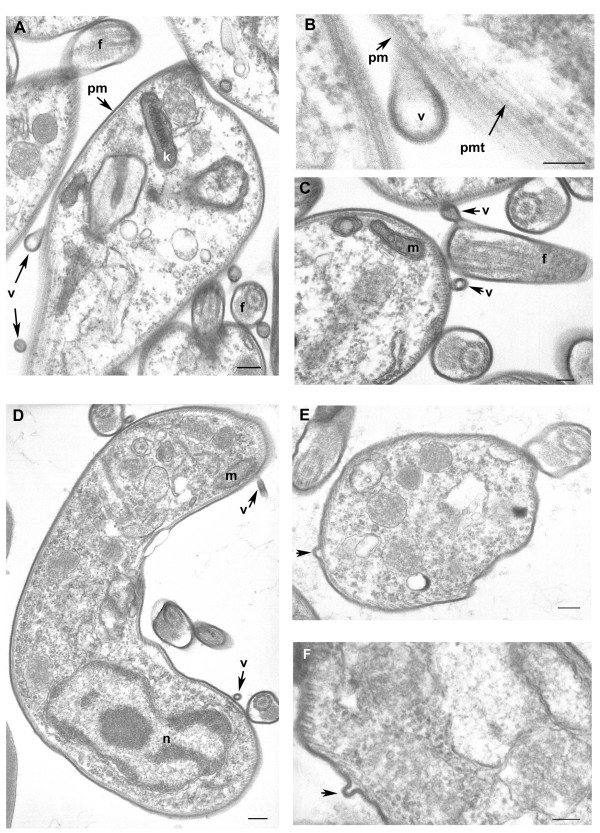
**Cross-sections of *Trypanosoma brucei gambiense *purified from infected rat blood by chromatography on DEAE cellulose column and incubation in secretion medium (A, B, C) and directly after cardiac puncture of infected rat (D, E, F)**. A-C: parasites purified from secretion medium; D-F: parasites purified directly from infected rat blood. A-F: Free vesicles and budding of new vesicles at the coated plasma membrane surface of the parasite, high magnification of vesicle formation (B), budding vesicles at the flagellum (semi-longitudinal section) (C). *f *flagellum, *k *kinetoplast, *m *mitochondrion, *n *nucleus, *pm *plasma membrane with surface coat, *pmt *pellicular microtubules, *v *vesicle. Scale bars A, D, E 200 nm, B, C, F 100 nm.

In *Trypanosoma*, endocytosis and exocytosis occur through a sequestered organelle called the flagellar pocket (FP), an invagination of the pellicular membrane. This traffic is not fully understood and requires clathrin, actin, and GTPase Rab proteins [24-26]. We found these proteins in the secretome but electronic microscopy pictures clearly indicate a budding of microvesicles at the plasma membrane and flagellum (Figure 7). In human, many types of cells, such as reticulocytes, dendritic cells, tumor cells, neurones, or mast cells, are able to release microvesicles called exosomes. Cross-correlation between different exosome proteomics studies recently identified a set of 22 proteins commonly associated with exosomes of various origins [27]. Of these, 13 were found in our data set (clathrin heavy chain, ubiquitin, *14-3-3 *proteins, hsp70 and 90, enolase, RAB protein, GAPDH [glyceraldehyde-3-phosphate dehydrogenase], pyruvate kinase, cyclophilin, tubulin α and β, and histone). Moreover, translationally controlled tumor protein (TCTP) was also shown to be present in small secreted vesicles called exosomes, and participates in inflammatory responses by promoting the release of histamine [28,29]. We found this protein in both the procyclic (data not shown) and bloodstream form of the *T. brucei gambiense *secretome (see additional file 1, Table S1).

We also looked at proteins specifically involved in endocytosis/exosome formation, and identified nine proteins, including ubiquitin (which serves as an important sorting signal during endocytosis and early endosomal protein trafficking [30]), clathrin heavy chain (involved in vesicle traffic [31]), a dynamin (a GTPase involved in the fission of clathrin-coated vesicles [32]), two adaptins (one of the major proteins of clathrin-coated vesicles [33]), two RAB proteins (small GTPases playing an essential role in the regulation of membrane traffic [34,35]), a protein called CAP (cyclase-associated protein) (implicated in vesicle trafficking and endocytosis [36]), and Hsc70 (a chaperone regulating the disassembly of clathrin coats and participating in the translocation of cytoplasmic protein substrates into a subset of lysosome-like organelles [37]).

Finally, a modest proportion (~5%) of secreted proteins found in this study contains at least one predicted transmembrane span (TMHMM), supporting the idea that vesicles are present in the sample. Thus, our secretome data support the hypothesis that *Trypanosoma *could use microvesicles to secrete proteins.

**Figure 8:**
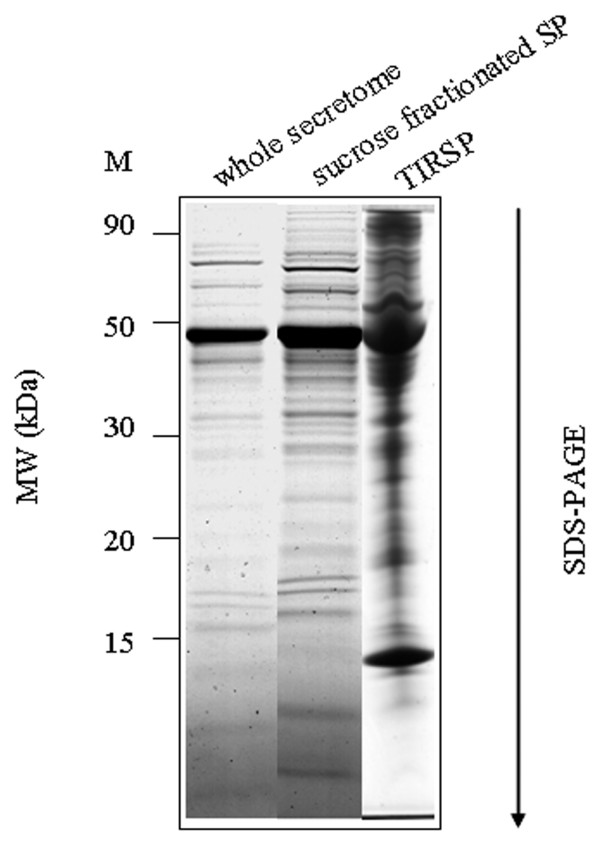
**Protein profile from the sucrose-fractionated SP and from the whole secretome**. Coomassie blue-stained SDS-PAGE (sodium dodecyl sulfate-polyacrylamide gel electrophoresis) gel showing (from left to right) marker (M), whole secretome, sucrose-fractionated SP and TIRSP (*Trypanosoma *infected rat serum). (TIRSP also contains rat proteins).

**Figure 9:**
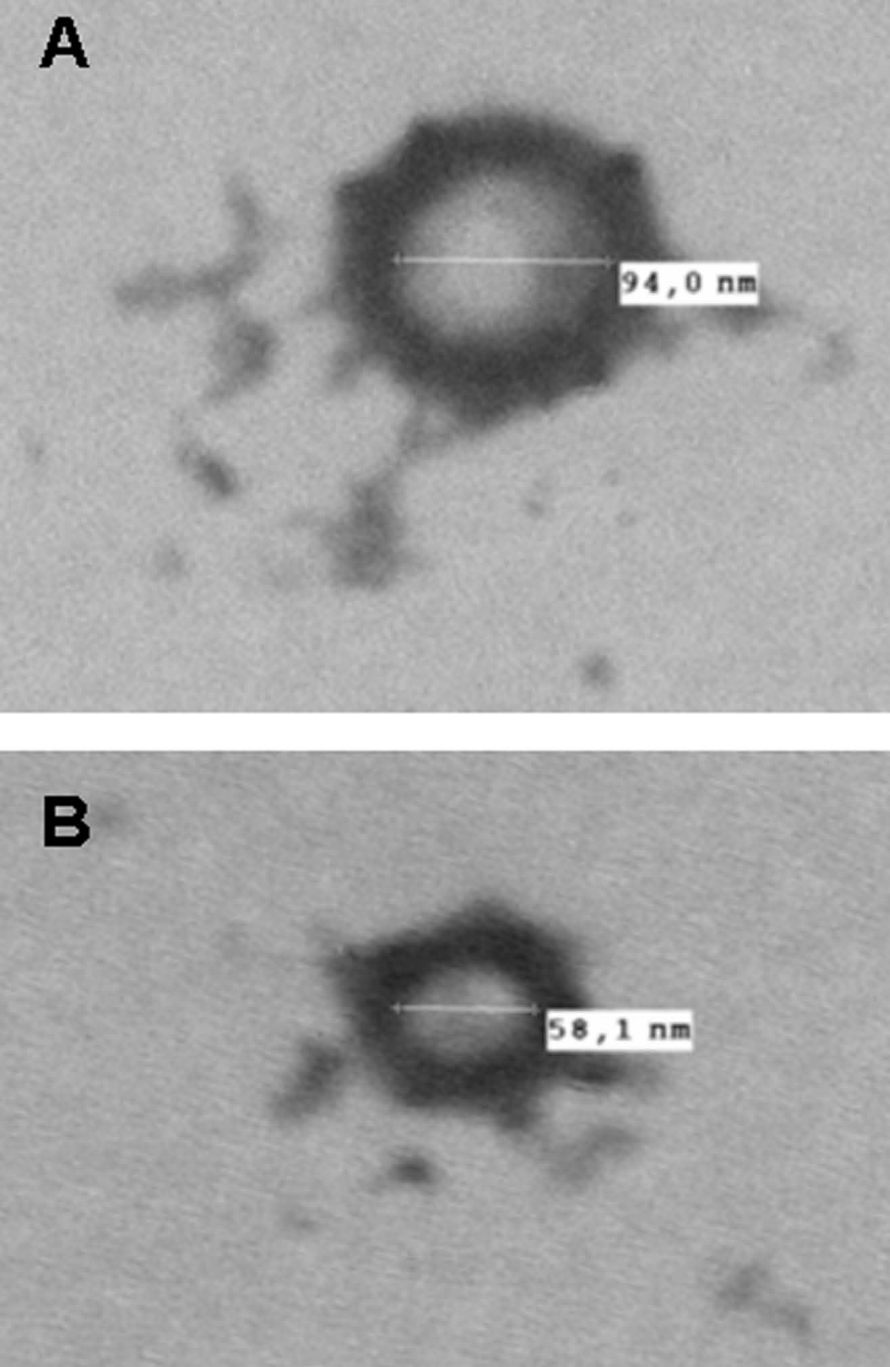
**Negative stain of vesicle-like structure found in the secretion buffer (A) and in the rat serum (B) after *Trypanosoma *depletion**. Microvesicles are typically 50-100 nm.

This hypothesis was reinforced by electron microscopic observation showing microvesicles budding at the surface of trypanosome plasma membrane. These vesicles were observed from parasites incubated in secretion medium as well as from parasites directly isolated from the blood of infected rat (Figure 7). To further verify the putative nature of the vesicles present in the sample, a 140,000 g centrifuged pellet fraction from the secretome (SP) and from *Trypanosoma*-infected rat serum (TIRSP) was layered on a step sucrose cushion (0.6-0.9-1.2-1.75 M sucrose). Sucrose-fractionated vesicles harvested at the 0.6- to 0.9-M, 0.9- to 1.2-M, and 1.2- to 1.75-M interfaces were pooled together, run on 1D gel, and analyzed by LC-MS/MS. Interestingly, the protein profile from sucrose-fractionated SP was nearly identical to the whole secretome profile (Figure 8). In addition, 65 *Trypanosoma *proteins were identified in the sucrose-fractionated TIRSP (additional file 7, Table S7) and were compared to the list of 444 ESPs identified previously. Table S7 highlights the similarity in both membrane fractions of TIRSP and ESPs (yellow boxes), suggesting a close relationship between the rat serum pellet and *Trypanosoma*-secreted proteins. Moreover, 40% of these 46 proteins (orange boxes) have already been identified in other exosome proteomics studies [27]. One can note that rat proteins were identified in this sample when specific searches were done but are not reported here. Membranes from SP and TIRSP were visualized by electron microscopy: 50- to 100-nm vesicle-like structures were observed (Figure 9).

## Discussion

### The secretome of *Trypanosoma *displays unique features

In this study, we combined different proteomics approaches, resulting in the identification of a total of 444 proteins excreted or secreted by *T. brucei gambiense*. These data make up the largest set of secreted proteins characterized to date in *Trypanosoma *and identify a specific pattern of functional categories that differs from the total proteome and from specific subcellular compartments such as the glycosome. In addition, this functional distribution is not a special case, but is shared by different strains covering the two subgroups of *T. brucei gambiense*. Thus, ESPs may be used as a general identifier of the *Trypanosoma *strains. The analysis of native proteins shows that many of them are in multiprotein complexes and form heteroligomers, again suggesting that this specific set of proteins is functional. Furthermore, in some cases, a different or original oligomeric status is observed. Taken together, these data strongly suggest that ESPs are not simply a population of unrelated proteins, but are a functionally oriented set of active proteins. Finally, genome-wide bioinformatics shows that although a number of *Trypanosoma *proteins are predicted to be secreted, few ESPs possess a transit peptide and most probably use a nonclassical secretion pathway. Thus, several lines of evidence converge to identify the *Trypanosoma *secretome as an original proteome, showing unique features both in terms of function and origin. It is noteworthy that some of the characteristics above, including the function of proteins and the absence of a transit peptide, were recently observed in the *Leishmania *secretome. This raises the question as to whether these features reveal a generic trait and whether the two parasites share common survival strategies.

### Function of secreted proteins

Our results showed that most ESPs delineate a quite limited set of functions. Generally speaking, the functions identified are not unexpected given the known physiology of *Trypanosoma *and the parasite's requirement for defense mechanisms against its host. However, for a number of proteins, previous evidence exists that they may also have other roles. Below we discuss a few examples.

*Proteins involved in folding and degradation *constitute a major class of proteins of the secretome, with more than 74 accessions identified here. Among proteins involved in folding, and shown here for the first time to be secreted by *Trypanosoma*, are cyclophilin A and hsp (heat shock protein). Interestingly, these proteins, when secreted, are known to be able to modulate the immune system of mammalian hosts [38,39], to stimulate macrophages [40], or to act as mediators for intercellular signaling [39]. In addition, the secretome appears to be unexpectedly rich in various peptidases, covering more than ten peptidase families or subfamilies (Table 1), some of which are known to be secreted. Several might play a role in the pathology. For instance, we identified two oligopeptidases. One (prolyl-oligopeptidase) was previously shown to be secreted by *T. cruzi *[41] and presumed to facilitate the infection of host cells by degrading the collagen of the extracellular matrix. Oligopeptidase B is secreted from *T. brucei *and *T. congolense *[42,43]. This enzyme is able to cleave host peptide hormones such as atrial natriuretic factor [44], thus contributing to the increase in blood volume [45] and possibly to the disruption of the blood-brain barrier [46], both associated with the infection. Other symptoms of trypanosomiasis, such as the perturbation of the endocrine rhythms [47], could also involve oligopeptidase B. More generally, it can be speculated that oligopeptidases, by cleaving regulatory peptides, could play pleiotropic roles in the pathogenic process developed during HAT. In contrast, for M20-M25-M40 and M17 family peptidases, where evidence also exists for their secretion by other organisms [48,49], the identification in the secretome of *Trypanosoma *is novel and it is too early to speculate on its functions.

**Table 1:** Diversity of peptidase families found in the secretome of *T. brucei gambiense *bloodstream form and their distribution in other organisms.

Families of Peptidases	Distribution
Serine peptidase family S9	Bacteria, Archaea, Protozoa, Fungi, Plants, Animals, Viruse
	
Cysteine peptidase family C2	Bacteria, ----------, Protozoa, Fungi, Plants, Animals, --------
Cysteine peptidase family C13	Bacteria, Archaea, Protozoa, Fungi, Plants, Animals, --------
Cysteine peptidase family C19	Bacteria, ----------, Protozoa, Fungi, Plants, Animals, Viruse
	
Metallo-peptidase family M1	Bacteria, Archaea, Protozoa, Fungi, Plants, Animals, --------
Metallo-peptidase family M3	Bacteria, Archaea, Protozoa, Fungi, Plants, Animals, --------
Metallo-peptidase family M16	Bacteria, Archaea, Protozoa, Fungi, Plants, Animals, Viruse
Metallo-peptidase family M17	Bacteria, Archaea, Protozoa, Fungi, Plants, Animals, --------
Metallo-peptidase family M20	Bacteria, Archaea, Protozoa, Fungi, Plants, Animals, --------
Metallo-peptidase family M24	Bacteria, Archaea, Protozoa, Fungi, Plants, Animals, --------
Metallo-peptidase family M32	Bacteria, Archaea, Protozoa, -------, Plants, ----------, --------

Among metallopeptidases, the thimet oligopeptidase A is the first member of the M3 family to be identified in *Protozoa*. Thus, this protease, which processes neuropeptides in humans [50], may be a good candidate for a specific diagnostic marker. Another metallopeptidase in the secretome belongs to the M32 family, absent in eukaryotic genomes other than trypanosomatids [51]. Although of unknown biological function, it might offer attractive drug targets against *Trypanosoma*.

Proteins involved in folding and degradation are the major class of secreted proteins. Apart from contributing to protecting the parasite against the defense mechanisms of the host, many of them also appear to have the capacity to induce perturbations in the host physiology. Given their abundance, one may speculate that they play a genuine role in the pathology. Some of these proteins may be promising candidates for diagnosis or therapy. As well as degrading proteins, proteases perform highly specific processing tasks that can affect protein structure, function, life span, and localization. By limited and specific cleavage, proteases can act as switches, turning protein activity on or off, or can modulate protein function in more complex ways, regulating vital processes. Indeed, more than 53 specific hereditary diseases of proteolysis are recognized and it is therefore not surprising that proteases are implicated in many pathologies. Hence, proteases account for 5-10% of drug targets, with protease inhibitor drugs already in use to treat AIDS (acquired immunodeficiency syndrome) by blocking HIV (human immunodeficiency virus) protease-1, cardiovascular disease by targeting angiotensin convertase enzyme and rennin, and multiple myeloma by the reversible covalent proteasome inhibitor. In addition, many biomarkers of disease, especially in cancer, are stable fragments generated by proteolysis and found in biological fluids [52].

*Enzymes of nucleotide metabolism *are another major class of ESPs represented here by more than 46 protein accessions. This is not unexpected, as *T. brucei *is incapable of *de novo *purine nucleotide synthesis and expresses purine salvage enzymes to recover host purines [53]. However, extracellular nucleotides are also signaling molecules that modulate a wide variety of physiological responses in mammalian tissues [54] and are archetypal activators of the innate immune system [55]. In this context, both hematophagous insects and endoparasites secrete enzymes degrading nucleotides, thus minimizing inflammatory reactions or purinergic signaling provoked by these mediators [56,57]. As such, the identification of several nucleotide-metabolizing enzymes in the secretome raises the question of whether *T. brucei *might exploit such strategies to modulate the concentration of extracellular nucleotides, hence affecting a range of inflammatory responses. If so, *Trypanosoma *would not only divert the host nucleotides for its own requirements, but also to evade an immune response.

*Enzymes involved in glycolysis and carbohydrate metabolism *are not a major class of the secretome, but this category still numbers more than 36 accessions. *Trypanosoma *have a simplified energy metabolism entirely dependent on external carbohydrate sources, such as blood glucose.

Most glycolysis enzymes are compartmented in glycosomes [58], but three are cytosolic: phosphoglycerate mutase, enolase, and pyruvate kinase [59]. We found all three in the *T. brucei *secretome, together with phosphoglycerate kinase, yet this secretome strongly differs from glycosomes. Although not yet reported as being secreted in *Trypanosoma*, all four enzymes are secreted by other organisms and may be involved in functions unrelated to glycolysis, such as peptide cleavage or immunosuppressive activity [60-63]. Therefore, it cannot be excluded that *Trypanosoma *might use part of its energy metabolism machinery for alternative purposes.

*Enzymes involved in signaling *constitute another group of proteins identified in the *T. brucei *secretome (26 accessions), and some could also play physiopathological roles. Two notable examples here concern calreticulin (CRT) and prostaglandin F (PGF) synthase. Autoantibodies against CRT are found in the sera of human hosts of a number of parasitic diseases [64] and it was suggested that the parasite-derived CRT could trigger an inappropriate immune response against self-antigens through molecular mimicry [65]. The gene encoding PGF synthase is present in *T. brucei*, and we show that this enzyme can be secreted. Given that African trypanosomiasis is characterized by miscarriage, due to PGF overproduction correlated with parasitemia peaks [66], the finding that *T. brucei *secretes a PGF synthase suggests that this enzyme may well play a role in pathogenesis.

One trivial role of protein secretion in hosts is usually associated with trophic purposes for the benefit of the parasites. Several recent proteomics studies highlighted other features, depending on the parasite. For instance, whereas for *Brugia malayi *a large fraction of the 80 proteins found to be secreted are involved in energy metabolism [67], for the helminth *Schistosoma mansoni *the 188 proteins identified include proteins involved in metabolic pathways and in protein folding, development, and signaling, or immune response modulation [68], and the secretome of *Plasmodium falciparum *is predicted to encompass several hundred proteins to both import nutrients and remodel the host erythrocyte [69]. In this work, the 444 identified *T. brucei*-secreted proteins display a specific pattern and, for a number of these, there is evidence for possible alternative functions. The various examples detailed above support the hypothesis that, far from being fortuitous, these features probably reveal an additional role for the secretome in manipulating the host in order to overcome its defenses. As such, the secretome would also play a key role in the pathogenicity of the parasite. More generally, this suggests that, apart from the production of VSGs to elude the host immune system, the secretome might also be another authentic component of the survival strategy of *Trypanosoma*.

### Origin and significance of the identified secretome for the survival strategy of *Trypanosoma*

Only a minority of ESPs appear to possess a transit peptide, raising the question of the nature of the secretory pathway in *Trypanosoma*. Several arguments support the hypothesis that secretion could take place by the release of microvesicles. Such small vesicles contain cytosol and proteins from the plasma membrane, but no protein from intracellular organelles. On the one hand, apart from the nine proteins specifically involved in vesicle transport and trafficking, we identified 13 out of the 22 most common exosome proteins. On the other hand, we found very few proteins from intracellular compartments (1% of the secretome). The viability of *Trypanosoma *tested with flow cytofluorometric analysis and microscopic analysis suggests that the nonspecific release of material from lysed cells is modest and the yield of secreted proteins is not correlated to viability but is strain-specific (for example, Biyamina produced 4 times less secreted proteins than other strains). Moreover, the comparison between the total proteome and the secretome showed in this study also suggests that contaminations from nonspecific release would be relevant only if the kinetics of release is highly protein-specific. In addition, ubiquitin seems to play a key role in the sorting of proteins into exosomes [70], and we identified ubiquitin and 25 related proteins of the ubiquitin/proteasome pathway. Thus, the overall picture of the *Trypanosoma *secretome shows homologies with exocytosis occurring in the flagellar pocket and with exosome-related proteomes. Interestingly, we have successfully demonstrated for the first time the presence of vesicles at the trypanosome surface using electron microscopy and further shown that similar vesicles are present in the secretion medium. Moreover, proteomic analysis of TIRSP confirmed the presence of a set of proteins that is very similar (71%, 46/65) to ESPs purified from isolated parasites. Thus, both approaches converge to strengthen the hypothesis of a new secretion pathway in *Trypanosoma*. Indeed, the size of the vesicle-like structure observed on electronic microscopy pictures fits with microvesicles (50-100 nm). This situation seems to be shared with *Leishmania*, a close relative of *Trypanosoma*, where the absence of transit peptides in secreted proteins and the presence of microvesicles at the promastigote surface were recently demonstrated [20]. This differs from the case of *P. falciparum*, where a specific host-targeting motif was described for secreted proteins [71].

This could be hypothesized to present several advantages for *Trypanosoma*, in comparison to the classical secretory pathway: it may deliver an avalanche of new epitopes to overwhelm the host immune system or to communicate between trypanosomes themselves by exchanging receptors in the form of non-protein cytosolic compounds or even potentially genomic information. As such, microvesicles could be a flexible way for *Trypanosoma *to reversibly adapt its machinery and to homogenize the survival strategy at the population level.

## Conclusions

This study provides the first overview of proteins secreted by *Trypanosoma brucei*. Several of them had not yet been discovered and studied in trypanosomes, and some may be new potential therapeutic targets or diagnostic markers. Strikingly, some proteins do not use the classical secretory pathway and many probably play additional roles once secreted. Collectively, these data lead to novel hypotheses concerning both the pathogenic role of secreted proteins and the secretion pathway in trypanosomatids, providing insight into the complex survival strategy of *T. brucei*.

## Methods

Ethical statement: all the experiments on animals reported in this article were performed according to internationally recognized guidelines; the experimental protocols were approved by the Ethical Committee on Animal Experiments and the Veterinary Department of the Centre International de Recherche Agronomique pour le Développement (CIRAD), Montpellier-France. No experiment was performed on human.

### Rats

Male Wistar rats (6-12 weeks old) were purchased from Charles Rivers (France).

### Parasites

Feo [72,73], OK [73] and Biyamina [74] parasite bloodstream strains were used for the experiment. The parasites were intraperitoneally injected into rats. When their multiplication reached the logarithmic growth stage, the parasites were purified from blood by chromatography on a DEAE (diethylaminoethyl) cellulose column, as previously described [75]. After elution, the parasites were washed three times in sterile phosphate-buffered saline (PBS) solution. This resulted in a complete elimination of the rat blood proteins.

### Excreted/secreted protein (ESP) production

The parasites were resuspended at a concentration of 200.10^6 ^cells/ml in a secretion buffer (Ringer lactate, glucose 0.6%, Kcl 0.4%, NaHCO3 0.125%, polymixin B 5 μg/ml, L-glutamine 2 mM, MEM nonessential amino acids, pH 8) [76]. The secretion of ESPs was performed at 37°C/5% CO_2 _for 2 h. At the end of the experiment, the reaction was stopped by centrifugation of parasites at 4°C, 1000 g for 10 min. The supernatant was collected and filtered on a 0.2-μm filter and immediately mixed with a protease inhibitor mix. ESPs were concentrated by ultrafiltration using a PM - 10-kDa membrane (Amicon). The protein concentration was determined by the Bradford dye binding procedure (Bio-Rad). Concentrated ESPs were analyzed further by SDS- and BN-PAGE and visualized after staining with coomassie blue.

### Apoptosis assay

The percentage of apoptotic parasites was quantitated every 15 min by flow cytofluorometric analysis using the DNA intercalant propidium iodide (IP), as recommended by the manufacturer (Immunotech, Marseille, France). Cells were immediately analyzed with a FACScan (fluorescence-activated cell sorting) flow cytometer (Becton Dickinson, Ivry, France) using an argon-ion laser. Parasite viability, determined every 15 min, remained constant for 2 h and was more than 95%. Moreover, cellular integrity was controlled by microscopic examination of aliquots of the incubation medium during the 2-h period of trypanosome incubation.

### One-dimensional electrophoretic analysis

Proteins from the different samples were heated at 100°C for 2 min and spun for 5 min at 14,000 g prior to separation by one-dimensional SDS-PAGE. Proteins were separated on 24 × 18-cm Tricine/SDS-PAGE (12% acrylamide) [77].

After migration, the gels were fixed, and the proteins were visualized by coomassie brilliant blue R-250. Images of the gels were taken with a high-resolution scanner (Amersham Biosciences).

### BN-PAGE

Proteins were concentrated and directly loaded on native PAGE gradients 6-15% acrylamide for the first dimension and on a 12% Tricine-SDS-PAGE for the second dimension, as described in Peltier et al., 2004 [78]. Proteins were visualized by coomassie blue staining.

### Protein identification by mass spectrometry

Stained protein spots were manually excised, washed, digested with trypsin, and extracted using formic acid. Protein digests were analyzed using either a hybrid triple-quadrupole linear ion trap mass spectrometer (Q-TRAP 4000; Applied Biosystems), coupled to a nano-chromatography system (Dionex), or an ion trap mass spectrometer (Esquire HCT; Bruker), interfaced with an HPLC (high-performance liquid chromatography) chip system (Agilent). MS/MS data were searched against NCBI (National Center for Biotechnology Information) and *Trypanosoma brucei *databases using Mascot software. Raw data were analyzed using Data Analysis software (Bruker) to generate a peak list for searching a *Trypanosoma *database extracted from the Sanger Institute. The Mascot (v2.2) search engine was used with the following parameters: one missed cleavage allowed for trypsin, carboxymethylation of cyst as fixed modification, methionine oxidation as variable modification, and a 0.6-Da tolerance range for mass accuracy in MS/MS. At least one matching sequence tags of high quality was needed for positive identification of proteins. Potential false positive identifications have been addressed as described in Elias et al., 2005 [79] using identical search parameters against a database in which the sequences have been reversed. We set a false discovery rate (FDR) of 1%. When the Mascot peptide score was below (and even above) the Mascot peptide score indicated for a FDR of 1%, a systematic manual validation was done with stringent parameters (at least 6 y or b ions, at least 4 consecutive ions, and peptidic sequence formed of more than 7 amino acids). The proteins were classified according to MapMan http://mapman.gabipd.org. Raw data will be made available upon request for research purposes. Additional data on identified proteins are supplied in additional file 8 (Table S8).

### Preparation of vesicles by ultracentrifugation and sucrose gradient

Secretion buffer and infected rat serum after parasite depletion were filtered (0.2 μm). Membranes were isolated from secretion buffer and serum of *Trypanosoma*-infected rats by a 140,000 g ultracentrifugation for 30 min at 4°C. Pellets were resuspended in 20 mM Tris/Hcl buffer pH 7.8 and layered on top of a step sucrose gradient (20-30-40-60% sucrose [Sigma-Aldrich]). Gradients were centrifuged for 2 h at 100,000 g at 4°C. Fractions were collected at the interface 20/30%, 30/40%, and 40/60%, diluted in 20 mM Tris-Hcl buffer pH 7.8, and pelleted (140,000 g for 30 min at 4°C). Pellets were resuspended in Tris buffer and loaded on SDS-PAGE. After staining protein bands were picked and washed and proteins were trypsinated. Peptides were analyzed by LC-MS/MS on an orbitrap.

### Transmission electron microscopy

#### Sample preparation for ultrastructure

Observations on whole trypanosomes obtained after incubation in secretion medium or directly from blood of infected rat were conducted as described previously [80]. After centrifugation, pellets of trypanosomes were fixed in 4% glutaraldehyde in 0.1 M cacodylate buffer (pH 7.2) overnight at 4°C, washed in fixation buffer, postfixed in osmium tetroxide for 1 h at 4°C and washed. Pellets were dehydrated in an acetone series and embedded in TAAB 812 epon resin [80].

Thin sections, mounted on 75 mesh collodion carbon-coated copper grids, were contrasted with uranyl acetate and lead citrate and examined at 80 KV with a transmission electron microscope (Jeol 100CX II).

#### Negative stain

The stains were prepared according to Brun et al., 2008 [81]. Basically, 2-3 μl of sample (vesicles obtained by ultracentrifugation and sucrose gradient) were layered onto a 200-formvar-coated grids for 1 min. Liquid was remove with filter paper. The grid was incubated with a negative stain (1.5% uranylacetate in 70% ethanol for 1 min) and washed 3 times with water. The preparation was observed with a Hitachi H. 7100 transmission electron microscope.

## Abbreviations

AIDS: acquired immunodeficiency syndrome; BN-SDS-PAGE: blue native-sodium dodecyl sulfate-polyacrylamide gel electrophoresis; BLAST: Basic Local Alignment Search Tool; BARP: bloodstream stage alanine-rich surface protein; CAP: cyclase-associated protein; CRT: calreticulin; DNA: deoxyribonucleic acid; DEAE: diethylaminoethyl; ESPs: expressed/secreted proteins; FACS: fluorescence-activated cell sorting; GRESAGs: receptor for adenylate cyclase; GPI: glycosylphosphatidylinositol; GAPDH: glyceraldehyde-3-phosphate dehydrogenase; HAT: human African trypanosomiasis; Hsp: heat shock protein; HIV: human immunodeficiency virus; HPLC: high performance liquid chromatography; IP: propidium iodide; ISG: invariant surface glycoprotein; LC-ESI MS/MS: liquid chromatography-electrospray tandem mass spectrometry; MW: molecular weight; NCBI: National Center for Biotechnology Information; PA26: proteasome activator protein with a molecular mass of 26-kDa; PGF: prostaglandin F; RNA: ribonucleic acid; SP: secretome; TMHMM: transmembrane protein topology with a Hidden Markov Model; TCTP: translationally controlled tumor protein; TIRSP: *Trypanosoma *infected rat serum; VSG: variant surface glycoprotein

## Competing interests

The authors declare that they have no competing interests.

## Authors' contributions

AG, GC, and J-BP designed the research; AG, CH, TB, EB, DC, DG, and J-BP carried out the experiment; AG and J-BP analyzed the data; and AG, MR, and J-BP wrote the paper. All authors read and approved the final manuscript.

## Supplementary Material

Additional file 1 **Table S1. Secreted proteins identified in 3 *T. brucei gambiense *strains separated on 1D gel**. contains the identification of the proteins secreted by Biyamina (sheet 1), Feo (sheet 2), and OK strain (sheet 3) and their classification according to functional categories (MapMan bins nomenclature). For each protein, the number of matched peptides and the highest score are described. Click here for file

Additional file 2 **Table S2. Secreted proteins from OK strain identified on BN-PAGE gel**. contains the list of the proteins identified in each spot (sheet 1), a nonredundant list of proteins classified according to their functional categories (sheet 2), and a nonredundant list of all the secreted proteins identified (BN+1D) in this study (sheet 3). Click here for file

Additional file 3 **Table S3. Secreted proteins from *Leishmania donovanii *and their corresponding *Trypanosoma *orthologs**. contains the list of 358 proteins from *L. donovanii *identified in Silverman et al., 2008 [20] which were blasted against the *T. brucei *genome. The blast e scores > e^-50 ^were reported as positive identification of *T. brucei *orthologs. Functional categories were assigned to *L. donovanii-*secreted proteins as well as the transmembrane span prediction (TMHMM) of these proteins. Click here for file

Additional file 4 **Table S4. Proteins identified in glycosome from *T. brucei ***[19]. contains the list of 163 proteins from the glycosome proteome which were classified into functional categories (MapMan bins nomenclature). Click here for file

Additional file 5 **Table S5. Proteins identified in total proteome from *T. brucei ***[18]. contains the list of 1071 proteins from the total proteome which were classified into functional categories (MapMan bins nomenclature). Click here for file

Additional file 6 **Table S6. Genome-wide prediction of secreted proteins using SignalP and secretomeP**. contains the list of 1445 SignalP-predicted proteins (containing a putative transit peptide) from *T. brucei *and classified according to the number of predicted transmembrane spans (TMHMM prediction) (sheet 1). SecretomeP-predicted proteins from *T. brucei *were reported according to their p-value (sheet 2). The 3 highest classes p>0.9, 0.9>p>0.8, and 0.8>p>0.7 containing, respectively, 128, 583, and 875 proteins and their number of predicted transmembrane spans (TMHMM prediction) were reported. Click here for file

Additional file 7 **Table S7. Proteins identified in sucrose fractionated membranes from infected rat serum (IRS)**. contains the list of the IRS proteins. IRS proteins shared with ESPs or exosome are boxed in yellow and orange, respectively. Click here for file

Additional file 8 **Table S8. Additional informations on proteins identified in secretome**. contains the list of the proteins identified in 1D and BN-PAGE gels spots. Protein score, number of peptides identified and number of peptides that fit to our stringent filter are provided. Click here for file

## References

[B1] Robinson NP Burman N Melville SE Barry JD Predominance of duplicative VSG gene conversion in antigenic variation in African trypanosomes Mol Cell Biol 1999 19 5839 46 10454531 10.1128/mcb.19.9.5839 PMC84433

[B2] Dubois ME Demick KP Mansfield JM Trypanosomes expressing a mosaic variant surface glycoprotein coat escape early detection by the immune system Infect Immun 2005 73 2690 7 10.1128/IAI.73.5.2690-2697.2005 15845470 PMC1087325

[B3] MacGregor P Matthews KR Modelling trypanosome chronicity: VSG dynasties and parasite density Trends Parasitol 2008 24 1 4 10.1016/j.pt.2007.09.006 18024198 PMC2855958

[B4] WHO Human African Trypanosomiasis (sleeping sickness): epidemiological update Wkly Epidemiol Rec 2006 81 71 80 16673459

[B5] Stich A Abel PM Krishna S Human African Trypanosomiasis Br Med J 2002 325 203 206 10.1136/bmj.325.7357.203 PMC1123723 12142311

[B6] Pépin J Milord F The treatment of human African trypanosomiasis Adv Parasitol 1994 33 1 47 full_text 8122565 10.1016/s0065-308x(08)60410-8

[B7] Legros D Ollivier G Gastellu-Etchegorry M Paquet C Burri C Jannin J Buscher P Treatment of human African trypanosomiasis - present situation and needs for research and development Lancet Infect Dis 2002 2 437 440 10.1016/S1473-3099(02)00321-3 12127356

[B8] Okenu DMN Opara KN Nwuba RI Nwagwu M Purification and characterisation of an extracellular released protease of *Trypanosoma brucei* Parasitol Res 1999 85 424 428 10.1007/s004360050571 10227063

[B9] Lonsdale-Eccles JD Grab DJ Trypanosome hydrolase and the blood-brain barrier Trends Parasitol 2002 18 17 19 10.1016/S1471-4922(01)02120-1 11850009

[B10] Girard M Bisser S Courtioux B Vermot-Desroches C Bouteille B Wijdenes J Preud'homme JL Janberteau MO *In vitro *induction of microglial and endothelial cell apoptosis by cerebrospinal fluids from patients with human African trypanosomiasis Int J Parasitol 2003 33 713 720 10.1016/S0020-7519(03)00033-X 12814651

[B11] Garzon E Geiger A Totte P Regnier C Cuny G Dedieu L *Trypanosoma brucei *secrete factors able to inhibit dendritic cells maturation and their ability to induce lymphocytic allogenic responses Infectiology VII Meeting 2006 S171 COL1-SFP

[B12] Gibson WC Backhouse T Griffiths A The human serum resistance associated gene is ubiquitous and conserved in *Trypanosoma brucei rhodesiense *throughout East Africa Inf Genet Evol 2002 1 207 214 10.1016/S1567-1348(02)00028-X 12798017

[B13] Thimm O Bläsing O Gibon Y Nagel A Meyer S Krüger P Selbig J Müller LA Rhee SY Stitt M MAPMAN: a user-driven tool to display genomics data sets onto diagrams of metabolic pathways and other biological processes Plant J 2004 37 914 39 10.1111/j.1365-313X.2004.02016.x 14996223

[B14] Schägger H Cramer WA von Jagow G Analysis of molecular masses and oligomeric states of protein complexes by blue native electrophoresis and isolation of membrane protein complexes by two-dimensional native electrophoresis Anal Biochem 1994 217 220 30 10.1006/abio.1994.1112 8203750

[B15] Herrera-Camacho I Rosas-Murrieta NH Rojo-Dominguez A Millàn L Reyes-Leyva J Santos-Lopez G Suarez-Rendueles Biochemical characterization and structural prediction of a novel cytosolic leucyl aminopeptidase of the M17 family from *Schizosaccharomyces pombe* FEBS J 2007 274 6228 40 10.1111/j.1742-4658.2007.06142.x 18028193

[B16] To WY Wang CC Identification and characterization of an activated 20S proteasome in *Trypanosoma brucei* FEBS Lett 1997 404 253 62 10.1016/S0014-5793(97)00116-6 9119074

[B17] Yao Y Huang L Krutchinsky A Wong ML Standing KG Burlingame AL Wang CC Structural and functional characterizations of the proteasome-activating protein PA26 from *Trypanosoma brucei* J Biol Chem 1999 274 33921 30 10.1074/jbc.274.48.33921 10567354

[B18] Jones A Faldas A Foucher A Hunt E Tait A Wastling JM Turner CM Visualisation and analysis of proteomic data from the procyclic form of *Trypanosoma brucei* Proteomics 2006 6 259 67 10.1002/pmic.200500119 16302277

[B19] Colasante C Ellis M Ruppert T Voncken F Comparative proteomics of glycosomes from bloodstream form and procyclic culture form *Trypanosoma brucei brucei* Proteomics 2006 6 3275 93 10.1002/pmic.200500668 16622829

[B20] Silverman JM Chan SK Robinson DP Dwyer DM Nandan D Foster LJ Reiner NE Proteomic analysis of the secretome of *Leishmania donovani* Genome Biol 2008 9 R35 10.1186/gb-2008-9-2-r35 18282296 PMC2374696

[B21] Dyrløv Bendtsen J Nielsen H von Heijne G Brunak B Improved Prediction of Signal Peptides: SignalP 3.0 J Mol Biol 2004 340 783 79 10.1016/j.jmb.2004.05.028 15223320

[B22] Krogh A Larsson B von Heijne G Sonnhammer EL Predicting transmembrane protein topology with a hidden Markov model: application to complete genomes J Mol Biol 2001 305 567 80 10.1006/jmbi.2000.4315 11152613

[B23] Dyrløv Bendtsen J Jensen LJ Blom N von Heijne G Brunak S Feature-based prediction of non-classical and leaderless protein secretion Protein Engineering, Design & Selection 2004 17 349 356 10.1093/protein/gzh037 15115854

[B24] Gull K Host-parasite interactions and trypanosome morphogenesis: a flagellar pocketful of goodies Curr Opinion Microbiol 2003 6 365 370 10.1016/S1369-5274(03)00092-4 12941406

[B25] Field MC Natesan SKA Gabernet-Castello C Koumandou VL Intracellular trafficking in the Trypanosomatids Traffic 2007 8 629 639 10.1111/j.1600-0854.2007.00558.x 17461800

[B26] Garcia-Salcedo JA Perez-Morga D Gijon P Dilbeck V Pays E Nolan DP A differential role for actin during the life cycle of *Trypanosoma brucei* EMBO J 2004 23 780 789 10.1038/sj.emboj.7600094 14963487 PMC381002

[B27] Olver C Vidal M Proteomic analysis of secreted exosomes Subcell Biochem 2007 43 99 131 full_text 17953393 10.1007/978-1-4020-5943-8_7

[B28] Amzallag N Passer BJ Allanic D Segura E Théry C Goud B Amson R Telerman A TSAP6 facilitates the secretion of translationally controlled tumor protein/histamine-releasing factor via a nonclassical pathway J Biol Chem 2004 279 46104 46112 10.1074/jbc.M404850200 15319436

[B29] Lespagnol A Duflaut D Beekman C Blanc L Fiucci G Marine JC Vidal M Amson R Telerman A Exosome secretion, including the DNA damage-induced p53-dependent secretory pathway, is severely compromised in TSAP6/Steap3-null mice Cell Death Differ 2008 15 1723 1733 10.1038/cdd.2008.104 18617898

[B30] Hicke L Dunn R Regulation of membrane protein transport by ubiquitin and ubiquitin-binding proteins Annu Rev Cell Dev Biol 2003 19 141 72 10.1146/annurev.cellbio.19.110701.154617 14570567

[B31] Raiborg C Bache KG Gillooly DJ Madshus ICH Stang E Stenmark H Hrs sorts ubiquitinated proteins into clathrin-coated microdomains of early endosomes Nat Cell Biol 2002 4 394 8 10.1038/ncb791 11988743

[B32] Takei K Yoshida Y Yamada H Regulatory mechanisms of dynamin-dependent endocytosis J Biochem 2005 137 243 7 10.1093/jb/mvi052 15809324

[B33] Ritter B Blondeau F Denisov AY Gehring K McPherson PS Molecular mechanisms in clathrin-mediated membrane budding revealed through subcellular proteomics Biochem Soc Trans 2004 32 769 73 10.1042/BST0320769 15494011

[B34] Schimmöller F Simon I Pfeffer SR Rab GTPases, directors of vesicle docking J Biol Chem 1998 273 22161 4 10.1074/jbc.273.35.22161 9712825

[B35] Brennwald P Reversal of fortune. Do Rab GTPases act on the target membrane? J Cell Biol 2000 149 1 4 10.1083/jcb.149.1.1 10747079 PMC2175097

[B36] Hubberstey AV Mottillo EP Cyclase-associated proteins: CAPacity for linking signal transduction and actin polymerization FASEB J 2002 16 487 99 10.1096/fj.01-0659rev 11919151

[B37] Agarraberes FA Dice JF A molecular chaperone complex at the lysosomal membrane is required for protein translocation J Cell Sci 2001 114 2491 9 11559757 10.1242/jcs.114.13.2491

[B38] Darji A Beschin A Sileghem M Heremans H Brys L De Baetselier P *In vitro *simulation of immunosuppression caused by *Trypanosoma brucei*: active involvement of gamma interferon and tumor necrosis factor in the pathway of suppression Infect Immun 1996 64 1937 43 8675290 10.1128/iai.64.6.1937-1943.1996 PMC174019

[B39] Calderwood SK Mambula SS Gray PJ Jr Theriault JR Extracellular heat shock proteins in cell signaling FEBS Lett 2007 581 3689 94 10.1016/j.febslet.2007.04.044 17499247

[B40] Kim H Kim WJ Jeon ST Koh EM Cha HS Ahn KS Lee WH Cyclophilin A may contribute to the inflammatory processes in rheumatoid arthritis through induction of matrix degrading enzymes and inflammatory cytokines from macrophages Clin Immunol 2005 116 217 24 10.1016/j.clim.2005.05.004 15993649

[B41] Santana JM Grellier P Schrével J Teixeira AR A *Trypanosoma cruzi*-secreted 80 kDa proteinase with specificity for human collagen types I and IV Biochem J 1997 325 129 37 9224638 10.1042/bj3250129 PMC1218537

[B42] Morty RE Authié E Troeberg L Lonsdale-Eccles JD Coetzer TH Purification and characterisation of a trypsin-like serine oligopeptidase from *Trypanosoma congolense* Mol Biochem Parasitol 1999 102 145 55 10.1016/S0166-6851(99)00097-3 10477183

[B43] Morty RE Shih AY Fülöp V Andrews NW Identification of the reactive cysteine residues in oligopeptidase B from *Trypanosoma brucei* FEBS Lett 2005 579 2191 6 10.1016/j.febslet.2005.03.014 15811340

[B44] Troeberg L Pike RN Morty RE Berry RK Coetzer TH Lonsdale-Eccles JD Proteases from *Trypanosoma brucei brucei*. Purification, characterisation and interactions with host regulatory molecules Eur J Biochem 1996 238 728 36 10.1111/j.1432-1033.1996.0728w.x 8706674

[B45] Anosa VO Isoun TT Serum proteins, blood and plasma volumes in experimental *Trypanosoma vivax *infections of sheep and goats Trop Anim Health Prod 1976 8 14 9 10.1007/BF02383360 1258150

[B46] Philip KA Dascombe MJ Fraser PA Pentreath VW Blood-brain barrier damage in experimental African trypanosomiasis Ann Trop Med Parasitol 1994 88 607 16 7893174 10.1080/00034983.1994.11812911

[B47] Brandenberger G Buguet A Spiegel K Stanghellini A Muanga G Bogui P Dumas M Disruption of endocrine rhythms in sleeping sickness with preserved relationship between hormonal pulsatility and the REM-NREM sleep cycles J Biol Rhythms 1996 11 258 67 10.1177/074873049601100307 8872597

[B48] Pera EM Martinez SL Flanagan JJ Brechner M Wessely O De Robertis EM Darmin is a novel secreted protein expressed during endoderm development in *Xenopus* Gene Expr Patterns 2003 3 147 52 10.1016/S1567-133X(03)00011-5 12711541

[B49] Hatta T Kazama K Miyoshi T Umemiya R Liao M Inoue N Xuan X Tsuji N Fujisaki K Identification and characterisation of a leucine aminopeptidase from the hard tick *Haemaphysalis longicornis* Int J Parasitol 2006 36 1123 32 10.1016/j.ijpara.2006.05.010 16814790

[B50] Pierotti A Dong KW Glucksman MJ Orlowski M Roberts JL Molecular cloning and primary structure of rat testes metalloendopeptidase EC 3.4.24.15 Biochemistry 1990 29 10323 9 10.1021/bi00497a006 2261476

[B51] Niemirowicz G Parussini F Agüero F Cazzulo JJ Two metallocarboxypeptidases from the protozoan *Trypanosoma cruzi *belong to the M32 family, found so far only in prokaryotes Biochem J 2007 401 399 410 10.1042/BJ20060973 17007610 PMC1820797

[B52] Doucet A Butler GS Rodriguez D Prudova A Overall CM Quantitative degradomics analysis of proteolytic post-translational modifications of the cancer proteome Mol Cell Proteomics 2008 7 1925 1951 10.1074/mcp.R800012-MCP200 18596063

[B53] Pellé R Schramm VL Parkin DW Molecular cloning and expression of a purine-specific N-ribohydrolase from *Trypanosoma brucei brucei*. Sequence, expression, and molecular analysis J Biol Chem 1998 273 2118 26 10.1074/jbc.273.4.2118 9442052

[B54] Di Virgilio F Chiozzi P Ferrari D Falzoni S Sanz JM Morelli A Torboli M Bolognesi G Baricordi OR Nucleotide receptors: an emerging family of regulatory molecules in blood cells Blood 2001 97 587 600 10.1182/blood.V97.3.587 11157473

[B55] Haskó G Cronstein Adenosine: an endogenous regulator of innate immunity Trends Immunol 2004 25 33 9 10.1016/j.it.2003.11.003 14698282

[B56] Ribeiro JM Valenzuela JG The salivary purine nucleosidase of the mosquito, *Aedes aegypti* Insect Biochem Mol Biol 2003 33 13 22 10.1016/S0965-1748(02)00078-4 12459196

[B57] Gounaris K Selkirk ME Parasite nucleotide-metabolizing enzymes and host purinergic signalling Trends Parasitol 2005 21 17 21 10.1016/j.pt.2004.10.005 15639736

[B58] Opperdoes FR Borst P Localization of nine glycolytic enzymes in a microbody-like organelle in *Trypanosoma brucei*: the glycosome FEBS Lett 1977 80 360 4 10.1016/0014-5793(77)80476-6 142663

[B59] Albert MA Haanstra JR Hannaert V Van Roy J Opperdoes FR Bakker BM Michels PA Experimental and in silico analyses of glycolytic flux control in bloodstream form *Trypanosoma brucei* J Biol Chem 2005 280 28306 15 10.1074/jbc.M502403200 15955817

[B60] Lay AJ Jiang XM Daly E Sun L Hogg PJ Plasmin reduction by phosphoglycerate kinase is a thiol-independent process J Biol Chem 2002 277 9062 9068 10.1074/jbc.M111531200 11782484

[B61] Veiga-Malta I Duarte M Dinis M Tavares D Videira A Ferreira P Enolase from *Streptococcus sobrinus *is an immunosuppressive protein Cell Microbiol 2004 6 79 88 10.1046/j.1462-5822.2003.00344.x 14678332

[B62] Huang LJ Chen SX Luo WJ Jiang HH Zhang PF Yi H Proteomic analysis of secreted proteins of non-small cell lung cancer Ai Zheng 2006 25 1361 7 17094902

[B63] Labbé M Péroval M Bourdieu C Girard-Misguich F Péry P *Eimeria tenella *enolase and pyruvate kinase: a likely role in glycolysis and in others functions Int J Parasitol 2006 36 1443 52 10.1016/j.ijpara.2006.08.011 17030033

[B64] Rokeach LA Zimmerman PA Unnasch TR Epitopes of the *Onchocerca volvulus *RAL1 antigen, a member of the calreticulin family of proteins, recognized by sera from patients with onchocerciasis Infect Immun 1994 62 3696 704 7520419 10.1128/iai.62.9.3696-3704.1994 PMC303020

[B65] Dupuis M Schaerer E Krause KH Tschopp J The calcium-binding protein calreticulin is a major constituent of lytic granules in cytolytic T lymphocytes J Exp Med 1993 177 1 7 10.1084/jem.177.1.1 8418194 PMC2190868

[B66] Mutayoba BM Meyer HH Osaso J Gombe S Trypanosome-induced increase in prostaglandin F(2alpha) and its relationship with corpus luteum function in the goat Theriogenology 1989 32 545 55 10.1016/0093-691X(89)90276-8 16726702

[B67] Hewitson JP Harcus YM Curwen RS Dowle AA Atmadja AK Ashton PD Wilson A Maizels RM The secretome of the filarial parasite, *Brugia malayi*: Proteomic profile of adult excretory-secretory products Mol Biochem Parasitol 2008 160 8 21 10.1016/j.molbiopara.2008.02.007 18439691

[B68] Cass CL Johnson JR Califf LL Xu T Hernandez HJ Stadecker MJ Yates JR Williams DL Proteomic analysis of *Schistosoma mansoni *egg secretions Mol Biochem Parasitol 2007 155 84 9 10.1016/j.molbiopara.2007.06.002 17644200 PMC2077830

[B69] Van Ooij C Tamez P Bhattacharjee S Hiller NL Harrison T Liolios K Kooij T Ramesar J Balu B Adams J Waters A Janse C Haldar K The malaria secretome: from algorithms to essential function in blood stage infection PLoS Pathog 2008 4 e1000084 10.1371/journal.ppat.1000084 18551176 PMC2408878

[B70] Reggiori F Pelham HR Sorting of proteins into multivesicular bodies: ubiquitin-dependent and -independent targeting EMBO J 2001 20 5176 86 10.1093/emboj/20.18.5176 11566881 PMC125630

[B71] Hiller NL Bhattacharjee S Van Ooij C Liolios K Harrison T Lopez-Estrano C Haldar K A host-targeting signal in virulence proteins reveals a secretome in malarial infection Science 2004 306 1934 1937 10.1126/science.1102737 15591203

[B72] Paindavoine P Pays E Laurent M Geltmeyer Y Le Ray D Mehlitz D Steinert M The use of DNA hybridization and numerical taxonomy in determining relationships between *Trypanosoma brucei *stocks and subspecies Parasitology 1986 92 31 50 10.1017/S0031182000063435 3960593

[B73] Tait A Babiker EA Le Ray D Enzyme variation in *Trypanosoma brucei *spp. I. Evidence for the sub-speciation of *Trypanosoma brucei gambiense* Parasitology 1984 89 311 26 10.1017/S0031182000001335 6504561

[B74] Mathieu-Daude F Bicart-See A Bosseno MF Breniere SF Tibayrenc M Identification of *Trypanosoma brucei gambiense *group I by a specific kinetoplast DNA probe Am J Trop Med Hyg 1994 50 13 9 8304568 10.4269/ajtmh.1994.50.13

[B75] Lanham SM Godfrey DG Isolation of salivarian trypanosomes from man and other mammals using DEAE-Cellulose Experimental Parasitol 1970 28 521 534 10.1016/0014-4894(70)90120-7 4993889

[B76] Holzmuller P Biron DG Courtois P Koffi M Bras-Goncalves R Daulouède S Solano P Cuny G Vincendeau P Jamonneau V Virulence and pathogenicity patterns of *Trypanosoma brucei gambiense *field isolates in experimentally infected mouse: differences in host immune response modulation by secretome and proteomics Microbes Infect 2008 10 79 86 10.1016/j.micinf.2007.10.008 18068387

[B77] Schägger H Von Jagow G Tricine-sodium dodecyl sulfate-polyacrylamide gel electrophoresis for the separation of proteins in the range from 1 to 100 kDa Anal Biochem 1987 166 368 379 10.1016/0003-2697(87)90587-2 2449095

[B78] Peltier J-B Ripoll DR Friso G Rudella A Cai Y Ytterberg J Giacomelli L Pillardy J Van Wijk KJ Clp protease complexes from photosynthetic and non-photosynthetic plastids and mitochondria of plants, their predicted three-dimensional structures, and functional implications J Biol Chem 2004 279 4768 4781 10.1074/jbc.M309212200 14593120

[B79] Elias JE Haas W Faherty BK Gygi SP Comparative evaluation of mass spectrometry platforms used in large-scale proteomics investigations Nature Methods 2005 2 667 675 10.1038/nmeth785 16118637

[B80] Uzest M Gargani D Drucker M Hébrard E Garzo E Candresse T Fereres A Blanc S A protein key to plant virus transmission at the tip of the insect vector stylet Proc Natl Acad Sci USA 2007 46 17959 17964 10.1073/pnas.0706608104 PMC2084279 17962414

[B81] Brun S Solignat M Gay B Bernard E Chaloin L Fenard D Devaux C Chazal N Briant L VSV-G pseudotyping rescues HIV-1 CA mutations that impair core assembly or stability Retrovirology 2008 5 57 1 15 18605989 10.1186/1742-4690-5-57 PMC2474847

