# Supplementary material for: Exocytosis and protein secretion in Trypanosoma
Source: BMC Microbiol. 2010 Jan 26;10:20. doi: 10.1186/1471-2180-10-20 (PMC3224696; doi:10.1186/1471-2180-10-20)
Supplement: Additional file 1 — Table S1. Secreted proteins identified in 3 T. brucei gambiense strains separated on 1D gel. contains the identification of the proteins secreted by Biyamina (sheet 1), Feo (sheet 2), and OK strain (sheet 3) and their classification according to functional categories (MapMan bins nomenclature). For each protein, the number of matched peptides and the highest score are described. [file 1471-2180-10-20-S1.PDF]

Table S1-All 1D

SHEET 1 **Biyamina 1D secretome**

Accession number	Protein name	MapMan bins	Matching peptides	Highest peptide score	Molecular Weight
Tb09.211.3550	glycerol kinase, glycosomal	11.5 lipid metabolism.glycerol metabolism	6	67	56365.88
Tb927.3.4680	RAB GDP dissociation inhibitor alpha, putative	31.4 cell. vesicle transport	1	51	49396.71
Tb09.160.3710	proliferative cell nuclear antigen (PCNA), putative	31.3 cell.cycle	2	57	32314.61
Tb09.211.4511	kinetoplastid membrane protein KMP-11	31.1 cell.organisation	1	31	11076.49
Tb10.61.1750	C-terminal motor kinesin, putative	31.1 cell.organisation	1	82	91485
Tb927.1.2330	beta tubulin	31.1 cell.organisation	3	53	49703.96
Tb927.1.2340	alpha tubulin	31.1 cell.organisation	1	44	49787.13
Tb10.389.1730	mitogen-activated protein kinase 11, putative	31.3 cell.cycle ou 31.2 cell.division	1	34	47389.49
Tb927.8.4970	69 kDa paraflagellar rod protein,PFR2	31.6* cell organization-cytoskeleton	1	63	69597.02
Tb11.47.0001	65 kDa invariant surface glycoprotein-like protein	31.99 cell.unspecified	1	30	47801.74
Tb10.61.2680	pyruvate kinase 1	4 glycolysis	3	58	38453.32
Tb10.6K15.3850	GAP glyceraldehyde 3-phosphate dehydrogenase, cytosolic	4 glycolysis	2	82	35610.62
Tb10.70.1370	fructose-bisphosphate aldolase, glycosomal	4 glycolysis	1	38	41071.12
Tb927.1.700	phosphoglycerate kinase	4 glycolysis	4	62	47245.76
Tb10.70.4740	enolase	4.12 glycolysis.enolase	9	111	46592.13
Tb11.02.3210	triosephosphate isomerase	4.8 glycolysis.TPI	2	45	26973
Tb927.8.5600	transaldolase, putative	7.2.2 OPP.non-reductive PP.transaldolase	2	59	36626.86
Tb11.01.3040	cytosolic malate dehydrogenase, putative	8.1 TCA / org. transformation.TCA	2	58	35151.21
Tb11.01.8470	dihydrolipoyl dehydrogenase	8.1.1.3 TCA / org. transformation.	2	29	50432.99
Tb09.V1.0380	spermidine synthase, putative	22.1.6 polyamine metabolism.synthesis	3	63	32926.41
Tb09.160.4570	arginine kinase	13.1.1 amino acid metabolism	3	66	41597.10
Tb09.160.4590	arginine kinase	13.1.1 amino acid metabolism	4	56	40196.59
Tb10.70.3710	aspartate aminotransferase	13.1.1.2 amino acid metabolism	2	75	44787.43
Tb927.1.3950	alanine aminotransferase, putative	13.1.1.3 amino acid metabolism	2	96	63137.20

Tb927.2.4590	branched-chain amino acid aminotransferase, putative	13.1.1.3 amino acid metabolism	2	43	40432.42
Tb10.406.0520	trypanothione reductase	21.2 redox.ascorbate and glutathione	3	61	53156.79
Tb927.3.3760	tryparedoxin	21.5 redox.peroxiredoxins	2	88	15921.00
Tb09.211.4460	ADP-ribosylation factor, putative	23 nucleotide metabolism	1	26	20753
Tb11.01.7800	nucleoside diphosphate kinase	23 nucleotide metabolism	4	80	16904
Tb927.3.2960	inosine-adenosine-guanosine-nucleosidehydrolase	23 nucleotide metabolism	2	33	36509
Tb927.6.2300	adenosine kinase, putative	23 nucleotide metabolism	1	28	37908.21
Tb927.7.4570	nucleoside hydrolase, putative	23 nucleotide metabolism	1	36	39390.73
Tb927.5.3820	aspartate carbamoyltransferase, putative	23.1.1 nucleotide metabolism	1	58	35910.64
Tb10.26.0200	guanylate kinase, putative	23.1.2 nucleotide metabolism	1	28	20264
Tb11.02.1120	adenylosuccinate synthetase, putative	23.1.2 nucleotide metabolism	3	53	66674.89
Tb927.5.3830	dihydroorotate dehydrogenase, putative	23.2 nucleotide metabolism	1	51	34112.29
Tb09.211.0560	RNA-binding protein, putative,DRBD3	27.4 RNA.RNA binding	2	58	36984.69
Tb10.70.5360	La protein,RNA-binding protein, putative	27.4 RNA.RNA binding	1	56	37659.61
Tb927.8.740	nucleolar RNA-binding protein, truncated	27.4 RNA.RNA binding	1	38	13803.88
Tb11.01.4621	calmodulin	30.3 signalling.calcium	5	77	16828
Tb927.8.5440	flagellar calcium-binding protein,24 kDa calflagin	30.3 signalling.calcium	1	66	24580
Tb11.01.3170	guanine nucleotide-binding protein beta subunit-like protein,	30.5 signalling.G-proteins	1	59	35181
Tb11.01.1290	14-3-3-like protein, putative	30.7 signalling.14-3-3 proteins	8	91	30310.38
Tb11.02.4700	14-3-3-like protein, putative	30.7 signalling.14-3-3 proteins	6	84	29196.24
Tb11.02.2310	prostaglandin f synthase	30.99 signalling.unspecified	3	41	30992.51
Tb11.01.1350	S-adenosylhomocysteine hydrolase, putative	13.1.3.4 amino acid metabolism	2	38	48447.50
Tb11.01.1680	polyubiquitin, putative	29.4 protein.postranslational modification	3	83	76603.14
Tb11.02.5020	seryl-tRNA synthetase, putative	29.1 protein.aa activation	1	61	53857.17
Tb09.160.3270	eukaryotic initiation factor 4a, putative	29.2.4 protein.synthesis.elongation	2	73	45361.53
Tb09.244.2730	60S ribosomal protein L5, putative	29.2.4 protein.synthesis.elongation	1	20	34635.92
Tb10.6K15.2050	RPS12 40S ribosomal protein S12, putative	29.2.4 protein.synthesis.elongation	1	61	16061.40
Tb10.70.2650	elongation factor 2	29.2.4 protein.synthesis.elongation	6	57	94334.21
Tb10.26.1080	heat shock protein 83,heat shock protein	29.6 protein.(un)folding	1	93	80763.23
Tb10.389.0880	heat shock protein, putative	29.6 protein.(un)folding	9	65	90863.73

Tb10.61.0180	peptidylprolyl isomerase-like protein, putative	29.6 protein.(un)folding	1	47	47604.30
Tb10.6K15.2290	BS2 protein disulfide isomerase	29.6 protein.(un)folding	5	48	55887
Tb10.6K15.2520	prolyl oligopeptidase, putative; serine peptidase , family S9A	29.6 protein.(un)folding	1	61	78033
Tb10.70.0850	proteasome alpha 1 subunit, putative,20S proteasome subunit	29.6 protein.(un)folding	1	55	29449
Tb10.70.5250	metacaspase MCA4,cysteine peptidase, Clan CD, family C13	29.6 protein.(un)folding	1	43	38969
Tb11.01.3110	heat shock protein 70	29.6 protein.(un)folding	18	101	75366.42
Tb11.02.0100	carboxypeptidase, putative,metallo-peptidase, Family M32	29.6 protein.(un)folding	1	54	57683.01
Tb11.02.1070	aminopeptidase, putative,metallo-peptidase, Family M1	29.6 protein.(un)folding	1	47	98099.41
Tb11.02.4440	aminopeptidase, putative,metallo-peptidase, Family M17	29.6 protein.(un)folding	2	67	55359.24
Tb11.02.5450	glucose-regulated protein 78, putative,	29.6 protein.(un)folding	4	86	71505
Tb11.03.0250	cyclophilin a,cyclophilin type peptidyl-prolyl cis-trans isomerase	29.6 protein.(un)folding	3	35	18933
Tb927.6.1260	proteasome beta-1 subunit, putative	29.6 protein.(un)folding	1	50	30878
Tb927.6.400	peptidase M20/M25/M40, putative	29.6 protein.(un)folding	2	30	52185.07
Tb927.7.4060	calpain-like cysteine peptidase, putative, family C2, putative	29.6 protein.(un)folding	1	49	12979
Tb927.7.4420	proteasome alpha 3 subunit, putative	29.6 protein.(un)folding	1	61	32154.10
Tb927.7.4770	cyclophilin-type peptidyl-prolyl cis-trans isomerase, putative	29.6 protein.(un)folding	2	40	18635
Tb927.7.4790	proteasome beta 6 subunit,20S proteasome beta 6 subunit	29.6 protein.(un)folding	1	27	28958
Tb927.7.5790	protein disulfide isomerase, putative	29.6 protein.(un)folding	2	47	15400.76
Tb09.160.5530	hypothetical protein, conserved	35.1 not assigned.no ontology	3	99	38016.33
Tb09.211.0610	hypothetical protein, conserved	35.1 not assigned.no ontology	1	23	119546.78
Tb10.389.0720	hypothetical protein, conserved	35.1 not assigned.no ontology	6	71	110243.85
Tb10.61.0540	hypothetical protein, conserved	35.1 not assigned.no ontology	1	57	36411.55
Tb10.70.1130	hypothetical protein, conserved	35.1 not assigned.no ontology	1	69	48257.68
Tb11.01.5680	hypothetical protein, conserved	35.1 not assigned.no ontology	1	58	51983
Tb11.46.0009	hypothetical protein, conserved,zinc finger protein family member	35.1 not assigned.no ontology	1	46	58459.91
Tb11.55.0024	hypothetical protein, conserved	35.1 not assigned.no ontology	1	41	37610.93
Tb927.1.1100	hypothetical protein, conserved	35.1 not assigned.no ontology	1	25	108810.61
Tb927.1.180	retrotransposon hot spot (RHS) protein, putative	35.1 not assigned.no ontology	1	37	94829
Tb927.3.3560	hypothetical protein, conserved	35.1 not assigned.no ontology	4	80	69087.74
Tb927.4.1300	hypothetical protein, conserved	35.1 not assigned.no ontology	1	44	42014.86

Tb927.4.2030	hypothetical protein, conserved	35.1 not assigned.no ontology	1	56	22691
Tb927.7.3440	I/6 autoantigen	35.2 not assigned.unknown	1	56	27050.07

Table S1-All 1D

SHEET 2 **FEO 1D secretome**

Accession number	Protein name	MapMan bins	Matching peptides	Highest peptide score	Molecular Weight
Tb09.211.3550	glycerol kinase glycosomal	11.5 lipid metabolism.glycerol metabolism	11	72	56365.88
Tb09.211.3540	glycerol kinase glycosomal	11.5 lipid metabolism.glycerol metabolism	2	46	56335.85
Tb927.8.3530	glycerol-3-phosphate dehydrogenase [NAD+], glycosomal	11.3 lipid metabolism.Phospholipid synthesis	7	85	37805.01
Tb927.8.6390	lysophospholipase, putative	11.9.3 lipid metabolism	5	64	30092
Tb09.160.3710	proliferative cell nuclear antigen (PCNA), putative	31.3 cell.cycle	1	88	32314.61
Tb09.211.4511	kinetoplastid membrane protein KMP-11	31.1 cell.organisation	1	59	11076.49
Tb10.406.0560	microtubule-associated protein	31.1 cell.organisation	3	56	237491.49
Tb927.1.2340	alpha tubulin	31.1 cell.organisation	9	77	49787.13
Tb927.1.2330	beta tubulin	31.1 cell.organisation	6	77	49703.96
Tb927.8.4970	69 kDa paraflagellar rod protein	31.6* cell organization	6	64	69597.02
Tb927.3.4290	73 kDa paraflagellar rod protein	31.6* cell organization	6	84	68682.76
Tb10.6k15.1160	G-actin binding protein	31.6* cell organization	1	40	29333.07
Tb09.160.0260	variant surface glycoprotein	31.99 cell.unspecified	1	36	57871.54
Tb927.8.890	small GTP-binding protein Rab1, putative	31.4 cell. vesicle transport	1	39	22714.65
Tb10.70.0830	clathrin heavy chain	31.4 cell. vesicle transport	8	62	190625.63
Tb10.70.1190	Valosin VCP-like	29.3 protein.targeting	4	38	86570
Tb09.v1.0380	spermidine synthase, putative	22.1.6 polyamine metabolism	1	50	32926.41
Tb927.2.4590	aa aminotransferase	13 amino acid metabolism	4	71	40432.42
Tb927.7.1110	asparagine synthetase a, putative	13.1.1 amino acid metabolism	1	43	39597.28
Tb09.160.4590	arginine kinase	13.1.1 amino acid metabolism	2	89	40196.59
Tb09.160.4570	arginine kinase	13.1.1 amino acid metabolism	3	47	41597.10
Tb10.70.3710	aspartate aminotransferase	13.1.1.2 amino acid metabolism	6	90	44787.43
Tb927.1.3950	Alanine aminotransferase	13.1.1.3 amino acid metabolism	4	63	63137.20
Tb927.8.6750	translationally controlled tumor protein (TCTP), putative	31.3 cell.cycle	2	60	19367
Tb10.61.2680	pyruvate kinase	4 glycolysis	5	49	38453.32

Tb10.70.5800	hexokinase	4 glycolysis	6	58	51148.82
Tb927.1.700	phosphoglycerate kinase	4 glycolysis	4	74	47245.76
Tb10.70.1370	fructose-bisphosphate aldolase glycosomal	4 glycolysis	7	64	41071.12
Tb927.6.4280	GAPDH glycosomal	4 glycolysis	6	61	43869.18
Tb10.6k15.3850	GAP glyceraldehyde 3-phosphate dehydrogenase, cytosolic	4 glycolysis	6	84	35610.62
Tb11.02.3210	Triose P isomerase	4 glycolysis	4	65	26818.79
Tb10.6k15.2620	P-glycerate kinase	4 glycolysis	1	51	60603.34
Tb10.70.4740	Enolase	4.12 glycolysis.enolase	13	104	46592.13
Tb11.01.3040	cytosolic malate dehydrogenase, putative	8.1 TCA / org. transformation.TCA	6	58	35151.21
Tb927.6.1570	2-hydroxy-3-oxopropionate reductase, putative	6 gluconeogenese/ glyoxylate cycle	1	50	31622
Tb10.70.7730	ATP-dependent DEAD/H RNA helicase	2.1.1 major CHO metabolism	1	36	49651
Tb10.406.0520	trypanothione reductase	21.2 redox.ascorbate and glutathione	3	32	53156.79
Tb09.160.4250	tryparedoxin peroxidase	21.5 redox.peroxiredoxins	3	56	22424.65
Tb927.8.1990	tryparedoxin peroxidase	21.5 redox.peroxiredoxins	2	62	25631.46
Tb927.3.3760	tryparedoxin	21.5 redox.peroxiredoxins	3	59	15921.00
Tb11.01.7550	iron superoxide dismutase	21.6 redox.dismutases and catalases	2	79	22047.76
Tb11.01.1350	SAM hydrolase	23 nucleotide metabolism	5	61	48447.50
Tb927.6.4840	S-adenosylmethionine synthetase, putative	23 nucleotide metabolism	6	69	43540.13
Tb927.6.2360	Adenosine kinase	23 nucleotide metabolism	5	55	37958.29
Tb927.7.4570	nucleoside hydrolase, putative	23 nucleotide metabolism	1	55	39390.73
Tb927.8.4430	Nucleoside Pylase	23 nucleotide metabolism	6	94	36924.95
Tb927.3.2960	IAG nucleoside hydrolase	23 nucleotide metabolism	4	48	36509
Tb09.211.4460	ADP-ribosylation factor, putative	23 nucleotide metabolism	2	48	20651.84
Tb927.3.3450	ADP-ribosylation factor-like protein 3A, putative	23 nucleotide metabolism	4	74	19880.70
Tb11.01.7800	Nucleoside diP kinase	23 nucleotide metabolism	6	105	16857.24
Tb11.02.1120	adenylosuccinate synthetase	23.1.2 nucleotide metabolism	1	47	66674.89
Tb10.70.6660	hypoxanthine-guanine phosphoribosyltransferase	23.1.2 nucleotide metabolism	1	62	26360.31
Tb09.211.0350	adenylate kinase	23.1.2 nucleotide metabolism	2	48	22440.56
Tb10.26.0200	guanylate kinase	23.1.2 nucleotide metabolism	5	94	20106.00
Tb927.7.7040	Methylthioadenosine Pylase	28.1 DNA.synthesis/chromatin structure	5	43	33443.37
Tb11.46.0008	arginyl-tRNA synthetase	29.1 protein.aa activation	2	45	78432.50

Tb11.03.0410	Translation initiation factor	29.2 protein.synthesis	2	82	17820.01
Tb11.01.2560	40S ribosomal protein SA	29.2.4 protein.synthesis.elongation	1	48	27609.60
Tb927.4.3570	translation elongation factor 1-beta, putative	29.2.4 protein.synthesis.elongation	1	47	28403.66
Tb10.6k15.2050	40S ribosomal protein S12	29.2.4 protein.synthesis.elongation	2	68	16061.40
Tb10.70.2650	Elongation factor	29.2.4 protein.synthesis.elongation	14	88	94334.21
Tb10.70.5650	elongation factor 1-alpha	29.2.4 protein.synthesis.elongation	1	60	49105.63
Tb11.01.4660	elongation factor 1 gamma	29.2.4 protein.synthesis.elongation	2	48	46303.53
Tb11.01.1680	Ubiquitin	29.4 protein.postranslational modification	3	54	76603.14
Tb11.52.0003	oligopeptidase b	29.5 protein.degradation	2	58	81320
Tb11.02.4440	aminopeptidase	29.5 protein.degradation	3	55	55359.24
Tb11.02.0815	ubiquitin-conjugating enzyme	29.5.11 protein.degradation.ubiquitin	2	58	15815
Tb11.02.4870	proteasome subunit alpha 7	29.5.11.20 protein.degradation.ubiquitin	1	44	27865.39
Tb927.6.1260	proteasome beta-1 subunit, putative	29.5.11.20 protein.degradation.ubiquitin	1	54	30441.39
Tb927.7.4790	proteasome beta 6 subunit	29.5.11.20 protein.degradation.ubiquitin	2	55	28958
Tb10.70.3660	proteasome activator protein PA26	29.5.11.20 protein.degradation.ubiquitin	1	37	25257.72
Tb927.7.4060	calpain-like cysteine peptidase, putative	29.5.3 protein.degradation.cysteine protease	2	46	12979
Tb10.26.1080	HSP83	29.6 protein.(un)folding	4	47	80763.23
Tb09.211.1350	peptidyl-prolyl cis-trans isomerase (cyclophilin- 40)	29.6 protein.(un)folding	4	47	38584
Tb927.7.4770	cyclophilin-type peptidyl-prolyl cis-trans isomerase, putative	29.6 protein.(un)folding	2	45	18589.
Tb10.389.0880	HSP	29.6 protein.(un)folding	14	109	90863.73
Tb11.03.0250	cyclophilin A	29.6 protein.(un)folding	2	39	18717
Tb10.6K15.2290	protein disulfide isomerase	29.6 protein.(un)folding	12	67	55887
Tb11.01.3110	HSP70	29.6 protein.(un)folding	21	90	75366.42
Tb10.61.0180	peptidylprolyl isomerase-like protein	29.6 protein.(un)folding	3	36	47604.30
Tb927.7.5790	protein disulfide isomerase, putative	29.6 protein.(un)folding	3	38	15400.76
Tb927.8.5440	flagellar calcium-binding protein TB-24	30.3 signalling.calcium	1	30	24253.22
Tb11.01.3170	guanine nucleotide-binding protein subunit beta-like protein	30.5 signalling.G-proteins	2	59	35181
Tb11.01.1290	14-3-3 protein I	30.7 signalling.14-3-3 proteins	6	82	30310.38
Tb11.02.4700	14-3-3 protein II	30.7 signalling.14-3-3 proteins	6	79	29196.24
Tb11.02.2310	Chain B, The Crystal Structure Of Prostaglandin F Synthase	30.99 signalling.unspecified	4	53	30992.51
Tb10.389.0720	hypoth prot	35.1 not assigned.no ontology	2	52	110243.85

Tb927.4.860	hypoth prot	35.1 not assigned.no ontology	1	50	33014
Tb927.4.3700	hypothetical protein, conserved	35.1 not assigned.no ontology	1	50	92552
Tb927.7.4520	hypoth prot	35.1 not assigned.no ontology	3	103	22315.20
Tb10.70.5630	hypoth prot	35.1 not assigned.no ontology	1	32	100062.57
Tb09.160.1160	hypothetical protein Tb09.160.1160	35.1 not assigned.no ontology	2	46	85913.86
Tb10.6k15.2630	hypothetical protein Tb10.6k15.2630	35.1 not assigned.no ontology	1	50	25849
Tb10.61.3210	hypothetical protein Tb10.61.3210	35.1 not assigned.no ontology	1	43	37612.28
Tb927.4.1300	hypothetical protein, conserved	35.1 not assigned.no ontology	1	57	42014.86
Tb927.7.4290	hypothetical protein, conserved	35.1 not assigned.no ontology	1	30	67283.77
Tb11.01.5680	hypothetical protein Tb11.01.5680	35.1 not assigned.no ontology	1	58	51674.18
Tb10.70.6610	hypothetical protein Tb10.70.6610	35.1 not assigned.no ontology	2	39	27338.99
Tb927.8.2260	hypothetical protein, conserved	35.1 not assigned.no ontology	2	36	21768.62
Tb927.2.4580	hypothetical protein, conserved	35.1 not assigned.no ontology	1	42	22660.49
Tb927.4.2740	hypothetical protein, conserved	35.1 not assigned.no ontology	3	55	16327.52
Tb10.26.0680	hypothetical protein Tb10.26.0680	35.1 not assigned.no ontology	1	50	14328.23
Tb09.160.5530	hypothetical protein Tb09.160.5530	35.1 not assigned.no ontology	3	61	38016.33
Tb11.55.0024	hypoth prot ARD motif	35.1 not assigned.no ontology	2	47	37610.93
Tb09.211.1690	hypothetical protein, conserved	35.1 not assigned.no ontology	2	56	18768.80
Tb10.70.1130	hypothetical protein Tb10.70.1130	35.1 not assigned.no ontology	5	50	48257.68
Tb10.6k15.3580	hypoth prot	35.1 not assigned.no ontology	1	42	37269.32
Tb927.4.2030	hypothetical protein, conserved	35.1 not assigned.no ontology	3	35	22703.92
Tb927.8.6760	IgE-dpt histidine release factor	35.2 not assigned.unknown	1	50	19219
Tb11.01.0120	haloacid dehalogenase-like hydrolase	35.2 not assigned.unknown	2	56	31584.91
Tb10.70.2770	stress-inducible protein STI1-like	35.2 not assigned.unknown	4	69	28424.92
Tb927.7.3440	I/6 autoantigen	35.2 not assigned.unknown	1	62	27050.07

Table S1-All 1D

SHEET 3 **OK 1D secretome**

Accession number	Protein name	MapMan bins	Matching peptides	Highest peptide score	Molecular Weight
Tb10.406.0520	trypanothione reductase	21.2 redox.ascorbate and glutathione	1	40	53156.79
Tb927.7.1120	trypanothione/tryparedoxin dependent peroxidase 1, cytosolic	21.5 redox.peroxiredoxins	1	38	18747
Tb927.3.3760	tryparedoxin	21.5 redox.peroxiredoxins	1	41	15921.00
Tb09.160.4250	tryparedoxin peroxidase	21.5 redox.peroxiredoxins	3	46	22424.65
Tb927.8.1990	tryparedoxin peroxidase	21.5 redox.peroxiredoxins	3	66	25631.46
Tb11.01.7550	iron superoxide dismutase	21.6 redox.dismutases and catalases	3	73	22047.76
Tb09.211.3540	glycerol kinase, glycosomal	11.5 lipid metabolism	3	52	56335.85
Tb927.8.3530	glycerol-3-phosphate dehydrogenase [NAD+], glycosomal	11.3 lipid metabolism.	5	79	37805.01
Tb927.6.2740	pyridoxal kinase	18 Co-factor and vitamine metabolism	1	52	33332.03
Tb11.01.3170	guanine nucleotide-binding protein beta subunit-like protein	30.5 signalling.G-proteins	3	55	35181
Tb11.01.1290	14-3-3-like protein, putative	30.7 signalling.14-3-3 proteins	7	90	30310.38
Tb11.02.4700	14-3-3-like protein, putative	30.7 signalling.14-3-3 proteins	4	54	29196.24
Tb11.02.2310	prostaglandin f synthase	30.99 signalling.unspecified	3	45	30992.51
Tb927.1.3200	phosphatase-like protein, putative	29.4 protein.postranslational modification	1	69	24493.00
Tb09.211.2410	protein kinase A catalytic subunit isoform 1	29.4 protein.postranslational modification	1	47	38187
Tb927.4.2110	protein phosphatase 2C, putative	29.4 protein.postranslational modification	2	46	33456.31
Tb927.6.1800	protein phosphatase 2C, putative	29.4 protein.postranslational modification	1	53	41135.15
Tb11.01.8740	protein phosphatase 2A, putative	29.4 protein.postranslational modification	3	57	35094
Tb10.05.0110	serine/threonine protein phosphatase 5	29.4 protein.postranslational modification	1	46	53312.62
Tb09.V1.0380	spermidine synthase, putative	22.1.6 polyamine metabolism	2	71	32926.41
Tb09.160.4570	arginine kinase	13.1.1 amino acid metabolism	5	78	41597.10
Tb09.160.4590	arginine kinase	13.1.1 amino acid metabolism	3	50	40196.59
Tb10.70.3710	aspartate aminotransferase	13.1.1.2 amino acid metabolism	2	51	44787.43
Tb927.1.3950	alanine aminotransferase, putative	13.1.1.3 amino acid metabolism	4	79	63137.20

Tb927.2.4590	branched-chain amino acid aminotransferase, putative	13.1.1.3 amino acid metabolism	6	54	40432.42
Tb927.6.1080	hydroxyacylglutathione hydrolase, putative,glyoxalase II	13.2.3.2 amino acid metabolism	1	71	32830
Tb10.70.5800	hexokinase	2.2.1.4 major CHO metabolism	3	44	51148.82
Tb10.70.1370	fructose-bisphosphate aldolase, glycosomal	4 glycolysis	2	64	41071.12
Tb10.6K15.3850	GAP glyceraldehyde 3-phosphate dehydrogenase, cytosolic	4 glycolysis	3	90	35610.62
Tb927.6.4280	glyceraldehyde 3-phosphate dehydrogenase, glycosomal	4 glycolysis	5	61	43869.18
Tb927.1.700	phosphoglycerate kinase	4 glycolysis	3	76	47245.76
Tb10.61.2680	pyruvate kinase 1	4 glycolysis	3	67	38453.32
Tb10.6K15.2620	bisphosphoglycerate-independent phosphoglycerate mutase	4 glycolysis	7	82	60603.34
Tb10.70.4740	enolase	4.12 glycolysis.enolase	11	104	46592.13
Tb11.02.3210	triosephosphate isomerase	4.8 glycolysis.TPI	3	56	26818.79
Tb927.8.5600	transaldolase, putative	7.2.2 OPP.non-reductive PP.transaldolase	1	36	36626.86
Tb11.01.3040	cytosolic malate dehydrogenase, putative	8.1 TCA / org. transformation.TCA	4	52	35151.21
Tb09.160.3630	cAMP-specific phosphodiesterase	23 nucleotide metabolism	2	59	103217.86
Tb927.7.1780	adenine phosphoribosyltransferase, putative	23 nucleotide metabolism	1	55	25972.97
Tb927.6.2300	adenosine kinase, putative	23 nucleotide metabolism	1	44	37908.21
Tb927.6.2360	adenosine kinase, putative	23 nucleotide metabolism	3	53	37958.29
Tb09.211.4460	ADP-ribosylation factor, putative	23 nucleotide metabolism	4	64	20651.84
Tb927.6.3650	ADP-ribosylation factor, putative	23 nucleotide metabolism	1	43	20071.68
Tb927.3.3450	ADP-ribosylation factor-like protein 3A, putative	23 nucleotide metabolism	2	83	19880.70
Tb10.70.6540	hypoxanthine-guanine phosphoribosyltransferase	23 nucleotide metabolism	2	54	23371.90
Tb10.70.6660	hypoxanthine-guanine phosphoribosyltransferase, putative	23 nucleotide metabolism	1	64	26360.31
Tb927.3.2960	inosine-adenosine-guanosine-nucleoside hydrolase	23 nucleotide metabolism	3	46	36509
Tb927.8.2050	mannose-1-phosphate guanyltransferase, putative	**23** nucleotide metabolism	1	66	40704.08
Tb11.01.7800	nucleoside diphosphate kinase	23 nucleotide metabolism	2	47	16857.24
Tb927.7.4570	nucleoside hydrolase, putative	23 nucleotide metabolism	1	54	39390.73
Tb927.8.4430	nucleoside phosphorylase, putative	23 nucleotide metabolism	3	77	36924.95
Tb11.01.1350	S-adenosylhomocysteine hydrolase, putative	23 nucleotide metabolism	4	49	48447.50
Tb927.6.4840	S-adenosylmethionine synthetase, putative	23 nucleotide metabolism	1	55	43540.13
Tb11.V4.0004	RNR2 ribonucleoside-diphosphate reductase small chain	23 nucleotide metabolism	1	59	39017.78
Tb927.5.3820	aspartate carbamoyltransferase, putative	23.1.1 nucleotide metabolism	1	62	35910.64

Tb10.26.0200	guanylate kinase, putative	23.1.2 nucleotide metabolism	1	72	20106.00
Tb11.02.1120	adenylosuccinate synthetase, putative	23.1.2 nucleotide metabolism	8	60	66674.89
Tb927.5.3830	dihydroorotate dehydrogenase, putative	23.2 nucleotide metabolism	1	72	34112.29
Tb10.70.3070	TFIIF-stimulated CTD phosphatase, putative	27.3 RNA.regulation of transcription	1	50	28189.10
Tb10.70.7730	ATP-dependent DEAD/H RNA helicase, putative,DEAD box RNA he	28.1 DNA.synthesis/chromatin structure	2	43	49651
Tb927.3.3490	high mobility group protein, putative	28.1 DNA.synthesis/chromatin structure	1	82	30866.36
Tb927.7.7040	methylthioadenosine phosphorylase, putative	28.1 DNA.synthesis/chromatin structure	2	30	33443.37
Tb11.46.0008	arginyl-tRNA synthetase, putative	29.1 protein.aa activation	5	45	78432.50
Tb11.02.1210	leucyl-tRNA synthetase, putative	29.1 protein.aa activation	4	50	121824.99
Tb10.70.6470	methionyl-tRNA synthetase, putative	29.1 protein.aa activation	2	35	86910.83
Tb11.02.5020	seryl-tRNA synthetase, putative	29.1 protein.aa activation	1	39	53857.17
Tb11.01.4660	elongation factor 1 gamma, putative	29.2.4 protein.synthesis.elongation	2	49	46303.53
Tb10.70.5650	elongation factor 1-alpha	29.2.4 protein.synthesis.elongation	4	61	49105.63
Tb10.70.2650	elongation factor 2	29.2.4 protein.synthesis.elongation	11	85	94334.21
Tb10.70.1670	40S ribosomal protein S10, putative	29.2.4 protein.synthesis.elongation	1	35	19285.66
Tb10.70.3360	40S ribosomal protein S3a, putative	29.2.4 protein.synthesis.elongation	1	63	29422.06
Tb11.02.1085	40s ribosomal protein S4, putative	29.2.4 protein.synthesis.elongation	1	33	30643.93
Tb11.01.2560	40S ribosomal protein SA, putative	29.2.4 protein.synthesis.elongation	1	48	27609.60
Tb09.211.4550	60S ribosomal protein L12, putative	29.2.4 protein.synthesis.elongation	1	29	24017.29
Tb09.160.3270	eukaryotic initiation factor 4a, putative	29.2.4 protein.synthesis.elongation	3	68	45361.53
Tb11.03.0410	eukaryotic translation initiation factor 5a, putative	29.2.4 protein.synthesis.elongation	2	58	17820.01
Tb927.4.3570	translation elongation factor 1-beta, putative	29.2.4 protein.synthesis.elongation	1	49	28403.66
Tb10.6K15.2290	BS2 protein disulfide isomerase	29.6 protein.(un)folding	6	70	55887
Tb927.1.2100	calpain-like cysteine peptidase, putative, family C2	29.6 protein.(un)folding	3	73	127046
Tb11.02.1070	aminopeptidase, putative,metallo-peptidase, Clan MA,Family M1	29.6 protein.(un)folding	2	58	98099.41
Tb11.02.4440	aminopeptidase, putative,metallo-peptidase, Clan MF, Family M17	29.6 protein.(un)folding	1	41	55359.24
Tb927.2.5160	chaperone protein DnaJ, putative	29.6 protein.(un)folding	1	33	44813.55
Tb11.03.0250	cyclophilin a,cyclophilin type peptidyl-prolyl cis-trans isomerase	29.6 protein.(un)folding	6	65	18717
Tb927.7.4770	cyclophilin-type peptidyl-prolyl cis-trans isomerase, putative	29.6 protein.(un)folding	1	43	18589.
Tb11.02.5450	glucose-regulated protein 78, putative	29.6 protein.(un)folding	3	88	71505
Tb927.7.710	heat shock 70 kDa protein, putative	29.6 protein.(un)folding	1	40	70211.63

Tb11.01.3110	heat shock protein 70	29.6 protein.(un)folding	17	106	75366.42
Tb11.01.3080	heat shock protein 70, putative	29.6 protein.(un)folding	1	49	73630.61
Tb10.26.1080	heat shock protein 83,heat shock protein	29.6 protein.(un)folding	11	106	80763.23
Tb10.389.0880	heat shock protein, putative	29.6 protein.(un)folding	9	84	90863.73
Tb10.70.5250	metacaspase MCA4,cysteine peptidase, Clan CD, family C13, puta	29.6 protein.(un)folding	1	34	38969
Tb11.02.0730	metacaspase,cysteine peptidase, Clan CD, family C13	29.6 protein.(un)folding	1	44	40180.06
Tb11.52.0003	oligopeptidase b,serine peptidase, clan SC, family S9A-like protein	29.6 protein.(un)folding	1	68	81320
Tb10.61.0180	peptidylprolyl isomerase-like protein, putative	29.6 protein.(un)folding	1	31	47604.30
Tb10.6K15.2520	prolyl oligopeptidase, ; serine peptidase clan SC, family S9A	29.6 protein.(un)folding	3	71	78033
Tb10.70.0850	proteasome alpha 1 subunit, putative,20S proteasome subunit alph	29.6 protein.(un)folding	2	61	29449
Tb10.100.0170	proteasome alpha 2 subunit, putative	29.6 protein.(un)folding	1	79	25354.60
Tb927.7.4420	proteasome alpha 3 subunit, putative	29.6 protein.(un)folding	3	60	32154.10
Tb10.100.0120	proteasome alpha 5 subunit, putative,20S proteasome subunit alph	29.6 protein.(un)folding	1	72	27176.51
Tb11.02.4870	proteasome alpha 7 subunit, putative	29.6 protein.(un)folding	1	42	27865.39
Tb10.70.0790	proteasome beta 5 subunit, putative,proteasome beta 5 subunit	29.6 protein.(un)folding	1	61	34415.80
Tb11.01.8510	t-complex protein 1, alpha subunit, putative	29.6 protein.(un)folding	1	41	54623.76
Tb927.7.190	thimet oligopeptidase A, putative,metallo-peptidase, Clan MA(E) Fa	29.6 protein.(un)folding	1	37	77937
Tb09.211.3610	ubiquitin-activating enzyme E1, putative	29.6 protein.(un)folding	1	45	134654.90
Tb11.02.0815	ubiquitin-conjugating enzyme, putative,ubiquitin-conjugating enzym	29.6 protein.(un)folding	1	56	15815
Tb10.70.0830	clathrin heavy chain	31.4 cell. vesicle transport	10	82	190625.63
Tb927.3.4720	dynamin, putative,vacuolar sortin protein 1, putative	31.4 cell. vesicle transport	1	43	73321.94
Tb10.70.1190	valosin-containing protein homolog	29.3 protein.targeting	1	63	86570
Tb10.6K15.3970	developmentally regulated GTP-binding protein, putative	31.4 cell. vesicle transport	1	42	41267.84
Tb927.1.2340	alpha tubulin	31.1 cell.organisation	4	81	49787.13
Tb927.1.2330	beta tubulin	31.1 cell.organisation	8	81	49703.96
Tb09.211.4511	kinetoplastid membrane protein KMP-11	31.1 cell.organisation	1	45	11076.49
Tb927.8.6750	translationally controlled tumor protein (TCTP)	31.3 cell.cycle	1	61	19367
Tb10.70.7040	cell division protein kinase 2 homolog 1,cdc2-like protein kinase	31.3 cell.cycle ou 31.2 cell.division	1	47	34671
Tb10.389.1730	protein kinase, putative,mitogen-activated protein kinase 11, putativ	31.3 cell.cycle ou 31.2 cell.division	3	52	47389.49
Tb10.61.1880	protein kinase, putative,mitogen-activated protein kinase kinase kin	31.3 cell.cycle ou 31.2 cell.division	2	52	31374.9
Tb927.8.4640	flagellar protofilament ribbon protein, putative	31.6* cell organization	1	27	46865.43

Tb10.61.1750	C-terminal motor kinesin, putative	31.6* cell organization	5	89	90800.49
Tb927.8.4970	69 kDa paraflagellar rod protein,PFR2	31.6* cell organization	3	95	69597.02
Tb927.3.4290	73 kDa paraflagellar rod protein,PFR1	31.6* cell organization	3	61	68682.76
Tb927.5.1410	65 kDa invariant surface glycoprotein	31.99 cell.unspecified	1	36	46895.49
Tb09.160.3710	proliferative cell nuclear antigen (PCNA), putative	31.3 cell.cycle	3	57	32314.61
Tb11.01.0120	haloacid dehalogenase-like hydrolase, putative	35.1 not assigned.no ontology	4	62	31584.91
Tb09.160.5530	hypothetical protein, conserved	35.1 not assigned.no ontology	5	94	38016.33
Tb09.211.0690	hypothetical protein, conserved	35.1 not assigned.no ontology	1	72	38856.16
Tb09.211.1690	hypothetical protein, conserved	35.1 not assigned.no ontology	1	41	18768.80
Tb10.26.0680	hypothetical protein, conserved	35.1 not assigned.no ontology	1	27	14328.23
Tb10.389.0720	hypothetical protein, conserved	35.1 not assigned.no ontology	5	66	110243.85
Tb10.61.0540	hypothetical protein, conserved	35.1 not assigned.no ontology	1	36	36411.55
Tb10.70.1130	hypothetical protein, conserved	35.1 not assigned.no ontology	4	69	48257.68
Tb10.70.4840	hypothetical protein, conserved	35.1 not assigned.no ontology	1	52	38588.92
Tb10.70.4930	hypothetical protein, conserved	35.1 not assigned.no ontology	1	70	32833.06
Tb11.01.5680	hypothetical protein, conserved	35.1 not assigned.no ontology	1	45	51674.18
Tb11.01.8400	hypothetical protein, conserved	35.1 not assigned.no ontology	1	20	63505.07
Tb11.02.0140	hypothetical protein, conserved	35.1 not assigned.no ontology	2	39	33848.34
Tb11.02.0300	hypothetical protein, conserved	35.1 not assigned.no ontology	1	31	69229.86
Tb11.02.2040	hypothetical protein, conserved	35.1 not assigned.no ontology	1	40	14037.89
Tb11.42.0004	hypothetical protein, conserved	35.1 not assigned.no ontology	1	38	36025.05
Tb11.55.0024	hypothetical protein, conserved	35.1 not assigned.no ontology	2	50	37610.93
Tb927.2.4580	hypothetical protein, conserved	35.1 not assigned.no ontology	2	59	22660.49
Tb927.3.1680	hypothetical protein, conserved	35.1 not assigned.no ontology	1	75	35191.36
Tb927.3.2100	hypothetical protein, conserved	35.1 not assigned.no ontology	3	55	33996.14
Tb927.3.3560	hypothetical protein, conserved	35.1 not assigned.no ontology	7	58	69087.74
Tb927.4.1300	hypothetical protein, conserved	35.1 not assigned.no ontology	2	72	42014.86
Tb927.4.2030	hypothetical protein, conserved	35.1 not assigned.no ontology	1	30	22703.92
Tb927.4.2040	hypothetical protein, conserved	35.1 not assigned.no ontology	1	40	20808.23
Tb927.4.2740	hypothetical protein, conserved	35.1 not assigned.no ontology	3	67	16327.52
Tb927.4.380	hypothetical protein, conserved	35.1 not assigned.no ontology	1	47	35612.91

Tb927.5.1460	hypothetical protein, conserved	35.1 not assigned.no ontology	2	62	36762.16
Tb927.6.4770	hypothetical protein, conserved	35.1 not assigned.no ontology	4	86	83306.48
Tb11.02.2520	hypothetical protein, conserved,predicted ankyrin repeat family prot	35.1 not assigned.no ontology	1	57	43962
Tb927.7.4290	hypothetical protein, conserved	35.2 not assigned.unknown	1	66	33703.32
Tb927.7.4520	hypothetical protein, conserved	35.2 not assigned.unknown	2	69	22315.20
Tb927.8.1170	hypothetical protein, conserved	35.2 not assigned.unknown	1	52	36419.22
Tb927.8.4230	hypothetical protein, conserved	35.2 not assigned.unknown	1	23	119022.43
Tb927.7.3440	I/6 autoantigen	35.2 not assigned.unknown	1	40	27050.07
Tb927.5.2940	stress-induced protein sti1, putative	35.2 not assigned.unknown	4	89	62327.63
Tb10.70.2770	stress-inducible protein STI1-like, putative	35.2 not assigned.unknown	5	71	28424.92
