# Supplementary material for: Exocytosis and protein secretion in Trypanosoma
Source: BMC Microbiol. 2010 Jan 26;10:20. doi: 10.1186/1471-2180-10-20 (PMC3224696; doi:10.1186/1471-2180-10-20)
Supplement: Additional file 2 — Table S2. Secreted proteins from OK strain identified on BN-PAGE gel. contains the list of the proteins identified in each spot (sheet 1), a nonredundant list of proteins classified according to their functional categories (sheet 2), and a nonredundant list of all the secreted proteins identified (BN+1D) in this study (sheet 3). [file 1471-2180-10-20-S2.PDF]

Table S2 / BN-PAGE gel

SHEET 1

BN spots - Proteins identified in each spots

Spot number	Accession number	protein name
**1**	Tb10.70.2650	elongation factor 2 [1168806 - 1166266] MW:94334.21
	Tb927.1.120	retrotransposon hot spot (RHS) protein, putative; retrotransposon hot spot protein 4 (RHS4), putati
	Tb927.1.2330	beta tubulin [570482 - 571810] MW:49703.96
	Tb927.2.5980	HSP100 ATP-dependent Clp protease subunit, heat shock protein 100 (HSP100), putative; Serine peptid
	Tb09.160.3590	PDE2C cAMP-specific phosphodiesterase [852556 - 855348] MW:103651.08
	Tb10.70.5650	TEF1 elongation factor 1-alpha [548535 - 549884] MW:49105.63
	Tb927.1.2340	alpha tubulin [572442 - 573797] MW:49787.13
	Tb927.2.100	retrotransposon hot spot (RHS) protein, putative; retrotransposon hot spot protein 1 (RHS1), putati
	Tb927.2.380	retrotransposon hot spot (RHS) protein, putative; retrotransposon hot spot protein 2 (RHS2), putati
	Tb927.1.180	retrotransposon hot spot (RHS) protein, putative; retrotransposon hot spot protein 1 (RHS1), putati
	Tb09.160.1160	hypothetical protein, conserved [521174 - 518901] MW:85913.86
	Tb927.2.6050	beta prime COP protein [1092946 - 1095501] MW:94052.12
	Tb10.61.1750	TBKIFC1 C-terminal motor kinesin, putative [3561647 - 3564109] MW:90800.49
	Tb11.01.8770	hypothetical protein, conserved; leucine-rich repeat protein (LRRP), putative [4475937 - 4472923] M
	Tb11.01.3740	coatomer gamma subunit, putative; cytosolic coat protein, putative [3120728 - 3123364] MW:97587.26
	Tb927.3.5520	RPN1 26S proteasome regulatory non-ATPase subunit [1542913 - 1545648] MW:99898.08
**2**	Tb10.389.0880	heat shock protein, putative [3016541 - 3014106] MW:90863.73
	Tb10.61.1750	TBKIFC1 C-terminal motor kinesin, putative [3561647 - 3564109] MW:90800.49
	Tb10.70.2650	elongation factor 2 [1168806 - 1166266] MW:94334.21
	Tb10.70.1190	VCP valosin-containing protein homolog; Transitional endoplasmic reticulum ATPase, putative [143224
	Tb927.7.6090	hypothetical protein, conserved [1661292 - 1659004] MW:88162.89
	Tb927.1.2340	alpha tubulin [572442 - 573797] MW:49787.13
	Tb927.1.180	retrotransposon hot spot (RHS) protein, putative; retrotransposon hot spot protein 1 (RHS1), putati
	Tb927.1.2330	beta tubulin [570482 - 571810] MW:49703.96
	Tb10.70.5650	TEF1 elongation factor 1-alpha [548535 - 549884] MW:49105.63
**3**	Tb10.26.1080	heat shock protein 83; heat shock protein [2613113 - 2610999] MW:80763.23
	Tb10.70.2650	elongation factor 2 [1168806 - 1166266] MW:94334.21
	Tb10.70.5650	TEF1 elongation factor 1-alpha [548535 - 549884] MW:49105.63

	Tb927.1.2330	beta tubulin [570482 - 571810] MW:49703.96
	Tb10.61.1750	TBKIFC1 C-terminal motor kinesin, putative [3561647 - 3564109] MW:90800.49
	Tb927.4.760	gamma-adaptin 1, putative; AP-1 adapter complex gamma subunit, putative [211089 - 208684] MW:87344.
	Tb11.55.0006	TbIFT88 intraflagellar transport protein IFT88, putative [508741 - 506339] MW:89755.06
	Tb927.5.2570	translation initiation factor, putative [825553 - 827643] MW:79811.15
	Tb11.02.0250	heat shock protein, mitochondrial precursor, putative; TNFR-associated protein 1 [759396 - 757135]
**ambigu**	Tb927.2.470	retrotransposon hot spot (RHS) protein, putative; retrotransposon hot spot protein 4 (RHS4), putati
	Tb11.01.2790	hypothetical protein, conserved [2883272 - 2884753] MW:54823.94
**4**	Tb11.01.3110	heat shock protein 70 [2971037 - 2973109] MW:75366.42
	Tb927.8.4970	PFR 69 kDa paraflagellar rod protein; PFR2 [1474818 - 1476620] MW:69597.02
	Tb927.3.4290	PFR-C; PFR-D 73 kDa paraflagellar rod protein; PFR1 [1210633 - 1208864] MW:68682.76
	Tb927.7.710	HSP70 heat shock 70 kDa protein, putative [158165 - 156246] MW:70211.63
	Tb927.3.1590	hypothetical protein, conserved [417246 - 415240] MW:72678.15
	Tb10.26.1080	heat shock protein 83; heat shock protein [2613113 - 2610999] MW:80763.23
	Tb927.1.2330	beta tubulin [570482 - 571810] MW:49703.96
	Tb10.6k15.2500	BAD1 adaptin complex 1 subunit, putative; beta-adaptin, fragment [1983280 - 1981193] MW:76279.46
	Tb927.1.2340	alpha tubulin [572442 - 573797] MW:49787.13
	Tb10.70.5650	TEF1 elongation factor 1-alpha [548535 - 549884] MW:49105.63
	Tb10.61.1590	intraflagellar transport protein component, putative [3587443 - 3589410] MW:72377.72
	Tb927.3.4040	hypothetical protein, conserved [1142118 - 1140259] MW:67631.70
	Tb927.3.5490	hypothetical protein, conserved [1535292 - 1537319] MW:77659.71
	Tb10.61.2370	hypothetical protein, conserved [3429745 - 3428078] MW:60759.48
	Tb11.01.6690	hypothetical protein, conserved [3944798 - 3943368] MW:51912.37
**5**	Tb927.5.2940	stress-induced protein sti1, putative [927151 - 928803] MW:62327.63
	Tb927.4.1080	V-type ATPase, A subunit, putative [289967 - 288135] MW:67749.33
	Tb10.70.0280	HSP60 chaperonin Hsp60, mitochondrial precursor [1644448 - 1646136] MW:59503.07
	Tb927.1.2330	beta tubulin [570482 - 571810] MW:49703.96
	Tb11.02.1120	adenylosuccinate synthetase, putative [1055003 - 1056811] MW:66674.89
	Tb10.70.7050	TCP-1-delta t-complex protein 1, delta subunit, putative [286524 - 284914] MW:58361.67
	Tb09.160.3730	glutaminyl-tRNA synthetase, putative [867532 - 869415] MW:73330.45
	Tb11.01.8510	TCP-1-alpha t-complex protein 1, alpha subunit, putative [4400794 - 4399277] MW:54623.76
	Tb927.8.1600	lysyl-tRNA synthetase, putative [531411 - 533165] MW:66786.31
	Tb10.61.2670	hypothetical protein, conserved [3367350 - 3365911] MW:55311.17

	Tb10.70.5650	TEF1 elongation factor 1-alpha [548535 - 549884] MW:49105.63
	Tb927.1.2340	alpha tubulin [572442 - 573797] MW:49787.13
	Tb11.01.5860	TCP-1-epsilon t-complex protein 1, epsilon subunit, putative [3733928 - 3735544] MW:59381.19
	Tb927.3.4290	PFR-C; PFR-D 73 kDa paraflagellar rod protein; PFR1 [1210633 - 1208864] MW:68682.76
	Tb10.61.2680	PYK1 pyruvate kinase 1 [3364197 - 3363142] MW:38453.32
	Tb11.02.0210	hypothetical protein, conserved [747704 - 746319] MW:50887.21
	Tb09.160.1180	hypothetical protein, conserved [523907 - 522177] MW:66256.87
	Tb927.3.3560	hypothetical protein, conserved [1001768 - 1003642] MW:69087.74
	Tb927.8.6660	hypothetical protein, conserved [1930528 - 1928705] MW:69055.48
	Tb927.7.6860	expression site-associated gene (ESAG) protein, putative; expression site-associated gene 5 (ESAG5)
	Tb09.211.2150	poly(A)-binding protein 1; PABP2 [1651052 - 1649385] MW:62147.04
	Tb11.02.5450	glucose-regulated protein 78, putative; luminal binding protein 1 (BiP), putative [2095561 - 209360
	Tb927.4.1850	hypothetical protein, conserved [464351 - 462615] MW:64384.15
	Tb927.6.4590	glutamyl-tRNA synthetase, putative [1285581 - 1287404] MW:68931.84
**6**	Tb927.6.5070	hypothetical protein, conserved [1383939 - 1385303] MW:51821.01
	Tb10.61.2680	PYK1 pyruvate kinase 1 [3364197 - 3363142] MW:38453.32
	Tb927.1.2330	beta tubulin [570482 - 571810] MW:49703.96
	Tb11.42.0003	TCP-1-beta t-complex protein 1, beta subunit, putative [537538 - 535949] MW:58057.86
	Tb927.1.2340	alpha tubulin [572442 - 573797] MW:49787.13
	Tb11.02.2210	PKA-R; RSU protein kinase A regulatory subunit [1302874 - 1304373] MW:56734.19
	Tb10.70.5650	TEF1 elongation factor 1-alpha [548535 - 549884] MW:49105.63
	Tb11.01.8510	TCP-1-alpha t-complex protein 1, alpha subunit, putative [4400794 - 4399277] MW:54623.76
	Tb927.2.4210	glycosomal phosphoenolpyruvate carboxykinase; glycosomal protein P60 [742706 - 741129] MW:58564.86
	Tb10.389.1070	hypothetical protein, conserved [2982652 - 2979851] MW:105847.36
	Tb927.8.1600	lysyl-tRNA synthetase, putative [531411 - 533165] MW:66786.31
	Tb11.01.4830	eIF-2-gamma eukaryotic translation initiation factor 2 gamma, putative [3494854 - 3493421] MW:52200
	Tb09.211.3540	glk1; gk glycerol kinase, glycosomal [1917219 - 1918757] MW:56335.85
	Tb10.61.0380	glycerol uptake protein, putative [3850573 - 3852402] MW:69718.53
**7**	Tb927.1.2340	alpha tubulin [572442 - 573797] MW:49787.13
	Tb927.1.2330	beta tubulin [570482 - 571810] MW:49703.96
	Tb11.02.0290	succinyl-coA:3-ketoacid-coenzyme A transferase, mitochondrial precursor, putative [772192 - 770711]
	Tb10.70.5650	TEF1 elongation factor 1-alpha [548535 - 549884] MW:49105.63
	Tb10.61.0180	peptidylprolyl isomerase-like protein, putative [3887943 - 3889220] MW:47604.30
	Tb927.4.1270	RuvB-like DNA helicase, putative [337939 - 336560] MW:49905.13

	Tb10.70.6360	RPN5 proteasome regulatory non-ATP-ase subunit 5; 19S proteasome regulatory subunit [426845 - 42539
	Tb10.61.2680	PYK1 pyruvate kinase 1 [3364197 - 3363142] MW:38453.32
	Tb09.160.5560	adenylosuccinate lyase, putative [1193479 - 1192064] MW:53119.86
	Tb11.01.1350	S-adenosylhomocysteine hydrolase, putative [2558252 - 2556939] MW:48447.50
	Tb09.211.3180	hypothetical protein, conserved [2021829 - 2020498] MW:49450.71
	Tb09.211.3540	glk1; gk glycerol kinase, glycosomal [1917219 - 1918757] MW:56335.85
**8**	Tb10.70.3290	DHH1 ATP-dependent DEAD-box RNA helicase, putative; DHH1 [1056598 - 1055378] MW:46468.25
	Tb10.70.4740	enolase [745416 - 746705] MW:46592.13
	Tb09.211.4240	phosphoinositide-binding protein, putative [2069958 - 2068699] MW:48172.33
	Tb927.1.2330	beta tubulin [570482 - 571810] MW:49703.96
	Tb927.1.2340	alpha tubulin [572442 - 573797] MW:49787.13
	Tb927.1.700	PGKC; gPGK phosphoglycerate kinase [233826 - 232504] MW:47245.76
	Tb09.211.2150	poly(A)-binding protein 1; PABP2 [1651052 - 1649385] MW:62147.04
	Tb927.8.1960	hypothetical protein, conserved [629740 - 631044] MW:48425.71
	Tb10.70.5650	TEF1 elongation factor 1-alpha [548535 - 549884] MW:49105.63
	Tb11.01.4660	elongation factor 1 gamma, putative [3437582 - 3436368] MW:46303.53
	Tb927.2.5160	chaperone protein DnaJ, putative [910842 - 912056] MW:44813.55
	Tb10.70.7480	ATP synthase, putative [186531 - 187937] MW:52900.99
	Tb927.2.2230	hypothetical protein, conserved [427069 - 425903] MW:42703.14
**9**	Tb10.70.1370	ALD fructose-bisphosphate aldolase, glycosomal, putative [1403364 - 1404482] MW:41071.12
	Tb927.1.2340	alpha tubulin [572442 - 573797] MW:49787.13
	Tb927.4.1300	hypothetical protein, conserved [342776 - 341652] MW:42014.86
	Tb927.1.2330	beta tubulin [570482 - 571810] MW:49703.96
	Tb11.01.1370	eIF-3 beta; TRIP-1 eukaryotic translation initiation factor 3 subunit, putative; TGF-beta receptor
	Tb10.61.0540	hypothetical protein, conserved [3824087 - 3825034] MW:36411.55
	Tb10.70.5360	LA La protein; RNA-binding protein, putative [606492 - 607499] MW:37659.61
	Tb09.211.0560	DRBD3 RNA-binding protein, putative; DRBD3 [1359352 - 1358369] MW:36984.69
	Tb927.6.1800	PP2C protein phosphatase 2C, putative [603410 - 602262] MW:41135.15
	Tb927.3.5050	60S ribosomal protein L4 [1417717 - 1418841] MW:41855.88
	Tb10.70.5650	TEF1 elongation factor 1-alpha [548535 - 549884] MW:49105.63
	Tb10.61.3210	hypothetical protein, conserved [3279514 - 3278519] MW:37612.28
**10**	Tb10.70.1370	ALD fructose-bisphosphate aldolase, glycosomal, putative [1403364 - 1404482] MW:41071.12

**11**	Tb11.02.4870	PSA4 proteasome alpha 7 subunit, putative [1954053 - 1953310] MW:27865.39
	Tb11.02.1085	40s ribosomal protein S4, putative [1041968 - 1042789] MW:30643.93
	Tb927.4.3550	60S ribosomal protein L13a, putative [912419 - 911751] MW:25711.55
	Tb927.4.3570	translation elongation factor 1-beta, putative [915890 - 915105] MW:28403.66
	Tb10.100.0120	proteasome alpha 5 subunit, putative; 20S proteasome subunit alpha 5 [76763 - 77503] MW:27176.51
	Tb927.4.2030	hypothetical protein, conserved [511106 - 510453] MW:22703.92
	Tb10.389.0910	60S ribosomal protein L34, putative [3009371 - 3008859] MW:19366.04
	Tb927.1.2340	alpha tubulin [572442 - 573797] MW:49787.13
	Tb10.61.1960	RPS2 40S ribosomal protein S2, putative [3521328 - 3520528] MW:28641.95
	Tb10.70.3360	40S ribosomal protein S3a, putative [1044038 - 1043268] MW:29422.06
**12**	Tb11.01.1190	ATP synthase, putative [2528152 - 2527502] MW:24788.35
	Tb09.160.4450	RPS3 40S ribosomal protein S3, putative [957257 - 958057] MW:30401.37
	Tb10.05.0220	60S ribosomal protein L10a [3224822 - 3224178] MW:24597.00
	Tb927.4.2030	hypothetical protein, conserved [511106 - 510453] MW:22703.92
	Tb927.1.2340	alpha tubulin [572442 - 573797] MW:49787.13
	Tb927.3.1120	rtb2 GTP-binding nuclear protein rtb2, putative [273590 - 274243] MW:24405.85
	Tb10.389.0910	60S ribosomal protein L34, putative [3009371 - 3008859] MW:19366.04
**13**	Tb09.160.4250	TRYP1; TXNPx tryparedoxin peroxidase [936746 - 937345] MW:22424.65
	Tb11.02.3210	TIM triosephosphate isomerase [1543092 - 1543844] MW:26818.79
	Tb10.100.0170	proteasome alpha 2 subunit, putative [84003 - 84698] MW:25354.60
	Tb10.70.5650	TEF1 elongation factor 1-alpha [548535 - 549884] MW:49105.63
	Tb927.1.2340	alpha tubulin [572442 - 573797] MW:49787.13
	Tb09.211.0110	QM 60S ribosomal protein L10, putative; QM-like protein [1285542 - 1284901] MW:24736.07
	Tb10.70.7695	40S ribosomal proteins S11, putative [146230 - 146754] MW:20087.25
	Tb10.26.0560	60S ribosomal protein L6, putative [2707740 - 2708318] MW:21184.84
	Tb10.389.0910	60S ribosomal protein L34, putative [3009371 - 3008859] MW:19366.04
**14**	Tb10.70.7695	40S ribosomal proteins S11, putative [146230 - 146754] MW:20087.25
	Tb10.70.3510	60S ribosomal protein L18a, putative [1011318 - 1010779] MW:20904.27
	Tb09.160.2550	ribosomal protein S7, putative [720303 - 720911] MW:23841.01
	Tb10.70.5650	TEF1 elongation factor 1-alpha [548535 - 549884] MW:49105.63
	Tb10.70.7010	60S ribosomal protein L9, putative [291415 - 290846] MW:21857.31
	Tb927.7.3450	I/6 autoantigen [893109 - 892522] MW:21584.99
	Tb11.01.3020	40S ribosomal protein L14, putative [2952037 - 2952606] MW:21469.27

	Tb927.1.2340	alpha tubulin [572442 - 573797] MW:49787.13
	Tb10.70.1380	40S ribosomal protein S9, putative [1402480 - 1403052] MW:22051.52
	Tb10.389.0910	60S ribosomal protein L34, putative [3009371 - 3008859] MW:19366.04
	Tb11.01.3080	heat shock protein 70, putative [2963244 - 2965274] MW:73630.61
	Tb09.211.0110	QM 60S ribosomal protein L10, putative; QM-like protein [1285542 - 1284901] MW:24736.07
	Tb927.8.1990	TRYP2 tryparedoxin peroxidase [634173 - 634853] MW:25631.46
	Tb11.02.5170	PSB3 proteasome beta 3 subunit, putative [2029089 - 2029706] MW:22483.92
	Tb11.46.0001	60S acidic ribosomal subunit protein, putative [572314 - 573288] MW:34627.22
**15**	Tb927.7.5170	60S ribosomal protein L23a; 60S ribosomal protein L25 [1362946 - 1363440] MW:18170.39
	Tb927.1.2340	alpha tubulin [572442 - 573797] MW:49787.13
	Tb10.61.2090	60S ribosomal protein L17, putative [3502175 - 3501675] MW:19105.48
**16**		
**17**	Tb927.6.4980	RPS14 40S ribosomal protein S14 [1370239 - 1370673] MW:15512.80
	Tb11.02.2040	hypothetical protein, conserved [1268639 - 1269016] MW:14037.89
	Tb09.211.2630	60S ribosomal protein L23, putative [1737671 - 1738090] MW:14962.60
	Tb927.6.3290	intraflagellar transport (IFT) protein, putative [972730 - 973122] MW:15419.41
	Tb10.406.0330	histone H2B, putative [2541174 - 2540836] MW:12569.59
	Tb11.46.0001	60S acidic ribosomal subunit protein, putative [572314 - 573288] MW:34627.22
	Tb11.01.0700	ribose 5-phosphate isomerase, putative [2408554 - 2409021] MW:16957.30
	Tb927.1.2340	alpha tubulin [572442 - 573797] MW:49787.13
	Tb09.211.4540	RNA-binding protein, putative; DRBD2 [2134994 - 2134077] MW:34618.44
**18**	Tb09.160.4200	60S acidic ribosomal protein, putative [925061 - 925402] MW:11090.51
	Tb927.2.2770	hypothetical protein, conserved [541346 - 541723] MW:13445.37
**19**	Tb927.3.4290	PFR-C; PFR-D 73 kDa paraflagellar rod protein; PFR1 [1210633 - 1208864] MW:68682.76
	Tb927.8.4970	PFR 69 kDa paraflagellar rod protein; PFR2 [1474818 - 1476620] MW:69597.02
	Tb11.01.3080	heat shock protein 70, putative [2963244 - 2965274] MW:73630.61
	Tb927.1.2340	alpha tubulin [572442 - 573797] MW:49787.13
	Tb927.1.2330	beta tubulin [570482 - 571810] MW:49703.96
**20**	Tb927.3.4290	PFR-C; PFR-D 73 kDa paraflagellar rod protein; PFR1 [1210633 - 1208864] MW:68682.76
	Tb927.8.4970	PFR 69 kDa paraflagellar rod protein; PFR2 [1474818 - 1476620] MW:69597.02

	Tb927.1.2340	alpha tubulin [572442 - 573797] MW:49787.13
	Tb927.3.4720	dynamin, putative; vacuolar sortin protein 1, putative [1336468 - 1334486] MW:73321.94
	Tb10.61.1590	intraflagellar transport protein component, putative [3587443 - 3589410] MW:72377.72
	Tb927.1.2330	beta tubulin [570482 - 571810] MW:49703.96
	Tb11.02.5450	glucose-regulated protein 78, putative; luminal binding protein 1 (BiP), putative [2095561 - 209360
	Tb927.6.1770	kinesin, putative [592460 - 590574] MW:69409.26
	Tb927.3.5490	hypothetical protein, conserved [1535292 - 1537319] MW:77659.71
	Tb927.7.3370	hypothetical protein, conserved [881750 - 879960] MW:67485.37
	Tb11.01.3110	heat shock protein 70 [2971037 - 2973109] MW:75366.42
	Tb927.7.710	HSP70 heat shock 70 kDa protein, putative [158165 - 156246] MW:70211.63
**22**	Tb10.61.2680	PYK1 pyruvate kinase 1 [3364197 - 3363142] MW:38453.32
	Tb927.2.2440	RPN6 proteasome regulatory non-ATPase subunit 6 [482150 - 480609] MW:57323.81
	Tb927.1.2340	alpha tubulin [572442 - 573797] MW:49787.13
**23**	Tb10.61.2680	PYK1 pyruvate kinase 1 [3364197 - 3363142] MW:38453.32
	Tb927.1.2340	alpha tubulin [572442 - 573797] MW:49787.13
	Tb927.1.2330	beta tubulin [570482 - 571810] MW:49703.96
	Tb927.6.5070	hypothetical protein, conserved [1383939 - 1385303] MW:51821.01
	Tb11.42.0003	TCP-1-beta t-complex protein 1, beta subunit, putative [537538 - 535949] MW:58057.86
	Tb10.389.1070	hypothetical protein, conserved [2982652 - 2979851] MW:105847.36
**24**	Tb927.1.2340	alpha tubulin [572442 - 573797] MW:49787.13
	Tb927.1.2330	beta tubulin [570482 - 571810] MW:49703.96
	Tb10.70.5650	TEF1 elongation factor 1-alpha [548535 - 549884] MW:49105.63
	Tb09.211.3180	gnD 6-phosphogluconate dehydrogenase, decarboxylating, putative [1843482 - 1844921] MW:52165.75
	Tb10.70.6360	RPN5 proteasome regulatory non-ATP-ase subunit 5; 19S proteasome regulatory subunit [426845 - 42539
	Tb10.61.0180	peptidylprolyl isomerase-like protein, putative [3887943 - 3889220] MW:47604.30
	Tb927.7.3620	tyrosyl-tRNA synthetase, putative [942837 - 940744] MW:76657.88
	Tb09.160.5560	adenylosuccinate lyase, putative [1193479 - 1192064] MW:53119.86
**25**	Tb927.1.2330	beta tubulin [570482 - 571810] MW:49703.96
	Tb10.70.4740	enolase [745416 - 746705] MW:46592.13
	Tb927.1.2340	alpha tubulin [572442 - 573797] MW:49787.13
	Tb09.211.4240	phosphoinositide-binding protein, putative [2069958 - 2068699] MW:48172.33
	Tb10.70.3290	DHH1 ATP-dependent DEAD-box RNA helicase, putative; DHH1 [1056598 - 1055378] MW:46468.25

	Tb09.211.2150	poly(A)-binding protein 1; PABP2 [1651052 - 1649385] MW:62147.04
**26**	Tb10.70.1370	ALD fructose-bisphosphate aldolase, glycosomal, putative [1403364 - 1404482] MW:41071.12
	Tb927.4.1300	hypothetical protein, conserved [342776 - 341652] MW:42014.86
	Tb927.1.2340	alpha tubulin [572442 - 573797] MW:49787.13
**27**	Tb927.8.3530	glycerol-3-phosphate dehydrogenase [NAD+], glycosomal [1058561 - 1057497] MW:37805.01
	Tb927.1.2330	beta tubulin [570482 - 571810] MW:49703.96
	Tb09.244.2730	60S ribosomal protein L5, putative [2307524 - 2308450] MW:34635.92
	Tb927.5.1460	hypothetical protein, conserved [465482 - 464502] MW:36762.16
**28**	Tb10.61.1960	RPS2 40S ribosomal protein S2, putative [3521328 - 3520528] MW:28641.95
	Tb11.01.7960	60S ribosomal protein L2, putative; 60S ribosomal protein L8, putative [4261852 - 4261070] MW:28313
	Tb927.1.2340	alpha tubulin [572442 - 573797] MW:49787.13
	Tb927.4.2030	hypothetical protein, conserved [511106 - 510453] MW:22703.92
**29**	Tb09.160.4250	TRYP1; TXNPx tryparedoxin peroxidase [936746 - 937345] MW:22424.65
	Tb11.02.3210	TIM triosephosphate isomerase [1543092 - 1543844] MW:26818.79
	Tb09.211.0110	QM 60S ribosomal protein L10, putative; QM-like protein [1285542 - 1284901] MW:24736.07
**30**	Tb11.03.0250	CYPA cyclophilin a; cyclophilin type peptidyl-prolyl cis-trans isomerase [224513 - 225046] MW:18717
	Tb09.211.4460	ADP-ribosylation factor, putative [2120958 - 2120410] MW:20651.84
	Tb09.211.4850	60S ribosomal protein L26, putative [2196000 - 2195569] MW:16454.32
**31**	Tb11.03.0250	CYPA cyclophilin a; cyclophilin type peptidyl-prolyl cis-trans isomerase [224513 - 225046] MW:18717
	Tb927.7.6090	hypothetical protein, conserved [1661292 - 1659004] MW:88162.89
	Tb10.70.2650	elongation factor 2 [1168806 - 1166266] MW:94334.21
	Tb927.1.120	retrotransposon hot spot (RHS) protein, putative; retrotransposon hot spot protein 4 (RHS4), putati
	Tb927.1.2340	alpha tubulin [572442 - 573797] MW:49787.13
	Tb927.1.420	retrotransposon hot spot (RHS) protein, putative; retrotransposon hot spot protein 5 (RHS5), putati
**32**	Tb10.26.1080	heat shock protein 83; heat shock protein [2613113 - 2610999] MW:80763.23
	Tb927.1.2340	alpha tubulin [572442 - 573797] MW:49787.13
	Tb927.4.760	gamma-adaptin 1, putative; AP-1 adapter complex gamma subunit, putative [211089 - 208684] MW:87344.
	Tb927.1.2330	beta tubulin [570482 - 571810] MW:49703.96
	Tb10.6k15.2220	eukaryotic translation initiation factor 3 subunit 8, putative [2033435 - 2031213] MW:84339.71

**33**	Tb11.v4.0040	variant surface glycoprotein (VSG), putative [191889 - 190408] MW:52763.30
**34**	Tb927.1.2340	alpha tubulin [572442 - 573797] MW:49787.13
	Tb927.1.2330	beta tubulin [570482 - 571810] MW:49703.96
	Tb10.61.2680	PYK1 pyruvate kinase 1 [3364197 - 3363142] MW:38453.32
	Tb927.6.5070	hypothetical protein, conserved [1383939 - 1385303] MW:51821.01
	Tb09.211.3540	glk1; gk glycerol kinase, glycosomal [1917219 - 1918757] MW:56335.85
**35**	Tb927.1.2340	alpha tubulin [572442 - 573797] MW:49787.13
	Tb927.1.2330	beta tubulin [570482 - 571810] MW:49703.96
	Tb10.70.5650	TEF1 elongation factor 1-alpha [548535 - 549884] MW:49105.63
**36**	Tb927.1.2330	beta tubulin [570482 - 571810] MW:49703.96
	Tb10.70.4740	enolase [745416 - 746705] MW:46592.13
	Tb09.211.4240	phosphoinositide-binding protein, putative [2069958 - 2068699] MW:48172.33
	Tb927.1.2340	alpha tubulin [572442 - 573797] MW:49787.13
	Tb09.211.2150	poly(A)-binding protein 1; PABP2 [1651052 - 1649385] MW:62147.04
	Tb927.2.5160	chaperone protein DnaJ, putative [910842 - 912056] MW:44813.55
	Tb927.1.700	PGKC; gPGK phosphoglycerate kinase [233826 - 232504] MW:47245.76
**37**	Tb927.1.2340	alpha tubulin [572442 - 573797] MW:49787.13
	Tb09.211.0620	actin A [1375684 - 1374554] MW:41895.68
**38**	Tb09.244.2730	60S ribosomal protein L5, putative [2307524 - 2308450] MW:34635.92
	Tb927.1.2340	alpha tubulin [572442 - 573797] MW:49787.13
**39**	Tb11.01.6030	proteasome regulatory non-ATP-ase subunit [3777343 - 3778167] MW:31280.91
**39**	Tb11.01.2560	40S ribosomal protein SA, putative [2829298 - 2830032] MW:27609.60
**39**	Tb927.4.2030	hypothetical protein, conserved [511106 - 510453] MW:22703.92
**39**	Tb927.3.3310	60S ribosomal protein L13, putative [851587 - 850898] MW:26639.22
**39**	Tb11.02.4700	14-3-3-like protein, putative [1912331 - 1911573] MW:29196.24
**39**	Tb10.61.1960	RPS2 40S ribosomal protein S2, putative [3521328 - 3520528] MW:28641.95
**39**	Tb927.1.2340	alpha tubulin [572442 - 573797] MW:49787.13
**39**	Tb10.70.7330	adenylate kinase, putative [214354 - 215136] MW:29764.86

**40**	Tb927.1.2340	alpha tubulin [572442 - 573797] MW:49787.13
**41**	Tb09.160.4250	TRYP1; TXNPx tryparedoxin peroxidase [936746 - 937345] MW:22424.65
	Tb11.02.3210	TIM triosephosphate isomerase [1543092 - 1543844] MW:26818.79
**42**	Tb11.03.0250	CYPA cyclophilin a; cyclophilin type peptidyl-prolyl cis-trans isomerase [224513 - 225046] MW:18717
	Tb09.211.4460	ADP-ribosylation factor, putative [2120958 - 2120410] MW:20651.84
	Tb927.1.2340	alpha tubulin [572442 - 573797] MW:49787.13
**43**	Tb11.v4.0040	variant surface glycoprotein (VSG), putative [191889 - 190408] MW:52763.30
**44**	Tb11.01.8510	TCP-1-alpha t-complex protein 1, alpha subunit, putative [4400794 - 4399277] MW:54623.76
	Tb10.70.7050	TCP-1-delta t-complex protein 1, delta subunit, putative [286524 - 284914] MW:58361.67
	Tb11.02.0750	TCP-1-zeta t-complex protein 1, zeta subunit, putative [904971 - 906605] MW:59579.38
	Tb11.01.5860	TCP-1-epsilon t-complex protein 1, epsilon subunit, putative [3733928 - 3735544] MW:59381.19
**45**	Tb11.01.8510	TCP-1-alpha t-complex protein 1, alpha subunit, putative [4400794 - 4399277] MW:54623.76
**46**	Tb927.1.2330	beta tubulin [570482 - 571810] MW:49703.96
	Tb927.1.2340	alpha tubulin [572442 - 573797] MW:49787.13
	Tb10.70.5650	TEF1 elongation factor 1-alpha [548535 - 549884] MW:49105.63
**47**	Tb10.70.1370	ALD fructose-bisphosphate aldolase, glycosomal, putative [1403364 - 1404482] MW:41071.12
**49**	Tb10.70.0850	TbPSA6 proteasome alpha 1 subunit, putative; 20S proteasome subunit alpha-6, putative [1515342 - 15
	Tb927.4.2030	hypothetical protein, conserved [511106 - 510453] MW:22703.92
**50**	Tb10.100.0120	proteasome alpha 5 subunit, putative; 20S proteasome subunit alpha 5 [76763 - 77503] MW:27176.51
	Tb11.02.4870	PSA4 proteasome alpha 7 subunit, putative [1954053 - 1953310] MW:27865.39
	Tb09.211.1250	TbPSA6 proteasome alpha 1 subunit, putative; 20S proteasome subunit alpha-6, putative [1490115 - 14
**51**	Tb10.70.3660	proteasome activator protein PA26 [965880 - 965185] MW:25257.72
	Tb11.01.1190	ATP synthase, putative [2528152 - 2527502] MW:24788.35
	Tb927.3.780	TbPSA7 proteasome alpha 7 subunit [187958 - 187245] MW:25462.76
	Tb09.211.1250	TbPSA6 proteasome alpha 1 subunit, putative; 20S proteasome subunit alpha-6, putative [1490115 - 14
	Tb927.6.1260	TbPSB1 proteasome beta-1 subunit, putative [470586 - 469738] MW:30441.39

	Tb927.4.2030	hypothetical protein, conserved [511106 - 510453] MW:22703.92
**52**	Tb10.70.3660	proteasome activator protein PA26 [965880 - 965185] MW:25257.72
	Tb09.211.2590	proteasome beta 2 subunit, putative; 20S proteasome subunit [1732494 - 1733261] MW:27402.47
	Tb09.160.4250	TRYP1; TXNPx tryparedoxin peroxidase [936746 - 937345] MW:22424.65
	Tb10.100.0170	proteasome alpha 2 subunit, putative [84003 - 84698] MW:25354.60
	Tb927.6.1260	TbPSB1 proteasome beta-1 subunit, putative [470586 - 469738] MW:30441.39
	Tb11.02.3210	TIM triosephosphate isomerase [1543092 - 1543844] MW:26818.79
	Tb927.4.430	proteasome beta 7 subunit [128728 - 128072] MW:24408.88
	Tb927.4.2030	hypothetical protein, conserved [511106 - 510453] MW:22703.92
**53**	Tb11.02.5170	PSB3 proteasome beta 3 subunit, putative [2029089 - 2029706] MW:22483.92
	Tb927.7.4790	BETA6 proteasome beta 6 subunit; 20S proteasome beta 6 subunit, putative [1267631 - 1266855] MW:286
	Tb09.160.4250	TRYP1; TXNPx tryparedoxin peroxidase [936746 - 937345] MW:22424.65
	Tb10.100.0170	proteasome alpha 2 subunit, putative [84003 - 84698] MW:25354.60
	Tb927.7.3450	I/6 autoantigen [893109 - 892522] MW:21584.99
	Tb10.70.2490	PSB4 proteasome beta 2 subunit, putative; 20S proteasome subunit [1187314 - 1186694] MW:22776.06
	Tb11.01.3110	heat shock protein 70 [2971037 - 2973109] MW:75366.42
	Tb927.1.2340	alpha tubulin [572442 - 573797] MW:49787.13
	Tb10.70.6540	HGPRT hypoxanthine-guanine phosphoribosyltransferase [390899 - 390267] MW:23371.90
	Tb09.211.2590	proteasome beta 2 subunit, putative; 20S proteasome subunit [1732494 - 1733261] MW:27402.47
	Tb11.01.3020	40S ribosomal protein L14, putative [2952037 - 2952606] MW:21469.27
	Tb927.8.5440	TB-24 flagellar calcium-binding protein TB-24 [1613150 - 1613806] MW:24253.22
	Tb927.8.6270	hypothetical protein, conserved [1822881 - 1824698] MW:69168.79
	Tb927.8.5880	eukaryotic translation initiation factor 1A, putative [1732407 - 1732925] MW:19445.67
**54**	Tb10.70.0790	PRCE proteasome beta 5 subunit, putative; proteasome beta 5 subunit [1531155 - 1530223] MW:34415.80
	Tb927.6.4990	ATP synthase, epsilon chain, putative [1370982 - 1371530] MW:20146.88
	Tb11.02.4870	PSA4 proteasome alpha 7 subunit, putative [1954053 - 1953310] MW:27865.39
**55**	Tb09.211.4460	ADP-ribosylation factor, putative [2120958 - 2120410] MW:20651.84
	Tb11.03.0250	CYPA cyclophilin a; cyclophilin type peptidyl-prolyl cis-trans isomerase [224513 - 225046] MW:18717
	Tb927.3.3450	ADP-ribosylation factor-like protein 3A, putative [979573 - 980109] MW:19880.70
	Tb09.211.0740	p21 antigen protein, putative [1398292 - 1397720] MW:21059.98
	Tb927.1.2340	alpha tubulin [572442 - 573797] MW:49787.13

**56**	Tb09.211.4460	ADP-ribosylation factor, putative [2120958 - 2120410] MW:20651.84
	Tb927.3.3450	ADP-ribosylation factor-like protein 3A, putative [979573 - 980109] MW:19880.70
	Tb11.03.0250	CYPA cyclophilin a; cyclophilin type peptidyl-prolyl cis-trans isomerase [224513 - 225046] MW:18717
	Tb11.02.4870	PSA4 proteasome alpha 7 subunit, putative [1954053 - 1953310] MW:27865.39
	Tb927.1.2340	alpha tubulin [572442 - 573797] MW:49787.13
**57**	Tb10.70.0850	TbPSA6 proteasome alpha 1 subunit, putative; 20S proteasome subunit alpha-6, putative [1515342 - 15
	Tb11.01.1290	14-3-3-like protein, putative [2547863 - 2547075] MW:30310.38
	Tb927.4.2030	hypothetical protein, conserved [511106 - 510453] MW:22703.92
	Tb11.02.4700	14-3-3-like protein, putative [1912331 - 1911573] MW:29196.24
**58**	Tb11.02.4870	PSA4 proteasome alpha 7 subunit, putative [1954053 - 1953310] MW:27865.39
	Tb10.100.0120	proteasome alpha 5 subunit, putative; 20S proteasome subunit alpha 5 [76763 - 77503] MW:27176.51
	Tb927.3.780	TbPSA7 proteasome alpha 7 subunit [187958 - 187245] MW:25462.76
	Tb927.4.3570	translation elongation factor 1-beta, putative [915890 - 915105] MW:28403.66
	Tb927.6.1260	TbPSB1 proteasome beta-1 subunit, putative [470586 - 469738] MW:30441.39
**59**	Tb11.02.4870	PSA4 proteasome alpha 7 subunit, putative [1954053 - 1953310] MW:27865.39
	Tb10.100.0120	proteasome alpha 5 subunit, putative; 20S proteasome subunit alpha 5 [76763 - 77503] MW:27176.51
	Tb927.3.780	TbPSA7 proteasome alpha 7 subunit [187958 - 187245] MW:25462.76
	Tb09.211.1250	TbPSA6 proteasome alpha 1 subunit, putative; 20S proteasome subunit alpha-6, putative [1490115 - 14
	Tb927.6.1260	TbPSB1 proteasome beta-1 subunit, putative [470586 - 469738] MW:30441.39
	Tb927.4.3590	translation elongation factor 1-beta, putative [917947 - 917162] MW:28375.65
	Tb927.4.2030	hypothetical protein, conserved [511106 - 510453] MW:22703.92
**60**	Tb927.6.1260	TbPSB1 proteasome beta-1 subunit, putative [470586 - 469738] MW:30441.39
	Tb927.3.780	TbPSA7 proteasome alpha 7 subunit [187958 - 187245] MW:25462.76
	Tb11.02.4870	PSA4 proteasome alpha 7 subunit, putative [1954053 - 1953310] MW:27865.39
	Tb09.211.1250	TbPSA6 proteasome alpha 1 subunit, putative; 20S proteasome subunit alpha-6, putative [1490115 - 14
	Tb11.01.1190	ATP synthase, putative [2528152 - 2527502] MW:24788.35
**61**	Tb09.211.2590	proteasome beta 2 subunit, putative; 20S proteasome subunit [1732494 - 1733261] MW:27402.47
	Tb927.4.430	proteasome beta 7 subunit [128728 - 128072] MW:24408.88
	Tb927.6.1260	TbPSB1 proteasome beta-1 subunit, putative [470586 - 469738] MW:30441.39
	Tb10.70.3660	proteasome activator protein PA26 [965880 - 965185] MW:25257.72
	Tb09.160.4250	TRYP1; TXNPx tryparedoxin peroxidase [936746 - 937345] MW:22424.65

	Tb09.211.1250	TbPSA6 proteasome alpha 1 subunit, putative; 20S proteasome subunit alpha-6, putative [1490115 - 14
	Tb11.01.1190	ATP synthase, putative [2528152 - 2527502] MW:24788.35
	Tb927.4.2030	hypothetical protein, conserved [511106 - 510453] MW:22703.92
**62**	Tb10.100.0170	proteasome alpha 2 subunit, putative [84003 - 84698] MW:25354.60
	Tb09.211.2590	proteasome beta 2 subunit, putative; 20S proteasome subunit [1732494 - 1733261] MW:27402.47
	Tb09.160.4250	TRYP1; TXNPx tryparedoxin peroxidase [936746 - 937345] MW:22424.65
	Tb10.70.3660	proteasome activator protein PA26 [965880 - 965185] MW:25257.72
	Tb11.02.3210	TIM triosephosphate isomerase [1543092 - 1543844] MW:26818.79
	Tb927.6.1260	TbPSB1 proteasome beta-1 subunit, putative [470586 - 469738] MW:30441.39
	Tb927.7.4790	BETA6 proteasome beta 6 subunit; 20S proteasome beta 6 subunit, putative [1267631 - 1266855] MW:286
	Tb11.02.4870	PSA4 proteasome alpha 7 subunit, putative [1954053 - 1953310] MW:27865.39
	Tb927.4.430	proteasome beta 7 subunit [128728 - 128072] MW:24408.88
	Tb927.1.2340	alpha tubulin [572442 - 573797] MW:49787.13
**63**	Tb10.100.0170	proteasome alpha 2 subunit, putative [84003 - 84698] MW:25354.60
	Tb09.160.4250	TRYP1; TXNPx tryparedoxin peroxidase [936746 - 937345] MW:22424.65
	Tb09.211.2590	proteasome beta 2 subunit, putative; 20S proteasome subunit [1732494 - 1733261] MW:27402.47
	Tb11.02.5170	PSB3 proteasome beta 3 subunit, putative [2029089 - 2029706] MW:22483.92
	Tb927.7.4790	BETA6 proteasome beta 6 subunit; 20S proteasome beta 6 subunit, putative [1267631 - 1266855] MW:286
	Tb927.7.3440	I/6 autoantigen [892212 - 891472] MW:27050.07
	Tb10.70.2490	PSB4 proteasome beta 2 subunit, putative; 20S proteasome subunit [1187314 - 1186694] MW:22776.06
	Tb11.01.3080	heat shock protein 70, putative [2963244 - 2965274] MW:73630.61
	Tb927.8.1990	TRYP2 tryparedoxin peroxidase [634173 - 634853] MW:25631.46
	Tb927.8.5440	TB-24 flagellar calcium-binding protein TB-24 [1613150 - 1613806] MW:24253.22
	Tb927.1.2340	alpha tubulin [572442 - 573797] MW:49787.13
	Tb10.70.1100	translation elongation factor 1-beta, putative [1449046 - 1448441] MW:21958.91
	Tb11.01.3020	40S ribosomal protein L14, putative [2952037 - 2952606] MW:21469.27
	Tb11.02.4870	PSA4 proteasome alpha 7 subunit, putative [1954053 - 1953310] MW:27865.39
	Tb927.8.6270	hypothetical protein, conserved [1822881 - 1824698] MW:69168.79
	Tb927.5.780	hypothetical protein, conserved [266344 - 267906] MW:55350.13
**64**	Tb10.70.0790	PRCE proteasome beta 5 subunit, putative; proteasome beta 5 subunit [1531155 - 1530223] MW:34415.80
	Tb927.6.4990	ATP synthase, epsilon chain, putative [1370982 - 1371530] MW:20146.88
**65**	Tb927.7.4420	proteasome alpha 3 subunit, putative [1176212 - 1175352] MW:32154.10

	Tb927.1.2340	alpha tubulin [572442 - 573797] MW:49787.13
	Tb10.100.0070	ATP synthase F1 subunit gamma protein, putative [71480 - 72397] MW:34370.09
	Tb09.160.0580	Mov34/MPN/PAD-1 metallopeptidase, putative; metallo-peptidase, Clan MP, Family M67 [378119 - 377286
**66**	Tb927.4.1080	V-type ATPase, A subunit, putative [289967 - 288135] MW:67749.33
	Tb11.02.1120	adenylosuccinate synthetase, putative [1055003 - 1056811] MW:66674.89
	Tb927.5.2940	stress-induced protein sti1, putative [927151 - 928803] MW:62327.63
	Tb927.8.1600	lysyl-tRNA synthetase, putative [531411 - 533165] MW:66786.31
	Tb10.70.0280	HSP60 chaperonin Hsp60, mitochondrial precursor [1644448 - 1646136] MW:59503.07
	Tb927.1.2340	alpha tubulin [572442 - 573797] MW:49787.13
	Tb927.3.3560	hypothetical protein, conserved [1001768 - 1003642] MW:69087.74
	Tb11.02.5450	glucose-regulated protein 78, putative; luminal binding protein 1 (BiP), putative [2095561 - 209360
	Tb11.02.0210	hypothetical protein, conserved [747704 - 746319] MW:50887.21
	Tb927.3.4290	PFR-C; PFR-D 73 kDa paraflagellar rod protein; PFR1 [1210633 - 1208864] MW:68682.76
	Tb11.01.3080	heat shock protein 70, putative [2963244 - 2965274] MW:73630.61
**67**	Tb10.70.1190	VCP valosin-containing protein homolog; Transitional endoplasmic reticulum ATPase, putative [143224
	Tb10.389.0880	heat shock protein, putative [3016541 - 3014106] MW:90863.73
	Tb10.26.1080	heat shock protein 83; heat shock protein [2613113 - 2610999] MW:80763.23
**68**	Tb11.02.1120	adenylosuccinate synthetase, putative [1055003 - 1056811] MW:66674.89
	Tb927.4.1080	V-type ATPase, A subunit, putative [289967 - 288135] MW:67749.33
	Tb927.5.2940	stress-induced protein sti1, putative [927151 - 928803] MW:62327.63
**71**	Tb927.3.3410	aspartyl aminopeptidase, putative; metallo-peptidase, Clan MH, Family M20 [872519 - 871164] MW:4932
	Tb11.02.4440	aminopeptidase, putative; metallo-peptidase, Clan MF, Family M17 [1830540 - 1828975] MW:55359.24
	Tb11.01.1350	S-adenosylhomocysteine hydrolase, putative [2558252 - 2556939] MW:48447.50
	Tb927.2.5160	chaperone protein DnaJ, putative [910842 - 912056] MW:44813.55
	Tb10.70.4740	enolase [745416 - 746705] MW:46592.13
**72**	Tb09.160.4250	TRYP1; TXNPx tryparedoxin peroxidase [936746 - 937345] MW:22424.65
	Tb10.100.0170	proteasome alpha 2 subunit, putative [84003 - 84698] MW:25354.60
	Tb11.02.3210	TIM triosephosphate isomerase [1543092 - 1543844] MW:26818.79
	Tb927.3.3410	aspartyl aminopeptidase, putative; metallo-peptidase, Clan MH, Family M20 [872519 - 871164] MW:4932
	Tb10.70.1100	translation elongation factor 1-beta, putative [1449046 - 1448441] MW:21958.91

**73**	Tb10.406.0650	microtubule-associated protein, putative [2492839 - 2486066] MW:254641.15
	Tb927.5.4570	hypothetical protein, conserved [1356572 - 1359028] MW:88866.90
	Tb11.02.4440	aminopeptidase, putative; metallo-peptidase, Clan MF, Family M17 [1830540 - 1828975] MW:55359.24
**74**	Tb10.26.1080	heat shock protein 83; heat shock protein [2613113 - 2610999] MW:80763.23
**75**	Tb927.3.4290	PFR-C; PFR-D 73 kDa paraflagellar rod protein; PFR1 [1210633 - 1208864] MW:68682.76
	Tb927.8.4970	PFR 69 kDa paraflagellar rod protein; PFR2 [1474818 - 1476620] MW:69597.02
	Tb11.01.3110	heat shock protein 70 [2971037 - 2973109] MW:75366.42
	Tb10.26.1080	heat shock protein 83; heat shock protein [2613113 - 2610999] MW:80763.23
	Tb11.02.1120	adenylosuccinate synthetase, putative [1055003 - 1056811] MW:66674.89
**76**	Tb11.02.1120	adenylosuccinate synthetase, putative [1055003 - 1056811] MW:66674.89
	Tb927.5.2940	stress-induced protein sti1, putative [927151 - 928803] MW:62327.63
	Tb11.16.0003	variant surface glycoprotein (VSG), putative [4969971 - 4971455] MW:51467.19
	Tb11.47.0001	65 kDa invariant surface glycoprotein-like protein [428987 - 430300] MW:47801.74
**77**	Tb11.01.1350	S-adenosylhomocysteine hydrolase, putative [2558252 - 2556939] MW:48447.50
	Tb11.02.1120	adenylosuccinate synthetase, putative [1055003 - 1056811] MW:66674.89
	Tb927.3.4680	RAB GDP dissociation inhibitor alpha, putative [1325313 - 1323976] MW:49396.71
	Tb11.01.3110	heat shock protein 70 [2971037 - 2973109] MW:75366.42
	Tb927.1.2340	alpha tubulin [572442 - 573797] MW:49787.13
**78**	Tb10.70.4740	enolase [745416 - 746705] MW:46592.13
	Tb11.01.4660	elongation factor 1 gamma, putative [3437582 - 3436368] MW:46303.53
	Tb09.211.4240	phosphoinositide-binding protein, putative [2069958 - 2068699] MW:48172.33
	Tb927.2.5160	chaperone protein DnaJ, putative [910842 - 912056] MW:44813.55
	Tb927.1.2330	beta tubulin [570482 - 571810] MW:49703.96
	Tb927.1.2340	alpha tubulin [572442 - 573797] MW:49787.13
	Tb11.01.1350	S-adenosylhomocysteine hydrolase, putative [2558252 - 2556939] MW:48447.50
**79**	Tb10.70.1370	ALD fructose-bisphosphate aldolase, glycosomal, putative [1403364 - 1404482] MW:41071.12
	Tb927.4.1300	hypothetical protein, conserved [342776 - 341652] MW:42014.86
	Tb10.61.3210	hypothetical protein, conserved [3279514 - 3278519] MW:37612.28
**80**	Tb927.5.1460	hypothetical protein, conserved [465482 - 464502] MW:36762.16

	Tb10.6k15.3850	GAP glyceraldehyde 3-phosphate dehydrogenase, cytosolic [1726753 - 1727748] MW:35610.62
	Tb09.211.0560	DRBD3 RNA-binding protein, putative; DRBD3 [1359352 - 1358369] MW:36984.69
	Tb09.244.2730	60S ribosomal protein L5, putative [2307524 - 2308450] MW:34635.92
**81**	Tb10.6k15.3850	GAP glyceraldehyde 3-phosphate dehydrogenase, cytosolic [1726753 - 1727748] MW:35610.62
	Tb927.5.3820	aspartate carbamoyltransferase, putative [1191671 - 1192654] MW:35910.64
	Tb09.244.2730	60S ribosomal protein L5, putative [2307524 - 2308450] MW:34635.92
	Tb09.160.5200	hypothetical protein, conserved [1103278 - 1104165] MW:32572.74
**82**	Tb927.7.7040	methylthioadenosine phosphorylase, putative [2013549 - 2012620] MW:33443.37
	Tb10.6k15.3850	GAP glyceraldehyde 3-phosphate dehydrogenase, cytosolic [1726753 - 1727748] MW:35610.62
**83**	Tb927.4.2030	hypothetical protein, conserved [511106 - 510453] MW:22703.92
	Tb11.02.4700	14-3-3-like protein, putative [1912331 - 1911573] MW:29196.24
	Tb10.70.7330	adenylate kinase, putative [214354 - 215136] MW:29764.86
	Tb09.211.0120	nascent polypeptide associated complex subunit, putative [1286359 - 1285793] MW:20129.46
	Tb11.01.1290	14-3-3-like protein, putative [2547863 - 2547075] MW:30310.38
	Tb10.6k15.3850	GAP glyceraldehyde 3-phosphate dehydrogenase, cytosolic [1726753 - 1727748] MW:35610.62
**84**	Tb927.4.3590	translation elongation factor 1-beta, putative [917947 - 917162] MW:28375.65
	Tb10.70.6610	hypothetical protein, conserved [374200 - 373463] MW:27338.99
	Tb927.4.2030	hypothetical protein, conserved [511106 - 510453] MW:22703.92
	Tb11.02.4870	PSA4 proteasome alpha 7 subunit, putative [1954053 - 1953310] MW:27865.39
**86**	Tb09.211.4460	ADP-ribosylation factor, putative [2120958 - 2120410] MW:20651.84
	Tb11.03.0250	CYPA cyclophilin a; cyclophilin type peptidyl-prolyl cis-trans isomerase [224513 - 225046] MW:18717
**87**	Tb09.211.4460	ADP-ribosylation factor, putative [2120958 - 2120410] MW:20651.84
	Tb927.7.4770	PPIase cyclophilin-type peptidyl-prolyl cis-trans isomerase, putative [1265057 - 1264536] MW:18589.
	Tb11.03.0250	CYPA cyclophilin a; cyclophilin type peptidyl-prolyl cis-trans isomerase [224513 - 225046] MW:18717
	Tb09.211.4550	60S ribosomal protein L12, putative [2136698 - 2136039] MW:24017.29
**88**	Tb11.02.0815	ubiquitin-conjugating enzyme, putative; ubiquitin-conjugating enzyme-like, putative [948990 - 94940
	Tb927.7.4770	PPIase cyclophilin-type peptidyl-prolyl cis-trans isomerase, putative [1265057 - 1264536] MW:18589.
	Tb11.01.7800	NDPK nucleoside diphosphate kinase [4213191 - 4212730] MW:16857.24
	Tb11.46.0001	60S acidic ribosomal subunit protein, putative [572314 - 573288] MW:34627.22

**89**	Tb10.26.1080	heat shock protein 83; heat shock protein [2613113 - 2610999] MW:80763.23
**90**	Tb10.70.2650	elongation factor 2 [1168806 - 1166266] MW:94334.21
	Tb10.26.1080	heat shock protein 83; heat shock protein [2613113 - 2610999] MW:80763.23
	Tb10.389.0880	heat shock protein, putative [3016541 - 3014106] MW:90863.73
	Tb10.406.0650	microtubule-associated protein, putative [2492839 - 2486066] MW:254641.15
	Tb927.2.5980	HSP100 ATP-dependent Clp protease subunit, heat shock protein 100 (HSP100), putative; Serine peptid
	Tb09.160.3590	PDE2C cAMP-specific phosphodiesterase [852556 - 855348] MW:103651.08
	Tb927.2.100	retrotransposon hot spot (RHS) protein, putative; retrotransposon hot spot protein 1 (RHS1), putati
	Tb927.5.1090	threonyl-tRNA synthetase, putative [352618 - 355017] MW:90983.63
**91**	Tb10.26.1080	heat shock protein 83; heat shock protein [2613113 - 2610999] MW:80763.23
**92**	Tb11.01.3110	heat shock protein 70 [2971037 - 2973109] MW:75366.42
	Tb927.7.710	HSP70 heat shock 70 kDa protein, putative [158165 - 156246] MW:70211.63
	Tb10.26.1080	heat shock protein 83; heat shock protein [2613113 - 2610999] MW:80763.23
	Tb11.02.5450	glucose-regulated protein 78, putative; luminal binding protein 1 (BiP), putative [2095561 - 209360
**97**	Tb11.01.8520	glucosamine-6-phosphate isomerase, putative [4403503 - 4402661] MW:31087.32
	Tb927.6.1570	2-hydroxy-3-oxopropionate reductase, putative [544399 - 543497] MW:31752.74
	Tb927.4.2030	hypothetical protein, conserved [511106 - 510453] MW:22703.92
**99**	Tb927.4.3590	translation elongation factor 1-beta, putative [917947 - 917162] MW:28375.65
	Tb10.70.1100	translation elongation factor 1-beta, putative [1449046 - 1448441] MW:21958.91
	Tb927.4.2040	hypothetical protein, conserved [513261 - 512689] MW:20808.23
	Tb927.5.1700	replication Factor A 28 kDa subunit, putative [537824 - 538591] MW:27592.91
	Tb10.70.6610	hypothetical protein, conserved [374200 - 373463] MW:27338.99
	Tb11.46.0001	60S acidic ribosomal subunit protein, putative [572314 - 573288] MW:34627.22
	Tb10.70.5650	TEF1 elongation factor 1-alpha [548535 - 549884] MW:49105.63
	Tb10.70.2770	stress-inducible protein STI1-like, putative [1145116 - 1144343] MW:28424.92
	Tb10.70.3070	hypothetical protein, conserved [1100000 - 1099257] MW:28189.10
100	Tb10.70.1100	translation elongation factor 1-beta, putative [1449046 - 1448441] MW:21958.91
	Tb11.01.3110	heat shock protein 70 [2971037 - 2973109] MW:75366.42
	Tb10.70.6540	HGPRT hypoxanthine-guanine phosphoribosyltransferase [390899 - 390267] MW:23371.90

	Tb927.7.570	prefoldin, putative [117587 - 116994] MW:22753.76
	Tb927.7.3450	I/6 autoantigen [893109 - 892522] MW:21584.99
	Tb927.6.2170	co-chaperone GrpE, putative [691093 - 691761] MW:23877.13
	Tb927.8.5880	eukaryotic translation initiation factor 1A, putative [1732407 - 1732925] MW:19445.67
	Tb11.01.6670	X3; RAB11B small GTPase [3940046 - 3939447] MW:22782.18
	Tb11.03.0620	UBP1 RNA-binding protein, putative [119615 - 118956] MW:24295.10
101	Tb11.01.3080	heat shock protein 70, putative [2963244 - 2965274] MW:73630.61
	Tb11.01.7550	iron superoxide dismutase [4153236 - 4152640] MW:22047.76
	Tb09.211.4610	vesicle-associated membrane protein, putative [2146972 - 2146355] MW:23167.44
102	Tb09.211.4460	ADP-ribosylation factor, putative [2120958 - 2120410] MW:20651.84
	Tb11.03.0250	CYPA cyclophilin a; cyclophilin type peptidyl-prolyl cis-trans isomerase [224513 - 225046] MW:18717
	Tb09.211.0740	p21 antigen protein, putative [1398292 - 1397720] MW:21059.98
	Tb09.211.4550	60S ribosomal protein L12, putative [2136698 - 2136039] MW:24017.29
	Tb927.6.2200	hypothetical protein, conserved [698549 - 699142] MW:21125.70
	Tb10.61.1750	TBKIFC1 C-terminal motor kinesin, putative [3561647 - 3564109] MW:90800.49
	Tb927.3.3450	ADP-ribosylation factor-like protein 3A, putative [979573 - 980109] MW:19880.70
	Tb927.7.4770	PPIase cyclophilin-type peptidyl-prolyl cis-trans isomerase, putative [1265057 - 1264536] MW:18589
103	Tb11.01.7800	NDPK nucleoside diphosphate kinase [4213191 - 4212730] MW:16857.24
	Tb11.02.0815	ubiquitin-conjugating enzyme, putative; ubiquitin-conjugating enzyme-like, putative [948990 - 94940
	Tb11.02.2040	hypothetical protein, conserved [1268639 - 1269016] MW:14037.89
	Tb11.46.0001	60S acidic ribosomal subunit protein, putative [572314 - 573288] MW:34627.22
	Tb927.5.1000	ubiquitin-conjugating enzyme E2, putative; ubiquitin-protein ligase, putative; ubiquitin carrier protein, putative
104	Tb927.3.5180	cofilin/actin depolymerizing factor, putative [1453906 - 1454316] MW:15119.06
	Tb11.02.2040	hypothetical protein, conserved [1268639 - 1269016] MW:14037.89
	Tb10.70.0830	CHC clathrin heavy chain [1523188 - 1518077] MW:190625.63
105	Tb927.7.1320	HSP10 10 kDa heat shock protein, putative [341451 - 341753] MW:10670.40
	Tb09.160.0465	hypothetical protein, conserved [351811 - 351554] MW:9759.29
	Tb11.50.0007	dynein light chain, putative [173608 - 173336] MW:10428.91
106	Tb11.02.1070	aminopeptidase, putative; metallo-peptidase, Clan MA(E) Family M1 [1037043 - 1039658] MW:98099.41
	Tb10.389.0880	heat shock protein, putative [3016541 - 3014106] MW:90863.73

	Tb10.70.2650	elongation factor 2 [1168806 - 1166266] MW:94334.21
	Tb927.1.180	retrotransposon hot spot (RHS) protein, putative; retrotransposon hot spot protein 1 (RHS1), putati
	Tb927.2.100	retrotransposon hot spot (RHS) protein, putative; retrotransposon hot spot protein 1 (RHS1), putati
	Tb927.2.5980	HSP100 ATP-dependent Clp protease subunit, heat shock protein 100 (HSP100), putative; Serine peptid
	Tb10.26.1080	heat shock protein 83; heat shock protein [2613113 - 2610999] MW:80763.23
	Tb927.1.4480	hypothetical protein, conserved [923539 - 927993] MW:158447.08
107	Tb10.389.0880	heat shock protein, putative [3016541 - 3014106] MW:90863.73
	Tb10.70.2650	elongation factor 2 [1168806 - 1166266] MW:94334.21
	Tb927.1.180	retrotransposon hot spot (RHS) protein, putative; retrotransposon hot spot protein 1 (RHS1), putati
	Tb927.2.100	retrotransposon hot spot (RHS) protein, putative; retrotransposon hot spot protein 1 (RHS1), putati
	Tb11.02.1070	aminopeptidase, putative; metallo-peptidase, Clan MA(E) Family M1 [1037043 - 1039658] MW:98099.41
	Tb10.26.1080	heat shock protein 83; heat shock protein [2613113 - 2610999] MW:80763.23
	Tb927.6.1290	hypothetical protein, conserved [475628 - 472929] MW:100579.20
	Tb927.2.470	retrotransposon hot spot (RHS) protein, putative; retrotransposon hot spot protein 4 (RHS4), putati
	Tb927.2.5980	HSP100 ATP-dependent Clp protease subunit, heat shock protein 100 (HSP100), putative; Serine peptid
	Tb927.2.1180	retrotransposon hot spot (RHS) protein, putative; retrotransposon hot spot protein 3 (RHS3), putati
108	Tb10.70.2650	elongation factor 2 [1168806 - 1166266] MW:94334.21
	Tb927.1.180	retrotransposon hot spot (RHS) protein, putative; retrotransposon hot spot protein 1 (RHS1), putati
	Tb927.2.100	retrotransposon hot spot (RHS) protein, putative; retrotransposon hot spot protein 1 (RHS1), putati
	Tb927.7.5210	hypothetical protein, conserved [1372419 - 1375115] MW:100479.79
	Tb10.389.0880	heat shock protein, putative [3016541 - 3014106] MW:90863.73
	Tb11.01.3110	heat shock protein 70 [2971037 - 2973109] MW:75366.42
	Tb10.26.1080	heat shock protein 83; heat shock protein [2613113 - 2610999] MW:80763.23
	Tb927.3.4750	aminopeptidase, putative; metallo-peptidase, Clan MA(E) Family M1 [1342356 - 1339741] MW:97251.35
	Tb10.70.4740	enolase [745416 - 746705] MW:46592.13
	Tb927.2.280	retrotransposon hot spot (RHS) protein, putative; retrotransposon hot spot protein 2 (RHS2), putati
	Tb927.2.340	retrotransposon hot spot (RHS) protein, putative; retrotransposon hot spot protein 4 (RHS4), putati
109	Tb11.01.7010	hypothetical protein, conserved [4017280 - 4014062] MW:117882.50
	Tb10.70.2650	elongation factor 2 [1168806 - 1166266] MW:94334.21
	Tb10.70.4740	enolase [745416 - 746705] MW:46592.13
	Tb927.1.2340	alpha tubulin [572442 - 573797] MW:49787.13
	Tb11.01.3110	heat shock protein 70 [2971037 - 2973109] MW:75366.42
	Tb927.6.700	alanyl-tRNA synthetase, putative [296642 - 293742] MW:105913.11

	Tb11.52.0013	hypothetical protein, conserved [3407660 - 3406476] MW:43332.89
	Tb10.26.1080	heat shock protein 83; heat shock protein [2613113 - 2610999] MW:80763.23
	Tb09.160.5550	calpain-like cysteine peptidase, putative; cysteine peptidase, Clan CA, family C2, putative [119072
	Tb927.7.5210	hypothetical protein, conserved [1372419 - 1375115] MW:100479.79
	Tb927.8.7020	peptidase, putative; metallo-peptidase, Clan ME, Family M16 [2018034 - 2021228] MW:118860.34
	Tb927.1.2330	beta tubulin [570482 - 571810] MW:49703.96
	Tb927.3.4750	aminopeptidase, putative; metallo-peptidase, Clan MA(E) Family M1 [1342356 - 1339741] MW:97251.35
110	Tb11.02.1210	LeuRS leucyl-tRNA synthetase, putative [1078518 - 1081742] MW:121824.99
	Tb10.6k15.1220	IleRS isoleucyl-tRNA synthetase, putative [2236069 - 2239500] MW:130603.20
	Tb10.389.0880	heat shock protein, putative [3016541 - 3014106] MW:90863.73
	Tb11.01.7010	hypothetical protein, conserved [4017280 - 4014062] MW:117882.50
	Tb927.6.4480	ValRS valyl-tRNA synthetase, putative [1260182 - 1257255] MW:110922.46
	Tb10.70.2650	elongation factor 2 [1168806 - 1166266] MW:94334.21
	Tb927.1.2340	alpha tubulin [572442 - 573797] MW:49787.13
	Tb927.8.2640	UBA1 ubiquitin-activating enzyme E1, putative [779218 - 782385] MW:116406.51
	Tb11.01.3110	heat shock protein 70 [2971037 - 2973109] MW:75366.42
111	Tb10.26.1080	heat shock protein 83; heat shock protein [2613113 - 2610999] MW:80763.23
	Tb10.389.0880	heat shock protein, putative [3016541 - 3014106] MW:90863.73
	Tb11.01.3110	heat shock protein 70 [2971037 - 2973109] MW:75366.42
	Tb10.70.6470	MetRS methionyl-tRNA synthetase, putative [403376 - 401055] MW:86910.83
	Tb10.6k15.2290	BS2 protein disulfide isomerase; bloodstream- specific protein 2 precursor [2023751 - 2022258] MW:5
	Tb10.70.2650	elongation factor 2 [1168806 - 1166266] MW:94334.21
	Tb927.8.3680	hypothetical protein, conserved [1102829 - 1100892] MW:69597.10
112	Tb11.02.5450	glucose-regulated protein 78, putative; luminal binding protein 1 (BiP), putative [2095561 - 209360
	Tb927.2.4370	TRYS; TrS trypanothione synthetase, putative [772890 - 771007] MW:71612.91
	Tb11.01.3110	heat shock protein 70 [2971037 - 2973109] MW:75366.42
	Tb927.7.2100	GMP synthase, putative; glutamine amidotransferase, putative [534639 - 532663] MW:71794.49
	Tb927.3.3560	hypothetical protein, conserved [1001768 - 1003642] MW:69087.74
	Tb927.6.3740	heat shock 70 kDa protein, mitochondrial precursor, putative [1114866 - 1112893] MW:71474.85
	Tb927.6.4590	glutamyl-tRNA synthetase, putative [1285581 - 1287404] MW:68931.84
113	Tb10.26.1080	heat shock protein 83; heat shock protein [2613113 - 2610999] MW:80763.23
	Tb11.01.3110	heat shock protein 70 [2971037 - 2973109] MW:75366.42

	Tb927.3.4840	ubiquitin hydrolase, putative; cysteine peptidase, Clan CA, family C19, putative [1364198 - 1362003
114	Tb11.01.3110	heat shock protein 70 [2971037 - 2973109] MW:75366.42
	Tb10.6k15.2520	prolyl oligopeptidase, putative; serine peptidase clan SC, family S9A, putative [1978365 - 1976269]
	Tb11.02.5450	glucose-regulated protein 78, putative; luminal binding protein 1 (BiP), putative [2095561 - 209360
	Tb11.46.0008	arginyl-tRNA synthetase, putative [558094 - 560172] MW:78432.50
	Tb10.6k15.2290	BS2 protein disulfide isomerase; bloodstream- specific protein 2 precursor [2023751 - 2022258] MW:5
	Tb927.7.190	OPA thimet oligopeptidase A, putative; metallo-peptidase, Clan MA(E) Family M3, putative [34206 - 3
	Tb927.3.3560	hypothetical protein, conserved [1001768 - 1003642] MW:69087.74
115	Tb11.01.3110	heat shock protein 70 [2971037 - 2973109] MW:75366.42
	Tb10.70.0280	HSP60 chaperonin Hsp60, mitochondrial precursor [1644448 - 1646136] MW:59503.07
	Tb927.5.2940	stress-induced protein sti1, putative [927151 - 928803] MW:62327.63
	Tb11.02.5450	glucose-regulated protein 78, putative; luminal binding protein 1 (BiP), putative [2095561 - 209360
	Tb927.3.3560	hypothetical protein, conserved [1001768 - 1003642] MW:69087.74
	Tb10.6k15.2290	BS2 protein disulfide isomerase; bloodstream- specific protein 2 precursor [2023751 - 2022258] MW:5
	Tb927.4.1080	V-type ATPase, A subunit, putative [289967 - 288135] MW:67749.33
	Tb10.6k15.2620	PGAM 2,3-bisphosphoglycerate-independent phosphoglycerate mutase [1961899 - 1960244] MW:60603.34
	Tb927.6.4590	glutamyl-tRNA synthetase, putative [1285581 - 1287404] MW:68931.84
	Tb10.26.1080	heat shock protein 83; heat shock protein [2613113 - 2610999] MW:80763.23
	Tb927.7.710	HSP70 heat shock 70 kDa protein, putative [158165 - 156246] MW:70211.63
ambiguite	Tb10.61.1750	TBKIFC1 C-terminal motor kinesin, putative [3561647 - 3564109] MW:90800.49
	Tb09.160.5530	hypothetical protein, conserved [1184832 - 1183792] MW:38016.33
	Tb927.1.2340	alpha tubulin [572442 - 573797] MW:49787.13
	Tb10.70.0830	CHC clathrin heavy chain [1523188 - 1518077] MW:190625.63
116	Tb10.6k15.2620	PGAM 2,3-bisphosphoglycerate-independent phosphoglycerate mutase [1961899 - 1960244] MW:60603.34
	Tb10.70.0280	HSP60 chaperonin Hsp60, mitochondrial precursor [1644448 - 1646136] MW:59503.07
	Tb11.01.3110	heat shock protein 70 [2971037 - 2973109] MW:75366.42
	Tb10.6k15.2330	TCP-1-theta t-complex protein 1, theta subunit, putative; CCT-theta, putative [2017097 - 2015484] M
	Tb927.8.980	phosphoacetylglucosamine mutase, putative; acetylglucosaminephosphomutase, putative; N-acetylglucos
117	Tb11.02.0100	carboxypeptidase, putative; metallo-peptidase, Clan MA(E) Family M32 [717422 - 715911] MW:57683.01
	Tb11.02.2210	PKA-R; RSU protein kinase A regulatory subunit [1302874 - 1304373] MW:56734.19
	Tb10.70.1130	hypothetical protein, conserved [1441960 - 1440659] MW:48257.68
	Tb927.1.3950	alanine aminotransferase, putative [840682 - 842391] MW:63137.20

	Tb10.70.4740	enolase [745416 - 746705] MW:46592.13
	Tb11.42.0003	TCP-1-beta t-complex protein 1, beta subunit, putative [537538 - 535949] MW:58057.86
	Tb927.1.2340	alpha tubulin [572442 - 573797] MW:49787.13
	Tb10.61.2680	PYK1 pyruvate kinase 1 [3364197 - 3363142] MW:38453.32
	Tb09.211.4330	aminopeptidase P, putative; metallo-peptidase, Clan MG, Family M24 [2089342 - 2087873] MW:54408.19
	Tb927.2.3800	hypothetical protein, conserved [679117 - 677639] MW:55460.11
	Tb927.2.3800	hypothetical protein, conserved [679117 - 677639] MW:55460.11
	Tb927.7.2240	hypothetical protein, conserved [580626 - 578992] MW:59665.51
	Tb11.02.5020	seryl-tRNA synthetase, putative [1983474 - 1982035] MW:53857.17
	Tb927.1.1100	hypothetical protein, conserved [308704 - 311616] MW:108810.61
118	Tb10.70.1130	hypothetical protein, conserved [1441960 - 1440659] MW:48257.68
	Tb10.389.1480	cytosolic nonspecific dipeptidase, putative; peptidase (M20/M25/M40 family), putative [2908817 - 29
	Tb10.61.0180	peptidylprolyl isomerase-like protein, putative [3887943 - 3889220] MW:47604.30
	Tb10.70.4740	enolase [745416 - 746705] MW:46592.13
	Tb927.1.3950	alanine aminotransferase, putative [840682 - 842391] MW:63137.20
	Tb927.1.2340	alpha tubulin [572442 - 573797] MW:49787.13
	Tb09.211.3180	gnD 6-phosphogluconate dehydrogenase, decarboxylating, putative [1843482 - 1844921] MW:52165.75
	Tb11.01.0870	replication factor A, 51kDa subunit, putative [2447406 - 2448797] MW:52331.41
	Tb10.406.0520	trypanothione reductase [2521506 - 2520028] MW:53156.79
	Tb927.6.400	peptidase M20/M25/M40, putative [190663 - 192090] MW:52185.07
	Tb927.4.730	hypothetical protein, conserved [205139 - 203661] MW:55016.63
	Tb10.70.7730	ATP-dependent DEAD/H RNA helicase, putative; DEAD box RNA helicase, putative [139933 - 141243] MW:4
	Tb11.01.1350	S-adenosylhomocysteine hydrolase, putative [2558252 - 2556939] MW:48447.50
	Tb11.01.3110	heat shock protein 70 [2971037 - 2973109] MW:75366.42
	Tb927.1.2330	beta tubulin [570482 - 571810] MW:49703.96
	Tb10.61.2680	PYK1 pyruvate kinase 1 [3364197 - 3363142] MW:38453.32
119	Tb10.70.1130	hypothetical protein, conserved [1441960 - 1440659] MW:48257.68
	Tb10.70.4740	enolase [745416 - 746705] MW:46592.13
	Tb09.211.4330	aminopeptidase P, putative; metallo-peptidase, Clan MG, Family M24 [2089342 - 2087873] MW:54408.19
	Tb927.1.2340	alpha tubulin [572442 - 573797] MW:49787.13
	Tb927.7.2240	hypothetical protein, conserved [580626 - 578992] MW:59665.51
	Tb927.1.2330	beta tubulin [570482 - 571810] MW:49703.96
	Tb10.61.2680	PYK1 pyruvate kinase 1 [3364197 - 3363142] MW:38453.32

120	Tb10.70.0280	HSP60 chaperonin Hsp60, mitochondrial precursor [1644448 - 1646136] MW:59503.07
	Tb10.6k15.2620	PGAM 2,3-bisphosphoglycerate-independent phosphoglycerate mutase [1961899 - 1960244] MW:60603.34
	Tb927.1.2340	alpha tubulin [572442 - 573797] MW:49787.13
	Tb10.70.4740	enolase [745416 - 746705] MW:46592.13
	Tb10.61.2680	PYK1 pyruvate kinase 1 [3364197 - 3363142] MW:38453.32
	Tb927.4.3790	ubiquitin carboxyl-terminal hydrolase, putative; cysteine peptidase, Clan CA, family C19, putative
	Tb10.70.1130	hypothetical protein, conserved [1441960 - 1440659] MW:48257.68
ambiguity witl	Tb927.2.470	retrotransposon hot spot (RHS) protein, putative; retrotransposon hot spot protein 4 (RHS4), putati
	Tb11.01.6510	hypothetical protein, conserved [3892801 - 3891686] MW:41086.00
	Tb11.02.2210	PKA-R; RSU protein kinase A regulatory subunit [1302874 - 1304373] MW:56734.19
	Tb11.01.3110	heat shock protein 70 [2971037 - 2973109] MW:75366.42
	Tb11.01.8510	TCP-1-alpha t-complex protein 1, alpha subunit, putative [4400794 - 4399277] MW:54623.76
	Tb10.26.1080	heat shock protein 83; heat shock protein [2613113 - 2610999] MW:80763.23
	Tb10.61.0980	gMDH glycosomal malate dehydrogenase [3714549 - 3715520] MW:33710.35
	Tb927.7.180	hypothetical protein [28385 - 27072] MW:49811.88
121	Tb10.70.4740	enolase [745416 - 746705] MW:46592.13
	Tb10.389.1730	protein kinase, putative; tyrosine protein kinase, putative [2856535 - 2857776] MW:47389.49
	Tb927.1.700	PGKC; gPGK phosphoglycerate kinase [233826 - 232504] MW:47245.76
	Tb10.70.7730	ATP-dependent DEAD/H RNA helicase, putative; DEAD box RNA helicase, putative [139933 - 141243] MW:4
	Tb927.6.3110	protein kinase, putative; cdc2, putative [919976 - 921295] MW:48868.19
	Tb11.01.3110	heat shock protein 70 [2971037 - 2973109] MW:75366.42
122	Tb927.2.4590	branched-chain amino acid aminotransferase, putative [819424 - 818321] MW:40432.42
	Tb11.46.0001	60S acidic ribosomal subunit protein, putative [572314 - 573288] MW:34627.22
	Tb927.7.1110	asparagine synthetase a, putative [288012 - 289067] MW:39597.28
	Tb10.70.4740	enolase [745416 - 746705] MW:46592.13
	Tb927.8.4430	nucleoside phosphorylase, putative [1316456 - 1315431] MW:36924.95
	Tb927.7.4570	nucleoside hydrolase, putative [1215724 - 1214651] MW:39390.73
	Tb927.4.1300	hypothetical protein, conserved [342776 - 341652] MW:42014.86
123	Tb09.160.4590	AK arginine kinase [982261 - 983331] MW:40196.59
	Tb927.7.4570	nucleoside hydrolase, putative [1215724 - 1214651] MW:39390.73
	Tb927.7.1300	protein disulfide isomerase, putative [334736 - 335869] MW:41939.65
	Tb11.55.0024	hypothetical protein, conserved [468674 - 467679] MW:37610.93
	Tb927.4.1300	hypothetical protein, conserved [342776 - 341652] MW:42014.86

	Tb927.2.4590	branched-chain amino acid aminotransferase, putative [819424 - 818321] MW:40432.42
	Tb927.5.1460	hypothetical protein, conserved [465482 - 464502] MW:36762.16
	Tb10.70.5360	LA La protein; RNA-binding protein, putative [606492 - 607499] MW:37659.61
	Tb09.211.2360	PKAC2 protein kinase A catalytic subunit isoform 2; protein kinase A catalytic subunit [1686962 - 1
	Tb10.61.3210	hypothetical protein, conserved [3279514 - 3278519] MW:37612.28
	Tb927.3.4180	hypothetical protein [1174436 - 1173210] MW:43143.08
124	Tb927.5.1460	hypothetical protein, conserved [465482 - 464502] MW:36762.16
	Tb11.01.3040	cytosolic malate dehydrogenase, putative [2954034 - 2955020] MW:35151.21
	Tb927.8.4430	nucleoside phosphorylase, putative [1316456 - 1315431] MW:36924.95
	Tb10.6k15.1160	G-actin binding protein, putative; CAP/Srv2p, putative [2253242 - 2254051] MW:29333.07
	Tb11.01.3170	TRACK guanine nucleotide-binding protein beta subunit- like protein; activated protein kinase c rec
	Tb927.7.4290	hypothetical protein, conserved [1140765 - 1139872] MW:33703.32
	Tb09.160.3710	proliferative cell nuclear antigen (PCNA), putative [865854 - 866732] MW:32314.61
	Tb927.7.4570	nucleoside hydrolase, putative [1215724 - 1214651] MW:39390.73
	Tb927.6.2790	L-threonine 3-dehydrogenase, putative [836359 - 837357] MW:36957.64
	Tb927.3.2960	IAGNH inosine-adenosine-guanosine-nucleoside hydrolase; IAG-nucleoside hydrolase [764580 - 763597]
	Tb10.70.4740	enolase [745416 - 746705] MW:46592.13
	Tb10.6k15.3850	GAP glyceraldehyde 3-phosphate dehydrogenase, cytosolic [1726753 - 1727748] MW:35610.62
	Tb09.244.2730	60S ribosomal protein L5, putative [2307524 - 2308450] MW:34635.92
	Tb927.4.1300	hypothetical protein, conserved [342776 - 341652] MW:42014.86
	Tb11.v4.0004	RNR2 ribonucleoside-diphosphate reductase small chain [3357966 - 3356953] MW:39017.78
125	Tb11.01.3040	cytosolic malate dehydrogenase, putative [2954034 - 2955020] MW:35151.21
	Tb09.160.3710	proliferative cell nuclear antigen (PCNA), putative [865854 - 866732] MW:32314.61
	Tb927.5.1460	hypothetical protein, conserved [465482 - 464502] MW:36762.16
	Tb927.3.2960	IAGNH inosine-adenosine-guanosine-nucleoside hydrolase; IAG-nucleoside hydrolase [764580 - 763597]
	Tb11.01.3170	TRACK guanine nucleotide-binding protein beta subunit- like protein; activated protein kinase c rec
	Tb927.7.4290	hypothetical protein, conserved [1140765 - 1139872] MW:33703.32
	Tb09.244.2730	60S ribosomal protein L5, putative [2307524 - 2308450] MW:34635.92
	Tb927.6.2790	L-threonine 3-dehydrogenase, putative [836359 - 837357] MW:36957.64
	Tb10.6k15.1160	G-actin binding protein, putative; CAP/Srv2p, putative [2253242 - 2254051] MW:29333.07
	Tb10.70.2160	chaperone protein DNAJ, putative [1246723 - 1247595] MW:30863.11
126	Tb11.01.3040	cytosolic malate dehydrogenase, putative [2954034 - 2955020] MW:35151.21
	Tb927.3.2960	IAGNH inosine-adenosine-guanosine-nucleoside hydrolase; IAG-nucleoside hydrolase [764580 - 763597]

	Tb09.160.3710	proliferative cell nuclear antigen (PCNA), putative [865854 - 866732] MW:32314.61
	Tb11.01.3170	TRACK guanine nucleotide-binding protein beta subunit- like protein; activated protein kinase c rec
	Tb09.244.2730	60S ribosomal protein L5, putative [2307524 - 2308450] MW:34635.92
	Tb927.5.2890	hypothetical protein, conserved [912979 - 906488] MW:236856.89
127	Tb10.70.4740	enolase [745416 - 746705] MW:46592.13
	Tb927.8.7410	calreticulin, putative [2134955 - 2136142] MW:45021.67
	Tb927.1.2340	alpha tubulin [572442 - 573797] MW:49787.13
	Tb10.70.7730	ATP-dependent DEAD/H RNA helicase, putative; DEAD box RNA helicase, putative [139933 - 141243] MW:4
	Tb10.70.1130	hypothetical protein, conserved [1441960 - 1440659] MW:48257.68
	Tb11.01.1350	S-adenosylhomocysteine hydrolase, putative [2558252 - 2556939] MW:48447.50
	Tb10.61.0180	peptidylprolyl isomerase-like protein, putative [3887943 - 3889220] MW:47604.30
	Tb11.01.3110	heat shock protein 70 [2971037 - 2973109] MW:75366.42
	Tb10.389.1730	protein kinase, putative; tyrosine protein kinase, putative [2856535 - 2857776] MW:47389.49
	Tb10.70.0280	HSP60 chaperonin Hsp60, mitochondrial precursor [1644448 - 1646136] MW:59503.07
	Tb927.6.400	peptidase M20/M25/M40, putative [190663 - 192090] MW:52185.07
	Tb11.01.0950	hypothetical protein, conserved [2471414 - 2473114] MW:62384.20
128	Tb10.70.4740	enolase [745416 - 746705] MW:46592.13
	Tb927.8.7410	calreticulin, putative [2134955 - 2136142] MW:45021.67
	Tb927.1.2340	alpha tubulin [572442 - 573797] MW:49787.13
	Tb10.70.7730	ATP-dependent DEAD/H RNA helicase, putative; DEAD box RNA helicase, putative [139933 - 141243] MW:4
	Tb11.01.1350	S-adenosylhomocysteine hydrolase, putative [2558252 - 2556939] MW:48447.50
	Tb927.1.2330	beta tubulin [570482 - 571810] MW:49703.96
	Tb10.61.0180	peptidylprolyl isomerase-like protein, putative [3887943 - 3889220] MW:47604.30
	Tb10.70.0280	HSP60 chaperonin Hsp60, mitochondrial precursor [1644448 - 1646136] MW:59503.07
	Tb11.01.3110	heat shock protein 70 [2971037 - 2973109] MW:75366.42
	Tb927.3.5320	hypothetical protein, conserved [1496116 - 1497300] MW:43835.12
	Tb927.6.400	peptidase M20/M25/M40, putative [190663 - 192090] MW:52185.07
129	Tb10.70.4740	enolase [745416 - 746705] MW:46592.13
	Tb10.389.1730	protein kinase, putative; tyrosine protein kinase, putative [2856535 - 2857776] MW:47389.49
	Tb11.01.4660	elongation factor 1 gamma, putative [3437582 - 3436368] MW:46303.53
	Tb11.01.3110	heat shock protein 70 [2971037 - 2973109] MW:75366.42
	Tb11.01.3080	heat shock protein 70, putative [2963244 - 2965274] MW:73630.61
	Tb09.160.3270	eukaryotic initiation factor 4a, putative [813720 - 814934] MW:45361.53

	Tb927.8.1710	protein phosphatase 2A, regulatory subunit B, putative; phosphotyrosyl phosphate activator protein,
	Tb09.160.4570	AK arginine kinase [978506 - 979618] MW:41597.10
	Tb927.4.5010	calreticulin, putative [1373216 - 1372029] MW:45042.51
	Tb10.70.7730	ATP-dependent DEAD/H RNA helicase, putative; DEAD box RNA helicase, putative [139933 - 141243] MW:4
	Tb927.1.2340	alpha tubulin [572442 - 573797] MW:49787.13
	Tb09.211.2150	poly(A)-binding protein 1; PABP2 [1651052 - 1649385] MW:62147.04
130	Tb10.70.4740	enolase [745416 - 746705] MW:46592.13
	Tb11.01.3110	heat shock protein 70 [2971037 - 2973109] MW:75366.42
	Tb10.389.1730	protein kinase, putative; tyrosine protein kinase, putative [2856535 - 2857776] MW:47389.49
	Tb927.8.7410	calreticulin, putative [2134955 - 2136142] MW:45021.67
	Tb10.70.7730	ATP-dependent DEAD/H RNA helicase, putative; DEAD box RNA helicase, putative [139933 - 141243] MW:4
	Tb09.160.4570	AK arginine kinase [978506 - 979618] MW:41597.10
	Tb11.01.1350	S-adenosylhomocysteine hydrolase, putative [2558252 - 2556939] MW:48447.50
	Tb11.01.4660	elongation factor 1 gamma, putative [3437582 - 3436368] MW:46303.53
	Tb09.160.3270	eukaryotic initiation factor 4a, putative [813720 - 814934] MW:45361.53
131	Tb10.70.3710	aspartate aminotransferase [953163 - 951952] MW:44787.43
	Tb11.01.3110	heat shock protein 70 [2971037 - 2973109] MW:75366.42
	Tb10.70.4740	enolase [745416 - 746705] MW:46592.13
	Tb09.160.4570	AK arginine kinase [978506 - 979618] MW:41597.10
	Tb09.160.4590	AK arginine kinase [982261 - 983331] MW:40196.59
	Tb10.6k15.3250	succinyl-CoA ligase [GDP-forming] beta-chain, putative [1850403 - 1848883] MW:54807.20
	Tb11.01.0320	hypothetical protein, conserved [2256203 - 2257396] MW:45975.96
	Tb09.160.3270	eukaryotic initiation factor 4a, putative [813720 - 814934] MW:45361.53
	Tb927.8.1990	TRYP2 tryparedoxin peroxidase [634173 - 634853] MW:25631.46
	Tb09.211.1350	peptidyl-prolyl cis-trans isomerase (cyclophilin- 40), putative; cyclophilin-40, putative [1505691
	Tb11.02.0660	hypothetical protein, conserved [879024 - 877852] MW:43836.49
	Tb10.70.5360	LA La protein; RNA-binding protein, putative [606492 - 607499] MW:37659.61
	Tb927.4.1300	hypothetical protein, conserved [342776 - 341652] MW:42014.86
	Tb927.8.3550	mitogen-activated protein kinase 3, putative [1066185 - 1065070] MW:42672.19
	Tb927.6.4670	hypothetical protein, conserved [1303156 - 1304232] MW:40686.20
	Tb09.160.5530	hypothetical protein, conserved [1184832 - 1183792] MW:38016.33
	Tb927.7.1330	hypothetical protein, conserved [342229 - 343296] MW:39755.38
	Tb09.160.4620	IMPase inositol-1(or 4)-monophosphatase, putative [989868 - 990962] MW:39976.50
	Tb927.2.4710	RRM1 RNA-binding protein, putative [835441 - 834116] MW:49983.47

	Tb11.01.5730	ethanolamine-phosphate cytidylyltransferase, putative [3687081 - 3688235] MW:43424.77
132	Tb927.8.5600	transaldolase, putative [1663004 - 1664002] MW:36626.86
	Tb09.160.3710	proliferative cell nuclear antigen (PCNA), putative [865854 - 866732] MW:32314.61
	Tb11.01.3170	TRACK guanine nucleotide-binding protein beta subunit- like protein; activated protein kinase c rec
	Tb10.6k15.3850	GAP glyceraldehyde 3-phosphate dehydrogenase, cytosolic [1726753 - 1727748] MW:35610.62
	Tb11.01.3040	cytosolic malate dehydrogenase, putative [2954034 - 2955020] MW:35151.21
	Tb927.3.2100	hypothetical protein, conserved [553951 - 553025] MW:33996.14
	Tb09.211.0560	DRBD3 RNA-binding protein, putative; DRBD3 [1359352 - 1358369] MW:36984.69
	Tb927.6.2740	pdxK pyridoxal kinase [823608 - 824510] MW:33332.03
	Tb09.244.2730	60S ribosomal protein L5, putative [2307524 - 2308450] MW:34635.92
	Tb927.5.3830	dihydroorotate dehydrogenase, putative [1193464 - 1194405] MW:34112.29
	Tb927.3.2960	IAGNH inosine-adenosine-guanosine-nucleoside hydrolase; IAG-nucleoside hydrolase [764580 - 763597]
	Tb927.7.2640	hypothetical protein, conserved [678317 - 676956] MW:50853.10
	Tb11.02.0730	mca1 metacaspase; cysteine peptidase, Clan CD, family C13 [895761 - 894643] MW:40180.06
	Tb10.70.4930	hypothetical protein, conserved [700304 - 701188] MW:32833.06
133	Tb927.6.2740	pdxK pyridoxal kinase [823608 - 824510] MW:33332.03
	Tb927.3.3490	TDP1 high mobility group protein, putative [985621 - 986436] MW:30866.36
	Tb09.244.2730	60S ribosomal protein L5, putative [2307524 - 2308450] MW:34635.92
	Tb927.5.3830	dihydroorotate dehydrogenase, putative [1193464 - 1194405] MW:34112.29
	Tb11.01.3170	TRACK guanine nucleotide-binding protein beta subunit- like protein; activated protein kinase c rec
	Tb11.02.4250	hypothetical protein, conserved [1773128 - 1772250] MW:32851.85
	Tb09.211.0560	DRBD3 RNA-binding protein, putative; DRBD3 [1359352 - 1358369] MW:36984.69
	Tb11.01.3040	cytosolic malate dehydrogenase, putative [2954034 - 2955020] MW:35151.21
	Tb09.160.3710	proliferative cell nuclear antigen (PCNA), putative [865854 - 866732] MW:32314.61
	Tb10.70.7040	CRK1; kin1 cell division protein kinase 2 homolog 1; cdc2- like protein kinase [288378 - 287473] MW
134	Tb11.01.1290	14-3-3-like protein, putative [2547863 - 2547075] MW:30310.38
	Tb11.01.5680	hypothetical protein, conserved [3671680 - 3673122] MW:51674.18
	Tb11.02.4200	6PGL 6-phosphogluconolactonase [1758790 - 1757990] MW:28649.81
	Tb927.4.2030	hypothetical protein, conserved [511106 - 510453] MW:22703.92
	Tb09.211.0120	nascent polypeptide associated complex subunit, putative [1286359 - 1285793] MW:20129.46
	Tb11.02.4700	14-3-3-like protein, putative [1912331 - 1911573] MW:29196.24
	Tb927.8.2210	pteridine reductase, putative [677634 - 678743] MW:40289.31
	Tb09.244.2730	60S ribosomal protein L5, putative [2307524 - 2308450] MW:34635.92

	Tb927.7.3440	I/6 autoantigen [892212 - 891472] MW:27050.07
	Tb927.4.2280	hypothetical protein, conserved [593290 - 594438] MW:42285.16
	Tb927.1.2340	alpha tubulin [572442 - 573797] MW:49787.13
	Tb09.v1.0660	hypothetical protein, unlikely [1812682 - 1812804] MW:4529.59
	Tb10.70.2770	stress-inducible protein STI1-like, putative [1145116 - 1144343] MW:28424.92
135	Tb10.61.1880	protein kinase, putative; mitogen-activated protein kinase, putative [3534140 - 3533286] MW:31374.9
	Tb927.5.1460	hypothetical protein, conserved [465482 - 464502] MW:36762.16
	Tb927.4.2030	hypothetical protein, conserved [511106 - 510453] MW:22703.92
	Tb927.7.5160	deoxyuridine triphosphatase, putative; dUTP diphosphatase [1361423 - 1362286] MW:31942.99
	Tb09.244.2730	60S ribosomal protein L5, putative [2307524 - 2308450] MW:34635.92
	Tb927.3.2230	succinyl-CoA synthetase alpha subunit, putative [581836 - 580931] MW:31464.60
	Tb927.6.1080	glxII hydroxyacylglutathione hydrolase, putative; glyoxalase II [429000 - 428110] MW:32507.83
	Tb927.7.3440	I/6 autoantigen [892212 - 891472] MW:27050.07
	Tb927.6.2740	pdxK pyridoxal kinase [823608 - 824510] MW:33332.03
136	Tb927.4.2030	hypothetical protein, conserved [511106 - 510453] MW:22703.92
	Tb10.70.6610	hypothetical protein, conserved [374200 - 373463] MW:27338.99
	Tb927.4.3590	translation elongation factor 1-beta, putative [917947 - 917162] MW:28375.65
	Tb10.70.6540	HGPRT hypoxanthine-guanine phosphoribosyltransferase [390899 - 390267] MW:23371.90
	Tb10.70.3070	hypothetical protein, conserved [1100000 - 1099257] MW:28189.10
	Tb10.70.4740	enolase [745416 - 746705] MW:46592.13
	Tb11.03.0620	UBP1 RNA-binding protein, putative [119615 - 118956] MW:24295.10
	Tb10.05.0220	60S ribosomal protein L10a [3224822 - 3224178] MW:24597.00
	Tb09.v1.0660	hypothetical protein, unlikely [1812682 - 1812804] MW:4529.59
137	Tb11.02.4700	14-3-3-like protein, putative [1912331 - 1911573] MW:29196.24
	Tb11.01.1290	14-3-3-like protein, putative [2547863 - 2547075] MW:30310.38
	Tb09.160.0770	nitrilase, putative [429953 - 429132] MW:30298.74
	Tb11.01.5680	hypothetical protein, conserved [3671680 - 3673122] MW:51674.18
	Tb927.1.4830	phospholipase A1, putative [986718 - 987620] MW:32403.83
	Tb09.244.2730	60S ribosomal protein L5, putative [2307524 - 2308450] MW:34635.92
	Tb09.211.0120	nascent polypeptide associated complex subunit, putative [1286359 - 1285793] MW:20129.46
	Tb10.70.4740	enolase [745416 - 746705] MW:46592.13
	Tb927.4.2030	hypothetical protein, conserved [511106 - 510453] MW:22703.92
	Tb927.7.3440	I/6 autoantigen [892212 - 891472] MW:27050.07

	Tb11.02.2310	prostaglandin f synthase [1332644 - 1333474] MW:30992.51
	Tb11.01.7120	hypothetical protein, conserved [4055862 - 4055236] MW:23084.79
	Tb927.4.4770	hypothetical protein [1311562 - 1310579] MW:34906.10
	Tb927.6.4140	hypothetical protein, conserved [1195110 - 1194763] MW:13008.76
	Tb927.3.2220	hypothetical protein, conserved [580061 - 579399] MW:23800.14
	Tb11.01.1570	NUDIX hydrolase, conserved [2596513 - 2595773] MW:27514.54
138	Tb927.7.570	prefoldin, putative [117587 - 116994] MW:22753.76
	Tb11.01.3110	heat shock protein 70 [2971037 - 2973109] MW:75366.42
	Tb10.70.6540	HGPRT hypoxanthine-guanine phosphoribosyltransferase [390899 - 390267] MW:23371.90
	Tb10.70.1100	translation elongation factor 1-beta, putative [1449046 - 1448441] MW:21958.91
	Tb11.01.6670	X3; RAB11B small GTPase [3940046 - 3939447] MW:22782.18
	Tb927.8.5880	eukaryotic translation initiation factor 1A, putative [1732407 - 1732925] MW:19445.67
	Tb11.01.4660	elongation factor 1 gamma, putative [3437582 - 3436368] MW:46303.53
	Tb11.03.0620	UBP1 RNA-binding protein, putative [119615 - 118956] MW:24295.10
	Tb09.211.4460	ADP-ribosylation factor, putative [2120958 - 2120410] MW:20651.84
	Tb927.7.1290	hypothetical protein, conserved [333218 - 333946] MW:26998.71
139	Tb11.01.3110	heat shock protein 70 [2971037 - 2973109] MW:75366.42
	Tb927.8.1990	TRYP2 tryparedoxin peroxidase [634173 - 634853] MW:25631.46
	Tb10.26.0200	guanylate kinase, putative [2789549 - 2790085] MW:20106.00
	Tb10.70.6540	HGPRT hypoxanthine-guanine phosphoribosyltransferase [390899 - 390267] MW:23371.90
	Tb927.7.3440	I/6 autoantigen [892212 - 891472] MW:27050.07
	Tb927.6.2130	nuclear movement protein, putative; NUDC-like protein [681443 - 680916] MW:19667.41
	Tb11.01.7550	iron superoxide dismutase [4153236 - 4152640] MW:22047.76
	Tb927.1.3200	phosphatase-like protein, putative [690155 - 690820] MW:24493.00
	Tb927.2.2770	hypothetical protein, conserved [541346 - 541723] MW:13445.37
	Tb927.4.3590	translation elongation factor 1-beta, putative [917947 - 917162] MW:28375.65
	Tb11.01.6670	X3; RAB11B small GTPase [3940046 - 3939447] MW:22782.18
	Tb09.211.3210	transport protein particle (TRAPP) subunit, putative [1848013 - 1848621] MW:23009.87
	Tb11.03.0410	eIF-5A eukaryotic translation initiation factor 5a, putative [184550 - 185050] MW:17820.01
	Tb927.5.1160	hypothetical protein, conserved [395802 - 396488] MW:25569.59
	Tb11.01.6660	iron superoxide dismutase [3937799 - 3937173] MW:23280.13
	Tb09.211.4550	60S ribosomal protein L12, putative [2136698 - 2136039] MW:24017.29
	Tb11.02.5450	glucose-regulated protein 78, putative; luminal binding protein 1 (BiP), putative [2095561 - 209360
	Tb11.03.0620	UBP1 RNA-binding protein, putative [119615 - 118956] MW:24295.10

140	Tb09.211.4460	ADP-ribosylation factor, putative [2120958 - 2120410] MW:20651.84
	Tb09.211.0740	p21 antigen protein, putative [1398292 - 1397720] MW:21059.98
	Tb927.3.3450/	ADP-ribosylation factor-like protein 3A, putative [979573 - 980109] MW:19880.70
	Tb11.03.0250	CYPA cyclophilin a; cyclophilin type peptidyl-prolyl cis-trans isomerase [224513 - 225046] MW:18717
	Tb927.5.4500	ADP-ribosylation factor, putative [1326928 - 1326344] MW:21771.20
	Tb09.211.4550	60S ribosomal protein L12, putative [2136698 - 2136039] MW:24017.29
	Tb11.01.7550	iron superoxide dismutase [4153236 - 4152640] MW:22047.76
	Tb927.7.1120	GPX1 trypanothione/tryparedoxin dependent peroxidase 1, cytosolic; glutathione peroxidase-like prot
	Tb927.7.2590	prefoldin, putative [667376 - 666864] MW:18777.10
	Tb11.03.0530	hypothetical protein, conserved [140958 - 140116] MW:31292.20
	Tb927.4.2740	hypothetical protein, conserved [723054 - 723506] MW:16327.52
	Tb927.6.2200	hypothetical protein, conserved [698549 - 699142] MW:21125.70
	Tb09.160.3270	eukaryotic initiation factor 4a, putative [813720 - 814934] MW:45361.53
	Tb11.03.0410	eIF-5A eukaryotic translation initiation factor 5a, putative [184550 - 185050] MW:17820.01
141	Tb09.211.4460	ADP-ribosylation factor, putative [2120958 - 2120410] MW:20651.84
142	Tb11.02.0815	ubiquitin-conjugating enzyme, putative; ubiquitin-conjugating enzyme-like, putative [948990 - 94940
	Tb11.02.2040	hypothetical protein, conserved [1268639 - 1269016] MW:14037.89
143	Tb927.7.5790	protein disulfide isomerase, putative [1562540 - 1562947] MW:15400.76
144	Tb927.7.5790	protein disulfide isomerase, putative [1562540 - 1562947] MW:15400.76
	Tb10.26.0680	hypothetical protein, conserved [2690850 - 2691230] MW:14328.23
	Tb11.02.2030	hypothetical protein, conserved [1267722 - 1268075] MW:12757.37
145	Tb11.01.1680	polyubiquitin, putative [2621515 - 2623572] MW:76603.14
146	Tb927.7.5790	protein disulfide isomerase, putative [1562540 - 1562947] MW:15400.76
	Tb11.02.2030	hypothetical protein, conserved [1267722 - 1268075] MW:12757.37
	Tb11.01.5350	profilin [3613516 - 3613968] MW:16122.17
147	Tb927.5.1360	hypothetical protein, conserved [441346 - 441807] MW:17297.74
	Tb10.6k15.2050	RPS12 40S ribosomal protein S12, putative [2070976 - 2071404] MW:16061.40
	Tb11.02.0210	hypothetical protein, conserved [747704 - 746319] MW:50887.21

	Tb11.02.0815	ubiquitin-conjugating enzyme, putative; ubiquitin-conjugating enzyme-like, putative [948990 - 94940
	Tb927.6.4440	hypothetical protein, conserved [1248097 - 1247039] MW:37718.41
	Tb927.8.1440	maoC-like dehydratase, putative [469241 - 469729] MW:17815.52
	Tb11.01.5170	hypothetical protein, conserved [3567908 - 3567516] MW:14737.54
148	Tb11.03.0410	eIF-5A eukaryotic translation initiation factor 5a, putative [184550 - 185050] MW:17820.01
	Tb927.8.890	small GTP-binding protein Rab1, putative [266738 - 266112] MW:22714.65
	Tb09.211.4460	ADP-ribosylation factor, putative [2120958 - 2120410] MW:20651.84
	Tb927.3.3450	ADP-ribosylation factor-like protein 3A, putative [979573 - 980109] MW:19880.70
	Tb11.01.7550	iron superoxide dismutase [4153236 - 4152640] MW:22047.76
	Tb09.211.1690	hypothetical protein, conserved [1558872 - 1558366] MW:18768.80
149	Tb11.02.4700	14-3-3-like protein, putative [1912331 - 1911573] MW:29196.24
	Tb11.01.1290	14-3-3-like protein, putative [2547863 - 2547075] MW:30310.38
	Tb927.1.4830	phospholipase A1, putative [986718 - 987620] MW:32403.83
	Tb09.v1.0380	spermidine synthase, putative [1225692 - 1224796] MW:32926.41
	Tb09.211.0120	nascent polypeptide associated complex subunit, putative [1286359 - 1285793] MW:20129.46
	Tb11.01.5680	hypothetical protein, conserved [3671680 - 3673122] MW:51674.18
150	Tb09.v1.0380	spermidine synthase, putative [1225692 - 1224796] MW:32926.41
	Tb11.02.2310	prostaglandin f synthase [1332644 - 1333474] MW:30992.51
	Tb11.02.4700	14-3-3-like protein, putative [1912331 - 1911573] MW:29196.24
	Tb927.3.2100	hypothetical protein, conserved [553951 - 553025] MW:33996.14
	Tb11.01.0700	ribose 5-phosphate isomerase, putative [2408554 - 2409021] MW:16957.30
	Tb09.244.2730	60S ribosomal protein L5, putative [2307524 - 2308450] MW:34635.92
	Tb11.01.1290	14-3-3-like protein, putative [2547863 - 2547075] MW:30310.38
	Tb927.7.3440	I/6 autoantigen [892212 - 891472] MW:27050.07
	Tb927.7.2640	hypothetical protein, conserved [678317 - 676956] MW:50853.10
	Tb927.7.2260	hypothetical protein, conserved [585228 - 584389] MW:30058.81
	Tb10.389.0880	heat shock protein, putative [3016541 - 3014106] MW:90863.73
	Tb10.70.0280	HSP60 chaperonin Hsp60, mitochondrial precursor [1644448 - 1646136] MW:59503.07
	Tb927.7.7140	hypothetical protein, conserved [2045985 - 2045035] MW:35064.14
151	Tb927.5.1460	hypothetical protein, conserved [465482 - 464502] MW:36762.16
	Tb10.70.1490	hypothetical protein, conserved [1384855 - 1385826] MW:36137.95
	Tb927.8.3530	glycerol-3-phosphate dehydrogenase [NAD+], glycosomal [1058561 - 1057497] MW:37805.01

	Tb927.1.2340	alpha tubulin [572442 - 573797] MW:49787.13
152	Tb09.160.4590	AK arginine kinase [982261 - 983331] MW:40196.59
	Tb09.160.5530	hypothetical protein, conserved [1184832 - 1183792] MW:38016.33
	Tb927.7.1300	protein disulfide isomerase, putative [334736 - 335869] MW:41939.65
	Tb10.70.3710	aspartate aminotransferase [953163 - 951952] MW:44787.43
	Tb10.70.5360	LA La protein; RNA-binding protein, putative [606492 - 607499] MW:37659.61
	Tb09.160.4620	IMPase inositol-1(or 4)-monophosphatase, putative [989868 - 990962] MW:39976.50
	Tb09.160.1950	acidocalcisomal exopolyphosphatase, putative [642371 - 643522] MW:42865.13
	Tb11.55.0024	hypothetical protein, conserved [468674 - 467679] MW:37610.93
	Tb927.4.1300	hypothetical protein, conserved [342776 - 341652] MW:42014.86
	Tb10.61.3210	hypothetical protein, conserved [3279514 - 3278519] MW:37612.28
	Tb927.7.1330	hypothetical protein, conserved [342229 - 343296] MW:39755.38
	Tb10.61.0540	hypothetical protein, conserved [3824087 - 3825034] MW:36411.55
	Tb927.8.740	nucleolar RNA-binding protein, truncated [206538 - 206119] MW:13803.88
	Tb09.211.1350	peptidyl-prolyl cis-trans isomerase (cyclophilin- 40), putative; cyclophilin-40, putative [1505691
	Tb927.4.4240	hypothetical protein, conserved [1126471 - 1125479] MW:36183.86
	Tb10.389.0570	hypothetical protein, conserved; predicted zinc finger protein [3074891 - 3073917] MW:34477.06
	Tb11.02.1510	hypothetical protein, conserved [1152288 - 1155722] MW:127425.46
153	Tb927.8.6060	2-amino-3-ketobutyrate coenzyme A ligase, putative; glycine acetyltransferase, putative [1766446 -
	Tb10.70.3710	aspartate aminotransferase [953163 - 951952] MW:44787.43
	Tb11.01.3110	heat shock protein 70 [2971037 - 2973109] MW:75366.42
	Tb927.4.1300	hypothetical protein, conserved [342776 - 341652] MW:42014.86
	Tb927.6.1800	PP2C protein phosphatase 2C, putative [603410 - 602262] MW:41135.15
	Tb10.6k15.3970	developmentally regulated GTP-binding protein, putative [1711738 - 1712844] MW:41267.84
	Tb09.160.4590	AK arginine kinase [982261 - 983331] MW:40196.59
	Tb10.70.4740	enolase [745416 - 746705] MW:46592.13
	Tb10.70.1130	hypothetical protein, conserved [1441960 - 1440659] MW:48257.68
	Tb927.7.1300	protein disulfide isomerase, putative [334736 - 335869] MW:41939.65
	Tb927.6.1990	hypothetical protein, conserved [651077 - 650103] MW:37832.43
	Tb927.6.4670	hypothetical protein, conserved [1303156 - 1304232] MW:40686.20
	Tb927.3.2960	IAGNH inosine-adenosine-guanosine-nucleoside hydrolase; IAG-nucleoside hydrolase [764580 - 763597]
	Tb09.211.1350	peptidyl-prolyl cis-trans isomerase (cyclophilin- 40), putative; cyclophilin-40, putative [1505691
	Tb10.6k15.0990	selenophosphate synthetase, putative [2287044 - 2288225] MW:42984.29
	Tb927.7.1330	hypothetical protein, conserved [342229 - 343296] MW:39755.38

	Tb927.4.2450	thioredoxin, putative [636846 - 638081] MW:44490.23
154	Tb11.02.1690	hypothetical protein, conserved [1209703 - 1207811] MW:69793.86
	Tb10.70.0280	HSP60 chaperonin Hsp60, mitochondrial precursor [1644448 - 1646136] MW:59503.07
	Tb10.6k15.2330	TCP-1-theta t-complex protein 1, theta subunit, putative; CCT-theta, putative [2017097 - 2015484] M
	Tb927.6.2740	pdxK pyridoxal kinase [823608 - 824510] MW:33332.03
155	Tb927.6.4840	S-adenosylmethionine synthetase, putative [1344594 - 1345787] MW:43540.13
	Tb10.70.4740	enolase [745416 - 746705] MW:46592.13
	Tb09.160.3270	eukaryotic initiation factor 4a, putative [813720 - 814934] MW:45361.53
	Tb11.52.0013	hypothetical protein, conserved [3407660 - 3406476] MW:43332.89
	Tb10.70.1130	hypothetical protein, conserved [1441960 - 1440659] MW:48257.68
	Tb927.5.1460	hypothetical protein, conserved [465482 - 464502] MW:36762.16
	Tb11.01.5730	ethanolamine-phosphate cytidylyltransferase, putative [3687081 - 3688235] MW:43424.77
	Tb10.70.3710	aspartate aminotransferase [953163 - 951952] MW:44787.43
	Tb11.01.3080	heat shock protein 70, putative [2963244 - 2965274] MW:73630.61
	Tb09.160.4570	AK arginine kinase [978506 - 979618] MW:41597.10
156	Tb09.160.5530	hypothetical protein, conserved [1184832 - 1183792] MW:38016.33
	Tb11.02.1690	hypothetical protein, conserved [1209703 - 1207811] MW:69793.86
	Tb10.70.3070	hypothetical protein, conserved [1100000 - 1099257] MW:28189.10
157	Tb10.70.4740	enolase [745416 - 746705] MW:46592.13
	Tb09.160.3270	eukaryotic initiation factor 4a, putative [813720 - 814934] MW:45361.53
	Tb11.01.3110	heat shock protein 70 [2971037 - 2973109] MW:75366.42
	Tb11.01.4660	elongation factor 1 gamma, putative [3437582 - 3436368] MW:46303.53
	Tb10.61.1870	aminopeptidase, putative; metallo-peptidase, Clan MG, Family M24 [3536046 - 3534898] MW:42690.00
	Tb927.1.2330	beta tubulin [570482 - 571810] MW:49703.96
	Tb09.160.4570	AK arginine kinase [978506 - 979618] MW:41597.10
158	Tb10.6k15.1220	IleRS isoleucyl-tRNA synthetase, putative [2236069 - 2239500] MW:130603.20
	Tb11.02.1210	LeuRS leucyl-tRNA synthetase, putative [1078518 - 1081742] MW:121824.99
	Tb11.02.5550	hypothetical protein, conserved; predicted WD40 repeat protein [2116321 - 2114432] MW:67695.84
	Tb10.389.0880	heat shock protein, putative [3016541 - 3014106] MW:90863.73
	Tb927.1.3950	alanine aminotransferase, putative [840682 - 842391] MW:63137.20
	Tb11.01.3110	heat shock protein 70 [2971037 - 2973109] MW:75366.42

	Tb927.1.2340	alpha tubulin [572442 - 573797] MW:49787.13
	Tb10.26.1080	heat shock protein 83; heat shock protein [2613113 - 2610999] MW:80763.23
	Tb11.01.7010	hypothetical protein, conserved [4017280 - 4014062] MW:117882.50
159	Tb09.211.3610	UBA2 ubiquitin-activating enzyme E1, putative [1935154 - 1938798] MW:134654.90
	Tb11.01.8770	hypothetical protein, conserved; leucine-rich repeat protein (LRRP), putative [4475937 - 4472923] M
	Tb10.26.1080	heat shock protein 83; heat shock protein [2613113 - 2610999] MW:80763.23
	Tb927.5.3510	SMC3 structural maintenance of chromosome 3, putative [1102436 - 1106035] MW:136371.02
	Tb927.1.1100	hypothetical protein, conserved [308704 - 311616] MW:108810.61
	Tb10.61.2680	PYK1 pyruvate kinase 1 [3364197 - 3363142] MW:38453.32
160	Tb10.389.0720	hypothetical protein, conserved [3045775 - 3042734] MW:110243.85
	Tb927.8.8330	calpain, putative; cysteine peptidase, putative [2466411 - 2469077] MW:98457.24
	Tb927.1.2330	beta tubulin [570482 - 571810] MW:49703.96
161	Tb10.26.1080	heat shock protein 83; heat shock protein [2613113 - 2610999] MW:80763.23
	Tb927.8.3680	hypothetical protein, conserved [1102829 - 1100892] MW:69597.10
	Tb09.211.3350	hypothetical protein, conserved [1872213 - 1874258] MW:76666.61
162	Tb11.22.0005	phenylalanyl-tRNA synthetase, putative [656609 - 658483] MW:70311.27
	Tb927.3.4290	PFR-C; PFR-D 73 kDa paraflagellar rod protein; PFR1 [1210633 - 1208864] MW:68682.76
	Tb11.01.3110	heat shock protein 70 [2971037 - 2973109] MW:75366.42
	Tb11.01.3080	heat shock protein 70, putative [2963244 - 2965274] MW:73630.61
	Tb927.8.4970	PFR 69 kDa paraflagellar rod protein; PFR2 [1474818 - 1476620] MW:69597.02
163	Tb10.70.4740	enolase [745416 - 746705] MW:46592.13
	Tb927.3.5340	Hsc70-interacting protein (Hip), putative [1499136 - 1500290] MW:42027.88
	Tb927.2.5160	chaperone protein DnaJ, putative [910842 - 912056] MW:44813.55
	Tb927.1.2340	alpha tubulin [572442 - 573797] MW:49787.13
	Tb927.1.700	PGKC; gPGK phosphoglycerate kinase [233826 - 232504] MW:47245.76
	Tb10.70.7480	ATP synthase, putative [186531 - 187937] MW:52900.99
164	Tb927.7.7420	ATP synthase alpha chain, mitochondrial precursor; ATP synthase F1, alpha subunit [2134761 - 213300
	Tb09.160.4250	TRYP1; TXNPx tryparedoxin peroxidase [936746 - 937345] MW:22424.65
	Tb927.1.2340	alpha tubulin [572442 - 573797] MW:49787.13

165	Tb11.02.4440	aminopeptidase, putative; metallo-peptidase, Clan MF, Family M17 [1830540 - 1828975] MW:55359.24
	Tb11.01.5710	phenylalanyl-tRNA synthetase alpha chain, putative [3683172 - 3684662] MW:57056.38
	Tb10.61.2680	PYK1 pyruvate kinase 1 [3364197 - 3363142] MW:38453.32
	Tb927.1.2340	alpha tubulin [572442 - 573797] MW:49787.13
	Tb927.1.3950	alanine aminotransferase, putative [840682 - 842391] MW:63137.20
	Tb09.211.4880	PPIase cyclophilin-like protein, putative [2201295 - 2200141] MW:42646.96
166	Tb11.02.4440	aminopeptidase, putative; metallo-peptidase, Clan MF, Family M17 [1830540 - 1828975] MW:55359.24
	Tb11.01.4660	elongation factor 1 gamma, putative [3437582 - 3436368] MW:46303.53
	Tb10.61.2680	PYK1 pyruvate kinase 1 [3364197 - 3363142] MW:38453.32
	Tb927.8.6280	hypothetical protein, conserved [1825049 - 1825798] MW:27122.89
167	Tb09.160.4250	TRYP1; TXNPx tryparedoxin peroxidase [936746 - 937345] MW:22424.65
	Tb10.70.3710	aspartate aminotransferase [953163 - 951952] MW:44787.43
168	Tb927.8.2050	mannose-1-phosphate guanyltransferase, putative [642645 - 643754] MW:40704.08
	Tb927.4.1300	hypothetical protein, conserved [342776 - 341652] MW:42014.86
	Tb11.02.4440	aminopeptidase, putative; metallo-peptidase, Clan MF, Family M17 [1830540 - 1828975] MW:55359.24
	Tb927.1.2340	alpha tubulin [572442 - 573797] MW:49787.13
169	Tb927.5.1460	hypothetical protein, conserved [465482 - 464502] MW:36762.16
	Tb10.70.1490	hypothetical protein, conserved [1384855 - 1385826] MW:36137.95
	Tb927.8.3530	glycerol-3-phosphate dehydrogenase [NAD+], glycosomal [1058561 - 1057497] MW:37805.01
	Tb927.1.2340	alpha tubulin [572442 - 573797] MW:49787.13
	Tb11.02.4440	aminopeptidase, putative; metallo-peptidase, Clan MF, Family M17 [1830540 - 1828975] MW:55359.24
170	Tb10.70.1370	ALD fructose-bisphosphate aldolase, glycosomal, putative [1403364 - 1404482] MW:41071.12
171	Tb10.26.1080	heat shock protein 83; heat shock protein [2613113 - 2610999] MW:80763.23
	Tb11.01.3110	heat shock protein 70 [2971037 - 2973109] MW:75366.42
	Tb10.70.2650	elongation factor 2 [1168806 - 1166266] MW:94334.21
	Tb09.211.3610	UBA2 ubiquitin-activating enzyme E1, putative [1935154 - 1938798] MW:134654.90
	Tb10.389.0880	heat shock protein, putative [3016541 - 3014106] MW:90863.73
	Tb10.389.0630	prolyl-trna synthetase, putative; bifunctional aminoacyl-trna synthetase, putative [3063227 - 30608
	Tb927.1.2340	alpha tubulin [572442 - 573797] MW:49787.13
	Tb10.6k15.3600	myo-inositol-1-phosphate synthase, putative [1769650 - 1771236] MW:58274.40

	Tb927.8.4970	PFR 69 kDa paraflagellar rod protein; PFR2 [1474818 - 1476620] MW:69597.02
	Tb11.02.5550	hypothetical protein, conserved; predicted WD40 repeat protein [2116321 - 2114432] MW:67695.84
	Tb11.02.0300	hypothetical protein, conserved [775298 - 773409] MW:69229.86
	Tb11.02.5450	glucose-regulated protein 78, putative; luminal binding protein 1 (BiP), putative [2095561 - 209360
172	Tb10.26.1080	heat shock protein 83; heat shock protein [2613113 - 2610999] MW:80763.23
	Tb927.5.2940	stress-induced protein sti1, putative [927151 - 928803] MW:62327.63
	Tb10.61.1750	TBKIFC1 C-terminal motor kinesin, putative [3561647 - 3564109] MW:90800.49
	Tb927.8.4970	PFR 69 kDa paraflagellar rod protein; PFR2 [1474818 - 1476620] MW:69597.02
	Tb11.01.3110	heat shock protein 70 [2971037 - 2973109] MW:75366.42
	Tb09.211.3610	UBA2 ubiquitin-activating enzyme E1, putative [1935154 - 1938798] MW:134654.90
	Tb927.3.4290	PFR-C; PFR-D 73 kDa paraflagellar rod protein; PFR1 [1210633 - 1208864] MW:68682.76
	Tb927.1.2340	alpha tubulin [572442 - 573797] MW:49787.13
	Tb10.389.0880	heat shock protein, putative [3016541 - 3014106] MW:90863.73
	Tb10.70.2650	elongation factor 2 [1168806 - 1166266] MW:94334.21
	Tb11.02.1120	adenylosuccinate synthetase, putative [1055003 - 1056811] MW:66674.89
	Tb927.1.2330	beta tubulin [570482 - 571810] MW:49703.96
	Tb927.4.3740	hypothetical protein, conserved [945122 - 940044] MW:192569.03
	Tb09.160.3590	PDE2C cAMP-specific phosphodiesterase [852556 - 855348] MW:103651.08
173	Tb10.389.0720	hypothetical protein, conserved [3045775 - 3042734] MW:110243.85
	Tb10.61.1750	TBKIFC1 C-terminal motor kinesin, putative [3561647 - 3564109] MW:90800.49
	Tb927.3.4290	PFR-C; PFR-D 73 kDa paraflagellar rod protein; PFR1 [1210633 - 1208864] MW:68682.76
	Tb10.26.1080	heat shock protein 83; heat shock protein [2613113 - 2610999] MW:80763.23
	Tb927.1.2340	alpha tubulin [572442 - 573797] MW:49787.13
	Tb927.1.2330	beta tubulin [570482 - 571810] MW:49703.96
	Tb11.02.4830	protein kinase, putative; serine/threonine protein kinase, putative [1945257 - 1943431] MW:67118.12
	Tb927.4.3740	hypothetical protein, conserved [945122 - 940044] MW:192569.03
174		

Table S2 / BN-PAGE gel

SHEET 2 BN class -Non-redundant list of proteins

Accession number	protein name	MapMan bins	Matching peptides	Highest peptide score
Tb927.6.2790	L-threonine 3-dehydrogenase, putative [836359 - 837357] MW:36957.64	13.2 amino acid metabolism.degradation	3	53
Tb09.160.4570	AK arginine kinase [978506 - 979618] MW:41597.10	13.1.1 amino acid metabolism	3	54
Tb09.160.4590	AK arginine kinase [982261 - 983331] MW:40196.59	13.1.1 amino acid metabolism	20	82
Tb927.7.1110	asparagine synthetase a, putative [288012 - 289067] MW:39597.28	13.1.1 amino acid metabolism	4	53
Tb10.70.3710	aspartate aminotransferase [953163 - 951952] MW:44787.43	13.1.1.2 amino acid metabolism	1	90
Tb927.2.4590	branched-chain amino acid aminotransferase, putative [819424 - 818321] MW:40432.42	13.1.1.2 amino acid metabolism	6	77
Tb927.8.6060	2-amino-3-ketobutyrate coenzyme A ligase, putative; MW : 44049	13.1.1.2 amino acid metabolism	12	76
Tb927.1.3950	alanine aminotransferase, putative [840682 - 842391] MW:63137.20	13.1.1.3 amino acid metabolism	5	102
Tb927.6.4840	S-adenosylmethionine synthetase, putative [1344594 - 1345787] MW:43540.13	13.1.3.4 amino acid metabolism.	11	89
Tb10.6k15.0990	selenophosphate synthetase, putative [2287044 - 2288225] MW:42984.29	13.1.5 amino acid metabolism.synthesis.serine	1	40
Tb09.160.1950	acidocalcisomal exopolyphosphatase, putative [642371 - 643522] MW:42865.13	13.1.5.1.3 amino acid metabolism.	1	59
Tb11.01.1350	S-adenosylhomocysteine hydrolase, putative [2558252 - 2556939] MW:48447.50	13.2.3.4 amino acid metabolism.	8	77
Tb927.7.7040	methylthioadenosine phosphorylase, putative [2013549 - 2012620] MW:33443.37	13.1.1 amino acid metabolism	5	59
Tb927.6.1080	hydroxyacylglutathione hydrolase, putative; glyoxalase II [429000 - 428110] MW:32507.83	24.1 Biodegradation of Xenobiotics.	1	44
Tb10.406.0520	trypanothione reductase [2521506 - 2520028] MW:53156.79	21.2 redox.ascorbate and glutathione	16	102
Tb927.2.4370	TRYS; TrS trypanothione synthetase, putative [772890 - 771007] MW:71612.91	21.2.2 redox.ascorbate and glutathione.	24	84
Tb09.160.4250	TRYP1; TXNPx tryparedoxin peroxidase [936746 - 937345] MW:22424.65	21.5 redox.peroxiredoxins	6	81
Tb927.4.2450	thioredoxin, putative [636846 - 638081] MW:44490.23	21.5 redox.peroxiredoxins	1	66
Tb927.7.1120	GPX1 trypanothione/tryparedoxin dependent peroxidase 1, cytosolic; MW:18747	21.5 redox.peroxiredoxins	1	52
Tb927.8.1990	TRYP2 tryparedoxin peroxidase [634173 - 634853] MW:25631.46	21.5 redox.peroxiredoxins	1	124
Tb11.01.6660	iron superoxide dismutase [3937799 - 3937173] MW:23280.13	21.6 redox.dismutases and catalases	1	39
Tb11.01.7550	iron superoxide dismutase [4153236 - 4152640] MW:22047.76	21.6 redox.dismutases and catalases	1	87
Tb09.211.4460	ADP-ribosylation factor, putative [2120958 - 2120410] MW:20651.84	23 nucleotide metabolism	6	81
Tb11.01.3170	TRACK guanine nucleotide-binding protein beta subunit- like protein; MW : 35181	23 nucleotide metabolism	7	64
Tb11.01.7800	NDPK nucleoside diphosphate kinase [4213191 - 4212730] MW:16857.24	23 nucleotide metabolism	4	61
Tb11.v4.0004	ribonucleoside-diphosphate reductase small chain [3357966 - 3356953] MW:39017.78	23 nucleotide metabolism	1	50
Tb927.3.2960	IAGNH inosine-adenosine-guanosine-nucleoside hydrolase; MW :36509	23 nucleotide metabolism	8	67
Tb927.3.3450	ADP-ribosylation factor-like protein 3A, putative [979573 - 980109] MW:19880.70	23 nucleotide metabolism	2	64
Tb927.5.3820	aspartate carbamoyltransferase, putative [1191671 - 1192654] MW:35910.64	23 nucleotide metabolism	9	95
Tb927.5.4500	ADP-ribosylation factor, putative [1326928 - 1326344] MW:21771.20	23 nucleotide metabolism	2	63

Tb927.7.4570	nucleoside hydrolase, putative [1215724 - 1214651] MW:39390.73	23 nucleotide metabolism	9	68
Tb927.7.5160	deoxyuridine triphosphatase, putative; [1361423 - 1362286] MW:31942.99	23 nucleotide metabolism	2	48
Tb927.8.4430	nucleoside phosphorylase, putative [1316456 - 1315431] MW:36924.95	23 nucleotide metabolism	5	66
Tb09.160.5560	adenylosuccinate lyase, putative [1193479 - 1192064] MW:53119.86	23.1.2 nucleotide metabolism.	2	36
Tb10.26.0200	guanylate kinase, putative [2789549 - 2790085] MW:20106.00	23.1.2 nucleotide metabolism.	4	88
Tb10.70.6540	hypoxanthine-guanine phosphoribosyltransferase [390899 - 390267] MW:23371.90	23.1.2 nucleotide metabolism.	3	79
Tb10.70.7330	adenylate kinase, putative [214354 - 215136] MW:29764.86	23.1.2 nucleotide metabolism.	1	44
Tb11.02.1120	adenylosuccinate synthetase, putative [1055003 - 1056811] MW:66674.89	23.1.2 nucleotide metabolism.	8	111
Tb927.7.2100	GMP synthase, putative; glutamine amidotransferase, putative;MW:71794.49	23.1.2.31 nucleotide metabolism.	7	97
Tb09.160.4620	IMPase inositol-1(or 4)-monophosphatase, putative [989868 - 990962] MW:39976.50	23.2 nucleotide metabolism.degradation	2	43
Tb927.5.3830	dihydroorotate dehydrogenase, putative [1193464 - 1194405] MW:34112.29	23.2 nucleotide metabolism.degradation	3	92
Tb11.01.1570	NUDIX hydrolase, conserved [2596513 - 2595773] MW:27514.54	23.5 nucleotide metabolism.	1	50
Tb09.211.2150	poly(A)-binding protein 1; PABP2 [1651052 - 1649385] MW:62147.04	27.3 RNA.regulation of transcription	3	62
Tb09.211.0560	DRBD3 RNA-binding protein, putative; DRBD3 [1359352 - 1358369] MW:36984.69	27.4 RNA.RNA binding	3	57
Tb09.211.4540	RNA-binding protein, putative; DRBD2 [2134994 - 2134077] MW:34618.44	27.4 RNA.RNA binding	1	36
Tb10.70.5360	LA La protein; RNA-binding protein, putative [606492 - 607499] MW:37659.61	27.4 RNA.RNA binding	3	78
Tb11.03.0620	UBP1 RNA-binding protein, putative [119615 - 118956] MW:24295.10	27.4 RNA.RNA binding	1	39
Tb927.2.4710	RRM1 RNA-binding protein, putative [835441 - 834116] MW:49983.47	27.4 RNA.RNA binding	1	36
Tb927.8.740	nucleolar RNA-binding protein, truncated [206538 - 206119] MW:13803.88	27.4 RNA.RNA binding	1	48
Tb10.70.3290	ATP-dependent DEAD-box RNA helicase, putative; DHH1 [1056598 - 1055378] MW:46468.25	27.5* RNA.DEAD/DEAH BOX helicase	10	87
Tb10.70.7730	ATP-dependent DEAD/H RNA helicase, putative; MW:49651	27.5* RNA.DEAD/DEAH BOX helicase	5	88
Tb927.4.1270	RuvB-like DNA helicase, putative [337939 - 336560] MW:49905.13	27.5* RNA.DEAD/DEAH BOX helicase	3	63
Tb927.5.1700	replication Factor A 28 kDa subunit, putative [537824 - 538591] MW:27592.91	28.1 DNA.synthesis/chromatin structure	2	69
Tb927.5.3510	structural maintenance of chromosome 3, putative [1102436 - 1106035] MW:136371.02	28.1 DNA.synthesis/chromatin structure	1	30
Tb11.01.0870	replication factor A, 51kDa subunit, putative [2447406 - 2448797] MW:52331.41	28.1 DNA.synthesis/chromatin structure	5	79
Tb10.406.0330	histone H2B, putative [2541174 - 2540836] MW:12569.59	28.1.3 DNA.synthesis/chromatin structure.	1	64
Tb927.3.3490	high mobility group protein, putative for tyrosyl-DNA phosphodiesterase 1;MW:30866.36	28.2 DNA.repair	4	75
Tb09.160.3730	glutaminyl-tRNA synthetase, putative [867532 - 869415] MW:73330.45	29.1 protein.aa activation	5	63
Tb10.389.0630	prolyl-trna synthetase, putative; MW:92329	29.1 protein.aa activation	2	61
Tb10.6k15.1220	IleRS isoleucyl-tRNA synthetase, putative [2236069 - 2239500] MW:130603.20	29.1 protein.aa activation	14	96
Tb10.70.6470	MetRS methionyl-tRNA synthetase, putative [403376 - 401055] MW:86910.83	29.1 protein.aa activation	4	68
Tb11.01.5710	phenylalanyl-tRNA synthetase alpha chain, putative [3683172 - 3684662] MW:57056.38	29.1 protein.aa activation	10	78
Tb11.02.5020	seryl-tRNA synthetase, putative [1983474 - 1982035] MW:53857.17	29.1 protein.aa activation	2	39
Tb11.22.0005	phenylalanyl-tRNA synthetase, putative [656609 - 658483] MW:70311.27	29.1 protein.aa activation	11	88
Tb11.46.0008	arginyl-tRNA synthetase, putative [558094 - 560172] MW:78432.50	29.1 protein.aa activation	4	69
Tb927.5.1090	threonyl-tRNA synthetase, putative [352618 - 355017] MW:90983.63	29.1 protein.aa activation	2	38

Tb927.6.4480	ValRS valyl-tRNA synthetase, putative [1260182 - 1257255] MW:110922.46	29.1 protein.aa activation	2	61
Tb927.6.4590	glutamyl-tRNA synthetase, putative [1285581 - 1287404] MW:68931.84	29.1 protein.aa activation	3	102
Tb927.6.700	alanyl-tRNA synthetase, putative [296642 - 293742] MW:105913.11	29.1 protein.aa activation	2	44
Tb927.7.3620	tyrosyl-tRNA synthetase, putative [942837 - 940744] MW:76657.88	29.1 protein.aa activation	1	36
Tb927.8.1600	lysyl-tRNA synthetase, putative [531411 - 533165] MW:66786.31	29.1 protein.aa activation	3	71
Tb09.160.3270	eukaryotic initiation factor 4a, putative [813720 - 814934] MW:45361.53	29.2 protein.synthesis	8	86
Tb10.6k15.2220	eukaryotic translation initiation factor 3 subunit 8, [2033435 - 2031213] MW:84339.71	29.2 protein.synthesis	1	45
Tb11.01.4830	eIF-2-gamma eukaryotic translation initiation factor 2 gamma, putative ; MW:52200	29.2 protein.synthesis	1	35
Tb11.03.0410	eIF-5A eukaryotic translation initiation factor 5a, putative [184550 - 185050] MW:17820.01	29.2 protein.synthesis	4	105
Tb927.5.2570	translation initiation factor, putative [825553 - 827643] MW:79811.15	29.2 protein.synthesis	2	42
Tb927.8.5880	eukaryotic translation initiation factor 1A, putative [1732407 - 1732925] MW:19445.67	29.2 protein.synthesis	1	58
Tb09.160.2550	ribosomal protein S7, putative [720303 - 720911] MW:23841.01	29.2.4 protein.synthesis.elongation	2	48
Tb09.160.3590	PDE2C cAMP-specific phosphodiesterase [852556 - 855348] MW:103651.08	29.2.4 protein.synthesis.elongation	2	58
Tb09.160.4200	60S acidic ribosomal protein, putative [925061 - 925402] MW:11090.51	29.2.4 protein.synthesis.elongation	1	52
Tb09.160.4450	RPS3 40S ribosomal protein S3, putative [957257 - 958057] MW:30401.37	29.2.4 protein.synthesis.elongation	2	85
Tb09.211.0110	QM 60S ribosomal protein L10, putative; QM-like protein; MW:24736.07	29.2.4 protein.synthesis.elongation	2	84
Tb09.211.2630	60S ribosomal protein L23, putative [1737671 - 1738090] MW:14962.60	29.2.4 protein.synthesis.elongation	2	72
Tb09.211.4550	60S ribosomal protein L12, putative [2136698 - 2136039] MW:24017.29	29.2.4 protein.synthesis.elongation	1	59
Tb09.211.4850	60S ribosomal protein L26, putative [2196000 - 2195569] MW:16454.32	29.2.4 protein.synthesis.elongation	1	47
Tb09.244.2730	60S ribosomal protein L5, putative [2307524 - 2308450] MW:34635.92	29.2.4 protein.synthesis.elongation	2	57
Tb10.05.0220	60S ribosomal protein L10a [3224822 - 3224178] MW:24597.00	29.2.4 protein.synthesis.elongation	2	60
Tb10.26.0560	60S ribosomal protein L6, putative [2707740 - 2708318] MW:21184.84	29.2.4 protein.synthesis.elongation	1	26
Tb10.389.0910	60S ribosomal protein L34, putative [3009371 - 3008859] MW:19366.04	29.2.4 protein.synthesis.elongation	1	55
Tb10.61.1960	RPS2 40S ribosomal protein S2, putative [3521328 - 3520528] MW:28641.95	29.2.4 protein.synthesis.elongation	1	60
Tb10.61.2090	60S ribosomal protein L17, putative [3502175 - 3501675] MW:19105.48	29.2.4 protein.synthesis.elongation	1	36
Tb10.6k15.2050	RPS12 40S ribosomal protein S12, putative [2070976 - 2071404] MW:16061.40	29.2.4 protein.synthesis.elongation	2	47
Tb10.70.1100	translation elongation factor 1-beta, putative [1449046 - 1448441] MW:21958.91	29.2.4 protein.synthesis.elongation	6	83
Tb10.70.1380	40S ribosomal protein S9, putative [1402480 - 1403052] MW:22051.52	29.2.4 protein.synthesis.elongation	1	55
Tb10.70.2650	elongation factor 2 [1168806 - 1166266] MW:94334.21	29.2.4 protein.synthesis.elongation	26	106
Tb10.70.3360	40S ribosomal protein S3a, putative [1044038 - 1043268] MW:29422.06	29.2.4 protein.synthesis.elongation	1	36
Tb10.70.3510	60S ribosomal protein L18a, putative [1011318 - 1010779] MW:20904.27	29.2.4 protein.synthesis.elongation	1	85
Tb10.70.5650	TEF1 elongation factor 1-alpha [548535 - 549884] MW:49105.63	29.2.4 protein.synthesis.elongation	3	86
Tb10.70.7010	60S ribosomal protein L9, putative [291415 - 290846] MW:21857.31	29.2.4 protein.synthesis.elongation	1	58
Tb10.70.7695	40S ribosomal proteins S11, putative [146230 - 146754] MW:20087.25	29.2.4 protein.synthesis.elongation	3	49
Tb11.01.2560	40S ribosomal protein SA, putative [2829298 - 2830032] MW:27609.60	29.2.4 protein.synthesis.elongation	2	63
Tb11.01.3020	40S ribosomal protein L14, putative [2952037 - 2952606] MW:21469.27	29.2.4 protein.synthesis.elongation	1	65

Tb11.01.4660	elongation factor 1 gamma, putative [3437582 - 3436368] MW:46303.53	29.2.4 protein.synthesis.elongation	8	75
Tb11.01.7960	60S ribosomal protein L2, putative; 60S ribosomal protein L8, putative ;MW:28313	29.2.4 protein.synthesis.elongation	1	42
Tb11.02.1085	40s ribosomal protein S4, putative [1041968 - 1042789] MW:30643.93	29.2.4 protein.synthesis.elongation	3	63
Tb11.46.0001	60S acidic ribosomal subunit protein, putative [572314 - 573288] MW:34627.22	29.2.4 protein.synthesis.elongation	4	102
Tb927.3.3310	60S ribosomal protein L13, putative [851587 - 850898] MW:26639.22	29.2.4 protein.synthesis.elongation	2	48
Tb927.3.5050	60S ribosomal protein L4 [1417717 - 1418841] MW:41855.88	29.2.4 protein.synthesis.elongation	1	39
Tb927.4.3550	60S ribosomal protein L13a, putative [912419 - 911751] MW:25711.55	29.2.4 protein.synthesis.elongation	2	56
Tb927.4.3570	translation elongation factor 1-beta, putative [915890 - 915105] MW:28403.66	29.2.4 protein.synthesis.elongation	2	52
Tb927.4.3590	translation elongation factor 1-beta, putative [917947 - 917162] MW:28375.65	29.2.4 protein.synthesis.elongation	11	97
Tb927.6.4980	RPS14 40S ribosomal protein S14 [1370239 - 1370673] MW:15512.80	29.2.4 protein.synthesis.elongation	2	86
Tb927.7.5170	60S ribosomal protein L23a; 60S ribosomal protein L25;MW:18170.39	29.2.4 protein.synthesis.elongation	1	124
Tb09.211.0120	nascent polypeptide associated complex subunit, putative; MW:20129.46	29.2.4 protein.synthesis.elongation	3	65
Tb09.160.0580	Mov34/MPN/PAD-1 metallopeptidase, putative; , Family M67 MW :31496	29.5 protein.degradation	1	49
Tb09.211.4330	aminopeptidase P, putative; metallo-peptidase, Clan MG, Family M24 ; MW:54408.19	29.5 protein.degradation	2	105
Tb10.389.1480	cytosolic nonspecific dipeptidase; peptidase (M20/M25/M40 family), MW :52002	29.5 protein.degradation	10	100
Tb10.61.1870	aminopeptidase, putative; metallo-peptidase, Clan MG, Family M24 ; MW:42690.00	29.5 protein.degradation	2	42
Tb10.6k15.2520	prolyl oligopeptidase, putative; serine peptidase, family S9A, MW:78033	29.5 protein.degradation	9	84
Tb11.02.0100	carboxypeptidase, putative; metallo-peptidase, Clan MA(E) Family M32 ; MW:57683.01	29.5 protein.degradation	21	121
Tb11.02.0730	metacaspase; cysteine peptidase, Clan CD, family C13 [895761 - 894643] MW:40180.06	29.5 protein.degradation	1	53
Tb11.02.1070	aminopeptidase, ; metallo-peptidase, Family M1 [1037043 - 1039658] MW:98099.41	29.5 protein.degradation	46	106
Tb11.02.4440	aminopeptidase, putative; metallo-peptidase,, Family M17 [1830540 - 1828975] MW:55359.24	29.5 protein.degradation	11	133
Tb927.3.3410	aspartyl aminopeptidase, putative; metallo-peptidase, Family M20 MW:49809	29.5 protein.degradation	10	79
Tb927.3.4750	aminopeptidase, putative; metallo-peptidase, Clan MA(E) Family M1 ;MW:97251.35	29.5 protein.degradation	3	63
Tb927.6.400	peptidase M20/M25/M40, putative [190663 - 192090] MW:52185.07	29.5 protein.degradation	7	124
Tb927.7.190	OPA thimet oligopeptidase A, putative; Clan MA(E) Family M3, MW:77937	29.5 protein.degradation	2	62
Tb927.8.7020	peptidase, putative; metallo-peptidase, Clan ME, Family M16 ;MW:118860.34	29.5 protein.degradation	1	50
Tb927.8.8330	calpain, putative; cysteine peptidase, putative [2466411 - 2469077] MW:98457.24	29.5.3 protein.degradation	1	57
Tb09.160.5550	calpain-like cysteine peptidase, putative; Clan CA, family C2, MW:96528	29.5.3 protein.degradation.	1	74
Tb927.3.4840	ubiquitin hydrolase, putative; cysteine peptidase, family C19, MW:84094	29.5.3 protein.degradation.	2	60
Tb927.4.3790	ubiquitin carboxyl-terminal hydrolase, putative; Clan CA, family C19, MW :52426	29.5.3 protein.degradation.	2	85
Tb11.01.1680	polyubiquitin, putative [2621515 - 2623572] MW:76603.14	29.5.11 protein.degradation.ubiquitin	3	56
Tb927.8.2640	UBA1 ubiquitin-activating enzyme E1, putative [779218 - 782385] MW:116406.51	29.5.11 protein.degradation.ubiquitin	2	76
Tb09.211.3610	UBA2 ubiquitin-activating enzyme E1, putative [1935154 - 1938798] MW:134654.90	29.5.11 protein.degradation.ubiquitin	8	59
Tb927.5.1000	ubiquitin-conjugating enzyme E2, putative; [328251 - 328697] MW:16766.07	29.5.11.20 protein.degradation.ubiquitin.	1	26
Tb11.02.0815	ubiquitin-conjugating enzyme, putative; MW:15815	29.5.11 protein.degradation.ubiquitin	2	70
Tb09.211.1250	TbPSA6 proteasome alpha 1 subunit, putative; MW: 27925	29.5.11.20 protein.degradation.ubiquitin.	3	53

Tb10.100.0170	proteasome alpha 2 subunit, putative [84003 - 84698] MW:25354.60	29.5.11.20 protein.degradation.ubiquitin.	7	100
Tb927.7.4420	proteasome alpha 3 subunit, putative [1176212 - 1175352] MW:32154.10	29.5.11.20 protein.degradation.ubiquitin.	3	107
Tb11.02.4870	PSA4 proteasome alpha 4 subunit, putative [1954053 - 1953310] MW:27865.39	29.5.11.20 protein.degradation.ubiquitin.	11	109
Tb10.100.0120	proteasome alpha 5 subunit, putative; MW:27176.51	29.5.11.20 protein.degradation.ubiquitin.	8	128
Tb10.70.0850	TbPSA6 proteasome alpha 6 subunit, putative; MW:29449	29.5.11.20 protein.degradation.ubiquitin.	8	93
Tb927.3.780	TbPSA7 proteasome alpha 7 subunit [187958 - 187245] MW:25462.76	29.5.11.20 protein.degradation.ubiquitin.	4	65
Tb927.6.1260	TbPSB1 proteasome beta-1 subunit, putative [470586 - 469738] MW:30441.39	29.5.11.20 protein.degradation.ubiquitin.	2	74
Tb09.211.2590	proteasome beta 2 subunit, putative; MW:27402.47	29.5.11.20 protein.degradation.ubiquitin.	7	75
Tb11.02.5170	PSB3 proteasome beta 3 subunit, putative [2029089 - 2029706] MW:22483.92	29.5.11.20 protein.degradation.ubiquitin.	4	98
Tb10.70.2490	PSB4 proteasome beta 4 subunit, putative; MW:22776.06	29.5.11.20 protein.degradation.ubiquitin.	3	67
Tb10.70.0790	PRCE proteasome beta 5 subunit, putative; MW:34415.80	29.5.11.20 protein.degradation.ubiquitin.	4	68
Tb927.7.4790	BETA6 proteasome beta 6 subunit; 20S proteasome beta 6 subunit, putative;	29.5.11.20 protein.degradation.ubiquitin.	5	72
Tb927.4.430	proteasome beta 7 subunit [128728 - 128072] MW:24408.88	29.5.11.20 protein.degradation.ubiquitin.	3	52
Tb10.70.3660	proteasome activator protein PA26 [965880 - 965185] MW:25257.72	29.5.11.20 protein.degradation.ubiquitin.	9	85
Tb10.70.6360	RPN5 proteasome regulatory non-ATP-ase subunit 5; MW:55375	29.5.11.20 protein.degradation.ubiquitin.	2	56
Tb11.01.6030	proteasome regulatory non-ATP-ase subunit [3777343 - 3778167] MW:31280.91	29.5.11.20 protein.degradation.ubiquitin.	1	76
Tb927.2.2440	RPN6 proteasome regulatory non-ATPase subunit 6 [482150 - 480609] MW:57323.81	29.5.11.20 protein.degradation.ubiquitin.	1	48
Tb927.3.5520	RPN1 26S proteasome regulatory non-ATPase subunit ; MW:99898.08	29.5.11.20 protein.degradation.ubiquitin.	1	24
Tb927.3.5340	Hsc70-interacting protein (Hip), putative [1499136 - 1500290] MW:42027.88	29.5.11.4.2 protein.degradation.ubiquitin.	2	46
Tb09.211.1350	peptidyl-prolyl cis-trans isomerase (cyclophilin- 40), putative; MW:38584	29.6 protein.(un)folding	1	72
Tb09.211.4880	PPIase cyclophilin-like protein, putative [2201295 - 2200141] MW:42646.96	29.6 protein.(un)folding	1	35
Tb927.7.4770	PPIase cyclophilin-type peptidyl-prolyl cis-trans isomerase,MW:18589	29.6 protein.(un)folding	3	56
Tb11.03.0250	CYPA cyclophilin a; MW:18717	29.6 protein.(un)folding	6	103
Tb10.61.0180	peptidylprolyl isomerase-like protein, putative [3887943 - 3889220] MW:47604.30	29.6 protein.(un)folding	4	100
Tb10.6k15.2290	BS2 protein disulfide isomerase; MW:55887	29.6 protein.(un)folding	3	110
Tb927.7.5790	protein disulfide isomerase, putative [1562540 - 1562947] MW:15400.76	29.6 protein.(un)folding	4	74
Tb927.7.1300	protein disulfide isomerase, putative [334736 - 335869] MW:41939.65	29.6 protein.(un)folding	6	77
Tb10.70.2160	chaperone protein DNAJ, putative [1246723 - 1247595] MW:30863.11	29.6 protein.(un)folding	2	41
Tb927.2.5160	chaperone protein DnaJ, putative [910842 - 912056] MW:44813.55	29.6 protein.(un)folding	2	56
Tb10.389.0880	heat shock protein, putative [3016541 - 3014106] MW:90863.73	29.6 protein.(un)folding	31	97
Tb927.2.5980	HSP100 ATP-dependent Clp protease subunit, MW:97263	29.6 protein.(un)folding	3	50
Tb10.26.1080	heat shock protein 83; heat shock protein [2613113 - 2610999] MW:80763.23	29.6 protein.(un)folding	26	114
Tb11.01.3080	heat shock protein 70, putative [2963244 - 2965274] MW:73630.61	29.6 protein.(un)folding	1	89
Tb11.01.3110	heat shock protein 70 [2971037 - 2973109] MW:75366.42	29.6 protein.(un)folding	18	133
Tb927.7.710	HSP70 heat shock 70 kDa protein, putative [158165 - 156246] MW:70211.63	29.6 protein.(un)folding	7	79
Tb927.6.3740	heat shock 70 kDa protein, MW:71474.85	29.6 protein.(un)folding	3	77

Tb927.6.2170	co-chaperone GrpE, putative [691093 - 691761] MW:23877.13	29.6 protein.(un)folding	2	35
Tb10.70.0280	HSP60 chaperonin Hsp60, mitochondrial precursor [1644448 - 1646136] MW:59503.07	29.6 protein.(un)folding	14	132
Tb927.7.1320	HSP10 10 kDa heat shock protein, putative [341451 - 341753] MW:10670.40	29.6 protein.(un)folding	4	112
Tb11.01.8510	TCP-1-alpha t-complex protein 1, MW:54623.76	29.6 protein.(un)folding	3	71
Tb11.42.0003	TCP-1-beta t-complex protein 1, MW:58057.86	29.6 protein.(un)folding	3	78
Tb10.70.7050	TCP-1-delta t-complex protein 1, MW:58361.67	29.6 protein.(un)folding	4	77
Tb11.01.5860	TCP-1-epsilon t-complex protein 1, MW:59381.19	29.6 protein.(un)folding	3	63
Tb11.02.0750	TCP-1-zeta t-complex protein 1,MW:59579.38	29.6 protein.(un)folding	2	32
Tb10.6k15.2330	TCP-1-theta t-complex protein 1, MW: 58501	29.6 protein.(un)folding	1	98
Tb11.02.5450	glucose-regulated protein 78, putative; MW:71505	29.6 protein.(un)folding	22	99
Tb927.5.2940	stress-induced protein sti1, putative [927151 - 928803] MW:62327.63	35.2 not assigned.unknown	17	102
Tb927.7.570	prefoldin, putative [117587 - 116994] MW:22753.76	29.6 protein.(un)folding	7	93
Tb927.7.2590	prefoldin, putative [667376 - 666864] MW:18777.10	29.6 protein.(un)folding	1	50
Tb10.389.1730	protein kinase, putative; tyrosine protein kinase, putative ; MW:47389.49	29.4 protein.postranslational modification	8	85
Tb11.02.4830	protein kinase, putative; serine/threonine protein kinase, putative ; MW:67118.12	29.4 protein.postranslational modification	1	38
Tb927.1.3200	phosphatase-like protein, putative [690155 - 690820] MW:24493.00	29.4 protein.postranslational modification	2	73
Tb927.6.1800	PP2C protein phosphatase 2C, putative [603410 - 602262] MW:41135.15	29.4 protein.postranslational modification	2	58
Tb927.8.1710	protein phosphatase 2A, regulatory subunit B, putative;MW: 46368	29.4 protein.postranslational modification	4	50
Tb11.02.2210	PKA-R; RSU protein kinase A regulatory subunit [1302874 - 1304373] MW:56734.19	30 signalling	15	76
Tb927.4.5010	calreticulin, putative [1373216 - 1372029] MW:45042.51	30.3 signaling.calcium	21	90
Tb927.8.7410	calreticulin, putative [2134955 - 2136142] MW:45021.67	30.3 signaling.calcium	19	107
Tb927.8.5440	TB-24 flagellar calcium-binding protein TB-24 [1613150 - 1613806] MW:24253.22	30.3 signalling.calcium	1	83
Tb10.61.1880	protein kinase, putative; mitogen-activated protein kinase, putative; MW:31374.9	30.6 signalling.MAP kinases	5	72
Tb927.8.3550	mitogen-activated protein kinase 3, putative [1066185 - 1065070] MW:42672.19	30.6 signalling.MAP kinases	1	79
Tb11.01.1290	14-3-3-like protein, putative [2547863 - 2547075] MW:30310.38	30.7 signalling.14-3-3 proteins	6	89
Tb11.02.4700	14-3-3-like protein, putative [1912331 - 1911573] MW:29196.24	30.7 signalling.14-3-3 proteins	8	98
Tb11.02.2310	prostaglandin f synthase [1332644 - 1333474] MW:30992.51	30.99 signalling.unspecified	6	56
Tb09.211.0620	actin A [1375684 - 1374554] MW:41895.68	31.1 cell.organisation	1	32
Tb10.406.0650	microtubule-associated protein, putative [2492839 - 2486066] MW:254641.15	31.1 cell.organisation	2	82
Tb10.61.1750	TBKIFC1 C-terminal motor kinesin, putative [3561647 - 3564109] MW:90800.49	31.1 cell.organisation	4	104
Tb11.01.5350	profilin [3613516 - 3613968] MW:16122.17	31.1 cell.organisation	1	34
Tb11.50.0007	dynein light chain, putative [173608 - 173336] MW:10428.91	31.1 cell.organisation	2	27
Tb927.1.2330	beta tubulin [570482 - 571810] MW:49703.96	31.1 cell.organisation	9	79
Tb927.1.2340	alpha tubulin [572442 - 573797] MW:49787.13	31.1 cell.organisation	15	99
Tb927.3.4290	PFR-C; PFR-D 73 kDa paraflagellar rod protein;] MW:68682.76	31.1 cell.organisation	7	109
Tb927.3.5180	cofilin/actin depolymerizing factor, putative [1453906 - 1454316] MW:15119.06	31.1 cell.organisation	2	102

Tb927.6.1770	kinesin, putative [592460 - 590574] MW:69409.26	31.1 cell.organisation	1	88
Tb927.6.2130	nuclear movement protein, putative; NUDC-like protein [681443 - 680916] MW:19667.41	31.1 cell.organisation	2	58
Tb927.7.3440	I/6 autoantigen [892212 - 891472] MW:27050.07	31.1 cell.organisation	2	96
Tb927.7.3450	I/6 autoantigen [893109 - 892522] MW:21584.99	31.1 cell.organisation	4	67
Tb927.8.4970	PFR 69 kDa paraflagellar rod protein; PFR2 [1474818 - 1476620] MW:69597.02	31.1 cell.organisation	13	124
Tb927.6.3110	protein kinase, putative; cdc2, putative [919976 - 921295] MW:48868.19	31.3 cell.cycle	1	45
Tb09.160.3710	proliferative cell nuclear antigen (PCNA), putative [865854 - 866732] MW:32314.61	31.3 cell.cycle	8	90
Tb09.211.0740	p21 antigen protein, putative [1398292 - 1397720] MW:21059.98	31.3 cell.cycle	2	60
Tb09.211.2360	PKAC2 protein kinase A catalytic subunit isoform 2;MW: 38716	31.3 cell.cycle	2	32
Tb10.70.7040	CRK1; kin1 cell division protein kinase 2 homolog 1;MW: 34671	31.3 cell.cycle	1	38
Tb11.02.0250	heat shock protein, mitochondrial precursor, putative;MW: 84832	31.3 cell.cycle	1	66
Tb10.70.1190	VCP valosin-containing protein homolog;MW: 86570	29.3 protein.targeting	19	79
Tb09.211.3210	transport protein particle (TRAPP) subunit, putative [1848013 - 1848621] MW:23009.87	31.4 cell. vesicle transport	1	64
Tb09.211.4610	vesicle-associated membrane protein, putative [2146972 - 2146355] MW:23167.44	31.4 cell. vesicle transport	1	34
Tb10.70.0830	CHC clathrin heavy chain [1523188 - 1518077] MW:190625.63	31.4 cell. vesicle transport	1	38
Tb927.3.4720	dynamin, putative; vacuolar sortin protein 1, putative [1336468 - 1334486] MW:73321.94	31.4 cell. vesicle transport	3	55
Tb10.6k15.2500	BAD1 adaptin complex 1 subunit, putative; MW:76279.46	31.4 cell. vesicle transport	2	55
Tb927.4.760	gamma-adaptin 1, putative; AP-1 adapter complex gamma subunit, MW:87344.	31.4 cell. vesicle transport	2	49
Tb927.6.3290	intraflagellar transport (IFT) protein, putative [972730 - 973122] MW:15419.41	31.4 cell. vesicle transport	2	34
Tb11.55.0006	TbIFT88 intraflagellar transport protein IFT88, putative [508741 - 506339] MW:89755.06	31.4 cell. vesicle transport	2	41
Tb10.61.1590	intraflagellar transport protein component, putative [3587443 - 3589410] MW:72377.72	31.4 cell. vesicle transport	1	74
Tb10.6k15.1160	G-actin binding protein, putative; CAP/Srv2p, putative ; MW:29333.07	31.6* cell organization-	4	56
Tb927.8.890	small GTP-binding protein Rab1, putative [266738 - 266112] MW:22714.65	31.4 cell. vesicle transport	4	78
Tb927.3.4680	RAB GDP dissociation inhibitor alpha, putative [1325313 - 1323976] MW:49396.71	31.4 cell. vesicle transport	3	65
Tb10.6k15.3970	developmentally regulated GTP-binding protein, putative ; MW:41267.84	31.4 cell. vesicle transport	2	48
Tb11.01.3740	coatomer gamma subunit, putative; [3120728 - 3123364] MW:97587.26	31.4 cell. vesicle transport	1	32
Tb927.2.6050	beta prime COP protein [1092946 - 1095501] MW:94052.12	31.4 cell. vesicle transport	1	42
Tb11.16.0003	variant surface glycoprotein (VSG), putative [4969971 - 4971455] MW:51467.19	31.99 cell.unspecified	1	23
Tb11.47.0001	65 kDa invariant surface glycoprotein-like protein [428987 - 430300] MW:47801.74	31.99 cell.unspecified	1	31
Tb11.v4.0040	variant surface glycoprotein (VSG), putative [191889 - 190408] MW:52763.30	31.99 cell.unspecified	1	34
Tb10.100.0070	ATP synthase F1 subunit gamma protein, putative [71480 - 72397] MW:34370.09	34.1 transport.p- and v-ATPases	3	33
Tb10.70.7480	ATP synthase, putative [186531 - 187937] MW:52900.99	34.1 transport.p- and v-ATPases	2	48
Tb11.01.1190	ATP synthase, putative [2528152 - 2527502] MW:24788.35	34.1 transport.p- and v-ATPases	2	103
Tb927.4.1080	V-type ATPase, A subunit, putative [289967 - 288135] MW:67749.33	34.1 transport.p- and v-ATPases	8	112
Tb927.6.4990	ATP synthase, epsilon chain, putative [1370982 - 1371530] MW:20146.88	34.1 transport.p- and v-ATPases	2	77
Tb927.7.7420	ATP synthase alpha chain; ATP synthase F1, MW: 63862	34.1 transport.p- and v-ATPases	6	62

Tb10.61.0380	glycerol uptake protein, putative [3850573 - 3852402] MW:69718.53	34.2 transporter.sugars	1	29
Tb927.3.1120	rtb2 GTP-binding nuclear protein rtb2, putative [273590 - 274243] MW:24405.85	34.99 transport.misc	1	41
Tb10.61.2680	PYK1 pyruvate kinase 1 [3364197 - 3363142] MW:38453.32	4 glycolysis	8	69
Tb10.6k15.3850	glyceraldehyde 3-phosphate dehydrogenase, cytosolic MW:35610.62	4 glycolysis	3	105
Tb10.70.1370	fructose-bisphosphate aldolase, glycosomal, putative; MW:41071.12	4 glycolysis	13	74
Tb927.1.700	PGKC; gPGK phosphoglycerate kinase [233826 - 232504] MW:47245.76	4 glycolysis	4	83
Tb10.70.4740	enolase [745416 - 746705] MW:46592.13	4.12 glycolysis.enolase	14	132
Tb10.6k15.2620	2,3-bisphosphoglycerate-independent phosphoglycerate mutase MW:60603.34	4.4.11 glycolysis.	11	115
Tb927.6.1570	2-hydroxy-3-oxopropionate reductase, putative [544399 - 543497] MW:31752.74	6 gluconeogenese/ glyoxylate cycle	2	69
Tb10.61.0980	gMDH glycosomal malate dehydrogenase [3714549 - 3715520] MW:33710.35	6.3 gluconeogenesis.Malate DH	1	30
Tb11.01.3040	cytosolic malate dehydrogenase, putative [2954034 - 2955020] MW:35151.21	6.3 gluconeogenesis.Malate DH	13	115
Tb927.2.4210	glycosomal phosphoenolpyruvate carboxykinase; MW:58564.86	6.4 gluconeogenese/ glyoxylate cycle.	1	43
Tb11.01.8520	glucosamine-6-phosphate isomerase, putative [4403503 - 4402661] MW:31087.32	7.1.2 OPP.oxidative PP.	8	79
Tb11.02.4200	6PGL 6-phosphogluconolactonase [1758790 - 1757990] MW:28649.81	7.1.2 OPP.oxidative PP.	4	69
Tb927.8.5600	transaldolase, putative [1663004 - 1664002] MW:36626.86	7.2.2 OPP.non-reductive PP.	11	79
Tb11.01.0700	ribose 5-phosphate isomerase, putative [2408554 - 2409021] MW:16957.30	7.2.4 OPP.non-reductive PP.	1	80
Tb10.6k15.3250	succinyl-CoA ligase [GDP-forming] beta-chain, MW:54807.20	8.1.6 TCA / org. transformation.	4	81
Tb927.3.2230	succinyl-CoA synthetase alpha subunit, putative [581836 - 580931] MW:31464.60	8.1.6 TCA / org. transformation.	2	45
Tb10.6k15.3600	myo-inositol-1-phosphate synthase, putative [1769650 - 1771236] MW:58274.40	3.4.3 minor CHO metabolism.	2	57
Tb927.8.980	phosphoacetylglucosamine mutase, putative; MW: 65651	3 minor CHO metabolism	1	73
Tb11.02.0290	succinyl-coA:3-ketoacid-coenzyme A transferase, MW:53576	11.1.10 lipid metabolism.	3	110
Tb09.211.3540	glk1; gk glycerol kinase, glycosomal [1917219 - 1918757] MW:56335.85	11.5 lipid metabolism.glycerol metabolism	1	42
Tb927.8.3530	glycerol-3-phosphate dehydrogenase [NAD+], glycosomal MW:37805.01	11.5.2 lipid metabolism.glyceral metabolism.	4	78
Tb927.8.1440	maoC-like dehydratase, putative [469241 - 469729] MW:17815.52	11.8 lipid metabolism.exotics	1	46
Tb927.1.4830	phospholipase A1, putative [986718 - 987620] MW:32403.83	11.9.3 lipid metabolism.lipid degradation.	3	96
Tb11.01.5730	ethanolamine-phosphate cytidylyltransferase, putative;MW:43424.77	11.5 lipid metabolism.glycerol metabolism	1	34
Tb927.6.2740	pdxK pyridoxal kinase [823608 - 824510] MW:33332.03	18.20* Co-factor and vitamine metabolism.vitar	8	114
Tb927.8.2210	pteridine reductase, putative [677634 - 678743] MW:40289.31	18.5 Co-factor and vitamine metabolism.	2	70
Tb09.160.0770	nitrilase, putative [429953 - 429132] MW:30298.74	22.1.5 polyamine metabolism.	7	71
Tb09.v1.0380	spermidine synthase, putative [1225692 - 1224796] MW:32926.41	22.1.6 polyamine metabolism.	6	60
Tb09.160.0465	hypothetical protein, conserved [351811 - 351554] MW:9759.29	35.1 not assigned.no ontology	2	99
Tb09.160.1160	hypothetical protein, conserved [521174 - 518901] MW:85913.86	35.1 not assigned.no ontology	1	49
Tb09.160.1180	hypothetical protein, conserved [523907 - 522177] MW:66256.87	35.1 not assigned.no ontology	3	46
Tb09.160.5200	hypothetical protein, conserved [1103278 - 1104165] MW:32572.74	35.1 not assigned.no ontology	1	57
Tb09.160.5530	hypothetical protein, conserved [1184832 - 1183792] MW:38016.33	35.1 not assigned.no ontology	1	94
Tb09.211.1690	hypothetical protein, conserved [1558872 - 1558366] MW:18768.80	35.1 not assigned.no ontology	1	47

Tb09.211.3180	6-phosphogluconate dehydrogenase, MW:52165.75	7.1.2 OPP.oxidative PP.	6	62
Tb09.211.3350	hypothetical protein, conserved [1872213 - 1874258] MW:76666.61	35.1 not assigned.no ontology	1	37
Tb09.211.4240	phosphoinositide-binding protein, putative [2069958 - 2068699] MW:48172.33	35.1 not assigned.no ontology	7	122
Tb09.v1.0660	hypothetical protein, unlikely [1812682 - 1812804] MW:4529.59	35.1 not assigned.no ontology	1	27
Tb10.389.0570	hypothetical protein, conserved; predicted zinc finger protein MW:34477.06	35.1 not assigned.no ontology	1	32
Tb10.389.0720	hypothetical protein, conserved [3045775 - 3042734] MW:110243.85	35.1 not assigned.no ontology	9	95
Tb10.389.1070	hypothetical protein, conserved [2982652 - 2979851] MW:105847.36	35.1 not assigned.no ontology	1	58
Tb10.61.0540	hypothetical protein, conserved [3824087 - 3825034] MW:36411.55	35.1 not assigned.no ontology	2	70
Tb10.61.2370	hypothetical protein, conserved [3429745 - 3428078] MW:60759.48	35.1 not assigned.no ontology	1	40
Tb10.61.2670	hypothetical protein, conserved [3367350 - 3365911] MW:55311.17	35.1 not assigned.no ontology	5	77
Tb10.61.3210	hypothetical protein, conserved [3279514 - 3278519] MW:37612.28	35.1 not assigned.no ontology	2	75
Tb10.70.1130	hypothetical protein, conserved [1441960 - 1440659] MW:48257.68	35.1 not assigned.no ontology	8	123
Tb10.70.1490	hypothetical protein, conserved [1384855 - 1385826] MW:36137.95	35.1 not assigned.no ontology	1	92
Tb10.70.3070	hypothetical protein, conserved [1100000 - 1099257] MW:28189.10	35.1 not assigned.no ontology	1	56
Tb10.70.4930	hypothetical protein, conserved [700304 - 701188] MW:32833.06	35.1 not assigned.no ontology	2	43
Tb10.70.6610	hypothetical protein, conserved [374200 - 373463] MW:27338.99	35.1 not assigned.no ontology	3	73
Tb11.01.0320	hypothetical protein, conserved [2256203 - 2257396] MW:45975.96	35.1 not assigned.no ontology	4	76
Tb11.01.0950	hypothetical protein, conserved [2471414 - 2473114] MW:62384.20	35.1 not assigned.no ontology	1	29
Tb11.01.2790	hypothetical protein, conserved [2883272 - 2884753] MW:54823.94	35.1 not assigned.no ontology	1	31
Tb11.01.5170	hypothetical protein, conserved [3567908 - 3567516] MW:14737.54	35.1 not assigned.no ontology	1	28
Tb11.01.5680	hypothetical protein, conserved [3671680 - 3673122] MW:51674.18	35.1 not assigned.no ontology	4	99
Tb11.01.6510	hypothetical protein, conserved [3892801 - 3891686] MW:41086.00	35.1 not assigned.no ontology	1	83
Tb11.01.6690	hypothetical protein, conserved [3944798 - 3943368] MW:51912.37	35.1 not assigned.no ontology	1	28
Tb11.01.7010	hypothetical protein, conserved [4017280 - 4014062] MW:117882.50	35.1 not assigned.no ontology	20	114
Tb11.01.7120	hypothetical protein, conserved [4055862 - 4055236] MW:23084.79	35.1 not assigned.no ontology	1	49
Tb11.02.0210	hypothetical protein, conserved [747704 - 746319] MW:50887.21	35.1 not assigned.no ontology	2	51
Tb11.02.0300	hypothetical protein, conserved [775298 - 773409] MW:69229.86	35.1 not assigned.no ontology	1	27
Tb11.02.0660	hypothetical protein, conserved [879024 - 877852] MW:43836.49	35.1 not assigned.no ontology	2	58
Tb11.02.1510	hypothetical protein, conserved [1152288 - 1155722] MW:127425.46	35.1 not assigned.no ontology	1	50
Tb11.02.1690	hypothetical protein, conserved [1209703 - 1207811] MW:69793.86	35.1 not assigned.no ontology	6	69
Tb11.02.2030	hypothetical protein, conserved [1267722 - 1268075] MW:12757.37	35.1 not assigned.no ontology	2	105
Tb11.02.2040	hypothetical protein, conserved [1268639 - 1269016] MW:14037.89	35.1 not assigned.no ontology	3	57
Tb11.02.4250	hypothetical protein, conserved [1773128 - 1772250] MW:32851.85	35.1 not assigned.no ontology	3	95
Tb11.02.5550	hypothetical protein, conserved; predicted WD40 repeat protein;MW:67695.84	35.1 not assigned.no ontology	5	75
Tb11.03.0530	hypothetical protein, conserved [140958 - 140116] MW:31292.20	35.1 not assigned.no ontology	1	45
Tb11.52.0013	hypothetical protein, conserved [3407660 - 3406476] MW:43332.89	35.1 not assigned.no ontology	4	65

Tb11.55.0024	hypothetical protein, conserved [468674 - 467679] MW:37610.93	35.1 not assigned.no ontology	6	82
Tb927.1.1100	hypothetical protein, conserved [308704 - 311616] MW:108810.61	35.1 not assigned.no ontology	1	35
Tb927.1.120	retrotransposon hot spot (RHS) protein, putative; MW:98534	35.1 not assigned.no ontology	4	56
Tb927.1.180	retrotransposon hot spot (RHS) protein, putative; MW:94829	35.1 not assigned.no ontology	10	72
Tb927.1.420	retrotransposon hot spot (RHS) protein, putative; MW:76698	35.1 not assigned.no ontology	1	40
Tb927.1.4480	hypothetical protein, conserved [923539 - 927993] MW:158447.08	35.1 not assigned.no ontology	1	43
Tb927.2.100	retrotransposon hot spot (RHS) protein, putative; MW:95216	35.1 not assigned.no ontology	2	41
Tb927.2.1180	retrotransposon hot spot (RHS) protein, putative;MW:68024	35.1 not assigned.no ontology	1	43
Tb927.2.2230	hypothetical protein, conserved [427069 - 425903] MW:42703.14	35.1 not assigned.no ontology	1	34
Tb927.2.2770	hypothetical protein, conserved [541346 - 541723] MW:13445.37	35.1 not assigned.no ontology	2	53
Tb927.2.280	retrotransposon hot spot (RHS) protein, putative;MW: 90678	35.1 not assigned.no ontology	1	69
Tb927.2.340	retrotransposon hot spot (RHS) protein, putative; MW: 98350	35.1 not assigned.no ontology	3	54
Tb927.2.380	retrotransposon hot spot (RHS) protein, putative; MW:91171	35.1 not assigned.no ontology	1	63
Tb927.2.3800	hypothetical protein, conserved [679117 - 677639] MW:55460.11	35.1 not assigned.no ontology	1	68
Tb927.2.470	retrotransposon hot spot (RHS) protein, putative; MW: 98769	35.1 not assigned.no ontology	1	88
Tb927.3.1590	hypothetical protein, conserved [417246 - 415240] MW:72678.15	35.1 not assigned.no ontology	6	73
Tb927.3.2100	hypothetical protein, conserved [553951 - 553025] MW:33996.14	35.1 not assigned.no ontology	4	71
Tb927.3.2220	hypothetical protein, conserved [580061 - 579399] MW:23800.14	35.1 not assigned.no ontology	1	50
Tb927.3.3560	hypothetical protein, conserved [1001768 - 1003642] MW:69087.74	35.1 not assigned.no ontology	9	86
Tb927.3.4040	hypothetical protein, conserved [1142118 - 1140259] MW:67631.70	35.1 not assigned.no ontology	1	61
Tb927.3.4180	hypothetical protein [1174436 - 1173210] MW:43143.08	35.1 not assigned.no ontology	1	50
Tb927.3.5320	hypothetical protein, conserved [1496116 - 1497300] MW:43835.12	35.1 not assigned.no ontology	2	52
Tb927.3.5490	hypothetical protein, conserved [1535292 - 1537319] MW:77659.71	35.1 not assigned.no ontology	1	48
Tb927.4.1300	hypothetical protein, conserved [342776 - 341652] MW:42014.86	35.1 not assigned.no ontology	5	66
Tb927.4.1850	hypothetical protein, conserved [464351 - 462615] MW:64384.15	35.1 not assigned.no ontology	1	50
Tb927.4.2030	hypothetical protein, conserved [511106 - 510453] MW:22703.92	35.1 not assigned.no ontology	5	85
Tb927.4.2040	hypothetical protein, conserved [513261 - 512689] MW:20808.23	35.1 not assigned.no ontology	4	55
Tb927.4.2280	hypothetical protein, conserved [593290 - 594438] MW:42285.16	35.1 not assigned.no ontology	1	36
Tb927.4.2740	hypothetical protein, conserved [723054 - 723506] MW:16327.52	35.1 not assigned.no ontology	1	44
Tb927.4.3740	hypothetical protein, conserved [945122 - 940044] MW:192569.03	35.1 not assigned.no ontology	1	41
Tb927.4.4240	hypothetical protein, conserved [1126471 - 1125479] MW:36183.86	35.1 not assigned.no ontology	1	32
Tb927.4.4770	hypothetical protein [1311562 - 1310579] MW:34906.10	35.1 not assigned.no ontology	1	44
Tb927.4.730/	hypothetical protein, conserved [205139 - 203661] MW:55016.63	35.1 not assigned.no ontology	1	64
Tb927.5.1160	hypothetical protein, conserved [395802 - 396488] MW:25569.59	35.1 not assigned.no ontology	1	42
Tb927.5.1360/	hypothetical protein, conserved [441346 - 441807] MW:17297.74	35.1 not assigned.no ontology	4	55
Tb927.5.1460	hypothetical protein, conserved [465482 - 464502] MW:36762.16	35.1 not assigned.no ontology	14	110

Tb927.5.2890	hypothetical protein, conserved [912979 - 906488] MW:236856.89	35.1 not assigned.no ontology	1	35
Tb927.5.4570	hypothetical protein, conserved [1356572 - 1359028] MW:88866.90	35.1 not assigned.no ontology	3	52
Tb927.5.780	hypothetical protein, conserved [266344 - 267906] MW:55350.13	35.1 not assigned.no ontology	1	37
Tb927.6.1290	hypothetical protein, conserved [475628 - 472929] MW:100579.20	35.1 not assigned.no ontology	2	73
Tb927.6.1990	hypothetical protein, conserved [651077 - 650103] MW:37832.43	35.1 not assigned.no ontology	1	59
Tb927.6.2200	hypothetical protein, conserved [698549 - 699142] MW:21125.70	35.1 not assigned.no ontology	1	55
Tb927.6.4140/	hypothetical protein, conserved [1195110 - 1194763] MW:13008.76	35.1 not assigned.no ontology	1	33
Tb927.6.4440	hypothetical protein, conserved [1248097 - 1247039] MW:37718.41	35.1 not assigned.no ontology	1	47
Tb927.6.4670	hypothetical protein, conserved [1303156 - 1304232] MW:40686.20	35.1 not assigned.no ontology	1	69
Tb927.6.5070/	hypothetical protein, conserved [1383939 - 1385303] MW:51821.01	35.1 not assigned.no ontology	7	80
Tb927.7.1290/	hypothetical protein, conserved [333218 - 333946] MW:26998.71	35.1 not assigned.no ontology	1	22
Tb927.7.1330/	hypothetical protein, conserved [342229 - 343296] MW:39755.38	35.1 not assigned.no ontology	2	65
Tb927.7.180	hypothetical protein [28385 - 27072] MW:49811.88	35.1 not assigned.no ontology	1	31
Tb927.7.2240	hypothetical protein, conserved [580626 - 578992] MW:59665.51	35.1 not assigned.no ontology	2	64
Tb927.7.2260	hypothetical protein, conserved [585228 - 584389] MW:30058.81	35.1 not assigned.no ontology	1	47
Tb927.7.2640	hypothetical protein, conserved [678317 - 676956] MW:50853.10	35.1 not assigned.no ontology	1	54
Tb927.7.3370	hypothetical protein, conserved [881750 - 879960] MW:67485.37	35.1 not assigned.no ontology	2	48
Tb927.7.4290	hypothetical protein, conserved [1140765 - 1139872] MW:33703.32	35.1 not assigned.no ontology	3	65
Tb927.7.5210	hypothetical protein, conserved [1372419 - 1375115] MW:100479.79	35.1 not assigned.no ontology	4	69
Tb927.7.6090	hypothetical protein, conserved [1661292 - 1659004] MW:88162.89	35.1 not assigned.no ontology	3	39
Tb927.7.6860	expression site-associated gene (ESAG) protein, putative; MW : 52337	35.1 not assigned.no ontology	1	65
Tb927.7.7140	hypothetical protein, conserved [2045985 - 2045035] MW:35064.14	35.1 not assigned.no ontology	1	35
Tb927.8.1960	hypothetical protein, conserved [629740 - 631044] MW:48425.71	35.1 not assigned.no ontology	3	60
Tb927.8.3680	hypothetical protein, conserved [1102829 - 1100892] MW:69597.10	35.1 not assigned.no ontology	1	37
Tb927.8.6270	hypothetical protein, conserved [1822881 - 1824698] MW:69168.79	35.1 not assigned.no ontology	1	29
Tb927.8.6280	hypothetical protein, conserved [1825049 - 1825798] MW:27122.89	35.1 not assigned.no ontology	1	29
Tb927.8.6660	hypothetical protein, conserved [1930528 - 1928705] MW:69055.48	35.1 not assigned.no ontology	2	43
Tb10.70.2770	stress-inducible protein STI1-like, putative [1145116 - 1144343] MW:28424.92	35.2 not assigned.unknown	17	102

Table S2 / BN-PAGE gel
SHEET 3

All secretome proteins (BN+1D Non redundant)	Accession number
	Tb927.6.3740/Tb06.4F7.750
	Tb927.6.4670/Tb06.26G9.330
	Tb927.8.980/Tb08.25L8.80
	Tb11.01.1370
	Tb09.211.0120
	Tb11.01.3020
	Tb927.6.4480/Tb06.26G9.740
	Tb10.61.0980
	Tb927.7.5210/Tb07.27E10.470
	Tb10.70.7695
	Tb11.46.0001
	Tb927.3.5320/Tb03.5L5.500
	Tb927.8.3550/Tb08.10J17.940
	Tb927.7.6860/Tb07.25D22.140
	Tb09.211.2360
	Tb11.02.1510
	Tb927.6.4590/Tb06.26G9.450
	Tb927.5.1160/Tb05.30H13.70
	Tb927.7.570/Tb07.8P12.860
	Tb927.3.3310/Tb03.3K10.380
	Tb927.2.4210/28H13.455
	Tb10.70.1380
	Tb11.01.1190
	Tb09.211.0620
	Tb927.3.5490/Tb03.5L5.700
	Tb09.211.0740
	Tb09.211.4330
	Tb927.8.6270/Tb08.11J15.440
	Tb927.6.4980/Tb06.30P15.510
	Tb927.4.760/Tb04.5E12.910
	Tb11.01.7010
	Tb09.211.4240

Tb927.4.1080/Tb04.5E12.370
Tb09.V1.0660
Tb927.2.340/3B10.125
Tb927.7.6090/Tb07.2F2.320
Tb927.2.470/3B10.190
Tb11.01.0950
Tb927.4.3740/Tb04.26G5.680
Tb09.211.2590
Tb927.6.1990/Tb06.4M18.80
Tb09.160.5560
Tb09.160.5200
Tb10.6K15.0990
Tb10.61.2670
Tb11.01.8520
Tb11.01.1570
Tb927.4.1850/Tb04.29M18.150
Tb927.4.4770/Tb04.3I12.120
Tb927.8.1600/Tb08.29O9.340
Tb927.6.2200/Tb06.4M18.560
Tb927.2.2230/25N14.150
Tb927.6.2790/Tb06.5F5.290
Tb11.02.0660
Tb927.2.1180/25N24.100
Tb09.211.4540
Tb927.7.1300/Tb07.27M11.560
Tb11.42.0003
Tb10.70.6360
Tb09.211.3180
Tb927.6.3110/Tb06.5F5.880
Tb927.3.2220/Tb03.48O8.730
Tb927.5.1000/Tb05.28F8.340
Tb11.22.0005
Tb927.3.5340/Tb03.5L5.520
Tb927.8.3680/Tb08.10J17.750
Tb11.01.7120
Tb10.61.2090

Tb927.2.2770/10C8.185
Tb927.4.3790/Tb04.30O21.180
Tb09.211.0110
Tb927.6.4440/Tb06.26G9.840
Tb11.V4.0040
Tb09.160.0770
Tb11.55.0006
Tb927.4.430/Tb04.5D20.530
Tb927.7.7420/Tb07.30D13.360
Tb927.6.5070/Tb06.30P15.360
Tb927.3.2230/Tb03.48O8.710
Tb10.61.1870
Tb10.6K15.2220
Tb10.6K15.1220
Tb09.160.4620
Tb11.01.6510
Tb11.01.0320
Tb10.6K15.2500
Tb11.01.3740
Tb09.160.1180
Tb927.6.1770/Tb06.28P18.680
Tb927.8.7020/Tb08.10K10.800
Tb927.2.280/3B10.95
Tb927.2.6050/36E18.25
Tb927.7.2100/Tb07.43M14.550
Tb927.7.5170/Tb07.27E10.400
Tb11.02.0250
Tb927.5.780/Tb05.28F8.790
Tb927.8.6280/Tb08.11J15.430
Tb927.3.1120/Tb03.27F10.90
Tb11.01.5170
Tb11.02.4830
Tb10.70.1100
Tb927.7.3370/Tb07.28B13.700
Tb11.02.0290
Tb10.70.2160

Tb10.26.0560
Tb11.02.0750
Tb927.7.3620/Tb07.28B13.380
Tb927.6.4990/Tb06.30P15.500
Tb10.70.0280
Tb10.389.0910
Tb11.02.2210
Tb10.70.2490
Tb927.7.1290/Tb07.27M11.530
Tb927.7.2260/Tb07.22O10.210
Tb927.5.1360/Tb05.30H13.400
Tb927.5.4570/Tb05.26C7.190
Tb927.1.4830
Tb927.8.8330/Tb08.28A12.350
Tb927.7.3450/Tb07.28B13.620
Tb09.211.4610
Tb927.2.2440/25N14.45
Tb927.3.4040/Tb03.28C22.60
Tb927.6.2130/Tb06.4M18.380
Tb09.160.0580
Tb927.2.5980/1F7.360
Tb10.6K15.3600
Tb09.211.1250
Tb10.05.0220
Tb927.3.5180/Tb03.5L5.210
Tb10.70.7050
Tb09.160.5550
Tb10.70.1490
Tb11.01.6670
Tb10.389.1070
Tb11.01.4830
Tb11.01.6660
Tb11.52.0013
Tb09.160.1950
Tb09.211.4880
Tb927.8.1710/Tb08.29O9.490

Tb927.3.4180/Tb03.26J7.310
Tb927.6.4140/Tb06.4F7.140
Tb09.211.3350
Tb927.7.180/Tb07.8P12.190
Tb927.4.2450/Tb04.1H19.870
Tb927.6.1290/Tb06.3A7.840
Tb11.03.0620
Tb927.3.4840/Tb03.48K5.520
Tb09.160.0465
Tb10.389.0570
Tb11.01.6030
Tb10.6K15.2330
Tb927.6.700/Tb06.3D8.610
Tb927.5.3510/Tb05.6E7.170
Tb10.70.3290
Tb11.02.5550
Tb927.5.4500/Tb05.26C7.80
Tb927.7.5160/Tb07.27E10.390
Tb09.160.4200
Tb10.70.7330
Tb927.4.4240/Tb04.1D20.250
Tb10.406.0330
Tb11.02.5170
Tb11.01.6690
Tb10.61.0380
Tb11.01.2790
Tb11.01.0700
Tb927.5.2570/Tb05.26K5.1010
Tb09.211.2630
Tb927.2.100/3B10.5
Tb927.5.2890/Tb05.26K5.300
Tb09.211.2150
Tb927.5.1700/Tb05.1P6.900
Tb11.01.8770
Tb11.02.4250
Tb927.8.1960/Tb08.26N11.430

Tb10.61.1960
Tb10.61.2370
Tb10.389.0630
Tb927.8.7410/Tb08.10K10.160
Tb927.7.2640/Tb07.22O10.840
Tb927.8.6660/Tb08.30K1.530
Tb927.7.7140/Tb07.21H15.220
Tb927.8.6060/Tb08.11J15.760
Tb11.01.5860
Tb10.100.0070
Tb11.02.0210
Tb11.50.0007
Tb927.8.2640/Tb08.26A17.680
Tb927.4.3590/Tb04.26G5.430
Tb927.6.3290/Tb06.2N9.700
Tb927.3.3410/Tb03.3K10.500
Tb927.5.1090/Tb05.28F8.150
Tb11.01.5710
Tb927.3.1590/Tb03.30P12.70
Tb11.01.7960
Tb927.3.5520/Tb03.5L5.730
Tb11.02.1690
Tb927.4.2280/Tb04.1H19.550
Tb927.4.3550/Tb04.26G5.360
Tb927.4.730/Tb04.5E12.960
Tb09.211.3210
Tb10.61.1590
Tb927.7.1320/Tb07.27M11.580
Tb927.6.2170/Tb06.4M18.480
Tb927.8.5880/Tb08.11J15.1060
Tb927.2.380/3B10.145
Tb927.1.120
Tb11.02.2030
Tb927.2.4710/30M24.225
Tb10.70.7480
Tb11.02.4200

Tb10.70.7010
Tb11.03.0530
Tb927.8.1440/Tb08.29O9.70
Tb927.7.2590/Tb07.22O10.720
Tb09.160.3730
Tb927.4.1270/Tb04.2L9.390
Tb927.7.2240/Tb07.22O10.180
Tb11.01.0870
Tb09.160.3590
Tb10.6K15.3250
Tb927.3.4750/Tb03.48K5.320
Tb11.01.5730
Tb10.389.1480
Tb09.211.4850
Tb927.2.4370/30M24.395
Tb927.2.3800/28H13.250
Tb927.1.420
Tb927.1.4480
Tb11.01.5350
Tb11.16.0003
Tb10.406.0650
Tb10.70.3510
Tb09.160.4450
Tb927.3.780/Tb03.27F10.710
Tb927.3.5050/Tb03.48K5.830
Tb09.160.2550
Tb927.8.2210/Tb08.26N11.790
Tb927.7.1330/Tb07.27M11.600
Tb11.02.5450
Tb927.7.1120/Tb07.27M11.270
Tb11.02.3210
Tb11.V4.0004
Tb927.3.3560/Tb03.25B21.20
Tb09.160.1160
Tb927.1.1100
Tb10.6K15.3970

Tb927.8.4430/Tb08.29H22.830
Tb927.7.710/Tb07.29K4.620
Tb927.6.2740/Tb06.5F5.240
Tb10.6K15.1160
Tb11.02.4700
Tb10.61.0540
Tb09.160.4570
Tb927.6.400/Tb06.28F21.350
Tb10.70.7040
Tb11.01.1680
Tb10.6K15.2050
Tb10.26.0680
Tb11.55.0024
Tb09.211.4460
Tb927.1.2340
Tb10.61.1880
Tb10.70.6610
Tb11.01.7800
Tb927.7.4790/Tb07.26A24.340
Tb10.6K15.2290
Tb927.1.180
Tb927.7.7040/Tb07.21H15.360
Tb927.5.1460/Tb05.30H13.580
Tb927.7.190/Tb07.8P12.250
Tb10.70.1130
Tb09.160.5530
Tb11.02.2310
Tb11.02.1085
Tb927.7.3440/Tb07.28B13.630
Tb11.01.3040
Tb11.01.1350
Tb10.70.2650
Tb10.70.4740
Tb927.7.4290/Tb07.5F10.150
Tb11.02.1070
Tb927.4.2030/Tb04.29M18.690

Tb09.211.3610
Tb11.02.4870
Tb10.26.0200
Tb10.70.2770
Tb927.5.2940/Tb05.26K5.210
Tb927.3.4720/Tb03.48K5.290
Tb927.8.740/Tb08.12O16.310
Tb10.70.7730
Tb11.01.2560
Tb09.160.3270
Tb927.8.3530/Tb08.28L1.740
Tb927.6.1080/Tb06.3A7.500
Tb10.389.0880
Tb09.211.4550
Tb10.100.0170
Tb927.7.4570/Tb07.26A24.720
Tb927.3.2100/Tb03.30P12.1130
Tb11.01.3080
Tb11.01.3110
Tb927.4.5010/Tb04.3M17.390
Tb09.160.4250
Tb09.160.3710
Tb927.7.1110/Tb07.27M11.260
Tb10.389.1730
Tb11.02.1120
Tb11.02.0730
Tb927.7.5790/Tb07.10C21.170
Tb10.61.2680
Tb927.3.2960/Tb03.27C5.420
Tb10.70.0850
Tb10.61.1750
Tb10.70.3360
Tb11.01.7550
Tb10.70.3070
Tb927.4.3570/Tb04.26G5.400
Tb09.160.4590

Tb10.26.1080
Tb09.211.1350
Tb11.01.8510
Tb10.6K15.2520
Tb11.47.0001
Tb927.6.1570/Tb06.28P18.310
Tb11.46.0008
Tb09.211.0560
Tb11.02.0100
Tb927.1.3200
Tb10.100.0120
Tb927.4.1300/Tb04.2L9.440
Tb10.61.0180
Tb09.211.3540
Tb09.244.2730
Tb10.406.0520
Tb10.70.0790
Tb927.1.700
Tb10.6K15.3850
Tb11.01.4660
Tb10.70.4930
Tb11.03.0410
Tb927.8.5440/Tb08.5H5.50
Tb10.70.6540
Tb11.02.5020
Tb927.7.4770/Tb07.26A24.370
Tb11.01.1290
Tb11.02.0815
Tb11.02.2040
Tb927.7.4420/Tb07.26A24.1040
Tb927.5.3830/Tb05.6E7.780
Tb927.4.2740/Tb04.2H8.1370
Tb927.3.4290/Tb03.26J7.510
Tb927.3.3450/Tb03.25B21.123
Tb927.4.2040/Tb04.29M18.710
Tb10.70.5650

Tb11.02.4440
Tb10.6K15.2620
Tb10.70.3710
Tb10.70.1190
Tb10.70.1370
Tb10.70.3660
Tb11.01.5680
Tb927.6.1800/Tb06.28P18.780
Tb09.211.1690
Tb09.V1.0380
Tb10.70.0830
Tb927.5.3820/Tb05.6E7.770
Tb927.8.4970/Tb08.5H5.920
Tb927.8.890/Tb08.12O16.530
Tb927.6.4840/Tb06.30P15.650
Tb927.1.2330
Tb927.2.4590/30M24.285
Tb927.3.4680/Tb03.48K5.180
Tb10.61.3210
Tb11.02.0300
Tb11.01.3170
Tb11.02.1210
Tb927.8.5600/Tb08.26E13.220
Tb10.70.6470
Tb927.2.5160/30J2.30
Tb927.6.1260/Tb06.3A7.810
Tb927.1.3950
Tb927.3.3490/Tb03.25B21.90
Tb10.389.0720
Tb11.03.0250
Tb927.8.2050/Tb08.26N11.540
Tb927.8.1990/Tb08.26N11.460
Tb10.70.5360
Tb927.6.3650/Tb06.4F7.880
Tb11.01.8740
Tb09.160.4560

Tb927.8.6390/Tb08.11J15.160
Tb09.211.0350
Tb10.6K15.3580
Tb10.05.0110
Tb927.4.2110/Tb04.29M18.870
Tb927.7.4060/Tb07.5F10.560
Tb11.02.0140
Tb927.6.2360/Tb06.4M18.780
Tb10.406.0560
Tb927.6.4280/Tb06.26G9.1050
Tb09.160.2960
Tb11.01.8470
Tb10.70.1250
Tb927.5.1410/Tb05.30H13.510
Tb09.211.4511
Tb927.8.2260/Tb08.26A17.40
Tb927.3.1680/Tb03.30P12.280
Tb09.211.2410
Tb11.01.8400
Tb927.7.1780/Tb07.43M14.180
Tb10.70.5250
Tb09.160.0260
Tb927.1.2100
Tb09.211.2700
Tb09.211.3550
Tb11.52.0003
Tb09.211.0610
Tb927.4.2490/Tb04.1H19.950
Tb927.4.860/Tb04.5E12.720
Tb927.6.4770/Tb06.26G9.150
Tb10.70.4840
Tb11.02.2520
Tb11.46.0009
Tb927.8.4230/Tb08.29H22.370
Tb09.160.3630
Tb927.8.6750/Tb08.30K1.660

Tb927.2.4580/30M24.290
Tb927.8.1170/Tb08.29O4.300
Tb10.70.1670
Tb11.42.0004
Tb10.70.5800
Tb09.211.4450
Tb927.8.4640/Tb08.4A8.250
Tb10.70.5630
Tb11.01.4621
Tb927.7.4520/Tb07.26A24.810
Tb927.4.380/Tb04.5D20.460
Tb927.6.2300/Tb06.4M18.700
Tb11.01.0120
Tb09.211.0690
Tb927.3.3760/Tb03.28C22.740
Tb10.70.6660
