# Supplementary material for: Exocytosis and protein secretion in Trypanosoma
Source: BMC Microbiol. 2010 Jan 26;10:20. doi: 10.1186/1471-2180-10-20 (PMC3224696; doi:10.1186/1471-2180-10-20)
Supplement: Additional file 3 — Table S3. Secreted proteins from Leishmania donovanii and their corresponding Trypanosoma orthologs. contains the list of 358 proteins from L. donovanii identified in Silverman et al., 2008 [20] which were blasted against the T. brucei genome. The blast e scores > e-50 were reported as positive identification of T. brucei orthologs. Functional categories were assigned to L. donovanii-secreted proteins as well as the transmembrane span prediction (TMHMM) of these proteins. [file 1471-2180-10-20-S3.PDF]

Table S3

Lm accession	protein name	Tb accession	Blast e score	MapMan bins	TMHMM prediction
LmjF14.1360	myo-inositol-1-phosphate synthase	Tb10.6k15.3600	1.1e-194	3.4.3 minor CHO metabolism.	0
LmjF23.0200	endoribonuclease L-PSP (pb5), putative			27.1.19 RNA.processing.ribonucleases	0
LmjF15.1203	60S acidic ribosomal protein P2			29.2.2 protein.synthesis.misc ribososomal protein	0
LmjF35.2420	phosphoinositide-binding protein, putative			35.1 not assigned.no ontology	0
LmjF16.0140	eukaryotic translation initiation factor 1A, putative	Tb927.8.5880	1.8e-57	29.2.3 protein.synthesis.initiation	0
LmjF32.2180	hypothetical protein, conserved			35.1 not assigned.no ontology	0
LmjF11.0630	aminopeptidase, putative,metallo-peptidase, Clan MF, Family M17	Tb11.02.4440	1.4e-95	29.5 protein.degradation	0
LmjF35.3340	6-phosphogluconate dehydrogenase, decarboxylating, putative	Tb09.211.3180	4.6e-190	7.1.3 OPP.oxidative PP.	0
LmjF04.0310	beta-fructofuranosidase, putative			2.2.1.3.1 major CHO metabolism.degradation.sucrose.	1
LmjF36.3840	glycyl tRNA synthetase, putative	Tb11.01.1400	1.6e-231	29.1.14 protein.aa activation.	0
LmjF36.5620	isoleucyl-tRNA synthetase, putative	Tb10.6k15.1220	0.	29.1.5 protein.aa activation.	0
LmjF29.0510	cofilin-like protein			31.1 cell.organisation	0
LmjF34.3500	ruvb-like 1 DNA helicase, putative	Tb927.4.1270	5.4e-198	28.2 DNA.repair	0
LmjF33.2740	translation initiation factor if-2, putative	Tb927.2.3780	1.1e-256	29.2.2 protein.synthesis.misc ribososomal protein	0
LmjF24.2210	60S ribosomal protein L12, putative	Tb09.211.4550	5.2e-58	29.2.2 protein.synthesis.misc ribososomal protein	0
LmjF36.6910	chaperonin, putative,T-complex protein 1 (theta subunit), putative	Tb10.6k15.2330	1.7e-230	29.6 protein.(un)folding	0
LmjF01.0770	eukaryotic initiation factor 4a, putative	Tb09.160.3270	1.2e-183	29.2.2 protein.synthesis.misc ribososomal protein	0
LmjF36.1960	phosphomannomutase, putative	Tb10.70.0370	6.4e-93	10.1.21 cell wall.precursor synthesis.	0
LmjF34.2610	ATP-dependent DNA helicase, putative,	Tb927.4.2000	4.7e-204	28.2 DNA.repair	0
LmjF29.1240	hypothetical protein, unknown function			35.1 not assigned.no ontology	0
LmjF30.0460	aspartyl-tRNA synthetase, putative	Tb927.6.1880	1.0e-198	29.1.5 protein.aa activation.	0
LmjF32.2950	nucleoside diphosphate kinase b	Tb11.01.7800	2.6e-64	23.4.10 nucleotide metabolism.	0
LmjF34.1520	hypothetical protein, conserved	Tb927.4.2740	2.2e-51	35.1 not assigned.no ontology	0
LmjF12.0530	glucose-6-phosphate isomerase	Tb927.1.3830	2.1e-226	4.3 glycolysis.G6PIsomerase	0
LmjF09.0100	hypothetical protein, conserved,calmodulin-like protein	Tb11.01.3960	1.2e-138	35.1 not assigned.no ontology	0
LmjF33.1380	mitogen activated protein kinase, putative,map kinase, putative	Tb10.389.1730	9.5e-152	30.6 signalling.MAP kinases	0
LmjF22.0470	hypothetical protein, conserved	Tb927.7.2400	1.5e-55	35.1 not assigned.no ontology	0
LmjF28.1730	proteasome regulatory non-ATP-ase subunit 2, putative	Tb11.01.0960	7.4e-263	29.5.11.20 protein.degradation.ubiquitin.proteasom	0
LmjF13.0570	40S ribosomal protein S12, putative			29.2.2 protein.synthesis.misc ribososomal protein	0
LmjF32.0630	hypothetical protein, conserved			35.1 not assigned.no ontology	0

LmjF22.0300	hypothetical protein, conserved	Tb927.7.2240	1.0e-90	35.1 not assigned.no ontology	0
LmjF36.0180	elongation factor 2	Tb10.70.2650	0.	29.2.2 protein.synthesis.misc ribososomal protein	0
LmjF34.1040	uracil phosphoribosyltransferase, putative	Tb927.4.3320	3.3e-73	23.3.1.3 nucleotide metabolism.	0
LmjF13.1580	ubiquitin-conjugating enzyme-like protein			29.5.11.20 protein.degradation.ubiquitin.	0
LmjF09.0770	oligopeptidase b,serine peptidase, clan SC, family S9A-like protein	Tb11.52.0003	2.5e-250	29.5 protein.degradation	0
LmjF29.2340	hypothetical protein, conserved			35.1 not assigned.no ontology	0
LmjF34.2820	regulatory subunit of protein kinase a-like protein			29.4 protein.postranslational modification	0
LmjF32.1820	iron superoxide dismutase, putative	Tb11.01.7550	3.4e-76	21.6 redox.dismutases and catalases	0
LmjF07.0870	splicing factor ptsr1-like protein			27.1.1 RNA.processing.splicing	0
LmjF28.2740	activated protein kinase c receptor (LACK),	Tb11.01.3180	1.6e-110	29.4 protein.postranslational modification	0
LmjF35.3230	cystathione gamma lyase, putative	Tb09.211.3330	1.4e-176	13.1 amino acid metabolism.synthesis	0
LmjF27.0760	small GTP-binding protein Rab1, putative	Tb927.8.890	2.7e-81	30.5 signalling.G-proteins	0
LmjF25.0750	protein phosphatase, putative	Tb11.03.0390	1.4e-143	29.4 protein.postranslational modification	0
LmjF25.1610	hypothetical protein, conserved			35.1 not assigned.no ontology	0
LmjF18.1370	heat shock protein, putative	Tb10.389.0880	3.1e-217	29.6 protein.(un)folding	0
LmjF35.0820	aspartate aminotransferase, putative	Tb10.70.3710	4.2e-112	13.1.1.2.1 amino acid metabolism.	0
LmjF36.1370	Transitional endoplasmic reticulum ATPase, ,VCP homolog	Tb10.70.1190	0.	29.3 protein.targeting	0
LmjF33.1750	macrophage migration inhibitory factor-like protein			35.1 not assigned.no ontology	0
LmjF28.2860	cytosolic malate dehydrogenase, putative	Tb11.01.3040	1.3e-106	6.3 gluconeogenesis.Malate DH	0
LmjF36.5150	hypothetical protein, conserved	Tb11.01.2530	2.6e-123	35.1 not assigned.no ontology	0
LmjF24.2060	transketolase, putative	Tb927.8.6170	1.8e-237	7.2.1 OPP.non-reductive PP.transketolase	0
LmjF36.0740	hypothetical protein, conserved			35.1 not assigned.no ontology	0
LmjF33.2550	isocitrate dehydrogenase, putative			8.1.4 TCA / org. transformation.TCA.IDH	0
LmjF15.1450	proliferative cell nuclear antigen (PCNA), putative	Tb09.160.3710	2.7e-105	31.3 cell.cycle	0
LmjF25.2020	hypothetical protein, conserved	Tb927.3.2100	8.4e-68	35.1 not assigned.no ontology	0
LmjF36.0070	stress-inducible protein STI1 homolog	Tb10.v4.0035	1.7e-81	35.2 not assigned.unknown	0
LmjF13.0090	carboxypeptidase, putative,metallo-peptidase, family 32	Tb11.02.0100	5.5e-135	29.5 protein.degradation	0
LmjF09.0970	elongation factor-1 gamma	Tb11.01.4750	3.3e-144	29.2.2 protein.synthesis.misc ribososomal protein	0
LmjF36.3780	hypothetical protein, conserved	Tb11.01.1460	2.6e-108	35.1 not assigned.no ontology	0
LmjF28.2770	heat-shock protein hsp70, putative	Tb11.01.3110	1.8e-283	29.6 protein.(un)folding	0
LmjF35.3860	t-complex protein 1, eta subunit, putative	Tb09.211.2570	1.5e-227	29.6 protein.(un)folding	0
LmjF36.1430	translation elongation factor 1-beta, putative	Tb10.70.1100	1.5e-64	29.2.2 protein.synthesis.misc ribososomal protein	0
LmjF26.1570	thimet oligopeptidase, putative,metallo-peptidase, Family M3	Tb927.7.190	2.4e-220	29.5 protein.degradation	0
LmjF30.3090	hypothetical protein, conserved			35.1 not assigned.no ontology	0
LmjF32.1730	coatomer epsilon subunit, putative	Tb11.01.6530	2.2e-58	31.4 cell. vesicle transport	0
LmjF35.1010	casein kinase, putative	Tb927.5.800	4.0e-151	29.4 protein.postranslational modification	0

LmjF05.0960	dipeptidyl-peptidase III, putative,metallo-peptidase, Family M49			29.5 protein.degradation	0
LmjF35.0400	40S ribosomal protein S3a, putative	Tb10.70.3360	6.5e-89	29.2.2 protein.synthesis.misc ribososomal protein	0
LmjF12.0250	cysteinyl-tRNA synthetase, putative	Tb927.6.950	5.8e-277	29.1.40 protein.aa activation.	0
LmjF14.1320	serine hydroxymethyltransferase, putative			13.1.5.2.1 amino acid metabolism.synthesis.	0
LmjF21.1090	t-complex protein 1, delta subunit, putative	Tb10.70.7050	2.8e-239	29.6 protein.(un)folding	0
LmjF25.0720	eukaryotic initiation factor 5a, putative	Tb11.03.0410	3.7e-59	29.2.2 protein.synthesis.misc ribososomal protein	0
LmjF15.1190	hypothetical protein, conserved			35.1 not assigned.no ontology	0
LmjF14.1160	enolase	Tb10.70.4740	6.4e-183	4.12 glycolysis.enolase	0
LmjF19.0160	aminopeptidase, putative,metallo-peptidase, Clan MG, Family M24	Tb10.61.1870	6.4e-143	29.5 protein.degradation	0
LmjF35.0370	ATP-dependent DEAD-box RNA helicase, putative	Tb10.70.3290	1.6e-188	27.1.2 RNA.processing.RNA helicase	0
LmjF36.2030	chaperonin Hsp60, mitochondrial precursor	Tb10.70.0430	8.8e-216	29.6 protein.(un)folding	0
LmjF21.1830	proteasome alpha 5 subunit, putative,	Tb10.100.0120	1.7e-92	29.5.11.20 protein.degradation.ubiquitin.	0
LmjF28.2310	eukaryotic translation initiation factor, putative	Tb11.01.3420	7.2e-99	29.2.2 protein.synthesis.misc ribososomal protein	0
LmjF23.1220	t-complex protein 1, gamma subunit, putative	Tb927.8.3150	1.7e-237	29.6 protein.(un)folding	0
LmjF16.1425	paraflagellar rod protein 2C	Tb927.8.5010	9.7e-245	31.1 cell.organisation	0
LmjF35.1220	short chain dehydrogenase, putative			35.1 not assigned.no ontology	0
LmjF05.0350	trypanothione reductase	Tb10.406.0520	6.3e-175	21.2 redox.ascorbate and glutathione	0
LmjF36.2020	chaperonin Hsp60, mitochondrial precursor	Tb10.70.0430	1.1e-208	29.6 protein.(un)folding	0
LmjF36.1630	clathrin heavy chain, putative	Tb10.70.0830	0.	31.4 cell. vesicle transport	0
LmjF23.1580	hypothetical protein, conserved			35.1 not assigned.no ontology	0
LmjF03.0690	hypothetical protein, conserved	Tb927.3.2600	0.	35.1 not assigned.no ontology	0
LmjF04.0470	60S ribosomal protein L11 (L5, L16)	Tb09.160.5580	2.8e-74	29.2.2 protein.synthesis.misc ribososomal protein	0
LmjF21.0760	proteasome regulatory non-ATP-ase subunit 5, putative,	Tb10.70.6360	3.2e-150	29.5.11.20 protein.degradation.ubiquitin.	0
LmjF23.0110	mannose-1-phosphate guanyltransferase	Tb927.8.2050	5.2e-104	23 nucleotide metabolism	0
LmjF14.0850	calpain-like cysteine peptidase, putative,Clan CA, family C2			29.5 protein.degradation	0
LmjF17.1290	translation initiation factor, putative	Tb927.5.2570	1.1e-164	29.2.2 protein.synthesis.misc ribososomal protein	0
LmjF16.0540	aspartate carbamoyltransferase, putative	Tb927.5.3820	1.6e-108	23.1 nucleotide metabolism.synthesis	0
LmjF35.1410	threonyl-tRNA synthetase, putative	Tb927.5.1090	0.	29.1.40 protein.aa activation.	0
LmjF30.2740	TPR domain protein, conserved	Tb927.6.4000	1.5e-119	35.1 not assigned.no ontology	0
LmjF23.0270	pteridine reductase 1	Tb927.8.2210	4.5e-50	18 Co-factor and vitamine metabolism	0
LmjF32.0080	N-myristoyl transferase, putative	Tb10.61.2550	3.4e-132	29.4 protein.postranslational modification	0
LmjF11.0820	hypothetical protein, conserved			35.1 not assigned.no ontology	0
LmjF03.0520	hypothetical protein, conserved	Tb10.70.3950	8.7e-89	35.1 not assigned.no ontology	0
LmjF36.6650	2,3-bisphosphoglycerate-independent phosphoglycerate mutase	Tb10.6k15.2620	3.7e-242	4.11 glycolysis.phosphoglycerate mutase	0
LmjF23.1480	hypothetical protein, conserved	Tb927.5.1280	3.7e-86	35.1 not assigned.no ontology	0
LmjF10.0070	ribosomal protein l35a, putative	Tb927.4.2180	3.1e-61	29.2.2 protein.synthesis.misc ribososomal protein	0

LmjF27.2000	hypothetical protein, conserved	Tb927.2.4580	1.1e-58	35.1 not assigned.no ontology	0
LmjF26.0810	glutathione peroxidase-like protein, putative	Tb927.7.1130	9.0e-63	21.5 redox.peroxiredoxins	0
LmjF27.0190	proteasome alpha 7 subunit, putative	Tb927.3.780	1.1e-104	29.5.11.20 protein.degradation.ubiquitin.	0
LmjF31.1070	biotin/lipoate protein ligase-like protein			29.4 protein.postranslational modification	0
LmjF35.3100	ATP-dependent RNA helicase, putative	Tb09.211.3510	4.1e-177	27.1.2 RNA.processing.RNA helicase	0
LmjF28.1820	replication factor A, 51kDa subunit, putative	Tb11.01.0870	1.3e-162	28.1 DNA.synthesis/chromatin structure	0
LmjF21.0540	la RNA binding protein, putative	Tb10.70.5360	2.2e-102	27.4 RNA.RNA binding	0
LmjF35.2200	RNA-binding protein, putative	Tb09.211.4540	7.6e-83	27.4 RNA.RNA binding	0
LmjF24.1500	IgE-dependent histamine-releasing factor, putative	Tb927.8.6760	7.3e-68	35.2 not assigned.unknown	0
LmjF26.1380	prefoldin-like protein			29.6 protein.(un)folding	0
LmjF26.1240	heat shock protein 70-related protein	Tb927.7.710	8.6e-265	29.6 protein.(un)folding	0
LmjF26.2280	nitrilase, putative	Tb09.160.0770	2.0e-99	22.1.5 polyamine metabolism.	0
LmjF31.1890	peptidase m20/m25/m40 family-like protein	Tb927.6.400	2.8e-160	29.5 protein.degradation	0
LmjF06.0140	20S proteasome beta 6 subunit, putative	Tb927.7.4790	7.7e-93	29.5.11.20 protein.degradation.ubiquitin.	0
LmjF21.1760	centromere/microtubule binding protein cbf5, putative	Tb10.100.0060	6.3e-208	31.1 cell.organisation	0
LmjF18.0700	hypothetical protein, conserved	Tb10.05.0170	9.8e-181	35.1 not assigned.no ontology	0
LmjF11.1170	eukaryotic release factor 3, putative	Tb11.02.4030	6.3e-212	29.2.2 protein.synthesis.misc ribososomal protein	0
LmjF27.1310	arginyl-tRNA synthetase, putative	Tb11.46.0008	2.7e-248	29.1.19 protein.aa activation.	0
LmjF21.0800	hypothetical protein, conserved	Tb10.70.6450	0.	35.1 not assigned.no ontology	0
LmjF34.0650	proteasome regulatory non-ATP-ase subunit 11, putative,	Tb10.70.4570	2.7e-110	29.5.11.20 protein.degradation.ubiquitin.	0
LmjF35.0750	proteasome activator protein pa26, putative			29.5.11.20 protein.degradation.ubiquitin.	0
LmjF21.0430	hypothetical protein, conserved			35.1 not assigned.no ontology	0
LmjF35.2350	aminopeptidase P, putative,metallo-peptidase, Family M24	Tb09.211.4330	5.9e-172	29.5 protein.degradation	0
LmjF02.0370	proteasome regulatory non-ATPase subunit 6, putative	Tb927.2.2440	1.1e-127	29.5.11.20 protein.degradation.ubiquitin.	0
LmjF36.5100	hypothetical protein, conserved			35.1 not assigned.no ontology	0
LmjF04.0960	adenylate kinase, putative	Tb09.211.0350	1.0e-58	23.4.1 nucleotide metabolism.	0
LmjF22.1540	alanyl-tRNA synthetase, putative	Tb927.6.700	0.	29.1.40 protein.aa activation.	0
LmjF06.0370	glutamine synthetase, putative	Tb927.7.4970	1.9e-144	12.2.2 N-metabolism.ammonia metabolism.	0
LmjF31.2150	prostaglandin f2-alpha synthase	Tb11.02.2310	2.5e-90	30.99 signalling.unspecified	0
LmjF34.2580	hypothetical protein, conserved			35.1 not assigned.no ontology	0
LmjF27.1260	T-complex protein 1, beta subunit, putative	Tb11.42.0003	1.2e-229	29.6 protein.(un)folding	0
LmjF13.0450	hypothetical protein, conserved			35.1 not assigned.no ontology	0
LmjF07.0640	hypothetical protein, conserved			35.1 not assigned.no ontology	0
LmjF30.3240	glutamyl-tRNA synthetase, putative	Tb927.6.4590	5.0e-216	29.1.40 protein.aa activation.	0
LmjF13.1220	40S ribosomal protein S4, putative	Tb11.02.1085	7.3e-109	29.2.2 protein.synthesis.misc ribososomal protein	0
LmjF21.0810	methionyl-tRNA synthetase, putative	Tb10.70.6470	7.0e-257	29.1.40 protein.aa activation.	0

LmjF36.3210	14-3-3 protein-like protein	Tb11.01.1290	1.5e-96	30.7 signalling.14-3-3 proteins	0
LmjF17.0010	hypothetical protein, conserved	Tb927.7.6090	1.6e-70	35.1 not assigned.no ontology	0
LmjF33.2540	carboxypeptidase, putative,metallo-peptidase, Family M32	Tb11.02.0100	2.6e-151	29.5 protein.degradation	0
LmjF36.3880	eukaryotic translation initiation factor 3 subunit, putative	Tb11.01.1370	3.0e-89	29.2.2 protein.synthesis.misc ribososomal protein	0
LmjF10.0490	mitogen-activated protein kinase 3, putative,map kinase 3, putative	Tb927.8.3550	4.1e-143	30.6 signalling.MAP kinases	0
LmjF25.0910	cyclophilin a	Tb11.03.0250	1.8e-69	29.6 protein.(un)folding	0
LmjF08.1110	stress-induced protein sti1	Tb927.5.2940	7.1e-200	35.1 not assigned.no ontology	0
LmjF34.4600	hypothetical protein, conserved	Tb927.4.360	5.2e-74	35.1 not assigned.no ontology	0
LmjF35.5260	hypothetical protein, conserved			35.1 not assigned.no ontology	0
LmjF33.2570	aminopeptidase, putative,metallo-peptidase, Clan MF, Family M17	Tb11.02.0070	4.1e-153	29.5 protein.degradation	0
LmjF32.0840	hypothetical protein, conserved	Tb11.01.5680	3.7e-91	35.1 not assigned.no ontology	0
LmjF32.0950	hypothetical protein, conserved	Tb11.01.5780	1.7e-228	35.1 not assigned.no ontology	0
LmjF14.0190	hypothetical protein, conserved	Tb927.7.4520	1.2e-62	35.1 not assigned.no ontology	0
LmjF30.3130	valyl-tRNA synthetase, putative	Tb927.6.4480	0.	29.1 protein.aa activation	0
LmjF26.2700	6-phosphogluconolactonase	Tb11.02.4200	6.2e-50	7.1.2 OPP.oxidative PP.	0
LmjF11.0350	14-3-3 protein, putative	Tb11.02.4700	3.1e-98	30.7 signaling	0
LmjF31.2790	adp-ribosylation factor, putative	Tb09.211.4490	3.9e-81	23 nucleotide metabolism	0
LmjF21.1700	proteasome alpha 2 subunit, putative	Tb10.100.0170	6.3e-90	29.5.11.20 protein.degradation.ubiquitin.proteasom	0
LmjF18.0280	hypothetical protein, conserved	Tb10.61.3130	1.8e-68	35.1 not assigned.no ontology	0
LmjF15.1440	glutaminyl-tRNA synthetase, putative	Tb09.160.3730	4.4e-238	29.1 protein.aa activation	0
LmjF36.1600	proteasome alpha 1 subunit, putative	Tb10.70.0850	1.0e-104	29.5.11.20 protein.degradation.ubiquitin.proteasom	0
LmjF36.3910	S-adenosylhomocysteine hydrolase	Tb11.01.1350	3.0e-208	13.1.3.4 amino acid metabolism.synthesis.	0
LmjF04.0750	60S ribosomal protein L10, putative	Tb09.211.0340	1.7e-100	29.2.4 protein.synthesis.elongation	0
LmjF30.3040	eukaryotic translation initiation factor 3 subunit 7-like protein	Tb927.6.4370	6.5e-74	29.2.4 protein.synthesis.elongation	0
LmjF23.0360	NADP-dependent alcohol dehydrogenase, putative			26.11 misc.alcohol dehydrogenases	0
LmjF17.0735	lysine decarboxylase-like protein			13.2.3.5 amino acid metabolism.	0
LmjF35.1880	60S ribosomal protein L5, putative	Tb09.244.2730	1.1e-111	29.2.4 protein.synthesis.elongation	0
LmjF35.4850	proteasome alpha 1 subunit, putative	Tb09.211.1250	3.7e-88	29.5.11.20 protein.degradation.ubiquitin.proteasom	0
LmjF28.2170	hypothetical protein, conserved	Tb11.01.3290	1.4e-136	35.1 not assigned.no ontology	0
LmjF27.1300	hypothetical protein, conserved	Tb11.46.0009	2.8e-122	35.1 not assigned.no ontology	0
LmjF20.0240	hypothetical protein, conserved			35.1 not assigned.no ontology	1
LmjF36.3590	cysteine synthase, putative			13.1.5.3.1 amino acid metabolism.	0
LmjF13.0560	60S ribosomal protein L18, putative	Tb10.6k15.0410	2.0e-66	29.2.4 protein.synthesis.elongation	0
LmjF32.0230	dynein light chain, flagellar outer arm, putative			31.1 cell.organisation	0
LmjF30.3500	S-adenosylmethionine synthetase,	Tb927.6.4850	5.5e-158	13.1.3.4.11 amino acid metabolism.	0

LmjF08.0290	iron superoxide dismutase	Tb927.5.3350	4.1e-84	21.6 redox.dismutases and catalases	0
LmjF07.0340	ATP-dependent DEAD/H RNA helicase, putative	Tb927.8.1510	1.1e-160	28.1 DNA.synthesis/chromatin structure	0
LmjF30.2600	c-1-tetrahydrofolate synthase, cytoplasmic, putative			?	0
LmjF10.0290	isocitrate dehydrogenase [NADP], mitochondrial precursor,	Tb927.8.3690	1.5e-179	8.1.4 TCA / org. transformation.TCA.IDH	0
LmjF33.2270	hypothetical protein, conserved			35.1 not assigned.no ontology	0
LmjF08.0550	translation initiation factor-like protein	Tb927.5.3120	5.8e-60	29.2.4 protein.synthesis.elongation	0
LmjF06.0120	cyclophilin	Tb927.7.4770	2.8e-57	29.6 protein.(un)folding	0
LmjF20.1280	calpain-like cysteine peptidase, Clan CA, family C2			29.5 protein.degradation	0
LmjF01.0410	ribosomal protein S7, putative	Tb09.160.2550	2.4e-75	29.2.4 protein.synthesis.elongation	0
LmjF33.0312	heat shock protein 83-1	Tb10.26.1080	4.8e-286	29.6 protein.(un)folding	0
LmjF32.1000	chaperonin containing t-complex protein, putative	Tb11.01.5860	1.3e-239	29.6 protein.(un)folding	0
LmjF15.0200	60S ribosomal protein L13a, putative	Tb927.4.3550	8.3e-94	29.2.4 protein.synthesis.elongation	0
LmjF34.3670	vacuolar ATP synthase catalytic subunit a, putative	Tb927.4.1080	1.3e-286	34.1 transport.p- and v-ATPases	0
LmjF24.1800	hypothetical protein, conserved			35.1 not assigned.no ontology	0
LmjF28.2910	glutamate dehydrogenase, putative			12.3.1 N-metabolism.N-degradation.	0
LmjF15.0230	lysyl-tRNA synthetase, putative	Tb927.8.1600	1.9e-227	29.1 protein.aa activation	0
LmjF36.4650	60S acidic ribosomal protein, putative	Tb10.6k15.0270	1.5e-83	29.2.4 protein.synthesis.elongation	0
LmjF09.1010	hypothetical protein, conserved	Tb11.01.4740	6.2e-133	35.1 not assigned.no ontology	1
LmjF30.0880	adenosine kinase, putative	Tb927.6.2300	8.3e-101	23.3.2.1 nucleotide metabolism.	0
LmjF21.1060	40S ribosomal protein S23, putative	Tb10.70.7020	2.5e-70	29.2.4 protein.synthesis.elongation	0
LmjF32.1200	proteasome regulatory non-ATP-ase subunit, putative	Tb11.01.6030	2.1e-72	29.5.11.20 protein.degradation.ubiquitin.proteasom	0
LmjF05.0830	methylthioadenosine phosphorylase, putative	Tb927.7.7040	2.9e-97	23 nucleotide metabolism	0
LmjF36.5010	40S ribosomal protein SA, putative	Tb11.01.2680	6.6e-99	29.2.4 protein.synthesis.elongation	0
LmjF31.0010	homocyst ei nemethyltransferase, putative	Tb927.8.2610	7.0e-233	13.2.3.4 amino acid metabolism.	0
LmjF16.1310	cytochrome c, putative	Tb11.01.2680	6.6e-99	9.6 mitochondrial electron transport /	1
LmjF29.2510	6-phospho-1-fructokinase, putative	Tb927.3.3270	2.0e-180	4.4 glycolysis.PPFK	0
LmjF27.2400	heat shock protein DnaJ, putative	Tb927.2.5160	1.7e-138	29.6 protein.(un)folding	0
LmjF08.0890	mitochondrial DNA polymerase beta	Tb927.5.2780	1.1e-127	28.1 DNA.synthesis/chromatin structure	0
LmjF32.3900	60S ribosomal protein L2, putative	Tb927.5.1110	8.5e-126	29.2.4 protein.synthesis.elongation	0
LmjF21.1552	RNA helicase, putative	Tb10.70.7730	2.0e-193	27.1.2 RNA.processing.RNA helicase	0
LmjF32.0400	ATP-dependent RNA helicase, putative	Tb10.61.2130	5.7e-163	27.1.2 RNA.processing.RNA helicase	0
LmjF32.3270	chaperonin alpha subunit, putative	Tb11.01.8510	4.2e-192	29.6 protein.folding	0
LmjF34.4310	coatomer alpha subunit, putative			31.4 cell. vesicle transport	0
LmjF11.0100	seryl-tRNA synthetase, putative	Tb11.02.5020	3.6e-190	29.1 protein.aa activation	0
LmjF32.3130	ribosomal protein l3, putative	Tb927.4.1790	4.3e-194	29.2.4 protein.synthesis.elongation	0
LmjF36.2950	succinyl-CoA ligase [GDP-forming] beta-chain, putative	Tb10.6k15.3250	8.0e-150	8.1.6 TCA / org. transformation.	0

LmjF25.1420	GTP-binding protein, putative	Tb927.3.1120	7.8e-96	30.5 signalling.G-proteins	0
LmjF24.2110	hypothetical protein, conserved	Tb927.8.6110	3.4e-190	35.1 not assigned.no ontology	0
LmjF04.1230	actin	Tb09.211.0630	6.4e-184	31.1 cell.organisation	0
LmjF07.0680	40S ribosomal protein S9, putative	Tb10.70.1380	8.3e-84	29.2.4 protein.synthesis.elongation	0
LmjF14.0450	hypothetical protein, conserved	Tb927.7.4290	7.9e-81	35.1 not assigned.no ontology	0
LmjF22.1520	40S ribosomal protein L14, putative	Tb927.6.720	3.7e-58	29.2.4 protein.synthesis.elongation	0
LmjF11.1000	pyruvate phosphate dikinase, putative	Tb11.02.4150	0.	4.4 glycolysis.PPDK	0
LmjF35.1300	ubiquitin-conjugating enzyme E2, putative	Tb927.5.1000	1.0e-78	29.5.11.3 protein.degradation.ubiquitin.E2	0
LmjF16.0460	60S ribosomal protein l21, putative	Tb11.0880	3.2e-62	29.2.4 protein.synthesis.elongation	0
LmjF29.2200	GTP-binding protein, putative	Tb927.3.4720	1.9e-237	30.5 signalling.G-proteins	0
LmjF29.2460	60S ribosomal protein L13, putative	Tb927.3.3310	3.0e-83	29.2.4 protein.synthesis.elongation	0
LmjF36.0930	40S ribosomal protein S18, putative	Tb10.70.1740	5.9e-65	29.2.4 protein.synthesis.elongation	0
LmjF18.0510	aconitase, putative	Tb10.61.2880	0.	8.2.3 TCA / org. transformation.	0
LmjF31.1750	nucleosome assembly protein-like protein	Tb09.160.4240	4.1e-56	28.1 DNA.synthesis/chromatin structure	0
LmjF13.1480	Ran-binding protein 1, putative	Tb11.02.0870	6.7e-55	30.5 signalling.G-proteins	0
LmjF28.2430	vacuolar ATP synthase subunit B, putative	Tb11.01.3560	8.6e-242	34.1 transport.p- and v-ATPases	0
LmjF27.1380	60S acidic ribosomal subunit protein, putative	Tb11.46.0001	7.5e-104	29.2.4 protein.synthesis.elongation	0
LmjF15.0950	40S ribosomal protein S3, putative	Tb09.160.4450	3.0e-89	29.2.4 protein.synthesis.elongation	0
LmjF13.0870	mitochondrial processing peptidase alpha subunit,	Tb11.02.1480	1.1e-127	29.5 protein.degradation	0
LmjF07.0500	60S ribosomal protein L7a, putative	Tb927.8.1330	8.5e-90	29.2.4 protein.synthesis.elongation	0
LmjF25.1120	aldehyde dehydrogenase, mitochondrial precursor	Tb10.70.0630	1.3e-63	4 glycolysis	0
LmjF11.1110	60S ribosomal protein L28, putative			29.2.4 protein.synthesis.elongation	0
LmjF22.1460	i/6 autoantigen-like protein			31.1 cell.organisation	0
LmjF14.1440	hypothetical protein, conserved	Tb927.7.3550	3.7e-148	35.1 not assigned.no ontology	0
LmjF21.1555	hypothetical protein, conserved	Tb10.70.7760	6.8e-153	35.1 not assigned.no ontology	1
LmjF29.1750	paraflagellar rod protein 1D, putative	Tb927.3.4300	1.1e-247	31.6* cell organization-	0
LmjF36.6940	protein disulfide isomerase	Tb10.6k15.2290	4.7e-83	29.6 protein.(un)folding	0
LmjF10.0210	nucleolar protein, putative	Tb927.8.3750	2.0e-203	28.1 DNA.synthesis/chromatin structure	0
LmjF11.1190	40S ribosomal protein S15a, putative	Tb11.02.4000	1.9e-62	29.2.4 protein.synthesis.elongation	0
LmjF27.1805	glycosomal phosphoenolpyruvate carboxykinase, putative	Tb927.2.4210	2.2e-230	6.4 gluconeogenese/ glyoxylate cycle.PEPCK	0
LmjF26.0620	10 kDa heat shock protein, putative			29.6 protein.(un)folding	0
LmjF24.0640	MCAK-like kinesin, putative	Tb11.02.2970	1.8e-169	31.6* cell organization-	0
LmjF22.1370	intraflagellar transport protein-like protein	Tb927.7.3370	4.7e-135	31.4 cell. vesicle transport	0
LmjF35.4130	poly(a) binding protein, putative	Tb09.211.2150	8.9e-175	RNA.processing	0
LmjF18.0670	citrate synthase, putative	Tb10.05.0150	1.7e-151	6.1 gluconeogenese/ glyoxylate cycle.citrate synthase	0
LmjF26.0140	adenine phosphoribosyltransferase	Tb927.7.1780	4.3e-66	23 nucleotide metabolism	0

LmjF28.1200	glucose-regulated protein 78, putative	Tb11.02.5500	1.7e-259	29.6 protein.folding	1
LmjF36.2860	40S ribosomal protein S24e			29.2.4 protein.synthesis.elongation	0
LmjF27.0930	isovaleryl-coA dehydrogenase, putative	Tb11.55.0026	7.4e-145	13.2.4.4 amino acid metabolism.degradation.	0
LmjF26.0170	60S ribosomal protein L7, putative	Tb927.7.1750	8.6e-94	29.2.4 protein.synthesis.elongation	0
LmjF32.0880	60S ribosomal protein L18a, putative	Tb10.70.3510	1.7e-80	29.2.4 protein.synthesis.elongation	0
LmjF18.1380	pyruvate dehydrogenase E1 component alpha subunit, putative	Tb10.389.0890	5.3e-148	11.1.31 lipid metabolism.FA synthesis and FA elongation.	0
LmjF21.0030	hypothetical protein, conserved			35.1 not assigned.no ontology	0
LmjF33.2340	succinyl-coA:3-ketoacid-coenzyme A transferase,	Tb11.02.0290	8.8e-169	11.1.10 lipid metabolism.	0
LmjF19.0060	40S ribosomal protein S2	Tb10.61.2070	2.7e-104	29.2.4 protein.synthesis.elongation	0
LmjF24.2070	40S ribosomal protein S8, putative	Tb927.8.6150	1.2e-92	29.2.4 protein.synthesis.elongation	0
LmjF25.2130	succinyl-CoA synthetase alpha subunit, putative	Tb927.3.2230	1.9e-109	8.1.6 TCA / org. transformation.TCA.	0
LmjF32.2690	ribosomal protein L27, putative	Tb11.01.7535	1.0e-54	29.2.4 protein.synthesis.elongation	0
LmjF11.0960	40S ribosomal protein S5	Tb11.02.4170	2.7e-85	29.2.4 protein.synthesis.elongation	0
LmjF34.0110	adenylate kinase, putative	Tb10.70.5150	3.6e-73	23.4.1 nucleotide metabolism.	0
LmjF36.3990	hs1vu complex proteolytic subunit-like,	Tb11.01.2000	2.7e-81	29.5 protein.degradation	0
LmjF18.1510	P-type H+-ATPase, putative			34.1 transport.p- and v-ATPases	8
LmjF03.0200	delta-1-pyrroline-5-carboxylate dehydrogenase, putative	Tb10.70.4280	6.2e-215	?	0
LmjF06.1070	deoxyribose-phosphate aldolase, putative	Tb927.7.5680	5.3e-72	13.1.5.2.11 amino acid metabolism.	0
LmjF36.1260	fructose-1,6-bisphosphate aldolase	Tb10.70.1370	2.2e-158	4.8 glycolysis	0
LmjF29.1100	hypothetical protein, conserved	Tb927.3.5060	9.7e-91	35.1 not assigned.no ontology	0
LmjF29.0760	,heat shock protein 90, putative,glucose regulated protein 94,	Tb927.3.3580	7.8e-203	29.6 protein.folding	1
LmjF29.2860	40S ribosomal protein S19-like protein	Tb927.4.1860	4.2e-58	29.2.4 protein.synthesis.elongation	0
LmjF19.1400	hypothetical protein, conserved			35.1 not assigned.no ontology	0
LmjF32.3010	hypothetical protein, conserved,leucine rich repeat protein,	Tb11.01.8770	1.6e-281	35.1 not assigned.no ontology	0
LmjF35.1180	NADH-dependent fumarate reductase, putative	Tb927.5.940	3.0e-201	2.2.1.4 major CHO metabolism.	0
LmjF04.0760	nascent polypeptide associated complex subunit-like protein,			29.2.4 protein.synthesis.elongation	0
LmjF28.2555	40S ribosomal protein S17, putative			29.2.4 protein.synthesis.elongation	0
LmjF35.0030	pyruvate kinase, putative	Tb10.61.2680	1.5e-198	4.13 glycolysis.PK	0
LmjF32.3310	dihydrolipoamide dehydrogenase, putative	Tb11.01.8470	3.8e-195	8.1.1.3 TCA / org. transformation.TCA.pyruvate DH.E3	0
LmjF22.0730	hypothetical protein, conserved			35.1 not assigned.no ontology	0
LmjF35.3840	proteasome beta 2 subunit, putative	Tb09.211.2590	3.0e-107	29.5.11.20 protein.degradation.ubiquitin.proteasom	0
LmjF10.0910	small GTP-binding protein Rab11, putative,	Tb927.8.4330	1.5e-85	30.5 signalling.G-proteins	0
LmjF28.1010	ribosomal protein s20, putative			29.2.4 protein.synthesis.elongation	0
LmjF15.1010	glutamate dehydrogenase	Tb09.160.4310	3.3e-299	12.3.1 N-metabolism.N-degradation.	0
LmjF21.0730	60S ribosomal protein l36, putative			29.2.4 protein.synthesis.elongation	0
LmjF26.1960	hypothetical protein, conserved	Tb09.160.1160	8.9e-54	35.1 not assigned.no ontology	0

LmjF27.1870	trypanothione synthetase, putative	Tb927.2.4370	1.7e-213	21.2 redox.ascorbate and glutathione	0
LmjF17.0080	elongation factor 1-alpha	Tb10.70.5670	4.7e-224	29.2.4 protein.synthesis.elongation	0
LmjF02.0460	hypothetical protein, conserved	Tb927.2.2510	1.9e-53	35.1 not assigned.no ontology	0
LmjF09.1120	mitochondrial RNA binding protein 2,MRP2, gBP25	Tb11.01.4860	6.6e-49	27.4 RNA.RNA binding	0
LmjF19.0710	glycosomal malate dehydrogenase	Tb10.61.0980	3.5e-122	6.3 gluconeogenesis.Malate DH	0
LmjF35.2210	kinetoplastid membrane protein-11			31.1 cell.organisation	0
LmjF23.0040	peroxidoxin,tryparedoxin peroxidase	Tb927.8.1990	5.2e-88	21.5 redox.peroxiredoxins	0
LmjF27.0510	calpain-like cysteine peptidase, putative, Clan CA, family C2,	Tb11.v4.0001	1.2e-282	29.5 protein.degradation	0
LmjF21.1770	ATP synthase F1 subunit gamma protein, putative	Tb10.100.0070	5.1e-109	34.1 transport.p- and v-ATPases	0
LmjF26.2000	hypothetical protein, conserved	Tb09.160.1070	1.7e-70	35.1 not assigned.no ontology	1
LmjF35.3060	ubiquitin-activating enzyme E1, putative	Tb927.8.2640	2.7e-91	29.5.11.3 protein.degradation.ubiquitin	0
LmjF21.1050	60S ribosomal protein L9, putative	Tb10.70.7010	4.0e-68	29.2.4 protein.synthesis.elongation	0
LmjF23.0560	kinesin, putative	Tb927.8.2630	5.1e-193	31.6* cell organization-	0
LmjF29.2300	ubiquitin hydrolase, putative,cysteine peptidase, family C19,	Tb927.3.4840	2.1e-182	29.5 protein.degradation	0
LmjF24.0850	triosephosphate isomerase	Tb11.02.3210	6.9e-94	4.8 glycolysis	0
LmjF35.4770	peptidyl-prolyl cis-trans isomerase (cyclophilin-40), putative	Tb09.211.1350	1.5e-106	29.6 protein.(un)folding	0
LmjF30.2470	heat shock 70-related protein 1, mitochondrial precursor, putative	Tb927.6.3740	2.6e-277	29.6 protein.folding	0
LmjF05.0030	small GTP-binding protein, putative	Tb05.5K5.150	4.7e-81	30.5 signalling.G-proteins	0
LmjF21.0845	hypoxanthine-guanine phosphoribosyltransferase			23.1.2 nucleotide metabolism.synthesis.purine	0
LmjF15.1000	60S ribosomal protein L6, putative			29.2.4 protein.synthesis.elongation	0
LmjF13.0880	hypothetical protein, conserved	Tb11.02.1470	2.8e-59	35.1 not assigned.no ontology	0
LmjF33.0792	beta-tubulin	Tb927.1.2330	9.8e-217	31.1 cell.organisation	0
LmjF29.0870	hypothetical protein, conserved			35.1 not assigned.no ontology	0
LmjF33.2390	heat shock protein, putative	Tb11.02.0250	9.0e-299	29.6 protein.(un)folding	0
LmjF36.3750	40S ribosomal protein S27-1, putative			29.2.4 protein.synthesis.elongation	0
LmjF04.0460	adenylosuccinate lyase, putative	Tb09.160.5560	4.1e-175	23.1.2 nucleotide metabolism.synthesis.purine	0
LmjF23.0690	3-ketoacyl-coa thiolase-like protein	Tb927.8.2540	4.0e-165	11.1.10 lipid metabolism.	0
LmjF13.0280	alpha tubulin	Tb927.1.2380	1.2e-227	31.1 cell.organisation	0
LmjF21.1860	beta tubulin	Tb927.1.2330	5.2e-218	31.1 cell.organisation	0
LmjF35.3760	60S ribosomal protein L27A/L29, putative	Tb09.v1.0640	1.8e-60	29.2.4 protein.synthesis.elongation	0
LmjF21.0240	hexokinase, putative	Tb10.70.5820	4.0e-152	2.2.1.4 major CHO metabolism.	0
LmjF02.0710	ATP-dependent Clp protease subunit,	Tb927.2.3030	5.4e-295	29.5 protein.degradation	0
LmjF35.3280	60S ribosomal subunit protein L31, putative	Tb09.211.3280	9.6e-60	29.2.4 protein.synthesis.elongation	0
LmjF30.2970	glyceraldehyde 3-phosphate dehydrogenase, glycosomal	Tb927.6.4280	7.8e-158	4 glycolysis	0
LmjF16.0520	hypothetical protein, conserved	Tb927.8.5640	3.7e-77	35.1 not assigned.no ontology	0
LmjF35.1380	mitochondrial processing peptidase,	Tb927.5.1060	3.3e-201	29.5 protein.degradation	0

LmjF27.1110	mitochondrial RNA binding protein 1,gBP21, MRP1	Tb11.55.0009	6.3e-50	27.4 RNA.RNA binding	0
LmjF12.0670	cytochrome c oxidase subunit iv	Tb927.1.4100	7.3e-132	9.7 mitochondrial electron transport /	0
LmjF32.0750	RNA binding protein, putative	Tb11.01.5590	4.3e-63	27.4 RNA.RNA binding	0
LmjF36.1070	ribosomal protein L24, putative			29.2.4 protein.synthesis.elongation	0
LmjF26.0660	protein disulfide isomerase, putative	Tb927.7.1300	1.9e-79	29.6 protein.(un)folding	1
LmjF12.0210	proteasome regulatory ATPase subunittcc1l8.3, putative	Tb927.6.1090	1.3e-172	29.5.11.20 protein.degradation.ubiquitin.proteasom	0
LmjF25.1710	pyruvate dehydrogenase E1 beta subunit, putative	Tb927.3.1790	5.9e-139	8.1.1.1 TCA / org. transformation.TCA.pyruvate DH.E1	0
LmjF08.1100	hypothetical protein, conserved	Tb927.5.2930	1.2e-129	35.1 not assigned.no ontology	0
LmjF33.2610	mitochondrial processing peptidase alpha subunit, putative,	Tb927.2.4110	2.2e-144	29.5 protein.degradation	0
LmjF19.0200	ADP, ATP carrier protein 1, mitochondrial precursor, putative,ADP/ATP tr	Tb10.61.1820	8.9e-128	cell. transport	4
LmjF06.0570	60S ribosomal protein L23a, putative			29.2.4 protein.synthesis.elongation	0
LmjF30.2820	chaperonin HSP60/CNP60, putative	Tb927.6.4090	1.3e-182	29.6 protein.(un)folding	0
LmjF36.2380	sterol 24-c-methyltransferase, putative	Tb10.v4.0247	3.6e-121	11.1.10 lipid metabolism.	0
LmjF23.1410	hypothetical protein, conserved	Tb927.8.3380	1.9e-79	35.1 not assigned.no ontology	0
LmjF30.1380	n-acyl-l-amino acid amidohydrolase, putative	Tb927.1.3000	1.5e-52	13.2.3.4 amino acid metabolism.	0
LmjF25.1170	ATPase beta subunit, putative	Tb927.3.1380	3.7e-223	34.1 transport.p- and v-ATPases	0
LmjF18.1400	60S ribosomal protein L34, putative			29.2.4 protein.synthesis.elongation	0
LmjF15.0280	ribonucleoprotein p18, mitochondrial precursor, putative	Tb927.5.1710	7.7e-74	27.1 RNA.processing	0
LmjF19.0320	intraflagellar transport protein component, putative	Tb10.61.1590	2.7e-188	31.4 cell. vesicle transport	0
LmjF26.0880	40S ribosomal protein S16, putative	Tb927.7.1050	2.3e-56	29.2.4 protein.synthesis.elongation	0
LmjF31.1220	vacuolar-type proton translocating pyrophosphatase 1, putative			23.4.99 nucleotide metabolism.phosphotransfer	15
LmjF33.1610	peptidase M20/M25/M40, putative	Tb927.6.400	8.9e-171	29.5 protein.degradation	0
LmjF09.1340	histone H2B			28.1.3 DNA.synthesis/chromatin structure.histone	0
LmjF25.0490	RNA-binding protein, putative, UPB1			27.4 RNA.RNA binding	0
LmjF10.0870	histone h3			28.1.3 DNA.synthesis/chromatin structure.histone	0
LmjF05.0500	ATPase alpha subunit	Tb927.7.7420	2.3e-270	34.1 transport.p- and v-ATPases	0
LmjF36.6760	hypothetical protein, conserved	Tb10.6k15.2510	6.4e-82	35.1 not assigned.no ontology	0
LmjF23.0760	mitochondrial RNA binding protein, putative	Tb927.8.2740	2.4e-128	27.4 RNA.RNA binding	0
LmjF15.0010	histone h4			28.1.3 DNA.synthesis/chromatin structure.histone	0
LmjF35.3790	60S ribosomal protein L23, putative	Tb09.211.2640	3.4e-68	29.2.4 protein.synthesis.elongation	0
LmjF28.2420	2-oxoglutarate dehydrogenase, E2 component, dihydrolipoamide succinyl	Tb11.01.3550	4.1e-141	8.1 TCA / org. transformation.TCA	0
LmjF35.3700	Gim5A protein, putative,glycosomal membrane protein	Tb09.211.2730	9.0e-62	?	1
LmjF29.0720	hypothetical protein, conserved	Tb927.3.3630	3.1e-78	35.1 not assigned.no ontology	0
LmjF35.0070	prohibitin, putative	Tb10.v4.0045	3.0e-106	31.3 cell.cycle	1
LmjF26.1710	cytochrome c oxidase subunit V, putative	Tb09.160.1820	4.5e-90	9.7 mitochondrial electron transport /	0
LmjF19.0970	peptidyl-prolyl cis-trans isomerase,			29.6 protein.folding	1

LmjF35.0240	60S ribosomal protein L30			29.2.4 protein.synthesis.elongation	0
LmjF36.3470	2-oxoglutarate dehydrogenase E1 component, putative	Tb11.47.0004	3.1e-152	8.1 TCA / org. transformation.TCA	0
LmjF25.1190	ribosomal protein S25			29.2.4 protein.synthesis.elongation	0
LmjF30.3600	ATP synthase, epsilon chain, putative	Tb927.6.4990	3.7e-60	34.1 transport.p- and v-ATPases	0
LmjF31.2600	calreticulin, putative	Tb927.8.7410	1.6e-105	30.3 signalling.calcium	5
