# Supplementary material for: Exocytosis and protein secretion in Trypanosoma
Source: BMC Microbiol. 2010 Jan 26;10:20. doi: 10.1186/1471-2180-10-20 (PMC3224696; doi:10.1186/1471-2180-10-20)
Supplement: Additional file 4 — Table S4. Proteins identified in glycosome from T. brucei [19]. contains the list of 163 proteins from the glycosome proteome which were classified into functional categories (MapMan bins nomenclature). [file 1471-2180-10-20-S4.PDF]

Table S4
Glycosome proteome

Accession number	Protein name	MapMan bins
Tb927.6.1500	alkyl-dihydroxyacetone phosphate synthase,	11.3 lipid metabolism.Phospholipid synthesis
Tb927.3.1840	3-oxo-5-alpha-steroid 4-dehydrogenase, putative	11.1.13 lipid metabolism.FA synthesis and FA elongation.acyl-CoA binding protein
Tb927.4.2010	acyl-CoA binding protein, putative	11.1.13 lipid metabolism.FA synthesis and FA elongation.acyl-CoA binding protein
Tb09.211.3540	glycerol kinase, glycosomal	11.5 lipid metabolism.glycerol metabolism
Tb09.211.3550	glycerol kinase, glycosomal	11.5 lipid metabolism.glycerol metabolism
Tb09.211.3560	glycerol kinase, glycosomal	11.5 lipid metabolism.glycerol metabolism
Tb09.211.3570	glycerol kinase, glycosomal	11.5 lipid metabolism.glycerol metabolism
Tb11.02.5280	glycerol-3-phosphate dehydrogenase, putative	11.5.2 lipid metabolism.glyceral metabolism.Glycerol-3-phosphate dehydrogenase (NAD+)
Tb927.8.3530	glycerol-3-phosphate dehydrogenase [NAD+], glycosomal	11.5.2 lipid metabolism.glyceral metabolism.Glycerol-3-phosphate dehydrogenase (NAD+)
Tb927.8.6390	lysophospholipase, putative,alpha/beta hydrolase, putative	11.9.2 lipid metabolism.lipid degradation.lipases
Tb927.6.2790	L-threonine 3-dehydrogenase, putative	13.2 amino acid metabolism.degradation
Tb09.160.4560	arginine kinase	13.1.1 amino acid metabolism
Tb09.160.4570	arginine kinase	13.1.1 amino acid metabolism
Tb927.4.4070	mevalonate kinase, putative	16.1.2.4 secondary metabolism.isoprenoids.mevalonate pathway.mevalonate kinase
Tb927.5.930	NADH-dependent fumarate reductase	2 major CHO metabolism
Tb10.70.5800	hexokinase	2.2.1.4 major CHO metabolism.degradation.sucrose.hexokinase
Tb10.70.5820	hexokinase	2.2.1.4 major CHO metabolism.degradation.sucrose.hexokinase
Tb10.100.0130	peroxin 14, putative	21.2 redox.ascorbate and glutathione
Tb927.3.3780	tryparedoxin	21.2 redox.ascorbate and glutathione
Tb927.5.300	iron/ascorbate oxidoreductase family protein, putative	21.2 redox.ascorbate and glutathione
Tb927.7.1140	trypanothione/tryparedoxin dependent peroxidase 3,	21.2 redox.ascorbate and glutathione
Tb927.7.7500	iron/ascorbate oxidoreductase family protein, putative	21.2 redox.ascorbate and glutathione
Tb11.01.7550	iron superoxide dismutase	21.6 redox.dismutases and catalases
Tb927.5.3810	orotidine-5-phosphate decarboxylase	23.1.1.5 nucleotide metabolism.synthesis.pyrimidine.UMP Synthase
Tb10.6k15.3960	guanylate kinase, putative	23.1.2 nucleotide metabolism.synthesis.purine
Tb10.70.1200	adenylate kinase, putative	23.1.2 nucleotide metabolism.synthesis.purine
Tb10.70.6540	hypoxanthine-guanine phosphoribosyltransferase	23.1.2 nucleotide metabolism.synthesis.purine
Tb10.70.6660	hypoxanthine-guanine phosphoribosyltransferase, putative	23.1.2 nucleotide metabolism.synthesis.purine
Tb10.70.7330	adenylate kinase, putative	23.1.2 nucleotide metabolism.synthesis.purine
Tb927.2.5660	adenylate kinase, putative	23.1.2 nucleotide metabolism.synthesis.purine
Tb927.5.2080	inosine-5'-monophosphate dehydrogenase, putative	23.1.2 nucleotide metabolism.synthesis.purine

Tb927.7.1790	adenine phosphoribosyltransferase, putative	23.1.2 nucleotide metabolism.synthesis.purine
Tb927.7.5680	deoxyribose-phosphate aldolase, putative	23.1.2 nucleotide metabolism.synthesis.purine
Tb10.61.0150	inosine-5'-monophosphate dehydrogenase,	23.1.2.30 nucleotide metabolism.synthesis.purine.IMP dehydrogenase
Tb927.5.4350	NUDIX hydrolase, putative	23.5 nucleotide metabolism.deoxynucleotide metabolism
Tb10.406.0330	histone H2B, putative	28.1.3 DNA.synthesis/chromatin structure.histone
Tb10.406.0460	histone H2B, putative	28.1.3 DNA.synthesis/chromatin structure.histone
Tb927.5.4170	histone H4, putative	28.1.3 DNA.synthesis/chromatin structure.histone
Tb10.70.5650	elongation factor 1-alpha	29.2.4 protein.synthesis.elongation
Tb11.03.0410	eukaryotic translation initiation factor 5a, putative	29.2.4 protein.synthesis.elongation
Tb11.46.0001	60S acidic ribosomal subunit protein, putative	29.2.4 protein.synthesis.elongation
Tb10.70.0280	chaperonin Hsp60, mitochondrial precursor	29.6 protein.(un)folding
Tb11.01.3110	heat shock protein 70	29.6 protein.(un)folding
Tb11.01.6780	chaperone protein DNAJ, putative,heat shock protein-	29.6 protein.(un)folding
Tb11.02.5450	glucose-regulated protein 78, putative,	29.6 protein.(un)folding
Tb927.6.3800	heat shock 70 kDa protein, mitochondrial precursor, putative	29.6 protein.(un)folding
Tb11.01.6410	phosphomannose isomerase, putative	3 minor CHO metabolism
Tb927.8.7170	inositol polyphosphate 1-phosphatase, putative	3.4.1 minor CHO metabolism.myo-inositol.phosphatases
Tb11.03.0090	ribokinase, putative	3.5 minor CHO metabolism.others
Tb09.160.4460	TFIIF-stimulated CTD phosphatase, putative	30 signalling
Tb09.160.4480	TFIIF-stimulated CTD phosphatase, putative	30 signalling
Tb09.160.4520	calmodulin, putative	30.3 signalling.calcium
Tb11.01.4621	calmodulin	30.3 signalling.calcium
Tb09.211.1470	PACRGB,flagellar component	31.1 cell.organisation
Tb11.01.5100	paraflagellar rod component, putative	31.1 cell.organisation
Tb11.01.6740	paraflagellar rod protein	31.1 cell.organisation
Tb11.01.7750	dynein docking complex 2 (ODA1) protein, putative	31.1 cell.organisation
Tb11.47.0034	radial spoke protein RSP3, putative	31.1 cell.organisation
Tb11.50.0007	dynein light chain, putative,dynein light chain LC8, putative	31.1 cell.organisation
Tb927.1.2330	beta tubulin	31.1 cell.organisation
Tb927.1.2340	alpha tubulin	31.1 cell.organisation
Tb927.1.2670	axoneme central apparatus protein,flagellar protein PF16	31.1 cell.organisation
Tb927.3.2310	flagellar component,PACRGA	31.1 cell.organisation
Tb927.3.4290	73 kDa paraflagellar rod protein,PFR1	31.1 cell.organisation
Tb927.5.2850	radial spoke protein RSP2, putative	31.1 cell.organisation
Tb927.5.4480	paraflagellar rod component Par4, putative	31.1 cell.organisation
Tb927.8.4640	flagellar protofilament ribbon protein, putative	31.1 cell.organisation

Tb927.8.5010	69 kDa paraflagellar rod protein,PFR2	31.1 cell.organisation
Tb09.211.1750	mitochondrial phosphate transporter, putative	34 transport
Tb10.389.0690	mitochondrial 2-oxoglutarate/malate carrier protein, putative	34 transport
Tb10.61.1810	ADP/ATP translocase 1, putative	34 transport
Tb10.61.1820	ADP/ATP translocase 1, putative	34 transport
Tb10.61.1830	ADP/ATP translocase 1, putative	34 transport
Tb11.03.0030	ABC transporter, putative	34 transport
Tb927.3.2340	peroxin-2,glycosome import protein (gim1)	34 transport
Tb10.100.0090	vacuolar ATP synthase, putative	34.1 transport.p- and v-ATPases
Tb10.61.2680	pyruvate kinase 1	4 glycolysis
Tb927.1.3830	glucose-6-phosphate isomerase, glycosomal	4 glycolysis
Tb927.6.4300	glyceraldehyde 3-phosphate dehydrogenase, glycosomal	4 glycolysis
Tb927.1.700	phosphoglycerate kinase	4.1 glycolysis.phosphoglycerate kinase
Tb927.1.710	phosphoglycerate kinase	4.1 glycolysis.phosphoglycerate kinase
Tb927.1.720	phosphoglycerate kinase	4.1 glycolysis.phosphoglycerate kinase
Tb11.02.4150	pyruvate phosphate dikinase	4.4 glycolysis.PPFK
Tb09.211.0540	fructose-1,6-bisphosphatase	4.6 glycolysis.Fruc2,6BisPase
Tb927.2.5800	sedoheptulose-1,7-bisphosphatase	4.6 glycolysis.Fruc2,6BisPase
Tb10.70.1370	fructose-bisphosphate aldolase, glycosomal	4.7 glycolysis.aldolase
Tb11.02.3210	triosephosphate isomerase	4.8 glycolysis.TPI
Tb10.61.0980	glycosomal malate dehydrogenase	6.3 gluconeogenesis.Malate DH
Tb927.2.4210	glycosomal phosphoenolpyruvate carboxykinase,	6.4 gluconeogenese/ glyoxylate cycle.PEPCK
Tb10.70.5200	glucose-6-phosphate 1-dehydrogenase	7.1.1 OPP.oxidative PP.G6PD
Tb927.8.6170	transketolase, putative	7.2.1 OPP.non-reductive PP.transketolase
Tb11.01.3550	2-oxoglutarate dehydrogenase E2 component, putative	8 TCA / org. transformation
Tb11.03.0230	isocitrate dehydrogenase, putative	8.1.4 TCA / org. transformation.TCA.IDH
Tb10.6k15.3640	alternative oxidase	9.4 mitochondrial electron transport / ATP synthesis.alternative oxidase
Tb927.3.3270	6-phospho-1-fructokinase	4 glycolysis
Tb927.3.1380	mitochondrial precursor,ATP synthase F1, beta subunit	34.1 transport.p- and v-ATPases
Tb927.7.7430	mitochondrial precursor,ATP synthase F1, alpha subunit	34.1 transport.p- and v-ATPases
Tb09.160.0620	peroxisomal membrane protein 4, putative	35.1 not assigned.no ontology
Tb09.211.0170	hypothetical protein, conserved	35.1 not assigned.no ontology
Tb09.211.2250	hypothetical protein, conserved	35.1 not assigned.no ontology
Tb09.211.2730	Gim5A protein,glycosomal membrane protein	35.1 not assigned.no ontology
Tb09.211.4511	kinetoplastid membrane protein KMP-11	31.1 cell.organisation
Tb10.26.0680	hypothetical protein, conserved	35.1 not assigned.no ontology

Tb10.61.0440	peroxisome assembly protein, putative	35.1 not assigned.no ontology
Tb10.61.1260	hypothetical protein, conserved	35.1 not assigned.no ontology
Tb10.61.1550	hypothetical protein, conserved	35.1 not assigned.no ontology
Tb10.61.2210	hypothetical protein, conserved	35.1 not assigned.no ontology
Tb10.61.2220	hypothetical protein, conserved	35.1 not assigned.no ontology
Tb10.6k15.0400	hypothetical protein, conserved	35.1 not assigned.no ontology
Tb10.6k15.0810	hypothetical protein, conserved,leucine-rich repeat protein (LRF	35.1 not assigned.no ontology
Tb10.6k15.1510	hypothetical protein, conserved	35.1 not assigned.no ontology
Tb10.6k15.2920	hypothetical protein, conserved,rib72 protein-like protein	35.1 not assigned.no ontology
Tb10.70.1080	hypothetical protein, conserved	35.1 not assigned.no ontology
Tb10.70.5560	hypothetical protein, conserved	35.1 not assigned.no ontology
Tb10.v4.0053	hypothetical protein,chrX additional, unordered contigs	35.1 not assigned.no ontology
Tb10.v4.0248	hypothetical protein,chrX additional, unordered contigs	35.1 not assigned.no ontology
Tb11.01.0840	hypothetical protein, conserved	35.1 not assigned.no ontology
Tb11.01.1210	hypothetical protein, conserved	35.1 not assigned.no ontology
Tb11.01.1625	hypothetical protein, conserved	35.1 not assigned.no ontology
Tb11.01.1780	short-chain dehydrogenase, putative	35.1 not assigned.no ontology
Tb11.01.2800	hypothetical protein, conserved	35.1 not assigned.no ontology
Tb11.01.3000	hypothetical protein, conserved	35.1 not assigned.no ontology
Tb11.01.3370	glycosomal membrane protein, putative	35.1 not assigned.no ontology
Tb11.01.4030	hypothetical protein, conserved	35.1 not assigned.no ontology
Tb11.02.0140	hypothetical protein, conserved	35.1 not assigned.no ontology
Tb11.02.1260	hypothetical protein, conserved	35.1 not assigned.no ontology
Tb11.02.2490	hypothetical protein, conserved	35.1 not assigned.no ontology
Tb11.02.2530	hypothetical protein, conserved	35.1 not assigned.no ontology
Tb11.02.4320	hypothetical protein, conserved	35.1 not assigned.no ontology
Tb11.02.4380	hypothetical protein, conserved	35.1 not assigned.no ontology
Tb11.02.5460	hypothetical protein, conserved	35.1 not assigned.no ontology
Tb11.03.0470	hypothetical protein, conserved	35.1 not assigned.no ontology
Tb11.1220	hypothetical protein,chrXI additional, unordered contigs	35.1 not assigned.no ontology
Tb11.46.0011	hypothetical protein, conserved,leucine-rich repeat protein (LRF	35.1 not assigned.no ontology
Tb11.47.0006	hypothetical protein, conserved	35.1 not assigned.no ontology
Tb927.1.4490	acetyltransferase, putative	35.1 not assigned.no ontology
Tb927.1.5000	hypothetical protein, conserved	35.1 not assigned.no ontology
Tb927.2.2160	hypothetical protein, conserved	35.1 not assigned.no ontology
Tb927.2.2770	hypothetical protein, conserved	35.1 not assigned.no ontology

Tb927.3.3770	hypothetical protein, conserved	35.1 not assigned.no ontology
Tb927.3.3790	hypothetical protein, conserved	35.1 not assigned.no ontology
Tb927.3.4420	hypothetical protein, conserved	35.1 not assigned.no ontology
Tb927.4.1360	hypothetical protein, conserved	35.1 not assigned.no ontology
Tb927.4.1740	hypothetical protein, conserved	35.1 not assigned.no ontology
Tb927.4.2840	hypothetical protein, conserved	35.1 not assigned.no ontology
Tb927.4.4040	hypothetical protein, conserved	35.1 not assigned.no ontology
Tb927.4.4690	hypothetical protein, conserved	35.1 not assigned.no ontology
Tb927.4.4700	hypothetical protein, conserved	35.1 not assigned.no ontology
Tb927.5.1230	hypothetical protein, conserved	35.1 not assigned.no ontology
Tb927.5.2370	hydrolase, alpha/beta fold family, putative	35.1 not assigned.no ontology
Tb927.5.2650	hypothetical protein, conserved	35.1 not assigned.no ontology
Tb927.5.2950	hypothetical protein, conserved	35.1 not assigned.no ontology
Tb927.6.2200	hypothetical protein, conserved	35.1 not assigned.no ontology
Tb927.6.4140	hypothetical protein, conserved	35.1 not assigned.no ontology
Tb927.6.4520	hypothetical protein, conserved	35.1 not assigned.no ontology
Tb927.7.2190	hypothetical protein, conserved	35.1 not assigned.no ontology
Tb927.7.3740	hypothetical protein, conserved	35.1 not assigned.no ontology
Tb927.8.1550	hypothetical protein, conserved	35.1 not assigned.no ontology
Tb927.8.4580	hypothetical protein, conserved	35.1 not assigned.no ontology
Tb927.8.6240	hypothetical protein, conserved	35.1 not assigned.no ontology
Tb927.8.6640	hypothetical protein, conserved	35.1 not assigned.no ontology
Tb927.8.6660	hypothetical protein, conserved	35.1 not assigned.no ontology
Tb11.02.5550	hypothetical protein, conserved,WD40 repeat protein	35.1 not assigned.no ontology
