# Supplementary material for: Exocytosis and protein secretion in Trypanosoma
Source: BMC Microbiol. 2010 Jan 26;10:20. doi: 10.1186/1471-2180-10-20 (PMC3224696; doi:10.1186/1471-2180-10-20)
Supplement: Additional file 5 — Table S5. Proteins identified in total proteome from T. brucei [18]. contains the list of 1071 proteins from the total proteome which were classified into functional categories (MapMan bins nomenclature). [file 1471-2180-10-20-S5.PDF]

Table S5
Total proteome

Tb accession	Protein Name	mapman bin
Tb10.05.0080	glucosidase, putative	2.2.2.1 major CHO metabolism
Tb10.70.1370	ALD fructose-bisphosphate aldolase, glycosomal, putative	4 glycolysis
Tb09.211.0540	FBPase fructose-1,6-bisphosphate, cytosolic	4 glycolysis
Tb10.70.5200	G6PD glucose-6-phosphate 1-dehydrogenase	4 glycolysis
Tb06.26G9.1030	GAPDH glyceraldehyde 3-phosphate dehydrogenase, putative	4 glycolysis
Tb10.70.5800	hexokinase	4 glycolysis
Tb10.70.5820	hexokinase	4 glycolysis
Tb927.1.3830	PGI glucose-6-phosphate isomerase, glycosomal	4 glycolysis
Tb927.1.710	PGKB phosphoglycerate kinase	4 glycolysis
Tb11.22.0003	phosphoglycerate kinase, putative	4 glycolysis
Tb11.02.4150	PPDK pyruvate phosphate dikinase	4 glycolysis
Tb03.30P12.440	pyruvate dehydrogenase E1 beta subunit, putative	4 glycolysis
Tb10.389.0890	pyruvate dehydrogenase E1 component alpha subunit, putative	4 glycolysis
Tb03.3K10.320	TbPFK ATP-dependent phosphofructokinase; 6-phospho-1-fructokinase	4 glycolysis
Tb11.02.3210	TIM triosephosphate isomerase	4 glycolysis
Tb10.70.0630	aldehyde dehydrogenase, putative	4 glycolysis
Tb10.389.0330	UTP-glucose-1-phosphate uridylyltransferase 2, putative	4.1 glycolysis
Tb10.70.4740	enolase	4.12 glycolysis.enolase
Tb11.01.3040	cytosolic malate dehydrogenase, putative	6.3 gluconeogenesis
Tb11.02.3120	malic enzyme, putative	6.3 gluconeogenesis.
Tb11.02.3130	malic enzyme, putative	6.3 gluconeogenesis.
Tb10.70.5110	mMDH malate dehydrogenase	6.3 gluconeogenesis.
Tb10.61.0980	gMDH glycosomal malate dehydrogenase	6.3 gluconeogenesis.Malate DH
Tb927.2.4210	glycosomal phosphoenolpyruvate carboxykinase; glycosomal protein P60	6.4 gluconeogenese/ glyoxylate cycle.PEPCK
Tb06.28P18.310	2-hydroxy-3-oxopropionate reductase, putative	6 gluconeogenese/ glyoxylate cycle
Tb11.01.2440	glycosyl hydrolase, putative	26.3 misc.gluco-, galacto- and mannosidases
Tb08.26E13.220	transaldolase, putative	7.2.2 OPP.non-reductive PP.transaldolase
Tb10.61.2880	ACO aconitase	8.1.3 TCA / org. transformation.TCA.aconitase
Tb10.1650	AcO aconitase 24529:27222 forward MW:98316	8.1.3 TCA / org. transformation.TCA.aconitase
Tb08.10J17.710	isocitrate dehydrogenase	8.1.4 TCA / org. transformation.TCA.IDH
Tb11.03.0230	isocitrate dehydrogenase, putative	8.1.4 TCA / org. transformation.TCA.IDH
Tb11.01.1740	2-oxoglutarate dehydrogenase E1 component, putative	8.1.5 TCA / org. transformation.TCA.2-oxoglutarate dehydrogenase

Tb11.47.0004	2-oxoglutarate dehydrogenase subunit, putative	8.1.5 TCA / org. transformation.TCA.2-oxoglutarate dehydrogenase
Tb11.01.3550	2-oxoglutarate dehydrogenase, E2 component,	8.1.5 TCA / org. transformation.TCA.2-oxoglutarate dehydrogenase
Tb10.6k15.3250	succinyl-CoA ligase	8.1.6 TCA / org. transformation.TCA.succinyl-CoA ligase
Tb03.48O8.710	succinyl-CoA synthetase alpha subunit, putative	8.1.6 TCA / org. transformation.TCA.succinyl-CoA ligase
Tb11.02.0290	succinyl-coA:3-ketoacid-coenzyme A transferase,	8.1.6 TCA / org. transformation.TCA.succinyl-CoA ligase
TB05.28F8.440	NADH-dependent fumarate reductase, putative	8.1.7 TCA / org. transformation.TCA.succinate dehydrogenase
Tb08.30K1.380	succinate dehydrogenase flavoprotein, putative	8.1.7 TCA / org. transformation.TCA.succinate dehydrogenase
TB05.28F8.460	FRDg NADH-dependent fumarate reductase	8.1.8 TCA / org. transformation.TCA.fumarase
Tb03.26J7.860	fumarate hydratase, putative	8.1.8 TCA / org. transformation.TCA.fumarase
Tb08.10K10.130	ubiquinol-cytochrome C reductase hinge prote	9.5 mitochondrial electron transport / ATP synthesis.cytochrome c reductase
Tb04.3M17.340	ubiquinol-cytochrome C reductase, putative	9.5 mitochondrial electron transport / ATP synthesis.cytochrome c reductase
Tb09.160.1820	COX V cytochrome c oxidase subunit V, putative	9.7 mitochondrial electron transport / ATP synthesis.cytochrome c oxidase
Tb08.5H5.620	cytochrome c	9.7 mitochondrial electron transport / ATP synthesis.cytochrome c oxidase
Tb11.01.4702	cytochrome c oxidase subunit 10, putative	9.7 mitochondrial electron transport / ATP synthesis.cytochrome c oxidase
Tb09.211.4110	cytochrome p450 reductase, putative	9.7 mitochondrial electron transport / ATP synthesis.cytochrome c oxidase
Tb927.1.4100	trCOIV cytochrome C oxidase subunit IV (PMID:12467979)	9.7 mitochondrial electron transport / ATP synthesis.cytochrome c oxidase
Tb03.28C22.510	carnitine/choline acetyltransferase, putative	11 lipid metabolism
Tb09.211.3540	glk1 glycerol kinase, glycosomal	11.5 lipid metabolism.glycerol metabolism
Tb09.211.3550	glk1 glycerol kinase, glycosomal	11.5 lipid metabolism.glycerol metabolism
Tb04.2H8.550	acyltransferase, putative	11.1 lipid metabolism.glycolipid synthesis
Tb10.6k15.3080	dihydrolipoamide acetyltransferase precursor, putative	11.1.31 lipid metabolism.
Tb04.3M17.450	dihydrolipoamide dehydrogenase, putative	11.1.31 lipid metabolism.
Tb11.01.8470	dihydrolipoyl dehydrogenase	11.1.31 lipid metabolism.
Tb08.11J15.550	transketolase, putative	11.1.31 lipid metabolism.FA synthesis and FA elongation.pyruvate DH
Tb08.11J15.870	fatty acid desaturase, putative	11.2.1 lipid metabolism.FA desaturation.desaturase
Tb927.2.3080	fatty acid desaturase, putative; oleate desaturase, putative	11.2.1 lipid metabolism.FA desaturation.desaturase
Tb08.28L1.740	glycerol-3-phosphate dehydrogenase	11.3 lipid metabolism.Phospholipid synthesis
Tb08.29O9.70	maoC-like dehydratase, putative	11.8 lipid metabolism.exotics
Tb11.02.4080	lanosterol 14-alpha-demethylase	11.8.6 lipid metabolism.exotics (steroids, squalene etc).
Tb07.27E10.490	lanosterol synthase	11.8.6 lipid metabolism.exotics (steroids, squalene etc).
Tb03.28C22.580	lipase domain protein, putative	11.9.2.1 lipid metabolism.lipid degradation.lipases.triacylglycerol lipase
Tb927.1.4830	phospholipase A1, putative	11.9.3 lipid metabolism.lipid degradation.lysophospholipase
Tb03.48K5.550	enoyl-CoA hydratase, mitochondrial precursor, putative	11.9.4.3 lipid metabolism.lipid degradation.beta-oxidation.enoyl CoA hydratase
Tb11.01.8200	enoyl-CoA hydratase/isomerase family protein, putative	11.9.4.3 lipid metabolism.lipid degradation.beta-oxidation.enoyl CoA hydratase
Tb09.211.3850	aminotransferase, putative	13 amino acid metabolism
Tb10.389.1810	aminotransferase, putative	13 amino acid metabolism

Tb927.2.4590	branched-chain amino acid aminotransferase, putative	13 amino acid metabolism
Tb09.160.4310	GDH glutamate dehydrogenase	13 amino acid metabolism
Tb08.11J15.760	2-amino-3-ketobutyrate coenzyme A ligase, putative; glycine acetyltransferase,	13 amino acid metabolism
Tb11.02.4480	3-methylcrotonoyl-CoA carboxylase beta subunit, putative	13.1.1 amino acid metabolism
Tb09.160.4570	AK1 arginine kinase	13.1.1 amino acid metabolism
Tb09.160.4560	arginine kinase	13.1.1 amino acid metabolism
Tb07.27M11.260	asparagine synthetase a, putative	13.1.1 amino acid metabolism
Tb11.02.2740	aspartate aminotransferase, mitochondrial	13.1.1.2 amino acid metabolism
Tb05.6E7.770	aspartate carbamoyltransferase, putative	13.1.1.2 amino acid metabolism
Tb05.6E7.720	carbamoyl-phosphate synthase, putative	13.1.2.3.11 amino acid metabolism.synthesis.
Tb11.02.5400	cystathionine beta-synthase, putative	13.1.2.3.11 amino acid metabolism.synthesis.
Tb06.5F5.290	L-threonine 3-dehydrogenase, putative	13.1.2.3.11 amino acid metabolism.synthesis.
Tb11.01.1350	S-adenosylhomocysteine hydrolase, putative	13.1.3.4 amino acid metabolism.synthesis.aspartate family.methionine
Tb06.30P15.570	S-adenosylmethionine synthetase	13.1.3.4 amino acid metabolism.synthesis.aspartate family.methionine
Tb06.30P15.600	S-adenosylmethionine synthetase	13.1.3.4 amino acid metabolism.synthesis.aspartate family.methionine
Tb06.30P15.650	S-adenosylmethionine synthetase	13.1.3.4 amino acid metabolism.synthesis.aspartate family.methionine
Tb10.1520	s-adenosyl-L-methionine-c-24-delta-sterol-methy lt ransferase a, putative 1029	13.1.3.4 amino acid metabolism.synthesis.aspartate family.methionine
Tb927.1.1270	homocysteine S-methyltransferase, putative	13.1.3.4.3 amino acid metabolism.
Tb03.27F10.1000	N-acetyltransferase complex ARD1 subunit, putative	13.1.3.4.3 amino acid metabolism.
Tb10.70.4280	delta-1-pyrroline-5-carboxylate dehydrogenase, putative;	13.2.2.2 amino acid metabolism.degradation
Tb08.26N11.490	arginase, putative	13.2.2.3 amino acid metabolism
Tb06.26G9.610	3-hydroxy-3-methylglutaryl-CoA reductase, putative	16.1.2.3 secondary metabolism.isoprenoids.mevalonate pathway.
Tb10.6k15.3820	sterol 24-c-methyltransferase, putative	17.3.1.2.2 hormone metabolism.brassinosteroid.synthesis-degradation.
Tb11.01.1820	biotin/lipoate protein ligase-like protein	18 Co-factor and vitamine metabolism
Tb06.5F5.240	pyridoxal kinase, putative	18.20* Co-factor and vitamine metabolism.vitamin b6
Tb10.406.0520	trypanothione reductase	21.2 redox.ascorbate and glutathione
Tb927.2.4370	TRYS trypanothione synthetase, putative	21.2 redox.ascorbate and glutathione
Tb11.47.0012	glutaredoxin, putative	21.4 redox.glutaredoxins
Tb08.26N11.460	peroxidoxin; tryparedoxin peroxidase	21.5 redox.peroxiredoxins
TB05.28F8.400	thioredoxin-like protein	21.5 redox.peroxiredoxins
Tb09.160.4250	TRYP1 tryparedoxin peroxidase	21.5 redox.peroxiredoxins
Tb10.70.0480	trypanin trypanin	21.5 redox.peroxiredoxins
Tb03.28C22.710	tryparedoxin	21.5 redox.peroxiredoxins
Tb11.01.7550	iron superoxide dismutase	21.6 redox.dismutases and catalases
Tb05.27M3.490	iron superoxide dismutase, putative	21.6 redox.dismutases and catalases
Tb07.13M20.750	NADH-cytochrome b5 reductase, putative	21.99 redox.misc

Tb07.13M20.760	NADH-cytochrome b5 reductase, putative	21.99 redox.misc
Tb10.389.1850	NAD or NADP dependent oxidoreductase, putative; short chain dehydrogenase,	21.99 redox.misc
Tb11.01.5300	ornithine decarboxylase	22.1 polyamine metabolism.synthesis
Tb07.43M14.180	adenine phosphoribosyltransferase, putative	23 nucleotide metabolism
Tb06.4M18.780	adenosine kinase, putative	23 nucleotide metabolism
Tb09.211.4460	ADP-ribosylation factor, putative	23 nucleotide metabolism
Tb11.01.7890	cyclic nucleotide-binding protein, putative	23 nucleotide metabolism
Tb11.01.5730	ethanolamine-phosphate cytidylyltransferase, putative	23 nucleotide metabolism
Tb11.01.7800	NDPK nucleoside diphosphate kinase	23 nucleotide metabolism
Tb08.29H22.830	nucleoside phosphorylase, putative	23 nucleotide metabolism
Tb09.160.3630	PDE cyclic nucleotide phosphodiesterase	23 nucleotide metabolism
Tb09.160.3590	PDE2C cAMP-specific phosphodiesterase	23 nucleotide metabolism
Tb06.28P18.320	polynucleotide kinase 3'-phosphatase, putative	23 nucleotide metabolism
Tb09.160.2600	pyrazinamidase/nicotinamidase, putative	23 nucleotide metabolism
Tb06.26F19.110	Receptor-type adenylate cyclase GRESAG 4, putative	23 nucleotide metabolism
Tb06.28F21.250	receptor-type adenylate cyclase GRESAG 4, putative	23 nucleotide metabolism
Tb08.30P3.110	receptor-type adenylate cyclase GRESAG 4, putative	23 nucleotide metabolism
Tb08.6H23.130	receptor-type adenylate cyclase GRESAG 4, putative	23 nucleotide metabolism
Tb08.6H23.210	receptor-type adenylate cyclase GRESAG 4, putative	23 nucleotide metabolism
Tb05.29K2.90	receptor-type adenylate cyclase GRESAG 4, putative; adenylyl cyclase, putative	23 nucleotide metabolism
Tb11.02.3740	receptor-type adenylate cyclase, putative	23 nucleotide metabolism
Tb11.03.0090	ribokinase, putative	23 nucleotide metabolism
Tb11.01.0700	ribose 5-phosphate isomerase, putative	23 nucleotide metabolism
Tb11.01.3170	guanine nucleotide-binding protein beta subunit- like protein;	23 nucleotide metabolism
Tb11.01.3180	guanine nucleotide-binding protein beta subunit-like protein;	23 nucleotide metabolism
Tb05.6E7.780	dihydroorotate dehydrogenase, putative	23.1.1.4 nucleotide metabolism.
Tb927.2.5660	adenylate kinase	23.1.2 nucleotide metabolism.synthesis.purine
Tb10.70.5150	adenylate kinase, putative	23.1.2 nucleotide metabolism.synthesis.purine
Tb10.70.7330	adenylate kinase, putative	23.1.2 nucleotide metabolism.synthesis.purine
Tb11.02.1120	adenylosuccinate synthetase, putative	23.1.2 nucleotide metabolism.synthesis.purine
Tb07.27E10.300	ATP-NAD kinase-like protein	23.1.2 nucleotide metabolism.synthesis.purine
Tb10.70.6540	HGPRT hypoxanthine-guanine phosphoribosyltransferase	23.1.2 nucleotide metabolism.synthesis.purine
Tb10.70.6650	HGPRT hypoxanthine-guanine phosphoribosyltransferase	23.1.2 nucleotide metabolism.synthesis.purine
Tb05.1P6.110	inosine-5'-monophosphate dehydrogenase, putative	23.1.2.30 nucleotide metabolism.synthesis.purine.IMP dehydrogenase
Tb10.61.0150	inosine-5'-monophosphate dehydrogenase; IMP dehydrogenase	23.1.2.30 nucleotide metabolism.synthesis.purine.IMP dehydrogenase
Tb07.26A24.1100	inositol polyphosphate kinase-like protein, putative	23.1.2.30 nucleotide metabolism.synthesis.purine.IMP dehydrogenase

Tb07.43M14.550	GMP synthase, putative; glutamine amidotransferase, putative	23.1.2.31 nucleotide metabolism.synthesis.purine.GMP synthetase
Tb09.211.1320	AMP deaminase, putative	23.2 nucleotide metabolism.degradation
Tb11.02.1340	AMP deaminase, putative	23.2 nucleotide metabolism.degradation
Tb10.70.7270	thymidine kinase, putative	23.3.2.3 nucleotide metabolism.salvage.nucleoside kinases.thymidine kinase
Tb10.70.0780	tRNA pseudouridine synthase A, putative	23.5.2 nucleotide metabolism.deoxynucleotide metabolism
Tb04.2L9.510	cleavage and polyadenylation specificity factor, putative	27.1 RNA.processing
Tb10.6k15.3160	fibrillarin	27.1 RNA.processing
Tb10.61.2040	fibrillarin, putative	27.1 RNA.processing
Tb927.2.3880	heterogeneous nuclear ribonucleoprotein H/F, putative	27.1 RNA.processing
Tb11.02.0490	MP46 RNA editing complex protein MP46; mitochondrial	27.1 RNA.processing
Tb07.28B13.30	poly(A) polymerase, putative	27.1 RNA.processing
Tb09.211.2150	poly(A)-binding protein; PABP2	27.1 RNA.processing
Tb09.211.0930	polyadenylate-binding protein 1, putative; PABP1	27.1 RNA.processing
Tb09.211.2420	PRP8 protein homologue; U5 snRNA-associated splicing factor	27.1 RNA.processing
Tb06.3A7.50	PUF4 pumillio RNA binding protein 4, putative	27.1 RNA.processing
Tb11.02.5770	RBP16 mitochondrial RNA binding protein, putative; RBP16	27.1 RNA.processing
Tb07.13M20.190	Sm-D1 small nuclear ribonucleoprotein, putative;	27.1 RNA.processing
Tb927.2.4550	FtsJ cell division protein, putative	27.1 RNA.processing
Tb11.01.3690	splicing factor 3B subunit 1, putative	27.1.1 RNA.processing.splicing
Tb10.70.2470	ribonuclease HII, putative	27.1.19 RNA.processing.ribonucleases
Tb05.1P6.870	ribonucleoprotein p18, mitochondrial precursor, putative	27.1.19 RNA.processing.ribonucleases
Tb07.26A24.100	XRNA; KEM; XNR1 5'-3' exonuclease XRNA, putative; exoribonuclease 1, putative	27.1.19 RNA.processing.ribonucleases
Tb10.70.0610	XRND exoribonuclease 2, putative	27.1.19 RNA.processing.ribonucleases
Tb05.45E22.770	nucleolar RNA helicase II, putative; nucleolar RNA helicase Gu, putative	27.1.2 RNA.processing.RNA helicase
Tb05.30H13.60	pre-mRNA splicing factor ATP-dependent RNA helicase, putative	27.1.2 RNA.processing.RNA helicase
Tb10.6k15.0530	RNA helicase, putative; ATP-dependent RNA helicase, putative	27.1.2 RNA.processing.RNA helicase
Tb10.70.7730	RNA helicase, putative; DEAD box RNA helicase, putative	27.1.2 RNA.processing.RNA helicase
Tb11.01.6260	RNA helicase, putative; DEAD/DEAH box helicase, putative	27.1.2 RNA.processing.RNA helicase
Tb04.30O21.140	DNA-directed RNA polymerase II subunit 2, putative	27.2 RNA.transcription
Tb927.1.540	DNA-directed RNA polymerase III, putative	27.2 RNA.transcription
Tb11.02.5790	RNA polymerase B subunit RPB8, putative	27.2 RNA.transcription
Tb08.5H5.740	TRP11 DNA-directed RNA polymerase I largest subunit	27.2 RNA.transcription
Tb927.2.1810	transcription activator, putative	27.2 RNA.transcription
Tb11.02.2300	POLIB mitochondrial DNA polymerase I protein B, putative;	27.3.67 RNA.regulation of transcription.putative transcription regulator
Tb06.4M18.600	mitochondrial oligo_U binding protein TBRGG1	27.3.99 RNA.regulation of transcription.unclassified
Tb11.02.1390	N(2), N(2)-dimethylguanosine tRNA methyltransferase, putative	27.3.99 RNA.regulation of transcription.unclassified

Tb11.55.0009	GBP21 mitochondrial RNA binding protein 1; gBP21, MRP1	27.4 RNA.RNA binding
Tb10.70.5360	LA RNA binding protein, putative; RNA-binding protein	27.4 RNA.RNA binding
Tb08.12O16.330	Nopp44/46 nucleolar RNA-binding protein	27.4 RNA.RNA binding
Tb08.10J17.600	nucleolar protein, putative	27.4 RNA.RNA binding
Tb08.12O16.310	nucleolar RNA-binding protein, truncated	27.4 RNA.RNA binding
Tb09.211.4540	RNA-binding protein, putative; DRBD2	27.4 RNA.RNA binding
Tb09.211.0560	RNA-binding protein, putative; DRBD4	27.4 RNA.RNA binding
Tb10.389.1650	RNA-binding protein, putative; RBP7	27.4 RNA.RNA binding
Tb11.03.0580	RNA-binding protein, putative; UBP1A	27.4 RNA.RNA binding
Tb11.03.0620	RNA-binding protein, putative; UBP1B	27.4 RNA.RNA binding
Tb10.6k15.0620	REAP-1 RNA-editing-associated protein 1; RNA-binding protein;	27.4 RNA.RNA binding
Tb08.26A17.830	TbRBP38 mitochondrial RNA binding protein, putative	27.4 RNA.RNA binding
Tb03.27F10.990	ATP-dependent DEAD/H RNA helicase, putative	28.1 DNA.synthesis/chromatin structure
Tb05.6E7.340	ATP-dependent DEAD/H RNA helicase, putative	28.1 DNA.synthesis/chromatin structure
Tb08.29O9.160	ATP-dependent DEAD/H RNA helicase, putative	28.1 DNA.synthesis/chromatin structure
Tb10.70.3290	ATP-dependent DEAD-box RNA helicase, putative	28.1 DNA.synthesis/chromatin structure
Tb05.45E22.570	ATP-dependent RNA helicase, putative	28.1 DNA.synthesis/chromatin structure
Tb06.26G9.430	ATP-dependent RNA helicase, putative	28.1 DNA.synthesis/chromatin structure
Tb09.211.3510	ATP-dependent RNA helicase, putative	28.1 DNA.synthesis/chromatin structure
Tb10.61.2130	ATP-dependent RNA helicase, putative	28.1 DNA.synthesis/chromatin structure
Tb10.6k15.3220	ATP-dependent RNA helicase, putative; ATP- dependent RNA helicase, putative	28.1 DNA.synthesis/chromatin structure
Tb10.70.0540	DEAD box RNA helicase, putative	28.1 DNA.synthesis/chromatin structure
Tb11.01.8290	DEAD/DEAH box helicase, putative	28.1 DNA.synthesis/chromatin structure
Tb10.70.1790	DEAH-box RNA helicase, putative	28.1 DNA.synthesis/chromatin structure
Tb07.5F10.540	DNA excision repair protein, putative; SNF2 family helicase-like protein, putative	28.1 DNA.synthesis/chromatin structure
Tb06.26G9.80	DNA ligase I, putative	28.1 DNA.synthesis/chromatin structure
Tb08.28L1.310	DNA polymerase zeta catalytic subunit, putative	28.1 DNA.synthesis/chromatin structure
Tb11.01.7810	DNA replication licensing factor, putative	28.1 DNA.synthesis/chromatin structure
Tb10.70.5940	DNA topoisomerase IA, putative	28.1 DNA.synthesis/chromatin structure
Tb11.01.3390	DNA topoisomerase II, putative	28.1 DNA.synthesis/chromatin structure
Tb11.01.1280	DNA topoisomerase III, putative	28.1 DNA.synthesis/chromatin structure
Tb03.5L5.180	exonuclease, putative	28.1 DNA.synthesis/chromatin structure
Tb03.27F10.640	flap endonuclease-1 (FEN-1), putative	28.1 DNA.synthesis/chromatin structure
Tb927.1.2430	histone H3, putative	28.1 DNA.synthesis/chromatin structure
Tb05.45E22.470	histone H4, putative	28.1 DNA.synthesis/chromatin structure
Tb11.01.3510	minchromosome maintenance (MCM) complex subunit, putative	28.1 DNA.synthesis/chromatin structure

Tb05.1P6.900	replication Factor A 28 kDa subunit, putative	28.1 DNA.synthesis/chromatin structure
Tb11.01.0870	replication factor A, 51kDa subunit, putative	28.1 DNA.synthesis/chromatin structure
Tb04.2L9.390	RuvB-like DNA helicase, putative	28.1 DNA.synthesis/chromatin structure
Tb04.29M18.640	RuvB-like DNA helicase, putative; ATP-dependent DNA helicase, putative	28.1 DNA.synthesis/chromatin structure
Tb11.02.5060	SNF2/RAD54 related DNA helicase, putative	28.1 DNA.synthesis/chromatin structure
Tb08.11J15.890	TFIIH basal transcription factor complex helicase subunit, putative;	28.1 DNA.synthesis/chromatin structure
Tb10.1680	uracil-DNA glycosylase, putative 32424:33317 forward MW:3269	28.1 DNA.synthesis/chromatin structure
Tb10.406.0500	DNA replication licensing factor, putative; minichromosome maintenance protein-like	28.1 DNA.synthesis/chromatin structure
Tb10.70.0800	universal minicircle sequence binding protein (UMSBP), putative;	28.1 DNA.synthesis/chromatin structure
Tb10.70.0820	universal minicircle sequence binding protein (UMSBP), putative;	28.1 DNA.synthesis/chromatin structure
Tb11.01.1950	telomerase reverse transcriptase, putative	28.1 DNA.synthesis/chromatin structure
Tb09.160.4090	TOP2 mitochondrial DNA topoisomerase II	28.1 DNA.synthesis/chromatin structure
Tb10.61.2910	uracil-DNA glycosylase, putative	28.1 DNA.synthesis/chromatin structure
Tb09.211.2970	structural maintenance of chromosome (SMC) family protein, putative	28.1 DNA.synthesis/chromatin structure
Tb10.406.0600	structural maintenance of chromosome (SMC), putative	28.1 DNA.synthesis/chromatin structure
Tb11.01.3270	A/G-specific adenine glycosylase, putative	28.2 DNA.repair
Tb10.26.0940	Deoxyribodipyrimidine photolyase, putative; DNA repair enzyme, putative	28.2 DNA.repair
Tb09.211.4430	helicase, putative	28.2 DNA.repair ou 27.3.44 RNA.regulation of transcription.
Tb11.01.1100	helicase-like protein	28.2 DNA.repair ou 27.3.44 RNA.regulation of transcription.
Tb11.02.3400	helicase-like protein , putative; SNF2 family protein	28.2 DNA.repair ou 27.3.44 RNA.regulation of transcription.
Tb03.30P12.1230	helicase-like protein, putative	28.2 DNA.repair ou 27.3.44 RNA.regulation of transcription.
Tb04.1H19.630	asparaginyl-tRNA synthetase, putative	29.1 protein.aa activation
Tb06.3A7.350	cysteinyl-tRNA synthetase, putative	29.1 protein.aa activation
Tb09.160.3730	glutaminyl-tRNA synthetase, putative	29.1 protein.aa activation
Tb11.01.1400	glycyl-tRNA synthetase, putative	29.1 protein.aa activation
Tb11.22.0005	phenylalanyl-tRNA synthetase, putative	29.1 protein.aa activation
TB05.28F8.150	threonyl-tRNA synthetase, putative	29.1.15 protein.aa activation.
Tb03.5L5.820	tryptophanyl-tRNA synthetase, putative	29.1.15 protein.aa activation.
Tb08.5H5.270	tyrosyl or methionyl-tRNA synthetase, putative	29.1.15 protein.aa activation.
Tb07.28B13.380	tyrosyl-tRNA synthetase, putative	29.1.15 protein.aa activation.
Tb06.26G9.740	valyl-tRNA synthetase, putative	29.1.15 protein.aa activation.
Tb10.389.0630	prolyl-trna synthetase, putative; bifunctional aminoacyl-trna synthetase, putative	29.1.15 protein.aa activation.proline-tRNA ligase
Tb11.02.4030	ERF3 eukaryotic release factor 3, putative	29.2 protein.synthesis
Tb09.160.3270	eukaryotic initiation factor 4a, putative	29.2 protein.synthesis
Tb11.03.0410	eukaryotic initiation factor 5a, putative	29.2 protein.synthesis
Tb08.11J15.1060	eukaryotic translation initiation factor 1A, putative	29.2 protein.synthesis

Tb06.26G9.950	eukaryotic translation initiation factor 3 subunit 7-like protein	29.2 protein.synthesis
Tb10.6k15.2220	eukaryotic translation initiation factor 3 subunit 8, putative	29.2 protein.synthesis
Tb11.01.1370	eukaryotic translation initiation factor 3 subunit, putative	29.2 protein.synthesis
Tb10.70.4880	eukaryotic translation initiation factor 5, putative	29.2 protein.synthesis
Tb10.70.1770	Eukaryotic translation initiation factor 6 (eIF- 6), putative	29.2 protein.synthesis
Tb11.01.3420	eukaryotic translation initiation factor, putative	29.2 protein.synthesis
Tb11.01.4830	eukaryotic translation initiation factor, putative	29.2 protein.synthesis
Tb04.26G5.400	translation elongation factor 1-beta, putative	29.2 protein.synthesis
Tb04.26G5.430	translation elongation factor 1-beta, putative	29.2 protein.synthesis
Tb10.70.1100	translation elongation factor 1-beta, putative	29.2 protein.synthesis
Tb03.48O8.820	translation elongation factor EF-2, putative	29.2 protein.synthesis
Tb11.01.2780	translation initiation factor 2 subunit, putative	29.2 protein.synthesis
Tb11.39.0006	translation initiation factor eIF2B subunit-like protein, putative	29.2 protein.synthesis
Tb08.30K1.810	translation initiation factor IF-2, putative	29.2 protein.synthesis
Tb04.5E12.640	ribosomal protein L14, putative	29.2.2 protein.synthesis.misc ribososomal protein
Tb06.30P15.400	ribosomal protein L15, putative	29.2.2 protein.synthesis.misc ribososomal protein
Tb04.2L9.1330	ribosomal protein L3, putative	29.2.2 protein.synthesis.misc ribososomal protein
Tb03.1J15.510	ribosomal protein s25, putative	29.2.2 protein.synthesis.misc ribososomal protein
Tb10.70.3160	RPL30 60S ribosomal protein L30	29.2.2 protein.synthesis.misc ribososomal protein
Tb11.02.5020	seryl-tRNA synthetase, putative	29.2.4 protein.synthesis.
Tb10.6k15.2050	40S ribosomal protein S12, putative	29.2.4 protein.synthesis.elongation
Tb07.22O10.320	40S ribosomal protein S15, putative	29.2.4 protein.synthesis.elongation
Tb10.70.7020	40S ribosomal protein S23, putative	29.2.4 protein.synthesis.elongation
Tb11.01.1475	40S ribosomal protein S27, putative	29.2.4 protein.synthesis.elongation
Tb09.160.4450	40S ribosomal protein S3, putative	29.2.4 protein.synthesis.elongation
Tb10.70.3360	40S ribosomal protein S3a, putative	29.2.4 protein.synthesis.elongation
Tb11.02.1085	40s ribosomal protein S4, putative	29.2.4 protein.synthesis.elongation
Tb11.02.1090	40S ribosomal protein S4, putative	29.2.4 protein.synthesis.elongation
Tb09.244.2630	40S ribosomal protein S6, putative	29.2.4 protein.synthesis.elongation
Tb11.01.2560	40S ribosomal protein SA, putative	29.2.4 protein.synthesis.elongation
Tb06.30P15.270	60S acidic ribosomal protein P2, putative	29.2.4 protein.synthesis.elongation
Tb09.160.4200	60S acidic ribosomal protein, putative	29.2.4 protein.synthesis.elongation
Tb11.46.0001	60S acidic ribosomal subunit protein, putative	29.2.4 protein.synthesis.elongation
Tb09.244.2730	60S ribosomal protein L5, putative	29.2.4 protein.synthesis.elongation
Tb10.26.0560	60S ribosomal protein L6, putative	29.2.4 protein.synthesis.elongation
Tb10.70.7010	60S ribosomal protein L9, putative	29.2.4 protein.synthesis.elongation

Tb11.01.4660	elongation factor 1 gamma, putative	29.2.4 protein.synthesis.elongation
Tb10.70.5680	Elongation factor 1-alpha	29.2.4 protein.synthesis.elongation
Tb09.211.2110	elongation factor 1-alpha, putative; hsp70 subfamily B suppressor 1	29.2.4 protein.synthesis.elongation
Tb10.70.2650	elongation factor 2	29.2.4 protein.synthesis.elongation
Tb10.70.2660	elongation factor 2	29.2.4 protein.synthesis.elongation
Tb10.389.0070	elongation factor TU, putative	29.2.4 protein.synthesis.elongation
Tb03.27C5.290	elongation initiation factor 2 alpha subunit, putative	29.2.4 protein.synthesis.elongation
Tb927.2.3620	GTP-binding elongation factor Tu family, putative	29.2.4 protein.synthesis.elongation
Tb10.70.2190	Mitochondrial elongation factor G, putative	29.2.4 protein.synthesis.elongation
Tb09.160.3670	NHP2 ribosomal protein S6, putative; NPH2/RS6-like protein	29.2.4 protein.synthesis.elongation
Tb04.29M18.70	selenocysteine-tRNA-specific elongation factor, putative	29.2.4 protein.synthesis.elongation
Tb10.70.5650	TEF1 Elongation factor 1-alpha	29.2.4 protein.synthesis.elongation
Tb10.70.5670	TEF1 Elongation factor 1-alpha	29.2.4 protein.synthesis.elongation
Tb07.26A24.1130	threonine synthase, putative	29.2.4 protein.synthesis.elongation
Tb11.01.1465	nascent polypeptide associated complex alpha subunit, putative	29.2.4 protein.synthesis.elongation ou 27.3.67 RNA.regulation of transcription.
Tb09.211.0120	nascent polypeptide associated complex subunit, putative	29.2.4 protein.synthesis.elongation ou 27.3.67 RNA.regulation of transcription
Tb10.70.1190	VCP Transitional endoplasmic reticulum ATPase, putative;	29.3 protein.targeting
Tb10.61.0850	signal recognition particle protein, putative	29.3 protein.targeting
Tb10.6k15.2350	nuclear pore complex protein (NUP155), putative; nucleoporin, putative	29.3.1 protein.targeting.nucleus
Tb07.10C21.220	nuclear transport factor 2 protein(NFT2), putative	29.3.1 protein.targeting.nucleus
Tb08.29H22.480	nuclear transport factor 2, putative	29.3.1 protein.targeting.nucleus
Tb927.2.4230	NUP-1 protein, putative	29.3.1 protein.targeting.nucleus
Tb10.70.5460	vacuolar protein sorting-associated protein 35, putative	29.3.4.3 protein.targeting.secretory pathway.vacuole
Tb10.61.2630	protein transport protein sec13, putative	29.3.4.4 protein.targeting.secretory pathway.plasma membrane
Tb08.10J17.780	protein transport protein Sec23, putative	29.3.4.4 protein.targeting.secretory pathway.plasma membrane
Tb927.1.4690	arginine N-methyltransferase, putative	29.4 protein.postranslational modification
TB05.28F8.770	casein kinase I, epsilon isoform, putative	29.4 protein.postranslational modification
Tb927.1.870	deoxyhypusine synthase, putative	29.4 protein.postranslational modification
Tb05.6E7.390	dual-specificity protein phosphatase, putative	29.4 protein.postranslational modification
Tb10.61.2550	N-myristoyl transferase, putative	29.4 protein.postranslational modification
Tb927.1.3200	phosphatase-like protein, putative	29.4 protein.postranslational modification
Tb10.389.0020	phosphatidic acid phosphatase alpha, putative	29.4 protein.postranslational modification
Tb11.02.2210	PKA-R; RSU protein kinase A regulatory subunit	29.4 protein.postranslational modification
Tb07.27E10.480	protein kinase, putative	29.4 protein.postranslational modification
Tb07.29K4.230	protein kinase, putative	29.4 protein.postranslational modification
Tb07.6C8.130	protein kinase, putative	29.4 protein.postranslational modification

Tb09.160.0450	protein kinase, putative	29.4 protein.postranslational modification
Tb09.211.3410	protein kinase, putative	29.4 protein.postranslational modification
Tb10.61.0100	protein kinase, putative	29.4 protein.postranslational modification
Tb11.01.0400	protein kinase, putative	29.4 protein.postranslational modification
Tb927.1.1530	protein kinase, putative	29.4 protein.postranslational modification
Tb11.02.0640	protein kinase, putative; dual-specificity protein kinase, putative	29.4 protein.postranslational modification
Tb10.61.1880	protein kinase, putative; mitogen-activated protein kinase, putative	29.4 protein.postranslational modification
Tb10.70.2070	protein kinase, putative; mitogen-activated protein kinase, putative	29.4 protein.postranslational modification
Tb10.61.2490	protein kinase, putative; serine/threonine protein kinase, putative	29.4 protein.postranslational modification
Tb11.02.2050	protein kinase, putative; serine/threonine protein kinase, putative	29.4 protein.postranslational modification
Tb05.30H13.1000	protein phosphatase 2C, putative	29.4 protein.postranslational modification
Tb07.5F10.630	protein phosphatase 2C, putative	29.4 protein.postranslational modification
Tb11.03.0390	protein phosphatase, putative; protein phosphatase 2c, putative	29.4 protein.postranslational modification
Tb10.6k15.2720	protein-L-isoaspartate, putative	29.4 protein.postranslational modification
Tb927.1.4050	Ser/Thr protein phosphatase, putative	29.4 protein.postranslational modification
Tb10.05.0110	serine/threonine protein phosphatase type 5	29.4 protein.postranslational modification
Tb06.3D8.430	serine/threonine protein phosphatase, putative	29.4 protein.postranslational modification
Tb04.26G5.380	serine/threonine-protein phosphatase PP1, putative	29.4 protein.postranslational modification
Tb08.10K10.880	NRKB serine/threonine-protein kinase NrkA	29.4 protein.postranslational modification
Tb07.2F2.640	tbplk protein kinase, putative; polo-like protein kinase, putative	29.4 protein.postranslational modification
Tb11.02.1070	aminopeptidase, putative; metallo-peptidase, Clan MA(E) Family M1	29.5 protein.degradation
Tb08.30P3.40	aminopeptidase, putative; metallo-peptidase, Clan MA(E) Family M1, putative	29.5 protein.degradation
Tb11.02.4440	aminopeptidase, putative; metallo-peptidase, Clan MF, Family M17	29.5 protein.degradation
Tb11.02.0100	carboxypeptidase, putative; metallo-peptidase, Clan MA(E) Family M32	29.5 protein.degradation
Tb03.3K10.340	katanin-like protein; serine peptidase, Clan SJ, family S16, putative	29.5 protein.degradation
Tb11.02.1370	katanin-like protein; serine peptidase, Clan SJ, family S16, putative	29.5 protein.degradation
Tb10.389.1480	metalloprotease, putative; peptidase (M20/M25/M40 family), putative	29.5 protein.degradation
Tb10.61.1210	methionine aminopeptidase, putative; metallo- peptidase, Clan MG, Family M24	29.5 protein.degradation
Tb11.52.0003	OPB oligopeptidase b; serine peptidase, clan SC, family S9A-like protein	29.5 protein.degradation
Tb10.6k15.2520	prolyl oligopeptidase, putative; serine peptidase clan SC, family S9A, putative	29.5 protein.degradation
Tb03.26J7.400	subtilisin-like serine peptidase; serine peptidase, clan SB, family S8-like protein	29.5 protein.degradation
Tb11.02.1280	subtilisin-like serine peptidase; serine peptidase, clan SB, family S8-like protein	29.5 protein.degradation
TB05.28F8.230	mitochondrial processing peptidase, beta subunit, putative; metallo-peptidase	29.5 protein.degradation
Tb10.6k15.3800	dipeptidyl-peptidase 8-like serine peptidase; serine peptidase, Family S9B	29.5 protein.degradation
Tb11.01.2000	hs1vu complex proteolytic subunit-like protein, putative;	29.5 protein.degradation
Tb07.8P12.250	thimet oligopeptidase, putative; metallo-peptidase, Clan MA(E) Family M3	29.5 protein.degradation

Tb03.48K5.520	ubiquitin hydrolase, putative; cysteine peptidase, Clan CA, family C19, putative	29.5.11 protein.degradation.ubiquitin
Tb09.160.4020	ubiquitin hydrolase, putative; cysteine peptidase, Clan CA, family C19, putative	29.5.11 protein.degradation.ubiquitin
Tb09.211.3610	ubiquitin-activating enzyme E1, putative	29.5.11 protein.degradation.ubiquitin
Tb04.2H8.480	ubiquitin-conjugating enzyme E2, putative	29.5.11 protein.degradation.ubiquitin
Tb11.02.0815	ubiquitin-conjugating enzyme, putative	29.5.11 protein.degradation.ubiquitin
Tb10.6k15.2090	ubiquitin-protein ligase, putative	29.5.11 protein.degradation.ubiquitin
Tb11.01.1680	polyubiquitin, putative	29.5.11.1 protein.degradation.ubiquitin.ubiquitin
Tb10.70.0790	PRCE proteasome beta 5 subunit, putative; proteasome beta 5 subunit	29.5.11.20 protein.degradation.ubiquitin.proteasom
Tb10.70.3660	proteasome activator protein PA26	29.5.11.20 protein.degradation.ubiquitin.proteasom
Tb10.70.0850	proteasome alpha 1 subunit, putative	29.5.11.20 protein.degradation.ubiquitin.proteasom
Tb10.100.0170	proteasome alpha 2 subunit, putative	29.5.11.20 protein.degradation.ubiquitin.proteasom
Tb07.26A24.1040	proteasome alpha 3 subunit, putative	29.5.11.20 protein.degradation.ubiquitin.proteasom
Tb10.100.0120	proteasome alpha 5 subunit, putative; 20S proteasome subunit alpha 5	29.5.11.20 protein.degradation.ubiquitin.proteasom
Tb09.211.2590	proteasome beta 2 subunit, putative; 20S proteasome subunit	29.5.11.20 protein.degradation.ubiquitin.proteasom
Tb07.26A24.340	proteasome beta 6 subunit, putative; 20S proteasome beta 6 subunit, putative	29.5.11.20 protein.degradation.ubiquitin.proteasom
Tb07.22O10.570	proteasome regulatory ATPase subunit 1	29.5.11.20 protein.degradation.ubiquitin.proteasom
Tb11.02.4870	PSA4 proteasome alpha 7 subunit, putative	29.5.11.20 protein.degradation.ubiquitin.proteasom
Tb03.5L5.730	RPN1 26S proteasome regulatory non-ATPase subunit	29.5.11.20 protein.degradation.ubiquitin.proteasom
Tb927.2.2440	RPN6 proteasome regulatory non-ATPase subunit 6, putative	29.5.11.20 protein.degradation.ubiquitin.proteasom
Tb10.70.4510	RPN11 proteasome regulatory non-ATP-ase subunit 11;	29.5.11.20 protein.degradation.ubiquitin.proteasom
Tb06.3A7.810	TbPSB1 proteasome beta-1 subunit, putative	29.5.11.20 protein.degradation.ubiquitin.proteasom
Tb11.01.3260	cullin 2, putative	29.5.11.4.3.3 protein.degradation.ubiquitin
Tb06.2N9.710	cyclosome subunit, possible, conserved	29.5.11.4.4 protein.degradation.ubiquitin ou 31.2 cell.division
Tb927.1.2260	calpain-like protein fragment, putative	29.5.3 protein.degradation.cysteine protease
Tb07.5F10.550	calpain-like cysteine peptidase, putative; cysteine peptidase, Clan CA, family C2,	29.5.3 protein.degradation.cysteine protease
Tb07.5F10.560	calpain-like cysteine peptidase, putative; cysteine peptidase, Clan CA, family C2,	29.5.3 protein.degradation.cysteine protease
Tb11.01.5800	calpain-like cysteine peptidase, putative; cysteine peptidase, Clan CA, family C2,	29.5.3 protein.degradation.cysteine protease
Tb11.01.6870	calpain-like cysteine peptidase, putative; cysteine peptidase, Clan CA, family C2,	29.5.3 protein.degradation.cysteine protease
Tb11.02.1480	mitochondrial processing peptidase alpha subunit, putative; metallo-peptidase,	29.5.3 protein.degradation.cysteine protease
Tb927.1.2100	calpain-like cysteine peptidase, putative; cysteine peptidase, Clan CA,	29.5.3 protein.degradation.cysteine protease
Tb03.1J15.400	AAA ATPase, putative	29.5.9 protein.degradation.AAA type
Tb11.01.4940	AAA family ATPase, putative	29.5.9 protein.degradation.AAA type
Tb11.02.5450	glucose-regulated protein 78, putative	29.6 protein.(un)folding
Tb11.02.5500	glucose-regulated protein 78, putative	29.6 protein.(un)folding
Tb07.29K4.130	chaperone protein DNAJ, putative	29.6 protein.(un)folding
Tb08.29O4.50	chaperone protein DNAJ, putative	29.6 protein.(un)folding

Tb09.211.3680	chaperone protein DNAJ, putative	29.6 protein.(un)folding
Tb10.6k15.0460	chaperone protein DNAJ, putative	29.6 protein.(un)folding
Tb927.2.5160	chaperone protein DnaJ, putative	29.6 protein.(un)folding
Tb11.01.8510	chaperonin alpha subunit	29.6 protein.(un)folding
Tb11.01.5860	chaperonin containing t-complex protein, putative	29.6 protein.(un)folding
Tb11.02.0750	chaperonin TCP20, putative	29.6 protein.(un)folding
Tb10.6k15.2330	chaperonin, putative; T-complex protein 1 (theta subunit), putative	29.6 protein.(un)folding
Tb08.28L1.90	chaperonin/T-complex protein 1 gamma subunit, putative	29.6 protein.(un)folding
Tb06.4M18.480	co-chaperone GrpE, putative	29.6 protein.(un)folding
Tb07.26A24.370	cyclophilin-type peptidyl-prolyl cis-trans isomerase, putative	29.6 protein.(un)folding
Tb11.03.0250	CYPA cyclophilin a	29.6 protein.(un)folding
Tb11.02.3430	DNAJ domain protein, putative	29.6 protein.(un)folding
Tb11.01.3140	heat shock 70 kDa protein (C-terminal fragment), putative	29.6 protein.(un)folding
Tb11.01.3135	heat shock 70 kDa protein (N-terminal fragment), putative	29.6 protein.(un)folding
Tb06.4F7.670	heat shock 70 kDa protein, mitochondrial precursor, putative	29.6 protein.(un)folding
Tb06.4F7.750	heat shock 70 kDa protein, mitochondrial precursor, putative	29.6 protein.(un)folding
Tb03.3K10.400	heat shock protein 20, putative	29.6 protein.(un)folding
Tb11.01.3110	heat shock protein 70	29.6 protein.(un)folding
Tb11.01.3120	heat shock protein 70	29.6 protein.(un)folding
Tb11.01.3080	heat shock protein 70, putative	29.6 protein.(un)folding
Tb10.26.1080	heat shock protein 83; heat shock protein	29.6 protein.(un)folding
Tb07.43M14.520	heat shock protein DnaJ, putative	29.6 protein.(un)folding
Tb10.389.0880	heat shock protein, putative	29.6 protein.(un)folding
Tb11.02.0250	heat shock protein, putative	29.6 protein.(un)folding
Tb07.27M11.580	HSP10 10 kDa heat shock protein, putative	29.6 protein.(un)folding
Tb10.70.0280	HSP60 chaperonin Hsp60, mitochondrial precursor	29.6 protein.(un)folding
Tb10.70.0430	HSP60 chaperonin Hsp60, mitochondrial precursor	29.6 protein.(un)folding
Tb07.29K4.620	HSP70 heat shock 70 kDa protein, putative	29.6 protein.(un)folding
Tb10.70.2600	peptidyl-prolyl cis-trans isomerase, putative	29.6 protein.(un)folding
Tb07.22O10.720	prefoldin, putative	29.6 protein.(un)folding
Tb07.8P12.860	prefoldin, putative	29.6 protein.(un)folding
Tb07.27M11.560	protein disulfide isomerase, putative	29.6 protein.(un)folding
Tb10.6k15.2290	protein disulfide isomerase; bloodstream- specific protein 2 precursor	29.6 protein.(un)folding
Tb03.28C22.1060	lipophosphoglycan biosynthetic protein, putative; heat shock protein 90, putative;	29.6 protein.(un)folding
Tb09.211.2570	t-complex protein 1, eta subunit, putative;	29.6 protein.(un)folding
Tb11.01.4050	heat shock protein HslVU, ATPase subunit HslU, putative;	29.6 protein.(un)folding

Tb927.2.5980	HSP100 ATP-dependent Clp protease subunit, heat shock protein 100 (HSP100),	29.6 protein.(un)folding
Tb11.42.0003	T-complex protein 1, beta subunit, putative	29.6 protein.(un)folding
Tb10.70.7050	t-complex protein 1, delta subunit, putative	29.6 protein.(un)folding
Tb09.211.1510	SNAP50 small nuclear RNA gene activation protein (SNAP) 50, putative;	30 signalling
Tb04.3M17.390	calreticulin, putative	30.3 signaling.calcium
Tb09.211.2540	calmodulin-like protein; EF hand containing protein	30.3 signalling.calcium
Tb04.5D20.520	phosphatidylinositol 3-kinase tor, putative	30.4 signalling.phosphinositides
Tb927.1.1930	phosphatidylinositol 3-kinase, putative	30.4 signalling.phosphinositides
Tb04.1H19.200	GTP-binding protein, putative	30.5 signalling.G-proteins
Tb07.29D18.100	GTP-binding protein, putative	30.5 signalling.G-proteins
Tb07.8P12.880	GTP-binding protein, putative	30.5 signalling.G-proteins
Tb09.211.0720	GTP-binding protein, putative	30.5 signalling.G-proteins
Tb11.52.0014	ras-related GTP-binding protein, putative	30.5 signalling.G-proteins
Tb03.27F10.90	rtb2 GTP-binding nuclear protein rtb2, putative	30.5 signalling.G-proteins
Tb10.70.6420	small GTPase, putative; similar to Ras-related protein Rab-21	30.5 signalling.G-proteins
Tb11.02.2310	prostaglandin f synthase	30.99 signalling.unspecified
Tb11.02.0310	cell division cycle 45 (CDC45), putative	31.3 cell.cycle
Tb08.30K1.260	cell division cycle protein, putative	31.3 cell.cycle
Tb927.1.3230	cell division cycle protein, putative	31.3 cell.cycle
Tb10.70.7040	CRK1 cell division protein kinase 2; cdc2-like protein kinase	31.3 cell.cycle
Tb927.1.3220	GTPase activating protein of Rab-like GTPase, putative	31.4 cell. vesicle transport
Tb09.211.0620	actin	31.1 cell.organisation
Tb10.389.0270	actin related protein 2/3 complex, putative; ARP2/3 complex subunit, putative	31.1 cell.organisation
Tb09.160.3960	actin-like protein, putative	31.1 cell.organisation
Tb09.160.3850	actin-related protein 3, putative	31.1 cell.organisation
Tb927.1.2340	alpha tubulin	31.1 cell.organisation
Tb927.1.2360	alpha tubulin	31.1 cell.organisation
Tb927.1.2400	alpha tubulin	31.1 cell.organisation
Tb927.1.2330	beta tubulin	31.1 cell.organisation
Tb927.1.2350	beta tubulin	31.1 cell.organisation
Tb927.1.2390	beta tubulin	31.1 cell.organisation
Tb03.5L5.210	beta tubulin	31.1 cell.organisation
Tb10.70.6960	cofilin/actin depolymerizing factor, putative	31.1 cell.organisation
Tb08.6H23.110	dynein arm light chain, putative	31.1 cell.organisation
Tb11.02.2640	dynein heavy chain, cytosolic, putative	31.1 cell.organisation
Tb10.70.2520	dynein intermediate-chain-like	31.1 cell.organisation

Tb11.02.3390	dynein light chain, putative	31.1 cell.organisation
Tb09.211.4920	dynein light chain, putative	31.1 cell.organisation
Tb10.70.6950	dynein-associated protein, putative	31.1 cell.organisation
Tb11.02.3470	epsilon tubulin	31.1 cell.organisation
Tb03.27F10.510	formin, putative; formin-like protein	31.1 cell.organisation
Tb927.1.1350	gamma-tubulin	31.1 cell.organisation
Tb03.48K5.730	kinesin heavy chain, putative	31.1 cell.organisation
Tb04.2H8.1400	kinesin, putative	31.1 cell.organisation
Tb05.3C6.520	kinesin, putative	31.1 cell.organisation
Tb06.28P18.680	kinesin, putative	31.1 cell.organisation
Tb07.13M20.410	kinesin, putative	31.1 cell.organisation
Tb08.26A17.630	kinesin, putative	31.1 cell.organisation
Tb08.30K1.750	kinesin, putative	31.1 cell.organisation
Tb08.4A8.660	kinesin, putative	31.1 cell.organisation
Tb10.61.0990	kinesin, putative	31.1 cell.organisation
Tb10.61.1020	kinesin, putative	31.1 cell.organisation
Tb10.70.6990	kinesin, putative	31.1 cell.organisation
Tb10.70.7260	kinesin, putative	31.1 cell.organisation
Tb11.01.2530	kinesin, putative	31.1 cell.organisation
Tb08.10K10.440	kinesin-like protein	31.1 cell.organisation
Tb09.211.4511	kinetoplast-associated protein, putative	31.1 cell.organisation
Tb09.211.4512	kinetoplastid membrane protein KMP-11	31.1 cell.organisation
Tb09.211.4513	kinetoplastid membrane protein KMP-11	31.1 cell.organisation
Tb11.02.2970	kinetoplastid membrane protein KMP-11	31.1 cell.organisation
Tb06.4M18.380	MCAK-like kinesin, putative	31.1 cell.organisation
Tb11.01.5350	nuclear movement protein, putative; NUDC-like protein	31.1 cell.organisation
Tb06.5F5.840	profilin	31.1 cell.organisation
Tb10.61.1750	microtubule-associated protein, putative;	31.1 cell.organisation
Tb08.11J15.520	TBKIFC1 C-terminal motor kinesin, putative	31.1 cell.organisation
Tb10.61.3050	tubulin folding cofactor D, putative	31.1 cell.organisation
Tb09.211.1170	tubulin tyrosine ligase protein, putative	31.1 cell.organisation
Tb10.61.1030	tubulin tyrosine ligase, putative	31.1 cell.organisation
Tb08.4A8.510	U3 snoRNA-associated protein UTP11, putative	31.1 cell.organisation
Tb10.6k15.0060	prohibitin	31.3 cell.cycle
Tb10.70.0830	procyclin-associated gene 2 (PAG2) polypeptide, putative	31.3 cell.cycle
Tb10.61.1380	CHC clathrin heavy chain	31.4 cell. vesicle transport

Tb11.02.3170	synaptobrevin-type transport protein, putative	31.4 cell. vesicle transport
Tb10.6k15.1160	synaptojanin (N-terminal domain), putative	31.4 cell. vesicle transport
Tb09.211.0740	G-actin binding protein, putative; CAP/Srv2p, putative	31.6* cell organization-cytoskeleton/myosin/actin/tubulin/movement
Tb05.45E22.830	p21 antigen protein, putative	31.6* cell organization-cytoskeleton/myosin/actin/tubulin/movement
Tb11.01.5100	paraflagellar rod component Par4, putative	31.6* cell organization-cytoskeleton/myosin/actin/tubulin/movement
Tb08.5H5.880	paraflagellar rod component, putative	31.6* cell organization-cytoskeleton/myosin/actin/tubulin/movement
Tb08.5H5.920	PFR 69 kDa paraflagellar rod protein	31.6* cell organization-cytoskeleton/myosin/actin/tubulin/movement
Tb08.5H5.890	PFR 69 kDa paraflagellar rod protein	31.6* cell organization-cytoskeleton/myosin/actin/tubulin/movement
Tb03.26J7.510	PFR-A; PFR-B 69 kDa paraflagellar rod protein	31.6* cell organization-cytoskeleton/myosin/actin/tubulin/movement
Tb03.26J7.550	PFR-C; PFR-D 73 kDa paraflagellar rod protein	31.6* cell organization-cytoskeleton/myosin/actin/tubulin/movement
Tb10.1760	PFR-C; PFR-D 73 kDa paraflagellar rod protein	31.6* cell organization-cytoskeleton/myosin/actin/tubulin/movement
Tb08.11J15.740	Tb10.1760 Tb10.1760 tubulin tyrosine ligase-like protein, putative 51340:52644	31.6* cell organization-cytoskeleton/myosin/actin/tubulin/movement
Tb11.47.0001	TBBC basal body component; ML protein	31.6* cell organization-cytoskeleton/myosin/actin/tubulin/movement
Tb05.29K2.280	65 kDa invariant surface glycoprotein-like protein	31.99 cell.unspecified
Tb09.0470	ISG75 75 kDa invariant surface glycoprotein, putative	31.99 cell.unspecified
Tb09.0770	Tb09.0470 variant surface glycoprotein (VSG), 250794:252272 forward	31.99 cell.unspecified
Tb10.0300	Tb09.0770 variant surface glycoprotein 367396:368943 reverse MW:55489	31.99 cell.unspecified
Tb10.0370	Tb10.0300 variant surface glycoprotein (VSG), 609880:611313 forward	31.99 cell.unspecified
Tb10.06.0240	Tb10.0370 variant surface glycoprotein (VSG), 400494:402077 reverse	31.99 cell.unspecified
Tb10.0610	Tb10.06.0240 variant surface glycoprotein (VSG), 755939:757411 forward	31.99 cell.unspecified
Tb11.0230	Tb10.0610 variant surface glycoprotein (VSG), 635061:636599 forward	31.99 cell.unspecified
Tb05.25P15.230	Tb11.0230 variant surface glycoprotein (VSG), 108740:110302 forward	31.99 cell.unspecified
Tb05.26C7.330	variant surface glycoprotein (VSG), putative	31.99 cell.unspecified
Tb05.5M21.150	variant surface glycoprotein (VSG), putative	31.99 cell.unspecified
Tb05.5M21.20	variant surface glycoprotein (VSG), putative	31.99 cell.unspecified
Tb06.1I18.610	variant surface glycoprotein (VSG), putative	31.99 cell.unspecified
Tb07.15M23.100	variant surface glycoprotein (VSG), putative	31.99 cell.unspecified
Tb08.25E9.50	variant surface glycoprotein (VSG), putative	31.99 cell.unspecified
Tb09.244.0640	variant surface glycoprotein (VSG), putative	31.99 cell.unspecified
Tb09.244.1580	variant surface glycoprotein (VSG), putative	31.99 cell.unspecified
Tb11.14.0015	variant surface glycoprotein (VSG), putative	31.99 cell.unspecified
Tb927.1.5300	variant surface glycoprotein (VSG), putative	31.99 cell.unspecified
Tb927.1.5330	variant surface glycoprotein (VSG), putative	31.99 cell.unspecified
Tb11.14.0008	variant surface glycoprotein (VSG), putative	31.99 cell.unspecified
Tb11.24.0012	variant surface glycoprotein (VSG, atypical), putative	31.99 cell.unspecified
Tb11.38.0005	variant surface glycoprotein (VSG, atypical), putative	31.99 cell.unspecified

Tb11.55.0006	variant surface glycoprotein (VSG, atypical), putative	31.99 cell.unspecified
Tb11.55.0014	intraflagellar transport protein IFT88, putative	**34** transport
Tb10.70.4750	vesicular transport protein (CDC48 homologue), putative	34 transport
Tb06.2N9.700	calcium channel protein, putative	34 transport
Tb11.01.5040	intraflagellar transport (IFT) protein, putative	34 transport
Tb11.02.0850	mitochondrial carrier protein, putative	34 transport
Tb07.5F10.810	phospholipid-transporting ATPase 1-like protein, putative	34 transport
Tb08.26N11.730	Tb07.5F10.810 ADP/ATP mitochondrial carrier protein, putative;	34 transport
Tb08.26N11.310	TbMRPA multidrug resistance protein A	34 transport
Tb07.30D13.350	tGLP1 Golgi/lysosome glycoprotein 1	34 transport
Tb03.1J15.500	ATP synthase alpha chain, mitochondrial precursor;	34.1 transport.p- and v-ATPases
Tb10.100.0070	ATP synthase beta chain, mitochondrial precursor;	34.1 transport.p- and v-ATPases
Tb06.30P15.500	ATP synthase F1 subunit gamma protein, putative	34.1 transport.p- and v-ATPases
Tb11.02.4210	ATP synthase, epsilon chain, putative	34.1 transport.p- and v-ATPases
Tb10.70.3600	ATPase, putative	34.1 transport.p- and v-ATPases
Tb11.55.0012	vacuolar ATP synthase subunit D, putative	34.1 transport.p- and v-ATPases
Tb04.5E12.370	vesicular-fusion ATPase-like protein, putative	34.1 transport.p- and v-ATPases
Tb10.389.1170	V-type ATPase, A subunit, putative	34.1 transport.p- and v-ATPases
Tb04.2L9.1150	P-type H+-ATPase, putative	34.1.1 transport.p- and v-ATPases.H+-transporting two-sector ATPase
Tb11.02.0630	ADP/ATP mitochondrial carrier protein, putative	34.10 transport.nucleotides
Tb11.02.3950	ABC transporter, putative	34.16 transport.ABC transporters and multidrug resistance systems
Tb04.1D20.560	ABC transporter, putative	34.16 transport.ABC transporters and multidrug resistance systems
Tb03.1J15.280	amino acid transporter, putative	34.3 transport.amino acids
Tb03.48K5.300	leucine-rich repeat protein (LRRP), putative	**35** not assigned
Tb11.01.4390	leucine-rich repeat protein (LRRP), putative	**35** not assigned
Tb927.1.290	leucine-rich repeat protein (LRRP), putative	**35** not assigned
Tb927.1.370	leucine-rich repeat protein (LRRP), putative	**35** not assigned
Tb927.1.480	leucine-rich repeat protein (LRRP), putative	**35** not assigned
Tb10.406.0260	leucine-rich repeat protein (LRRP), putative	**35** not assigned
Tb09.160.2540	iron-containing hydrogenase, putative	35 not assigned
Tb09.244.2600	alpha/beta-hydrolase-like protein	35.1 not assigned.no ontology
Tb03.1J15.360	ankyrin-repeat protein, putative	35.1 not assigned.no ontology
Tb03.1J15.420	hypothetical protein	35.1 not assigned.no ontology
Tb03.5L5.410	hypothetical protein	35.1 not assigned.no ontology
Tb03.5L5.720	hypothetical protein	35.1 not assigned.no ontology
Tb04.1D20.10	hypothetical protein	35.1 not assigned.no ontology

Tb04.1H19.220	hypothetical protein	35.1 not assigned.no ontology
Tb04.30O21.100	hypothetical protein	35.1 not assigned.no ontology
Tb04.3I12.110	hypothetical protein	35.1 not assigned.no ontology
Tb04.3I12.580	hypothetical protein	35.1 not assigned.no ontology
Tb04.3I12.660	hypothetical protein	35.1 not assigned.no ontology
Tb04.3I12.950	hypothetical protein	35.1 not assigned.no ontology
Tb05.30H13.1040	hypothetical protein	35.1 not assigned.no ontology
Tb05.30H13.500	hypothetical protein	35.1 not assigned.no ontology
Tb06.3D8.840	hypothetical protein	35.1 not assigned.no ontology
Tb07.21H15.100	hypothetical protein	35.1 not assigned.no ontology
Tb07.21H15.20	hypothetical protein	35.1 not assigned.no ontology
Tb07.21H15.290	hypothetical protein	35.1 not assigned.no ontology
Tb07.21H15.30	hypothetical protein	35.1 not assigned.no ontology
Tb07.26A24.450	hypothetical protein	35.1 not assigned.no ontology
Tb07.27E10.220	hypothetical protein	35.1 not assigned.no ontology
Tb07.27E10.520	hypothetical protein	35.1 not assigned.no ontology
Tb07.2F2.560	hypothetical protein	35.1 not assigned.no ontology
Tb07.30D13.260	hypothetical protein	35.1 not assigned.no ontology
Tb07.8P12.330	hypothetical protein	35.1 not assigned.no ontology
Tb08.12O16.630	hypothetical protein	35.1 not assigned.no ontology
Tb08.26A17.170	hypothetical protein	35.1 not assigned.no ontology
Tb08.26A17.710	hypothetical protein	35.1 not assigned.no ontology
Tb08.26N11.130	hypothetical protein	35.1 not assigned.no ontology
Tb08.26N11.590	hypothetical protein	35.1 not assigned.no ontology
Tb08.6H23.120	hypothetical protein	35.1 not assigned.no ontology
Tb08.6H23.220	hypothetical protein	35.1 not assigned.no ontology
Tb08.6H23.340	hypothetical protein	35.1 not assigned.no ontology
Tb08.6H23.430	hypothetical protein	35.1 not assigned.no ontology
Tb09.211.0480	hypothetical protein	35.1 not assigned.no ontology
Tb09.211.4300	hypothetical protein	35.1 not assigned.no ontology
Tb09.211.4560	hypothetical protein	35.1 not assigned.no ontology
Tb09.211.4730	hypothetical protein	35.1 not assigned.no ontology
Tb09.244.2050	hypothetical protein	35.1 not assigned.no ontology
Tb10.26.0050	hypothetical protein	35.1 not assigned.no ontology
Tb10.26.0680	hypothetical protein	35.1 not assigned.no ontology
Tb10.26.0950	hypothetical protein	35.1 not assigned.no ontology

Tb10.389.0290	hypothetical protein	35.1 not assigned.no ontology
Tb10.389.0310	hypothetical protein	35.1 not assigned.no ontology
Tb10.389.0660	hypothetical protein	35.1 not assigned.no ontology
Tb10.389.1370	hypothetical protein	35.1 not assigned.no ontology
Tb10.389.1630	hypothetical protein	35.1 not assigned.no ontology
Tb10.389.1900	hypothetical protein	35.1 not assigned.no ontology
Tb10.406.0160	hypothetical protein	35.1 not assigned.no ontology
Tb10.61.0620	hypothetical protein	35.1 not assigned.no ontology
Tb10.61.1060	hypothetical protein	35.1 not assigned.no ontology
Tb10.6k15.1720	hypothetical protein	35.1 not assigned.no ontology
Tb10.6k15.2980	hypothetical protein	35.1 not assigned.no ontology
Tb10.70.1910	hypothetical protein	35.1 not assigned.no ontology
Tb10.70.4860	hypothetical protein	35.1 not assigned.no ontology
Tb10.70.5580	hypothetical protein	35.1 not assigned.no ontology
Tb10.70.7640	hypothetical protein	35.1 not assigned.no ontology
Tb11.01.5090	hypothetical protein	35.1 not assigned.no ontology
Tb11.01.6520	hypothetical protein	35.1 not assigned.no ontology
Tb11.01.6560	hypothetical protein	35.1 not assigned.no ontology
Tb11.01.8020	hypothetical protein	35.1 not assigned.no ontology
Tb11.02.0354	hypothetical protein	35.1 not assigned.no ontology
Tb11.02.0720	hypothetical protein	35.1 not assigned.no ontology
Tb11.02.2670	hypothetical protein	35.1 not assigned.no ontology
Tb11.02.2690	hypothetical protein	35.1 not assigned.no ontology
Tb11.02.3250	hypothetical protein	35.1 not assigned.no ontology
Tb927.1.2750	hypothetical protein	35.1 not assigned.no ontology
Tb927.1.4280	hypothetical protein	35.1 not assigned.no ontology
Tb927.1.4580	hypothetical protein	35.1 not assigned.no ontology
Tb927.2.5760	hypothetical protein	35.1 not assigned.no ontology
Tb927.2.6170	hypothetical protein	35.1 not assigned.no ontology
Tb03.1J15.700	hypothetical protein	35.1 not assigned.no ontology
Tb03.25B21.20	hypothetical protein, conserved	35.1 not assigned.no ontology
Tb03.26J7.720	hypothetical protein, conserved	35.1 not assigned.no ontology
Tb03.26J7.870	hypothetical protein, conserved	35.1 not assigned.no ontology
Tb03.27C5.160	hypothetical protein, conserved	35.1 not assigned.no ontology
Tb03.27C5.510	hypothetical protein, conserved	35.1 not assigned.no ontology
Tb03.27C5.640	hypothetical protein, conserved	35.1 not assigned.no ontology

Tb03.27F10.610	hypothetical protein, conserved	35.1 not assigned.no ontology
Tb03.28C22.770	hypothetical protein, conserved	35.1 not assigned.no ontology
Tb03.28C22.80	hypothetical protein, conserved	35.1 not assigned.no ontology
Tb03.28C22.940	hypothetical protein, conserved	35.1 not assigned.no ontology
Tb03.30P12.1130	hypothetical protein, conserved	35.1 not assigned.no ontology
Tb03.30P12.280	hypothetical protein, conserved	35.1 not assigned.no ontology
Tb03.30P12.450	hypothetical protein, conserved	35.1 not assigned.no ontology
Tb03.30P12.590	hypothetical protein, conserved	35.1 not assigned.no ontology
Tb03.30P12.690	hypothetical protein, conserved	35.1 not assigned.no ontology
Tb03.30P12.70	hypothetical protein, conserved	35.1 not assigned.no ontology
Tb03.30P12.80	hypothetical protein, conserved	35.1 not assigned.no ontology
Tb03.30P12.810	hypothetical protein, conserved	35.1 not assigned.no ontology
Tb03.3K10.420	hypothetical protein, conserved	35.1 not assigned.no ontology
Tb03.48K5.610	hypothetical protein, conserved	35.1 not assigned.no ontology
Tb03.48K5.660	hypothetical protein, conserved	35.1 not assigned.no ontology
Tb03.48K5.80	hypothetical protein, conserved	35.1 not assigned.no ontology
Tb03.48K5.800	hypothetical protein, conserved	35.1 not assigned.no ontology
Tb03.48O8.200	hypothetical protein, conserved	35.1 not assigned.no ontology
Tb03.48O8.40	hypothetical protein, conserved	35.1 not assigned.no ontology
Tb03.48O8.520	hypothetical protein, conserved	35.1 not assigned.no ontology
Tb03.48O8.620	hypothetical protein, conserved	35.1 not assigned.no ontology
Tb03.5L5.480	hypothetical protein, conserved	35.1 not assigned.no ontology
Tb03.5L5.670	hypothetical protein, conserved	35.1 not assigned.no ontology
Tb04.1H19.1150	hypothetical protein, conserved	35.1 not assigned.no ontology
Tb04.26G5.100	hypothetical protein, conserved	35.1 not assigned.no ontology
Tb04.29M18.550	hypothetical protein, conserved	35.1 not assigned.no ontology
Tb04.29M18.710	hypothetical protein, conserved	35.1 not assigned.no ontology
Tb04.29M18.770	hypothetical protein, conserved	35.1 not assigned.no ontology
Tb04.2H8.1000	hypothetical protein, conserved	35.1 not assigned.no ontology
Tb04.2H8.1270	hypothetical protein, conserved	35.1 not assigned.no ontology
Tb04.2H8.400	hypothetical protein, conserved	35.1 not assigned.no ontology
Tb04.2H8.580	hypothetical protein, conserved	35.1 not assigned.no ontology
Tb04.2L9.440	hypothetical protein, conserved	35.1 not assigned.no ontology
Tb04.3M17.590	hypothetical protein, conserved	35.1 not assigned.no ontology
Tb04.5E12.1130	hypothetical protein, conserved	35.1 not assigned.no ontology
Tb04.5E12.1170	hypothetical protein, conserved	35.1 not assigned.no ontology

Tb04.5E12.820	hypothetical protein, conserved	35.1 not assigned.no ontology
Tb05.1P6.530	hypothetical protein, conserved	35.1 not assigned.no ontology
Tb05.26K5.220	hypothetical protein, conserved	35.1 not assigned.no ontology
Tb05.26K5.300	hypothetical protein, conserved	35.1 not assigned.no ontology
Tb05.26K5.540	hypothetical protein, conserved	35.1 not assigned.no ontology
Tb05.26K5.670	hypothetical protein, conserved	35.1 not assigned.no ontology
Tb05.26K5.730	hypothetical protein, conserved	35.1 not assigned.no ontology
Tb05.27M3.280	hypothetical protein, conserved	35.1 not assigned.no ontology
Tb05.27M3.370	hypothetical protein, conserved	35.1 not assigned.no ontology
TB05.28F8.310	hypothetical protein, conserved	35.1 not assigned.no ontology
TB05.28F8.480	hypothetical protein, conserved	35.1 not assigned.no ontology
Tb05.29K2.440	hypothetical protein, conserved	35.1 not assigned.no ontology
Tb05.30F7.330	hypothetical protein, conserved	35.1 not assigned.no ontology
Tb05.30H13.120	hypothetical protein, conserved	35.1 not assigned.no ontology
Tb05.30H13.210	hypothetical protein, conserved	35.1 not assigned.no ontology
Tb05.30H13.220	hypothetical protein, conserved	35.1 not assigned.no ontology
Tb05.30H13.330	hypothetical protein, conserved	35.1 not assigned.no ontology
Tb05.30H13.350	hypothetical protein, conserved	35.1 not assigned.no ontology
Tb05.30H13.360	hypothetical protein, conserved	35.1 not assigned.no ontology
Tb05.30H13.710	hypothetical protein, conserved	35.1 not assigned.no ontology
Tb05.30H13.940	hypothetical protein, conserved	35.1 not assigned.no ontology
Tb05.3C6.120	hypothetical protein, conserved	35.1 not assigned.no ontology
Tb05.3C6.130	hypothetical protein, conserved	35.1 not assigned.no ontology
Tb05.3C6.250	hypothetical protein, conserved	35.1 not assigned.no ontology
Tb05.3C6.330	hypothetical protein, conserved	35.1 not assigned.no ontology
Tb05.3C6.60	hypothetical protein, conserved	35.1 not assigned.no ontology
Tb05.3C6.740	hypothetical protein, conserved	35.1 not assigned.no ontology
Tb05.45E22.390	hypothetical protein, conserved	35.1 not assigned.no ontology
Tb05.45E22.410	hypothetical protein, conserved	35.1 not assigned.no ontology
Tb05.45E22.650	hypothetical protein, conserved	35.1 not assigned.no ontology
Tb05.45E22.740	hypothetical protein, conserved	35.1 not assigned.no ontology
Tb05.45E22.750	hypothetical protein, conserved	35.1 not assigned.no ontology
Tb05.6E7.1020	hypothetical protein, conserved	35.1 not assigned.no ontology
Tb05.6E7.560	hypothetical protein, conserved	35.1 not assigned.no ontology
Tb05.6E7.590	hypothetical protein, conserved	35.1 not assigned.no ontology
Tb05.6E7.90	hypothetical protein, conserved	35.1 not assigned.no ontology

Tb05.6E7.980	hypothetical protein, conserved	35.1 not assigned.no ontology
Tb06.26G9.150	hypothetical protein, conserved	35.1 not assigned.no ontology
Tb06.26G9.330	hypothetical protein, conserved	35.1 not assigned.no ontology
Tb06.26G9.420	hypothetical protein, conserved	35.1 not assigned.no ontology
Tb06.2N9.100	hypothetical protein, conserved	35.1 not assigned.no ontology
Tb06.2N9.200	hypothetical protein, conserved	35.1 not assigned.no ontology
Tb06.30P15.660	hypothetical protein, conserved	35.1 not assigned.no ontology
Tb06.3A7.200	hypothetical protein, conserved	35.1 not assigned.no ontology
Tb06.3A7.590	hypothetical protein, conserved	35.1 not assigned.no ontology
Tb06.4F7.480	hypothetical protein, conserved	35.1 not assigned.no ontology
Tb06.4F7.600	hypothetical protein, conserved	35.1 not assigned.no ontology
Tb06.4M18.580	hypothetical protein, conserved	35.1 not assigned.no ontology
Tb06.4M18.830	hypothetical protein, conserved	35.1 not assigned.no ontology
Tb06.5F5.340	hypothetical protein, conserved	35.1 not assigned.no ontology
Tb06.5F5.920	hypothetical protein, conserved	35.1 not assigned.no ontology
Tb07.10C21.590	hypothetical protein, conserved	35.1 not assigned.no ontology
Tb07.13M20.180	hypothetical protein, conserved	35.1 not assigned.no ontology
Tb07.13M20.230	hypothetical protein, conserved	35.1 not assigned.no ontology
Tb07.13M20.380	hypothetical protein, conserved	35.1 not assigned.no ontology
Tb07.15M23.240	hypothetical protein, conserved	35.1 not assigned.no ontology
Tb07.15M23.280	hypothetical protein, conserved	35.1 not assigned.no ontology
Tb07.15M23.350	hypothetical protein, conserved	35.1 not assigned.no ontology
Tb07.15M23.550	hypothetical protein, conserved	35.1 not assigned.no ontology
Tb07.15M23.600	hypothetical protein, conserved	35.1 not assigned.no ontology
Tb07.15M23.630	hypothetical protein, conserved	35.1 not assigned.no ontology
Tb07.21H15.130	hypothetical protein, conserved	35.1 not assigned.no ontology
Tb07.22O10.380	hypothetical protein, conserved	35.1 not assigned.no ontology
Tb07.22O10.390	hypothetical protein, conserved	35.1 not assigned.no ontology
Tb07.22O10.790	hypothetical protein, conserved	35.1 not assigned.no ontology
Tb07.22O10.840	hypothetical protein, conserved	35.1 not assigned.no ontology
Tb07.22O10.90	hypothetical protein, conserved	35.1 not assigned.no ontology
Tb07.26A24.190	hypothetical protein, conserved	35.1 not assigned.no ontology
Tb07.26A24.60	hypothetical protein, conserved	35.1 not assigned.no ontology
Tb07.26A24.800	hypothetical protein, conserved	35.1 not assigned.no ontology
Tb07.26A24.810	hypothetical protein, conserved	35.1 not assigned.no ontology
Tb07.27E10.150	hypothetical protein, conserved	35.1 not assigned.no ontology

Tb07.27E10.50	hypothetical protein, conserved	35.1 not assigned.no ontology
Tb07.27E10.570	hypothetical protein, conserved	35.1 not assigned.no ontology
Tb07.27E10.610	hypothetical protein, conserved	35.1 not assigned.no ontology
Tb07.28B13.40	hypothetical protein, conserved	35.1 not assigned.no ontology
Tb07.28B13.470	hypothetical protein, conserved	35.1 not assigned.no ontology
Tb07.28B13.480	hypothetical protein, conserved	35.1 not assigned.no ontology
Tb07.28I8.20	hypothetical protein, conserved	35.1 not assigned.no ontology
Tb07.29K4.430	hypothetical protein, conserved	35.1 not assigned.no ontology
Tb07.29K4.610	hypothetical protein, conserved	35.1 not assigned.no ontology
Tb07.2F2.320	hypothetical protein, conserved	35.1 not assigned.no ontology
Tb07.30D13.320	hypothetical protein, conserved	35.1 not assigned.no ontology
Tb07.33N13.400	hypothetical protein, conserved	35.1 not assigned.no ontology
Tb07.5F10.150	hypothetical protein, conserved	35.1 not assigned.no ontology
Tb07.5F10.420	hypothetical protein, conserved	35.1 not assigned.no ontology
Tb07.8P12.780	hypothetical protein, conserved	35.1 not assigned.no ontology
Tb08.10K10.490	hypothetical protein, conserved	35.1 not assigned.no ontology
Tb08.10K10.600	hypothetical protein, conserved	35.1 not assigned.no ontology
Tb08.10K10.740	hypothetical protein, conserved	35.1 not assigned.no ontology
Tb08.10K10.750	hypothetical protein, conserved	35.1 not assigned.no ontology
Tb08.11J15.380	hypothetical protein, conserved	35.1 not assigned.no ontology
Tb08.11J15.640	hypothetical protein, conserved	35.1 not assigned.no ontology
Tb08.12O16.100	hypothetical protein, conserved	35.1 not assigned.no ontology
Tb08.12O16.280	hypothetical protein, conserved	35.1 not assigned.no ontology
Tb08.12O16.410	hypothetical protein, conserved	35.1 not assigned.no ontology
Tb08.26A17.270	hypothetical protein, conserved	35.1 not assigned.no ontology
Tb08.26A17.570	hypothetical protein, conserved	35.1 not assigned.no ontology
Tb08.26E13.340	hypothetical protein, conserved	35.1 not assigned.no ontology
Tb08.26N11.120	hypothetical protein, conserved	35.1 not assigned.no ontology
Tb08.26N11.430	hypothetical protein, conserved	35.1 not assigned.no ontology
Tb08.26N11.450	hypothetical protein, conserved	35.1 not assigned.no ontology
Tb08.26N11.500	hypothetical protein, conserved	35.1 not assigned.no ontology
Tb08.26N11.820	hypothetical protein, conserved	35.1 not assigned.no ontology
Tb08.28F14.220	hypothetical protein, conserved	35.1 not assigned.no ontology
Tb08.28F14.590	hypothetical protein, conserved	35.1 not assigned.no ontology
Tb08.28L1.140	hypothetical protein, conserved	35.1 not assigned.no ontology
Tb08.28L1.190	hypothetical protein, conserved	35.1 not assigned.no ontology

Tb08.28L1.700	hypothetical protein, conserved	35.1 not assigned.no ontology
Tb08.29H22.550	hypothetical protein, conserved	35.1 not assigned.no ontology
Tb08.29H22.90	hypothetical protein, conserved	35.1 not assigned.no ontology
Tb08.29O4.100	hypothetical protein, conserved	35.1 not assigned.no ontology
Tb08.29O4.200	hypothetical protein, conserved	35.1 not assigned.no ontology
Tb08.29O4.460	hypothetical protein, conserved	35.1 not assigned.no ontology
Tb08.29O4.570	hypothetical protein, conserved	35.1 not assigned.no ontology
Tb08.29O4.640	hypothetical protein, conserved	35.1 not assigned.no ontology
Tb08.29O9.260	hypothetical protein, conserved	35.1 not assigned.no ontology
Tb08.29O9.50	hypothetical protein, conserved	35.1 not assigned.no ontology
Tb08.30P3.10	hypothetical protein, conserved	35.1 not assigned.no ontology
Tb08.4A8.180	hypothetical protein, conserved	35.1 not assigned.no ontology
Tb08.4A8.570	hypothetical protein, conserved	35.1 not assigned.no ontology
Tb08.4A8.650	hypothetical protein, conserved	35.1 not assigned.no ontology
Tb08.5H5.520	hypothetical protein, conserved	35.1 not assigned.no ontology
Tb08.5H5.570	hypothetical protein, conserved	35.1 not assigned.no ontology
Tb08.5H5.580	hypothetical protein, conserved	35.1 not assigned.no ontology
Tb08.5H5.710	hypothetical protein, conserved	35.1 not assigned.no ontology
Tb09.160.0540	hypothetical protein, conserved	35.1 not assigned.no ontology
Tb09.160.0830	hypothetical protein, conserved	35.1 not assigned.no ontology
Tb09.160.1160	hypothetical protein, conserved	35.1 not assigned.no ontology
Tb09.160.1560	hypothetical protein, conserved	35.1 not assigned.no ontology
Tb09.160.2900	hypothetical protein, conserved	35.1 not assigned.no ontology
Tb09.160.3120	hypothetical protein, conserved	35.1 not assigned.no ontology
Tb09.160.3980	hypothetical protein, conserved	35.1 not assigned.no ontology
Tb09.160.4500	hypothetical protein, conserved	35.1 not assigned.no ontology
Tb09.160.4680	hypothetical protein, conserved	35.1 not assigned.no ontology
Tb09.160.5120	hypothetical protein, conserved	35.1 not assigned.no ontology
Tb09.211.0040	hypothetical protein, conserved	35.1 not assigned.no ontology
Tb09.211.0160	hypothetical protein, conserved	35.1 not assigned.no ontology
Tb09.211.0180	hypothetical protein, conserved	35.1 not assigned.no ontology
Tb09.211.0580	hypothetical protein, conserved	35.1 not assigned.no ontology
Tb09.211.0610	hypothetical protein, conserved	35.1 not assigned.no ontology
Tb09.211.0690	hypothetical protein, conserved	35.1 not assigned.no ontology
Tb09.211.1620	hypothetical protein, conserved	35.1 not assigned.no ontology
Tb09.211.1690	hypothetical protein, conserved	35.1 not assigned.no ontology

Tb09.211.1880	hypothetical protein, conserved	35.1 not assigned.no ontology
Tb09.211.1890	hypothetical protein, conserved	35.1 not assigned.no ontology
Tb09.211.1910	hypothetical protein, conserved	35.1 not assigned.no ontology
Tb09.211.2700	hypothetical protein, conserved	35.1 not assigned.no ontology
Tb09.211.2790	hypothetical protein, conserved	35.1 not assigned.no ontology
Tb09.211.3690	hypothetical protein, conserved	35.1 not assigned.no ontology
Tb09.211.3780	hypothetical protein, conserved	35.1 not assigned.no ontology
Tb09.211.3830	hypothetical protein, conserved	35.1 not assigned.no ontology
Tb09.211.3940	hypothetical protein, conserved	35.1 not assigned.no ontology
Tb09.211.4000	hypothetical protein, conserved	35.1 not assigned.no ontology
Tb09.211.4210	hypothetical protein, conserved	35.1 not assigned.no ontology
Tb09.211.4810	hypothetical protein, conserved	35.1 not assigned.no ontology
Tb09.211.4820	hypothetical protein, conserved	35.1 not assigned.no ontology
Tb09.244.2650	hypothetical protein, conserved	35.1 not assigned.no ontology
Tb09.244.2750	hypothetical protein, conserved	35.1 not assigned.no ontology
Tb10.26.0600	hypothetical protein, conserved	35.1 not assigned.no ontology
Tb10.26.0850	hypothetical protein, conserved	35.1 not assigned.no ontology
Tb10.389.0150	hypothetical protein, conserved	35.1 not assigned.no ontology
Tb10.389.1560	hypothetical protein, conserved	35.1 not assigned.no ontology
Tb10.61.0450	hypothetical protein, conserved	35.1 not assigned.no ontology
Tb10.61.0540	hypothetical protein, conserved	35.1 not assigned.no ontology
Tb10.61.0680	hypothetical protein, conserved	35.1 not assigned.no ontology
Tb10.61.0780	hypothetical protein, conserved	35.1 not assigned.no ontology
Tb10.61.0930	hypothetical protein, conserved	35.1 not assigned.no ontology
Tb10.61.1110	hypothetical protein, conserved	35.1 not assigned.no ontology
Tb10.61.1190	hypothetical protein, conserved	35.1 not assigned.no ontology
Tb10.61.1370	hypothetical protein, conserved	35.1 not assigned.no ontology
Tb10.61.1970	hypothetical protein, conserved	35.1 not assigned.no ontology
Tb10.61.2290	hypothetical protein, conserved	35.1 not assigned.no ontology
Tb10.61.2450	hypothetical protein, conserved	35.1 not assigned.no ontology
Tb10.61.2580	hypothetical protein, conserved	35.1 not assigned.no ontology
Tb10.61.2720	hypothetical protein, conserved	35.1 not assigned.no ontology
Tb10.61.2890	hypothetical protein, conserved	35.1 not assigned.no ontology
Tb10.6k15.0160	hypothetical protein, conserved	35.1 not assigned.no ontology
Tb10.6k15.0255	hypothetical protein, conserved	35.1 not assigned.no ontology
Tb10.6k15.0290	hypothetical protein, conserved	35.1 not assigned.no ontology

Tb10.6k15.1210	hypothetical protein, conserved	35.1 not assigned.no ontology
Tb10.6k15.1390	hypothetical protein, conserved	35.1 not assigned.no ontology
Tb10.6k15.1500	hypothetical protein, conserved	35.1 not assigned.no ontology
Tb10.6k15.1530	hypothetical protein, conserved	35.1 not assigned.no ontology
Tb10.6k15.1850	hypothetical protein, conserved	35.1 not assigned.no ontology
Tb10.6k15.2510	hypothetical protein, conserved	35.1 not assigned.no ontology
Tb10.6k15.2670	hypothetical protein, conserved	35.1 not assigned.no ontology
Tb10.6k15.3040	hypothetical protein, conserved	35.1 not assigned.no ontology
Tb10.6k15.3700	hypothetical protein, conserved	35.1 not assigned.no ontology
Tb10.70.0090	hypothetical protein, conserved	35.1 not assigned.no ontology
Tb10.70.0360	hypothetical protein, conserved	35.1 not assigned.no ontology
Tb10.70.1000	hypothetical protein, conserved	35.1 not assigned.no ontology
Tb10.70.1420	hypothetical protein, conserved	35.1 not assigned.no ontology
Tb10.70.1620	hypothetical protein, conserved	35.1 not assigned.no ontology
Tb10.70.2080	hypothetical protein, conserved	35.1 not assigned.no ontology
Tb10.70.2200	hypothetical protein, conserved	35.1 not assigned.no ontology
Tb10.70.2390	hypothetical protein, conserved	35.1 not assigned.no ontology
Tb10.70.3090	hypothetical protein, conserved	35.1 not assigned.no ontology
Tb10.70.3540	hypothetical protein, conserved	35.1 not assigned.no ontology
Tb10.70.3570	hypothetical protein, conserved	35.1 not assigned.no ontology
Tb10.70.4080	hypothetical protein, conserved	35.1 not assigned.no ontology
Tb10.70.4130	hypothetical protein, conserved	35.1 not assigned.no ontology
Tb10.70.4430	hypothetical protein, conserved	35.1 not assigned.no ontology
Tb10.70.4540	hypothetical protein, conserved	35.1 not assigned.no ontology
Tb10.70.4940	hypothetical protein, conserved	35.1 not assigned.no ontology
Tb10.70.5020	hypothetical protein, conserved	35.1 not assigned.no ontology
Tb10.70.5080	hypothetical protein, conserved	35.1 not assigned.no ontology
Tb10.70.5170	hypothetical protein, conserved	35.1 not assigned.no ontology
Tb10.70.5420	hypothetical protein, conserved	35.1 not assigned.no ontology
Tb10.70.5500	hypothetical protein, conserved	35.1 not assigned.no ontology
Tb10.70.6920	hypothetical protein, conserved	35.1 not assigned.no ontology
Tb10.70.7180	hypothetical protein, conserved	35.1 not assigned.no ontology
Tb10.70.7300	hypothetical protein, conserved	35.1 not assigned.no ontology
Tb10.70.7360	hypothetical protein, conserved	35.1 not assigned.no ontology
Tb10.70.7490	hypothetical protein, conserved	35.1 not assigned.no ontology
Tb10.70.7530	hypothetical protein, conserved	35.1 not assigned.no ontology

Tb10.70.7650	hypothetical protein, conserved	35.1 not assigned.no ontology
Tb10.70.7920	hypothetical protein, conserved	35.1 not assigned.no ontology
Tb11.01.0270	hypothetical protein, conserved	35.1 not assigned.no ontology
Tb11.01.0620	hypothetical protein, conserved	35.1 not assigned.no ontology
Tb11.01.0780	hypothetical protein, conserved	35.1 not assigned.no ontology
Tb11.01.0920	hypothetical protein, conserved	35.1 not assigned.no ontology
Tb11.01.1420	hypothetical protein, conserved	35.1 not assigned.no ontology
Tb11.01.1540	hypothetical protein, conserved	35.1 not assigned.no ontology
Tb11.01.1625	hypothetical protein, conserved	35.1 not assigned.no ontology
Tb11.01.1640	hypothetical protein, conserved	35.1 not assigned.no ontology
Tb11.01.1670	hypothetical protein, conserved	35.1 not assigned.no ontology
Tb11.01.1720	hypothetical protein, conserved	35.1 not assigned.no ontology
Tb11.01.1910	hypothetical protein, conserved	35.1 not assigned.no ontology
Tb11.01.2200	hypothetical protein, conserved	35.1 not assigned.no ontology
Tb11.01.2520	hypothetical protein, conserved	35.1 not assigned.no ontology
Tb11.01.2800	hypothetical protein, conserved	35.1 not assigned.no ontology
Tb11.01.3090	hypothetical protein, conserved	35.1 not assigned.no ontology
Tb11.01.3290	hypothetical protein, conserved	35.1 not assigned.no ontology
Tb11.01.3400	hypothetical protein, conserved	35.1 not assigned.no ontology
Tb11.01.3620	hypothetical protein, conserved	35.1 not assigned.no ontology
Tb11.01.3980	hypothetical protein, conserved	35.1 not assigned.no ontology
Tb11.01.4780	hypothetical protein, conserved	35.1 not assigned.no ontology
Tb11.01.4880	hypothetical protein, conserved	35.1 not assigned.no ontology
Tb11.01.5020	hypothetical protein, conserved	35.1 not assigned.no ontology
Tb11.01.5500	hypothetical protein, conserved	35.1 not assigned.no ontology
Tb11.01.5780	hypothetical protein, conserved	35.1 not assigned.no ontology
Tb11.01.6070	hypothetical protein, conserved	35.1 not assigned.no ontology
Tb11.01.6340	hypothetical protein, conserved	35.1 not assigned.no ontology
Tb11.01.6390	hypothetical protein, conserved	35.1 not assigned.no ontology
Tb11.01.6840	hypothetical protein, conserved	35.1 not assigned.no ontology
Tb11.01.7490	hypothetical protein, conserved	35.1 not assigned.no ontology
Tb11.01.7530	hypothetical protein, conserved	35.1 not assigned.no ontology
Tb11.01.7770	hypothetical protein, conserved	35.1 not assigned.no ontology
Tb11.01.8390	hypothetical protein, conserved	35.1 not assigned.no ontology
Tb11.01.8590	hypothetical protein, conserved	35.1 not assigned.no ontology
Tb11.01.8630	hypothetical protein, conserved	35.1 not assigned.no ontology

Tb11.01.8720	hypothetical protein, conserved	35.1 not assigned.no ontology
Tb11.01.8770	hypothetical protein, conserved	35.1 not assigned.no ontology
Tb11.02.0170	hypothetical protein, conserved	35.1 not assigned.no ontology
Tb11.02.0210	hypothetical protein, conserved	35.1 not assigned.no ontology
Tb11.02.0300	hypothetical protein, conserved	35.1 not assigned.no ontology
Tb11.02.0358	hypothetical protein, conserved	35.1 not assigned.no ontology
Tb11.02.0460	hypothetical protein, conserved	35.1 not assigned.no ontology
Tb11.02.0520	hypothetical protein, conserved	35.1 not assigned.no ontology
Tb11.02.0610	hypothetical protein, conserved	35.1 not assigned.no ontology
Tb11.02.0810	hypothetical protein, conserved	35.1 not assigned.no ontology
Tb11.02.0980	hypothetical protein, conserved	35.1 not assigned.no ontology
Tb11.02.0990	hypothetical protein, conserved	35.1 not assigned.no ontology
Tb11.02.1260	hypothetical protein, conserved	35.1 not assigned.no ontology
Tb11.02.1350	hypothetical protein, conserved	35.1 not assigned.no ontology
Tb11.02.1500	hypothetical protein, conserved	35.1 not assigned.no ontology
Tb11.02.1540	hypothetical protein, conserved	35.1 not assigned.no ontology
Tb11.02.1670	hypothetical protein, conserved	35.1 not assigned.no ontology
Tb11.02.1810	hypothetical protein, conserved	35.1 not assigned.no ontology
Tb11.02.1950	hypothetical protein, conserved	35.1 not assigned.no ontology
Tb11.02.2030	hypothetical protein, conserved	35.1 not assigned.no ontology
Tb11.02.2040	hypothetical protein, conserved	35.1 not assigned.no ontology
Tb11.02.2100	hypothetical protein, conserved	35.1 not assigned.no ontology
Tb11.02.2230	hypothetical protein, conserved	35.1 not assigned.no ontology
Tb11.02.2350	hypothetical protein, conserved	35.1 not assigned.no ontology
Tb11.02.2560	hypothetical protein, conserved	35.1 not assigned.no ontology
Tb11.02.2780	hypothetical protein, conserved	35.1 not assigned.no ontology
Tb11.02.2840	hypothetical protein, conserved	35.1 not assigned.no ontology
Tb11.02.3180	hypothetical protein, conserved	35.1 not assigned.no ontology
Tb11.02.3330	hypothetical protein, conserved	35.1 not assigned.no ontology
Tb11.02.3520	hypothetical protein, conserved	35.1 not assigned.no ontology
Tb11.02.3650	hypothetical protein, conserved	35.1 not assigned.no ontology
Tb11.02.4120	hypothetical protein, conserved	35.1 not assigned.no ontology
Tb11.02.4760	hypothetical protein, conserved	35.1 not assigned.no ontology
Tb11.02.5080	hypothetical protein, conserved	35.1 not assigned.no ontology
Tb11.02.5660	hypothetical protein, conserved	35.1 not assigned.no ontology
Tb11.03.0530	hypothetical protein, conserved	35.1 not assigned.no ontology

Tb11.03.0965	hypothetical protein, conserved	35.1 not assigned.no ontology
Tb11.18.0015	hypothetical protein, conserved	35.1 not assigned.no ontology
Tb11.22.0007	hypothetical protein, conserved	35.1 not assigned.no ontology
Tb11.39.0003	hypothetical protein, conserved	35.1 not assigned.no ontology
Tb11.39.0004	hypothetical protein, conserved	35.1 not assigned.no ontology
Tb11.39.0007	hypothetical protein, conserved	35.1 not assigned.no ontology
Tb11.39.0010	hypothetical protein, conserved	35.1 not assigned.no ontology
Tb11.46.0009	hypothetical protein, conserved	35.1 not assigned.no ontology
Tb11.46.0013	hypothetical protein, conserved	35.1 not assigned.no ontology
Tb11.47.0014	hypothetical protein, conserved	35.1 not assigned.no ontology
Tb11.52.0002	hypothetical protein, conserved	35.1 not assigned.no ontology
Tb11.52.0006	hypothetical protein, conserved	35.1 not assigned.no ontology
Tb11.52.0009	hypothetical protein, conserved	35.1 not assigned.no ontology
Tb11.52.0013	hypothetical protein, conserved	35.1 not assigned.no ontology
Tb11.55.0017	hypothetical protein, conserved	35.1 not assigned.no ontology
Tb11.55.0023	hypothetical protein, conserved	35.1 not assigned.no ontology
Tb11.55.0024	hypothetical protein, conserved	35.1 not assigned.no ontology
Tb927.1.1670	hypothetical protein, conserved	35.1 not assigned.no ontology
Tb927.1.2190	hypothetical protein, conserved	35.1 not assigned.no ontology
Tb927.1.3310	hypothetical protein, conserved	35.1 not assigned.no ontology
Tb927.1.4760	hypothetical protein, conserved	35.1 not assigned.no ontology
Tb927.1.740	hypothetical protein, conserved	35.1 not assigned.no ontology
Tb927.1.990	hypothetical protein, conserved	35.1 not assigned.no ontology
Tb927.2.2360	hypothetical protein, conserved	35.1 not assigned.no ontology
Tb927.2.2370	hypothetical protein, conserved	35.1 not assigned.no ontology
Tb927.2.2530	hypothetical protein, conserved	35.1 not assigned.no ontology
Tb927.2.41400	hypothetical protein, conserved	35.1 not assigned.no ontology
Tb927.2.4620	hypothetical protein, conserved	35.1 not assigned.no ontology
Tb927.2.4810/	hypothetical protein, conserved	35.1 not assigned.no ontology
Tb927.2.4850	hypothetical protein, conserved	35.1 not assigned.no ontology
Tb927.2.5530	hypothetical protein, conserved	35.1 not assigned.no ontology
Tb927.2.5540	hypothetical protein, conserved	35.1 not assigned.no ontology
Tb927.2.5810	hypothetical protein, conserved	35.1 not assigned.no ontology
Tb10.70.5840	hypothetical protein, conserved	35.1 not assigned.no ontology
Tb11.02.2520	hypothetical protein, conserved; major vault protein, putative	35.1 not assigned.no ontology
Tb10.70.1860	hypothetical protein, conserved; predicted ankyrin repeat family protein	35.1 not assigned.no ontology

Tb09.160.5670	hypothetical protein, conserved; predicted nucleotide-binding protein	35.1 not assigned.no ontology
Tb10.70.4040	hypothetical protein, conserved; predicted tetratricopeptide repeat protein	35.1 not assigned.no ontology
Tb10.70.1660	hypothetical protein, conserved; predicted TLD domain protein	35.1 not assigned.no ontology
Tb10.26.0330	hypothetical protein, conserved; predicted WD40 protein	35.1 not assigned.no ontology
Tb11.02.4620	hypothetical protein, conserved; predicted WD40 repeat protein	35.1 not assigned.no ontology
Tb11.02.5550	hypothetical protein, conserved; predicted WD40 repeat protein	35.1 not assigned.no ontology
Tb11.03.0880	hypothetical protein, conserved; predicted WD40 repeat protein	35.1 not assigned.no ontology
Tb10.70.6450	hypothetical protein, conserved; predicted WD40 repeat protein	35.1 not assigned.no ontology
Tb10.6k15.3020	hypothetical protein, conserved; regulator of transcription factor TFIID, putative	35.1 not assigned.no ontology
Tb927.2.5240	hypothetical protein, conserved; transportin2- like protein	35.1 not assigned.no ontology
Tb03.27F10.200	hypothetical protein, conserved; WD-repeat protein	35.1 not assigned.no ontology
Tb03.1J15.260	hypothetical protein, interrupted	35.1 not assigned.no ontology
Tb03.2H15.510	hypothetical protein, unlikely	35.1 not assigned.no ontology
Tb03.2H15.610	hypothetical protein, unlikely	35.1 not assigned.no ontology
Tb04.3I12.80	hypothetical protein, unlikely	35.1 not assigned.no ontology
Tb05.26K5.70	hypothetical protein, unlikely	35.1 not assigned.no ontology
Tb09.160.4390	hypothetical protein, unlikely	35.1 not assigned.no ontology
Tb09.160.4940	hypothetical protein, unlikely	35.1 not assigned.no ontology
Tb09.160.5330	hypothetical protein, unlikely	35.1 not assigned.no ontology
Tb09.211.1110	hypothetical protein, unlikely	35.1 not assigned.no ontology
Tb09.211.1860	hypothetical protein, unlikely	35.1 not assigned.no ontology
Tb09.244.2030	hypothetical protein, unlikely	35.1 not assigned.no ontology
Tb927.1.2000	hypothetical protein, unlikely	35.1 not assigned.no ontology
Tb927.1.3580	hypothetical protein, unlikely	35.1 not assigned.no ontology
Tb927.1.3590	hypothetical protein, unlikely	35.1 not assigned.no ontology
Tb927.1.4940	hypothetical protein, unlikely	35.1 not assigned.no ontology
Tb927.2.2770	hypothetical protein, unlikely	35.1 not assigned.no ontology
Tb927.2.5560	hypothetical protein, unlikely	35.1 not assigned.no ontology
Tb10.05.0170	hypothetical protein, unlikely	35.1 not assigned.no ontology
Tb11.53.0001	hypothetical protein; hypothetical protein	35.1 not assigned.no ontology
Tb11.26.0002	hypothetical protein; leucine-rich repeat protein (LRRP), putative	35.1 not assigned.no ontology
Tb09.160.0890	hypothetical protein; predicted WD40 protein	35.1 not assigned.no ontology
Tb10.6k15.2610	hypothetival protein, conserved	35.1 not assigned.no ontology
Tb09.160.4460	methyltransferase, putative	35.1 not assigned.no ontology
Tb09.160.4480	NLI-interacting factor, putative	35.1 not assigned.no ontology
Tb04.2L9.350	NLI-interacting factor, putative	35.1 not assigned.no ontology

Tb06.26F19.70	peroxisome biogenesis factor 1, putative	35.1 not assigned.no ontology
Tb05.29K2.700	retrotransposon hot spot (RHS) protein	35.1 not assigned.no ontology
Tb03.48O8.330	SPFH domain / Band 7 family protein, putative	35.1 not assigned.no ontology
Tb03.6N20.530	Tb03.48O8.330 expression site-associated gene (ESAG) protein, putative;	35.1 not assigned.no ontology
Tb07.13M20.30	Tb03.6N20.530 expression site-associated gene (ESAG) protein, putative;	35.1 not assigned.no ontology
Tb10.08.0010	Tb07.13M20.30 expression site-associated gene (ESAG) protein, putative;	35.1 not assigned.no ontology
Tb10.1130	Tb10.08.0010 hypothetical protein 851393:851968 forward MW:21798	35.1 not assigned.no ontology
Tb10.1570	Tb10.1130 hypothetical protein 317995:318726 reverse MW:27176	35.1 not assigned.no ontology
Tb10.1640	Tb10.1570 hypothetical protein 4620:5207 forward MW:22254	35.1 not assigned.no ontology
Tb10.1660	Tb10.1640 hypothetical protein 21158:22549 forward MW:50631	35.1 not assigned.no ontology
Tb11.1240	Tb10.1660 hypothetical protein 29871:30956 forward MW:39633	35.1 not assigned.no ontology
Tb11.1370	Tb11.1240 hypothetical protein 545932:547119 forward MW:43784	35.1 not assigned.no ontology
Tb11.1380	Tb11.1370 hypothetical protein 606527:607849 forward MW:50849	35.1 not assigned.no ontology
Tb11.1400	Tb11.1380 hypothetical protein 608473:609672 forward MW:45634	35.1 not assigned.no ontology
Tb11.1410	Tb11.1400 hypothetical protein 615077:616963 forward MW:68583	35.1 not assigned.no ontology
Tb11.1420	Tb11.1410 hypothetical protein 617534:618490 forward MW:33748	35.1 not assigned.no ontology
Tb11.55.0027	Tb11.1420 hypothetical protein 622141:623463 forward MW:50918	35.1 not assigned.no ontology
Tb927.1.120	Tb11.55.0027 expression site-associated gene (ESAG) protein, putative;	35.1 not assigned.no ontology
Tb927.2.100	Tb927.1.120 retrotransposon hot spot (RHS) protein, putative;	35.1 not assigned.no ontology
Tb927.2.1080	Tb927.2.100/Tb08.30P3.280 retrotransposon hot spot (RHS) protein, putative;	35.1 not assigned.no ontology
Tb927.2.340	Tb927.2.1080/25N24.150 retrotransposon hot spot (RHS) protein, putative;	35.1 not assigned.no ontology
Tb927.2.470	Tb927.2.340/3B10.125 retrotransposon hot spot (RHS) protein, putative;	35.1 not assigned.no ontology
Tb927.2.690	Tb927.2.470/3B10.190 retrotransposon hot spot (RHS) protein, putative;	35.1 not assigned.no ontology
Tb06.4F7.320	Tb927.2.690/3B10.300 leucine-rich repeat protein (LRRP), putative;	35.1 not assigned.no ontology
Tb07.28B13.620	TPR domain protein, conserved	35.1 not assigned.no ontology
Tb07.28B13.630	I/6 autoantigen, putative	35.2 not assigned.unknown
Tb08.30K1.660	I/6 autoantigen, putative	35.2 not assigned.unknown
Tb08.11J15.120	IgE-dependent histamine-releasing factor, putative	35.2 not assigned.unknown
Tb05.26C7.80	short chain dehydrogenase/reductase, putative	35.2 not assigned.unknown
Tb05.26K5.210	small GTP-binding protein, putative	35.2 not assigned.unknown
Tb927.5.2940	stress-induced protein sti1, putative	35.2 not assigned.unknown
