# Supplementary material for: Exocytosis and protein secretion in Trypanosoma
Source: BMC Microbiol. 2010 Jan 26;10:20. doi: 10.1186/1471-2180-10-20 (PMC3224696; doi:10.1186/1471-2180-10-20)
Supplement: Additional file 6 — Table S6. Genome-wide prediction of secreted proteins using SignalP and secretomeP. contains the list of 1445 SignalP-predicted proteins (containing a putative transit peptide) from T. brucei and classified according to the number of predicted transmembrane spans (TMHMM prediction) (sheet 1). SecretomeP-predicted proteins from T. brucei were reported according to their p-value (sheet 2). The 3 highest classes p>0.9, 0.9>p>0.8, and 0.8>p>0.7 containing, respectively, 128, 583, and 875 proteins and their number of predicted transmembrane spans (TMHMM prediction) were reported. [file 1471-2180-10-20-S6.PDF]

**Table S6**
**SHEET 1 (SignalP Prediction)**

**SignalP Prediction on Trypanosome proteome**

| Accession number | Protein name | TMHMM prediction |
|---|---|---|
| Tb927.2.3270 | 65 kDa invariant surface glycoprotein | 1 |
| Tb927.2.3280 | 65 kDa invariant surface glycoprotein | 1 |
| Tb927.2.3290 | 65 kDa invariant surface glycoprotein | 1 |
| Tb927.2.3300 | 65 kDa invariant surface glycoprotein | 1 |
| Tb927.2.3310 | 65 kDa invariant surface glycoprotein | 1 |
| Tb927.2.3320 | 65 kDa invariant surface glycoprotein | 1 |
| Tb11.47.0001 | 65 kDa invariant surface glycoprotein-like protein | 1 |
| Tb927.5.390 | 75 kDa invariant surface glycoprotein, putative | 2 |
| Tb927.5.1430 | ISG64 64 kDa invariant surface glycoprotei | 1 |
| Tb927.5.1390 | ISG64 64 kDa invariant surface glycoprotein | 1 |
| Tb927.5.1410 | ISG64 64 kDa invariant surface glycoprotein | 1 |
| Tb927.5.360 | ISG75 75 kDa invariant surface glycoprotein | 1 |
| Tb927.5.350 | ISG75 75 kDa invariant surface glycoprotein, putative | 1 |
| Tb927.5.370 | ISG75 75 kDa invariant surface glycoprotein, putative | 1 |
| Tb927.5.380 | ISG75 75 kDa invariant surface glycoprotein, putative | 1 |
| Tb927.5.400 | ISG75 75 kDa invariant surface glycoprotein, putative | 1 |
| Tb927.7.6850 | TbTS trans-sialidase | 0 |
| Tb11.01.3240 | trans-sialidase, putative | 0 |
| Tb927.2.5280 | trans-sialidase, putative | 0 |
| Tb927.7.6830 | trans-sialidase, putative | 0 |
| Tb927.5.640 | trans-sialidase, putative,neuraminidase, putative | 2 |
| Tb927.8.7340 | trans-sialidase, putative,neuraminidase, putative | 0 |
| Tb927.8.7350 | trans-sialidase, putative,neuraminidase, putative | 0 |
| Tb927.2.6130 | ABC transporter, putative | 1 |
| Tb927.3.3730 | ABC transporter, putative | 11 |
| Tb927.8.2380 | ABC transporter, putative | 9 |
| Tb927.2.5410 | ABC transporter, putative,multidrug resistance protein | 1 |

| | | |
|---|---|---|
| Tb11.03.0540 | ABCB10  ABC transporter, putative | 6 |
| Tb10.6k15.3720 | acid phosphatase, putative | 1 |
| Tb927.5.610 | acidic phosphatase, putative | 2 |
| Tb927.5.630 | acidic phosphatase, putative | 1 |
| Tb927.3.2840 | inorganic pyrophosphatase, putative | 0 |
| Tb11.01.4701 | MBAP1   membrane-bound acid phosphatase 1 precursor | 2 |
| Tb10.6k15.3560 | MBAP2   membrane-bound acid phosphatase 2 | 1 |
| Tb11.01.3610 | membrane-bound acid phosphatase, putative | 2 |
| Tb10.406.0290 | protein tyrosine phosphatase, putative | 7 |
| Tb927.5.3150 | protein kinase , putative | 3 |
| Tb927.7.940 | protein kinase C substrate protein, heavy chain, putative,glucosidase II beta subunit, putative | 0 |
| Tb10.61.0100 | protein kinase, putative | 1 |
| Tb927.5.3160 | protein kinase, putative | 0 |
| Tb927.4.2500 | protein kinase, putative,eukaryotic translation initiation factor 2-alpha kinase precursor, putative | 2 |
| Tb927.4.3680 | protein phosphatase 2C, putative | 0 |
| Tb927.6.4630 | serine/threonine protein phosphatase, putative | 1 |
| Tb927.6.4630 | serine/threonine protein phosphatase, putative | 1 |
| Tb927.6.750 | serine/threonine protein phosphatase, putative | 0 |
| Tb10.70.2260 | PK50    serine/threonine protein kinase | 0 |
| Tb927.3.590 | adenosine transporter, putative | 11 |
| Tb11.02.1100 | NT8.1   nucleobase/nucleoside transporter 8.1,nucleobase transporter | 9 |
| Tb11.02.1105 | NT8.1   nucleobase/nucleoside transporter 8.1,nucleobase transporter | 9 |
| Tb11.02.1106 | nucleobase transporter, putative | 9 |
| Tb09.244.2020 | nucleoside transporter 1, putative | 11 |
| Tb09.160.5480 | TbNT10  purine nucleoside transporter | 10 |
| Tb927.2.6150 | TbNT2/927       adenosine transporter 2 | 11 |
| Tb927.2.6200 | TbNT3   adenosine transporter 2, putative | 11 |
| Tb927.2.6220 | TbNT4   adenosine transporter 2, putative | 10 |
| Tb927.2.6240 | TbNT5   adenosine transporter 2 | 11 |
| Tb927.2.6320 | TbNT6   adenosine transporter 2, putative | 11 |
| Tb927.2.6280 | TbNT7   adenosine transporter 2, putative | 11 |
| Tb927.6.220 | TbNT9   purine nucleoside transporter | 10 |
| Tb11.02.3020 | sugar transporter, putative | 12 |
| Tb927.8.7680 | amino acid transporter, putative | 10 |

| | | |
|---|---|---|
| Tb927.8.7700 | amino acid transporter, putative | 10 |
| Tb927.5.4500 | ADP-ribosylation factor, putative | 0 |
| Tb10.61.3010 | UDP-galactose transporter, putative | 7 |
| Tb927.1.580 | phosphate-repressible phosphate permease, putative | 10 |
| Tb927.1.600 | phosphate-repressible phosphate permease, putative | 10 |
| Tb10.389.1330 | membrane transporter protein, putative | 12 |
| Tb09.211.3870 | transporter, putative | 11 |
| Tb10.61.0890 | transporter, putative | 8 |
| Tb10.61.2750 | transporter, putative,major facilitator superfamily protein (MFS), putative | 4 |
| Tb11.02.4100 | pretranslocation protein, alpha subunit, putative,SEC61-like (pretranslocation process) protein, putative | 9 |
| Tb11.02.4040 | protein transport protein Sec31, putative,cytosolic coat protein, putative | 0 |
| Tb11.55.0012 | vesicular-fusion protein SEC18, putative | 1 |
| Tb11.55.0014 | vesicular transport protein (CDC48 homologue), putative | 1 |
| Tb927.4.4460 | receptor-type adenylate cyclase GRESAG 4, putative,receptor-type adenylate cyclase GRESAG 4.4B | 1 |
| Tb927.6.470 | gene related to expression site-associated gene 2 (GRESAG2), putative | 0 |
| Tb927.6.470 | gene related to expression site-associated gene 2 (GRESAG2), putative | 0 |
| Tb927.6.500 | gene related to expression site-associated gene 2 (GRESAG2), putative | 0 |
| Tb927.6.540 | gene related to expression site-associated gene 2 (GRESAG2), putative | 0 |
| Tb11.01.5310 | receptor-type adenylate cyclase GRESAG 4, putative | 2 |
| Tb11.27.0001 | receptor-type adenylate cyclase GRESAG 4, putative | 1 |
| Tb927.4.3750 | receptor-type adenylate cyclase GRESAG 4, putative | 2 |
| Tb927.4.4410 | receptor-type adenylate cyclase GRESAG 4, putative | 1 |
| Tb927.4.4430 | receptor-type adenylate cyclase GRESAG 4, putative | 1 |
| Tb927.4.4440 | receptor-type adenylate cyclase GRESAG 4, putative | 1 |
| Tb927.4.4450 | receptor-type adenylate cyclase GRESAG 4, putative | 1 |
| Tb927.4.4470 | receptor-type adenylate cyclase GRESAG 4, putative | 1 |
| Tb927.5.650 | receptor-type adenylate cyclase GRESAG 4, putative | 1 |
| Tb927.6.190 | receptor-type adenylate cyclase GRESAG 4, putative | 1 |
| Tb927.7.6040 | receptor-type adenylate cyclase GRESAG 4, putative | 1 |
| Tb927.7.6050 | receptor-type adenylate cyclase GRESAG 4, putative | 1 |
| Tb927.7.6060 | receptor-type adenylate cyclase GRESAG 4, putative | 2 |
| Tb927.7.6070 | receptor-type adenylate cyclase GRESAG 4, putative | 1 |
| Tb927.7.6080 | receptor-type adenylate cyclase GRESAG 4, putative | 1 |
| Tb927.7.7470 | receptor-type adenylate cyclase GRESAG 4, putative | 1 |

| | | |
|---|---|---|
| Tb927.7.7520 | receptor-type adenylate cyclase GRESAG 4, putative | 2 |
| Tb927.7.7530 | receptor-type adenylate cyclase GRESAG 4, putative | 2 |
| Tb927.8.7590 | receptor-type adenylate cyclase GRESAG 4, putative | 1 |
| Tb927.8.7860 | receptor-type adenylate cyclase GRESAG 4, putative | 5 |
| Tb927.8.7870 | receptor-type adenylate cyclase GRESAG 4, putative | 1 |
| Tb927.8.7890 | receptor-type adenylate cyclase GRESAG 4, putative | 2 |
| Tb927.8.7900 | receptor-type adenylate cyclase GRESAG 4, putative | 2 |
| Tb927.8.7920 | receptor-type adenylate cyclase GRESAG 4, putative | 2 |
| Tb927.8.7930 | receptor-type adenylate cyclase GRESAG 4, putative | 2 |
| Tb927.8.7940 | receptor-type adenylate cyclase GRESAG 4, putative | 2 |
| Tb927.5.320 | receptor-type adenylate cyclase GRESAG 4, putative,adenylyl cyclase, putative | 1 |
| Tb927.5.330 | receptor-type adenylate cyclase GRESAG 4, putative,adenylyl cyclase, putative,ATP pyrophosphate-lyase, putative | 1 |
| Tb927.5.4540 | receptor-type adenylate cyclase GRESAG 4, putative,adenylyl cyclase, putative,ATP pyrophosphate-lyase, putative | 1 |
| Tb927.5.4550 | receptor-type adenylate cyclase GRESAG 4, putative,adenylyl cyclase, putative,ATP pyrophosphate-lyase, putative | 1 |
| Tb10.389.0430 | receptor-type adenylate cyclase GRESAG 4, putative,ESAG 4 (expression site-associated gene 4) protein, putative | 1 |
| Tb927.7.5160 | deoxyuridine triphosphatase, putative,dUTP diphosphatase | 0 |
| Tb09.160.2000 | pseudouridylate synthase, putative | 0 |
| Tb927.7.1640 | GTP-binding protein, putative | 0 |
| Tb10.70.4600 | GTP-binding protein, putative,elongation factor, putative | 0 |
| Tb927.7.1930 | nucleoside diphosphatase, putative,ectonucleoside triphosphate diphosphohydrolase 5, putative | 1 |
| Tb927.8.3800 | nucleoside phosphatase, putative,guanosine diphosphatase, putative | 1 |
| Tb10.389.1430 | helicase-like protein | 0 |
| Tb11.01.1100 | helicase-like protein | 0 |
| Tb10.61.2130 | ATP-dependent DEAD/H RNA helicase, putative | 0 |
| Tb927.6.1640 | single strand-specific nuclease, putative | 0 |
| Tb927.6.1650 | single strand-specific nuclease, putative | 0 |
| Tb927.6.2890 | single strand-specific nuclease, putative | 1 |
| Tb927.8.2810 | XRNC 5'-3' exonuclease XRNC, putative,exoribonuclease 2, putative | 0 |
| Tb10.70.0610 | XRND 5'-3' exoribonuclease XRND, putative,5'-3' exoribonuclease 2, putative | 0 |
| Tb10.70.1510 | endonuclease/exonuclease/phosphatase, putative | 0 |
| Tb10.70.0780 | tRNA pseudouridine synthase A, putative | 0 |
| Tb10.61.0690 | tRNA pseudouridine synthase A-like protein | 0 |
| Tb09.244.2400 | BARP BARP protein | 0 |
| Tb09.244.2410 | BARP BARP protein | 0 |

| | | |
|---|---|---|
| Tb09.244.2420 | BARP BARP protein | 0 |
| Tb09.244.2430 | BARP BARP protein | 0 |
| Tb09.244.2440 | BARP BARP protein | 0 |
| Tb09.244.2450 | BARP BARP protein | 0 |
| Tb09.244.2460 | BARP BARP protein | 0 |
| Tb09.244.2470 | BARP BARP protein | 0 |
| Tb09.244.2480 | BARP BARP protein | 0 |
| Tb09.244.2490 | BARP BARP protein | 0 |
| Tb09.244.2500 | BARP BARP protein | 0 |
| Tb09.244.2510 | BARP BARP protein | 0 |
| Tb09.244.2520 | BARP BARP protein | 0 |
| Tb09.244.2530 | BARP BARP protein | 0 |
| Tb09.211.3680 | chaperone protein DNAJ, putative | 0 |
| Tb11.01.8480 | chaperone protein DNAJ, putative | 0 |
| Tb11.02.2880 | chaperone protein DNAJ, putative | 1 |
| Tb927.3.1430 | chaperone protein DnaJ, putative | 0 |
| Tb927.3.1760 | chaperone protein DNAJ, putative | 0 |
| Tb927.7.6200 | chaperone protein DNAJ, putative | 1 |
| Tb927.7.680 | chaperone protein DNAJ, putative | 0 |
| Tb927.8.7010 | chaperone protein DNAJ, putative | 0 |
| Tb927.7.3630 | TPR-repeat-containing chaperone protein DNAJ, putative | 0 |
| Tb927.6.2170 | co-chaperone GrpE, putative | 0 |
| Tb11.01.4050 | heat shock protein HslVU, ATPase subunit HslU, putative,ATP-dependent hsl protease ATP-binding subunit hslU, | 0 |
| Tb09.160.3090 | heat shock protein, putative,HSP70-like protein | 1 |
| Tb927.2.1560 | cyclophilin type peptidyl-prolyl cis-trans isomerase precursor, putative | 0 |
| Tb10.61.0760 | FKBP-type peptidyl-prolyl cis-trans isomerase, conserved,FKBP-type peptidylprolyl cis-trans isomerase, putative | 0 |
| Tb927.8.2000 | PPIase cyclophilin type peptidyl-prolyl cis-trans isomerase, putative,peptidyl-prolyl cis-trans isomerase, putative | 1 |
| Tb927.7.280 | PPIase cyclophilin-type peptidyl-prolyl cis-trans isomerase, putative | 0 |
| Tb927.5.1370 | PPIase cyclophilin-type peptidyl-prolyl cis-trans isomerase, putative, | 0 |
| Tb927.7.1300 | protein disulfide isomerase, putative | 0 |
| Tb927.7.5790 | protein disulfide isomerase, putative | 0 |
| Tb10.6k15.2290 | protein disulfide isomerase,bloodstream-specific protein 2 precursor | 0 |
| Tb927.5.1020 | disulfide isomerase, putative | 2 |
| Tb09.211.0680 | CAAX prenyl protease 1, putative,metallo-peptidase, Clan M-Family M48 | 5 |

| | | |
|---|---|---|
| Tb10.70.7090 | serine carboxypeptidase III precursor, putative,serine carboxypeptidase (CBP1) precursor, Clan SC, Family S10 | 0 |
| Tb10.70.7080 | CBP1 serine carboxypeptidase III precursor, putative,serine peptidase, Clan SC, Family S10 | 1 |
| Tb10.70.7100 | CBP1 serine carboxypeptidase III precursor, putative,serine peptidase, Clan SC, Family S10 | 0 |
| Tb10.389.0470 | calpain-like cysteine peptidase, putative,cysteine peptidase, Clan CA, family C2, putative | 0 |
| Tb11.01.5800 | calpain-like cysteine peptidase, putative,cysteine peptidase, Clan CA, family C2, putative | 0 |
| Tb11.02.5610 | Gp63-1 surface protease homolog, putative | 0 |
| Tb11.02.5630 | Gp63-1 surface protease homolog, putative | 0 |
| Tb11.02.5640 | Gp63-1 surface protease homolog, putative | 0 |
| Tb11.02.5310 | major surface protease A, putative,Gp63 major surface glycoprotein-like protein,MSP-A, putative | 0 |
| Tb927.8.1610 | major surface protease gp63, putative,GP63, putative,metallopeptidase, putative | 2 |
| Tb10.70.5290 | MSP-C major surface protease gp63, putative,surface protease homologue | 0 |
| Tb927.3.4910 | signal peptide peptidase, putative,aspartic peptidase, clan AD, family A22B, putative | 8 |
| Tb11.02.1280 | subtilisin-like serine peptidase,serine peptidase, clan SB, family S8-like protein | 1 |
| Tb927.3.4230 | subtilisin-like serine peptidase,serine peptidase, clan SB, family S8-like protein | 1 |
| Tb927.8.1810 | rhomboid-like protein,serine peptidase, Clan S-, family S54, putative | 6 |
| Tb927.6.560 | TbcatB cysteine peptidase C (CPC),CPC cysteine peptidase, Clan CA, family C1, Cathepsin B-like | 0 |
| Tb09.211.2310 | Bem46-like serine peptidase,Serine peptidase, Clan SC, Family S09X | 1 |
| Tb09.211.4760 | metacaspase 5, putative | 0 |
| Tb927.6.1000 | CP cysteine peptidase precursor,cysteine peptidase, Clan CA, family C1, Cathepsin L-like | 1 |
| Tb927.6.1010 | CP cysteine peptidase precursor,cysteine peptidase, Clan CA, family C1, Cathepsin L-like | 1 |
| Tb927.6.1020 | CP cysteine peptidase precursor,cysteine peptidase, Clan CA, family C1, Cathepsin L-like | 1 |
| Tb927.6.1030 | CP cysteine peptidase precursor,cysteine peptidase, Clan CA, family C1, Cathepsin L-like | 1 |
| Tb927.6.1040 | CP cysteine peptidase precursor,cysteine peptidase, Clan CA, family C1, Cathepsin L-like | 1 |
| Tb927.6.1050 | CP cysteine peptidase precursor,cysteine peptidase, Clan CA, family C1, Cathepsin L-like | 1 |
| Tb927.6.1060 | CP cysteine peptidase precursor,cysteine peptidase, Clan CA, family C1, Cathepsin L-like | 1 |
| Tb927.6.960 | CP cysteine peptidase precursor,cysteine peptidase, Clan CA, family C1, Cathepsin L-like | 1 |
| Tb927.6.970 | CP cysteine peptidase precursor,cysteine peptidase, Clan CA, family C1, Cathepsin L-like | 1 |
| Tb927.6.980 | CP cysteine peptidase precursor,cysteine peptidase, Clan CA, family C1, Cathepsin L-like | 1 |
| Tb927.6.990 | CP cysteine peptidase precursor,cysteine peptidase, Clan CA, family C1, Cathepsin L-like | 1 |
| Tb09.160.3110 | mitochondrial processing peptide beta subunit, putative,metallo-peptidase, Clan ME, Family M16 | 0 |
| Tb09.211.3330 | cystathione gamma lyase, putative | 1 |
| Tb927.7.3500 | glutathione-S-transferase/glutaredoxin,putative | 0 |
| Tb927.4.1350 | glx2-2; glx II hydroxyacylglutathione hydrolase, putative,glyoxalase II | 0 |
| Tb11.01.7480 | superoxide dismutase, putative | 0 |

| | | |
|---|---|---|
| Tb927.4.2450 | thioredoxin, putative | 1 |
| Tb927.4.4080 | C-5 sterol desaturase, putative | 4 |
| Tb927.3.4650 | C-8 sterol isomerase, putative | 1 |
| Tb11.18.0008 | diacylglycerol acyltransferase, putative | 25 |
| Tb10.6k15.3080 | dihydrolipoamide acetyltransferase precursor, putative | 0 |
| Tb10.70.5380 | dihydrolipoamide acetyltransferase, putative | 0 |
| Tb09.160.4300 | farnesyl pyrophosphate synthetase, putative | 0 |
| Tb927.1.1130 | glycerol-3-phosphate dehydrogenase (FAD-dependent), putative | 1 |
| Tb11.01.0580 | glycerophosphoryl diester phosphodiesterase, putative | 1 |
| Tb927.4.1020 | serine-palmitoyl-CoA transferase, putative | 1 |
| Tb09.160.0390 | monooxygenase, putative | 0 |
| Tb927.5.490 | methyltransferase, putative | 0 |
| Tb11.02.4080 | LDM; P450LI lanosterol 14-alpha-demethylase,cytochrome P450 51A1 | 0 |
| Tb927.3.3860 | lipase, putative,triacylglycerol lipase, putative | 6 |
| Tb10.61.1530 | lipoic acid synthetase, mitochondrial precursor, putative | 0 |
| Tb927.3.3580 | LPG3 lipophosphoglycan biosynthetic protein, putative,heat shock protein 90, putative,glucose regulated protein 94, | 1 |
| Tb927.8.480 | phosphatidic acid phosphatase protein, putative | 6 |
| Tb10.61.2970 | phosphatidic acid phosphatase, putative | 6 |
| Tb927.7.3760 | phosphatidyl serine synthase, putative | 8 |
| Tb09.211.1010 | phosphatidylcholine:ceramide cholinephosphotransferase 2, putative | 5 |
| Tb927.8.1720 | phosphatidylglycerophosphate synthase-like protein, putative | 1 |
| Tb927.4.1140 | phosphatidylinositol 4-kinase, putative | 0 |
| Tb927.4.1920 | glycosylphosphatidylinositol (GPI) anchor, putative | 2 |
| Tb11.01.2110 | phosphoglycerate mutase-like protein,phosphoglycerate mutase protein, putative | 0 |
| Tb09.211.3650 | phospholipase A2-like protein, putative | 2 |
| Tb11.02.0780 | squalene monooxygenase, putative | 2 |
| Tb11.01.6800 | 1-acyl-sn-glycerol-3-phosphate acyltransferase protein, putative | 1 |
| Tb927.3.3360 | acyltransferase, putative | 6 |
| Tb927.3.4820 | acyltransferase, putative | 3 |
| Tb927.5.2280 | acetyltransferase, putative | 4 |
| Tb10.100.0100 | GPI transamidase component GAA1 | 6 |
| Tb10.70.1440 | GPI10 GPI anchor biosynthesis protein | 8 |
| Tb11.01.3900 | GPI12 N-acetylglucosaminylphosphatidylinositoldeacetylase | 1 |
| Tb10.61.3060 | GPI-anchor transamidase subunit 8 (GPI8),cysteine peptidase, Clan CD, family C13 | 0 |

| | | |
|---|---|---|
| Tb10.70.2420 | GPIdeAc GPI inositol deacylase precursor | 0 |
| Tb927.6.4280 | GAPDH glyceraldehyde 3-phosphate dehydrogenase, glycosomal | 0 |
| Tb927.6.4300 | GAPDH glyceraldehyde 3-phosphate dehydrogenase, glycosomal | 0 |
| Tb11.01.5560 | GDP-L-fucose synthetase, putative | 0 |
| Tb11.02.5450 | glucose-regulated protein 78, putative,luminal binding protein 1 (BiP), putative | 0 |
| Tb11.02.5500 | glucose-regulated protein 78, putative,luminal binding protein 1 (BiP), putative | 0 |
| Tb10.05.0080 | glucosidase, putative | 0 |
| Tb927.8.2910 | mannosyl-oligosaccharide 1,2-alpha-mannosidase IB, putative | 0 |
| Tb927.8.2920 | mannosyl-oligosaccharide 1,2-alpha-mannosidase IB, putative | 0 |
| Tb927.8.2930 | mannosyl-oligosaccharide 1,2-alpha-mannosidase IB, putative | 0 |
| Tb927.8.2940 | mannosyl-oligosaccharide 1,2-alpha-mannosidase IB, putative | 0 |
| Tb927.6.3300 | TbPIG-M mannosyltransferase | 7 |
| Tb09.211.3640 | prenyl protein specific carboxyl methyltransferase | 4 |
| Tb10.389.0250 | glycosyltransferase, putative | 2 |
| Tb927.4.2230 | glycosyltransferase ALG2, putative | 1 |
| Tb927.6.1960 | glycosyltransferase family 28 protein, putative | 2 |
| Tb09.211.0860 | glycosyl transferase, putative | 0 |
| Tb09.142.0450 | UDP-Gal or UDP-GlcNAc-dependent glycosyltransferase, putative | 0 |
| Tb10.389.1450 | UDP-Gal or UDP-GlcNAc-dependent glycosyltransferase, putative | 1 |
| Tb927.2.3370 | UDP-Gal or UDP-GlcNAc-dependent glycosyltransferase, putative | 1 |
| Tb927.3.5660 | UDP-Gal or UDP-GlcNAc-dependent glycosyltransferase, putative | 1 |
| Tb927.4.4250 | UDP-Gal or UDP-GlcNAc-dependent glycosyltransferase, putative | 1 |
| Tb927.4.4270 | UDP-Gal or UDP-GlcNAc-dependent glycosyltransferase, putative | 1 |
| Tb927.4.4290 | UDP-Gal or UDP-GlcNAc-dependent glycosyltransferase, putative | 1 |
| Tb927.4.5240 | UDP-Gal or UDP-GlcNAc-dependent glycosyltransferase, putative | 1 |
| Tb927.4.5250 | UDP-Gal or UDP-GlcNAc-dependent glycosyltransferase, putative | 1 |
| Tb927.4.5260 | UDP-Gal or UDP-GlcNAc-dependent glycosyltransferase, putative | 1 |
| Tb927.4.5270 | UDP-Gal or UDP-GlcNAc-dependent glycosyltransferase, putative | 1 |
| Tb927.4.5280 | UDP-Gal or UDP-GlcNAc-dependent glycosyltransferase, putative | 1 |
| Tb927.4.5290 | UDP-Gal or UDP-GlcNAc-dependent glycosyltransferase, putative | 1 |
| Tb927.5.2760 | UDP-Gal or UDP-GlcNAc-dependent glycosyltransferase, putative | 1 |
| Tb927.7.300 | UDP-Gal or UDP-GlcNAc-dependent glycosyltransferase, putative | 0 |
| Tb927.8.7140 | UDP-Gal or UDP-GlcNAc-dependent glycosyltransferase, putative | 1 |
| Tb927.8.7150 | UDP-Gal or UDP-GlcNAc-dependent glycosyltransferase, putative | 1 |

| | | |
|---|---|---|
| Tb927.3.4630 | UDP-glucose:glycoprotein glucosyltransferase, putative | 1 |
| Tb927.2.4720 | ALG12 dolichyl-P-Man:Man7GlcNAc2-PP-dolichylalpha6-ma nn osyltransferase,putative | 7 |
| Tb927.6.1140 | Alg9-like mannosyltransferase, putative | 10 |
| Tb10.70.0260 | alpha-1,3-mannosyltransferase, putative | 8 |
| Tb11.01.7190 | B5R NADH-cytochrome b5 reductase, putative | 0 |
| Tb11.02.1230 | B5R NADH-cytochrome b5 reductase, putative | 1 |
| Tb927.5.1470 | B5R NADH-cytochrome b5 reductase, putative | 0 |
| Tb09.211.4110 | CPR NADPH--cytochrome p450 reductase, putative | 1 |
| Tb11.01.0170 | CPR NADPH--cytochrome P450 reductase, putative | 1 |
| Tb11.02.5420 | CPR NADPH--cytochrome p450 reductase, putative | 1 |
| Tb10.6k15.3510 | CRAM cysteine-rich, acidic integral membrane protein precursor | 1 |
| Tb11.55.0013 | cysteine desulfurase, putative | 0 |
| Tb09.244.2760 | cytosolic coat protein, putative | 2 |
| Tb11.01.6880 | cytosolic coat protein, putative | 2 |
| Tb11.01.0530 | DNA repair protein, putative | 0 |
| Tb11.02.5530 | electron-transfer-flavoprotein, alpha polypeptide, putative | 0 |
| Tb927.8.4890 | endoplasmic reticulum oxidoreductin, putative,pol-associated gene 1 | 1 |
| Tb11.02.0960 | endosomal integral membrane protein, putative | 9 |
| Tb927.3.4190 | endosomal integral membrane protein, putative | 12 |
| Tb927.4.1090 | endosomal integral membrane protein, putative | 9 |
| Tb927.8.1940 | endosomal integral membrane protein, putative | 9 |
| Tb10.6k15.0020 | EP1 EP1 procyclin | 1 |
| Tb10.6k15.0030 | EP2 EP2 procyclin | 1 |
| Tb927.6.480 | EP3-2 procyclin,PARP A-beta,surface protein EP3-2,surface protein EP3-2 procyclin precursor, | 1 |
| Tb927.6.480 | EP3-2 procyclin,PARP A-beta,surface protein EP3-2,surface protein EP3-2 procyclin precursor, | 1 |
| Tb927.6.520 | EP3-2 procyclin,PARP A-beta,surface protein EP3-3 procyclin precursor, | 1 |
| Tb927.6.450 | EP3-2 procyclin,PARP,procyclin PARP A,procyclin B1-alpha,procyclic acidic repetitive protein A.beta, | 1 |
| Tb927.6.510 | GPEET2 procyclin precursor,PARP A-alpha,procyclin A-alpha,procyclic form specific polypeptide A-alpha precursor | 1 |
| Tb10.6k15.0040 | PAG1 procyclin-associated gene 1 (PAG1) protein | 0 |
| Tb10.6k15.0060 | PAG2 procyclin-associated gene 2 (PAG2) protein | 0 |
| Tb10.6k15.0070 | PAG4 procyclin-associated gene 4 (PAG4) protein | 0 |
| Tb10.6k15.0050 | PAG5 procyclin-associated gene 5 (PAG5) protein | 0 |
| Tb10.70.1310 | procyclin-associated gene 1 (PAG1) protein, putative | 0 |
| Tb10.70.1300 | procyclin-associated gene 2 (PAG2) protein, putative | 0 |

| | | |
|---|---|---|
| Tb11.01.6210 | procyclin-associated gene 2-like protein, putative | 1 |
| Tb11.01.6220 | procyclin-associated gene 4 (PAG4) protein, putative | 0 |
| Tb10.v4.0002 | expression site-associated gene (ESAG) protein, putative,expression site-associated gene 1 (ESAG1) protein, putative | 0 |
| Tb11.17.0002 | expression site-associated gene (ESAG) protein, putative,expression site-associated gene 1 (ESAG1) protein, putative | 0 |
| Tb927.1.4870 | expression site-associated gene (ESAG) protein, putative,expression site-associated gene 1 (ESAG1) protein, putative | 0 |
| Tb927.1.4910 | expression site-associated gene (ESAG) protein, putative,expression site-associated gene 1 (ESAG1) protein, putative | 0 |
| Tb927.1.5120 | expression site-associated gene (ESAG) protein, putative,expression site-associated gene 1 (ESAG1) protein, putative | 0 |
| Tb927.1.5200 | expression site-associated gene (ESAG) protein, putative,expression site-associated gene 1 (ESAG1) protein, putative | 0 |
| Tb927.3.2520 | expression site-associated gene (ESAG) protein, putative,expression site-associated gene 1 (ESAG1) protein, putative | 0 |
| Tb927.3.520 | expression site-associated gene (ESAG) protein, putative,expression site-associated gene 1 (ESAG1) protein, putative | 0 |
| Tb927.3.5830 | expression site-associated gene (ESAG) protein, putative,expression site-associated gene 1 (ESAG1) protein, putative | 0 |
| Tb927.4.1200 | expression site-associated gene (ESAG) protein, putative,expression site-associated gene 1 (ESAG1) protein, putative | 0 |
| Tb927.5.4630 | expression site-associated gene (ESAG) protein, putative,expression site-associated gene 1 (ESAG1) protein, putative | 0 |
| Tb11.01.6250 | expression site-associated gene (ESAG) protein, putative,expression site-associated gene 11 (ESAG11) protein, putative | 0 |
| Tb927.3.560 | expression site-associated gene (ESAG) protein, putative,expression site-associated gene 11 (ESAG11) protein, putative | 0 |
| Tb11.01.6240 | expression site-associated gene (ESAG) protein, putative,expression site-associated gene 2 (ESAG2) protein, putative | 0 |
| Tb927.3.2510 | expression site-associated gene (ESAG) protein, putative,expression site-associated gene 2 (ESAG2) protein, putative | 0 |
| Tb927.3.570 | expression site-associated gene (ESAG) protein, putative,expression site-associated gene 2 (ESAG2) protein, putative | 0 |
| Tb927.4.3280 | expression site-associated gene (ESAG) protein, putative,expression site-associated gene 2 (ESAG2) protein, putative | 0 |
| Tb10.6k15.0910 | expression site-associated gene (ESAG) protein, putative,expression site-associated gene 3 (ESAG3) protein, putative | 2 |
| Tb10.6k15.0920 | expression site-associated gene (ESAG) protein, putative,expression site-associated gene 3 (ESAG3) protein, putative | 1 |
| Tb10.70.2860 | expression site-associated gene (ESAG) protein, putative,expression site-associated gene 3 (ESAG3) protein, putative | 4 |
| Tb11.55.0027 | expression site-associated gene (ESAG) protein, putative,expression site-associated gene 3 (ESAG3) protein, putative | 2 |
| Tb11.55.0028 | expression site-associated gene (ESAG) protein, putative,expression site-associated gene 3 (ESAG3) protein, putative | 2 |
| Tb927.5.4600 | expression site-associated gene (ESAG) protein, putative,expression site-associated gene 3 (ESAG3) protein, putative | 1 |
| Tb09.244.2120 | expression site-associated gene (ESAG) protein, putative,expression site-associated gene 5 (ESAG5) protein, putative | 1 |
| Tb927.2.1920 | expression site-associated gene (ESAG) protein, putative,expression site-associated gene 5 (ESAG5) protein, putative | 0 |
| Tb927.4.810 | expression site-associated gene (ESAG) protein, putative,expression site-associated gene 5 (ESAG5) protein, putative | 0 |
| Tb927.5.340 | expression site-associated gene (ESAG) protein, putative,expression site-associated gene 5 (ESAG5) protein, putative | 0 |
| Tb927.7.6860 | expression site-associated gene (ESAG) protein, putative,expression site-associated gene 5 (ESAG5) protein, putative | 1 |
| Tb927.7.3250 | expression site-associated gene (ESAG) protein, putative,expression site-associated gene 6 (ESAG6) protein, putative | 0 |
| Tb927.7.3260 | expression site-associated gene (ESAG) protein, putative,expression site-associated gene 7 (ESAG7) protein, putative | 0 |
| Tb927.1.5080 | expression site-associated gene (ESAG) protein, putative,expression site-associated gene 9 (ESAG9) protein, putative | 0 |
| Tb927.5.120 | expression site-associated gene (ESAG) protein, putative,expression site-associated gene 9 (ESAG9) protein, putative | 0 |

| | | |
|---|---|---|
| Tb927.5.4620 | expression site-associated gene (ESAG) protein, putative,expression site-associated gene 9 (ESAG9) protein, putative | 0 |
| Tb927.7.170 | expression site-associated gene (ESAG) protein, putative,expression site-associated gene 9 (ESAG9) protein, putative | 0 |
| Tb09.142.0310 | expression site-associated gene (ESAG) protein,expression site-associated gene 1 (ESAG1) protein | 0 |
| Tb927.1.5240 | expression site-associated gene 1 (ESAG1) protein, putative | 0 |
| Tb09.160.5380 | expression site-associated gene 11 (ESAG11) protein, putative | 1 |
| Tb09.v1.0310 | expression site-associated gene 11 (ESAG11) protein, putative | 1 |
| Tb927.1.4900 | expression site-associated gene 11 (ESAG11) protein, putative | 0 |
| Tb927.1.5110 | expression site-associated gene 11 (ESAG11) protein, putative | 0 |
| Tb09.160.5390 | expression site-associated gene 2 (ESAG2) protein, putative | 0 |
| Tb09.v1.0320 | expression site-associated gene 2 (ESAG2) protein, putative | 0 |
| Tb11.01.6230 | expression site-associated gene 2 (ESAG2) protein, putative | 0 |
| Tb927.1.2040 | expression site-associated gene 2 (ESAG2) protein, putative | 0 |
| Tb927.1.4890 | expression site-associated gene 2 (ESAG2) protein, putative | 0 |
| Tb927.1.5100 | expression site-associated gene 2 (ESAG2) protein, putative | 0 |
| Tb10.6k15.0900 | expression site-associated gene 3 (ESAG3)-like protein | 2 |
| Tb09.244.2380 | expression site-associated gene 4 (ESAG4) protein, putative,receptor-type adenylate cyclase, putative | 2 |
| Tb09.160.5400 | expression site-associated gene 9 (ESAG9) protein, putative | 0 |
| Tb09.160.5430 | expression site-associated gene 9 (ESAG9) protein, putative | 0 |
| Tb09.v1.0330 | expression site-associated gene 9 (ESAG9) protein, putative | 0 |
| Tb927.1.5220 | expression site-associated gene 9 (ESAG9) protein, putative | 0 |
| Tb11.01.0410 | ER lumen retaining receptor protein, putative | 4 |
| Tb11.01.3590 | FG-GAP repeat protein, putative,intergrin alpha chain protein, putative | 1 |
| Tb11.01.7770 | FG-GAP repeat protein, putative,intergrin alpha chain protein, putative | 1 |
| Tb927.7.6460 | FG-GAP repeat protein, putative,intergrin alpha chain protein, putative | 1 |
| Tb927.8.4010 | fla1 flagellum-adhesion glycoprotein | 2 |
| Tb927.8.4060 | flagellum-adhesion glycoprotein, putative | 2 |
| Tb927.8.4110 | flagellum-adhesion glycoprotein, putative | 2 |
| Tb11.01.3150 | gamma-tubulin complex subunit, putative,spindle pole body component alp6, putative | 1 |
| Tb927.4.5010 | calreticulin, putative | 1 |
| Tb927.8.7410 | calreticulin, putative | 1 |
| Tb11.01.0290 | carbonic anhydrase-like protein | 1 |
| Tb927.1.3230 | cell division cycle protein, putative | 0 |
| Tb11.18.0006 | CAT carnitine O-acetyltransferase, putative | 1 |
| Tb10.70.0120 | COP-coated vesicle membrane protein erv25 precursor, putative,ER--golgi transport protein erv25 precursor, putative | 2 |

| | | |
|---|---|---|
| Tb09.211.1380 | glycine cleavage system H protein, putative | 0 |
| Tb10.61.0980 | gMDH glycosomal malate dehydrogenase | 0 |
| Tb09.211.2830 | iron-sulfur cluster assembly protein, putative | 0 |
| Tb927.8.620 | KREPA3 RNA-editing complex protein MP42 | 0 |
| Tb10.70.2090 | KREPA6 RNA-editing complex protein,KREPA6 | 0 |
| Tb10.20.0130 | legume-like lectin, putative | 0 |
| Tb10.70.5100 | lysosomal alpha-mannosidase precursor, putative | 0 |
| Tb927.5.1810 | lysosomal/endosomal membrane protein p67,lysosomal membrane glycoprotein | 2 |
| Tb927.5.1830 | lysosomal/endosomal membrane protein p67,lysosomal membrane glycoprotein | 2 |
| Tb927.6.2790 | L-threonine 3-dehydrogenase, putative | 0 |
| Tb10.70.4420 | methionine biosynthetic protein, putative | 0 |
| Tb927.5.940 | NADH-dependent fumarate reductase, putative | 1 |
| Tb927.5.450 | NADH-ubiquinone oxidoreductase, mitochondrial, putative | 0 |
| Tb09.160.3820 | nucleolar RNA binding protein, putative | 1 |
| Tb11.01.7290 | NUDIX hydrolase, conserved | 0 |
| Tb09.160.5260 | oxidoreductase, putative | 0 |
| Tb927.6.2420 | p22 protein precursor | 0 |
| Tb927.3.1070 | peptide chain release factor 1, putative | 0 |
| Tb927.4.2510 | PGPA multidrug resistance-associated protein, putative,P-glycoprotein | 12 |
| Tb927.6.4600 | pre-mRNA splicing factor ATP-dependent RNA helicase, putative,ATP-dependent RNA helicase, putative | 1 |
| Tb927.6.4600 | pre-mRNA splicing factor ATP-dependent RNA helicase, putative,ATP-dependent RNA helicase, putative | 1 |
| Tb927.8.4810 | prohibitin | 0 |
| Tb10.100.0190 | pumillio RNA binding protein, putative | 1 |
| Tb11.02.5210 | RNA binding protein, putative | 1 |
| Tb11.01.6260 | RNA helicase, putative,DEAD/DEAH box helicase, putative | 1 |
| Tb927.4.1990 | RNA helicase, putative,mitochondrial, putative | 0 |
| Tb927.5.3750 | RNA-binding protein, putative | 0 |
| Tb927.6.4530 | RNA-binding protein, putative | 0 |
| Tb927.6.4530 | RNA-binding protein, putative | 0 |
| Tb10.61.1360 | RPC11 RNA polymerase III C11 subunit, putative,DNA-directed RNA polymerase III, putative,RNA polymerase subunit, | 0 |
| Tb09.160.4450 | RPS3 40S ribosomal protein S3, putative | 0 |
| Tb927.7.4670 | RRP4 ribosomal RNA processing protein 4,exosome complex exonuclease | 1 |
| Tb927.4.1630 | RRP6 ribosomal RNA processing protein 6 | 0 |
| Tb10.70.1690 | 40S ribosomal protein S10, putative | 1 |

| | | |
|---|---|---|
| Tb11.01.2680 | 40S ribosomal protein SA, putative | 0 |
| Tb927.8.5860 | 50S ribosomal protein L17, putative | 0 |
| Tb09.211.4550 | 60S ribosomal protein L12, putative | 0 |
| Tb927.4.1790 | ribosomal protein L3, putative | 1 |
| Tb927.5.1710 | ribonucleoprotein p18, mitochondrial precursor, putative | 0 |
| Tb927.7.7420 | ATP synthase alpha chain, mitochondrial precursor,ATP synthase F1, alpha subunit | 0 |
| Tb927.7.7430 | ATP synthase alpha chain, mitochondrial precursor,ATP synthase F1, alpha subunit | 0 |
| Tb927.3.1380 | ATP synthase beta chain, mitochondrial precursor,ATP synthase F1, beta subunit | 0 |
| Tb927.6.4990 | ATP synthase, epsilon chain, putative | 0 |
| Tb927.6.4990 | ATP synthase, epsilon chain, putative | 0 |
| Tb11.02.2950 | ATPase subunit 9, putative | 2 |
| Tb927.7.1470 | ATPase subunit 9, putative | 2 |
| Tb927.7.6930 | ATPase, putative | 0 |
| Tb10.61.2840 | vacuolar ATP synthase subunit c, putative | 0 |
| Tb10.100.0090 | vacuolar ATP synthase, putative | 5 |
| Tb927.5.2790 | Pol beta-PAK mitochondrial DNA polymerase beta-PAK | 0 |
| Tb11.01.3660 | DNA repair and recombination helicase protein PIF1, putative,DNA repair and recombination protein, mitochondrial precursor, putative | 0 |
| Tb927.4.4380 | PPase1 vacuolar-type proton translocating pyrophosphatase 1, putative | 14 |
| Tb10.70.5110 | mMDH mitochondrial malate dehydrogenase | 0 |
| Tb11.01.5040 | mitochondrial carrier protein, putative | 0 |
| Tb927.8.3330 | mitochondrial carrier protein, putative | 2 |
| Tb927.8.1870 | tGLP1 Golgi/lysosome glycoprotein 1 | 1 |
| Tb927.8.7980 | TVP1 vacuolar-type proton translocating pyrophosphatase 1,V-type H(+)-translocating pyrophosphatase | 14 |
| Tb927.5.860 | SelT selenoprotein, putative | 3 |
| Tb10.70.0630 | ALDH aldehyde dehydrogenase, putative | 0 |
| Tb927.6.4210 | ALDH aldehyde dehydrogenase, putative | 0 |
| Tb10.389.1500 | short-chain dehydrogenase, putative | 1 |
| Tb10.6k15.3880 | short-chain dehydrogenase, putative | 1 |
| Tb10.70.3240 | short-chain dehydrogenase, putative | 1 |
| Tb11.01.1780 | short-chain dehydrogenase, putative | 2 |
| Tb927.5.1210 | short-chain dehydrogenase, putative | 2 |
| Tb09.142.0240 | variant surface glycoprotein (VSG), putative | 0 |
| Tb09.244.0470 | variant surface glycoprotein (VSG), putative | 0 |
| Tb09.244.1410 | variant surface glycoprotein (VSG), putative | 0 |

| | | |
|---|---|---|
| Tb09.244.1740 | variant surface glycoprotein (VSG), putative | 0 |
| Tb09.354.0090 | variant surface glycoprotein (VSG), putative | 0 |
| Tb09.v4.0004 | variant surface glycoprotein (VSG), putative | 0 |
| Tb09.v4.0005 | variant surface glycoprotein (VSG), putative | 0 |
| Tb10.v4.0024 | variant surface glycoprotein (VSG), putative | 0 |
| Tb10.v4.0025 | variant surface glycoprotein (VSG), putative | 0 |
| Tb10.v4.0031 | variant surface glycoprotein (VSG), putative | 0 |
| Tb11.09.0003 | variant surface glycoprotein (VSG), putative | 0 |
| Tb11.16.0001 | variant surface glycoprotein (VSG), putative | 0 |
| Tb11.16.0002 | variant surface glycoprotein (VSG), putative | 0 |
| Tb11.16.0003 | variant surface glycoprotein (VSG), putative | 0 |
| Tb11.38.0003 | variant surface glycoprotein (VSG), putative | 0 |
| Tb11.51.0005 | variant surface glycoprotein (VSG), putative | 0 |
| Tb11.57.0019 | variant surface glycoprotein (VSG), putative | 0 |
| Tb11.57.0024 | variant surface glycoprotein (VSG), putative | 0 |
| Tb11.57.0035 | variant surface glycoprotein (VSG), putative | 0 |
| Tb11.57.0084 | variant surface glycoprotein (VSG), putative | 0 |
| Tb11.v4.0010 | variant surface glycoprotein (VSG), putative | 0 |
| Tb11.v4.0015 | variant surface glycoprotein (VSG), putative | 0 |
| Tb11.v4.0016 | variant surface glycoprotein (VSG), putative | 0 |
| Tb11.v4.0029 | variant surface glycoprotein (VSG), putative | 0 |
| Tb11.v4.0033 | variant surface glycoprotein (VSG), putative | 0 |
| Tb11.v4.0034 | variant surface glycoprotein (VSG), putative | 0 |
| Tb11.v4.0035 | variant surface glycoprotein (VSG), putative | 0 |
| Tb11.v4.0036 | variant surface glycoprotein (VSG), putative | 0 |
| Tb11.v4.0038 | variant surface glycoprotein (VSG), putative | 0 |
| Tb11.v4.0040 | variant surface glycoprotein (VSG), putative | 0 |
| Tb11.v4.0050 | variant surface glycoprotein (VSG), putative | 0 |
| Tb11.v4.0058 | variant surface glycoprotein (VSG), putative | 1 |
| Tb11.v4.0063 | variant surface glycoprotein (VSG), putative | 0 |
| Tb11.v4.0065 | variant surface glycoprotein (VSG), putative | 1 |
| Tb11.v4.0067 | variant surface glycoprotein (VSG), putative | 0 |
| Tb11.v4.0068 | variant surface glycoprotein (VSG), putative | 0 |
| Tb11.v4.0070 | variant surface glycoprotein (VSG), putative | 0 |

| | | |
|---|---|---|
| Tb927.1.05 | variant surface glycoprotein (VSG), putative | 0 |
| Tb927.1.5300 | variant surface glycoprotein (VSG), putative | 0 |
| Tb927.3.150 | variant surface glycoprotein (VSG), putative | 0 |
| Tb927.3.190 | variant surface glycoprotein (VSG), putative | 0 |
| Tb927.3.340 | variant surface glycoprotein (VSG), putative | 0 |
| Tb927.3.440 | variant surface glycoprotein (VSG), putative | 0 |
| Tb927.3.490 | variant surface glycoprotein (VSG), putative | 0 |
| Tb927.4.5460 | variant surface glycoprotein (VSG), putative | 1 |
| Tb927.4.5530 | variant surface glycoprotein (VSG), putative | 0 |
| Tb927.4.5560 | variant surface glycoprotein (VSG), putative | 0 |
| Tb927.5.4770 | variant surface glycoprotein (VSG), putative | 0 |
| Tb927.5.5080 | variant surface glycoprotein (VSG), putative | 0 |
| Tb927.5.5240 | variant surface glycoprotein (VSG), putative | 0 |
| Tb927.6.5260 | variant surface glycoprotein (VSG), putative | 0 |
| Tb927.6.5370 | variant surface glycoprotein (VSG), putative | 0 |
| Tb927.7.6500 | variant surface glycoprotein (VSG), putative | 0 |
| Tb10.v4.0001 | variant surface glycoprotein (VSG),variant surface glycoprotein VSG 10.1 | 0 |
| Tb09.160.5350 | variant surface glycoprotein (VSG)-related, putative | 0 |
| Tb09.244.2200 | variant surface glycoprotein (VSG)-related, putative | 0 |
| Tb09.244.2240 | variant surface glycoprotein (VSG)-related, putative | 0 |
| Tb09.244.2280 | variant surface glycoprotein (VSG)-related, putative | 0 |
| Tb09.244.2310 | variant surface glycoprotein (VSG)-related, putative | 0 |
| Tb09.244.2330 | variant surface glycoprotein (VSG)-related, putative | 0 |
| Tb09.v1.0300 | variant surface glycoprotein (VSG)-related, putative | 0 |
| Tb11.01.4560 | variant surface glycoprotein (VSG)-related, putative | 0 |
| Tb11.02.1566 | variant surface glycoprotein (VSG)-related, putative | 0 |
| Tb927.1.5060 | variant surface glycoprotein (VSG)-related, putative | 0 |
| Tb927.1.5170 | variant surface glycoprotein (VSG)-related, putative | 0 |
| Tb927.2.2060 | variant surface glycoprotein (VSG)-related, putative | 0 |
| Tb927.3.1470 | variant surface glycoprotein (VSG)-related, putative | 14 |
| Tb927.3.1500 | variant surface glycoprotein (VSG)-related, putative | 0 |
| Tb927.3.1510 | variant surface glycoprotein (VSG)-related, putative | 0 |
| Tb927.3.1520 | variant surface glycoprotein (VSG)-related, putative | 0 |
| Tb927.3.2540 | variant surface glycoprotein (VSG)-related, putative | 0 |

| | | |
|---|---|---|
| Tb927.3.5680 | variant surface glycoprotein (VSG)-related, putative | 0 |
| Tb927.5.110 | variant surface glycoprotein (VSG)-related, putative | 0 |
| Tb927.5.130 | variant surface glycoprotein (VSG)-related, putative | 0 |
| Tb927.8.7300 | variant surface glycoprotein (VSG)-related, putative | 0 |
| Tb927.8.7320 | variant surface glycoprotein (VSG)-related, putative | 0 |
| Tb09.142.0100 | variant surface glycoprotein (VSG, atypical), putative | 0 |
| Tb09.142.0230 | variant surface glycoprotein (VSG, atypical), putative | 0 |
| Tb09.160.0160 | variant surface glycoprotein (VSG, atypical), putative | 0 |
| Tb09.160.0280 | variant surface glycoprotein (VSG, atypical), putative | 0 |
| Tb09.244.0050 | variant surface glycoprotein (VSG, atypical), putative | 0 |
| Tb09.244.0200 | variant surface glycoprotein (VSG, atypical), putative | 0 |
| Tb09.244.0250 | variant surface glycoprotein (VSG, atypical), putative | 0 |
| Tb09.244.0640 | variant surface glycoprotein (VSG, atypical), putative | 0 |
| Tb09.244.1000 | variant surface glycoprotein (VSG, atypical), putative | 1 |
| Tb09.244.1570 | variant surface glycoprotein (VSG, atypical), putative | 0 |
| Tb09.244.1600 | variant surface glycoprotein (VSG, atypical), putative | 0 |
| Tb09.244.1790 | variant surface glycoprotein (VSG, atypical), putative | 0 |
| Tb09.244.1830 | variant surface glycoprotein (VSG, atypical), putative | 0 |
| Tb09.354.0060 | variant surface glycoprotein (VSG, atypical), putative | 0 |
| Tb09.354.0180 | variant surface glycoprotein (VSG, atypical), putative | 0 |
| Tb11.13.0004 | variant surface glycoprotein (VSG, atypical), putative | 0 |
| Tb11.14.0007 | variant surface glycoprotein (VSG, atypical), putative | 0 |
| Tb11.14.0008 | variant surface glycoprotein (VSG, atypical), putative | 0 |
| Tb11.14.0019 | variant surface glycoprotein (VSG, atypical), putative | 0 |
| Tb11.21.0004 | variant surface glycoprotein (VSG, atypical), putative | 0 |
| Tb11.24.0007 | variant surface glycoprotein (VSG, atypical), putative | 0 |
| Tb11.24.0011 | variant surface glycoprotein (VSG, atypical), putative | 0 |
| Tb11.24.0012 | variant surface glycoprotein (VSG, atypical), putative | 0 |
| Tb11.35.0001 | variant surface glycoprotein (VSG, atypical), putative | 0 |
| Tb11.38.0005 | variant surface glycoprotein (VSG, atypical), putative | 0 |
| Tb11.57.0052 | variant surface glycoprotein (VSG, atypical), putative | 0 |
| Tb927.1.5330 | variant surface glycoprotein (VSG, atypical), putative | 0 |
| Tb927.2.6410 | variant surface glycoprotein (VSG, atypical), putative | 0 |
| Tb927.3.180 | variant surface glycoprotein (VSG, atypical), putative | 1 |

| | | |
|---|---|---|
| Tb927.3.210 | variant surface glycoprotein (VSG, atypical), putative | 0 |
| Tb927.3.370 | variant surface glycoprotein (VSG, atypical), putative | 1 |
| Tb927.4.5400 | variant surface glycoprotein (VSG, atypical), putative | 1 |
| Tb927.4.5410 | variant surface glycoprotein (VSG, atypical), putative | 0 |
| Tb927.4.5420 | variant surface glycoprotein (VSG, atypical), putative | 0 |
| Tb927.4.5430 | variant surface glycoprotein (VSG, atypical), putative | 0 |
| Tb927.4.5580 | variant surface glycoprotein (VSG, atypical), putative | 0 |
| Tb927.4.5700 | variant surface glycoprotein (VSG, atypical), putative | 0 |
| Tb927.5.230 | variant surface glycoprotein (VSG, atypical), putative | 0 |
| Tb927.5.3990 | variant surface glycoprotein (VSG, atypical), putative | 0 |
| Tb927.5.4670 | variant surface glycoprotein (VSG, atypical), putative | 0 |
| Tb927.5.4690 | variant surface glycoprotein (VSG, atypical), putative | 0 |
| Tb927.5.4730 | variant surface glycoprotein (VSG, atypical), putative | 0 |
| Tb927.5.4810 | variant surface glycoprotein (VSG, atypical), putative | 0 |
| Tb927.5.4840 | variant surface glycoprotein (VSG, atypical), putative | 0 |
| Tb927.5.4950 | variant surface glycoprotein (VSG, atypical), putative | 0 |
| Tb927.5.5050 | variant surface glycoprotein (VSG, atypical), putative | 0 |
| Tb927.5.5210 | variant surface glycoprotein (VSG, atypical), putative | 0 |
| Tb927.6.5240 | variant surface glycoprotein (VSG, atypical), putative | 0 |
| Tb927.6.5550 | variant surface glycoprotein (VSG, atypical), putative | 0 |
| Tb927.6.5740 | variant surface glycoprotein (VSG, atypical), putative | 0 |
| Tb927.7.6540 | variant surface glycoprotein (VSG, atypical), putative | 1 |
| Tb927.8.170 | variant surface glycoprotein (VSG, atypical), putative | 1 |
| Tb927.8.460 | variant surface glycoprotein (VSG, atypical), putative | 0 |
| Tb927.6.4960 | zinc finger-domain protein, putative | 1 |
| Tb927.6.4960 | zinc finger-domain protein, putative | 1 |
| Tb927.8.6010 | hypothetical predicted multi-pass transmembrane protein | 3 |
| Tb09.142.0430 | hypothetical protein | 1 |
| Tb09.160.5510 | hypothetical protein | 3 |
| Tb09.211.0250 | hypothetical protein | 5 |
| Tb09.211.4070 | hypothetical protein | 0 |
| Tb09.211.4950 | hypothetical protein | 2 |
| Tb09.244.0370 | hypothetical protein | 0 |
| Tb09.244.0810 | hypothetical protein | 0 |

| | | |
|---|---|---|
| Tb09.244.2390 | hypothetical protein | 1 |
| Tb09.v4.0006 | hypothetical protein | 0 |
| Tb10.26.0190 | hypothetical protein | 1 |
| Tb10.389.0400 | hypothetical protein | 0 |
| Tb10.389.1100 | hypothetical protein | 1 |
| Tb10.61.2500 | hypothetical protein | 2 |
| Tb10.6k15.0730 | hypothetical protein | 3 |
| Tb10.6k15.0880 | hypothetical protein | 0 |
| Tb10.6k15.0940 | hypothetical protein | 1 |
| Tb10.6k15.1610 | hypothetical protein | 2 |
| Tb10.70.0560 | hypothetical protein | 0 |
| Tb10.70.1260 | hypothetical protein | 0 |
| Tb10.70.2840 | hypothetical protein | 0 |
| Tb10.70.2850 | hypothetical protein | 0 |
| Tb10.70.3250 | hypothetical protein | 1 |
| Tb10.70.3310 | hypothetical protein | 3 |
| Tb10.70.3780 | hypothetical protein | 0 |
| Tb10.70.4020 | hypothetical protein | 2 |
| Tb10.70.4480 | hypothetical protein | 0 |
| Tb10.70.5370 | hypothetical protein | 1 |
| Tb10.70.5580 | hypothetical protein | 0 |
| Tb10.70.6250 | hypothetical protein | 0 |
| Tb10.v4.0017 | hypothetical protein | 0 |
| Tb11.01.0550 | hypothetical protein | 0 |
| Tb11.01.2860 | hypothetical protein | 0 |
| Tb11.01.3220 | hypothetical protein | 0 |
| Tb11.01.3810 | hypothetical protein | 0 |
| Tb11.01.7870 | hypothetical protein | 2 |
| Tb11.02.1565 | hypothetical protein | 0 |
| Tb11.02.1850 | hypothetical protein | 2 |
| Tb11.02.2180 | hypothetical protein | 3 |
| Tb11.02.2200 | hypothetical protein | 4 |
| Tb11.02.2360 | hypothetical protein | 0 |
| Tb11.02.2370 | hypothetical protein | 0 |

| | | |
|---|---|---|
| Tb11.02.4690 | hypothetical protein | 3 |
| Tb11.02.5690 | hypothetical protein | 4 |
| Tb11.12.0017 | hypothetical protein | 0 |
| Tb11.42.0001 | hypothetical protein | 0 |
| Tb11.v4.0002 | hypothetical protein | 2 |
| Tb11.v4.0008 | hypothetical protein | 0 |
| Tb927.1.10 | hypothetical protein | 1 |
| Tb927.1.2310 | hypothetical protein | 2 |
| Tb927.1.2540 | hypothetical protein | 0 |
| Tb927.1.4020 | hypothetical protein | 5 |
| Tb927.2.1600 | hypothetical protein | 1 |
| Tb927.2.2490 | hypothetical protein | 1 |
| Tb927.2.4440 | hypothetical protein | 6 |
| Tb927.2.680 | hypothetical protein | 1 |
| Tb927.2.940 | hypothetical protein | 0 |
| Tb927.3.1460 | hypothetical protein | 0 |
| Tb927.3.1650 | hypothetical protein | 2 |
| Tb927.3.2500 | hypothetical protein | 0 |
| Tb927.3.2590 | hypothetical protein | 0 |
| Tb927.3.2800 | hypothetical protein | 1 |
| Tb927.3.3700 | hypothetical protein | 2 |
| Tb927.3.5670 | hypothetical protein | 1 |
| Tb927.3.600 | hypothetical protein | 1 |
| Tb927.4.1010 | hypothetical protein | 2 |
| Tb927.4.140 | hypothetical protein | 0 |
| Tb927.4.2100 | hypothetical protein | 0 |
| Tb927.4.2690 | hypothetical protein | 5 |
| Tb927.4.3480 | hypothetical protein | 4 |
| Tb927.4.4770 | hypothetical protein | 1 |
| Tb927.5.1740 | hypothetical protein | 1 |
| Tb927.5.310 | hypothetical protein | 1 |
| Tb927.5.4010 | hypothetical protein | 0 |
| Tb927.5.4020 | hypothetical protein | 0 |
| Tb927.5.410 | hypothetical protein | 1 |

| | | |
|---|---|---|
| Tb927.5.420 | hypothetical protein | 1 |
| Tb927.5.430 | hypothetical protein | 1 |
| Tb927.5.620 | hypothetical protein | 1 |
| Tb927.6.1150 | hypothetical protein | 3 |
| Tb927.6.120 | hypothetical protein | 1 |
| Tb927.6.1300 | hypothetical protein | 3 |
| Tb927.6.1790 | hypothetical protein | 4 |
| Tb927.6.2530 | hypothetical protein | 4 |
| Tb927.6.4110 | hypothetical protein | 3 |
| Tb927.6.420 | hypothetical protein | 1 |
| Tb927.6.440 | hypothetical protein | 0 |
| Tb927.6.4730 | hypothetical protein | 4 |
| Tb927.6.4730 | hypothetical protein | 4 |
| Tb927.7.180 | hypothetical protein | 0 |
| Tb927.7.3690 | hypothetical protein | 3 |
| Tb927.7.3720 | hypothetical protein | 4 |
| Tb927.7.4690 | hypothetical protein | 1 |
| Tb927.7.470 | hypothetical protein | 2 |
| Tb927.7.5860 | hypothetical protein | 4 |
| Tb927.7.6100 | hypothetical protein | 1 |
| Tb927.7.6580 | hypothetical protein | 0 |
| Tb927.7.7310 | hypothetical protein | 2 |
| Tb927.8.1760 | hypothetical protein | 1 |
| Tb927.8.2320 | hypothetical protein | 3 |
| Tb927.8.3670 | hypothetical protein | 2 |
| Tb927.8.3760 | hypothetical protein | 3 |
| Tb927.8.4300 | hypothetical protein | 3 |
| Tb927.8.490 | hypothetical protein | 0 |
| Tb927.8.510 | hypothetical protein | 0 |
| Tb927.8.510 | hypothetical protein | 0 |
| Tb927.8.5790 | hypothetical protein | 0 |
| Tb927.8.5910 | hypothetical protein | 0 |
| Tb927.8.5920 | hypothetical protein | 0 |
| Tb927.8.7190 | hypothetical protein | 0 |

| | | |
|---|---|---|
| Tb927.8.7310 | hypothetical protein | 0 |
| Tb927.8.7330 | hypothetical protein | 0 |
| Tb927.8.930 | hypothetical protein | 0 |
| Tb927.8.950 | hypothetical protein | 0 |
| Tb927.8.970 | hypothetical protein | 0 |
| Tb09.142.0370 | hypothetical protein, conserved | 0 |
| Tb09.142.0440 | hypothetical protein, conserved | 2 |
| Tb09.160.0400 | hypothetical protein, conserved | 0 |
| Tb09.160.0430 | hypothetical protein, conserved | 10 |
| Tb09.160.0550 | hypothetical protein, conserved | 0 |
| Tb09.160.1010 | hypothetical protein, conserved | 8 |
| Tb09.160.1030 | hypothetical protein, conserved | 0 |
| Tb09.160.1490 | hypothetical protein, conserved | 0 |
| Tb09.160.1520 | hypothetical protein, conserved | 1 |
| Tb09.160.2060 | hypothetical protein, conserved | 1 |
| Tb09.160.3140 | hypothetical protein, conserved | 0 |
| Tb09.160.3490 | hypothetical protein, conserved | 0 |
| Tb09.160.3780 | hypothetical protein, conserved | 6 |
| Tb09.160.4660 | hypothetical protein, conserved | 0 |
| Tb09.160.4690 | hypothetical protein, conserved | 2 |
| Tb09.160.4800 | hypothetical protein, conserved | 5 |
| Tb09.160.5040 | hypothetical protein, conserved | 3 |
| Tb09.160.5180 | hypothetical protein, conserved | 0 |
| Tb09.160.5310 | hypothetical protein, conserved | 3 |
| Tb09.211.0010 | hypothetical protein, conserved | 0 |
| Tb09.211.0020 | hypothetical protein, conserved | 1 |
| Tb09.211.0450 | hypothetical protein, conserved | 3 |
| Tb09.211.0800 | hypothetical protein, conserved | 0 |
| Tb09.211.0990 | hypothetical protein, conserved | 1 |
| Tb09.211.1270 | hypothetical protein, conserved | 0 |
| Tb09.211.1280 | hypothetical protein, conserved | 0 |
| Tb09.211.1290 | hypothetical protein, conserved | 1 |
| Tb09.211.1300 | hypothetical protein, conserved | 0 |
| Tb09.211.1360 | hypothetical protein, conserved | 3 |

| | | |
|---|---|---|
| Tb09.211.1390 | hypothetical protein, conserved | 0 |
| Tb09.211.1600 | hypothetical protein, conserved | 0 |
| Tb09.211.1670 | hypothetical protein, conserved | 4 |
| Tb09.211.1810 | hypothetical protein, conserved | 0 |
| Tb09.211.1890 | hypothetical protein, conserved | 2 |
| Tb09.211.1900 | hypothetical protein, conserved | 0 |
| Tb09.211.2200 | hypothetical protein, conserved | 0 |
| Tb09.211.2810 | hypothetical protein, conserved | 0 |
| Tb09.211.3530 | hypothetical protein, conserved | 1 |
| Tb09.211.3660 | hypothetical protein, conserved | 1 |
| Tb09.211.3720 | hypothetical protein, conserved | 0 |
| Tb09.211.3740 | hypothetical protein, conserved | 11 |
| Tb09.211.3800 | hypothetical protein, conserved | 0 |
| Tb09.211.3900 | hypothetical protein, conserved | 0 |
| Tb09.211.4155 | hypothetical protein, conserved | 2 |
| Tb09.211.4630 | hypothetical protein, conserved | 1 |
| Tb09.211.4680 | hypothetical protein, conserved | 2 |
| Tb09.211.4770 | hypothetical protein, conserved | 1 |
| Tb09.211.4800 | hypothetical protein, conserved | 7 |
| Tb09.244.2190 | hypothetical protein, conserved | 0 |
| Tb09.244.2230 | hypothetical protein, conserved | 0 |
| Tb09.244.2270 | hypothetical protein, conserved | 0 |
| Tb09.244.2770 | hypothetical protein, conserved | 2 |
| Tb09.244.2830 | hypothetical protein, conserved | 1 |
| Tb09.v1.0450 | hypothetical protein, conserved | 0 |
| Tb09.v1.0460 | hypothetical protein, conserved | 0 |
| Tb09.v1.0470 | hypothetical protein, conserved | 0 |
| Tb09.v1.0480 | hypothetical protein, conserved | 0 |
| Tb09.v1.0500 | hypothetical protein, conserved | 0 |
| Tb09.v1.0510 | hypothetical protein, conserved | 1 |
| Tb09.v1.0530 | hypothetical protein, conserved | 0 |
| Tb09.v1.0540 | hypothetical protein, conserved | 1 |
| Tb09.v1.0650 | hypothetical protein, conserved | 2 |
| Tb09.v1.0770 | hypothetical protein, conserved | 3 |

| | | |
|---|---|---|
| Tb10.100.0150 | hypothetical protein, conserved | 1 |
| Tb10.100.0200 | hypothetical protein, conserved | 2 |
| Tb10.26.0630 | hypothetical protein, conserved | 0 |
| Tb10.26.0690 | hypothetical protein, conserved | 0 |
| Tb10.26.0900 | hypothetical protein, conserved | 2 |
| Tb10.26.0910 | hypothetical protein, conserved | 0 |
| Tb10.389.0030 | hypothetical protein, conserved | 1 |
| Tb10.389.0045 | hypothetical protein, conserved | 1 |
| Tb10.389.0370 | hypothetical protein, conserved | 2 |
| Tb10.389.0610 | hypothetical protein, conserved | 0 |
| Tb10.389.0950 | hypothetical protein, conserved | 3 |
| Tb10.389.1220 | hypothetical protein, conserved | 9 |
| Tb10.389.1250 | hypothetical protein, conserved | 1 |
| Tb10.389.1260 | hypothetical protein, conserved | 1 |
| Tb10.389.1780 | hypothetical protein, conserved | 0 |
| Tb10.406.0030 | hypothetical protein, conserved | 1 |
| Tb10.406.0080 | hypothetical protein, conserved | 4 |
| Tb10.406.0110 | hypothetical protein, conserved | 0 |
| Tb10.61.0460 | hypothetical protein, conserved | 0 |
| Tb10.61.0480 | hypothetical protein, conserved | 0 |
| Tb10.61.1115 | hypothetical protein, conserved | 2 |
| Tb10.61.1440 | hypothetical protein, conserved | 4 |
| Tb10.61.2390 | hypothetical protein, conserved | 0 |
| Tb10.61.2720 | hypothetical protein, conserved | 1 |
| Tb10.61.2850 | hypothetical protein, conserved | 0 |
| Tb10.61.3170 | hypothetical protein, conserved | 1 |
| Tb10.6k15.0080 | hypothetical protein, conserved | 0 |
| Tb10.6k15.0150 | hypothetical protein, conserved | 0 |
| Tb10.6k15.0160 | hypothetical protein, conserved | 0 |
| Tb10.6k15.0180 | hypothetical protein, conserved | 0 |
| Tb10.6k15.0240 | hypothetical protein, conserved | 8 |
| Tb10.6k15.0280 | hypothetical protein, conserved | 0 |
| Tb10.6k15.0300 | hypothetical protein, conserved | 1 |
| Tb10.6k15.0480 | hypothetical protein, conserved | 0 |

| | | |
|---|---|---|
| Tb10.6k15.1030 | hypothetical protein, conserved | 0 |
| Tb10.6k15.1040 | hypothetical protein, conserved | 0 |
| Tb10.6k15.1110 | hypothetical protein, conserved | 0 |
| Tb10.6k15.1130 | hypothetical protein, conserved | 1 |
| Tb10.6k15.1210 | hypothetical protein, conserved | 1 |
| Tb10.6k15.1270 | hypothetical protein, conserved | 0 |
| Tb10.6k15.1640 | hypothetical protein, conserved | 6 |
| Tb10.6k15.1780 | hypothetical protein, conserved | 3 |
| Tb10.6k15.1800 | hypothetical protein, conserved | 2 |
| Tb10.6k15.1870 | hypothetical protein, conserved | 0 |
| Tb10.6k15.2300 | hypothetical protein, conserved | 0 |
| Tb10.6k15.2390 | hypothetical protein, conserved | 2 |
| Tb10.6k15.2580 | hypothetical protein, conserved | 7 |
| Tb10.6k15.2820 | hypothetical protein, conserved | 0 |
| Tb10.6k15.3420 | hypothetical protein, conserved | 5 |
| Tb10.6k15.3660 | hypothetical protein, conserved | 0 |
| Tb10.70.0440 | hypothetical protein, conserved | 4 |
| Tb10.70.0640 | hypothetical protein, conserved | 1 |
| Tb10.70.0770 | hypothetical protein, conserved | 1 |
| Tb10.70.1280 | hypothetical protein, conserved | 0 |
| Tb10.70.1290 | hypothetical protein, conserved | 0 |
| Tb10.70.1450 | hypothetical protein, conserved | 0 |
| Tb10.70.1640 | hypothetical protein, conserved | 3 |
| Tb10.70.1650 | hypothetical protein, conserved | 0 |
| Tb10.70.2120 | hypothetical protein, conserved | 1 |
| Tb10.70.2130 | hypothetical protein, conserved | 6 |
| Tb10.70.2150 | hypothetical protein, conserved | 0 |
| Tb10.70.2450 | hypothetical protein, conserved | 3 |
| Tb10.70.2540 | hypothetical protein, conserved | 1 |
| Tb10.70.2690 | hypothetical protein, conserved | 3 |
| Tb10.70.2990 | hypothetical protein, conserved | 10 |
| Tb10.70.3125 | hypothetical protein, conserved | 2 |
| Tb10.70.3185 | hypothetical protein, conserved | 2 |
| Tb10.70.3630 | hypothetical protein, conserved | 0 |

| | | |
|---|---|---|
| Tb10.70.3750 | hypothetical protein, conserved | 6 |
| Tb10.70.3940 | hypothetical protein, conserved | 0 |
| Tb10.70.4000 | hypothetical protein, conserved | 4 |
| Tb10.70.4110 | hypothetical protein, conserved | 1 |
| Tb10.70.4150 | hypothetical protein, conserved | 2 |
| Tb10.70.4430 | hypothetical protein, conserved | 0 |
| Tb10.70.4590 | hypothetical protein, conserved | 0 |
| Tb10.70.4610 | hypothetical protein, conserved | 1 |
| Tb10.70.5180 | hypothetical protein, conserved | 1 |
| Tb10.70.5630 | hypothetical protein, conserved | 0 |
| Tb10.70.5690 | hypothetical protein, conserved | 1 |
| Tb10.70.6720 | hypothetical protein, conserved | 0 |
| Tb10.70.6740 | hypothetical protein, conserved | 0 |
| Tb10.70.7150 | hypothetical protein, conserved | 0 |
| Tb10.70.7460 | hypothetical protein, conserved | 1 |
| Tb10.70.7510 | hypothetical protein, conserved | 2 |
| Tb10.70.7520 | hypothetical protein, conserved | 5 |
| Tb11.01.0070 | hypothetical protein, conserved | 0 |
| Tb11.01.0130 | hypothetical protein, conserved | 0 |
| Tb11.01.0240 | hypothetical protein, conserved | 0 |
| Tb11.01.0415 | hypothetical protein, conserved | 2 |
| Tb11.01.0590 | hypothetical protein, conserved | 4 |
| Tb11.01.0880 | hypothetical protein, conserved | 0 |
| Tb11.01.1000 | hypothetical protein, conserved | 1 |
| Tb11.01.1220 | hypothetical protein, conserved | 0 |
| Tb11.01.1340 | hypothetical protein, conserved | 3 |
| Tb11.01.1590 | hypothetical protein, conserved | 0 |
| Tb11.01.1660 | hypothetical protein, conserved | 0 |
| Tb11.01.2470 | hypothetical protein, conserved | 0 |
| Tb11.01.2640 | hypothetical protein, conserved | 2 |
| Tb11.01.2690 | hypothetical protein, conserved | 0 |
| Tb11.01.2740 | hypothetical protein, conserved | 0 |
| Tb11.01.3160 | hypothetical protein, conserved | 1 |
| Tb11.01.3540 | hypothetical protein, conserved | 6 |

| | | |
|---|---|---|
| Tb11.01.3570 | hypothetical protein, conserved | 0 |
| Tb11.01.3790 | hypothetical protein, conserved | 2 |
| Tb11.01.3860 | hypothetical protein, conserved | 0 |
| Tb11.01.4110 | hypothetical protein, conserved | 1 |
| Tb11.01.4220 | hypothetical protein, conserved | 7 |
| Tb11.01.4290 | hypothetical protein, conserved | 0 |
| Tb11.01.4530 | hypothetical protein, conserved | 0 |
| Tb11.01.4740 | hypothetical protein, conserved | 1 |
| Tb11.01.4900 | hypothetical protein, conserved | 0 |
| Tb11.01.5120 | hypothetical protein, conserved | 1 |
| Tb11.01.5160 | hypothetical protein, conserved | 1 |
| Tb11.01.5190 | hypothetical protein, conserved | 1 |
| Tb11.01.5200 | hypothetical protein, conserved | 0 |
| Tb11.01.5280 | hypothetical protein, conserved | 2 |
| Tb11.01.5760 | hypothetical protein, conserved | 1 |
| Tb11.01.5850 | hypothetical protein, conserved | 1 |
| Tb11.01.5920 | hypothetical protein, conserved | 0 |
| Tb11.01.6570 | hypothetical protein, conserved | 1 |
| Tb11.01.6710 | hypothetical protein, conserved | 0 |
| Tb11.01.6760 | hypothetical protein, conserved | 6 |
| Tb11.01.6860 | hypothetical protein, conserved | 1 |
| Tb11.01.7180 | hypothetical protein, conserved | 5 |
| Tb11.01.7540 | hypothetical protein, conserved | 2 |
| Tb11.01.7710 | hypothetical protein, conserved | 0 |
| Tb11.01.7840 | hypothetical protein, conserved | 0 |
| Tb11.01.8070 | hypothetical protein, conserved | 1 |
| Tb11.01.8580 | hypothetical protein, conserved | 0 |
| Tb11.01.8715 | hypothetical protein, conserved | 2 |
| Tb11.02.0060 | hypothetical protein, conserved | 1 |
| Tb11.02.0351 | hypothetical protein, conserved | 0 |
| Tb11.02.0445 | hypothetical protein, conserved | 0 |
| Tb11.02.0650 | hypothetical protein, conserved | 0 |
| Tb11.02.0670 | hypothetical protein, conserved | 4 |
| Tb11.02.0710 | hypothetical protein, conserved | 2 |

| | | |
|---|---|---|
| Tb11.02.0940 | hypothetical protein, conserved | 2 |
| Tb11.02.1150 | hypothetical protein, conserved | 0 |
| Tb11.02.1550 | hypothetical protein, conserved | 0 |
| Tb11.02.1710 | hypothetical protein, conserved | 5 |
| Tb11.02.1760 | hypothetical protein, conserved | 2 |
| Tb11.02.1890 | hypothetical protein, conserved | 0 |
| Tb11.02.1910 | hypothetical protein, conserved | 0 |
| Tb11.02.2220 | hypothetical protein, conserved | 5 |
| Tb11.02.2320 | hypothetical protein, conserved | 0 |
| Tb11.02.2420 | hypothetical protein, conserved | 0 |
| Tb11.02.2450 | hypothetical protein, conserved | 1 |
| Tb11.02.2460 | hypothetical protein, conserved | 1 |
| Tb11.02.2720 | hypothetical protein, conserved | 10 |
| Tb11.02.2730 | hypothetical protein, conserved | 3 |
| Tb11.02.2760 | hypothetical protein, conserved | 1 |
| Tb11.02.2770 | hypothetical protein, conserved | 0 |
| Tb11.02.2840 | hypothetical protein, conserved | 0 |
| Tb11.02.3230 | hypothetical protein, conserved | 0 |
| Tb11.02.3240 | hypothetical protein, conserved | 3 |
| Tb11.02.3760 | hypothetical protein, conserved | 2 |
| Tb11.02.3930 | hypothetical protein, conserved | 0 |
| Tb11.02.4120 | hypothetical protein, conserved | 0 |
| Tb11.02.4205 | hypothetical protein, conserved | 1 |
| Tb11.02.4460 | hypothetical protein, conserved | 0 |
| Tb11.02.4600 | hypothetical protein, conserved | 0 |
| Tb11.02.4710 | hypothetical protein, conserved | 6 |
| Tb11.02.4810 | hypothetical protein, conserved | 0 |
| Tb11.02.4850 | hypothetical protein, conserved | 4 |
| Tb11.02.4900 | hypothetical protein, conserved | 1 |
| Tb11.02.5165 | hypothetical protein, conserved | 1 |
| Tb11.02.5340 | hypothetical protein, conserved | 0 |
| Tb11.02.5370 | hypothetical protein, conserved | 1 |
| Tb11.02.5480 | hypothetical protein, conserved | 4 |
| Tb11.02.5490 | hypothetical protein, conserved | 4 |

| | | |
|---|---|---|
| Tb11.03.0060 | hypothetical protein, conserved | 0 |
| Tb11.03.0240 | hypothetical protein, conserved | 0 |
| Tb11.03.0370 | hypothetical protein, conserved | 1 |
| Tb11.03.0470 | hypothetical protein, conserved | 0 |
| Tb11.03.0900 | hypothetical protein, conserved | 0 |
| Tb11.12.0004 | hypothetical protein, conserved | 0 |
| Tb11.12.0008 | hypothetical protein, conserved | 4 |
| Tb11.18.0001 | hypothetical protein, conserved | 0 |
| Tb11.18.0010 | hypothetical protein, conserved | 7 |
| Tb11.39.0005 | hypothetical protein, conserved | 6 |
| Tb11.47.0016 | hypothetical protein, conserved | 0 |
| Tb11.47.0019 | hypothetical protein, conserved | 2 |
| Tb11.50.0004 | hypothetical protein, conserved | 0 |
| Tb11.52.0016 | hypothetical protein, conserved | 0 |
| Tb11.55.0010 | hypothetical protein, conserved | 0 |
| Tb11.55.0011 | hypothetical protein, conserved | 4 |
| Tb11.55.0022 | hypothetical protein, conserved | 0 |
| Tb927.1.1040 | hypothetical protein, conserved | 0 |
| Tb927.1.1060 | hypothetical protein, conserved | 0 |
| Tb927.1.110 | hypothetical protein, conserved | 1 |
| Tb927.1.1140 | hypothetical protein, conserved | 1 |
| Tb927.1.1310 | hypothetical protein, conserved | 1 |
| Tb927.1.1540 | hypothetical protein, conserved | 0 |
| Tb927.1.1650 | hypothetical protein, conserved | 0 |
| Tb927.1.2730 | hypothetical protein, conserved | 0 |
| Tb927.1.2970 | hypothetical protein, conserved | 0 |
| Tb927.1.2985 | hypothetical protein, conserved | 2 |
| Tb927.1.3410 | hypothetical protein, conserved | 2 |
| Tb927.1.3810 | hypothetical protein, conserved | 1 |
| Tb927.1.3840 | hypothetical protein, conserved | 1 |
| Tb927.1.4160 | hypothetical protein, conserved | 1 |
| Tb927.1.4220 | hypothetical protein, conserved | 0 |
| Tb927.1.4370 | hypothetical protein, conserved | 1 |
| Tb927.1.4380 | hypothetical protein, conserved | 1 |

| | | |
|---|---|---|
| Tb927.1.4550 | hypothetical protein, conserved | 3 |
| Tb927.1.4570 | hypothetical protein, conserved | 3 |
| Tb927.1.4590 | hypothetical protein, conserved | 3 |
| Tb927.1.4620 | hypothetical protein, conserved | 3 |
| Tb927.1.790 | hypothetical protein, conserved | 0 |
| Tb927.1.840 | hypothetical protein, conserved | 2 |
| Tb927.1.860 | hypothetical protein, conserved | 1 |
| Tb927.2.1700 | hypothetical protein, conserved | 2 |
| Tb927.2.1760 | hypothetical protein, conserved | 1 |
| Tb927.2.2380 | hypothetical protein, conserved | 1 |
| Tb927.2.2500 | hypothetical protein, conserved | 2 |
| Tb927.2.2530 | hypothetical protein, conserved | 0 |
| Tb927.2.2830 | hypothetical protein, conserved | 7 |
| Tb927.2.2920 | hypothetical protein, conserved | 6 |
| Tb927.2.2950 | hypothetical protein, conserved | 0 |
| Tb927.2.3180 | hypothetical protein, conserved | 0 |
| Tb927.2.3400 | hypothetical protein, conserved | 1 |
| Tb927.2.4090 | hypothetical protein, conserved | 2 |
| Tb927.2.4150 | hypothetical protein, conserved | 1 |
| Tb927.2.4460 | hypothetical protein, conserved | 0 |
| Tb927.2.4760 | hypothetical protein, conserved | 2 |
| Tb927.2.4840 | hypothetical protein, conserved | 3 |
| Tb927.2.4920 | hypothetical protein, conserved | 2 |
| Tb927.2.4920 | hypothetical protein, conserved | 2 |
| Tb927.2.4990 | hypothetical protein, conserved | 0 |
| Tb927.2.5290 | hypothetical protein, conserved | 2 |
| Tb927.2.5300 | hypothetical protein, conserved | 2 |
| Tb927.2.5310 | hypothetical protein, conserved | 2 |
| Tb927.2.5320 | hypothetical protein, conserved | 2 |
| Tb927.2.5330 | hypothetical protein, conserved | 2 |
| Tb927.2.5340 | hypothetical protein, conserved | 2 |
| Tb927.2.5350 | hypothetical protein, conserved | 2 |
| Tb927.2.5360 | hypothetical protein, conserved | 2 |
| Tb927.2.5440 | hypothetical protein, conserved | 1 |

| | | |
|---|---|---|
| Tb927.2.5970 | hypothetical protein, conserved | 1 |
| Tb927.2.6070 | hypothetical protein, conserved | 3 |
| Tb927.3.1390 | hypothetical protein, conserved | 9 |
| Tb927.3.1860 | hypothetical protein, conserved | 0 |
| Tb927.3.1870 | hypothetical protein, conserved | 1 |
| Tb927.3.2010 | hypothetical protein, conserved | 0 |
| Tb927.3.2050 | hypothetical protein, conserved | 0 |
| Tb927.3.2120 | hypothetical protein, conserved | 1 |
| Tb927.3.2360 | hypothetical protein, conserved | 1 |
| Tb927.3.2450 | hypothetical protein, conserved | 5 |
| Tb927.3.2550 | hypothetical protein, conserved | 0 |
| Tb927.3.2560 | hypothetical protein, conserved | 0 |
| Tb927.3.2570 | hypothetical protein, conserved | 0 |
| Tb927.3.2820 | hypothetical protein, conserved | 1 |
| Tb927.3.2850 | hypothetical protein, conserved | 6 |
| Tb927.3.2870 | hypothetical protein, conserved | 1 |
| Tb927.3.3130 | hypothetical protein, conserved | 1 |
| Tb927.3.3350 | hypothetical protein, conserved | 1 |
| Tb927.3.3820 | hypothetical protein, conserved | 5 |
| Tb927.3.4030 | hypothetical protein, conserved | 16 |
| Tb927.3.4080 | hypothetical protein, conserved | 14 |
| Tb927.3.4090 | hypothetical protein, conserved | 13 |
| Tb927.3.4100 | hypothetical protein, conserved | 14 |
| Tb927.3.4200 | hypothetical protein, conserved | 4 |
| Tb927.3.4440 | hypothetical protein, conserved | 0 |
| Tb927.3.4700 | hypothetical protein, conserved | 0 |
| Tb927.3.4740 | hypothetical protein, conserved | 0 |
| Tb927.3.4780 | hypothetical protein, conserved | 0 |
| Tb927.3.4870 | hypothetical protein, conserved | 0 |
| Tb927.3.5170 | hypothetical protein, conserved | 1 |
| Tb927.3.5300 | hypothetical protein, conserved | 0 |
| Tb927.3.5390 | hypothetical protein, conserved | 0 |
| Tb927.3.5430 | hypothetical protein, conserved | 3 |
| Tb927.3.5600 | hypothetical protein, conserved | 0 |

| | | |
|---|---|---|
| Tb927.3.5690 | hypothetical protein, conserved | 0 |
| Tb927.3.5700 | hypothetical protein, conserved | 0 |
| Tb927.3.5710 | hypothetical protein, conserved | 0 |
| Tb927.3.5720 | hypothetical protein, conserved | 0 |
| Tb927.3.5730 | hypothetical protein, conserved | 0 |
| Tb927.3.5760 | hypothetical protein, conserved | 0 |
| Tb927.3.640 | hypothetical protein, conserved | 1 |
| Tb927.3.710 | hypothetical protein, conserved | 5 |
| Tb927.3.840 | hypothetical protein, conserved | 1 |
| Tb927.4.1110 | hypothetical protein, conserved | 0 |
| Tb927.4.1120 | hypothetical protein, conserved | 2 |
| Tb927.4.1160 | hypothetical protein, conserved | 0 |
| Tb927.4.1190 | hypothetical protein, conserved | 1 |
| Tb927.4.1390 | hypothetical protein, conserved | 1 |
| Tb927.4.1600 | hypothetical protein, conserved | 1 |
| Tb927.4.1960 | hypothetical protein, conserved | 0 |
| Tb927.4.1970 | hypothetical protein, conserved | 1 |
| Tb927.4.2160 | hypothetical protein, conserved | 2 |
| Tb927.4.2170 | hypothetical protein, conserved | 2 |
| Tb927.4.2320 | hypothetical protein, conserved | 2 |
| Tb927.4.2420 | hypothetical protein, conserved | 3 |
| Tb927.4.2490 | hypothetical protein, conserved | 0 |
| Tb927.4.2530 | hypothetical protein, conserved | 4 |
| Tb927.4.2980 | hypothetical protein, conserved | 0 |
| Tb927.4.3040 | hypothetical protein, conserved | 1 |
| Tb927.4.320 | hypothetical protein, conserved | 8 |
| Tb927.4.3200 | hypothetical protein, conserved | 0 |
| Tb927.4.3220 | hypothetical protein, conserved | 0 |
| Tb927.4.3240 | hypothetical protein, conserved | 0 |
| Tb927.4.3260 | hypothetical protein, conserved | 1 |
| Tb927.4.3290 | hypothetical protein, conserved | 0 |
| Tb927.4.3500 | hypothetical protein, conserved | 5 |
| Tb927.4.3520 | hypothetical protein, conserved | 4 |
| Tb927.4.3710 | hypothetical protein, conserved | 3 |

| | | |
|---|---|---|
| Tb927.4.390 | hypothetical protein, conserved | 3 |
| Tb927.4.4350 | hypothetical protein, conserved | 1 |
| Tb927.4.4370 | hypothetical protein, conserved | 0 |
| Tb927.4.4640 | hypothetical protein, conserved | 0 |
| Tb927.4.4790 | hypothetical protein, conserved | 1 |
| Tb927.4.4880 | hypothetical protein, conserved | 6 |
| Tb927.4.4920 | hypothetical protein, conserved | 1 |
| Tb927.4.5080 | hypothetical protein, conserved | 3 |
| Tb927.4.510 | hypothetical protein, conserved | 1 |
| Tb927.4.5160 | hypothetical protein, conserved | 4 |
| Tb927.4.540 | hypothetical protein, conserved | 1 |
| Tb927.4.590 | hypothetical protein, conserved | 1 |
| Tb927.4.700 | hypothetical protein, conserved | 1 |
| Tb927.4.720 | hypothetical protein, conserved | 1 |
| Tb927.4.780 | hypothetical protein, conserved | 2 |
| Tb927.4.830 | hypothetical protein, conserved | 2 |
| Tb927.4.910 | hypothetical protein, conserved | 0 |
| Tb927.5.1030 | hypothetical protein, conserved | 0 |
| Tb927.5.1160 | hypothetical protein, conserved | 1 |
| Tb927.5.150 | hypothetical protein, conserved | 0 |
| Tb927.5.1630 | hypothetical protein, conserved | 1 |
| Tb927.5.1760 | hypothetical protein, conserved | 1 |
| Tb927.5.1930 | hypothetical protein, conserved | 1 |
| Tb927.5.1990 | hypothetical protein, conserved | 1 |
| Tb927.5.2030 | hypothetical protein, conserved | 6 |
| Tb927.5.2160 | hypothetical protein, conserved | 0 |
| Tb927.5.2170 | hypothetical protein, conserved | 0 |
| Tb927.5.2200 | hypothetical protein, conserved | 0 |
| Tb927.5.2230 | hypothetical protein, conserved | 0 |
| Tb927.5.2260 | hypothetical protein, conserved | 0 |
| Tb927.5.2350 | hypothetical protein, conserved | 1 |
| Tb927.5.2580 | hypothetical protein, conserved | 2 |
| Tb927.5.2600 | hypothetical protein, conserved | 0 |
| Tb927.5.2670 | hypothetical protein, conserved | 0 |

| | | |
|---|---|---|
| Tb927.5.2750 | hypothetical protein, conserved | 1 |
| Tb927.5.3540 | hypothetical protein, conserved | 7 |
| Tb927.5.3700 | hypothetical protein, conserved | 2 |
| Tb927.5.4060 | hypothetical protein, conserved | 1 |
| Tb927.5.4080 | hypothetical protein, conserved | 0 |
| Tb927.5.4100 | hypothetical protein, conserved | 0 |
| Tb927.5.4140 | hypothetical protein, conserved | 5 |
| Tb927.5.4570 | hypothetical protein, conserved | 2 |
| Tb927.5.4580 | hypothetical protein, conserved | 2 |
| Tb927.5.830 | hypothetical protein, conserved | 0 |
| Tb927.5.920 | hypothetical protein, conserved | 1 |
| Tb927.5.960 | hypothetical protein, conserved | 0 |
| Tb927.6.1170 | hypothetical protein, conserved | 5 |
| Tb927.6.1310 | hypothetical protein, conserved | 0 |
| Tb927.6.1330 | hypothetical protein, conserved | 0 |
| Tb927.6.1350 | hypothetical protein, conserved | 0 |
| Tb927.6.1370 | hypothetical protein, conserved | 0 |
| Tb927.6.1390 | hypothetical protein, conserved | 0 |
| Tb927.6.1420 | hypothetical protein, conserved | 5 |
| Tb927.6.1440 | hypothetical protein, conserved | 0 |
| Tb927.6.1710 | hypothetical protein, conserved | 0 |
| Tb927.6.1730 | hypothetical protein, conserved | 0 |
| Tb927.6.1740 | hypothetical protein, conserved | 2 |
| Tb927.6.1850 | hypothetical protein, conserved | 1 |
| Tb927.6.1930 | hypothetical protein, conserved | 4 |
| Tb927.6.1980 | hypothetical protein, conserved | 9 |
| Tb927.6.2140 | hypothetical protein, conserved | 0 |
| Tb927.6.2260 | hypothetical protein, conserved | 2 |
| Tb927.6.2320 | hypothetical protein, conserved | 2 |
| Tb927.6.2490 | hypothetical protein, conserved | 0 |
| Tb927.6.2560 | hypothetical protein, conserved | 1 |
| Tb927.6.2730 | hypothetical protein, conserved | 0 |
| Tb927.6.2990 | hypothetical protein, conserved | 0 |
| Tb927.6.370 | hypothetical protein, conserved | 2 |

| | | |
|---|---|---|
| Tb927.6.3700 | hypothetical protein, conserved | 2 |
| Tb927.6.380 | hypothetical protein, conserved | 2 |
| Tb927.6.3940 | hypothetical protein, conserved | 3 |
| Tb927.6.3960 | hypothetical protein, conserved | 8 |
| Tb927.6.4130 | hypothetical protein, conserved | 0 |
| Tb927.6.4240 | hypothetical protein, conserved | 0 |
| Tb927.6.4250 | hypothetical protein, conserved | 0 |
| Tb927.6.4260 | hypothetical protein, conserved | 1 |
| Tb927.6.4320 | hypothetical protein, conserved | 1 |
| Tb927.6.4500 | hypothetical protein, conserved | 1 |
| Tb927.6.4620 | hypothetical protein, conserved | 3 |
| Tb927.6.4620 | hypothetical protein, conserved | 3 |
| Tb927.6.4940 | hypothetical protein, conserved | 0 |
| Tb927.6.4940 | hypothetical protein, conserved | 0 |
| Tb927.6.650 | hypothetical protein, conserved | 1 |
| Tb927.6.660 | hypothetical protein, conserved | 0 |
| Tb927.6.880 | hypothetical protein, conserved | 1 |
| Tb927.7.1080 | hypothetical protein, conserved | 0 |
| Tb927.7.1150 | hypothetical protein, conserved | 0 |
| Tb927.7.1250 | hypothetical protein, conserved | 0 |
| Tb927.7.1270 | hypothetical protein, conserved | 0 |
| Tb927.7.1290 | hypothetical protein, conserved | 1 |
| Tb927.7.1480 | hypothetical protein, conserved | 1 |
| Tb927.7.1700 | hypothetical protein, conserved | 2 |
| Tb927.7.2190 | hypothetical protein, conserved | 2 |
| Tb927.7.2690 | hypothetical protein, conserved | 0 |
| Tb927.7.2950 | hypothetical protein, conserved | 2 |
| Tb927.7.3010 | hypothetical protein, conserved | 0 |
| Tb927.7.3240 | hypothetical protein, conserved | 0 |
| Tb927.7.3270 | hypothetical protein, conserved | 0 |
| Tb927.7.340 | hypothetical protein, conserved | 0 |
| Tb927.7.360 | hypothetical protein, conserved | 0 |
| Tb927.7.3600 | hypothetical protein, conserved | 1 |
| Tb927.7.3750 | hypothetical protein, conserved | 0 |

| | | |
|---|---|---|
| Tb927.7.380 | hypothetical protein, conserved | 0 |
| Tb927.7.3870 | hypothetical protein, conserved | 1 |
| Tb927.7.3890 | hypothetical protein, conserved | 0 |
| Tb927.7.3930 | hypothetical protein, conserved | 0 |
| Tb927.7.400 | hypothetical protein, conserved | 0 |
| Tb927.7.420 | hypothetical protein, conserved | 0 |
| Tb927.7.4230 | hypothetical protein, conserved | 1 |
| Tb927.7.4260 | hypothetical protein, conserved | 1 |
| Tb927.7.4270 | hypothetical protein, conserved | 1 |
| Tb927.7.4280 | hypothetical protein, conserved | 2 |
| Tb927.7.4310 | hypothetical protein, conserved | 0 |
| Tb927.7.440 | hypothetical protein, conserved | 0 |
| Tb927.7.4660 | hypothetical protein, conserved | 1 |
| Tb927.7.4820 | hypothetical protein, conserved | 0 |
| Tb927.7.4880 | hypothetical protein, conserved | 1 |
| Tb927.7.4890 | hypothetical protein, conserved | 1 |
| Tb927.7.5400 | hypothetical protein, conserved | 2 |
| Tb927.7.5420 | hypothetical protein, conserved | 2 |
| Tb927.7.5470 | hypothetical protein, conserved | 1 |
| Tb927.7.5700 | hypothetical protein, conserved | 1 |
| Tb927.7.5710 | hypothetical protein, conserved | 2 |
| Tb927.7.5780 | hypothetical protein, conserved | 1 |
| Tb927.7.590 | hypothetical protein, conserved | 0 |
| Tb927.7.6110 | hypothetical protein, conserved | 6 |
| Tb927.7.6130 | hypothetical protein, conserved | 5 |
| Tb927.7.6170 | hypothetical protein, conserved | 4 |
| Tb927.7.6370 | hypothetical protein, conserved | 0 |
| Tb927.7.6420 | hypothetical protein, conserved | 0 |
| Tb927.7.6490 | hypothetical protein, conserved | 0 |
| Tb927.7.6550 | hypothetical protein, conserved | 1 |
| Tb927.7.6560 | hypothetical protein, conserved | 0 |
| Tb927.7.6570 | hypothetical protein, conserved | 1 |
| Tb927.7.6590 | hypothetical protein, conserved | 1 |
| Tb927.7.6600 | hypothetical protein, conserved | 0 |

| | | |
|---|---|---|
| Tb927.7.6760 | hypothetical protein, conserved | 2 |
| Tb927.7.6920 | hypothetical protein, conserved | 1 |
| Tb927.7.900 | hypothetical protein, conserved | 1 |
| Tb927.7.950 | hypothetical protein, conserved | 7 |
| Tb927.8.1250 | hypothetical protein, conserved | 0 |
| Tb927.8.1370 | hypothetical protein, conserved | 2 |
| Tb927.8.1530 | hypothetical protein, conserved | 8 |
| Tb927.8.1580 | hypothetical protein, conserved | 1 |
| Tb927.8.1700 | hypothetical protein, conserved | 0 |
| Tb927.8.1740 | hypothetical protein, conserved | 0 |
| Tb927.8.2230 | hypothetical protein, conserved | 1 |
| Tb927.8.2390 | hypothetical protein, conserved | 0 |
| Tb927.8.2460 | hypothetical protein, conserved | 5 |
| Tb927.8.2510 | hypothetical protein, conserved | 1 |
| Tb927.8.2570 | hypothetical protein, conserved | 2 |
| Tb927.8.2680 | hypothetical protein, conserved | 0 |
| Tb927.8.2690 | hypothetical protein, conserved | 0 |
| Tb927.8.3040 | hypothetical protein, conserved | 0 |
| Tb927.8.3050 | hypothetical protein, conserved | 1 |
| Tb927.8.3320 | hypothetical protein, conserved | 2 |
| Tb927.8.3360 | hypothetical protein, conserved | 3 |
| Tb927.8.3390 | hypothetical protein, conserved | 2 |
| Tb927.8.3400 | hypothetical protein, conserved | 4 |
| Tb927.8.3540 | hypothetical protein, conserved | 1 |
| Tb927.8.3720 | hypothetical protein, conserved | 2 |
| Tb927.8.3730 | hypothetical protein, conserved | 4 |
| Tb927.8.3880 | hypothetical protein, conserved | 2 |
| Tb927.8.3890 | hypothetical protein, conserved | 2 |
| Tb927.8.3900 | hypothetical protein, conserved | 2 |
| Tb927.8.3960 | hypothetical protein, conserved | 0 |
| Tb927.8.4000 | hypothetical protein, conserved | 2 |
| Tb927.8.4150 | hypothetical protein, conserved | 4 |
| Tb927.8.4230 | hypothetical protein, conserved | 0 |
| Tb927.8.4240 | hypothetical protein, conserved | 0 |

| | | |
|---|---|---|
| Tb927.8.4340 | hypothetical protein, conserved | 4 |
| Tb927.8.4520 | hypothetical protein, conserved | 4 |
| Tb927.8.4600 | hypothetical protein, conserved | 2 |
| Tb927.8.4630 | hypothetical protein, conserved | 3 |
| Tb927.8.4760 | hypothetical protein, conserved | 0 |
| Tb927.8.5080 | hypothetical protein, conserved | 1 |
| Tb927.8.5150 | hypothetical protein, conserved | 1 |
| Tb927.8.5360 | hypothetical protein, conserved | 0 |
| Tb927.8.5420 | hypothetical protein, conserved | 1 |
| Tb927.8.5540 | hypothetical protein, conserved | 0 |
| Tb927.8.560 | hypothetical protein, conserved | 1 |
| Tb927.8.580 | hypothetical protein, conserved | 1 |
| Tb927.8.6020 | hypothetical protein, conserved | 1 |
| Tb927.8.6080 | hypothetical protein, conserved | 0 |
| Tb927.8.6310 | hypothetical protein, conserved | 1 |
| Tb927.8.6380 | hypothetical protein, conserved | 1 |
| Tb927.8.6480 | hypothetical protein, conserved | 0 |
| Tb927.8.6550 | hypothetical protein, conserved | 0 |
| Tb927.8.6570 | hypothetical protein, conserved | 4 |
| Tb927.8.6590 | hypothetical protein, conserved | 1 |
| Tb927.8.6620 | hypothetical protein, conserved | 3 |
| Tb927.8.6700 | hypothetical protein, conserved | 6 |
| Tb927.8.6710 | hypothetical protein, conserved | 6 |
| Tb927.8.6720 | hypothetical protein, conserved | 6 |
| Tb927.8.6730 | hypothetical protein, conserved | 6 |
| Tb927.8.6800 | hypothetical protein, conserved | 2 |
| Tb927.8.6960 | hypothetical protein, conserved | 1 |
| Tb927.8.7230 | hypothetical protein, conserved | 4 |
| Tb927.8.7280 | hypothetical protein, conserved | 1 |
| Tb927.8.7500 | hypothetical protein, conserved | 1 |
| Tb927.8.7580 | hypothetical protein, conserved | 7 |
| Tb927.8.7710 | hypothetical protein, conserved | 1 |
| Tb927.8.7720 | hypothetical protein, conserved | 1 |
| Tb927.8.8030 | hypothetical protein, conserved | 1 |

| | | |
|---|---|---|
| Tb927.8.8120 | hypothetical protein, conserved | 0 |
| Tb927.8.8170 | hypothetical protein, conserved | 0 |
| Tb927.4.3270 | hypothetical protein, conserved,ESAG11-related protein, putative | 0 |
| Tb10.05.0040 | hypothetical protein, conserved,hypothetical protein | 1 |
| Tb10.05.0100 | hypothetical protein, conserved,hypothetical protein | 5 |
| Tb11.46.0011 | hypothetical protein, conserved,leucine-rich repeat protein (LRRP), putative | 0 |
| Tb10.70.0930 | hypothetical protein, conserved,predicted tripartite motif protein,predicted zinc finger protein | 0 |
| Tb10.6k15.3270 | hypothetical protein, conserved,predicted zinc finger protein | 6 |
| Tb927.8.5570 | hypothetical protein, conserved,transporter, putative | 10 |
| Tb09.211.0210 | hypothetical protein, conserved,tyrosine phosphatase, putative | 1 |
| Tb11.01.4490 | hypothetical protein, conserved,Zinc finger DHHC domain containing transmembrane protein, putative | 6 |
| Tb09.160.0090 | hypothetical protein, unlikely | 4 |
| Tb09.160.0300 | hypothetical protein, unlikely | 2 |
| Tb09.160.0310 | hypothetical protein, unlikely | 2 |
| Tb09.160.0330 | hypothetical protein, unlikely | 0 |
| Tb09.160.0590 | hypothetical protein, unlikely | 3 |
| Tb09.160.0980 | hypothetical protein, unlikely | 2 |
| Tb09.160.1050 | hypothetical protein, unlikely | 1 |
| Tb09.160.1230 | hypothetical protein, unlikely | 2 |
| Tb09.160.1430 | hypothetical protein, unlikely | 2 |
| Tb09.160.1510 | hypothetical protein, unlikely | 2 |
| Tb09.160.1600 | hypothetical protein, unlikely | 0 |
| Tb09.160.1640 | hypothetical protein, unlikely | 1 |
| Tb09.160.1750 | hypothetical protein, unlikely | 2 |
| Tb09.160.1800 | hypothetical protein, unlikely | 1 |
| Tb09.160.1850 | hypothetical protein, unlikely | 0 |
| Tb09.160.2140 | hypothetical protein, unlikely | 1 |
| Tb09.160.2190 | hypothetical protein, unlikely | 1 |
| Tb09.160.2270 | hypothetical protein, unlikely | 1 |
| Tb09.160.2340 | hypothetical protein, unlikely | 1 |
| Tb09.160.2680 | hypothetical protein, unlikely | 1 |
| Tb09.160.2720 | hypothetical protein, unlikely | 0 |
| Tb09.160.2730 | hypothetical protein, unlikely | 5 |
| Tb09.160.2740 | hypothetical protein, unlikely | 0 |

| | | |
|---|---|---|
| Tb09.160.2750 | hypothetical protein, unlikely | 1 |
| Tb09.160.3100 | hypothetical protein, unlikely | 0 |
| Tb09.160.3170 | hypothetical protein, unlikely | 2 |
| Tb09.160.3180 | hypothetical protein, unlikely | 2 |
| Tb09.160.3340 | hypothetical protein, unlikely | 1 |
| Tb09.160.3600 | hypothetical protein, unlikely | 2 |
| Tb09.160.3610 | hypothetical protein, unlikely | 2 |
| Tb09.160.3620 | hypothetical protein, unlikely | 0 |
| Tb09.160.3680 | hypothetical protein, unlikely | 1 |
| Tb09.160.3700 | hypothetical protein, unlikely | 2 |
| Tb09.160.3770 | hypothetical protein, unlikely | 2 |
| Tb09.160.3810 | hypothetical protein, unlikely | 1 |
| Tb09.160.3870 | hypothetical protein, unlikely | 1 |
| Tb09.160.4060 | hypothetical protein, unlikely | 3 |
| Tb09.160.4070 | hypothetical protein, unlikely | 1 |
| Tb09.160.4100 | hypothetical protein, unlikely | 2 |
| Tb09.160.4110 | hypothetical protein, unlikely | 0 |
| Tb09.160.4230 | hypothetical protein, unlikely | 1 |
| Tb09.160.4370 | hypothetical protein, unlikely | 1 |
| Tb09.160.4390 | hypothetical protein, unlikely | 1 |
| Tb09.160.4510 | hypothetical protein, unlikely | 0 |
| Tb09.160.4740 | hypothetical protein, unlikely | 3 |
| Tb09.160.4810 | hypothetical protein, unlikely | 1 |
| Tb09.160.4880 | hypothetical protein, unlikely | 0 |
| Tb09.160.4980 | hypothetical protein, unlikely | 0 |
| Tb09.160.4990 | hypothetical protein, unlikely | 1 |
| Tb09.160.5090 | hypothetical protein, unlikely | 0 |
| Tb09.160.5140 | hypothetical protein, unlikely | 2 |
| Tb09.160.5210 | hypothetical protein, unlikely | 3 |
| Tb09.160.5290 | hypothetical protein, unlikely | 3 |
| Tb09.160.5340 | hypothetical protein, unlikely | 0 |
| Tb09.160.5450 | hypothetical protein, unlikely | 0 |
| Tb09.160.5500 | hypothetical protein, unlikely | 4 |
| Tb09.160.5520 | hypothetical protein, unlikely | 3 |

| | | |
|---|---|---|
| Tb09.160.5540 | hypothetical protein, unlikely | 4 |
| Tb09.160.5610 | hypothetical protein, unlikely | 1 |
| Tb09.160.5630 | hypothetical protein, unlikely | 1 |
| Tb09.211.0240 | hypothetical protein, unlikely | 3 |
| Tb09.211.0360 | hypothetical protein, unlikely | 0 |
| Tb09.211.0490 | hypothetical protein, unlikely | 0 |
| Tb09.211.0550 | hypothetical protein, unlikely | 1 |
| Tb09.211.0570 | hypothetical protein, unlikely | 0 |
| Tb09.211.0640 | hypothetical protein, unlikely | 0 |
| Tb09.211.0650 | hypothetical protein, unlikely | 0 |
| Tb09.211.0660 | hypothetical protein, unlikely | 0 |
| Tb09.211.0670 | hypothetical protein, unlikely | 0 |
| Tb09.211.0750 | hypothetical protein, unlikely | 3 |
| Tb09.211.1060 | hypothetical protein, unlikely | 2 |
| Tb09.211.1100 | hypothetical protein, unlikely | 1 |
| Tb09.211.1120 | hypothetical protein, unlikely | 2 |
| Tb09.211.1340 | hypothetical protein, unlikely | 0 |
| Tb09.211.1420 | hypothetical protein, unlikely | 0 |
| Tb09.211.1450 | hypothetical protein, unlikely | 1 |
| Tb09.211.1460 | hypothetical protein, unlikely | 1 |
| Tb09.211.1730 | hypothetical protein, unlikely | 1 |
| Tb09.211.1840 | hypothetical protein, unlikely | 2 |
| Tb09.211.1860 | hypothetical protein, unlikely | 2 |
| Tb09.211.1920 | hypothetical protein, unlikely | 2 |
| Tb09.211.1990 | hypothetical protein, unlikely | 1 |
| Tb09.211.2000 | hypothetical protein, unlikely | 0 |
| Tb09.211.2170 | hypothetical protein, unlikely | 0 |
| Tb09.211.2280 | hypothetical protein, unlikely | 4 |
| Tb09.211.2390 | hypothetical protein, unlikely | 0 |
| Tb09.211.2480 | hypothetical protein, unlikely | 0 |
| Tb09.211.2820 | hypothetical protein, unlikely | 0 |
| Tb09.211.2840 | hypothetical protein, unlikely | 1 |
| Tb09.211.2980 | hypothetical protein, unlikely | 3 |
| Tb09.211.3710 | hypothetical protein, unlikely | 1 |

| | | |
|---|---|---|
| Tb09.211.4050 | hypothetical protein, unlikely | 1 |
| Tb09.211.4250 | hypothetical protein, unlikely | 1 |
| Tb09.211.4340 | hypothetical protein, unlikely | 2 |
| Tb09.211.4410 | hypothetical protein, unlikely | 2 |
| Tb09.211.4620 | hypothetical protein, unlikely | 4 |
| Tb09.211.4660 | hypothetical protein, unlikely | 1 |
| Tb09.211.4750 | hypothetical protein, unlikely | 3 |
| Tb09.211.4790 | hypothetical protein, unlikely | 0 |
| Tb09.244.0510 | hypothetical protein, unlikely | 1 |
| Tb09.244.1270 | hypothetical protein, unlikely | 0 |
| Tb09.244.2040 | hypothetical protein, unlikely | 0 |
| Tb09.244.2110 | hypothetical protein, unlikely | 1 |
| Tb09.244.2360 | hypothetical protein, unlikely | 0 |
| Tb09.v1.0090 | hypothetical protein, unlikely | 0 |
| Tb09.v1.0100 | hypothetical protein, unlikely | 1 |
| Tb09.v1.0110 | hypothetical protein, unlikely | 0 |
| Tb09.v1.0210 | hypothetical protein, unlikely | 1 |
| Tb09.v1.0340 | hypothetical protein, unlikely | 1 |
| Tb09.v1.0430 | hypothetical protein, unlikely | 0 |
| Tb09.v1.0570 | hypothetical protein, unlikely | 1 |
| Tb09.v1.0750 | hypothetical protein, unlikely | 3 |
| Tb09.v1.0760 | hypothetical protein, unlikely | 1 |
| Tb09.v1.0840 | hypothetical protein, unlikely | 3 |
| Tb09.v1.0850 | hypothetical protein, unlikely | 2 |
| Tb09.v1.0860 | hypothetical protein, unlikely | 0 |
| Tb09.v1.0880 | hypothetical protein, unlikely | 3 |
| Tb09.v2.0050 | hypothetical protein, unlikely | 1 |
| Tb10.61.0710 | hypothetical protein, unlikely | 2 |
| Tb10.61.0790 | hypothetical protein, unlikely | 1 |
| Tb10.6k15.0230 | hypothetical protein, unlikely | 0 |
| Tb11.02.1960 | hypothetical protein, unlikely | 1 |
| Tb11.02.2990 | hypothetical protein, unlikely | 1 |
| Tb927.1.1110 | hypothetical protein, unlikely | 0 |
| Tb927.1.1180 | hypothetical protein, unlikely | 0 |

| | | |
|---|---|---|
| Tb927.1.1250 | hypothetical protein, unlikely | 0 |
| Tb927.1.1280 | hypothetical protein, unlikely | 2 |
| Tb927.1.1320 | hypothetical protein, unlikely | 1 |
| Tb927.1.1360 | hypothetical protein, unlikely | 2 |
| Tb927.1.1430 | hypothetical protein, unlikely | 3 |
| Tb927.1.1460 | hypothetical protein, unlikely | 1 |
| Tb927.1.1570 | hypothetical protein, unlikely | 0 |
| Tb927.1.1590 | hypothetical protein, unlikely | 1 |
| Tb927.1.1780 | hypothetical protein, unlikely | 5 |
| Tb927.1.1870 | hypothetical protein, unlikely | 0 |
| Tb927.1.2140 | hypothetical protein, unlikely | 3 |
| Tb927.1.2170 | hypothetical protein, unlikely | 4 |
| Tb927.1.2280 | hypothetical protein, unlikely | 3 |
| Tb927.1.2300 | hypothetical protein, unlikely | 3 |
| Tb927.1.2440 | hypothetical protein, unlikely | 0 |
| Tb927.1.2460 | hypothetical protein, unlikely | 0 |
| Tb927.1.2480 | hypothetical protein, unlikely | 0 |
| Tb927.1.2500 | hypothetical protein, unlikely | 0 |
| Tb927.1.2520 | hypothetical protein, unlikely | 0 |
| Tb927.1.2560 | hypothetical protein, unlikely | 0 |
| Tb927.1.2590 | hypothetical protein, unlikely | 2 |
| Tb927.1.2610 | hypothetical protein, unlikely | 1 |
| Tb927.1.2660 | hypothetical protein, unlikely | 1 |
| Tb927.1.2900 | hypothetical protein, unlikely | 0 |
| Tb927.1.2940 | hypothetical protein, unlikely | 0 |
| Tb927.1.3370 | hypothetical protein, unlikely | 0 |
| Tb927.1.3380 | hypothetical protein, unlikely | 0 |
| Tb927.1.3420 | hypothetical protein, unlikely | 1 |
| Tb927.1.3440 | hypothetical protein, unlikely | 2 |
| Tb927.1.3480 | hypothetical protein, unlikely | 2 |
| Tb927.1.3490 | hypothetical protein, unlikely | 0 |
| Tb927.1.3640 | hypothetical protein, unlikely | 0 |
| Tb927.1.3700 | hypothetical protein, unlikely | 3 |
| Tb927.1.3720 | hypothetical protein, unlikely | 0 |

| | | |
|---|---|---|
| Tb927.1.3900 | hypothetical protein, unlikely | 1 |
| Tb927.1.3930 | hypothetical protein, unlikely | 1 |
| Tb927.1.4000 | hypothetical protein, unlikely | 3 |
| Tb927.1.4030 | hypothetical protein, unlikely | 3 |
| Tb927.1.4070 | hypothetical protein, unlikely | 1 |
| Tb927.1.4130 | hypothetical protein, unlikely | 3 |
| Tb927.1.4150 | hypothetical protein, unlikely | 3 |
| Tb927.1.4290 | hypothetical protein, unlikely | 2 |
| Tb927.1.4350 | hypothetical protein, unlikely | 3 |
| Tb927.1.4430 | hypothetical protein, unlikely | 1 |
| Tb927.1.4530 | hypothetical protein, unlikely | 1 |
| Tb927.1.4610 | hypothetical protein, unlikely | 1 |
| Tb927.1.4640 | hypothetical protein, unlikely | 1 |
| Tb927.1.4670 | hypothetical protein, unlikely | 2 |
| Tb927.1.4770 | hypothetical protein, unlikely | 1 |
| Tb927.1.4790 | hypothetical protein, unlikely | 3 |
| Tb927.1.4820 | hypothetical protein, unlikely | 0 |
| Tb927.1.50 | hypothetical protein, unlikely | 0 |
| Tb927.1.5010 | hypothetical protein, unlikely | 0 |
| Tb927.1.5040 | hypothetical protein, unlikely | 1 |
| Tb927.1.5130 | hypothetical protein, unlikely | 1 |
| Tb927.1.5310 | hypothetical protein, unlikely | 2 |
| Tb927.1.660 | hypothetical protein, unlikely | 1 |
| Tb927.1.770 | hypothetical protein, unlikely | 4 |

**SignalP Prediction on Trypanosome proteome classified according TMHMM**

| Accession number | Protein name | TMHMM prediction |
|---|---|---|
| Tb927.7.6850 | TbTS trans-sialidase | 0 |
| Tb11.01.3240 | trans-sialidase, putative | 0 |
| Tb927.2.5280 | trans-sialidase, putative | 0 |
| Tb927.7.6830 | trans-sialidase, putative | 0 |
| Tb927.8.7340 | trans-sialidase, putative,neuraminidase, putative | 0 |
| Tb927.8.7350 | trans-sialidase, putative,neuraminidase, putative | 0 |
| Tb927.3.2840 | inorganic pyrophosphatase, putative | 0 |
| Tb927.7.940 | protein kinase C substrate protein, heavy chain, putative,glucosidase II beta subunit, putative | 0 |
| Tb927.5.3160 | protein kinase, putative | 0 |
| Tb927.4.3680 | protein phosphatase 2C, putative | 0 |
| Tb927.6.750 | serine/threonine protein phosphatase, putative | 0 |
| Tb10.70.2260 | PK50 serine/threonine protein kinase | 0 |
| Tb927.5.4500 | ADP-ribosylation factor, putative | 0 |
| Tb11.02.4040 | protein transport protein Sec31, putative,cytosolic coat protein, putative | 0 |
| Tb927.6.470 | gene related to expression site-associated gene 2 (GRESAG2), putative | 0 |
| Tb927.6.470 | gene related to expression site-associated gene 2 (GRESAG2), putative | 0 |
| Tb927.6.500 | gene related to expression site-associated gene 2 (GRESAG2), putative | 0 |
| Tb927.6.540 | gene related to expression site-associated gene 2 (GRESAG2), putative | 0 |
| Tb927.7.5160 | deoxyuridine triphosphatase, putative,dUTP diphosphatase | 0 |
| Tb09.160.2000 | pseudouridylate synthase, putative | 0 |
| Tb927.7.1640 | GTP-binding protein, putative | 0 |
| Tb10.70.4600 | GTP-binding protein, putative,elongation factor, putative | 0 |
| Tb10.389.1430 | helicase-like protein | 0 |
| Tb11.01.1100 | helicase-like protein | 0 |
| Tb10.61.2130 | ATP-dependent DEAD/H RNA helicase, putative | 0 |
| Tb927.6.1640 | single strand-specific nuclease, putative | 0 |
| Tb927.6.1650 | single strand-specific nuclease, putative | 0 |
| Tb927.8.2810 | XRNC 5'-3' exonuclease XRNC, putative,exoribonuclease 2, putative | 0 |

| | | |
|---|---|---|
| Tb10.70.0610 | XRND 5'-3' exoribonuclease XRND, putative,5'-3' exoribonuclease 2, putative | 0 |
| Tb10.70.1510 | endonuclease/exonuclease/phosphatase, putative | 0 |
| Tb10.70.0780 | tRNA pseudouridine synthase A, putative | 0 |
| Tb10.61.0690 | tRNA pseudouridine synthase A-like protein | 0 |
| Tb09.244.2400 | BARP BARP protein | 0 |
| Tb09.244.2410 | BARP BARP protein | 0 |
| Tb09.244.2420 | BARP BARP protein | 0 |
| Tb09.244.2430 | BARP BARP protein | 0 |
| Tb09.244.2440 | BARP BARP protein | 0 |
| Tb09.244.2450 | BARP BARP protein | 0 |
| Tb09.244.2460 | BARP BARP protein | 0 |
| Tb09.244.2470 | BARP BARP protein | 0 |
| Tb09.244.2480 | BARP BARP protein | 0 |
| Tb09.244.2490 | BARP BARP protein | 0 |
| Tb09.244.2500 | BARP BARP protein | 0 |
| Tb09.244.2510 | BARP BARP protein | 0 |
| Tb09.244.2520 | BARP BARP protein | 0 |
| Tb09.244.2530 | BARP BARP protein | 0 |
| Tb09.211.3680 | chaperone protein DNAJ, putative | 0 |
| Tb11.01.8480 | chaperone protein DNAJ, putative | 0 |
| Tb927.3.1430 | chaperone protein DnaJ, putative | 0 |
| Tb927.3.1760 | chaperone protein DNAJ, putative | 0 |
| Tb927.7.680 | chaperone protein DNAJ, putative | 0 |
| Tb927.8.7010 | chaperone protein DNAJ, putative | 0 |
| Tb927.7.3630 | TPR-repeat-containing chaperone protein DNAJ, putative | 0 |
| Tb927.6.2170 | co-chaperone GrpE, putative | 0 |
| Tb11.01.4050 | heat shock protein HslVU, ATPase subunit HslU, putative,ATP-dependent hsl protease ATP-binding subunit hslU, putative | 0 |
| Tb927.2.1560 | cyclophilin type peptidyl-prolyl cis-trans isomerase precursor, putative | 0 |
| Tb10.61.0760 | FKBP-type peptidyl-prolyl cis-trans isomerase, conserved,FKBP-type peptidylprolyl cis-trans isomerase, putative | 0 |
| Tb927.7.280 | PPIase cyclophilin-type peptidyl-prolyl cis-trans isomerase, putative | 0 |
| Tb927.5.1370 | PPIase cyclophilin-type peptidyl-prolyl cis-trans isomerase, putative,PPIase, putative,rotamase, putative, | 0 |
| Tb927.7.1300 | protein disulfide isomerase, putative | 0 |
| Tb927.7.5790 | protein disulfide isomerase, putative | 0 |
| Tb10.6k15.2290 | protein disulfide isomerase,bloodstream-specific protein 2 precursor | 0 |

| | | |
|---|---|---|
| Tb10.70.7090 | serine carboxypeptidase III precursor, putative,serine carboxypeptidase (CBP1) precursor, putative,Clan SC, Family S10 | 0 |
| Tb10.70.7100 | CBP1 serine carboxypeptidase III precursor, putative,serine peptidase, Clan SC, Family S10 | 0 |
| Tb10.389.0470 | calpain-like cysteine peptidase, putative,cysteine peptidase, Clan CA, family C2, putative | 0 |
| Tb11.01.5800 | calpain-like cysteine peptidase, putative,cysteine peptidase, Clan CA, family C2, putative | 0 |
| Tb11.02.5610 | Gp63-1 surface protease homolog, putative | 0 |
| Tb11.02.5630 | Gp63-1 surface protease homolog, putative | 0 |
| Tb11.02.5640 | Gp63-1 surface protease homolog, putative | 0 |
| Tb11.02.5310 | major surface protease A, putative,Gp63 major surface glycoprotein-like protein,MSP-A, putative | 0 |
| Tb10.70.5290 | MSP-C major surface protease gp63, putative,surface protease homologue | 0 |
| Tb927.6.560 | TbcatB cysteine peptidase C (CPC),CPC cysteine peptidase, Clan CA, family C1, Cathepsin B-like | 0 |
| Tb09.211.4760 | metacaspase 5, putative | 0 |
| Tb09.160.3110 | mitochondrial processing peptide beta subunit, putative,metallo-peptidase, Clan ME, Family M16 | 0 |
| Tb927.7.3500 | glutathione-S-transferase/glutaredoxin,putative | 0 |
| Tb927.4.1350 | glx2-2; glx II hydroxyacylglutathione hydrolase, putative,glyoxalase II | 0 |
| Tb11.01.7480 | superoxide dismutase, putative | 0 |
| Tb10.6k15.3080 | dihydrolipoamide acetyltransferase precursor, putative | 0 |
| Tb10.70.5380 | dihydrolipoamide acetyltransferase, putative | 0 |
| Tb09.160.4300 | farnesyl pyrophosphate synthetase, putative | 0 |
| Tb09.160.0390 | monooxygenase, putative | 0 |
| Tb927.5.490 | methyltransferase, putative | 0 |
| Tb11.02.4080 | LDM; P450LI lanosterol 14-alpha-demethylase,cytochrome P450 51A1 | 0 |
| Tb10.61.1530 | lipoic acid synthetase, mitochondrial precursor, putative | 0 |
| Tb927.4.1140 | phosphatidylinositol 4-kinase, putative | 0 |
| Tb11.01.2110 | phosphoglycerate mutase-like protein,phosphoglycerate mutase protein, putative | 0 |
| Tb10.61.3060 | GPI-anchor transamidase subunit 8 (GPI8),cysteine peptidase, Clan CD, family C13 | 0 |
| Tb10.70.2420 | GPIdeAc GPI inositol deacylase precursor | 0 |
| Tb927.6.4280 | GAPDH glyceraldehyde 3-phosphate dehydrogenase, glycosomal | 0 |
| Tb927.6.4300 | GAPDH glyceraldehyde 3-phosphate dehydrogenase, glycosomal | 0 |
| Tb11.01.5560 | GDP-L-fucose synthetase, putative | 0 |
| Tb11.02.5450 | glucose-regulated protein 78, putative,luminal binding protein 1 (BiP), putative | 0 |
| Tb11.02.5500 | glucose-regulated protein 78, putative,luminal binding protein 1 (BiP), putative | 0 |
| Tb10.05.0080 | glucosidase, putative | 0 |
| Tb927.8.2910 | mannosyl-oligosaccharide 1,2-alpha-mannosidase IB, putative | 0 |
| Tb927.8.2920 | mannosyl-oligosaccharide 1,2-alpha-mannosidase IB, putative | 0 |

| | | |
|---|---|---|
| Tb927.8.2930 | mannosyl-oligosaccharide 1,2-alpha-mannosidase IB, putative | 0 |
| Tb927.8.2940 | mannosyl-oligosaccharide 1,2-alpha-mannosidase IB, putative | 0 |
| Tb09.211.0860 | glycosyl transferase, putative | 0 |
| Tb09.142.0450 | UDP-Gal or UDP-GlcNAc-dependent glycosyltransferase, putative | 0 |
| Tb927.7.300 | UDP-Gal or UDP-GlcNAc-dependent glycosyltransferase, putative | 0 |
| Tb11.01.7190 | B5R NADH-cytochrome b5 reductase, putative | 0 |
| Tb927.5.1470 | B5R NADH-cytochrome b5 reductase, putative | 0 |
| Tb11.55.0013 | cysteine desulfurase, putative | 0 |
| Tb11.01.0530 | DNA repair protein, putative | 0 |
| Tb11.02.5530 | electron-transfer-flavoprotein, alpha polypeptide, putative | 0 |
| Tb10.6k15.0040 | PAG1 procyclin-associated gene 1 (PAG1) protein | 0 |
| Tb10.6k15.0060 | PAG2 procyclin-associated gene 2 (PAG2) protein | 0 |
| Tb10.6k15.0070 | PAG4 procyclin-associated gene 4 (PAG4) protein | 0 |
| Tb10.6k15.0050 | PAG5 procyclin-associated gene 5 (PAG5) protein | 0 |
| Tb10.70.1310 | procyclin-associated gene 1 (PAG1) protein, putative | 0 |
| Tb10.70.1300 | procyclin-associated gene 2 (PAG2) protein, putative | 0 |
| Tb11.01.6220 | procyclin-associated gene 4 (PAG4) protein, putative | 0 |
| Tb10.v4.0002 | expression site-associated gene (ESAG) protein, putative,expression site-associated gene 1 (ESAG1) protein, putative | 0 |
| Tb11.17.0002 | expression site-associated gene (ESAG) protein, putative,expression site-associated gene 1 (ESAG1) protein, putative | 0 |
| Tb927.1.4870 | expression site-associated gene (ESAG) protein, putative,expression site-associated gene 1 (ESAG1) protein, putative | 0 |
| Tb927.1.4910 | expression site-associated gene (ESAG) protein, putative,expression site-associated gene 1 (ESAG1) protein, putative | 0 |
| Tb927.1.5120 | expression site-associated gene (ESAG) protein, putative,expression site-associated gene 1 (ESAG1) protein, putative | 0 |
| Tb927.1.5200 | expression site-associated gene (ESAG) protein, putative,expression site-associated gene 1 (ESAG1) protein, putative | 0 |
| Tb927.3.2520 | expression site-associated gene (ESAG) protein, putative,expression site-associated gene 1 (ESAG1) protein, putative | 0 |
| Tb927.3.520 | expression site-associated gene (ESAG) protein, putative,expression site-associated gene 1 (ESAG1) protein, putative | 0 |
| Tb927.3.5830 | expression site-associated gene (ESAG) protein, putative,expression site-associated gene 1 (ESAG1) protein, putative | 0 |
| Tb927.4.1200 | expression site-associated gene (ESAG) protein, putative,expression site-associated gene 1 (ESAG1) protein, putative | 0 |
| Tb927.5.4630 | expression site-associated gene (ESAG) protein, putative,expression site-associated gene 1 (ESAG1) protein, putative | 0 |
| Tb11.01.6250 | expression site-associated gene (ESAG) protein, putative,expression site-associated gene 11 (ESAG11) protein, putative | 0 |
| Tb927.3.560 | expression site-associated gene (ESAG) protein, putative,expression site-associated gene 11 (ESAG11) protein, putative | 0 |
| Tb11.01.6240 | expression site-associated gene (ESAG) protein, putative,expression site-associated gene 2 (ESAG2) protein, putative | 0 |
| Tb927.3.2510 | expression site-associated gene (ESAG) protein, putative,expression site-associated gene 2 (ESAG2) protein, putative | 0 |
| Tb927.3.570 | expression site-associated gene (ESAG) protein, putative,expression site-associated gene 2 (ESAG2) protein, putative | 0 |
| Tb927.4.3280 | expression site-associated gene (ESAG) protein, putative,expression site-associated gene 2 (ESAG2) protein, putative | 0 |

| | | |
|---|---|---|
| Tb927.2.1920 | expression site-associated gene (ESAG) protein, putative,expression site-associated gene 5 (ESAG5) protein, putative | 0 |
| Tb927.4.810 | expression site-associated gene (ESAG) protein, putative,expression site-associated gene 5 (ESAG5) protein, putative | 0 |
| Tb927.5.340 | expression site-associated gene (ESAG) protein, putative,expression site-associated gene 5 (ESAG5) protein, putative | 0 |
| Tb927.7.3250 | expression site-associated gene (ESAG) protein, putative,expression site-associated gene 6 (ESAG6) protein, putative | 0 |
| Tb927.7.3260 | expression site-associated gene (ESAG) protein, putative,expression site-associated gene 7 (ESAG7) protein, putative | 0 |
| Tb927.1.5080 | expression site-associated gene (ESAG) protein, putative,expression site-associated gene 9 (ESAG9) protein, putative | 0 |
| Tb927.5.120 | expression site-associated gene (ESAG) protein, putative,expression site-associated gene 9 (ESAG9) protein, putative | 0 |
| Tb927.5.4620 | expression site-associated gene (ESAG) protein, putative,expression site-associated gene 9 (ESAG9) protein, putative | 0 |
| Tb927.7.170 | expression site-associated gene (ESAG) protein, putative,expression site-associated gene 9 (ESAG9) protein, putative | 0 |
| Tb09.142.0310 | expression site-associated gene (ESAG) protein,expression site-associated gene 1 (ESAG1) protein | 0 |
| Tb927.1.5240 | expression site-associated gene 1 (ESAG1) protein, putative | 0 |
| Tb927.1.4900 | expression site-associated gene 11 (ESAG11) protein, putative | 0 |
| Tb927.1.5110 | expression site-associated gene 11 (ESAG11) protein, putative | 0 |
| Tb09.160.5390 | expression site-associated gene 2 (ESAG2) protein, putative | 0 |
| Tb09.v1.0320 | expression site-associated gene 2 (ESAG2) protein, putative | 0 |
| Tb11.01.6230 | expression site-associated gene 2 (ESAG2) protein, putative | 0 |
| Tb927.1.2040 | expression site-associated gene 2 (ESAG2) protein, putative | 0 |
| Tb927.1.4890 | expression site-associated gene 2 (ESAG2) protein, putative | 0 |
| Tb927.1.5100 | expression site-associated gene 2 (ESAG2) protein, putative | 0 |
| Tb09.160.5400 | expression site-associated gene 9 (ESAG9) protein, putative | 0 |
| Tb09.160.5430 | expression site-associated gene 9 (ESAG9) protein, putative | 0 |
| Tb09.v1.0330 | expression site-associated gene 9 (ESAG9) protein, putative | 0 |
| Tb927.1.5220 | expression site-associated gene 9 (ESAG9) protein, putative | 0 |
| Tb927.1.3230 | cell division cycle protein, putative | 0 |
| Tb09.211.1380 | glycine cleavage system H protein, putative | 0 |
| Tb10.61.0980 | gMDH glycosomal malate dehydrogenase | 0 |
| Tb09.211.2830 | iron-sulfur cluster assembly protein, putative | 0 |
| Tb927.8.620 | KREPA3 RNA-editing complex protein MP42 | 0 |
| Tb10.70.2090 | KREPA6 RNA-editing complex protein,KREPA6 | 0 |
| Tb10.20.0130 | legume-like lectin, putative | 0 |
| Tb10.70.5100 | lysosomal alpha-mannosidase precursor, putative | 0 |
| Tb927.6.2790 | L-threonine 3-dehydrogenase, putative | 0 |
| Tb10.70.4420 | methionine biosynthetic protein, putative | 0 |
| Tb927.5.450 | NADH-ubiquinone oxidoreductase, mitochondrial, putative | 0 |

| | | |
|---|---|---|
| Tb11.01.7290 | NUDIX hydrolase, conserved | 0 |
| Tb09.160.5260 | oxidoreductase, putative | 0 |
| Tb927.6.2420 | p22 protein precursor | 0 |
| Tb927.3.1070 | peptide chain release factor 1, putative | 0 |
| Tb927.8.4810 | prohibitin | 0 |
| Tb927.4.1990 | RNA helicase, putative,mitochondrial, putative | 0 |
| Tb927.5.3750 | RNA-binding protein, putative | 0 |
| Tb927.6.4530 | RNA-binding protein, putative | 0 |
| Tb927.6.4530 | RNA-binding protein, putative | 0 |
| Tb10.61.1360 | RNA polymerase III C11 subunit, putative,DNA-directed RNA polymerase III, putative,RNA polymerase subunit, putative | 0 |
| Tb09.160.4450 | RPS3 40S ribosomal protein S3, putative | 0 |
| Tb927.4.1630 | RRP6 ribosomal RNA processing protein 6 | 0 |
| Tb11.01.2680 | 40S ribosomal protein SA, putative | 0 |
| Tb927.8.5860 | 50S ribosomal protein L17, putative | 0 |
| Tb09.211.4550 | 60S ribosomal protein L12, putative | 0 |
| Tb927.5.1710 | ribonucleoprotein p18, mitochondrial precursor, putative | 0 |
| Tb927.7.7420 | ATP synthase alpha chain, mitochondrial precursor,ATP synthase F1, alpha subunit | 0 |
| Tb927.7.7430 | ATP synthase alpha chain, mitochondrial precursor,ATP synthase F1, alpha subunit | 0 |
| Tb927.3.1380 | ATP synthase beta chain, mitochondrial precursor,ATP synthase F1, beta subunit | 0 |
| Tb927.6.4990 | ATP synthase, epsilon chain, putative | 0 |
| Tb927.6.4990 | ATP synthase, epsilon chain, putative | 0 |
| Tb927.7.6930 | ATPase, putative | 0 |
| Tb10.61.2840 | vacuolar ATP synthase subunit c, putative | 0 |
| Tb927.5.2790 | Pol beta-PAK mitochondrial DNA polymerase beta-PAK | 0 |
| Tb11.01.3660 | DNA repair and recombination helicase protein PIF1, putative,DNA repair and recombination protein, mitochondrial precursor, | 0 |
| Tb10.70.5110 | mMDH mitochondrial malate dehydrogenase | 0 |
| Tb11.01.5040 | mitochondrial carrier protein, putative | 0 |
| Tb10.70.0630 | ALDH aldehyde dehydrogenase, putative | 0 |
| Tb927.6.4210 | ALDH aldehyde dehydrogenase, putative | 0 |
| Tb09.142.0240 | variant surface glycoprotein (VSG), putative | 0 |
| Tb09.244.0470 | variant surface glycoprotein (VSG), putative | 0 |
| Tb09.244.1410 | variant surface glycoprotein (VSG), putative | 0 |
| Tb09.244.1740 | variant surface glycoprotein (VSG), putative | 0 |
| Tb09.354.0090 | variant surface glycoprotein (VSG), putative | 0 |

| | | |
|---|---|---|
| Tb09.v4.0004 | variant surface glycoprotein (VSG), putative | 0 |
| Tb09.v4.0005 | variant surface glycoprotein (VSG), putative | 0 |
| Tb10.v4.0024 | variant surface glycoprotein (VSG), putative | 0 |
| Tb10.v4.0025 | variant surface glycoprotein (VSG), putative | 0 |
| Tb10.v4.0031 | variant surface glycoprotein (VSG), putative | 0 |
| Tb11.09.0003 | variant surface glycoprotein (VSG), putative | 0 |
| Tb11.16.0001 | variant surface glycoprotein (VSG), putative | 0 |
| Tb11.16.0002 | variant surface glycoprotein (VSG), putative | 0 |
| Tb11.16.0003 | variant surface glycoprotein (VSG), putative | 0 |
| Tb11.38.0003 | variant surface glycoprotein (VSG), putative | 0 |
| Tb11.51.0005 | variant surface glycoprotein (VSG), putative | 0 |
| Tb11.57.0019 | variant surface glycoprotein (VSG), putative | 0 |
| Tb11.57.0024 | variant surface glycoprotein (VSG), putative | 0 |
| Tb11.57.0035 | variant surface glycoprotein (VSG), putative | 0 |
| Tb11.57.0084 | variant surface glycoprotein (VSG), putative | 0 |
| Tb11.v4.0010 | variant surface glycoprotein (VSG), putative | 0 |
| Tb11.v4.0015 | variant surface glycoprotein (VSG), putative | 0 |
| Tb11.v4.0016 | variant surface glycoprotein (VSG), putative | 0 |
| Tb11.v4.0029 | variant surface glycoprotein (VSG), putative | 0 |
| Tb11.v4.0033 | variant surface glycoprotein (VSG), putative | 0 |
| Tb11.v4.0034 | variant surface glycoprotein (VSG), putative | 0 |
| Tb11.v4.0035 | variant surface glycoprotein (VSG), putative | 0 |
| Tb11.v4.0036 | variant surface glycoprotein (VSG), putative | 0 |
| Tb11.v4.0038 | variant surface glycoprotein (VSG), putative | 0 |
| Tb11.v4.0040 | variant surface glycoprotein (VSG), putative | 0 |
| Tb11.v4.0050 | variant surface glycoprotein (VSG), putative | 0 |
| Tb11.v4.0063 | variant surface glycoprotein (VSG), putative | 0 |
| Tb11.v4.0067 | variant surface glycoprotein (VSG), putative | 0 |
| Tb11.v4.0068 | variant surface glycoprotein (VSG), putative | 0 |
| Tb11.v4.0070 | variant surface glycoprotein (VSG), putative | 0 |
| Tb927.1.05 | variant surface glycoprotein (VSG), putative | 0 |
| Tb927.1.5300 | variant surface glycoprotein (VSG), putative | 0 |
| Tb927.3.150 | variant surface glycoprotein (VSG), putative | 0 |
| Tb927.3.190 | variant surface glycoprotein (VSG), putative | 0 |

| | | |
|---|---|---|
| Tb927.3.340 | variant surface glycoprotein (VSG), putative | 0 |
| Tb927.3.440 | variant surface glycoprotein (VSG), putative | 0 |
| Tb927.3.490 | variant surface glycoprotein (VSG), putative | 0 |
| Tb927.4.5530 | variant surface glycoprotein (VSG), putative | 0 |
| Tb927.4.5560 | variant surface glycoprotein (VSG), putative | 0 |
| Tb927.5.4770 | variant surface glycoprotein (VSG), putative | 0 |
| Tb927.5.5080 | variant surface glycoprotein (VSG), putative | 0 |
| Tb927.5.5240 | variant surface glycoprotein (VSG), putative | 0 |
| Tb927.6.5260 | variant surface glycoprotein (VSG), putative | 0 |
| Tb927.6.5370 | variant surface glycoprotein (VSG), putative | 0 |
| Tb927.7.6500 | variant surface glycoprotein (VSG), putative | 0 |
| Tb10.v4.0001 | variant surface glycoprotein (VSG),variant surface glycoprotein VSG 10.1 | 0 |
| Tb09.160.5350 | variant surface glycoprotein (VSG)-related, putative | 0 |
| Tb09.244.2200 | variant surface glycoprotein (VSG)-related, putative | 0 |
| Tb09.244.2240 | variant surface glycoprotein (VSG)-related, putative | 0 |
| Tb09.244.2280 | variant surface glycoprotein (VSG)-related, putative | 0 |
| Tb09.244.2310 | variant surface glycoprotein (VSG)-related, putative | 0 |
| Tb09.244.2330 | variant surface glycoprotein (VSG)-related, putative | 0 |
| Tb09.v1.0300 | variant surface glycoprotein (VSG)-related, putative | 0 |
| Tb11.01.4560 | variant surface glycoprotein (VSG)-related, putative | 0 |
| Tb11.02.1566 | variant surface glycoprotein (VSG)-related, putative | 0 |
| Tb927.1.5060 | variant surface glycoprotein (VSG)-related, putative | 0 |
| Tb927.1.5170 | variant surface glycoprotein (VSG)-related, putative | 0 |
| Tb927.2.2060 | variant surface glycoprotein (VSG)-related, putative | 0 |
| Tb927.3.1500 | variant surface glycoprotein (VSG)-related, putative | 0 |
| Tb927.3.1510 | variant surface glycoprotein (VSG)-related, putative | 0 |
| Tb927.3.1520 | variant surface glycoprotein (VSG)-related, putative | 0 |
| Tb927.3.2540 | variant surface glycoprotein (VSG)-related, putative | 0 |
| Tb927.3.5680 | variant surface glycoprotein (VSG)-related, putative | 0 |
| Tb927.5.110 | variant surface glycoprotein (VSG)-related, putative | 0 |
| Tb927.5.130 | variant surface glycoprotein (VSG)-related, putative | 0 |
| Tb927.8.7300 | variant surface glycoprotein (VSG)-related, putative | 0 |
| Tb927.8.7320 | variant surface glycoprotein (VSG)-related, putative | 0 |
| Tb09.142.0100 | variant surface glycoprotein (VSG, atypical), putative | 0 |

| | | |
|---|---|---|
| Tb09.142.0230 | variant surface glycoprotein (VSG, atypical), putative | 0 |
| Tb09.160.0160 | variant surface glycoprotein (VSG, atypical), putative | 0 |
| Tb09.160.0280 | variant surface glycoprotein (VSG, atypical), putative | 0 |
| Tb09.244.0050 | variant surface glycoprotein (VSG, atypical), putative | 0 |
| Tb09.244.0200 | variant surface glycoprotein (VSG, atypical), putative | 0 |
| Tb09.244.0250 | variant surface glycoprotein (VSG, atypical), putative | 0 |
| Tb09.244.0640 | variant surface glycoprotein (VSG, atypical), putative | 0 |
| Tb09.244.1570 | variant surface glycoprotein (VSG, atypical), putative | 0 |
| Tb09.244.1600 | variant surface glycoprotein (VSG, atypical), putative | 0 |
| Tb09.244.1790 | variant surface glycoprotein (VSG, atypical), putative | 0 |
| Tb09.244.1830 | variant surface glycoprotein (VSG, atypical), putative | 0 |
| Tb09.354.0060 | variant surface glycoprotein (VSG, atypical), putative | 0 |
| Tb09.354.0180 | variant surface glycoprotein (VSG, atypical), putative | 0 |
| Tb11.13.0004 | variant surface glycoprotein (VSG, atypical), putative | 0 |
| Tb11.14.0007 | variant surface glycoprotein (VSG, atypical), putative | 0 |
| Tb11.14.0008 | variant surface glycoprotein (VSG, atypical), putative | 0 |
| Tb11.14.0019 | variant surface glycoprotein (VSG, atypical), putative | 0 |
| Tb11.21.0004 | variant surface glycoprotein (VSG, atypical), putative | 0 |
| Tb11.24.0007 | variant surface glycoprotein (VSG, atypical), putative | 0 |
| Tb11.24.0011 | variant surface glycoprotein (VSG, atypical), putative | 0 |
| Tb11.24.0012 | variant surface glycoprotein (VSG, atypical), putative | 0 |
| Tb11.35.0001 | variant surface glycoprotein (VSG, atypical), putative | 0 |
| Tb11.38.0005 | variant surface glycoprotein (VSG, atypical), putative | 0 |
| Tb11.57.0052 | variant surface glycoprotein (VSG, atypical), putative | 0 |
| Tb927.1.5330 | variant surface glycoprotein (VSG, atypical), putative | 0 |
| Tb927.2.6410 | variant surface glycoprotein (VSG, atypical), putative | 0 |
| Tb927.3.210 | variant surface glycoprotein (VSG, atypical), putative | 0 |
| Tb927.4.5410 | variant surface glycoprotein (VSG, atypical), putative | 0 |
| Tb927.4.5420 | variant surface glycoprotein (VSG, atypical), putative | 0 |
| Tb927.4.5430 | variant surface glycoprotein (VSG, atypical), putative | 0 |
| Tb927.4.5580 | variant surface glycoprotein (VSG, atypical), putative | 0 |
| Tb927.4.5700 | variant surface glycoprotein (VSG, atypical), putative | 0 |
| Tb927.5.230 | variant surface glycoprotein (VSG, atypical), putative | 0 |
| Tb927.5.3990 | variant surface glycoprotein (VSG, atypical), putative | 0 |

| | | |
|---|---|---|
| Tb927.5.4670 | variant surface glycoprotein (VSG, atypical), putative | 0 |
| Tb927.5.4690 | variant surface glycoprotein (VSG, atypical), putative | 0 |
| Tb927.5.4730 | variant surface glycoprotein (VSG, atypical), putative | 0 |
| Tb927.5.4810 | variant surface glycoprotein (VSG, atypical), putative | 0 |
| Tb927.5.4840 | variant surface glycoprotein (VSG, atypical), putative | 0 |
| Tb927.5.4950 | variant surface glycoprotein (VSG, atypical), putative | 0 |
| Tb927.5.5050 | variant surface glycoprotein (VSG, atypical), putative | 0 |
| Tb927.5.5210 | variant surface glycoprotein (VSG, atypical), putative | 0 |
| Tb927.6.5240 | variant surface glycoprotein (VSG, atypical), putative | 0 |
| Tb927.6.5550 | variant surface glycoprotein (VSG, atypical), putative | 0 |
| Tb927.6.5740 | variant surface glycoprotein (VSG, atypical), putative | 0 |
| Tb927.8.460 | variant surface glycoprotein (VSG, atypical), putative | 0 |
| Tb09.211.4070 | hypothetical protein | 0 |
| Tb09.244.0370 | hypothetical protein | 0 |
| Tb09.244.0810 | hypothetical protein | 0 |
| Tb09.v4.0006 | hypothetical protein | 0 |
| Tb10.389.0400 | hypothetical protein | 0 |
| Tb10.6k15.0880 | hypothetical protein | 0 |
| Tb10.70.0560 | hypothetical protein | 0 |
| Tb10.70.1260 | hypothetical protein | 0 |
| Tb10.70.2840 | hypothetical protein | 0 |
| Tb10.70.2850 | hypothetical protein | 0 |
| Tb10.70.3780 | hypothetical protein | 0 |
| Tb10.70.4480 | hypothetical protein | 0 |
| Tb10.70.5580 | hypothetical protein | 0 |
| Tb10.70.6250 | hypothetical protein | 0 |
| Tb10.v4.0017 | hypothetical protein | 0 |
| Tb11.01.0550 | hypothetical protein | 0 |
| Tb11.01.2860 | hypothetical protein | 0 |
| Tb11.01.3220 | hypothetical protein | 0 |
| Tb11.01.3810 | hypothetical protein | 0 |
| Tb11.02.1565 | hypothetical protein | 0 |
| Tb11.02.2360 | hypothetical protein | 0 |
| Tb11.02.2370 | hypothetical protein | 0 |

| | | |
|---|---|---|
| Tb11.12.0017 | hypothetical protein | 0 |
| Tb11.42.0001 | hypothetical protein | 0 |
| Tb11.v4.0008 | hypothetical protein | 0 |
| Tb927.1.2540 | hypothetical protein | 0 |
| Tb927.2.940 | hypothetical protein | 0 |
| Tb927.3.1460 | hypothetical protein | 0 |
| Tb927.3.2500 | hypothetical protein | 0 |
| Tb927.3.2590 | hypothetical protein | 0 |
| Tb927.4.140 | hypothetical protein | 0 |
| Tb927.4.2100 | hypothetical protein | 0 |
| Tb927.5.4010 | hypothetical protein | 0 |
| Tb927.5.4020 | hypothetical protein | 0 |
| Tb927.6.440 | hypothetical protein | 0 |
| Tb927.7.180 | hypothetical protein | 0 |
| Tb927.7.6580 | hypothetical protein | 0 |
| Tb927.8.490 | hypothetical protein | 0 |
| Tb927.8.510 | hypothetical protein | 0 |
| Tb927.8.510 | hypothetical protein | 0 |
| Tb927.8.5790 | hypothetical protein | 0 |
| Tb927.8.5910 | hypothetical protein | 0 |
| Tb927.8.5920 | hypothetical protein | 0 |
| Tb927.8.7190 | hypothetical protein | 0 |
| Tb927.8.7310 | hypothetical protein | 0 |
| Tb927.8.7330 | hypothetical protein | 0 |
| Tb927.8.930 | hypothetical protein | 0 |
| Tb927.8.950 | hypothetical protein | 0 |
| Tb927.8.970 | hypothetical protein | 0 |
| Tb09.142.0370 | hypothetical protein, conserved | 0 |
| Tb09.160.0400 | hypothetical protein, conserved | 0 |
| Tb09.160.0550 | hypothetical protein, conserved | 0 |
| Tb09.160.1030 | hypothetical protein, conserved | 0 |
| Tb09.160.1490 | hypothetical protein, conserved | 0 |
| Tb09.160.3140 | hypothetical protein, conserved | 0 |
| Tb09.160.3490 | hypothetical protein, conserved | 0 |

| | | |
|---|---|---|
| Tb09.160.4660 | hypothetical protein, conserved | 0 |
| Tb09.160.5180 | hypothetical protein, conserved | 0 |
| Tb09.211.0010 | hypothetical protein, conserved | 0 |
| Tb09.211.0800 | hypothetical protein, conserved | 0 |
| Tb09.211.1270 | hypothetical protein, conserved | 0 |
| Tb09.211.1280 | hypothetical protein, conserved | 0 |
| Tb09.211.1300 | hypothetical protein, conserved | 0 |
| Tb09.211.1390 | hypothetical protein, conserved | 0 |
| Tb09.211.1600 | hypothetical protein, conserved | 0 |
| Tb09.211.1810 | hypothetical protein, conserved | 0 |
| Tb09.211.1900 | hypothetical protein, conserved | 0 |
| Tb09.211.2200 | hypothetical protein, conserved | 0 |
| Tb09.211.2810 | hypothetical protein, conserved | 0 |
| Tb09.211.3720 | hypothetical protein, conserved | 0 |
| Tb09.211.3800 | hypothetical protein, conserved | 0 |
| Tb09.211.3900 | hypothetical protein, conserved | 0 |
| Tb09.244.2190 | hypothetical protein, conserved | 0 |
| Tb09.244.2230 | hypothetical protein, conserved | 0 |
| Tb09.244.2270 | hypothetical protein, conserved | 0 |
| Tb09.v1.0450 | hypothetical protein, conserved | 0 |
| Tb09.v1.0460 | hypothetical protein, conserved | 0 |
| Tb09.v1.0470 | hypothetical protein, conserved | 0 |
| Tb09.v1.0480 | hypothetical protein, conserved | 0 |
| Tb09.v1.0500 | hypothetical protein, conserved | 0 |
| Tb09.v1.0530 | hypothetical protein, conserved | 0 |
| Tb10.26.0630 | hypothetical protein, conserved | 0 |
| Tb10.26.0690 | hypothetical protein, conserved | 0 |
| Tb10.26.0910 | hypothetical protein, conserved | 0 |
| Tb10.389.0610 | hypothetical protein, conserved | 0 |
| Tb10.389.1780 | hypothetical protein, conserved | 0 |
| Tb10.406.0110 | hypothetical protein, conserved | 0 |
| Tb10.61.0460 | hypothetical protein, conserved | 0 |
| Tb10.61.0480 | hypothetical protein, conserved | 0 |
| Tb10.61.2390 | hypothetical protein, conserved | 0 |

| | | |
|---|---|---|
| Tb10.61.2850 | hypothetical protein, conserved | 0 |
| Tb10.6k15.0080 | hypothetical protein, conserved | 0 |
| Tb10.6k15.0150 | hypothetical protein, conserved | 0 |
| Tb10.6k15.0160 | hypothetical protein, conserved | 0 |
| Tb10.6k15.0180 | hypothetical protein, conserved | 0 |
| Tb10.6k15.0280 | hypothetical protein, conserved | 0 |
| Tb10.6k15.0480 | hypothetical protein, conserved | 0 |
| Tb10.6k15.1030 | hypothetical protein, conserved | 0 |
| Tb10.6k15.1040 | hypothetical protein, conserved | 0 |
| Tb10.6k15.1110 | hypothetical protein, conserved | 0 |
| Tb10.6k15.1270 | hypothetical protein, conserved | 0 |
| Tb10.6k15.1870 | hypothetical protein, conserved | 0 |
| Tb10.6k15.2300 | hypothetical protein, conserved | 0 |
| Tb10.6k15.2820 | hypothetical protein, conserved | 0 |
| Tb10.6k15.3660 | hypothetical protein, conserved | 0 |
| Tb10.70.1280 | hypothetical protein, conserved | 0 |
| Tb10.70.1290 | hypothetical protein, conserved | 0 |
| Tb10.70.1450 | hypothetical protein, conserved | 0 |
| Tb10.70.1650 | hypothetical protein, conserved | 0 |
| Tb10.70.2150 | hypothetical protein, conserved | 0 |
| Tb10.70.3630 | hypothetical protein, conserved | 0 |
| Tb10.70.3940 | hypothetical protein, conserved | 0 |
| Tb10.70.4430 | hypothetical protein, conserved | 0 |
| Tb10.70.4590 | hypothetical protein, conserved | 0 |
| Tb10.70.5630 | hypothetical protein, conserved | 0 |
| Tb10.70.6720 | hypothetical protein, conserved | 0 |
| Tb10.70.6740 | hypothetical protein, conserved | 0 |
| Tb10.70.7150 | hypothetical protein, conserved | 0 |
| Tb11.01.0070 | hypothetical protein, conserved | 0 |
| Tb11.01.0130 | hypothetical protein, conserved | 0 |
| Tb11.01.0240 | hypothetical protein, conserved | 0 |
| Tb11.01.0880 | hypothetical protein, conserved | 0 |
| Tb11.01.1220 | hypothetical protein, conserved | 0 |
| Tb11.01.1590 | hypothetical protein, conserved | 0 |

| | | |
|---|---|---|
| Tb11.01.1660 | hypothetical protein, conserved | 0 |
| Tb11.01.2470 | hypothetical protein, conserved | 0 |
| Tb11.01.2690 | hypothetical protein, conserved | 0 |
| Tb11.01.2740 | hypothetical protein, conserved | 0 |
| Tb11.01.3570 | hypothetical protein, conserved | 0 |
| Tb11.01.3860 | hypothetical protein, conserved | 0 |
| Tb11.01.4290 | hypothetical protein, conserved | 0 |
| Tb11.01.4530 | hypothetical protein, conserved | 0 |
| Tb11.01.4900 | hypothetical protein, conserved | 0 |
| Tb11.01.5200 | hypothetical protein, conserved | 0 |
| Tb11.01.5920 | hypothetical protein, conserved | 0 |
| Tb11.01.6710 | hypothetical protein, conserved | 0 |
| Tb11.01.7710 | hypothetical protein, conserved | 0 |
| Tb11.01.7840 | hypothetical protein, conserved | 0 |
| Tb11.01.8580 | hypothetical protein, conserved | 0 |
| Tb11.02.0351 | hypothetical protein, conserved | 0 |
| Tb11.02.0445 | hypothetical protein, conserved | 0 |
| Tb11.02.0650 | hypothetical protein, conserved | 0 |
| Tb11.02.1150 | hypothetical protein, conserved | 0 |
| Tb11.02.1550 | hypothetical protein, conserved | 0 |
| Tb11.02.1890 | hypothetical protein, conserved | 0 |
| Tb11.02.1910 | hypothetical protein, conserved | 0 |
| Tb11.02.2320 | hypothetical protein, conserved | 0 |
| Tb11.02.2420 | hypothetical protein, conserved | 0 |
| Tb11.02.2770 | hypothetical protein, conserved | 0 |
| Tb11.02.2840 | hypothetical protein, conserved | 0 |
| Tb11.02.3230 | hypothetical protein, conserved | 0 |
| Tb11.02.3930 | hypothetical protein, conserved | 0 |
| Tb11.02.4120 | hypothetical protein, conserved | 0 |
| Tb11.02.4460 | hypothetical protein, conserved | 0 |
| Tb11.02.4600 | hypothetical protein, conserved | 0 |
| Tb11.02.4810 | hypothetical protein, conserved | 0 |
| Tb11.02.5340 | hypothetical protein, conserved | 0 |
| Tb11.03.0060 | hypothetical protein, conserved | 0 |

| | | |
|---|---|---|
| Tb11.03.0240 | hypothetical protein, conserved | 0 |
| Tb11.03.0470 | hypothetical protein, conserved | 0 |
| Tb11.03.0900 | hypothetical protein, conserved | 0 |
| Tb11.12.0004 | hypothetical protein, conserved | 0 |
| Tb11.18.0001 | hypothetical protein, conserved | 0 |
| Tb11.47.0016 | hypothetical protein, conserved | 0 |
| Tb11.50.0004 | hypothetical protein, conserved | 0 |
| Tb11.52.0016 | hypothetical protein, conserved | 0 |
| Tb11.55.0010 | hypothetical protein, conserved | 0 |
| Tb11.55.0022 | hypothetical protein, conserved | 0 |
| Tb927.1.1040 | hypothetical protein, conserved | 0 |
| Tb927.1.1060 | hypothetical protein, conserved | 0 |
| Tb927.1.1540 | hypothetical protein, conserved | 0 |
| Tb927.1.1650 | hypothetical protein, conserved | 0 |
| Tb927.1.2730 | hypothetical protein, conserved | 0 |
| Tb927.1.2970 | hypothetical protein, conserved | 0 |
| Tb927.1.4220 | hypothetical protein, conserved | 0 |
| Tb927.1.790 | hypothetical protein, conserved | 0 |
| Tb927.2.2530 | hypothetical protein, conserved | 0 |
| Tb927.2.2950 | hypothetical protein, conserved | 0 |
| Tb927.2.3180 | hypothetical protein, conserved | 0 |
| Tb927.2.4460 | hypothetical protein, conserved | 0 |
| Tb927.2.4990 | hypothetical protein, conserved | 0 |
| Tb927.3.1860 | hypothetical protein, conserved | 0 |
| Tb927.3.2010 | hypothetical protein, conserved | 0 |
| Tb927.3.2050 | hypothetical protein, conserved | 0 |
| Tb927.3.2550 | hypothetical protein, conserved | 0 |
| Tb927.3.2560 | hypothetical protein, conserved | 0 |
| Tb927.3.2570 | hypothetical protein, conserved | 0 |
| Tb927.3.4440 | hypothetical protein, conserved | 0 |
| Tb927.3.4700 | hypothetical protein, conserved | 0 |
| Tb927.3.4740 | hypothetical protein, conserved | 0 |
| Tb927.3.4780 | hypothetical protein, conserved | 0 |
| Tb927.3.4870 | hypothetical protein, conserved | 0 |

| | | |
|---|---|---|
| Tb927.3.5300 | hypothetical protein, conserved | 0 |
| Tb927.3.5390 | hypothetical protein, conserved | 0 |
| Tb927.3.5600 | hypothetical protein, conserved | 0 |
| Tb927.3.5690 | hypothetical protein, conserved | 0 |
| Tb927.3.5700 | hypothetical protein, conserved | 0 |
| Tb927.3.5710 | hypothetical protein, conserved | 0 |
| Tb927.3.5720 | hypothetical protein, conserved | 0 |
| Tb927.3.5730 | hypothetical protein, conserved | 0 |
| Tb927.3.5760 | hypothetical protein, conserved | 0 |
| Tb927.4.1110 | hypothetical protein, conserved | 0 |
| Tb927.4.1160 | hypothetical protein, conserved | 0 |
| Tb927.4.1960 | hypothetical protein, conserved | 0 |
| Tb927.4.2490 | hypothetical protein, conserved | 0 |
| Tb927.4.2980 | hypothetical protein, conserved | 0 |
| Tb927.4.3200 | hypothetical protein, conserved | 0 |
| Tb927.4.3220 | hypothetical protein, conserved | 0 |
| Tb927.4.3240 | hypothetical protein, conserved | 0 |
| Tb927.4.3290 | hypothetical protein, conserved | 0 |
| Tb927.4.4370 | hypothetical protein, conserved | 0 |
| Tb927.4.4640 | hypothetical protein, conserved | 0 |
| Tb927.4.910 | hypothetical protein, conserved | 0 |
| Tb927.5.1030 | hypothetical protein, conserved | 0 |
| Tb927.5.150 | hypothetical protein, conserved | 0 |
| Tb927.5.2160 | hypothetical protein, conserved | 0 |
| Tb927.5.2170 | hypothetical protein, conserved | 0 |
| Tb927.5.2200 | hypothetical protein, conserved | 0 |
| Tb927.5.2230 | hypothetical protein, conserved | 0 |
| Tb927.5.2260 | hypothetical protein, conserved | 0 |
| Tb927.5.2600 | hypothetical protein, conserved | 0 |
| Tb927.5.2670 | hypothetical protein, conserved | 0 |
| Tb927.5.4080 | hypothetical protein, conserved | 0 |
| Tb927.5.4100 | hypothetical protein, conserved | 0 |
| Tb927.5.830 | hypothetical protein, conserved | 0 |
| Tb927.5.960 | hypothetical protein, conserved | 0 |

| | | |
|---|---|---|
| Tb927.6.1310 | hypothetical protein, conserved | 0 |
| Tb927.6.1330 | hypothetical protein, conserved | 0 |
| Tb927.6.1350 | hypothetical protein, conserved | 0 |
| Tb927.6.1370 | hypothetical protein, conserved | 0 |
| Tb927.6.1390 | hypothetical protein, conserved | 0 |
| Tb927.6.1440 | hypothetical protein, conserved | 0 |
| Tb927.6.1710 | hypothetical protein, conserved | 0 |
| Tb927.6.1730 | hypothetical protein, conserved | 0 |
| Tb927.6.2140 | hypothetical protein, conserved | 0 |
| Tb927.6.2490 | hypothetical protein, conserved | 0 |
| Tb927.6.2730 | hypothetical protein, conserved | 0 |
| Tb927.6.2990 | hypothetical protein, conserved | 0 |
| Tb927.6.4130 | hypothetical protein, conserved | 0 |
| Tb927.6.4240 | hypothetical protein, conserved | 0 |
| Tb927.6.4250 | hypothetical protein, conserved | 0 |
| Tb927.6.4940 | hypothetical protein, conserved | 0 |
| Tb927.6.4940 | hypothetical protein, conserved | 0 |
| Tb927.6.660 | hypothetical protein, conserved | 0 |
| Tb927.7.1080 | hypothetical protein, conserved | 0 |
| Tb927.7.1150 | hypothetical protein, conserved | 0 |
| Tb927.7.1250 | hypothetical protein, conserved | 0 |
| Tb927.7.1270 | hypothetical protein, conserved | 0 |
| Tb927.7.2690 | hypothetical protein, conserved | 0 |
| Tb927.7.3010 | hypothetical protein, conserved | 0 |
| Tb927.7.3240 | hypothetical protein, conserved | 0 |
| Tb927.7.3270 | hypothetical protein, conserved | 0 |
| Tb927.7.340 | hypothetical protein, conserved | 0 |
| Tb927.7.360 | hypothetical protein, conserved | 0 |
| Tb927.7.3750 | hypothetical protein, conserved | 0 |
| Tb927.7.380 | hypothetical protein, conserved | 0 |
| Tb927.7.3890 | hypothetical protein, conserved | 0 |
| Tb927.7.3930 | hypothetical protein, conserved | 0 |
| Tb927.7.400 | hypothetical protein, conserved | 0 |
| Tb927.7.420 | hypothetical protein, conserved | 0 |

| | | |
|---|---|---|
| Tb927.7.4310 | hypothetical protein, conserved | 0 |
| Tb927.7.440 | hypothetical protein, conserved | 0 |
| Tb927.7.4820 | hypothetical protein, conserved | 0 |
| Tb927.7.590 | hypothetical protein, conserved | 0 |
| Tb927.7.6370 | hypothetical protein, conserved | 0 |
| Tb927.7.6420 | hypothetical protein, conserved | 0 |
| Tb927.7.6490 | hypothetical protein, conserved | 0 |
| Tb927.7.6560 | hypothetical protein, conserved | 0 |
| Tb927.7.6600 | hypothetical protein, conserved | 0 |
| Tb927.8.1250 | hypothetical protein, conserved | 0 |
| Tb927.8.1700 | hypothetical protein, conserved | 0 |
| Tb927.8.1740 | hypothetical protein, conserved | 0 |
| Tb927.8.2390 | hypothetical protein, conserved | 0 |
| Tb927.8.2680 | hypothetical protein, conserved | 0 |
| Tb927.8.2690 | hypothetical protein, conserved | 0 |
| Tb927.8.3040 | hypothetical protein, conserved | 0 |
| Tb927.8.3960 | hypothetical protein, conserved | 0 |
| Tb927.8.4230 | hypothetical protein, conserved | 0 |
| Tb927.8.4240 | hypothetical protein, conserved | 0 |
| Tb927.8.4760 | hypothetical protein, conserved | 0 |
| Tb927.8.5360 | hypothetical protein, conserved | 0 |
| Tb927.8.5540 | hypothetical protein, conserved | 0 |
| Tb927.8.6080 | hypothetical protein, conserved | 0 |
| Tb927.8.6480 | hypothetical protein, conserved | 0 |
| Tb927.8.6550 | hypothetical protein, conserved | 0 |
| Tb927.8.8120 | hypothetical protein, conserved | 0 |
| Tb927.8.8170 | hypothetical protein, conserved | 0 |
| Tb927.4.3270 | hypothetical protein, conserved,ESAG11-related protein, putative | 0 |
| Tb11.46.0011 | hypothetical protein, conserved,leucine-rich repeat protein (LRRP), putative | 0 |
| Tb10.70.0930 | hypothetical protein, conserved,predicted tripartite motif protein,predicted zinc finger protein | 0 |
| Tb09.160.0330 | hypothetical protein, unlikely | 0 |
| Tb09.160.1600 | hypothetical protein, unlikely | 0 |
| Tb09.160.1850 | hypothetical protein, unlikely | 0 |
| Tb09.160.2720 | hypothetical protein, unlikely | 0 |

| | | |
|---|---|---|
| Tb09.160.2740 | hypothetical protein, unlikely | 0 |
| Tb09.160.3100 | hypothetical protein, unlikely | 0 |
| Tb09.160.3620 | hypothetical protein, unlikely | 0 |
| Tb09.160.4110 | hypothetical protein, unlikely | 0 |
| Tb09.160.4510 | hypothetical protein, unlikely | 0 |
| Tb09.160.4880 | hypothetical protein, unlikely | 0 |
| Tb09.160.4980 | hypothetical protein, unlikely | 0 |
| Tb09.160.5090 | hypothetical protein, unlikely | 0 |
| Tb09.160.5340 | hypothetical protein, unlikely | 0 |
| Tb09.160.5450 | hypothetical protein, unlikely | 0 |
| Tb09.211.0360 | hypothetical protein, unlikely | 0 |
| Tb09.211.0490 | hypothetical protein, unlikely | 0 |
| Tb09.211.0570 | hypothetical protein, unlikely | 0 |
| Tb09.211.0640 | hypothetical protein, unlikely | 0 |
| Tb09.211.0650 | hypothetical protein, unlikely | 0 |
| Tb09.211.0660 | hypothetical protein, unlikely | 0 |
| Tb09.211.0670 | hypothetical protein, unlikely | 0 |
| Tb09.211.1340 | hypothetical protein, unlikely | 0 |
| Tb09.211.1420 | hypothetical protein, unlikely | 0 |
| Tb09.211.2000 | hypothetical protein, unlikely | 0 |
| Tb09.211.2170 | hypothetical protein, unlikely | 0 |
| Tb09.211.2390 | hypothetical protein, unlikely | 0 |
| Tb09.211.2480 | hypothetical protein, unlikely | 0 |
| Tb09.211.2820 | hypothetical protein, unlikely | 0 |
| Tb09.211.4790 | hypothetical protein, unlikely | 0 |
| Tb09.244.1270 | hypothetical protein, unlikely | 0 |
| Tb09.244.2040 | hypothetical protein, unlikely | 0 |
| Tb09.244.2360 | hypothetical protein, unlikely | 0 |
| Tb09.v1.0090 | hypothetical protein, unlikely | 0 |
| Tb09.v1.0110 | hypothetical protein, unlikely | 0 |
| Tb09.v1.0430 | hypothetical protein, unlikely | 0 |
| Tb09.v1.0860 | hypothetical protein, unlikely | 0 |
| Tb10.6k15.0230 | hypothetical protein, unlikely | 0 |
| Tb927.1.1110 | hypothetical protein, unlikely | 0 |

| | | |
|---|---|---|
| Tb927.1.1180 | hypothetical protein, unlikely | 0 |
| Tb927.1.1250 | hypothetical protein, unlikely | 0 |
| Tb927.1.1570 | hypothetical protein, unlikely | 0 |
| Tb927.1.1870 | hypothetical protein, unlikely | 0 |
| Tb927.1.2440 | hypothetical protein, unlikely | 0 |
| Tb927.1.2460 | hypothetical protein, unlikely | 0 |
| Tb927.1.2480 | hypothetical protein, unlikely | 0 |
| Tb927.1.2500 | hypothetical protein, unlikely | 0 |
| Tb927.1.2520 | hypothetical protein, unlikely | 0 |
| Tb927.1.2560 | hypothetical protein, unlikely | 0 |
| Tb927.1.2900 | hypothetical protein, unlikely | 0 |
| Tb927.1.2940 | hypothetical protein, unlikely | 0 |
| Tb927.1.3370 | hypothetical protein, unlikely | 0 |
| Tb927.1.3380 | hypothetical protein, unlikely | 0 |
| Tb927.1.3490 | hypothetical protein, unlikely | 0 |
| Tb927.1.3640 | hypothetical protein, unlikely | 0 |
| Tb927.1.3720 | hypothetical protein, unlikely | 0 |
| Tb927.1.4820 | hypothetical protein, unlikely | 0 |
| Tb927.1.50 | hypothetical protein, unlikely | 0 |
| Tb927.1.5010 | hypothetical protein, unlikely | 0 |
| Tb927.2.3270 | 65 kDa invariant surface glycoprotein | 1 |
| Tb927.2.3280 | 65 kDa invariant surface glycoprotein | 1 |
| Tb927.2.3290 | 65 kDa invariant surface glycoprotein | 1 |
| Tb927.2.3300 | 65 kDa invariant surface glycoprotein | 1 |
| Tb927.2.3310 | 65 kDa invariant surface glycoprotein | 1 |
| Tb927.2.3320 | 65 kDa invariant surface glycoprotein | 1 |
| Tb11.47.0001 | 65 kDa invariant surface glycoprotein-like protein | 1 |
| Tb927.5.1430 | ISG64 64 kDa invariant surface glycoprotei | 1 |
| Tb927.5.1390 | ISG64 64 kDa invariant surface glycoprotein | 1 |
| Tb927.5.1410 | ISG64 64 kDa invariant surface glycoprotein | 1 |
| Tb927.5.360 | ISG75 75 kDa invariant surface glycoprotein | 1 |
| Tb927.5.350 | ISG75 75 kDa invariant surface glycoprotein, putative | 1 |
| Tb927.5.370 | ISG75 75 kDa invariant surface glycoprotein, putative | 1 |
| Tb927.5.380 | ISG75 75 kDa invariant surface glycoprotein, putative | 1 |

| | | |
|---|---|---|
| Tb927.5.400 | ISG75 75 kDa invariant surface glycoprotein, putative | 1 |
| Tb927.2.6130 | ABC transporter, putative | 1 |
| Tb927.2.5410 | ABC transporter, putative,multidrug resistance protein | 1 |
| Tb10.6k15.3720 | acid phosphatase, putative | 1 |
| Tb927.5.630 | acidic phosphatase, putative | 1 |
| Tb10.6k15.3560 | MBAP2 membrane-bound acid phosphatase 2 | 1 |
| Tb10.61.0100 | protein kinase, putative | 1 |
| Tb927.6.4630 | serine/threonine protein phosphatase, putative | 1 |
| Tb927.6.4630 | serine/threonine protein phosphatase, putative | 1 |
| Tb11.55.0012 | vesicular-fusion protein SEC18, putative | 1 |
| Tb11.55.0014 | vesicular transport protein (CDC48 homologue), putative | 1 |
| Tb927.4.4460 | GRESAG 4.4B receptor-type adenylate cyclase GRESAG 4, putative,receptor-type adenylate cyclase GRESAG 4.4B | 1 |
| Tb11.27.0001 | receptor-type adenylate cyclase GRESAG 4, putative | 1 |
| Tb927.4.4410 | receptor-type adenylate cyclase GRESAG 4, putative | 1 |
| Tb927.4.4430 | receptor-type adenylate cyclase GRESAG 4, putative | 1 |
| Tb927.4.4440 | receptor-type adenylate cyclase GRESAG 4, putative | 1 |
| Tb927.4.4450 | receptor-type adenylate cyclase GRESAG 4, putative | 1 |
| Tb927.4.4470 | receptor-type adenylate cyclase GRESAG 4, putative | 1 |
| Tb927.5.650 | receptor-type adenylate cyclase GRESAG 4, putative | 1 |
| Tb927.6.190 | receptor-type adenylate cyclase GRESAG 4, putative | 1 |
| Tb927.7.6040 | receptor-type adenylate cyclase GRESAG 4, putative | 1 |
| Tb927.7.6050 | receptor-type adenylate cyclase GRESAG 4, putative | 1 |
| Tb927.7.6070 | receptor-type adenylate cyclase GRESAG 4, putative | 1 |
| Tb927.7.6080 | receptor-type adenylate cyclase GRESAG 4, putative | 1 |
| Tb927.7.7470 | receptor-type adenylate cyclase GRESAG 4, putative | 1 |
| Tb927.8.7590 | receptor-type adenylate cyclase GRESAG 4, putative | 1 |
| Tb927.8.7870 | receptor-type adenylate cyclase GRESAG 4, putative | 1 |
| Tb927.5.320 | receptor-type adenylate cyclase GRESAG 4, putative,adenylyl cyclase, putative | 1 |
| Tb927.5.330 | receptor-type adenylate cyclase GRESAG 4, putative,adenylyl cyclase, putative,ATP pyrophosphate-lyase, putative | 1 |
| Tb927.5.4540 | receptor-type adenylate cyclase GRESAG 4, putative,adenylyl cyclase, putative,ATP pyrophosphate-lyase, putative | 1 |
| Tb927.5.4550 | receptor-type adenylate cyclase GRESAG 4, putative,adenylyl cyclase, putative,ATP pyrophosphate-lyase, putative | 1 |
| Tb10.389.0430 | receptor-type adenylate cyclase GRESAG 4, putative,ESAG 4 (expression site-associated gene 4) protein, putative | 1 |
| Tb927.7.1930 | nucleoside diphosphatase, putative,ectonucleoside triphosphate diphosphohydrolase 5, putative | 1 |
| Tb927.8.3800 | nucleoside phosphatase, putative,guanosine diphosphatase, putative | 1 |

| | | |
|---|---|---|
| Tb927.6.2890 | single strand-specific nuclease, putative | 1 |
| Tb11.02.2880 | chaperone protein DNAJ, putative | 1 |
| Tb927.7.6200 | chaperone protein DNAJ, putative | 1 |
| Tb09.160.3090 | heat shock protein, putative,HSP70-like protein | 1 |
| Tb927.8.2000 | PPIase cyclophilin type peptidyl-prolyl cis-trans isomerase, putative,peptidyl-prolyl cis-trans isomerase, putative | 1 |
| Tb10.70.7080 | CBP1 serine carboxypeptidase III precursor, putative,serine peptidase, Clan SC, Family S10 | 1 |
| Tb11.02.1280 | subtilisin-like serine peptidase,serine peptidase, clan SB, family S8-like protein | 1 |
| Tb927.3.4230 | subtilisin-like serine peptidase,serine peptidase, clan SB, family S8-like protein | 1 |
| Tb09.211.2310 | Bem46-like serine peptidase,Serine peptidase, Clan SC, Family S09X | 1 |
| Tb927.6.1000 | CP cysteine peptidase precursor,cysteine peptidase, Clan CA, family C1, Cathepsin L-like | 1 |
| Tb927.6.1010 | CP cysteine peptidase precursor,cysteine peptidase, Clan CA, family C1, Cathepsin L-like | 1 |
| Tb927.6.1020 | CP cysteine peptidase precursor,cysteine peptidase, Clan CA, family C1, Cathepsin L-like | 1 |
| Tb927.6.1030 | CP cysteine peptidase precursor,cysteine peptidase, Clan CA, family C1, Cathepsin L-like | 1 |
| Tb927.6.1040 | CP cysteine peptidase precursor,cysteine peptidase, Clan CA, family C1, Cathepsin L-like | 1 |
| Tb927.6.1050 | CP cysteine peptidase precursor,cysteine peptidase, Clan CA, family C1, Cathepsin L-like | 1 |
| Tb927.6.1060 | CP cysteine peptidase precursor,cysteine peptidase, Clan CA, family C1, Cathepsin L-like | 1 |
| Tb927.6.960 | CP cysteine peptidase precursor,cysteine peptidase, Clan CA, family C1, Cathepsin L-like | 1 |
| Tb927.6.970 | CP cysteine peptidase precursor,cysteine peptidase, Clan CA, family C1, Cathepsin L-like | 1 |
| Tb927.6.980 | CP cysteine peptidase precursor,cysteine peptidase, Clan CA, family C1, Cathepsin L-like | 1 |
| Tb927.6.990 | CP cysteine peptidase precursor,cysteine peptidase, Clan CA, family C1, Cathepsin L-like | 1 |
| Tb09.211.3330 | cystathione gamma lyase, putative | 1 |
| Tb927.4.2450 | thioredoxin, putative | 1 |
| Tb927.3.4650 | C-8 sterol isomerase, putative | 1 |
| Tb927.1.1130 | glycerol-3-phosphate dehydrogenase (FAD-dependent), putative | 1 |
| Tb11.01.0580 | glycerophosphoryl diester phosphodiesterase, putative | 1 |
| Tb927.4.1020 | serine-palmitoyl-CoA transferase, putative | 1 |
| Tb927.3.3580 | LPG3 lipophosphoglycan biosynthetic protein, putative,heat shock protein 90, putative,glucose regulated protein 94, putative | 1 |
| Tb927.8.1720 | phosphatidylglycerophosphate synthase-like protein, putative | 1 |
| Tb11.01.6800 | 1-acyl-sn-glycerol-3-phosphate acyltransferase protein, putative | 1 |
| Tb11.01.3900 | GPI12 N-acetylglucosaminylphosphatidylinositoldeacetylase | 1 |
| Tb927.4.2230 | glycosyltransferase ALG2, putative | 1 |
| Tb10.389.1450 | UDP-Gal or UDP-GlcNAc-dependent glycosyltransferase, putative | 1 |
| Tb927.2.3370 | UDP-Gal or UDP-GlcNAc-dependent glycosyltransferase, putative | 1 |
| Tb927.3.5660 | UDP-Gal or UDP-GlcNAc-dependent glycosyltransferase, putative | 1 |

| | | |
|---|---|---|
| Tb927.4.4250 | UDP-Gal or UDP-GlcNAc-dependent glycosyltransferase, putative | 1 |
| Tb927.4.4270 | UDP-Gal or UDP-GlcNAc-dependent glycosyltransferase, putative | 1 |
| Tb927.4.4290 | UDP-Gal or UDP-GlcNAc-dependent glycosyltransferase, putative | 1 |
| Tb927.4.5240 | UDP-Gal or UDP-GlcNAc-dependent glycosyltransferase, putative | 1 |
| Tb927.4.5250 | UDP-Gal or UDP-GlcNAc-dependent glycosyltransferase, putative | 1 |
| Tb927.4.5260 | UDP-Gal or UDP-GlcNAc-dependent glycosyltransferase, putative | 1 |
| Tb927.4.5270 | UDP-Gal or UDP-GlcNAc-dependent glycosyltransferase, putative | 1 |
| Tb927.4.5280 | UDP-Gal or UDP-GlcNAc-dependent glycosyltransferase, putative | 1 |
| Tb927.4.5290 | UDP-Gal or UDP-GlcNAc-dependent glycosyltransferase, putative | 1 |
| Tb927.5.2760 | UDP-Gal or UDP-GlcNAc-dependent glycosyltransferase, putative | 1 |
| Tb927.8.7140 | UDP-Gal or UDP-GlcNAc-dependent glycosyltransferase, putative | 1 |
| Tb927.8.7150 | UDP-Gal or UDP-GlcNAc-dependent glycosyltransferase, putative | 1 |
| Tb927.3.4630 | UDP-glucose:glycoprotein glucosyltransferase, putative | 1 |
| Tb11.02.1230 | B5R NADH-cytochrome b5 reductase, putative | 1 |
| Tb09.211.4110 | CPR NADPH--cytochrome p450 reductase, putative | 1 |
| Tb11.01.0170 | CPR NADPH--cytochrome P450 reductase, putative | 1 |
| Tb11.02.5420 | CPR NADPH--cytochrome p450 reductase, putative | 1 |
| Tb10.6k15.3510 | CRAM cysteine-rich, acidic integral membrane protein precursor | 1 |
| Tb927.8.4890 | endoplasmic reticulum oxidoreductin, putative,pol-associated gene 1 | 1 |
| Tb10.6k15.0020 | EP1 EP1 procyclin | 1 |
| Tb10.6k15.0030 | EP2 EP2 procyclin | 1 |
| Tb927.6.480 | EP3-2 procyclin,PARP A-beta,surface protein EP3-2,surface protein EP3-2 procyclin precursor, | 1 |
| Tb927.6.480 | EP3-2 procyclin,PARP A-beta,surface protein EP3-2,surface protein EP3-2 procyclin precursor, | 1 |
| Tb927.6.520 | EP3-2 procyclin,PARP A-beta,surface protein EP3-3 procyclin precursor, | 1 |
| Tb927.6.450 | EP3-2 procyclin,PARP,procyclin PARP A,procyclin B1-alpha,procyclic acidic repetitive protein A.beta, | 1 |
| Tb927.6.510 | GPEET2 procyclin precursor,PARP A-alpha,procyclin A-alpha,procyclic form specific polypeptide A-alpha precursor | 1 |
| Tb11.01.6210 | procyclin-associated gene 2-like protein, putative | 1 |
| Tb10.6k15.0920 | expression site-associated gene (ESAG) protein, putative,expression site-associated gene 3 (ESAG3) protein, putative | 1 |
| Tb927.5.4600 | expression site-associated gene (ESAG) protein, putative,expression site-associated gene 3 (ESAG3) protein, putative | 1 |
| Tb09.244.2120 | expression site-associated gene (ESAG) protein, putative,expression site-associated gene 5 (ESAG5) protein, putative | 1 |
| Tb927.7.6860 | expression site-associated gene (ESAG) protein, putative,expression site-associated gene 5 (ESAG5) protein, putative | 1 |
| Tb09.160.5380 | expression site-associated gene 11 (ESAG11) protein, putative | 1 |
| Tb09.v1.0310 | expression site-associated gene 11 (ESAG11) protein, putative | 1 |
| Tb11.01.3590 | FG-GAP repeat protein, putative,intergrin alpha chain protein, putative | 1 |

| | | |
|---|---|---|
| Tb11.01.7770 | FG-GAP repeat protein, putative,intergrin alpha chain protein, putative | 1 |
| Tb927.7.6460 | FG-GAP repeat protein, putative,intergrin alpha chain protein, putative | 1 |
| Tb11.01.3150 | gamma-tubulin complex subunit, putative,spindle pole body component alp6, putative | 1 |
| Tb927.4.5010 | calreticulin, putative | 1 |
| Tb927.8.7410 | calreticulin, putative | 1 |
| Tb11.01.0290 | carbonic anhydrase-like protein | 1 |
| Tb11.18.0006 | CAT carnitine O-acetyltransferase, putative | 1 |
| Tb927.5.940 | NADH-dependent fumarate reductase, putative | 1 |
| Tb09.160.3820 | nucleolar RNA binding protein, putative | 1 |
| Tb927.6.4600 | pre-mRNA splicing factor ATP-dependent RNA helicase, putative,ATP-dependent RNA helicase, putative | 1 |
| Tb927.6.4600 | pre-mRNA splicing factor ATP-dependent RNA helicase, putative,ATP-dependent RNA helicase, putative | 1 |
| Tb10.100.0190 | pumillio RNA binding protein, putative | 1 |
| Tb11.02.5210 | RNA binding protein, putative | 1 |
| Tb11.01.6260 | RNA helicase, putative,DEAD/DEAH box helicase, putative | 1 |
| Tb927.7.4670 | RRP4 ribosomal RNA processing protein 4,exosome complex exonuclease | 1 |
| Tb10.70.1690 | 40S ribosomal protein S10, putative | 1 |
| Tb927.4.1790 | ribosomal protein L3, putative | 1 |
| Tb927.8.1870 | tGLP1 Golgi/lysosome glycoprotein 1 | 1 |
| Tb10.389.1500 | short-chain dehydrogenase, putative | 1 |
| Tb10.6k15.3880 | short-chain dehydrogenase, putative | 1 |
| Tb10.70.3240 | short-chain dehydrogenase, putative | 1 |
| Tb11.v4.0058 | variant surface glycoprotein (VSG), putative | 1 |
| Tb11.v4.0065 | variant surface glycoprotein (VSG), putative | 1 |
| Tb927.4.5460 | variant surface glycoprotein (VSG), putative | 1 |
| Tb09.244.1000 | variant surface glycoprotein (VSG, atypical), putative | 1 |
| Tb927.3.180 | variant surface glycoprotein (VSG, atypical), putative | 1 |
| Tb927.3.370 | variant surface glycoprotein (VSG, atypical), putative | 1 |
| Tb927.4.5400 | variant surface glycoprotein (VSG, atypical), putative | 1 |
| Tb927.7.6540 | variant surface glycoprotein (VSG, atypical), putative | 1 |
| Tb927.8.170 | variant surface glycoprotein (VSG, atypical), putative | 1 |
| Tb927.6.4960 | zinc finger-domain protein, putative | 1 |
| Tb927.6.4960 | zinc finger-domain protein, putative | 1 |
| Tb09.142.0430 | hypothetical protein | 1 |
| Tb09.244.2390 | hypothetical protein | 1 |

| | | |
|---|---|---|
| Tb10.26.0190 | hypothetical protein | 1 |
| Tb10.389.1100 | hypothetical protein | 1 |
| Tb10.6k15.0940 | hypothetical protein | 1 |
| Tb10.70.3250 | hypothetical protein | 1 |
| Tb10.70.5370 | hypothetical protein | 1 |
| Tb927.1.10 | hypothetical protein | 1 |
| Tb927.2.1600 | hypothetical protein | 1 |
| Tb927.2.2490 | hypothetical protein | 1 |
| Tb927.2.680 | hypothetical protein | 1 |
| Tb927.3.2800 | hypothetical protein | 1 |
| Tb927.3.5670 | hypothetical protein | 1 |
| Tb927.3.600 | hypothetical protein | 1 |
| Tb927.4.4770 | hypothetical protein | 1 |
| Tb927.5.1740 | hypothetical protein | 1 |
| Tb927.5.310 | hypothetical protein | 1 |
| Tb927.5.410 | hypothetical protein | 1 |
| Tb927.5.420 | hypothetical protein | 1 |
| Tb927.5.430 | hypothetical protein | 1 |
| Tb927.5.620 | hypothetical protein | 1 |
| Tb927.6.120 | hypothetical protein | 1 |
| Tb927.6.420 | hypothetical protein | 1 |
| Tb927.7.4690 | hypothetical protein | 1 |
| Tb927.7.6100 | hypothetical protein | 1 |
| Tb927.8.1760 | hypothetical protein | 1 |
| Tb09.160.1520 | hypothetical protein, conserved | 1 |
| Tb09.160.2060 | hypothetical protein, conserved | 1 |
| Tb09.211.0020 | hypothetical protein, conserved | 1 |
| Tb09.211.0990 | hypothetical protein, conserved | 1 |
| Tb09.211.1290 | hypothetical protein, conserved | 1 |
| Tb09.211.3530 | hypothetical protein, conserved | 1 |
| Tb09.211.3660 | hypothetical protein, conserved | 1 |
| Tb09.211.4630 | hypothetical protein, conserved | 1 |
| Tb09.211.4770 | hypothetical protein, conserved | 1 |
| Tb09.244.2830 | hypothetical protein, conserved | 1 |

| | | |
|---|---|---|
| Tb09.v1.0510 | hypothetical protein, conserved | 1 |
| Tb09.v1.0540 | hypothetical protein, conserved | 1 |
| Tb10.100.0150 | hypothetical protein, conserved | 1 |
| Tb10.389.0030 | hypothetical protein, conserved | 1 |
| Tb10.389.0045 | hypothetical protein, conserved | 1 |
| Tb10.389.1250 | hypothetical protein, conserved | 1 |
| Tb10.389.1260 | hypothetical protein, conserved | 1 |
| Tb10.406.0030 | hypothetical protein, conserved | 1 |
| Tb10.61.2720 | hypothetical protein, conserved | 1 |
| Tb10.61.3170 | hypothetical protein, conserved | 1 |
| Tb10.6k15.0300 | hypothetical protein, conserved | 1 |
| Tb10.6k15.1130 | hypothetical protein, conserved | 1 |
| Tb10.6k15.1210 | hypothetical protein, conserved | 1 |
| Tb10.70.0640 | hypothetical protein, conserved | 1 |
| Tb10.70.0770 | hypothetical protein, conserved | 1 |
| Tb10.70.2120 | hypothetical protein, conserved | 1 |
| Tb10.70.2540 | hypothetical protein, conserved | 1 |
| Tb10.70.4110 | hypothetical protein, conserved | 1 |
| Tb10.70.4610 | hypothetical protein, conserved | 1 |
| Tb10.70.5180 | hypothetical protein, conserved | 1 |
| Tb10.70.5690 | hypothetical protein, conserved | 1 |
| Tb10.70.7460 | hypothetical protein, conserved | 1 |
| Tb11.01.1000 | hypothetical protein, conserved | 1 |
| Tb11.01.3160 | hypothetical protein, conserved | 1 |
| Tb11.01.4110 | hypothetical protein, conserved | 1 |
| Tb11.01.4740 | hypothetical protein, conserved | 1 |
| Tb11.01.5120 | hypothetical protein, conserved | 1 |
| Tb11.01.5160 | hypothetical protein, conserved | 1 |
| Tb11.01.5190 | hypothetical protein, conserved | 1 |
| Tb11.01.5760 | hypothetical protein, conserved | 1 |
| Tb11.01.5850 | hypothetical protein, conserved | 1 |
| Tb11.01.6570 | hypothetical protein, conserved | 1 |
| Tb11.01.6860 | hypothetical protein, conserved | 1 |
| Tb11.01.8070 | hypothetical protein, conserved | 1 |

| | | |
|---|---|---|
| Tb11.02.0060 | hypothetical protein, conserved | 1 |
| Tb11.02.2450 | hypothetical protein, conserved | 1 |
| Tb11.02.2460 | hypothetical protein, conserved | 1 |
| Tb11.02.2760 | hypothetical protein, conserved | 1 |
| Tb11.02.4205 | hypothetical protein, conserved | 1 |
| Tb11.02.4900 | hypothetical protein, conserved | 1 |
| Tb11.02.5165 | hypothetical protein, conserved | 1 |
| Tb11.02.5370 | hypothetical protein, conserved | 1 |
| Tb11.03.0370 | hypothetical protein, conserved | 1 |
| Tb927.1.110 | hypothetical protein, conserved | 1 |
| Tb927.1.1140 | hypothetical protein, conserved | 1 |
| Tb927.1.1310 | hypothetical protein, conserved | 1 |
| Tb927.1.3810 | hypothetical protein, conserved | 1 |
| Tb927.1.3840 | hypothetical protein, conserved | 1 |
| Tb927.1.4160 | hypothetical protein, conserved | 1 |
| Tb927.1.4370 | hypothetical protein, conserved | 1 |
| Tb927.1.4380 | hypothetical protein, conserved | 1 |
| Tb927.1.860 | hypothetical protein, conserved | 1 |
| Tb927.2.1760 | hypothetical protein, conserved | 1 |
| Tb927.2.2380 | hypothetical protein, conserved | 1 |
| Tb927.2.3400 | hypothetical protein, conserved | 1 |
| Tb927.2.4150 | hypothetical protein, conserved | 1 |
| Tb927.2.5440 | hypothetical protein, conserved | 1 |
| Tb927.2.5970 | hypothetical protein, conserved | 1 |
| Tb927.3.1870 | hypothetical protein, conserved | 1 |
| Tb927.3.2120 | hypothetical protein, conserved | 1 |
| Tb927.3.2360 | hypothetical protein, conserved | 1 |
| Tb927.3.2820 | hypothetical protein, conserved | 1 |
| Tb927.3.2870 | hypothetical protein, conserved | 1 |
| Tb927.3.3130 | hypothetical protein, conserved | 1 |
| Tb927.3.3350 | hypothetical protein, conserved | 1 |
| Tb927.3.5170 | hypothetical protein, conserved | 1 |
| Tb927.3.640 | hypothetical protein, conserved | 1 |
| Tb927.3.840 | hypothetical protein, conserved | 1 |

| | | |
|---|---|---|
| Tb927.4.1190 | hypothetical protein, conserved | 1 |
| Tb927.4.1390 | hypothetical protein, conserved | 1 |
| Tb927.4.1600 | hypothetical protein, conserved | 1 |
| Tb927.4.1970 | hypothetical protein, conserved | 1 |
| Tb927.4.3040 | hypothetical protein, conserved | 1 |
| Tb927.4.3260 | hypothetical protein, conserved | 1 |
| Tb927.4.4350 | hypothetical protein, conserved | 1 |
| Tb927.4.4790 | hypothetical protein, conserved | 1 |
| Tb927.4.4920 | hypothetical protein, conserved | 1 |
| Tb927.4.510 | hypothetical protein, conserved | 1 |
| Tb927.4.540 | hypothetical protein, conserved | 1 |
| Tb927.4.590 | hypothetical protein, conserved | 1 |
| Tb927.4.700 | hypothetical protein, conserved | 1 |
| Tb927.4.720 | hypothetical protein, conserved | 1 |
| Tb927.5.1160 | hypothetical protein, conserved | 1 |
| Tb927.5.1630 | hypothetical protein, conserved | 1 |
| Tb927.5.1760 | hypothetical protein, conserved | 1 |
| Tb927.5.1930 | hypothetical protein, conserved | 1 |
| Tb927.5.1990 | hypothetical protein, conserved | 1 |
| Tb927.5.2350 | hypothetical protein, conserved | 1 |
| Tb927.5.2750 | hypothetical protein, conserved | 1 |
| Tb927.5.4060 | hypothetical protein, conserved | 1 |
| Tb927.5.920 | hypothetical protein, conserved | 1 |
| Tb927.6.1850 | hypothetical protein, conserved | 1 |
| Tb927.6.2560 | hypothetical protein, conserved | 1 |
| Tb927.6.4260 | hypothetical protein, conserved | 1 |
| Tb927.6.4320 | hypothetical protein, conserved | 1 |
| Tb927.6.4500 | hypothetical protein, conserved | 1 |
| Tb927.6.650 | hypothetical protein, conserved | 1 |
| Tb927.6.880 | hypothetical protein, conserved | 1 |
| Tb927.7.1290 | hypothetical protein, conserved | 1 |
| Tb927.7.1480 | hypothetical protein, conserved | 1 |
| Tb927.7.3600 | hypothetical protein, conserved | 1 |
| Tb927.7.3870 | hypothetical protein, conserved | 1 |

| | | |
|---|---|---|
| Tb927.7.4230 | hypothetical protein, conserved | 1 |
| Tb927.7.4260 | hypothetical protein, conserved | 1 |
| Tb927.7.4270 | hypothetical protein, conserved | 1 |
| Tb927.7.4660 | hypothetical protein, conserved | 1 |
| Tb927.7.4880 | hypothetical protein, conserved | 1 |
| Tb927.7.4890 | hypothetical protein, conserved | 1 |
| Tb927.7.5470 | hypothetical protein, conserved | 1 |
| Tb927.7.5700 | hypothetical protein, conserved | 1 |
| Tb927.7.5780 | hypothetical protein, conserved | 1 |
| Tb927.7.6550 | hypothetical protein, conserved | 1 |
| Tb927.7.6570 | hypothetical protein, conserved | 1 |
| Tb927.7.6590 | hypothetical protein, conserved | 1 |
| Tb927.7.6920 | hypothetical protein, conserved | 1 |
| Tb927.7.900 | hypothetical protein, conserved | 1 |
| Tb927.8.1580 | hypothetical protein, conserved | 1 |
| Tb927.8.2230 | hypothetical protein, conserved | 1 |
| Tb927.8.2510 | hypothetical protein, conserved | 1 |
| Tb927.8.3050 | hypothetical protein, conserved | 1 |
| Tb927.8.3540 | hypothetical protein, conserved | 1 |
| Tb927.8.5080 | hypothetical protein, conserved | 1 |
| Tb927.8.5150 | hypothetical protein, conserved | 1 |
| Tb927.8.5420 | hypothetical protein, conserved | 1 |
| Tb927.8.560 | hypothetical protein, conserved | 1 |
| Tb927.8.580 | hypothetical protein, conserved | 1 |
| Tb927.8.6020 | hypothetical protein, conserved | 1 |
| Tb927.8.6310 | hypothetical protein, conserved | 1 |
| Tb927.8.6380 | hypothetical protein, conserved | 1 |
| Tb927.8.6590 | hypothetical protein, conserved | 1 |
| Tb927.8.6960 | hypothetical protein, conserved | 1 |
| Tb927.8.7280 | hypothetical protein, conserved | 1 |
| Tb927.8.7500 | hypothetical protein, conserved | 1 |
| Tb927.8.7710 | hypothetical protein, conserved | 1 |
| Tb927.8.7720 | hypothetical protein, conserved | 1 |
| Tb927.8.8030 | hypothetical protein, conserved | 1 |

| | | |
|---|---|---|
| Tb10.05.0040 | hypothetical protein, conserved,hypothetical protein | 1 |
| Tb09.211.0210 | hypothetical protein, conserved,tyrosine phosphatase, putative | 1 |
| Tb09.160.1050 | hypothetical protein, unlikely | 1 |
| Tb09.160.1640 | hypothetical protein, unlikely | 1 |
| Tb09.160.1800 | hypothetical protein, unlikely | 1 |
| Tb09.160.2140 | hypothetical protein, unlikely | 1 |
| Tb09.160.2190 | hypothetical protein, unlikely | 1 |
| Tb09.160.2270 | hypothetical protein, unlikely | 1 |
| Tb09.160.2340 | hypothetical protein, unlikely | 1 |
| Tb09.160.2680 | hypothetical protein, unlikely | 1 |
| Tb09.160.2750 | hypothetical protein, unlikely | 1 |
| Tb09.160.3340 | hypothetical protein, unlikely | 1 |
| Tb09.160.3680 | hypothetical protein, unlikely | 1 |
| Tb09.160.3810 | hypothetical protein, unlikely | 1 |
| Tb09.160.3870 | hypothetical protein, unlikely | 1 |
| Tb09.160.4070 | hypothetical protein, unlikely | 1 |
| Tb09.160.4230 | hypothetical protein, unlikely | 1 |
| Tb09.160.4370 | hypothetical protein, unlikely | 1 |
| Tb09.160.4390 | hypothetical protein, unlikely | 1 |
| Tb09.160.4810 | hypothetical protein, unlikely | 1 |
| Tb09.160.4990 | hypothetical protein, unlikely | 1 |
| Tb09.160.5610 | hypothetical protein, unlikely | 1 |
| Tb09.160.5630 | hypothetical protein, unlikely | 1 |
| Tb09.211.0550 | hypothetical protein, unlikely | 1 |
| Tb09.211.1100 | hypothetical protein, unlikely | 1 |
| Tb09.211.1450 | hypothetical protein, unlikely | 1 |
| Tb09.211.1460 | hypothetical protein, unlikely | 1 |
| Tb09.211.1730 | hypothetical protein, unlikely | 1 |
| Tb09.211.1990 | hypothetical protein, unlikely | 1 |
| Tb09.211.2840 | hypothetical protein, unlikely | 1 |
| Tb09.211.3710 | hypothetical protein, unlikely | 1 |
| Tb09.211.4050 | hypothetical protein, unlikely | 1 |
| Tb09.211.4250 | hypothetical protein, unlikely | 1 |
| Tb09.211.4660 | hypothetical protein, unlikely | 1 |

| | | |
|---|---|---|
| Tb09.244.0510 | hypothetical protein, unlikely | 1 |
| Tb09.244.2110 | hypothetical protein, unlikely | 1 |
| Tb09.v1.0100 | hypothetical protein, unlikely | 1 |
| Tb09.v1.0210 | hypothetical protein, unlikely | 1 |
| Tb09.v1.0340 | hypothetical protein, unlikely | 1 |
| Tb09.v1.0570 | hypothetical protein, unlikely | 1 |
| Tb09.v1.0760 | hypothetical protein, unlikely | 1 |
| Tb09.v2.0050 | hypothetical protein, unlikely | 1 |
| Tb10.61.0790 | hypothetical protein, unlikely | 1 |
| Tb11.02.1960 | hypothetical protein, unlikely | 1 |
| Tb11.02.2990 | hypothetical protein, unlikely | 1 |
| Tb927.1.1320 | hypothetical protein, unlikely | 1 |
| Tb927.1.1460 | hypothetical protein, unlikely | 1 |
| Tb927.1.1590 | hypothetical protein, unlikely | 1 |
| Tb927.1.2610 | hypothetical protein, unlikely | 1 |
| Tb927.1.2660 | hypothetical protein, unlikely | 1 |
| Tb927.1.3420 | hypothetical protein, unlikely | 1 |
| Tb927.1.3900 | hypothetical protein, unlikely | 1 |
| Tb927.1.3930 | hypothetical protein, unlikely | 1 |
| Tb927.1.4070 | hypothetical protein, unlikely | 1 |
| Tb927.1.4430 | hypothetical protein, unlikely | 1 |
| Tb927.1.4530 | hypothetical protein, unlikely | 1 |
| Tb927.1.4610 | hypothetical protein, unlikely | 1 |
| Tb927.1.4640 | hypothetical protein, unlikely | 1 |
| Tb927.1.4770 | hypothetical protein, unlikely | 1 |
| Tb927.1.5040 | hypothetical protein, unlikely | 1 |
| Tb927.1.5130 | hypothetical protein, unlikely | 1 |
| Tb927.1.660 | hypothetical protein, unlikely | 1 |
| Tb927.5.390 | 75 kDa invariant surface glycoprotein, putative | 2 |
| Tb927.5.640 | trans-sialidase, putative,neuraminidase, putative | 2 |
| Tb927.5.610 | acidic phosphatase, putative | 2 |
| Tb11.01.4701 | MBAP1 membrane-bound acid phosphatase 1 precursor | 2 |
| Tb11.01.3610 | membrane-bound acid phosphatase, putative | 2 |
| Tb927.4.2500 | protein kinase, putative,eukaryotic translation initiation factor 2-alpha kinase precursor, putative | 2 |

| | | |
|---|---|---|
| Tb11.01.5310 | receptor-type adenylate cyclase GRESAG 4, putative | 2 |
| Tb927.4.3750 | receptor-type adenylate cyclase GRESAG 4, putative | 2 |
| Tb927.7.6060 | receptor-type adenylate cyclase GRESAG 4, putative | 2 |
| Tb927.7.7520 | receptor-type adenylate cyclase GRESAG 4, putative | 2 |
| Tb927.7.7530 | receptor-type adenylate cyclase GRESAG 4, putative | 2 |
| Tb927.8.7890 | receptor-type adenylate cyclase GRESAG 4, putative | 2 |
| Tb927.8.7900 | receptor-type adenylate cyclase GRESAG 4, putative | 2 |
| Tb927.8.7920 | receptor-type adenylate cyclase GRESAG 4, putative | 2 |
| Tb927.8.7930 | receptor-type adenylate cyclase GRESAG 4, putative | 2 |
| Tb927.8.7940 | receptor-type adenylate cyclase GRESAG 4, putative | 2 |
| Tb927.5.1020 | disulfide isomerase, putative | 2 |
| Tb927.8.1610 | major surface protease gp63, putative,GP63, putative,metallopeptidase, putative | 2 |
| Tb927.4.1920 | glycosylphosphatidylinositol (GPI) anchor, putative | 2 |
| Tb09.211.3650 | phospholipase A2-like protein, putative | 2 |
| Tb11.02.0780 | squalene monooxygenase, putative | 2 |
| Tb10.389.0250 | glycosyltransferase, putative | 2 |
| Tb927.6.1960 | glycosyltransferase family 28 protein, putative | 2 |
| Tb09.244.2760 | cytosolic coat protein, putative | 2 |
| Tb11.01.6880 | cytosolic coat protein, putative | 2 |
| Tb10.6k15.0910 | expression site-associated gene (ESAG) protein, putative,expression site-associated gene 3 (ESAG3) protein, putative | 2 |
| Tb11.55.0027 | expression site-associated gene (ESAG) protein, putative,expression site-associated gene 3 (ESAG3) protein, putative | 2 |
| Tb11.55.0028 | expression site-associated gene (ESAG) protein, putative,expression site-associated gene 3 (ESAG3) protein, putative | 2 |
| Tb10.6k15.0900 | expression site-associated gene 3 (ESAG3)-like protein | 2 |
| Tb09.244.2380 | expression site-associated gene 4 (ESAG4) protein, putative,receptor-type adenylate cyclase, putative | 2 |
| Tb927.8.4010 | fla1 flagellum-adhesion glycoprotein | 2 |
| Tb927.8.4060 | flagellum-adhesion glycoprotein, putative | 2 |
| Tb927.8.4110 | flagellum-adhesion glycoprotein, putative | 2 |
| Tb10.70.0120 | COP-coated vesicle membrane protein erv25 precursor, putative,ER--golgi transport protein erv25 precursor, putative | 2 |
| Tb927.5.1810 | lysosomal/endosomal membrane protein p67,lysosomal membrane glycoprotein | 2 |
| Tb927.5.1830 | lysosomal/endosomal membrane protein p67,lysosomal membrane glycoprotein | 2 |
| Tb11.02.2950 | ATPase subunit 9, putative | 2 |
| Tb927.7.1470 | ATPase subunit 9, putative | 2 |
| Tb927.8.3330 | mitochondrial carrier protein, putative | 2 |
| Tb11.01.1780 | short-chain dehydrogenase, putative | 2 |

| | | |
|---|---|---|
| Tb927.5.1210 | short-chain dehydrogenase, putative | 2 |
| Tb09.211.4950 | hypothetical protein | 2 |
| Tb10.61.2500 | hypothetical protein | 2 |
| Tb10.6k15.1610 | hypothetical protein | 2 |
| Tb10.70.4020 | hypothetical protein | 2 |
| Tb11.01.7870 | hypothetical protein | 2 |
| Tb11.02.1850 | hypothetical protein | 2 |
| Tb11.v4.0002 | hypothetical protein | 2 |
| Tb927.1.2310 | hypothetical protein | 2 |
| Tb927.3.1650 | hypothetical protein | 2 |
| Tb927.3.3700 | hypothetical protein | 2 |
| Tb927.4.1010 | hypothetical protein | 2 |
| Tb927.7.470 | hypothetical protein | 2 |
| Tb927.7.7310 | hypothetical protein | 2 |
| Tb927.8.3670 | hypothetical protein | 2 |
| Tb09.142.0440 | hypothetical protein, conserved | 2 |
| Tb09.160.4690 | hypothetical protein, conserved | 2 |
| Tb09.211.1890 | hypothetical protein, conserved | 2 |
| Tb09.211.4155 | hypothetical protein, conserved | 2 |
| Tb09.211.4680 | hypothetical protein, conserved | 2 |
| Tb09.244.2770 | hypothetical protein, conserved | 2 |
| Tb09.v1.0650 | hypothetical protein, conserved | 2 |
| Tb10.100.0200 | hypothetical protein, conserved | 2 |
| Tb10.26.0900 | hypothetical protein, conserved | 2 |
| Tb10.389.0370 | hypothetical protein, conserved | 2 |
| Tb10.61.1115 | hypothetical protein, conserved | 2 |
| Tb10.6k15.1800 | hypothetical protein, conserved | 2 |
| Tb10.6k15.2390 | hypothetical protein, conserved | 2 |
| Tb10.70.3125 | hypothetical protein, conserved | 2 |
| Tb10.70.3185 | hypothetical protein, conserved | 2 |
| Tb10.70.4150 | hypothetical protein, conserved | 2 |
| Tb10.70.7510 | hypothetical protein, conserved | 2 |
| Tb11.01.0415 | hypothetical protein, conserved | 2 |
| Tb11.01.2640 | hypothetical protein, conserved | 2 |

| | | |
|---|---|---|
| Tb11.01.3790 | hypothetical protein, conserved | 2 |
| Tb11.01.5280 | hypothetical protein, conserved | 2 |
| Tb11.01.7540 | hypothetical protein, conserved | 2 |
| Tb11.01.8715 | hypothetical protein, conserved | 2 |
| Tb11.02.0710 | hypothetical protein, conserved | 2 |
| Tb11.02.0940 | hypothetical protein, conserved | 2 |
| Tb11.02.1760 | hypothetical protein, conserved | 2 |
| Tb11.02.3760 | hypothetical protein, conserved | 2 |
| Tb11.47.0019 | hypothetical protein, conserved | 2 |
| Tb927.1.2985 | hypothetical protein, conserved | 2 |
| Tb927.1.3410 | hypothetical protein, conserved | 2 |
| Tb927.1.840 | hypothetical protein, conserved | 2 |
| Tb927.2.1700 | hypothetical protein, conserved | 2 |
| Tb927.2.2500 | hypothetical protein, conserved | 2 |
| Tb927.2.4090 | hypothetical protein, conserved | 2 |
| Tb927.2.4760 | hypothetical protein, conserved | 2 |
| Tb927.2.4920 | hypothetical protein, conserved | 2 |
| Tb927.2.4920 | hypothetical protein, conserved | 2 |
| Tb927.2.5290 | hypothetical protein, conserved | 2 |
| Tb927.2.5300 | hypothetical protein, conserved | 2 |
| Tb927.2.5310 | hypothetical protein, conserved | 2 |
| Tb927.2.5320 | hypothetical protein, conserved | 2 |
| Tb927.2.5330 | hypothetical protein, conserved | 2 |
| Tb927.2.5340 | hypothetical protein, conserved | 2 |
| Tb927.2.5350 | hypothetical protein, conserved | 2 |
| Tb927.2.5360 | hypothetical protein, conserved | 2 |
| Tb927.4.1120 | hypothetical protein, conserved | 2 |
| Tb927.4.2160 | hypothetical protein, conserved | 2 |
| Tb927.4.2170 | hypothetical protein, conserved | 2 |
| Tb927.4.2320 | hypothetical protein, conserved | 2 |
| Tb927.4.780 | hypothetical protein, conserved | 2 |
| Tb927.4.830 | hypothetical protein, conserved | 2 |
| Tb927.5.2580 | hypothetical protein, conserved | 2 |
| Tb927.5.3700 | hypothetical protein, conserved | 2 |

| | | |
|---|---|---|
| Tb927.5.4570 | hypothetical protein, conserved | 2 |
| Tb927.5.4580 | hypothetical protein, conserved | 2 |
| Tb927.6.1740 | hypothetical protein, conserved | 2 |
| Tb927.6.2260 | hypothetical protein, conserved | 2 |
| Tb927.6.2320 | hypothetical protein, conserved | 2 |
| Tb927.6.370 | hypothetical protein, conserved | 2 |
| Tb927.6.3700 | hypothetical protein, conserved | 2 |
| Tb927.6.380 | hypothetical protein, conserved | 2 |
| Tb927.7.1700 | hypothetical protein, conserved | 2 |
| Tb927.7.2190 | hypothetical protein, conserved | 2 |
| Tb927.7.2950 | hypothetical protein, conserved | 2 |
| Tb927.7.4280 | hypothetical protein, conserved | 2 |
| Tb927.7.5400 | hypothetical protein, conserved | 2 |
| Tb927.7.5420 | hypothetical protein, conserved | 2 |
| Tb927.7.5710 | hypothetical protein, conserved | 2 |
| Tb927.7.6760 | hypothetical protein, conserved | 2 |
| Tb927.8.1370 | hypothetical protein, conserved | 2 |
| Tb927.8.2570 | hypothetical protein, conserved | 2 |
| Tb927.8.3320 | hypothetical protein, conserved | 2 |
| Tb927.8.3390 | hypothetical protein, conserved | 2 |
| Tb927.8.3720 | hypothetical protein, conserved | 2 |
| Tb927.8.3880 | hypothetical protein, conserved | 2 |
| Tb927.8.3890 | hypothetical protein, conserved | 2 |
| Tb927.8.3900 | hypothetical protein, conserved | 2 |
| Tb927.8.4000 | hypothetical protein, conserved | 2 |
| Tb927.8.4600 | hypothetical protein, conserved | 2 |
| Tb927.8.6800 | hypothetical protein, conserved | 2 |
| Tb09.160.0300 | hypothetical protein, unlikely | 2 |
| Tb09.160.0310 | hypothetical protein, unlikely | 2 |
| Tb09.160.0980 | hypothetical protein, unlikely | 2 |
| Tb09.160.1230 | hypothetical protein, unlikely | 2 |
| Tb09.160.1430 | hypothetical protein, unlikely | 2 |
| Tb09.160.1510 | hypothetical protein, unlikely | 2 |
| Tb09.160.1750 | hypothetical protein, unlikely | 2 |

| | | |
|---|---|---|
| Tb09.160.3170 | hypothetical protein, unlikely | 2 |
| Tb09.160.3180 | hypothetical protein, unlikely | 2 |
| Tb09.160.3600 | hypothetical protein, unlikely | 2 |
| Tb09.160.3610 | hypothetical protein, unlikely | 2 |
| Tb09.160.3700 | hypothetical protein, unlikely | 2 |
| Tb09.160.3770 | hypothetical protein, unlikely | 2 |
| Tb09.160.4100 | hypothetical protein, unlikely | 2 |
| Tb09.160.5140 | hypothetical protein, unlikely | 2 |
| Tb09.211.1060 | hypothetical protein, unlikely | 2 |
| Tb09.211.1120 | hypothetical protein, unlikely | 2 |
| Tb09.211.1840 | hypothetical protein, unlikely | 2 |
| Tb09.211.1860 | hypothetical protein, unlikely | 2 |
| Tb09.211.1920 | hypothetical protein, unlikely | 2 |
| Tb09.211.4340 | hypothetical protein, unlikely | 2 |
| Tb09.211.4410 | hypothetical protein, unlikely | 2 |
| Tb09.v1.0850 | hypothetical protein, unlikely | 2 |
| Tb10.61.0710 | hypothetical protein, unlikely | 2 |
| Tb927.1.1280 | hypothetical protein, unlikely | 2 |
| Tb927.1.1360 | hypothetical protein, unlikely | 2 |
| Tb927.1.2590 | hypothetical protein, unlikely | 2 |
| Tb927.1.3440 | hypothetical protein, unlikely | 2 |
| Tb927.1.3480 | hypothetical protein, unlikely | 2 |
| Tb927.1.4290 | hypothetical protein, unlikely | 2 |
| Tb927.1.4670 | hypothetical protein, unlikely | 2 |
| Tb927.1.5310 | hypothetical protein, unlikely | 2 |
| Tb927.5.3150 | protein kinase , putative | 3 |
| Tb927.3.4820 | acyltransferase, putative | 3 |
| Tb927.5.860 | SelT selenoprotein, putative | 3 |
| Tb927.8.6010 | hypothetical predicted multi-pass transmembrane protein | 3 |
| Tb09.160.5510 | hypothetical protein | 3 |
| Tb10.6k15.0730 | hypothetical protein | 3 |
| Tb10.70.3310 | hypothetical protein | 3 |
| Tb11.02.2180 | hypothetical protein | 3 |
| Tb11.02.4690 | hypothetical protein | 3 |

| | | |
|---|---|---|
| Tb927.6.1150 | hypothetical protein | 3 |
| Tb927.6.1300 | hypothetical protein | 3 |
| Tb927.6.4110 | hypothetical protein | 3 |
| Tb927.7.3690 | hypothetical protein | 3 |
| Tb927.8.2320 | hypothetical protein | 3 |
| Tb927.8.3760 | hypothetical protein | 3 |
| Tb927.8.4300 | hypothetical protein | 3 |
| Tb09.160.5040 | hypothetical protein, conserved | 3 |
| Tb09.160.5310 | hypothetical protein, conserved | 3 |
| Tb09.211.0450 | hypothetical protein, conserved | 3 |
| Tb09.211.1360 | hypothetical protein, conserved | 3 |
| Tb09.v1.0770 | hypothetical protein, conserved | 3 |
| Tb10.389.0950 | hypothetical protein, conserved | 3 |
| Tb10.6k15.1780 | hypothetical protein, conserved | 3 |
| Tb10.70.1640 | hypothetical protein, conserved | 3 |
| Tb10.70.2450 | hypothetical protein, conserved | 3 |
| Tb10.70.2690 | hypothetical protein, conserved | 3 |
| Tb11.01.1340 | hypothetical protein, conserved | 3 |
| Tb11.02.2730 | hypothetical protein, conserved | 3 |
| Tb11.02.3240 | hypothetical protein, conserved | 3 |
| Tb927.1.4550 | hypothetical protein, conserved | 3 |
| Tb927.1.4570 | hypothetical protein, conserved | 3 |
| Tb927.1.4590 | hypothetical protein, conserved | 3 |
| Tb927.1.4620 | hypothetical protein, conserved | 3 |
| Tb927.2.4840 | hypothetical protein, conserved | 3 |
| Tb927.2.6070 | hypothetical protein, conserved | 3 |
| Tb927.3.5430 | hypothetical protein, conserved | 3 |
| Tb927.4.2420 | hypothetical protein, conserved | 3 |
| Tb927.4.3710 | hypothetical protein, conserved | 3 |
| Tb927.4.390 | hypothetical protein, conserved | 3 |
| Tb927.4.5080 | hypothetical protein, conserved | 3 |
| Tb927.6.3940 | hypothetical protein, conserved | 3 |
| Tb927.6.4620 | hypothetical protein, conserved | 3 |
| Tb927.6.4620 | hypothetical protein, conserved | 3 |

| | | |
|---|---|---|
| Tb927.8.3360 | hypothetical protein, conserved | 3 |
| Tb927.8.4630 | hypothetical protein, conserved | 3 |
| Tb927.8.6620 | hypothetical protein, conserved | 3 |
| Tb09.160.0590 | hypothetical protein, unlikely | 3 |
| Tb09.160.4060 | hypothetical protein, unlikely | 3 |
| Tb09.160.4740 | hypothetical protein, unlikely | 3 |
| Tb09.160.5210 | hypothetical protein, unlikely | 3 |
| Tb09.160.5290 | hypothetical protein, unlikely | 3 |
| Tb09.160.5520 | hypothetical protein, unlikely | 3 |
| Tb09.211.0240 | hypothetical protein, unlikely | 3 |
| Tb09.211.0750 | hypothetical protein, unlikely | 3 |
| Tb09.211.2980 | hypothetical protein, unlikely | 3 |
| Tb09.211.4750 | hypothetical protein, unlikely | 3 |
| Tb09.v1.0750 | hypothetical protein, unlikely | 3 |
| Tb09.v1.0840 | hypothetical protein, unlikely | 3 |
| Tb09.v1.0880 | hypothetical protein, unlikely | 3 |
| Tb927.1.1430 | hypothetical protein, unlikely | 3 |
| Tb927.1.2140 | hypothetical protein, unlikely | 3 |
| Tb927.1.2280 | hypothetical protein, unlikely | 3 |
| Tb927.1.2300 | hypothetical protein, unlikely | 3 |
| Tb927.1.3700 | hypothetical protein, unlikely | 3 |
| Tb927.1.4000 | hypothetical protein, unlikely | 3 |
| Tb927.1.4030 | hypothetical protein, unlikely | 3 |
| Tb927.1.4130 | hypothetical protein, unlikely | 3 |
| Tb927.1.4150 | hypothetical protein, unlikely | 3 |
| Tb927.1.4350 | hypothetical protein, unlikely | 3 |
| Tb927.1.4790 | hypothetical protein, unlikely | 3 |
| Tb10.61.2750 | transporter, putative,major facilitator superfamily protein (MFS), putative | 4 |
| Tb927.4.4080 | C-5 sterol desaturase, putative | 4 |
| Tb927.5.2280 | acetyltransferase, putative | 4 |
| Tb09.211.3640 | prenyl protein specific carboxyl methyltransferase | 4 |
| Tb10.70.2860 | expression site-associated gene (ESAG) protein, putative,expression site-associated gene 3 (ESAG3) protein, putative | 4 |
| Tb11.01.0410 | ER lumen retaining receptor protein, putative | 4 |
| Tb11.02.2200 | hypothetical protein | 4 |

| | | |
|---|---|---|
| Tb11.02.5690 | hypothetical protein | 4 |
| Tb927.4.3480 | hypothetical protein | 4 |
| Tb927.6.1790 | hypothetical protein | 4 |
| Tb927.6.2530 | hypothetical protein | 4 |
| Tb927.6.4730 | hypothetical protein | 4 |
| Tb927.6.4730 | hypothetical protein | 4 |
| Tb927.7.3720 | hypothetical protein | 4 |
| Tb927.7.5860 | hypothetical protein | 4 |
| Tb09.211.1670 | hypothetical protein, conserved | 4 |
| Tb10.406.0080 | hypothetical protein, conserved | 4 |
| Tb10.61.1440 | hypothetical protein, conserved | 4 |
| Tb10.70.0440 | hypothetical protein, conserved | 4 |
| Tb10.70.4000 | hypothetical protein, conserved | 4 |
| Tb11.01.0590 | hypothetical protein, conserved | 4 |
| Tb11.02.0670 | hypothetical protein, conserved | 4 |
| Tb11.02.4850 | hypothetical protein, conserved | 4 |
| Tb11.02.5480 | hypothetical protein, conserved | 4 |
| Tb11.02.5490 | hypothetical protein, conserved | 4 |
| Tb11.12.0008 | hypothetical protein, conserved | 4 |
| Tb11.55.0011 | hypothetical protein, conserved | 4 |
| Tb927.3.4200 | hypothetical protein, conserved | 4 |
| Tb927.4.2530 | hypothetical protein, conserved | 4 |
| Tb927.4.3520 | hypothetical protein, conserved | 4 |
| Tb927.4.5160 | hypothetical protein, conserved | 4 |
| Tb927.6.1930 | hypothetical protein, conserved | 4 |
| Tb927.7.6170 | hypothetical protein, conserved | 4 |
| Tb927.8.3400 | hypothetical protein, conserved | 4 |
| Tb927.8.3730 | hypothetical protein, conserved | 4 |
| Tb927.8.4150 | hypothetical protein, conserved | 4 |
| Tb927.8.4340 | hypothetical protein, conserved | 4 |
| Tb927.8.4520 | hypothetical protein, conserved | 4 |
| Tb927.8.6570 | hypothetical protein, conserved | 4 |
| Tb927.8.7230 | hypothetical protein, conserved | 4 |
| Tb09.160.0090 | hypothetical protein, unlikely | 4 |

| | | |
|---|---|---|
| Tb09.160.5500 | hypothetical protein, unlikely | 4 |
| Tb09.160.5540 | hypothetical protein, unlikely | 4 |
| Tb09.211.2280 | hypothetical protein, unlikely | 4 |
| Tb09.211.4620 | hypothetical protein, unlikely | 4 |
| Tb927.1.2170 | hypothetical protein, unlikely | 4 |
| Tb927.1.770 | hypothetical protein, unlikely | 4 |
| Tb927.8.7860 | receptor-type adenylate cyclase GRESAG 4, putative | 5 |
| Tb09.211.0680 | CAAX prenyl protease 1, putative,metallo-peptidase, Clan M-Family M48 | 5 |
| Tb09.211.1010 | phosphatidylcholine:ceramide cholinephosphotransferase 2, putative | 5 |
| Tb10.100.0090 | vacuolar ATP synthase, putative | 5 |
| Tb09.211.0250 | hypothetical protein | 5 |
| Tb927.1.4020 | hypothetical protein | 5 |
| Tb927.4.2690 | hypothetical protein | 5 |
| Tb09.160.4800 | hypothetical protein, conserved | 5 |
| Tb10.6k15.3420 | hypothetical protein, conserved | 5 |
| Tb10.70.7520 | hypothetical protein, conserved | 5 |
| Tb11.01.7180 | hypothetical protein, conserved | 5 |
| Tb11.02.1710 | hypothetical protein, conserved | 5 |
| Tb11.02.2220 | hypothetical protein, conserved | 5 |
| Tb927.3.2450 | hypothetical protein, conserved | 5 |
| Tb927.3.3820 | hypothetical protein, conserved | 5 |
| Tb927.3.710 | hypothetical protein, conserved | 5 |
| Tb927.4.3500 | hypothetical protein, conserved | 5 |
| Tb927.5.4140 | hypothetical protein, conserved | 5 |
| Tb927.6.1170 | hypothetical protein, conserved | 5 |
| Tb927.6.1420 | hypothetical protein, conserved | 5 |
| Tb927.7.6130 | hypothetical protein, conserved | 5 |
| Tb927.8.2460 | hypothetical protein, conserved | 5 |
| Tb10.05.0100 | hypothetical protein, conserved,hypothetical protein | 5 |
| Tb09.160.2730 | hypothetical protein, unlikely | 5 |
| Tb927.1.1780 | hypothetical protein, unlikely | 5 |
| Tb11.03.0540 | ABCB10  ABC transporter, putative | 6 |
| Tb927.8.1810 | rhomboid-like protein,serine peptidase, Clan S-, family S54, putative | 6 |
| Tb927.3.3860 | lipase, putative,triacylglycerol lipase, putative | 6 |

| | | |
|---|---|---|
| Tb927.8.480 | phosphatidic acid phosphatase protein, putative | 6 |
| Tb10.61.2970 | phosphatidic acid phosphatase, putative | 6 |
| Tb927.3.3360 | acyltransferase, putative | 6 |
| Tb10.100.0100 | GPI transamidase component GAA1 | 6 |
| Tb927.2.4440 | hypothetical protein | 6 |
| Tb09.160.3780 | hypothetical protein, conserved | 6 |
| Tb10.6k15.1640 | hypothetical protein, conserved | 6 |
| Tb10.70.2130 | hypothetical protein, conserved | 6 |
| Tb10.70.3750 | hypothetical protein, conserved | 6 |
| Tb11.01.3540 | hypothetical protein, conserved | 6 |
| Tb11.01.6760 | hypothetical protein, conserved | 6 |
| Tb11.02.4710 | hypothetical protein, conserved | 6 |
| Tb11.39.0005 | hypothetical protein, conserved | 6 |
| Tb927.2.2920 | hypothetical protein, conserved | 6 |
| Tb927.3.2850 | hypothetical protein, conserved | 6 |
| Tb927.4.4880 | hypothetical protein, conserved | 6 |
| Tb927.5.2030 | hypothetical protein, conserved | 6 |
| Tb927.7.6110 | hypothetical protein, conserved | 6 |
| Tb927.8.6700 | hypothetical protein, conserved | 6 |
| Tb927.8.6710 | hypothetical protein, conserved | 6 |
| Tb927.8.6720 | hypothetical protein, conserved | 6 |
| Tb927.8.6730 | hypothetical protein, conserved | 6 |
| Tb10.6k15.3270 | hypothetical protein, conserved,predicted zinc finger protein | 6 |
| Tb11.01.4490 | hypothetical protein, conserved,Zinc finger DHHC domain containing transmembrane protein, putative | 6 |
| Tb10.406.0290 | protein tyrosine phosphatase, putative | 7 |
| Tb10.61.3010 | UDP-galactose transporter, putative | 7 |
| Tb927.6.3300 | TbPIG-M mannosyltransferase | 7 |
| Tb927.2.4720 | ALG12 dolichyl-P-Man:Man7GlcNAc2-PP-dolichylalpha6-ma nn osyltransferase,putative | 7 |
| Tb09.211.4800 | hypothetical protein, conserved | 7 |
| Tb10.6k15.2580 | hypothetical protein, conserved | 7 |
| Tb11.01.4220 | hypothetical protein, conserved | 7 |
| Tb11.18.0010 | hypothetical protein, conserved | 7 |
| Tb927.2.2830 | hypothetical protein, conserved | 7 |
| Tb927.5.3540 | hypothetical protein, conserved | 7 |

| | | |
|---|---|---|
| Tb927.7.950 | hypothetical protein, conserved | 7 |
| Tb927.8.7580 | hypothetical protein, conserved | 7 |
| Tb10.61.0890 | transporter, putative | 8 |
| Tb927.3.4910 | signal peptide peptidase, putative,aspartic peptidase, clan AD, family A22B, putative | 8 |
| Tb927.7.3760 | phosphatidyl serine synthase, putative | 8 |
| Tb10.70.1440 | GPI10 GPI anchor biosynthesis protein | 8 |
| Tb10.70.0260 | alpha-1,3-mannosyltransferase, putative | 8 |
| Tb09.160.1010 | hypothetical protein, conserved | 8 |
| Tb10.6k15.0240 | hypothetical protein, conserved | 8 |
| Tb927.4.320 | hypothetical protein, conserved | 8 |
| Tb927.6.3960 | hypothetical protein, conserved | 8 |
| Tb927.8.1530 | hypothetical protein, conserved | 8 |
| Tb927.8.2380 | ABC transporter, putative | 9 |
| Tb11.02.1100 | NT8.1 nucleobase/nucleoside transporter 8.1,nucleobase transporter | 9 |
| Tb11.02.1105 | NT8.1 nucleobase/nucleoside transporter 8.1,nucleobase transporter | 9 |
| Tb11.02.1106 | nucleobase transporter, putative | 9 |
| Tb11.02.4100 | pretranslocation protein, alpha subunit, putative,SEC61-like (pretranslocation process) protein, putative | 9 |
| Tb11.02.0960 | endosomal integral membrane protein, putative | 9 |
| Tb927.4.1090 | endosomal integral membrane protein, putative | 9 |
| Tb927.8.1940 | endosomal integral membrane protein, putative | 9 |
| Tb10.389.1220 | hypothetical protein, conserved | 9 |
| Tb927.3.1390 | hypothetical protein, conserved | 9 |
| Tb927.6.1980 | hypothetical protein, conserved | 9 |
| Tb09.160.5480 | TbNT10 purine nucleoside transporter | 10 |
| Tb927.2.6220 | TbNT4 adenosine transporter 2, putative | 10 |
| Tb927.6.220 | TbNT9 purine nucleoside transporter | 10 |
| Tb927.8.7680 | amino acid transporter, putative | 10 |
| Tb927.8.7700 | amino acid transporter, putative | 10 |
| Tb927.1.580 | phosphate-repressible phosphate permease, putative | 10 |
| Tb927.1.600 | phosphate-repressible phosphate permease, putative | 10 |
| Tb927.6.1140 | Alg9-like mannosyltransferase, putative | 10 |
| Tb09.160.0430 | hypothetical protein, conserved | 10 |
| Tb10.70.2990 | hypothetical protein, conserved | 10 |
| Tb11.02.2720 | hypothetical protein, conserved | 10 |

| | | |
|---|---|---|
| Tb927.8.5570 | hypothetical protein, conserved,transporter, putative | 10 |
| Tb927.3.3730 | ABC transporter, putative | 11 |
| Tb927.3.590 | adenosine transporter, putative | 11 |
| Tb09.244.2020 | nucleoside transporter 1, putative | 11 |
| Tb927.2.6150 | TbNT2/927 adenosine transporter 2 | 11 |
| Tb927.2.6200 | TbNT3 adenosine transporter 2, putative | 11 |
| Tb927.2.6240 | TbNT5 adenosine transporter 2 | 11 |
| Tb927.2.6320 | TbNT6 adenosine transporter 2, putative | 11 |
| Tb927.2.6280 | TbNT7 adenosine transporter 2, putative | 11 |
| Tb09.211.3870 | transporter, putative | 11 |
| Tb09.211.3740 | hypothetical protein, conserved | 11 |
| Tb11.02.3020 | sugar transporter, putative | 12 |
| Tb10.389.1330 | membrane transporter protein, putative | 12 |
| Tb927.3.4190 | endosomal integral membrane protein, putative | 12 |
| Tb927.4.2510 | PGPA multidrug resistance-associated protein, putative,P-glycoprotein | 12 |
| Tb927.3.4090 | hypothetical protein, conserved | 13 |
| Tb927.4.4380 | PPase1 vacuolar-type proton translocating pyrophosphatase 1, putative | 14 |
| Tb927.8.7980 | TVP1 vacuolar-type proton translocating pyrophosphatase 1,V-type H(+)-translocating pyrophosphatase | 14 |
| Tb927.3.1470 | variant surface glycoprotein (VSG)-related, putative | 14 |
| Tb927.3.4080 | hypothetical protein, conserved | 14 |
| Tb927.3.4100 | hypothetical protein, conserved | 14 |
| Tb927.3.4030 | hypothetical protein, conserved | 16 |
| Tb11.18.0008 | diacylglycerol acyltransferase, putative | 25 |

**Table S6**
**SHEET 2 (SecretomeP Prediction)**

**SecretomeP prediction NNscore>0,9 on T. brucei proteome**

| Accession number | protein name | TMHMM prediction |
|---|---|---|
| Tb10.61.0340 | cation transporter, putative | 1 |
| Tb927.3.2290 | chaperone protein DNAJ, putative | 0 |
| Tb927.7.5880 | cis-prenyltransferase-like protein | 1 |
| Tb11.01.6540 | PP2C protein phosphatase 2C, putative | 2 |
| Tb10.6k15.0890 | expression site-associated gene (ESAG) protein, putative,expression site-associated gene 3 (ESAG3) protein, putative | 1 |
| Tb10.70.6660 | hypoxanthine-guanine phosphoribosyltransferase, putative | 0 |
| Tb11.01.2886 | N-acetyltransferase, putative | 0 |
| Tb11.01.4702 | COXX cytochrome oxidase subunit X | 0 |
| Tb11.02.0820 | ras-family member, GTP-binding protein, putative | 1 |
| Tb11.01.6590 | tbZFP2 zinc finger protein 2 | 0 |
| Tb09.211.0790 | tRNA (guanine-N(7)-)-methyltransferase, putative,methyltransferase, putative | 0 |
| Tb09.211.4700 | RISP reiske iron-sulfur protein , mitochondrial precursor | 1 |
| Tb927.6.4340 | TbLSm5 U6 snRNA-associated Sm-like protein LSm5p | 0 |
| Tb10.6k15.2200 | RBP14A RNA-binding protein, putative,RBP14A | 0 |
| Tb10.6k15.2230 | RBP14B RNA-binding protein, putative,RBP14B | 0 |
| Tb09.211.4920 | dynein-associated protein, putative | 0 |
| Tb10.6k15.0510 | 60S ribosomal protein L22 | 0 |
| Tb11.01.1920 | 60S ribosomal protein L22, putative | 0 |
| Tb09.142.0320 | hypothetical protein | 0 |
| Tb10.70.7110 | hypothetical protein | 0 |
| Tb11.01.3250 | hypothetical protein | 0 |
| Tb11.01.3800 | hypothetical protein | 0 |
| Tb11.01.5030 | hypothetical protein | 1 |
| Tb11.01.7320 | hypothetical protein | 3 |
| Tb11.02.0390 | hypothetical protein | 0 |
| Tb11.02.1240 | hypothetical protein | 0 |
| Tb927.2.1290 | hypothetical protein | 0 |
| Tb927.2.5700 | hypothetical protein | 0 |

| | | |
|---|---|---|
| Tb927.3.4830 | hypothetical protein | 0 |
| Tb927.3.5770 | hypothetical protein | 0 |
| Tb927.4.3050 | hypothetical protein | 0 |
| Tb927.5.1040 | hypothetical protein | 0 |
| Tb927.5.1970 | hypothetical protein | 0 |
| Tb927.6.3080 | hypothetical protein | 1 |
| Tb927.7.7560 | hypothetical protein | 0 |
| Tb09.142.0390 | hypothetical protein, conserved | 0 |
| Tb09.160.0690 | hypothetical protein, conserved | 0 |
| Tb09.160.1690 | hypothetical protein, conserved | 0 |
| Tb09.160.2160 | hypothetical protein, conserved | 1 |
| Tb09.160.2480 | hypothetical protein, conserved | 0 |
| Tb09.211.0230 | hypothetical protein, conserved | 0 |
| Tb09.211.0890 | hypothetical protein, conserved | 0 |
| Tb09.211.3460 | hypothetical protein, conserved | 0 |
| Tb09.211.4740 | hypothetical protein, conserved | 1 |
| Tb10.26.0540 | hypothetical protein, conserved | 0 |
| Tb10.26.0640 | hypothetical protein, conserved | 0 |
| Tb10.389.1760 | hypothetical protein, conserved | 0 |
| Tb10.61.0510 | hypothetical protein, conserved | 0 |
| Tb10.61.3115 | hypothetical protein, conserved | 0 |
| Tb10.70.2155 | hypothetical protein, conserved | 0 |
| Tb10.70.6260 | hypothetical protein, conserved | 1 |
| Tb10.6k15.2440 | hypothetical protein, conserved | 0 |
| Tb10.6k15.3700 | hypothetical protein, conserved | 0 |
| Tb10.6k15.3900 | hypothetical protein, conserved | 0 |
| Tb11.01.0520 | hypothetical protein, conserved | 0 |
| Tb11.01.0640 | hypothetical protein, conserved | 1 |
| Tb11.01.2300 | hypothetical protein, conserved | 0 |
| Tb11.01.2970 | hypothetical protein, conserved | 0 |
| Tb11.01.4120 | hypothetical protein, conserved | 0 |
| Tb11.01.4190 | hypothetical protein, conserved | 1 |
| Tb11.01.4330 | hypothetical protein, conserved | 3 |
| Tb11.01.6350 | hypothetical protein, conserved | 0 |

| | | |
|---|---|---|
| Tb11.01.8000 | hypothetical protein, conserved | 1 |
| Tb11.01.8630 | hypothetical protein, conserved | 0 |
| Tb11.02.0420 | hypothetical protein, conserved | 0 |
| Tb11.02.3060 | hypothetical protein, conserved | 0 |
| Tb11.02.4780 | hypothetical protein, conserved | 0 |
| Tb11.02.5750 | hypothetical protein, conserved | 0 |
| Tb11.03.0510 | hypothetical protein, conserved | 1 |
| Tb11.57.0002 | hypothetical protein, conserved | 1 |
| Tb927.1.400 | hypothetical protein, conserved | 0 |
| Tb927.2.3920 | hypothetical protein, conserved | 0 |
| Tb927.2.5140 | hypothetical protein, conserved | 1 |
| Tb927.3.3530 | hypothetical protein, conserved | 0 |
| Tb927.4.3080 | hypothetical protein, conserved | 0 |
| Tb927.4.3450 | hypothetical protein, conserved | 1 |
| Tb927.4.3580 | hypothetical protein, conserved | 0 |
| Tb927.4.3600 | hypothetical protein, conserved | 0 |
| Tb927.4.4600 | hypothetical protein, conserved | 0 |
| Tb927.4.4810 | hypothetical protein, conserved | 2 |
| Tb927.4.940 | hypothetical protein, conserved | 1 |
| Tb927.5.3000 | hypothetical protein, conserved | 1 |
| Tb927.6.1100 | hypothetical protein, conserved | 0 |
| Tb927.6.3240 | hypothetical protein, conserved | 0 |
| Tb927.6.3770 | hypothetical protein, conserved | 3 |
| Tb927.6.4330 | hypothetical protein, conserved | 0 |
| Tb927.6.5080 | hypothetical protein, conserved | 1 |
| Tb927.7.2280 | hypothetical protein, conserved | 0 |
| Tb927.7.6300 | hypothetical protein, conserved | 0 |
| Tb927.7.6770 | hypothetical protein, conserved | 0 |
| Tb927.8.1120 | hypothetical protein, conserved | 1 |
| Tb927.8.1570 | hypothetical protein, conserved | 0 |
| Tb927.8.1770 | hypothetical protein, conserved | 1 |
| Tb927.8.1790 | hypothetical protein, conserved | 0 |
| Tb927.8.2340 | hypothetical protein, conserved | 0 |
| Tb927.8.2360 | hypothetical protein, conserved | 0 |

| | | |
|---|---|---|
| Tb927.8.3070 | hypothetical protein, conserved | 1 |
| Tb927.8.4380 | hypothetical protein, conserved | 1 |
| Tb927.8.4550 | hypothetical protein, conserved | 0 |
| Tb927.8.5190 | hypothetical protein, conserved | 0 |
| Tb927.8.5200 | hypothetical protein, conserved | 0 |
| Tb927.8.5760 | hypothetical protein, conserved | 1 |
| Tb927.8.7040 | hypothetical protein, conserved | 0 |
| Tb11.01.4160 | hypothetical protein, conserved,predicted Zinc finger, C3HC4 type (RING finger) protein | 0 |
| Tb09.160.2080 | hypothetical protein, unlikely | 0 |
| Tb09.211.1660 | hypothetical protein, unlikely | 1 |
| Tb09.211.2690 | hypothetical protein, unlikely | 0 |
| Tb09.211.2710 | hypothetical protein, unlikely | 1 |
| Tb09.244.2000 | hypothetical protein, unlikely | 0 |
| Tb09.244.2090 | hypothetical protein, unlikely | 0 |
| Tb09.v1.0080 | hypothetical protein, unlikely | 0 |
| Tb09.v1.0270 | hypothetical protein, unlikely | 0 |
| Tb09.v1.0730 | hypothetical protein, unlikely | 0 |
| Tb11.01.2080 | hypothetical protein, unlikely | 0 |
| Tb11.02.1570 | hypothetical protein, unlikely | 0 |
| Tb927.1.1800 | hypothetical protein, unlikely | 1 |
| Tb927.1.230 | hypothetical protein, unlikely | 0 |
| Tb927.1.250 | hypothetical protein, unlikely | 0 |
| Tb927.1.2960 | hypothetical protein, unlikely | 0 |
| Tb927.1.3470 | hypothetical protein, unlikely | 0 |
| Tb927.1.3590 | hypothetical protein, unlikely | 0 |
| Tb927.1.3630 | hypothetical protein, unlikely | 0 |
| Tb927.1.4460 | hypothetical protein, unlikely | 1 |
| Tb927.1.4880 | hypothetical protein, unlikely | 0 |
| Tb927.1.5210 | hypothetical protein, unlikely | 0 |
| Tb09.211.4940 | hypothetical protein, unlikely,Ingi-like sequence | 0 |
| Tb10.05.0180 | hypothetical protein,hypothetical protein | 0 |
| Tb09.211.4860 | hypothetical protein,leucine-rich repeat protein (LRRP), putative | 0 |

**SecretomeP prediction 0,9<NNscore>0,8 on T. brucei proteome**

| Accession number | protein name | TMHMM prediction |
|---|---|---|
| Tb09.211.1760 | amino acid transporter, putative | 11 |
| Tb927.4.4020 | amino acid transporter, putative | 11 |
| Tb927.8.4710 | amino acid transporter, putative | 10 |
| Tb927.8.4720 | amino acid transporter, putative | 10 |
| Tb927.8.4730 | amino acid transporter, putative | 10 |
| Tb927.8.4740 | amino acid transporter, putative | 10 |
| Tb927.8.7670 | amino acid transporter, putative | 11 |
| Tb927.4.4730 | AATP11 amino acid transporter, putative | 11 |
| Tb927.8.4700 | AATP6 amino acid transporter, putative | 10 |
| Tb11.03.0030 | ABCD3 ABC transporter, putative | 3 |
| Tb09.160.3380 | calcium/potassium channel (CAKC), putative | 7 |
| Tb927.8.650 | cation-transporting ATPase, putative | 8 |
| Tb09.211.0430 | chloride channel protein, putative | 11 |
| Tb11.01.8280 | CMP-sialic acid transporter, putative | 9 |
| Tb09.160.1140 | electon transport protein SCO1/SCO2, putative | 1 |
| Tb10.61.0090 | potassium voltage-gated channel, putative | 0 |
| Tb09.211.3210 | transport protein particle (TRAPP) subunit, putative | 0 |
| Tb10.61.1380 | SEC22 synaptobrevin-type vesicle transport protein, putative,protein SLY2 | 1 |
| Tb11.01.5650 | protein kinase, putative | 3 |
| Tb927.3.5650 | protein kinase, putative,serine/threonine protein kinase, putative | 2 |
| Tb11.01.8085 | CKS1 cyclin dependent kinases regulatory subunit, putative | 0 |
| Tb10.70.1200 | adenylate kinase, putative | 0 |
| Tb927.4.4070 | mevalonate kinase, putative | 0 |
| Tb10.70.0170 | chaperone protein DNAJ, putative | 1 |
| Tb10.70.2160 | chaperone protein DNAJ, putative | 0 |
| Tb927.4.650 | chaperone protein DNAJ, putative | 1 |
| Tb927.6.2480 | chaperone protein DNAJ, putative | 0 |
| Tb927.8.8310 | chaperone protein DnaJ, putative | 2 |
| Tb927.3.3330 | heat shock protein 20, putative | 0 |
| Tb927.8.690 | peptidyl-prolyl cis-trans isomerase/rotamase, putative,PPIase, putative | 0 |
| Tb927.2.3460 | D-alanyl-glycyl endopeptidase-like protein,cysteine peptidase, Clan CA, family C51, putative | 1 |

| | | |
|---|---|---|
| Tb09.160.3510 | presenilin-like aspartic peptidase, putative,presenilin-like aspartic peptidase, clan AD, family A22A, putative | 7 |
| Tb927.5.3220 | signal peptidase type I, putative,serine peptidase, Clan SF, Family S26A | 0 |
| Tb11.01.2000 | hslVU complex proteolytic subunit, putative,hslVU complex proteolytic subunit, threonine peptidase, Clan T(1), family T1B | 0 |
| Tb927.5.1880 | inhibitor of serine peptidase (ISP), putative,ecotin family (I11), putative | 0 |
| Tb927.6.4030 | superoxide dismutase, putative | 0 |
| Tb09.160.2020 | trx thioredoxin | 0 |
| Tb927.3.4240 | thioredoxin, putative | 0 |
| Tb09.160.2210 | glutaredoxin-like protein | 0 |
| Tb11.02.1990 | ferric reductase, putative,ferric reductase transmembrane component, putative | 11 |
| Tb10.70.6540 H | GPRT hypoxanthine-guanine phosphoribosyltransferase | 0 |
| Tb09.211.1000 | phosphatidylcholine:ceramide cholinephosphotransferase 2, putative | 5 |
| Tb09.211.1020 | phosphatidylcholine:ceramide cholinephosphotransferase 2, putative | 4 |
| Tb09.211.1030 | phosphatidylcholine:ceramide cholinephosphotransferase 2, putative | 5 |
| Tb927.5.3580 | phosphoglycerate mutase protein, putative | 0 |
| Tb11.01.4790 | phospholipid:diacylglycerol acyltransferase-like protein | 1 |
| Tb927.2.1780 | N-acetylglucosaminyl-phosphatidylinositol biosynthetic protein, putative | 1 |
| Tb10.61.3180 | CaLB calcium-dependent lipid binding protein, putative,synaptotagmin, putative | 3 |
| Tb11.01.4180 | ankyrin repeat protein, putative | 0 |
| Tb11.02.5800 | calmodulin, putative | 0 |
| Tb11.22.0008 | molybdopterin synthase sulphurylase protein, putative | 1 |
| Tb10.6k15.1570 | ethanolaminephosphotransferase, putative | 8 |
| Tb927.5.3810 | orotidine-5-phosphate decarboxylase/orotate phosphoribosyltransferase, putative,OMPDCase-OPRTase, putative | 0 |
| Tb927.8.3530 | glycerol-3-phosphate dehydrogenase [NAD+], glycosomal | 0 |
| Tb927.8.1440 | maoC-like dehydratase, putative | 0 |
| Tb927.8.5690 | KREPB8 hypothetical protein, conserved | 0 |
| Tb10.6k15.2720 | protein-L-isoaspartate, putative | 0 |
| Tb09.211.0740 | p21 antigen protein, putative | 0 |
| Tb927.6.460 | PAG3 procyclin associated gene 3 (PAG3) protein,procyclin PARP A,procyclic acidic repetitive protein A | 1 |
| Tb927.6.490 | PAG3 procyclin associated gene 3 (PAG3) protein,procyclin PARP A,procyclic acidic repetitive protein A | 1 |
| Tb927.6.530 | PAG3 procyclin associated gene 3 (PAG3) protein,procyclin PARP A,procyclic acidic repetitive protein A | 1 |
| Tb09.211.2730 | gim5A Gim5A protein,glycosomal membrane protein | 1 |
| Tb09.211.2740 | gim5B Gim5B protein,glycosomal membrane protein | 1 |
| Tb11.52.0014 | ras-related GTP-binding protein, putative | 0 |
| Tb09.211.0540 | FBPase fructose-1,6-bisphosphate, cytosolic | 0 |
| Tb927.2.5800 | SBPase sedoheptulose-1,7-bisphosphatase | 0 |
| Tb10.6k15.2180 | COXIX cytochrome oxidase subunit IX | 1 |

| | | |
|---|---|---|
| Tb927.3.3470 | CYB5 cytochrome b5, putative | 0 |
| Tb09.211.0510 | cytochrome c oxidase assembly factor, putative | 1 |
| Tb927.1.1580 | cytochrome c oxidase assembly factor, putative,electron transport protein SCO1/2, putative | 0 |
| Tb10.70.4380 | cytochrome C oxidase assembly protein, putative | 1 |
| Tb927.3.1790 | pyruvate dehydrogenase E1 beta subunit, putative | 0 |
| Tb11.55.0009 | GBP21 mitochondrial RNA binding protein 1,gBP21, MRP1 | 0 |
| Tb10.6k15.0980 | ATP-dependent chaperone, putative,mitochondrial chaperone BCS1, putative | 1 |
| Tb10.6k15.3030 | mitochondrial ATP-dependent zinc metallopeptidase, putative,metallo-peptidase, Clan MA(E) Family M41 | 1 |
| Tb927.4.3300 | mitochondrial ATP-dependent zinc metallopeptidase, putative,metallo-peptidase, Clan MA(E) Family M41 | 1 |
| Tb09.211.3200 | mitochondrial carrier protein, putative | 1 |
| Tb11.47.0017 | NDHK NADH-ubiquinone oxidoreductase 20 kDa subunit, mitochondrial precursor | 0 |
| Tb927.4.4910 | 3,2-trans-enoyl-CoA isomerase, mitochondrial precursor, putative | 0 |
| Tb927.8.7530 | 3,2-trans-enoyl-CoA isomerase, mitochondrial precursor, putative | 0 |
| Tb927.4.2260 | centrin, putative | 0 |
| Tb927.3.5180 | cofilin/actin depolymerizing factor, putative | 0 |
| Tb927.7.820 | dynein arm light chain, axonemal, putative | 0 |
| Tb10.61.0630 | anaphase promoting complex, subunit 10-like protein | 0 |
| Tb09.211.2300 | ATP-dependent DEAD/H RNA helicase, putative | 3 |
| Tb11.01.2190 | RPA12 DNA-directed RNA polymerase, putative | 0 |
| Tb11.01.6090 | RPB7 RNA polymerase subunit, putative | 0 |
| Tb927.7.5460 | EAP3 exosome-associated protein 3,3' exoribonuclease, putative | 0 |
| Tb10.61.1040 | RPC40 DNA-directed RNA polymerase, alpha subunit, putative,RNA polymerase subunit, putative | 0 |
| Tb11.01.0060 | DNA polymerase kappa, putative,DNA polymerase IV, putative | 0 |
| Tb927.3.1360 | cytosine-specific DNA methylase, putative | 0 |
| Tb10.6k15.3500 | D-tyrosyl-tRNA deacylase, putative | 0 |
| Tb927.3.4900 | tRNA isopentenyltransferase, putative | 0 |
| Tb927.8.1830 | tRNA-methyl transferase, putative | 0 |
| Tb927.1.1370 | rRNA biogenesis protein, putative | 0 |
| Tb927.1.3180 | 40S ribosomal protein S11, putative | 0 |
| Tb11.02.4000 | 40S ribosomal protein S15a, putative | 0 |
| Tb927.3.4360 | 40S ribosomal protein S15a, putative | 0 |
| Tb10.70.7695 | 40S ribosomal proteins S11, putative | 0 |
| Tb927.4.930 | 50S ribosomal protein L14, putative | 1 |
| Tb927.7.3960 | 50S ribosomal protein L16, putative | 0 |
| Tb927.5.3360 | 50S ribosomal protein L2, putative | 0 |
| Tb09.211.2630 | 60S ribosomal protein L23, putative | 0 |

| | | | |
|---|---|---|---|
| Tb10.70.3160 | R | PL30 60S ribosomal protein L30 | 0 |
| Tb09.211.2640 | | 60S ribosomal protein L23, putative | 0 |
| Tb927.3.2930 | | RBP6 RNA-binding protein RBP6, putative | 0 |
| Tb927.2.4540 | | small nuclear ribonucleoprotein SmD1, putative | 0 |
| Tb10.70.2250 | | small nuclear ribonucleoprotein, putative | 0 |
| Tb927.7.320 | | TbRBP8 hypothetical protein, conserved | 0 |
| Tb927.3.1780 | | TbLSm8 U6 snRNA-associated Sm-like protein LSm8p | 0 |
| Tb927.7.3120 | | Sm-D1 small nuclear ribonucleoprotein SmD1,SmD-1 like small nuclear ribonucleoprotein | 0 |
| Tb927.2.5850 | | Sm-D2 small nuclear ribonucleoprotein SmD2 | 0 |
| Tb927.4.890 | | SmD3 small nuclear ribonucleoprotein SmD3, putative | 0 |
| Tb10.70.2220 | D | SK2 ubiquitin-like protein DSK2, putative | 0 |
| Tb927.8.2560 | | spliceosomal U5 snRNP-specific protein, putative | 0 |
| Tb10.6k15.1460 | | kinetoplast DNA-associated protein, putative | 0 |
| Tb927.5.3210 | | small ubiquitin protein, putative | 0 |
| Tb927.4.2540 | | ubiquitin, putative | 0 |
| Tb10.70.2170 | | ubiquitin/ribosomal protein S27a, putative | 0 |
| Tb927.7.3680 | | ubiquitin/ribosomal protein S27a, putative | 0 |
| Tb927.5.1000 | | ubiquitin-conjugating enzyme E2, putative,ubiquitin carrier protein, putative,ubiquitin-protein ligase, putative | 0 |
| Tb927.8.6510 | | ubiquitin-conjugating enzyme E2, putative,ubiquitin-protein ligase, putative,ubiquitin carrier protein, putative | 0 |
| Tb927.8.920 | | ubiquitin-conjugating enzyme E2, putative,ubiquitin-protein ligase, putative,ubiquitin carrier protein, putative | 0 |
| Tb11.02.0815 | | ubiquitin-conjugating enzyme, putative,ubiquitin-conjugating enzyme-like, putative | 0 |
| Tb927.7.3900 | | vacuolar transporter chaperone, putative | 3 |
| Tb09.142.0410 | | hypothetical protein | 0 |
| Tb09.160.1670 | | hypothetical protein | 0 |
| Tb09.211.0260 | | hypothetical protein | 0 |
| Tb09.211.2320 | | hypothetical protein | 2 |
| Tb09.211.4030 | | hypothetical protein | 0 |
| Tb09.244.2150 | | hypothetical protein | 0 |
| Tb10.70.2240 | | hypothetical protein | 0 |
| Tb10.70.2870 | | hypothetical protein | 0 |
| Tb10.70.2880 | | hypothetical protein | 0 |
| Tb10.70.2890 | | hypothetical protein | 0 |
| Tb10.70.5240 | | hypothetical protein | 0 |
| Tb10.70.6070 | | hypothetical protein | 0 |
| Tb10.70.6110 | | hypothetical protein | 0 |
| Tb10.70.6870 | | hypothetical protein | 0 |

| | | |
|---|---|---|
| Tb10.70_v2.40 | hypothetical protein | 0 |
| Tb10.6k15.0720 | hypothetical protein | 4 |
| Tb10.6k15.2600 | hypothetical protein | 0 |
| Tb10.6k15.2980 | hypothetical protein | 0 |
| Tb10.6k15.3010 | hypothetical protein | 0 |
| Tb10.6k15.3520 | hypothetical protein | 0 |
| Tb10.61.2830 | hypothetical protein | 0 |
| Tb11.01.4580 | hypothetical protein | 0 |
| Tb11.01.5290 | hypothetical protein | 0 |
| Tb11.01.6110 | hypothetical protein | 0 |
| Tb11.01.7040 | hypothetical protein | 0 |
| Tb11.02.0865 | hypothetical protein | 0 |
| Tb927.1.30 | hypothetical protein | 0 |
| Tb927.1.3350 | hypothetical protein | 0 |
| Tb927.1.3890 | hypothetical protein | 0 |
| Tb927.1.490 | hypothetical protein | 0 |
| Tb927.1.4970 | hypothetical protein | 0 |
| Tb927.1.5250 | hypothetical protein | 0 |
| Tb927.2.3340 | hypothetical protein | 0 |
| Tb927.2.5710 | hypothetical protein | 0 |
| Tb927.3.4010 | hypothetical protein | 0 |
| Tb927.3.5780 | hypothetical protein | 0 |
| Tb927.4.4780 | hypothetical protein | 2 |
| Tb927.5.1480 | hypothetical protein | 2 |
| Tb927.5.2000 | hypothetical protein | 0 |
| Tb927.5.2010 | hypothetical protein | 0 |
| Tb927.6.250 | hypothetical protein | 0 |
| Tb927.7.330 | hypothetical protein | 0 |
| Tb927.7.6430 | hypothetical protein | 0 |
| Tb927.7.6690 | hypothetical protein | 0 |
| Tb927.7.7350 | hypothetical protein | 0 |
| Tb927.7.7390 | hypothetical protein | 0 |
| Tb927.7.7550 | hypothetical protein | 0 |
| Tb927.8.4490 | hypothetical protein | 0 |
| Tb927.8.5340 | hypothetical protein | 2 |
| Tb927.8.5930 | hypothetical protein | 0 |

| | | |
|---|---|---|
| Tb927.8.6630 | hypothetical protein | 0 |
| Tb927.8.7370 | hypothetical protein | 0 |
| Tb09.142.0420 | hypothetical protein, conserved | 0 |
| Tb09.160.0410 | hypothetical protein, conserved | 0 |
| Tb09.160.0465 | hypothetical protein, conserved | 0 |
| Tb09.160.0540 | hypothetical protein, conserved | 0 |
| Tb09.160.0560 | hypothetical protein, conserved | 0 |
| Tb09.160.0730 | hypothetical protein, conserved | 0 |
| Tb09.160.0950 | hypothetical protein, conserved | 0 |
| Tb09.160.1100 | hypothetical protein, conserved | 0 |
| Tb09.160.1470 | hypothetical protein, conserved | 0 |
| Tb09.160.1660 | hypothetical protein, conserved | 0 |
| Tb09.160.2130 | hypothetical protein, conserved | 0 |
| Tb09.160.2460 | hypothetical protein, conserved | 2 |
| Tb09.160.3910 | hypothetical protein, conserved | 0 |
| Tb09.160.5130 | hypothetical protein, conserved | 1 |
| Tb09.160.5150 | hypothetical protein, conserved | 0 |
| Tb09.160.5230 | hypothetical protein, conserved | 0 |
| Tb09.160.5270 | hypothetical protein, conserved | 0 |
| Tb09.211.0040 | hypothetical protein, conserved | 0 |
| Tb09.211.0530 | hypothetical protein, conserved | 0 |
| Tb09.211.1220 | hypothetical protein, conserved | 3 |
| Tb09.211.1940 | hypothetical protein, conserved | 0 |
| Tb09.211.2190 | hypothetical protein, conserved | 0 |
| Tb09.211.2205 | hypothetical protein, conserved | 0 |
| Tb09.211.2270 | hypothetical protein, conserved | 0 |
| Tb09.211.2620 | hypothetical protein, conserved | 0 |
| Tb09.211.3030 | hypothetical protein, conserved | 1 |
| Tb09.211.3300 | hypothetical protein, conserved | 0 |
| Tb09.211.3470 | hypothetical protein, conserved | 0 |
| Tb09.211.4010 | hypothetical protein, conserved | 0 |
| Tb09.211.4320 | hypothetical protein, conserved | 0 |
| Tb09.211.4360 | hypothetical protein, conserved | 0 |
| Tb09.211.4520 | hypothetical protein, conserved | 0 |
| Tb09.244.2810 | hypothetical protein, conserved | 0 |
| Tb09.244.2840 | hypothetical protein, conserved | 0 |

| | | |
|---|---|---|
| Tb10.70.0230 | hypothetical protein, conserved | 0 |
| Tb10.70.0730 | hypothetical protein, conserved | 0 |
| Tb10.70.1340 | hypothetical protein, conserved | 0 |
| Tb10.70.1420 | hypothetical protein, conserved | 0 |
| Tb10.70.2080 | hypothetical protein, conserved | 0 |
| Tb10.70.2310 | hypothetical protein, conserved | 0 |
| Tb10.70.2670 | hypothetical protein, conserved | 0 |
| Tb10.70.2830 | hypothetical protein, conserved | 0 |
| Tb10.70.2970 | hypothetical protein, conserved | 0 |
| Tb10.70.3150 | hypothetical protein, conserved | 0 |
| Tb10.70.3390 | hypothetical protein, conserved | 6 |
| Tb10.70.3910 | hypothetical protein, conserved | 0 |
| Tb10.70.4810 | hypothetical protein, conserved | 0 |
| Tb10.70.4990 | hypothetical protein, conserved | 0 |
| Tb10.70.5000 | hypothetical protein, conserved | 0 |
| Tb10.70.5210 | hypothetical protein, conserved | 3 |
| Tb10.70.5230 | hypothetical protein, conserved | 1 |
| Tb10.70.5590 | hypothetical protein, conserved | 0 |
| Tb10.70.5970 | hypothetical protein, conserved | 0 |
| Tb10.70.6310 | hypothetical protein, conserved | 0 |
| Tb10.70.6600 | hypothetical protein, conserved | 0 |
| Tb10.70.6890 | hypothetical protein, conserved | 0 |
| Tb10.70.7250 | hypothetical protein, conserved | 0 |
| Tb10.70.7420 | hypothetical protein, conserved | 0 |
| Tb10.70.7540 | hypothetical protein, conserved | 0 |
| Tb10.70.7680 | hypothetical protein, conserved | 0 |
| Tb10.70.7760 | hypothetical protein, conserved | 1 |
| Tb10.70.7820 | hypothetical protein, conserved | 0 |
| Tb10.70.7950 | hypothetical protein, conserved | 0 |
| Tb10.6k15.0170 | hypothetical protein, conserved | 2 |
| Tb10.6k15.0320 | hypothetical protein, conserved | 0 |
| Tb10.6k15.0500 | hypothetical protein, conserved | 0 |
| Tb10.6k15.0520 | hypothetical protein, conserved | 0 |
| Tb10.6k15.0710 | hypothetical protein, conserved | 0 |
| Tb10.6k15.1140 | hypothetical protein, conserved | 0 |
| Tb10.6k15.1190 | hypothetical protein, conserved | 0 |

| | | |
|---|---|---|
| Tb10.6k15.1240 | hypothetical protein, conserved | 2 |
| Tb10.6k15.1310 | hypothetical protein, conserved | 0 |
| Tb10.6k15.1400 | hypothetical protein, conserved | 0 |
| Tb10.6k15.1690 | hypothetical protein, conserved | 7 |
| Tb10.6k15.1920 | hypothetical protein, conserved | 0 |
| Tb10.6k15.2100 | hypothetical protein, conserved | 0 |
| Tb10.6k15.2110 | hypothetical protein, conserved | 0 |
| Tb10.6k15.2260 | hypothetical protein, conserved | 1 |
| Tb10.6k15.2510 | hypothetical protein, conserved | 0 |
| Tb10.6k15.2530 | hypothetical protein, conserved | 1 |
| Tb10.6k15.2730 | hypothetical protein, conserved | 0 |
| Tb10.6k15.3480 | hypothetical protein, conserved | 1 |
| Tb10.6k15.4000 | hypothetical protein, conserved | 0 |
| Tb10.61.0200 | hypothetical protein, conserved | 0 |
| Tb10.61.0395 | hypothetical protein, conserved | 0 |
| Tb10.61.1910 | hypothetical protein, conserved | 0 |
| Tb10.61.1930 | hypothetical protein, conserved | 0 |
| Tb10.61.2050 | hypothetical protein, conserved | 0 |
| Tb10.61.2400 | hypothetical protein, conserved | 1 |
| Tb10.61.2420 | hypothetical protein, conserved | 0 |
| Tb10.61.2860 | hypothetical protein, conserved | 3 |
| Tb10.61.2990 | hypothetical protein, conserved | 0 |
| Tb10.61.3000 | hypothetical protein, conserved | 0 |
| Tb10.61.3160 | hypothetical protein, conserved | 0 |
| Tb11.01.0110 | hypothetical protein, conserved | 4 |
| Tb11.01.0500 | hypothetical protein, conserved | 0 |
| Tb11.01.0790 | hypothetical protein, conserved | 0 |
| Tb11.01.0830 | hypothetical protein, conserved | 0 |
| Tb11.01.1510 | hypothetical protein, conserved | 0 |
| Tb11.01.1560 | hypothetical protein, conserved | 0 |
| Tb11.01.1620 | hypothetical protein, conserved | 1 |
| Tb11.01.1850 | hypothetical protein, conserved | 0 |
| Tb11.01.1900 | hypothetical protein, conserved | 1 |
| Tb11.01.2170 | hypothetical protein, conserved | 0 |
| Tb11.01.2260 | hypothetical protein, conserved | 0 |
| Tb11.01.2340 | hypothetical protein, conserved | 0 |

| | | |
|---|---|---|
| Tb11.01.2370 | hypothetical protein, conserved | 0 |
| Tb11.01.2570 | hypothetical protein, conserved | 0 |
| Tb11.01.2620 | hypothetical protein, conserved | 0 |
| Tb11.01.2650 | hypothetical protein, conserved | 1 |
| Tb11.01.2910 | hypothetical protein, conserved | 0 |
| Tb11.01.4140 | hypothetical protein, conserved | 3 |
| Tb11.01.4590 | hypothetical protein, conserved | 0 |
| Tb11.01.4650 | hypothetical protein, conserved | 0 |
| Tb11.01.4700 | hypothetical protein, conserved | 0 |
| Tb11.01.4780 | hypothetical protein, conserved | 0 |
| Tb11.01.4840 | hypothetical protein, conserved | 0 |
| Tb11.01.4990 | hypothetical protein, conserved | 0 |
| Tb11.01.5070 | hypothetical protein, conserved | 0 |
| Tb11.01.5440 | hypothetical protein, conserved | 0 |
| Tb11.01.5460 | hypothetical protein, conserved | 0 |
| Tb11.01.5740 | hypothetical protein, conserved | 0 |
| Tb11.01.6310 | hypothetical protein, conserved | 1 |
| Tb11.01.6380 | hypothetical protein, conserved | 0 |
| Tb11.01.6700 | hypothetical protein, conserved | 0 |
| Tb11.01.7430 | hypothetical protein, conserved | 0 |
| Tb11.01.7730 | hypothetical protein, conserved | 0 |
| Tb11.01.8210 | hypothetical protein, conserved | 0 |
| Tb11.01.8225 | hypothetical protein, conserved | 0 |
| Tb11.01.8226 | hypothetical protein, conserved | 1 |
| Tb11.01.8775 | hypothetical protein, conserved | 2 |
| Tb11.02.0010 | hypothetical protein, conserved | 0 |
| Tb11.02.0160 | hypothetical protein, conserved | 0 |
| Tb11.02.0520 | hypothetical protein, conserved | 8 |
| Tb11.02.0590 | hypothetical protein, conserved | 0 |
| Tb11.02.0950 | hypothetical protein, conserved | 0 |
| Tb11.02.1520 | hypothetical protein, conserved | 0 |
| Tb11.02.1660 | hypothetical protein, conserved | 0 |
| Tb11.02.1730 | hypothetical protein, conserved | 0 |
| Tb11.02.2680 | hypothetical protein, conserved | 0 |
| Tb11.02.3065 | hypothetical protein, conserved | 0 |
| Tb11.02.3310 | hypothetical protein, conserved | 0 |

| | | |
|---|---|---|
| Tb11.02.4160 | hypothetical protein, conserved | 0 |
| Tb11.02.4180 | hypothetical protein, conserved | 0 |
| Tb11.02.4340 | hypothetical protein, conserved | 0 |
| Tb11.02.4680 | hypothetical protein, conserved | 0 |
| Tb11.02.5080 | hypothetical protein, conserved | 2 |
| Tb11.02.5105 | hypothetical protein, conserved | 0 |
| Tb11.02.5330 | hypothetical protein, conserved | 0 |
| Tb11.03.0115 | hypothetical protein, conserved | 0 |
| Tb11.03.0260 | hypothetical protein, conserved | 0 |
| Tb11.03.0300 | hypothetical protein, conserved | 15 |
| Tb11.03.0440 | hypothetical protein, conserved | 1 |
| Tb11.03.0490 | hypothetical protein, conserved | 0 |
| Tb11.03.0725 | hypothetical protein, conserved | 0 |
| Tb11.03.0840 | hypothetical protein, conserved | 0 |
| Tb11.12.0009 | hypothetical protein, conserved | 0 |
| Tb11.22.0002 | hypothetical protein, conserved | 0 |
| Tb11.39.0007 | hypothetical protein, conserved | 0 |
| Tb11.39.0010 | hypothetical protein, conserved | 0 |
| Tb11.47.0005 | hypothetical protein, conserved | 0 |
| Tb11.47.0010 | hypothetical protein, conserved | 0 |
| Tb11.47.0025 | hypothetical protein, conserved | 4 |
| Tb11.50.0001 | hypothetical protein, conserved | 0 |
| Tb11.52.0004 | hypothetical protein, conserved | 0 |
| Tb11.57.0001 | hypothetical protein, conserved | 0 |
| Tb927.1.1820 | hypothetical protein, conserved | 0 |
| Tb927.1.2320 | hypothetical protein, conserved | 0 |
| Tb927.1.3010 | hypothetical protein, conserved | 0 |
| Tb927.1.3310 | hypothetical protein, conserved | 0 |
| Tb927.1.3390 | hypothetical protein, conserved | 0 |
| Tb927.1.3750 | hypothetical protein, conserved | 0 |
| Tb927.1.3850 | hypothetical protein, conserved | 1 |
| Tb927.1.4500 | hypothetical protein, conserved | 6 |
| Tb927.1.4800 | hypothetical protein, conserved | 0 |
| Tb927.1.530 | hypothetical protein, conserved | 0 |
| Tb927.1.650 | hypothetical protein, conserved | 0 |
| Tb927.1.690 | hypothetical protein, conserved | 0 |

| | | |
|---|---|---|
| Tb927.1.730 | hypothetical protein, conserved | 0 |
| Tb927.1.740 | hypothetical protein, conserved | 0 |
| Tb927.1.890 | hypothetical protein, conserved | 1 |
| Tb927.2.1790 | hypothetical protein, conserved | 0 |
| Tb927.2.2570 | hypothetical protein, conserved | 0 |
| Tb927.2.2910 | hypothetical protein, conserved | 0 |
| Tb927.2.4700 | hypothetical protein, conserved | 2 |
| Tb927.2.4830 | hypothetical protein, conserved | 1 |
| Tb927.2.4980 | hypothetical protein, conserved | 0 |
| Tb927.2.5190 | hypothetical protein, conserved | 0 |
| Tb927.3.1090 | hypothetical protein, conserved | 0 |
| Tb927.3.1450 | hypothetical protein, conserved | 2 |
| Tb927.3.1600 | hypothetical protein, conserved | 0 |
| Tb927.3.1690 | hypothetical protein, conserved | 0 |
| Tb927.3.2300 | hypothetical protein, conserved | 0 |
| Tb927.3.2370 | hypothetical protein, conserved | 1 |
| Tb927.3.2390 | hypothetical protein, conserved | 1 |
| Tb927.3.2420 | hypothetical protein, conserved | 2 |
| Tb927.3.3240 | hypothetical protein, conserved | 0 |
| Tb927.3.3430 | hypothetical protein, conserved | 0 |
| Tb927.3.3620 | hypothetical protein, conserved | 0 |
| Tb927.3.3680 | hypothetical protein, conserved | 1 |
| Tb927.3.3750 | hypothetical protein, conserved | 0 |
| Tb927.3.3810 | hypothetical protein, conserved | 2 |
| Tb927.3.3880 | hypothetical protein, conserved | 0 |
| Tb927.3.4130 | hypothetical protein, conserved | 0 |
| Tb927.3.4150 | hypothetical protein, conserved | 0 |
| Tb927.3.4480 | hypothetical protein, conserved | 0 |
| Tb927.3.650 | hypothetical protein, conserved | 1 |
| Tb927.3.730 | hypothetical protein, conserved | 0 |
| Tb927.3.740 | hypothetical protein, conserved | 0 |
| Tb927.3.770 | hypothetical protein, conserved | 0 |
| Tb927.3.850 | hypothetical protein, conserved | 0 |
| Tb927.4.1040 | hypothetical protein, conserved | 0 |
| Tb927.4.1290 | hypothetical protein, conserved | 0 |
| Tb927.4.1370 | hypothetical protein, conserved | 0 |

| | | |
|---|---|---|
| Tb927.4.1380 | hypothetical protein, conserved | 0 |
| Tb927.4.1470 | hypothetical protein, conserved | 0 |
| Tb927.4.1750 | hypothetical protein, conserved | 0 |
| Tb927.4.1880 | hypothetical protein, conserved | 0 |
| Tb927.4.2150 | hypothetical protein, conserved | 0 |
| Tb927.4.2900 | hypothetical protein, conserved | 0 |
| Tb927.4.2960 | hypothetical protein, conserved | 0 |
| Tb927.4.3900 | hypothetical protein, conserved | 0 |
| Tb927.4.4520 | hypothetical protein, conserved | 0 |
| Tb927.4.4540 | hypothetical protein, conserved | 0 |
| Tb927.4.4630 | hypothetical protein, conserved | 0 |
| Tb927.4.4680 | hypothetical protein, conserved | 0 |
| Tb927.4.4710 | hypothetical protein, conserved | 0 |
| Tb927.4.4800 | hypothetical protein, conserved | 2 |
| Tb927.4.5110 | hypothetical protein, conserved | 0 |
| Tb927.4.5230 | hypothetical protein, conserved | 0 |
| Tb927.4.550 | hypothetical protein, conserved | 0 |
| Tb927.4.850 | hypothetical protein, conserved | 0 |
| Tb927.4.970 | hypothetical protein, conserved | 0 |
| Tb927.5.1170 | hypothetical protein, conserved | 0 |
| Tb927.5.1590 | hypothetical protein, conserved | 0 |
| Tb927.5.2610 | hypothetical protein, conserved | 0 |
| Tb927.5.2990 | hypothetical protein, conserved | 0 |
| Tb927.5.3340 | hypothetical protein, conserved | 0 |
| Tb927.5.3740 | hypothetical protein, conserved | 0 |
| Tb927.5.4370 | hypothetical protein, conserved | 0 |
| Tb927.5.4390 | hypothetical protein, conserved | 0 |
| Tb927.5.4450 | hypothetical protein, conserved | 0 |
| Tb927.5.480 | hypothetical protein, conserved | 0 |
| Tb927.5.750 | hypothetical protein, conserved | 1 |
| Tb927.5.970 | hypothetical protein, conserved | 0 |
| Tb927.6.1540 | hypothetical protein, conserved | 5 |
| Tb927.6.1700 | hypothetical protein, conserved | 0 |
| Tb927.6.2160 | hypothetical protein, conserved | 0 |
| Tb927.6.2200 | hypothetical protein, conserved | 0 |
| Tb927.6.2220 | hypothetical protein, conserved | 0 |

| | | |
|---|---|---|
| Tb927.6.2240 | hypothetical protein, conserved | 0 |
| Tb927.6.3060 | hypothetical protein, conserved | 0 |
| Tb927.6.3360 | hypothetical protein, conserved | 0 |
| Tb927.6.3930 | hypothetical protein, conserved | 0 |
| Tb927.6.3950 | hypothetical protein, conserved | 0 |
| Tb927.6.3980 | hypothetical protein, conserved | 0 |
| Tb927.6.4080 | hypothetical protein, conserved | 0 |
| Tb927.6.4720 | hypothetical protein, conserved | 0 |
| Tb927.6.4820 | hypothetical protein, conserved | 0 |
| Tb927.6.5090 | hypothetical protein, conserved | 9 |
| Tb927.7.1160 | hypothetical protein, conserved | 0 |
| Tb927.7.1380 | hypothetical protein, conserved | 0 |
| Tb927.7.1460 | hypothetical protein, conserved | 0 |
| Tb927.7.2180 | hypothetical protein, conserved | 0 |
| Tb927.7.2200 | hypothetical protein, conserved | 0 |
| Tb927.7.2210 | hypothetical protein, conserved | 0 |
| Tb927.7.2290 | hypothetical protein, conserved | 0 |
| Tb927.7.2600 | hypothetical protein, conserved | 0 |
| Tb927.7.2640 | hypothetical protein, conserved | 0 |
| Tb927.7.2960 | hypothetical protein, conserved | 0 |
| Tb927.7.3350 | hypothetical protein, conserved | 4 |
| Tb927.7.3810 | hypothetical protein, conserved | 0 |
| Tb927.7.3920 | hypothetical protein, conserved | 0 |
| Tb927.7.3970 | hypothetical protein, conserved | 0 |
| Tb927.7.4320 | hypothetical protein, conserved | 0 |
| Tb927.7.4860 | hypothetical protein, conserved | 0 |
| Tb927.7.5010 | hypothetical protein, conserved | 0 |
| Tb927.7.5260 | hypothetical protein, conserved | 0 |
| Tb927.7.5640 | hypothetical protein, conserved | 0 |
| Tb927.7.5720 | hypothetical protein, conserved | 0 |
| Tb927.7.5740 | hypothetical protein, conserved | 0 |
| Tb927.7.6180 | hypothetical protein, conserved | 0 |
| Tb927.7.6840 | hypothetical protein, conserved | 0 |
| Tb927.7.6870 | hypothetical protein, conserved | 0 |
| Tb927.7.7010 | hypothetical protein, conserved | 0 |
| Tb927.7.7440 | hypothetical protein, conserved | 0 |

| | | |
|---|---|---|
| Tb927.8.1000 | hypothetical protein, conserved | 0 |
| Tb927.8.1750 | hypothetical protein, conserved | 0 |
| Tb927.8.1900 | hypothetical protein, conserved | 0 |
| Tb927.8.2010 | hypothetical protein, conserved | 0 |
| Tb927.8.2080 | hypothetical protein, conserved | 0 |
| Tb927.8.2140 | hypothetical protein, conserved | 0 |
| Tb927.8.2170 | hypothetical protein, conserved | 1 |
| Tb927.8.2330 | hypothetical protein, conserved | 0 |
| Tb927.8.2420 | hypothetical protein, conserved | 0 |
| Tb927.8.2470 | hypothetical protein, conserved | 0 |
| Tb927.8.2590 | hypothetical protein, conserved | 0 |
| Tb927.8.2700 | hypothetical protein, conserved | 0 |
| Tb927.8.2720 | hypothetical protein, conserved | 0 |
| Tb927.8.3260 | hypothetical protein, conserved | 0 |
| Tb927.8.3600 | hypothetical protein, conserved | 0 |
| Tb927.8.3740 | hypothetical protein, conserved | 0 |
| Tb927.8.3850 | hypothetical protein, conserved | 0 |
| Tb927.8.4420 | hypothetical protein, conserved | 0 |
| Tb927.8.4650 | hypothetical protein, conserved | 0 |
| Tb927.8.4790 | hypothetical protein, conserved | 0 |
| Tb927.8.5350 | hypothetical protein, conserved | 0 |
| Tb927.8.5550 | hypothetical protein, conserved | 0 |
| Tb927.8.5590 | hypothetical protein, conserved | 0 |
| Tb927.8.5800 | hypothetical protein, conserved | 0 |
| Tb927.8.640 | hypothetical protein, conserved | 0 |
| Tb927.8.7790 | hypothetical protein, conserved | 0 |
| Tb927.8.860 | hypothetical protein, conserved | 1 |
| Tb09.211.3240 | hypothetical protein, conserved,leucine-rich repeat protein (LRRP), putative | 0 |
| Tb10.70.4360 | hypothetical protein, conserved,leucine-rich repeat protein (LRRP), putative | 0 |
| Tb927.8.2650 | hypothetical protein, conserved,metallo-beta-lactamase-like protein, putative | 0 |
| Tb10.6k15.2370 | hypothetical protein, conserved,predicted bromodomain protein | 0 |
| Tb10.6k15.3020 | hypothetical protein, conserved,transportin2-like protein | 0 |
| Tb09.160.0270 | hypothetical protein, unlikely | 0 |
| Tb09.160.0290 | hypothetical protein, unlikely | 0 |
| Tb09.160.0320 | hypothetical protein, unlikely | 0 |
| Tb09.160.0640 | hypothetical protein, unlikely | 0 |

| | | |
|---|---|---|
| Tb09.160.1040 | hypothetical protein, unlikely | 0 |
| Tb09.160.1320 | hypothetical protein, unlikely | 0 |
| Tb09.160.1380 | hypothetical protein, unlikely | 0 |
| Tb09.160.1460 | hypothetical protein, unlikely | 0 |
| Tb09.160.1530 | hypothetical protein, unlikely | 0 |
| Tb09.160.1540 | hypothetical protein, unlikely | 0 |
| Tb09.160.1620 | hypothetical protein, unlikely | 0 |
| Tb09.160.1730 | hypothetical protein, unlikely | 0 |
| Tb09.160.1770 | hypothetical protein, unlikely | 0 |
| Tb09.160.1810 | hypothetical protein, unlikely | 0 |
| Tb09.160.1970 | hypothetical protein, unlikely | 0 |
| Tb09.160.2300 | hypothetical protein, unlikely | 0 |
| Tb09.160.2310 | hypothetical protein, unlikely | 0 |
| Tb09.160.2320 | hypothetical protein, unlikely | 0 |
| Tb09.160.2390 | hypothetical protein, unlikely | 0 |
| Tb09.160.2630 | hypothetical protein, unlikely | 0 |
| Tb09.160.2760 | hypothetical protein, unlikely | 0 |
| Tb09.160.2850 | hypothetical protein, unlikely | 0 |
| Tb09.160.2880 | hypothetical protein, unlikely | 1 |
| Tb09.160.3080 | hypothetical protein, unlikely | 2 |
| Tb09.160.3350 | hypothetical protein, unlikely | 0 |
| Tb09.160.3460 | hypothetical protein, unlikely | 0 |
| Tb09.160.3720 | hypothetical protein, unlikely | 1 |
| Tb09.160.3970 | hypothetical protein, unlikely | 0 |
| Tb09.160.4040 | hypothetical protein, unlikely | 0 |
| Tb09.160.4530 | hypothetical protein, unlikely | 3 |
| Tb09.160.4650 | hypothetical protein, unlikely | 0 |
| Tb09.160.4900 | hypothetical protein, unlikely | 0 |
| Tb09.160.4970 | hypothetical protein, unlikely | 0 |
| Tb09.160.5460 | hypothetical protein, unlikely | 3 |
| Tb09.211.0420 | hypothetical protein, unlikely | 1 |
| Tb09.211.1160 | hypothetical protein, unlikely | 0 |
| Tb09.211.1430 | hypothetical protein, unlikely | 0 |
| Tb09.211.2210 | hypothetical protein, unlikely | 0 |
| Tb09.211.2350 | hypothetical protein, unlikely | 1 |
| Tb09.211.2470 | hypothetical protein, unlikely | 0 |

| | | |
|---|---|---|
| Tb09.211.2490 | hypothetical protein, unlikely | 0 |
| Tb09.211.2510 | hypothetical protein, unlikely | 0 |
| Tb09.211.2520 | hypothetical protein, unlikely | 0 |
| Tb09.244.0190 | hypothetical protein, unlikely | 0 |
| Tb09.244.1990 | hypothetical protein, unlikely | 0 |
| Tb09.244.2080 | hypothetical protein, unlikely | 0 |
| Tb09.244.2610 | hypothetical protein, unlikely | 0 |
| Tb09.244.2870 | hypothetical protein, unlikely | 0 |
| Tb10.70.7290 | hypothetical protein, unlikely | 0 |
| Tb10.6k15.0840 | hypothetical protein, unlikely | 0 |
| Tb10.6k15.0870 | hypothetical protein, unlikely | 0 |
| Tb10.61.0130 | hypothetical protein, unlikely | 0 |
| Tb10.61.1800 | hypothetical protein, unlikely | 0 |
| Tb11.01.2980 | hypothetical protein, unlikely | 0 |
| Tb927.1.1090 | hypothetical protein, unlikely | 0 |
| Tb927.1.1260 | hypothetical protein, unlikely | 2 |
| Tb927.1.1490 | hypothetical protein, unlikely | 0 |
| Tb927.1.1860 | hypothetical protein, unlikely | 2 |
| Tb927.1.1950 | hypothetical protein, unlikely | 1 |
| Tb927.1.2000 | hypothetical protein, unlikely | 0 |
| Tb927.1.2080 | hypothetical protein, unlikely | 0 |
| Tb927.1.2200 | hypothetical protein, unlikely | 0 |
| Tb927.1.2250 | hypothetical protein, unlikely | 2 |
| Tb927.1.260 | hypothetical protein, unlikely | 0 |
| Tb927.1.2630 | hypothetical protein, unlikely | 2 |
| Tb927.1.2650 | hypothetical protein, unlikely | 2 |
| Tb927.1.2720 | hypothetical protein, unlikely | 2 |
| Tb927.1.2810 | hypothetical protein, unlikely | 0 |
| Tb927.1.2840 | hypothetical protein, unlikely | 0 |
| Tb927.1.2870 | hypothetical protein, unlikely | 0 |
| Tb927.1.2920 | hypothetical protein, unlikely | 0 |
| Tb927.1.3340 | hypothetical protein, unlikely | 0 |
| Tb927.1.3360 | hypothetical protein, unlikely | 0 |
| Tb927.1.3530 | hypothetical protein, unlikely | 1 |
| Tb927.1.3580 | hypothetical protein, unlikely | 0 |
| Tb927.1.3740 | hypothetical protein, unlikely | 1 |

| | | |
|---|---|---|
| Tb927.1.3770 | hypothetical protein, unlikely | 0 |
| Tb927.1.4080 | hypothetical protein, unlikely | 0 |
| Tb927.1.4090 | hypothetical protein, unlikely | 0 |
| Tb927.1.4170 | hypothetical protein, unlikely | 1 |
| Tb927.1.4240 | hypothetical protein, unlikely | 0 |
| Tb927.1.4930 | hypothetical protein, unlikely | 0 |
| Tb927.1.5190 | hypothetical protein, unlikely | 0 |
| Tb927.1.5290 | hypothetical protein, unlikely | 0 |
| Tb927.1.590 | hypothetical protein, unlikely | 2 |
| Tb927.1.850 | hypothetical protein, unlikely | 0 |
| Tb927.1.920 | hypothetical protein, unlikely | 0 |
| Tb927.1.950 | hypothetical protein, unlikely | 5 |

**SecretomeP prediction 0,8<NNscore>0,7 on T. brucei proteome**

| Accession number | protein name | TMHMM prediction |
|---|---|---|
| Tb10.70.1170 | amino acid tansporter, putative | 10 |
| Tb11.01.7500 | amino acid transporter, putative | 10 |
| Tb11.01.7520 | amino acid transporter, putative | 10 |
| Tb11.01.7590 | amino acid transporter, putative | 11 |
| Tb11.01.7600 | amino acid transporter, putative | 11 |
| Tb927.4.3990 | amino acid transporter, putative | 11 |
| Tb927.4.4000 | amino acid transporter, putative | 11 |
| Tb927.4.4010 | amino acid transporter, putative | 11 |
| Tb927.4.4830 | amino acid transporter, putative | 11 |
| Tb927.4.4850 | amino acid transporter, putative | 11 |
| Tb927.4.4870 | amino acid transporter, putative | 11 |
| Tb927.8.7600 | amino acid transporter, putative | 12 |
| Tb927.8.7650 | amino acid transporter, putative | 11 |
| Tb927.8.7740 | amino acid transporter, putative | 11 |
| Tb927.8.8220 | amino acid transporter, putative | 11 |
| Tb927.8.8240 | amino acid transporter, putative | 11 |
| Tb927.8.8300 | amino acid transporter, putative | 11 |
| Tb10.70.0300 | amino acid transporter, putative,amino acid permease, putative | 11 |

| | | |
|---|---|---|
| Tb927.8.7610 | AATP1 amino acid transporter 1, putative | 11 |
| Tb927.4.4840 | AATP7 amino acid transporter 7, putative | 11 |
| Tb927.4.4860 | AATP8 amino acid transporter 8, putative | 11 |
| Tb10.6k15.2900 | ABC transporter, putative | 5 |
| Tb11.01.8700 | ABCB7 ABC transporter, putative | 7 |
| Tb927.4.4050 | ABCD3; PMP70 ABC transporter, putative,70 kDa peroxisomal membrane protein | 1 |
| Tb927.4.2290 | glucose transporter, putative | 11 |
| Tb11.01.0420 | protein transport protein sec13, putative | 0 |
| Tb927.8.3660 | protein transport protein Sec23, putative | 0 |
| Tb10.6k15.2020 | THT2A glucose transporter,glucose transporter 2A | 12 |
| Tb10.61.2650 | aquaglyceroporin (small solute channel), putative | 6 |
| Tb10.61.2640 | aquaporin 9, putative | 6 |
| Tb927.5.3400 | calcium-translocating P-type ATPase,calcium pump | 10 |
| Tb10.70.4720 | importin beta-1 subunit, putative | 0 |
| Tb927.6.4740 | importin-alpha re-exporter protein, putative,cellular apoptosis susceptibility protein, putative | 0 |
| Tb10.389.1800 | syntaxin, putative,vesicle-associated membrane protein, putative | 0 |
| Tb927.6.180 | receptor-type adenylate cyclase GRESAG 4, putative | 0 |
| Tb09.160.5300 | ARL1B ADP-ribosylation factor-like protein, putative | 0 |
| Tb10.6k15.1960 | ARL3 ADP-ribosylation factor 3, putative,ADP-ribosylation factor-like protein, putative | 0 |
| Tb10.70.3000 | ARL8B ADP-ribosylation factor 6, putative,ADP-ribosylation factor-like protein, putative | 0 |
| Tb927.3.5330 | Cnt-b5 ADP-ribosylation factor GTPase activating protein, putative,centaurin beta 5 | 0 |
| Tb927.7.1780 | adenine phosphoribosyltransferase, putative | 0 |
| Tb927.7.4480 | adenosine 5'-monophosphoramidase, putative | 0 |
| Tb09.211.0350 | adenylate kinase, putative | 0 |
| Tb10.70.5150 | adenylate kinase, putative | 0 |
| Tb927.6.3650 | ADP-ribosylation factor, putative | 0 |
| Tb927.3.3450 | ADP-ribosylation factor-like protein 3A, putative | 0 |
| Tb10.26.0200 | guanylate kinase, putative | 0 |
| Tb10.61.0150 | inosine-5'-monophosphate dehydrogenase,IMP dehydrogenase | 0 |
| Tb927.7.7480 | trans-sialidase, putative | 2 |
| Tb927.7.2070 | heat shock protein DnaJ, putative | 0 |
| Tb09.211.2290 | chaperone protein DNAJ, putative | 0 |
| Tb09.211.4720 | chaperone protein DNAJ, putative | 0 |
| Tb10.389.1150 | chaperone protein DNAJ, putative | 0 |

| | | |
|---|---|---|
| Tb10.6k15.0460 | chaperone protein DNAJ, putative | 0 |
| Tb927.8.4470 | chaperone protein DNAJ, putative | 0 |
| Tb927.5.580 | prefoldin subunit, putative | 0 |
| Tb927.7.570 | prefoldin, putative | 0 |
| Tb927.8.6910 | cyclophilin, putative | 0 |
| Tb927.8.2090 | PPIase cyclophilin type peptidyl-prolyl cis-trans isomerase, putative | 0 |
| Tb09.211.4880 | PPIase cyclophilin-like protein, putative | 1 |
| Tb927.8.1620 | major surface protease gp63, putative,GP63, putative,metallopeptidase, putative | 1 |
| Tb927.8.1630 | major surface protease gp63, putative,GP63, putative,metallopeptidase, putative | 1 |
| Tb927.8.1640 | major surface protease gp63, putative,GP63, putative,metallopeptidase, putative | 1 |
| Tb11.02.0730 | mca1 metacaspase,cysteine peptidase, Clan CD, family C13 | 1 |
| Tb927.2.3440 | D-alanyl-glycyl endopeptidase-like protein,cysteine peptidase, Clan CA, family C51, putative | 1 |
| Tb10.61.1870 | aminopeptidase, putative,metallo-peptidase, Clan MG, Family M24 | 0 |
| Tb927.1.2160 | calpain-like protein fragment, putative | 0 |
| Tb927.1.2150 | calpain-like protein fragment, putative,calpain-like cysteine peptidase, Clan CA, family C2 | 0 |
| Tb927.8.6450 | ICP inhibitor of cysteine peptidase,chagasin family (I42) | 0 |
| Tb927.7.1140 | GPX3 trypanothione/tryparedoxin dependent peroxidase 3,glutathione peroxidase-like protein 3 | 0 |
| Tb11.01.7560 | glutathione peroxidase, putative | 0 |
| Tb927.6.1080 | glxII hydroxyacylglutathione hydrolase, putative,glyoxalase II | 0 |
| Tb927.3.3760 | TRYP1 tryparedoxin | 0 |
| Tb927.8.1990 | TRYP2 tryparedoxin peroxidase | 0 |
| Tb09.244.2800 | trypanin-related protein, putative | 0 |
| Tb927.3.3780 | tryparedoxin | 0 |
| Tb927.4.3390 | tuzin, putative | 1 |
| Tb11.01.1320 | oxidoreductase, putative | 0 |
| Tb927.7.7410 | oxidoreductase, putative | 0 |
| Tb927.3.2410 | peroxisome assembly protein, putative | 0 |
| Tb10.100.0130 | PEX14 peroxin 14, putative | 0 |
| Tb11.03.0950 | electron transfer flavoprotein, putative | 0 |
| Tb927.7.890 | electron transfer protein, putative | 0 |
| Tb927.8.3380 | electron transfer protein, putative | 0 |
| Tb10.389.1140 | electron transfer protein, putative,ferredoxin, putative | 0 |
| Tb927.6.3320 | ferric reductase transmembrane protein, putative | 6 |
| Tb09.244.2060 | expression site-associated gene (ESAG) protein, putative,expression site-associated gene 3 (ESAG3) protein, putative | 1 |

| | | |
|---|---|---|
| Tb927.5.3620 | dual-specificity protein phosphatase, putative | 0 |
| Tb10.389.0490 | PKA3 protein kinase A catalytic subunit | 0 |
| Tb927.4.3560 | serine/threonine protein phosphatase PP1, putative | 0 |
| Tb11.02.5220 | phopshatase, putative | 0 |
| Tb927.4.3610 | serine/threonine-protein phosphatase PP1, pseudogene, putative | 0 |
| Tb09.160.1780 | protein kinase, putative | 2 |
| Tb11.03.0290 | protein kinase, putative | 0 |
| Tb927.8.5500 | protein kinase, putative | 0 |
| Tb10.70.5340 | protein kinase, putative,casein kinase I, putative | 0 |
| Tb10.70.5920 | protein kinase, putative,serine/threonine protein kinase, putative | 1 |
| Tb927.8.5780 | protein tyrosine phosphatase, putative | 0 |
| Tb11.01.4270 | tyrosine phosphatase, putative | 0 |
| Tb11.01.7400 | GPI transamidase component Tta1 | 2 |
| Tb927.6.3000 | fatty acid desaturase, putative,sphingolipid delta 4 desaturase, putative | 5 |
| Tb927.5.3710 | Sphingomyelin phosphodiesterase, putative | 2 |
| Tb927.5.2690 | IMPase 1 inositol-1(or 4)-monophosphatase 1, putative | 0 |
| Tb927.8.7170 | inositol polyphosphate 1-phosphatase, putative | 0 |
| Tb09.160.0530 | phosphatidyltransferase, putative,phosphatidylinositol synthase, putative | 3 |
| Tb927.4.1510 | phospholipid-translocating ATPase, putative | 10 |
| Tb10.70.3220 | serine palmitoyltransferase, putative | 1 |
| Tb927.7.5820 | monooxygenase, putative | 0 |
| Tb927.3.3870 | lipase domain protein, putative | 7 |
| Tb927.1.2740 | lipase-like protein, putative | 1 |
| Tb927.1.3000 | aminoacylase, putative,N-acyl-L-amino acid amidohydrolase, putative | 0 |
| Tb927.4.2010 | ACBP acyl-CoA binding protein, putative | 0 |
| Tb09.211.3020 | acyl transferase-like protein | 0 |
| Tb927.5.890 | oligosaccharyl transferase subunit, putative | 10 |
| Tb927.5.910 | oligosaccharyl transferase subunit, putative | 10 |
| Tb927.8.8090 | UDP-Gal or UDP-GlcNAc-dependent glycosyltransferase, putative | 1 |
| Tb927.8.8100 | UDP-Gal or UDP-GlcNAc-dependent glycosyltransferase, putative | 1 |
| Tb927.5.2380 | hydrolase, alpha/beta fold family, putative | 0 |
| Tb11.01.0120 | haloacid dehalogenase-like hydrolase, putative | 0 |
| Tb09.160.4380 | succinate dehydrogenase, putative | 0 |
| Tb09.160.2010 | N-acetyltransferase, putative | 0 |

| | | |
|---|---|---|
| Tb927.7.2530 | N-acetyltransferase, putative | 0 |
| Tb10.6k15.3230 | GTP-binding protein, putative | 0 |
| Tb10.6k15.1160 | G-actin binding protein, putative,CAP/Srv2p, putative | 0 |
| Tb927.2.2130 | small GTP-binding protein RAB6, putative | 0 |
| Tb10.70.6420 | RAB21 small GTPase, putative,Ras-related protein, putative | 0 |
| Tb10.389.1550 | RAB-interacting protein, putative | 2 |
| Tb09.160.4520 | calmodulin, putative | 0 |
| Tb09.211.4511 | kinetoplastid membrane protein KMP-11 | 0 |
| Tb09.211.4512 | kinetoplastid membrane protein KMP-11 | 0 |
| Tb09.211.4513 | kinetoplastid membrane protein KMP-11 | 0 |
| Tb927.4.4980 | adrenodoxin precursor, putative | 0 |
| Tb927.8.4180 | deaminase, putative | 0 |
| Tb927.4.4320 | divalent cation tolerance protein, putative | 0 |
| Tb927.1.4100 | COXIV cytochrome oxidase subunit IV | 0 |
| Tb11.02.4485 | CYB5 cytochrome b5, putative | 1 |
| Tb11.01.5660 | CYC2 cyclin 2,G1 cyclin | 0 |
| Tb11.01.3805 | CAP15 microtubule-associated protein,corset-associated protein 15 | 0 |
| Tb927.8.1080 | centrin, putative,caltractin, putative | 0 |
| Tb10.406.0320 | ARP2/3 complex 16kDa subunit, putative,ARP2/3 complex subunit, putative | 0 |
| Tb927.2.2900 | ARP2/3 complex subunit, putative | 0 |
| Tb11.01.2030 | golgi SNARE protein-like,GOLGI SNAP receptor complex member, putative | 1 |
| Tb927.8.2310 | (H+)-ATPase G subunit, putative | 0 |
| Tb10.70.2900 | 2-oxoisovalerate dehydrogenase beta subunit, mitochondrial precursor, putative | 0 |
| Tb927.3.5630 | 3,2-trans-enoyl-CoA isomerase, mitochondrial precursor, putative | 0 |
| Tb10.70.7560 | TAX-2,flagellar protein | 0 |
| Tb11.01.0680 | HERTS,flagellar component | 0 |
| Tb927.7.3840 | kinesin-like protein, fragment, putative | 0 |
| Tb927.7.5580 | MOB1B cell cycle associated protein MOB1, putative | 0 |
| Tb10.70.5120 | malate dehydrogenase, putative | 0 |
| Tb11.01.6120 | polypeptide deformylase, putative | 0 |
| Tb927.7.3470 | p22 protein precursor, putative | 0 |
| Tb11.01.5350 | profilin | 0 |
| Tb927.3.4890 | ubiquinone biosynthesis protein COQ7 homolog, putative | 0 |
| Tb11.01.5180 | ubiquinone biosynthesis protein-like protein | 2 |

| | | |
|---|---|---|
| Tb927.7.2440 | pyrroline-5-carboxylate reductase, putative | 0 |
| Tb09.160.2150 | Lsm6 U6 snRNA-associated Sm-like protein LSm6p,Sm-like protein | 0 |
| Tb09.211.3970 | ankyrin repeat protein, putative | 0 |
| Tb927.1.630 | anti-silencing protein ASF 1 like protein, putative | 0 |
| Tb11.01.5260 | radial spoke protein RSP11, putative | 0 |
| Tb11.47.0034 | radial spoke protein RSP3, putative | 0 |
| Tb11.01.4600 | cleavage and polyadenylation specificity factor 30 kDa subunit,CPSF 30 kDa subunit,NS1 effector domain-binding protein 1 | 0 |
| Tb11.02.2620 | ATP-dependent DEAD/H RNA helicase, putative,ATP-dependent RNA helicase, putative | 0 |
| Tb927.8.3830 | ATP-dependent DEAD/H RNA helicase, putative,mitochondrial | 0 |
| Tb927.8.4040 | endonuclease G, putative | 1 |
| Tb927.8.4090 | endonuclease G, putative | 1 |
| Tb11.01.3910 | endonuclease III, putative | 0 |
| Tb927.3.5100 | DNA repair helicase and transcription factor protein, putative,Transcription factor II H complex, XPB subunit, putative | 0 |
| Tb09.211.3040 | DNA-repair protein, putative | 0 |
| Tb10.70.2100 | KREPA4 RNA-editing complex protein,RNA-binding protein,KREPA4 | 0 |
| Tb927.8.680 | KREPA5 RNA-editing complex protein | 0 |
| Tb11.02.0490 | KREPB4 RNA-editing complex protein,KREPB4 | 0 |
| Tb927.3.3990 | KREPB6 RNA-editing complex protein,KREPB6 | 0 |
| Tb927.7.880 | RNA-binding protein, putative | 0 |
| Tb927.8.6440 | RNA-binding protein, putative | 0 |
| Tb09.211.4540 | RNA-binding protein, putative,DRBD2 | 0 |
| Tb11.02.3610 | RNA-binding protein, putative,RBP38 | 0 |
| Tb11.v4.0004 | RNR2 ribonucleoside-diphosphate reductase small chain | 1 |
| Tb11.v4.0005 | RNR2 ribonucleoside-diphosphate reductase small chain | 1 |
| Tb11.v4.0006 | RNR2 ribonucleoside-diphosphate reductase small chain | 1 |
| Tb927.6.3130 | queuine tRNA-ribosyltransferase, putative | 0 |
| Tb11.02.5060 | SNF2 DNA repair protein, putative | 0 |
| Tb927.3.1050 | transposase of Tn10 | 0 |
| Tb927.6.5810 | transposase of Tn10 | 0 |
| Tb11.01.4860 | guide RNA binding protein | 0 |
| Tb927.7.5760 | NFT2 nuclear transport factor 2 protein, putative | 0 |
| Tb927.6.2130 | nuclear movement protein, putative,NUDC-like protein | 0 |
| Tb11.01.1270 | nucleic acid binding protein, putative | 0 |
| Tb09.211.3620 | nucleic acid binding protein, putative,predicted zinc finger protein | 0 |

| | | |
|---|---|---|
| Tb10.70.2465 | nucleolar RNA-binding protein, putative | 0 |
| Tb10.6k15.1480 | kinetoplast DNA-associated protein, putative | 1 |
| Tb10.70.1100 | translation elongation factor 1-beta, putative | 0 |
| Tb10.61.0330 | TFIID-like protein, putative | 0 |
| Tb11.02.2600 | TFIIS1 transcription elongation factor S-II, putative | 0 |
| Tb927.2.3580 | TFIIS2-1 transcription elongation factor S-II, putative | 0 |
| Tb927.8.5880 | eukaryotic translation initiation factor 1A, putative | 0 |
| Tb10.61.0210 | Eukaryotic translation initiation factor 4E, putative | 0 |
| Tb927.6.1870 | eukaryotic translation initiation factor 4E, putative | 0 |
| Tb927.3.1250 | RPB10 DNA-directed RNA polymerase subunit, putative,RNA polymerase subunit, putative | 0 |
| Tb11.57.0004 | RPB11 DNA-directed RNA polymerase II, putative,RNA polymerase subunit, putative | 0 |
| Tb927.3.5500 | RPB3 DNA-directed RNA polymerase II subunit 3, putative,RNA polymerase subunit, putative | 0 |
| Tb10.389.0120 | RPB5z DNA-directed RNA polymerase II, putative,RNA polymerase subunit, putative | 0 |
| Tb10.6k15.3190 | RBP15 RNA-binding protein, putative,RBP15 | 0 |
| Tb11.01.3915 | RBP5 RNA-binding protein, putative,RBP5 | 0 |
| Tb927.6.4350 | ribosomal RNA assembly protein, putative | 1 |
| Tb927.6.5140 | ribosomal P protein AGP2beta-1, putative | 0 |
| Tb11.50.0005 | ribosomal protein L21E (60S), putative | 0 |
| Tb927.4.1100 | ribosomal protein L21E (60S), putative | 0 |
| Tb927.4.1800 | ribosomal protein L3, mitochondrial, putative | 0 |
| Tb927.4.1860 | ribosomal protein S19, putative | 0 |
| Tb927.2.6090 | RPL44 60S ribosomal protein L44,ribosomal protein L36a.e | 0 |
| Tb10.70.1730 | RPS18 40S ribosomal protein S18, putative | 0 |
| Tb10.61.1390 | 40S ribosomal protein S13, putative | 0 |
| Tb927.2.5910 | 40S ribosomal protein S13, putative | 0 |
| Tb11.01.3675 | 40S ribosomal protein S17, putative | 0 |
| Tb11.01.3676 | 40S ribosomal protein S17, putative | 0 |
| Tb10.70.1740 | 40S ribosomal protein S18, putative | 0 |
| Tb927.6.2100 | 40S ribosomal protein S30, putative | 0 |
| Tb927.6.2110 | 40S ribosomal protein S30, putative | 0 |
| Tb927.7.230 | 40S ribosomal protein S33, putative | 0 |
| Tb927.7.240 | 40S ribosomal protein S33, putative | 0 |
| Tb11.02.4170 | 40S ribosomal protein S5, putative | 0 |
| Tb927.4.1070 | 50S ribosomal protein L13, putative | 0 |

| | | |
|---|---|---|
| Tb10.61.2090 | 60S ribosomal protein L17, putative | 0 |
| Tb11.02.2430 | 60S ribosomal protein L17, putative | 0 |
| Tb11.01.7960 | 60S ribosomal protein L2, putative,60S ribosomal protein L8, putative | 0 |
| Tb927.5.1110 | 60S ribosomal protein L2, putative,60S ribosomal protein L8, putative | 0 |
| Tb11.01.7535 | 60S ribosomal protein L27, putative | 0 |
| Tb11.01.7545 | 60S ribosomal protein L27, putative | 0 |
| Tb11.01.1790 | 60S ribosomal protein L29, putative | 0 |
| Tb10.389.0910 | 60S ribosomal protein L34, putative | 0 |
| Tb09.211.0865 | 60S ribosomal protein L37, putative | 0 |
| Tb11.02.0500 | 60S ribosomal protein L37, putative | 0 |
| Tb10.100.0110 | 60S ribosomal protein L37a, putative | 0 |
| Tb09.160.0815 | 60S ribosomal protein L38, putative | 0 |
| Tb11.02.0740 | 60S ribosomal protein L44 | 0 |
| Tb10.70.7010 | 60S ribosomal protein L9, putative | 0 |
| Tb927.6.4690 | 60S ribosomal protein L9, putative | 0 |
| Tb927.7.4550 | 60S ribosomal protein-like | 0 |
| Tb10.70.0465 | 60S ribosomal proteins L37, putative | 0 |
| Tb927.7.2820 | histone H2A, putative | 0 |
| Tb927.7.2830 | histone H2A, putative | 0 |
| Tb927.7.2840 | histone H2A, putative | 0 |
| Tb927.7.2850 | histone H2A, putative | 0 |
| Tb927.7.2860 | histone H2A, putative | 0 |
| Tb927.7.2870 | histone H2A, putative | 0 |
| Tb927.7.2880 | histone H2A, putative | 0 |
| Tb927.7.2890 | histone H2A, putative | 0 |
| Tb927.7.2900 | histone H2A, putative | 0 |
| Tb927.7.2910 | histone H2A, putative | 0 |
| Tb927.7.2920 | histone H2A, putative | 0 |
| Tb927.7.2930 | histone H2A, putative | 0 |
| Tb927.7.2940 | histone H2A, putative | 0 |
| Tb927.1.2430 | histone H3, putative | 0 |
| Tb927.1.2450 | histone H3, putative | 0 |
| Tb927.1.2470 | histone H3, putative | 0 |
| Tb927.1.2490 | histone H3, putative | 0 |

| | | |
|---|---|---|
| Tb927.1.2510 | histone H3, putative | 0 |
| Tb927.1.2530 | histone H3, putative | 0 |
| Tb927.1.2550 | histone H3, putative | 0 |
| Tb10.61.1090 | h3vaR histone H3 variant | 0 |
| Tb10.70.6220 | DAC1 histone deacetylase 1,histone deacetylase-like 1 protein | 0 |
| Tb10.26.0070 | 33 kDa inner dynein arm light chain, axonemal, putative,dynein light chain, putative | 0 |
| Tb10.70.6960 | dynein arm light chain, axonemal, putative | 0 |
| Tb927.4.5370 | dynein light chain 2B, cytoplasmic, putative,predicted dynein modulator,roadblock/LC7 family member | 0 |
| Tb927.8.6950 | dynein light chain 2B, cytoplasmic, putative,predicted dynein modulator,roadblock/LC7 family member | 0 |
| Tb10.61.0770 | dynein light chain, putative | 0 |
| Tb10.70.0090 | dynein light chain, putative | 0 |
| Tb11.02.3390 | dynein light chain, putative | 0 |
| Tb927.5.4440 | dynein light chain, putative | 0 |
| Tb10.389.0350 | dynein light chain, putative,dynein light chain 2B, cytoplasmic, putative,predicted dynein modulator,roadblock/LC7 family member | 0 |
| Tb11.02.5620 | dynein light chain, putative,dynein Tctex2 family, putative | 0 |
| Tb11.01.8570 | dynein light intermediate chain D1bLIC, putative | 0 |
| Tb10.6k15.2770 | COP9 signalosome complex subunit, putative,predicted PCI domain protein | 1 |
| Tb927.8.4830 | U1 small nuclear ribonucleoprotein, putative | 0 |
| Tb11.01.6080 | ubiquitin carboxyl-terminal hydrolase, putative | 0 |
| Tb927.4.3790 | ubiquitin carboxyl-terminal hydrolase, putative,cysteine peptidase, Clan CA, family C19, putative | 0 |
| Tb927.4.2710 | ubiquitin-conjugating enzyme E2, putative,ubiquitin carrier protein, putative,ubiquitin-protein ligase, putative | 0 |
| Tb927.8.6090 | ubiquitin-conjugating enzyme E2, putative,ubiquitin-protein ligase, putative,ubiquitin carrier protein, putative | 0 |
| Tb10.70.5480 | ubiquitin-conjugating enzyme variant Kua homologue, putative | 3 |
| Tb927.6.1090 | RPT3 proteasome regulatory ATPase subunit 3 | 0 |
| Tb927.7.1840 | zinc finger protein, putative | 0 |
| Tb927.7.1880 | zinc finger protein, putative | 0 |
| Tb10.05.0130 | hypothetical protein,hypothetical protein | 2 |
| Tb09.142.0350 | hypothetical protein | 0 |
| Tb09.160.1550 | hypothetical protein | 0 |
| Tb09.211.0380 | hypothetical protein | 0 |
| Tb09.211.2160 | hypothetical protein | 0 |
| Tb09.244.1960 | hypothetical protein | 0 |
| Tb09.244.2050 | hypothetical protein | 0 |
| Tb09.354.0050 | hypothetical protein | 0 |

| | | |
|---|---|---|
| Tb10.389.1420 | hypothetical protein | 0 |
| Tb10.61.0120 | hypothetical protein | 0 |
| Tb10.61.0270 | hypothetical protein | 0 |
| Tb10.61.2800 | hypothetical protein | 0 |
| Tb10.6k15.0790 | hypothetical protein | 0 |
| Tb10.6k15.2990 | hypothetical protein | 0 |
| Tb10.70.0410 | hypothetical protein | 0 |
| Tb10.70.1270 | hypothetical protein | 0 |
| Tb10.70.1930 | hypothetical protein | 0 |
| Tb10.70.3060 | hypothetical protein | 0 |
| Tb10.70.6130 | hypothetical protein | 0 |
| Tb11.01.0210 | hypothetical protein | 3 |
| Tb11.01.1180 | hypothetical protein | 0 |
| Tb11.01.4710 | hypothetical protein | 0 |
| Tb11.02.0080 | hypothetical protein | 2 |
| Tb11.03.0320 | hypothetical protein | 1 |
| Tb11.03.0420 | hypothetical protein | 0 |
| Tb11.12.0018 | hypothetical protein | 0 |
| Tb927.1.2420 | hypothetical protein | 0 |
| Tb927.2.3330 | hypothetical protein | 0 |
| Tb927.2.5720 | hypothetical protein | 0 |
| Tb927.3.4180 | hypothetical protein | 0 |
| Tb927.3.5740 | hypothetical protein | 0 |
| Tb927.4.3650 | hypothetical protein | 0 |
| Tb927.4.5060 | hypothetical protein | 0 |
| Tb927.4.5220 | hypothetical protein | 0 |
| Tb927.4.5440 | hypothetical protein | 0 |
| Tb927.5.1380 | hypothetical protein | 0 |
| Tb927.5.1400 | hypothetical protein | 0 |
| Tb927.5.1420 | hypothetical protein | 0 |
| Tb927.6.810 | hypothetical protein | 0 |
| Tb927.7.7370 | hypothetical protein | 0 |
| Tb09.142.0400 | hypothetical protein, conserved | 0 |
| Tb09.160.0360 | hypothetical protein, conserved | 0 |

| | | |
|---|---|---|
| Tb09.160.0380 | hypothetical protein, conserved | 0 |
| Tb09.160.0510 | hypothetical protein, conserved | 0 |
| Tb09.160.0740 | hypothetical protein, conserved | 0 |
| Tb09.160.0790 | hypothetical protein, conserved | 0 |
| Tb09.160.1000 | hypothetical protein, conserved | 0 |
| Tb09.160.1070 | hypothetical protein, conserved | 1 |
| Tb09.160.1110 | hypothetical protein, conserved | 0 |
| Tb09.160.1260 | hypothetical protein, conserved | 0 |
| Tb09.160.1710 | hypothetical protein, conserved | 0 |
| Tb09.160.1760 | hypothetical protein, conserved | 0 |
| Tb09.160.1990 | hypothetical protein, conserved | 0 |
| Tb09.160.2040 | hypothetical protein, conserved | 0 |
| Tb09.160.2220 | hypothetical protein, conserved | 0 |
| Tb09.160.2230 | hypothetical protein, conserved | 1 |
| Tb09.160.2250 | hypothetical protein, conserved | 0 |
| Tb09.160.2350 | hypothetical protein, conserved | 0 |
| Tb09.160.3370 | hypothetical protein, conserved | 0 |
| Tb09.160.3530 | hypothetical protein, conserved | 0 |
| Tb09.160.3740 | hypothetical protein, conserved | 0 |
| Tb09.160.4270 | hypothetical protein, conserved | 1 |
| Tb09.160.4610 | hypothetical protein, conserved | 0 |
| Tb09.160.4720 | hypothetical protein, conserved | 0 |
| Tb09.160.5050 | hypothetical protein, conserved | 0 |
| Tb09.160.5320 | hypothetical protein, conserved | 0 |
| Tb09.211.0170 | hypothetical protein, conserved | 0 |
| Tb09.211.0200 | hypothetical protein, conserved | 0 |
| Tb09.211.0300 | hypothetical protein, conserved | 0 |
| Tb09.211.0520 | hypothetical protein, conserved | 0 |
| Tb09.211.0600 | hypothetical protein, conserved | 0 |
| Tb09.211.0760 | hypothetical protein, conserved | 0 |
| Tb09.211.1050 | hypothetical protein, conserved | 3 |
| Tb09.211.1630 | hypothetical protein, conserved | 0 |
| Tb09.211.2090 | hypothetical protein, conserved | 0 |
| Tb09.211.2460 | hypothetical protein, conserved | 1 |

| | | |
|---|---|---|
| Tb09.211.2680 | hypothetical protein, conserved | 0 |
| Tb09.211.2750 | hypothetical protein, conserved | 0 |
| Tb09.211.3080 | hypothetical protein, conserved | 0 |
| Tb09.211.3150 | hypothetical protein, conserved | 0 |
| Tb09.211.3780 | hypothetical protein, conserved | 0 |
| Tb09.211.3840 | hypothetical protein, conserved | 0 |
| Tb09.211.4020 | hypothetical protein, conserved | 0 |
| Tb09.211.4150 | hypothetical protein, conserved | 0 |
| Tb09.211.4260 | hypothetical protein, conserved | 2 |
| Tb09.211.4290 | hypothetical protein, conserved | 0 |
| Tb09.211.4510 | hypothetical protein, conserved | 0 |
| Tb09.211.4670 | hypothetical protein, conserved | 0 |
| Tb09.211.4690 | hypothetical protein, conserved | 0 |
| Tb09.244.0440 | hypothetical protein, conserved | 0 |
| Tb09.244.2170 | hypothetical protein, conserved | 0 |
| Tb09.244.2620 | hypothetical protein, conserved | 0 |
| Tb09.244.2680 | hypothetical protein, conserved | 0 |
| Tb09.244.2710 | hypothetical protein, conserved | 0 |
| Tb09.v1.0030 | hypothetical protein, conserved | 0 |
| Tb09.v1.0400 | hypothetical protein, conserved | 0 |
| Tb10.26.0350 | hypothetical protein, conserved | 0 |
| Tb10.26.0360 | hypothetical protein, conserved | 0 |
| Tb10.26.0400 | hypothetical protein, conserved | 0 |
| Tb10.26.0600 | hypothetical protein, conserved | 0 |
| Tb10.26.0780 | hypothetical protein, conserved | 0 |
| Tb10.26.1010 | hypothetical protein, conserved | 0 |
| Tb10.26.1060 | hypothetical protein, conserved | 0 |
| Tb10.389.0140 | hypothetical protein, conserved | 8 |
| Tb10.389.0300 | hypothetical protein, conserved | 8 |
| Tb10.389.0540 | hypothetical protein, conserved | 0 |
| Tb10.389.0920 | hypothetical protein, conserved | 0 |
| Tb10.389.1320 | hypothetical protein, conserved | 0 |
| Tb10.389.1390 | hypothetical protein, conserved | 0 |
| Tb10.389.1520 | hypothetical protein, conserved | 0 |

| | | |
|---|---|---|
| Tb10.389.1580 | hypothetical protein, conserved | 0 |
| Tb10.389.1600 | hypothetical protein, conserved | 11 |
| Tb10.389.1630 | hypothetical protein, conserved | 0 |
| Tb10.406.0170 | hypothetical protein, conserved | 4 |
| Tb10.406.0210 | hypothetical protein, conserved | 0 |
| Tb10.406.0265 | hypothetical protein, conserved | 1 |
| Tb10.406.0270 | hypothetical protein, conserved | 0 |
| Tb10.406.0280 | hypothetical protein, conserved | 0 |
| Tb10.61.0220 | hypothetical protein, conserved | 0 |
| Tb10.61.0490 | hypothetical protein, conserved | 0 |
| Tb10.61.0940 | hypothetical protein, conserved | 0 |
| Tb10.61.1300 | hypothetical protein, conserved | 0 |
| Tb10.61.1350 | hypothetical protein, conserved | 0 |
| Tb10.61.1370 | hypothetical protein, conserved | 0 |
| Tb10.61.1730 | hypothetical protein, conserved | 0 |
| Tb10.61.1840 | hypothetical protein, conserved | 0 |
| Tb10.61.2190 | hypothetical protein, conserved | 0 |
| Tb10.61.2600 | hypothetical protein, conserved | 0 |
| Tb10.61.3040 | hypothetical protein, conserved | 0 |
| Tb10.61.3080 | hypothetical protein, conserved | 0 |
| Tb10.61.3110 | hypothetical protein, conserved | 0 |
| Tb10.61.3155 | hypothetical protein, conserved | 0 |
| Tb10.6k15.0100 | hypothetical protein, conserved | 0 |
| Tb10.6k15.0120 | hypothetical protein, conserved | 1 |
| Tb10.6k15.0600 | hypothetical protein, conserved | 0 |
| Tb10.6k15.0780 | hypothetical protein, conserved | 0 |
| Tb10.6k15.1080 | hypothetical protein, conserved | 0 |
| Tb10.6k15.1090 | hypothetical protein, conserved | 7 |
| Tb10.6k15.1330 | hypothetical protein, conserved | 0 |
| Tb10.6k15.1440 | hypothetical protein, conserved | 0 |
| Tb10.6k15.1860 | hypothetical protein, conserved | 0 |
| Tb10.6k15.2070 | hypothetical protein, conserved | 0 |
| Tb10.6k15.2380 | hypothetical protein, conserved | 0 |
| Tb10.6k15.2430 | hypothetical protein, conserved | 0 |

| | | |
|---|---|---|
| Tb10.6k15.2490 | hypothetical protein, conserved | 0 |
| Tb10.6k15.2660 | hypothetical protein, conserved | 0 |
| Tb10.6k15.2690 | hypothetical protein, conserved | 0 |
| Tb10.6k15.2870 | hypothetical protein, conserved | 0 |
| Tb10.6k15.3060 | hypothetical protein, conserved | 0 |
| Tb10.6k15.3090 | hypothetical protein, conserved | 0 |
| Tb10.6k15.3280 | hypothetical protein, conserved | 0 |
| Tb10.6k15.3760 | hypothetical protein, conserved | 0 |
| Tb10.70.0020 | hypothetical protein, conserved | 11 |
| Tb10.70.0040 | hypothetical protein, conserved | 11 |
| Tb10.70.0050 | hypothetical protein, conserved | 0 |
| Tb10.70.0190 | hypothetical protein, conserved | 0 |
| Tb10.70.0210 | hypothetical protein, conserved | 0 |
| Tb10.70.0530 | hypothetical protein, conserved | 0 |
| Tb10.70.0910 | hypothetical protein, conserved | 0 |
| Tb10.70.1070 | hypothetical protein, conserved | 0 |
| Tb10.70.1140 | hypothetical protein, conserved | 0 |
| Tb10.70.1250 | hypothetical protein, conserved | 0 |
| Tb10.70.1390 | hypothetical protein, conserved | 0 |
| Tb10.70.1470 | hypothetical protein, conserved | 3 |
| Tb10.70.1550 | hypothetical protein, conserved | 2 |
| Tb10.70.2200 | hypothetical protein, conserved | 0 |
| Tb10.70.2340 | hypothetical protein, conserved | 0 |
| Tb10.70.2400 | hypothetical protein, conserved | 0 |
| Tb10.70.2460 | hypothetical protein, conserved | 0 |
| Tb10.70.2750 | hypothetical protein, conserved | 1 |
| Tb10.70.2950 | hypothetical protein, conserved | 0 |
| Tb10.70.3550 | hypothetical protein, conserved | 0 |
| Tb10.70.4220 | hypothetical protein, conserved | 0 |
| Tb10.70.4340 | hypothetical protein, conserved | 0 |
| Tb10.70.4850 | hypothetical protein, conserved | 0 |
| Tb10.70.5070 | hypothetical protein, conserved | 0 |
| Tb10.70.5720 | hypothetical protein, conserved | 0 |
| Tb10.70.6030 | hypothetical protein, conserved | 0 |

| | | |
|---|---|---|
| Tb10.70.6820 | hypothetical protein, conserved | 0 |
| Tb10.70.6930 | hypothetical protein, conserved | 0 |
| Tb10.70.7640 | hypothetical protein, conserved | 0 |
| Tb10.70.7710 | hypothetical protein, conserved | 4 |
| Tb10.70.7770 | hypothetical protein, conserved | 0 |
| Tb11.01.0090 | hypothetical protein, conserved | 0 |
| Tb11.01.0270 | hypothetical protein, conserved | 0 |
| Tb11.01.0460 | hypothetical protein, conserved | 0 |
| Tb11.01.0780 | hypothetical protein, conserved | 0 |
| Tb11.01.0840 | hypothetical protein, conserved | 0 |
| Tb11.01.0970 | hypothetical protein, conserved | 0 |
| Tb11.01.1260 | hypothetical protein, conserved | 0 |
| Tb11.01.1330 | hypothetical protein, conserved | 2 |
| Tb11.01.1420 | hypothetical protein, conserved | 0 |
| Tb11.01.1625 | hypothetical protein, conserved | 0 |
| Tb11.01.1690 | hypothetical protein, conserved | 0 |
| Tb11.01.1730 | hypothetical protein, conserved | 0 |
| Tb11.01.1810 | hypothetical protein, conserved | 0 |
| Tb11.01.1980 | hypothetical protein, conserved | 1 |
| Tb11.01.2070 | hypothetical protein, conserved | 0 |
| Tb11.01.2120 | hypothetical protein, conserved | 0 |
| Tb11.01.2230 | hypothetical protein, conserved | 0 |
| Tb11.01.2390 | hypothetical protein, conserved | 0 |
| Tb11.01.2400 | hypothetical protein, conserved | 0 |
| Tb11.01.2720 | hypothetical protein, conserved | 0 |
| Tb11.01.2770 | hypothetical protein, conserved | 4 |
| Tb11.01.2920 | hypothetical protein, conserved | 0 |
| Tb11.01.3060 | hypothetical protein, conserved | 2 |
| Tb11.01.3100 | hypothetical protein, conserved | 0 |
| Tb11.01.3200 | hypothetical protein, conserved | 0 |
| Tb11.01.3360 | hypothetical protein, conserved | 0 |
| Tb11.01.4200 | hypothetical protein, conserved | 0 |
| Tb11.01.4340 | hypothetical protein, conserved | 2 |
| Tb11.01.4850 | hypothetical protein, conserved | 0 |

| | | |
|---|---|---|
| Tb11.01.5050 | hypothetical protein, conserved | 0 |
| Tb11.01.5150 | hypothetical protein, conserved | 6 |
| Tb11.01.5195 | hypothetical protein, conserved | 0 |
| Tb11.01.5390 | hypothetical protein, conserved | 1 |
| Tb11.01.5820 | hypothetical protein, conserved | 0 |
| Tb11.01.5980 | hypothetical protein, conserved | 0 |
| Tb11.01.6010 | hypothetical protein, conserved | 0 |
| Tb11.01.6100 | hypothetical protein, conserved | 0 |
| Tb11.01.6400 | hypothetical protein, conserved | 0 |
| Tb11.01.6715 | hypothetical protein, conserved | 4 |
| Tb11.01.6850 | hypothetical protein, conserved | 0 |
| Tb11.01.6950 | hypothetical protein, conserved | 0 |
| Tb11.01.7070 | hypothetical protein, conserved | 0 |
| Tb11.01.7160 | hypothetical protein, conserved | 0 |
| Tb11.01.7570 | hypothetical protein, conserved | 0 |
| Tb11.01.7620 | hypothetical protein, conserved | 0 |
| Tb11.01.7980 | hypothetical protein, conserved | 0 |
| Tb11.01.8110 | hypothetical protein, conserved | 0 |
| Tb11.01.8240 | hypothetical protein, conserved | 0 |
| Tb11.01.8710 | hypothetical protein, conserved | 0 |
| Tb11.02.0180 | hypothetical protein, conserved | 6 |
| Tb11.02.0270 | hypothetical protein, conserved | 0 |
| Tb11.02.0280 | hypothetical protein, conserved | 4 |
| Tb11.02.0356 | hypothetical protein, conserved | 0 |
| Tb11.02.0360 | hypothetical protein, conserved | 0 |
| Tb11.02.0560 | hypothetical protein, conserved | 2 |
| Tb11.02.0610 | hypothetical protein, conserved | 0 |
| Tb11.02.0680 | hypothetical protein, conserved | 4 |
| Tb11.02.0840 | hypothetical protein, conserved | 0 |
| Tb11.02.0900 | hypothetical protein, conserved | 1 |
| Tb11.02.0920 | hypothetical protein, conserved | 0 |
| Tb11.02.1110 | hypothetical protein, conserved | 0 |
| Tb11.02.1200 | hypothetical protein, conserved | 0 |
| Tb11.02.1320 | hypothetical protein, conserved | 0 |

| | | |
|---|---|---|
| Tb11.02.1610 | hypothetical protein, conserved | 0 |
| Tb11.02.1620 | hypothetical protein, conserved | 0 |
| Tb11.02.1750 | hypothetical protein, conserved | 0 |
| Tb11.02.1940 | hypothetical protein, conserved | 0 |
| Tb11.02.2080 | hypothetical protein, conserved | 0 |
| Tb11.02.2190 | hypothetical protein, conserved | 0 |
| Tb11.02.2340 | hypothetical protein, conserved | 0 |
| Tb11.02.2490 | hypothetical protein, conserved | 0 |
| Tb11.02.2530 | hypothetical protein, conserved | 0 |
| Tb11.02.2890 | hypothetical protein, conserved | 0 |
| Tb11.02.3000 | hypothetical protein, conserved | 0 |
| Tb11.02.3150 | hypothetical protein, conserved | 0 |
| Tb11.02.3330 | hypothetical protein, conserved | 0 |
| Tb11.02.3540 | hypothetical protein, conserved | 0 |
| Tb11.02.3790 | hypothetical protein, conserved | 0 |
| Tb11.02.3840 | hypothetical protein, conserved | 0 |
| Tb11.02.4240 | hypothetical protein, conserved | 0 |
| Tb11.02.4310 | hypothetical protein, conserved | 10 |
| Tb11.02.4330 | hypothetical protein, conserved | 0 |
| Tb11.02.4400 | hypothetical protein, conserved | 0 |
| Tb11.02.4630 | hypothetical protein, conserved | 0 |
| Tb11.02.4770 | hypothetical protein, conserved | 1 |
| Tb11.02.5290 | hypothetical protein, conserved | 0 |
| Tb11.02.5460 | hypothetical protein, conserved | 0 |
| Tb11.02.5540 | hypothetical protein, conserved | 4 |
| Tb11.02.5590 | hypothetical protein, conserved | 0 |
| Tb11.02.5725 | hypothetical protein, conserved | 0 |
| Tb11.03.0040 | hypothetical protein, conserved | 0 |
| Tb11.03.0150 | hypothetical protein, conserved | 0 |
| Tb11.03.0430 | hypothetical protein, conserved | 0 |
| Tb11.03.0475 | hypothetical protein, conserved | 0 |
| Tb11.03.0570 | hypothetical protein, conserved | 0 |
| Tb11.03.0650 | hypothetical protein, conserved | 2 |
| Tb11.03.0710 | hypothetical protein, conserved | 0 |

| | | |
|---|---|---|
| Tb11.12.0005 | hypothetical protein, conserved | 0 |
| Tb11.12.0014 | hypothetical protein, conserved | 2 |
| Tb11.44.0012 | hypothetical protein, conserved | 0 |
| Tb11.46.0010 | hypothetical protein, conserved | 0 |
| Tb11.47.0008 | hypothetical protein, conserved | 0 |
| Tb11.47.0021 | hypothetical protein, conserved | 0 |
| Tb11.47.0030 | hypothetical protein, conserved | 0 |
| Tb11.50.0003 | hypothetical protein, conserved | 0 |
| Tb11.52.0019 | hypothetical protein, conserved | 1 |
| Tb11.55.0005 | hypothetical protein, conserved | 1 |
| Tb11.57.0003 | hypothetical protein, conserved | 0 |
| Tb927.1.1210 | hypothetical protein, conserved | 0 |
| Tb927.1.1760 | hypothetical protein, conserved | 3 |
| Tb927.1.1850 | hypothetical protein, conserved | 0 |
| Tb927.1.1920 | hypothetical protein, conserved | 0 |
| Tb927.1.2750 | hypothetical protein, conserved | 0 |
| Tb927.1.280 | hypothetical protein, conserved | 0 |
| Tb927.1.4440 | hypothetical protein, conserved | 4 |
| Tb927.1.4450 | hypothetical protein, conserved | 7 |
| Tb927.1.4540 | hypothetical protein, conserved | 0 |
| Tb927.1.4560 | hypothetical protein, conserved | 0 |
| Tb927.1.4580 | hypothetical protein, conserved | 0 |
| Tb927.1.4600 | hypothetical protein, conserved | 0 |
| Tb927.1.4630 | hypothetical protein, conserved | 0 |
| Tb927.1.4650 | hypothetical protein, conserved | 0 |
| Tb927.1.470 | hypothetical protein, conserved | 0 |
| Tb927.1.4700 | hypothetical protein, conserved | 0 |
| Tb927.1.570 | hypothetical protein, conserved | 0 |
| Tb927.1.830 | hypothetical protein, conserved | 0 |
| Tb927.1.990 | hypothetical protein, conserved | 0 |
| Tb927.2.1720 | hypothetical protein, conserved | 0 |
| Tb927.2.1730 | hypothetical protein, conserved | 0 |
| Tb927.2.2160 | hypothetical protein, conserved | 0 |
| Tb927.2.2200 | hypothetical protein, conserved | 0 |

| | | |
|---|---|---|
| Tb927.2.2540 | hypothetical protein, conserved | 0 |
| Tb927.2.3160 | hypothetical protein, conserved | 0 |
| Tb927.2.3610 | hypothetical protein, conserved | 1 |
| Tb927.2.3800 | hypothetical protein, conserved | 0 |
| Tb927.2.4480 | hypothetical protein, conserved | 0 |
| Tb927.2.4620 | hypothetical protein, conserved | 4 |
| Tb927.2.4650 | hypothetical protein, conserved | 0 |
| Tb927.2.5070 | hypothetical protein, conserved | 0 |
| Tb927.2.5260 | hypothetical protein, conserved | 0 |
| Tb927.2.5500 | hypothetical protein, conserved | 0 |
| Tb927.2.5630 | hypothetical protein, conserved | 0 |
| Tb927.3.1020 | hypothetical protein, conserved | 0 |
| Tb927.3.1170 | hypothetical protein, conserved | 0 |
| Tb927.3.1530 | hypothetical protein, conserved | 2 |
| Tb927.3.1560 | hypothetical protein, conserved | 0 |
| Tb927.3.2260 | hypothetical protein, conserved | 0 |
| Tb927.3.2610 | hypothetical protein, conserved | 10 |
| Tb927.3.2670 | hypothetical protein, conserved | 0 |
| Tb927.3.3060 | hypothetical protein, conserved | 0 |
| Tb927.3.3260 | hypothetical protein, conserved | 0 |
| Tb927.3.3710 | hypothetical protein, conserved | 0 |
| Tb927.3.3740 | hypothetical protein, conserved | 0 |
| Tb927.3.3890 | hypothetical protein, conserved | 0 |
| Tb927.3.3980 | hypothetical protein, conserved | 0 |
| Tb927.3.4170 | hypothetical protein, conserved | 0 |
| Tb927.3.4410 | hypothetical protein, conserved | 0 |
| Tb927.3.4450 | hypothetical protein, conserved | 0 |
| Tb927.3.4530 | hypothetical protein, conserved | 0 |
| Tb927.3.5260 | hypothetical protein, conserved | 0 |
| Tb927.3.660 | hypothetical protein, conserved | 0 |
| Tb927.3.750 | hypothetical protein, conserved | 1 |
| Tb927.3.820 | hypothetical protein, conserved | 0 |
| Tb927.4.1000 | hypothetical protein, conserved | 0 |
| Tb927.4.1260 | hypothetical protein, conserved | 0 |

| | | |
|---|---|---|
| Tb927.4.1420 | hypothetical protein, conserved | 3 |
| Tb927.4.1440 | hypothetical protein, conserved | 0 |
| Tb927.4.1590 | hypothetical protein, conserved | 0 |
| Tb927.4.1670 | hypothetical protein, conserved | 0 |
| Tb927.4.1730 | hypothetical protein, conserved | 0 |
| Tb927.4.1770 | hypothetical protein, conserved | 1 |
| Tb927.4.1830 | hypothetical protein, conserved | 0 |
| Tb927.4.2210 | hypothetical protein, conserved | 0 |
| Tb927.4.2330 | hypothetical protein, conserved | 0 |
| Tb927.4.2360 | hypothetical protein, conserved | 0 |
| Tb927.4.2440 | hypothetical protein, conserved | 0 |
| Tb927.4.2840 | hypothetical protein, conserved | 0 |
| Tb927.4.2870 | hypothetical protein, conserved | 0 |
| Tb927.4.3000 | hypothetical protein, conserved | 0 |
| Tb927.4.3070 | hypothetical protein, conserved | 0 |
| Tb927.4.3690 | hypothetical protein, conserved | 0 |
| Tb927.4.3730 | hypothetical protein, conserved | 0 |
| Tb927.4.3780 | hypothetical protein, conserved | 1 |
| Tb927.4.3830 | hypothetical protein, conserved | 0 |
| Tb927.4.3970 | hypothetical protein, conserved | 0 |
| Tb927.4.4040 | hypothetical protein, conserved | 0 |
| Tb927.4.4120 | hypothetical protein, conserved | 0 |
| Tb927.4.4200 | hypothetical protein, conserved | 0 |
| Tb927.4.4240 | hypothetical protein, conserved | 0 |
| Tb927.4.4260 | hypothetical protein, conserved | 0 |
| Tb927.4.4280 | hypothetical protein, conserved | 0 |
| Tb927.4.4600 | hypothetical protein, conserved | 0 |
| Tb927.4.4690 | hypothetical protein, conserved | 0 |
| Tb927.4.4700 | hypothetical protein, conserved | 0 |
| Tb927.4.4800 | hypothetical protein, conserved | 2 |
| Tb927.4.5000 | hypothetical protein, conserved | 0 |
| Tb927.4.5130 | hypothetical protein, conserved | 0 |
| Tb927.4.5140 | hypothetical protein, conserved | 0 |
| Tb927.4.920 | hypothetical protein, conserved | 0 |

| | | |
|---|---|---|
| Tb927.5.140 | hypothetical protein, conserved | 0 |
| Tb927.5.1500 | hypothetical protein, conserved | 0 |
| Tb927.5.1620 | hypothetical protein, conserved | 1 |
| Tb927.5.170 | hypothetical protein, conserved | 0 |
| Tb927.5.1860 | hypothetical protein, conserved | 0 |
| Tb927.5.2110 | hypothetical protein, conserved | 0 |
| Tb927.5.2340 | hypothetical protein, conserved | 0 |
| Tb927.5.2470 | hypothetical protein, conserved | 0 |
| Tb927.5.2480 | hypothetical protein, conserved | 0 |
| Tb927.5.2620 | hypothetical protein, conserved | 0 |
| Tb927.5.2730 | hypothetical protein, conserved | 0 |
| Tb927.5.2810 | hypothetical protein, conserved | 0 |
| Tb927.5.2830 | hypothetical protein, conserved | 0 |
| Tb927.5.2910 | hypothetical protein, conserved | 0 |
| Tb927.5.2930 | hypothetical protein, conserved | 0 |
| Tb927.5.3040 | hypothetical protein, conserved | 1 |
| Tb927.5.3090 | hypothetical protein, conserved | 0 |
| Tb927.5.3110 | hypothetical protein, conserved | 0 |
| Tb927.5.3140 | hypothetical protein, conserved | 0 |
| Tb927.5.3180 | hypothetical protein, conserved | 0 |
| Tb927.5.3310 | hypothetical protein, conserved | 0 |
| Tb927.5.3410 | hypothetical protein, conserved | 0 |
| Tb927.5.3440 | hypothetical protein, conserved | 0 |
| Tb927.5.3470 | hypothetical protein, conserved | 0 |
| Tb927.5.3640 | hypothetical protein, conserved | 0 |
| Tb927.5.3660 | hypothetical protein, conserved | 0 |
| Tb927.5.3720 | hypothetical protein, conserved | 0 |
| Tb927.5.3770 | hypothetical protein, conserved | 0 |
| Tb927.5.3780 | hypothetical protein, conserved | 0 |
| Tb927.5.3890 | hypothetical protein, conserved | 0 |
| Tb927.5.4120 | hypothetical protein, conserved | 0 |
| Tb927.5.810 | hypothetical protein, conserved | 0 |
| Tb927.5.880 | hypothetical protein, conserved | 0 |
| Tb927.5.990 | hypothetical protein, conserved | 0 |

| | | |
|---|---|---|
| Tb927.6.1130 | hypothetical protein, conserved | 0 |
| Tb927.6.1410 | hypothetical protein, conserved | 0 |
| Tb927.6.1840 | hypothetical protein, conserved | 0 |
| Tb927.6.1990 | hypothetical protein, conserved | 0 |
| Tb927.6.2070 | hypothetical protein, conserved | 0 |
| Tb927.6.2120 | hypothetical protein, conserved | 0 |
| Tb927.6.2180 | hypothetical protein, conserved | 0 |
| Tb927.6.2190 | hypothetical protein, conserved | 0 |
| Tb927.6.2380 | hypothetical protein, conserved | 0 |
| Tb927.6.240 | hypothetical protein, conserved | 0 |
| Tb927.6.2410 | hypothetical protein, conserved | 0 |
| Tb927.6.2750 | hypothetical protein, conserved | 0 |
| Tb927.6.2940 | hypothetical protein, conserved | 0 |
| Tb927.6.3180 | hypothetical protein, conserved | 0 |
| Tb927.6.3270 | hypothetical protein, conserved | 0 |
| Tb927.6.3340 | hypothetical protein, conserved | 0 |
| Tb927.6.3910 | hypothetical protein, conserved | 0 |
| Tb927.6.4010 | hypothetical protein, conserved | 0 |
| Tb927.6.4050 | hypothetical protein, conserved | 0 |
| Tb927.6.4060 | hypothetical protein, conserved | 2 |
| Tb927.6.4150 | hypothetical protein, conserved | 0 |
| Tb927.6.4160 | hypothetical protein, conserved | 1 |
| Tb927.6.4440 | hypothetical protein, conserved | 0 |
| Tb927.6.4490 | hypothetical protein, conserved | 0 |
| Tb927.6.4570 | hypothetical protein, conserved | 1 |
| Tb927.6.4790 | hypothetical protein, conserved | 0 |
| Tb927.6.590 | hypothetical protein, conserved | 0 |
| Tb927.6.680 | hypothetical protein, conserved | 0 |
| Tb927.6.690 | hypothetical protein, conserved | 0 |
| Tb927.7.1520 | hypothetical protein, conserved | 0 |
| Tb927.7.2040 | hypothetical protein, conserved | 0 |
| Tb927.7.2380 | hypothetical protein, conserved | 0 |
| Tb927.7.2570 | hypothetical protein, conserved | 0 |
| Tb927.7.2740 | hypothetical protein, conserved | 0 |

| | | |
|---|---|---|
| Tb927.7.2760 | hypothetical protein, conserved | 0 |
| Tb927.7.2780 | hypothetical protein, conserved | 0 |
| Tb927.7.3140 | hypothetical protein, conserved | 0 |
| Tb927.7.3510 | hypothetical protein, conserved | 0 |
| Tb927.7.3540 | hypothetical protein, conserved | 0 |
| Tb927.7.3590 | hypothetical protein, conserved | 2 |
| Tb927.7.3640 | hypothetical protein, conserved | 0 |
| Tb927.7.370 | hypothetical protein, conserved | 0 |
| Tb927.7.4010 | hypothetical protein, conserved | 0 |
| Tb927.7.4030 | hypothetical protein, conserved | 0 |
| Tb927.7.4100 | hypothetical protein, conserved | 0 |
| Tb927.7.4200 | hypothetical protein, conserved | 0 |
| Tb927.7.4240 | hypothetical protein, conserved | 0 |
| Tb927.7.4250 | hypothetical protein, conserved | 0 |
| Tb927.7.4430 | hypothetical protein, conserved | 0 |
| Tb927.7.4470 | hypothetical protein, conserved | 4 |
| Tb927.7.4510 | hypothetical protein, conserved | 0 |
| Tb927.7.4540 | hypothetical protein, conserved | 0 |
| Tb927.7.4620 | hypothetical protein, conserved | 0 |
| Tb927.7.4710 | hypothetical protein, conserved | 0 |
| Tb927.7.4760 | hypothetical protein, conserved | 1 |
| Tb927.7.480 | hypothetical protein, conserved | 3 |
| Tb927.7.4980 | hypothetical protein, conserved | 1 |
| Tb927.7.5550 | hypothetical protein, conserved | 0 |
| Tb927.7.5610 | hypothetical protein, conserved | 0 |
| Tb927.7.5840 | hypothetical protein, conserved | 4 |
| Tb927.7.5850 | hypothetical protein, conserved | 0 |
| Tb927.7.6790 | hypothetical protein, conserved | 0 |
| Tb927.7.770 | hypothetical protein, conserved | 0 |
| Tb927.7.840 | hypothetical protein, conserved | 1 |
| Tb927.8.1260 | hypothetical protein, conserved | 0 |
| Tb927.8.1490 | hypothetical protein, conserved | 0 |
| Tb927.8.1920 | hypothetical protein, conserved | 0 |
| Tb927.8.2180 | hypothetical protein, conserved | 1 |

| | | |
|---|---|---|
| Tb927.8.2290 | hypothetical protein, conserved | 0 |
| Tb927.8.2440 | hypothetical protein, conserved | 0 |
| Tb927.8.2830 | hypothetical protein, conserved | 0 |
| Tb927.8.2890 | hypothetical protein, conserved | 0 |
| Tb927.8.3110 | hypothetical protein, conserved | 0 |
| Tb927.8.3440 | hypothetical protein, conserved | 0 |
| Tb927.8.3570 | hypothetical protein, conserved | 0 |
| Tb927.8.3640 | hypothetical protein, conserved | 0 |
| Tb927.8.3780 | hypothetical protein, conserved | 0 |
| Tb927.8.3990 | hypothetical protein, conserved | 0 |
| Tb927.8.4020 | hypothetical protein, conserved | 0 |
| Tb927.8.4070 | hypothetical protein, conserved | 0 |
| Tb927.8.4120 | hypothetical protein, conserved | 0 |
| Tb927.8.4190 | hypothetical protein, conserved | 0 |
| Tb927.8.4670 | hypothetical protein, conserved | 0 |
| Tb927.8.4690 | hypothetical protein, conserved | 0 |
| Tb927.8.4930 | hypothetical protein, conserved | 0 |
| Tb927.8.5070 | hypothetical protein, conserved | 0 |
| Tb927.8.5270 | hypothetical protein, conserved | 0 |
| Tb927.8.5520 | hypothetical protein, conserved | 0 |
| Tb927.8.5670 | hypothetical protein, conserved | 0 |
| Tb927.8.5700 | hypothetical protein, conserved | 0 |
| Tb927.8.5720 | hypothetical protein, conserved | 0 |
| Tb927.8.5960 | hypothetical protein, conserved | 0 |
| Tb927.8.6190 | hypothetical protein, conserved | 0 |
| Tb927.8.6400 | hypothetical protein, conserved | 0 |
| Tb927.8.6460 | hypothetical protein, conserved | 0 |
| Tb927.8.6530 | hypothetical protein, conserved | 0 |
| Tb927.8.6600 | hypothetical protein, conserved | 0 |
| Tb927.8.670 | hypothetical protein, conserved | 0 |
| Tb927.8.6890 | hypothetical protein, conserved | 1 |
| Tb927.8.7180 | hypothetical protein, conserved | 0 |
| Tb927.8.7770 | hypothetical protein, conserved | 0 |
| Tb927.8.7820 | hypothetical protein, conserved | 0 |

| | | |
|---|---|---|
| Tb927.8.8210 | hypothetical protein, conserved | 0 |
| Tb11.01.3960 | hypothetical protein, conserved,calmodulin-like protein containing EF hand domain | 0 |
| Tb11.02.0910 | hypothetical protein, conserved,leucine-rich repeat protein (LRRP), putative | 0 |
| Tb10.70.1530 | hypothetical protein, conserved,predicted HORMA domain protein | 0 |
| Tb10.70.2780 | hypothetical protein, conserved,predicted SAP domain protein | 0 |
| Tb11.02.4540 | hypothetical protein, conserved,predicted tetratricopeptide repeat (TPR) protein | 0 |
| Tb09.211.1655 | hypothetical protein, conserved,predicted zinc finger protein | 0 |
| Tb10.389.0570 | hypothetical protein, conserved,predicted zinc finger protein | 0 |
| Tb10.70.0450 | hypothetical protein, conserved,ring finger containing protein | 0 |
| Tb10.6k15.2170 | hypothetical protein, conserved,S. cerevisiae PSP1 homologue, putative | 0 |
| Tb09.160.0050 | hypothetical protein, unlikely | 0 |
| Tb09.160.1150 | hypothetical protein, unlikely | 0 |
| Tb09.160.1170 | hypothetical protein, unlikely | 0 |
| Tb09.160.1190 | hypothetical protein, unlikely | 0 |
| Tb09.160.1480 | hypothetical protein, unlikely | 0 |
| Tb09.160.1930 | hypothetical protein, unlikely | 0 |
| Tb09.160.2330 | hypothetical protein, unlikely | 0 |
| Tb09.160.2500 | hypothetical protein, unlikely | 0 |
| Tb09.160.2560 | hypothetical protein, unlikely | 0 |
| Tb09.160.2950 | hypothetical protein, unlikely | 1 |
| Tb09.160.3010 | hypothetical protein, unlikely | 1 |
| Tb09.160.3280 | hypothetical protein, unlikely | 0 |
| Tb09.160.3290 | hypothetical protein, unlikely | 0 |
| Tb09.160.3650 | hypothetical protein, unlikely | 0 |
| Tb09.160.3840 | hypothetical protein, unlikely | 0 |
| Tb09.160.3950 | hypothetical protein, unlikely | 0 |
| Tb09.160.4140 | hypothetical protein, unlikely | 0 |
| Tb09.160.4160 | hypothetical protein, unlikely | 0 |
| Tb09.160.4320 | hypothetical protein, unlikely | 0 |
| Tb09.160.4360 | hypothetical protein, unlikely | 0 |
| Tb09.160.5420 | hypothetical protein, unlikely | 0 |
| Tb09.211.0060 | hypothetical protein, unlikely | 0 |
| Tb09.211.0780 | hypothetical protein, unlikely | 0 |
| Tb09.211.2770 | hypothetical protein, unlikely | 1 |

| | | |
|---|---|---|
| Tb09.211.2890 | hypothetical protein, unlikely | 1 |
| Tb09.211.3190 | hypothetical protein, unlikely | 0 |
| Tb09.211.4040 | hypothetical protein, unlikely | 0 |
| Tb09.211.4220 | hypothetical protein, unlikely | 2 |
| Tb09.211.5000 | hypothetical protein, unlikely | 0 |
| Tb09.244.0260 | hypothetical protein, unlikely | 0 |
| Tb09.244.0300 | hypothetical protein, unlikely | 0 |
| Tb09.244.0380 | hypothetical protein, unlikely | 0 |
| Tb09.244.2880 | hypothetical protein, unlikely | 0 |
| Tb09.244.2990 | hypothetical protein, unlikely | 1 |
| Tb09.v1.0250 | hypothetical protein, unlikely | 0 |
| Tb09.v1.0280 | hypothetical protein, unlikely | 2 |
| Tb09.v1.0560 | hypothetical protein, unlikely | 0 |
| Tb09.v1.0630 | hypothetical protein, unlikely | 1 |
| Tb09.v1.0980 | hypothetical protein, unlikely | 0 |
| Tb10.26.0080 | hypothetical protein, unlikely | 0 |
| Tb10.26.0420 | hypothetical protein, unlikely | 0 |
| Tb10.70.4270 | hypothetical protein, unlikely | 0 |
| Tb10.70.4920 | hypothetical protein, unlikely | 0 |
| Tb927.1.1070 | hypothetical protein, unlikely | 0 |
| Tb927.1.2010 | hypothetical protein, unlikely | 0 |
| Tb927.1.2130 | hypothetical protein, unlikely | 0 |
| Tb927.1.2180 | hypothetical protein, unlikely | 1 |
| Tb927.1.2270 | hypothetical protein, unlikely | 0 |
| Tb927.1.2930 | hypothetical protein, unlikely | 0 |
| Tb927.1.2950 | hypothetical protein, unlikely | 0 |
| Tb927.1.3140 | hypothetical protein, unlikely | 1 |
| Tb927.1.3330 | hypothetical protein, unlikely | 0 |
| Tb927.1.3520 | hypothetical protein, unlikely | 0 |
| Tb927.1.3710 | hypothetical protein, unlikely | 0 |
| Tb927.1.3960 | hypothetical protein, unlikely | 0 |
| Tb927.1.4320 | hypothetical protein, unlikely | 0 |
| Tb927.1.4850 | hypothetical protein, unlikely | 0 |
| Tb927.1.5020 | hypothetical protein, unlikely | 0 |

| | | |
|---|---|---|
| Tb927.1.5070 | hypothetical protein, unlikely | 0 |
| Tb927.1.5140 | hypothetical protein, unlikely | 1 |
| Tb927.1.5270 | hypothetical protein, unlikely | 0 |
| Tb927.1.80 | hypothetical protein, unlikely | 0 |
| Tb927.1.910 | hypothetical protein, unlikely | 0 |
| Tb927.1.940 | hypothetical protein, unlikely | 0 |
| Tb927.1.970 | hypothetical protein, unlikely | 12 |
