# Supplementary material for: Exocytosis and protein secretion in Trypanosoma
Source: BMC Microbiol. 2010 Jan 26;10:20. doi: 10.1186/1471-2180-10-20 (PMC3224696; doi:10.1186/1471-2180-10-20)
Supplement: Additional file 7 — Table S7. Proteins identified in sucrose fractionated membranes from infected rat serum (IRS). contains the list of the IRS proteins. IRS proteins shared with ESPs or exosome are boxed in yellow and orange, respectively. [file 1471-2180-10-20-S7.PDF]

Table S7

proteins identified in sucrose fractionated membranes from infected rat serum (IRS)

proteins from IRS shared with ESPs

proteins identified in exosomes in the litterature

Accession number	Protein name	Matching peptides	Highest peptide score
Tb11.01.3110	heat shock protein 70	4	64
Tb10.26.1080	heat shock protein 83; heat shock protein	8	57
Tb927.2.5160/30J2.30	chaperone protein DnaJ, putative	1	74
Tb10.70.0280	HSP60 chaperonin Hsp60, mitochondrial precursor	1	32
Tb10.70.1190	VCP valosin-containing protein homolog; Transitional endoplasmic reticulum ATPase,	2	37
Tb11.02.0730	mca1 metacaspase; cysteine peptidase, Clan CD, family C13	1	35
Tb11.02.1070	aminopeptidase, putative; metallo-peptidase, Clan MA(E) Family M1	3	37
Tb09.160.4250	TRYP1; TXNPx tryparedoxin peroxidase	4	64
Tb10.61.0180	peptidylprolyl isomerase-like protein, putative	1	32
Tb10.6k15.2290	BS2 protein disulfide isomerase; bloodstream- specific protein 2 precursor	2	86
Tb10.70.3290	DHH1 ATP-dependent DEAD-box RNA helicase, putative; DHH1	2	48
Tb11.01.7800	NDPK nucleoside diphosphate kinase	2	75
Tb927.8.4430/Tb08.29H22.830	nucleoside phosphorylase, putative	4	64
Tb09.160.3630	PDE cAMP-specific phosphodiesterase	2	50
Tb927.3.4680/Tb03.48K5.180	RAB GDP dissociation inhibitor alpha, putative	1	26
Tb927.3.1120/Tb03.27F10.90	rtb2 GTP-binding nuclear protein rtb2, putative	3	60
Tb09.211.4240	phosphoinositide-binding protein, putative	1	33
Tb927.2.4590/30M24.285	branched-chain amino acid aminotransferase, putative	2	40
Tb11.01.1350	S-adenosylhomocysteine hydrolase, putative	2	43
Tb11.02.3210	TIM triosephosphate isomerase	3	79
Tb927.6.4280/Tb06.26G9.1050	GAPDH glyceraldehyde 3-phosphate dehydrogenase, glycosomal	11	71
Tb09.211.3540	glk1; gk glycerol kinase, glycosomal	4	67
Tb927.8.3530/Tb08.28L1.740	glycerol-3-phosphate dehydrogenase [NAD+], glycosomal	3	49

Tb10.70.1370	ALD fructose-bisphosphate aldolase, glycosomal, putative	20	93
Tb927.1.700	PGKC; gPGK phosphoglycerate kinase	7	84
Tb10.61.2680	PYK1 pyruvate kinase 1	7	65
Tb10.70.5650	TEF1 elongation factor 1-alpha	5	80
Tb10.70.1100	translation elongation factor 1-beta, putative	2	67
Tb10.70.2650	elongation factor 2	10	83
Tb09.160.3270	eukaryotic initiation factor 4a, putative	4	98
Tb11.46.0001	60S acidic ribosomal subunit protein, putative	4	82
Tb10.26.0560	60S ribosomal protein L6, putative	2	95
Tb09.211.0110	QM 60S ribosomal protein L10, putative; QM-like protein	2	63
Tb10.70.4740	enolase	12	92
Tb927.4.5010/Tb04.3M17.390	calreticulin, putative	1	42
Tb927.1.2340	alpha tubulin	4	62
Tb927.1.2330	beta tubulin	14	79
Tb927.7.7420/Tb07.30D13.360	ATP synthase alpha chain, mitochondrial precursor; ATP synthase F1, alpha subunit	1	53
Tb10.406.0330	histone H2B, putative	2	42
Tb927.8.4970/Tb08.5H5.920	PFR 69 kDa paraflagellar rod protein; PFR2	2	63
Tb927.3.4290/Tb03.26J7.510	PFR-C; PFR-D 73 kDa paraflagellar rod protein; PFR1	8	96
Tb10.61.1750	TBKIFC1 C-terminal motor kinesin, putative	2	45
Tb09.211.2700	hypothetical protein, conserved	1	72
Tb10.70.1130	hypothetical protein, conserved	1	74
Tb927.3.5050/Tb03.48K5.830	hypothetical protein, conserved	1	41
Tb09.160.1160	hypothetical protein, conserved	7	65
Tb927.6.1030	cysteine peptidase precursor,cysteine peptidase, Clan CA, family C1, Cathepsin L-like	1	37
Tb10.389.0550	ras-related protein rab-5,small GTPase, putative	1	41
Tb927.2.5060	GTP binding protein, putative	2	40
Tb10.70.5820	hexokinase	9	86
Tb927.3.3270	ATP-dependent phosphofructokinase,6-phospho-1-fructokinase	4	64
Tb10.70.5680	elongation factor 1-alpha,EF-1-alpha	3	70
Tb927.7.1040	40S ribosomal protein S16, putative	3	60
Tb09.244.2590	60S ribosomal protein L32	2	59
Tb10.70.1730	40S ribosomal protein S18, putative	4	70
Tb927.3.1380	ATP synthase beta chain, mitochondrial precursor,ATP synthase F1, beta subunit	2	53
Tb927.7.2820	histone H2A, putative	2	54
Tb927.8.5460	flagellar calcium-binding protein,44 kDa calflagin,44 kDa calcimedin	1	30

Tb927.7.3550	hypothetical protein, conserved	1	44
Tb927.3.1010	hypothetical protein, conserved	1	70
Tb927.3.1580	hypothetical protein, conserved	1	36
Tb927.5.4090	hypothetical protein, conserved	1	32
Tb927.5.960	hypothetical protein, conserved	1	53
