# Supplementary material for: Exocytosis and protein secretion in Trypanosoma
Source: BMC Microbiol. 2010 Jan 26;10:20. doi: 10.1186/1471-2180-10-20 (PMC3224696; doi:10.1186/1471-2180-10-20)
Supplement: Additional file 8 — Table S8. Additional informations on proteins identified in secretome. contains the list of the proteins identified in 1D and BN-PAGE gels spots. Protein score, number of peptides identified and number of peptides that fit to our stringent filter are provided. [file 1471-2180-10-20-S8.PDF]

# Table S8

| CODE SAMPLES | HIT | ACCESSION | PROTEIN SCORE | QUERIES MATCHED | QUERIES SELECTED | SEQUENCE COVERAGE |
|---|---|---|---|---|---|---|
| TRP_11 | 1 | Tb10.26.1080 | 487 | 15 peptides | 7 peptides | 16% |
| TRP_11 | 2 | Tb927.8.3530/Tb08.28L1.740 | 283 | 6 peptides | 6 peptides | 13% |
| TRP_11 | 3 | Tb927.6.4280/Tb06.26G9.1050 | 195 | 7 peptides | 3 peptides | 14% |
| TRP_11 | 4 | Tb10.6k15.3850 | 194 | 4 peptides | 2 peptides | 15% |
| TRP_11 | 5 | Tb11.01.3170 | 152 | 3 peptides | 2 peptides | 8% |
| TRP_11 | 6 | Tb10.70.2650 | 129 | 4 peptides | 2 peptides | 5% |
| TRP_11 | 7 | Tb09.160.4560 | 86 | 2 peptides | 1 peptides | 4% |
| TRP_11 | 8 | Tb10.70.1370 | 82 | 2 peptides | 1 peptides | 5% |
| TRP_11 | 9 | Tb09.211.3610 | 82 | 2 peptides | 1 peptides | 1% |
| TRP_11 | 26 | Tb11.01.2270 | 26 | 2 peptides | 0 peptides | 5% |
| TRP_11 | 28 | Tb927.8.7080/Tb08.10K10.740 | 25 | 3 peptides | 1 peptides | 1% |
| TRP_12 | 1 | Tb10.26.1080 | 527 | 13 peptides | 9 peptides | 17% |
| TRP_12 | 2 | Tb927.8.3530/Tb08.28L1.740 | 227 | 6 peptides | 4 peptides | 13% |
| TRP_12 | 3 | Tb10.70.2650 | 170 | 6 peptides | 3 peptides | 8% |
| TRP_12 | 4 | Tb11.01.3170 | 140 | 4 peptides | 1 peptides | 11% |
| TRP_12 | 5 | Tb927.6.4280/Tb06.26G9.1050 | 127 | 4 peptides | 2 peptides | 11% |
| TRP_12 | 6 | Tb10.6k15.3850 | 110 | 3 peptides | 2 peptides | 9% |
| TRP_12 | 7 | Tb11.01.3040 | 86 | 2 peptides | 1 peptides | 6% |
| TRP_12 | 8 | Tb09.211.3610 | 72 | 3 peptides | 2 peptides | 1% |
| TRP_12 | 9 | Tb09.160.3710 | 60 | 1 peptides | 1 peptides | 4% |
| TRP_12 | 10 | Tb11.02.3210 | 53 | 1 peptides | 1 peptides | 6% |
| TRP_12 | 11 | Tb927.7.1300/Tb07.27M11.560 | 40 | 1 peptides | 1 peptides | 2% |
| TRP_13 | 1 | Tb10.26.1080 | 579 | 15 peptides | 10 peptides | 16% |
| TRP_13 | 2 | Tb10.70.2650 | 240 | 6 peptides | 4 peptides | 7% |
| TRP_13 | 3 | Tb927.8.3530/Tb08.28L1.740 | 221 | 5 peptides | 4 peptides | 11% |
| TRP_13 | 4 | Tb10.6k15.3850 | 205 | 3 peptides | 3 peptides | 12% |
| TRP_13 | 5 | Tb11.01.3170 | 157 | 3 peptides | 1 peptides | 8% |
| TRP_13 | 6 | Tb09.211.3610 | 110 | 6 peptides | 1 peptides | 4% |
| TRP_13 | 7 | Tb09.160.3710 | 101 | 1 peptides | 1 peptides | 4% |
| TRP_13 | 8 | Tb11.01.3040 | 99 | 2 peptides | 2 peptides | 6% |
| TRP_13 | 10 | Tb927.6.4280/Tb06.26G9.1050 | 64 | 2 peptides | 1 peptides | 6% |
| TRP_13 | 11 | Tb927.7.1300/Tb07.27M11.560 | 54 | 1 peptides | 1 peptides | 2% |

| | | | | | | |
|---|---|---|---|---|---|---|
| TRP_13 | 13 | Tb927.7.3550/Tb07.28B13.500 | 45 | 3 peptides | 1 peptides | 2% |
| TRP_13 | 15 | Tb10.61.0540 | 34 | 1 peptides | 1 peptides | 3% |
| TRP_13 | 18 | Tb11.02.4700 | 29 | 1 peptides | 1 peptides | 3% |
| TRP_14 | 1 | Tb10.70.2650 | 257 | 8 peptides | 4 peptides | 10% |
| TRP_14 | 2 | Tb10.26.1080 | 164 | 3 peptides | 3 peptides | 5% |
| TRP_14 | 3 | Tb11.02.5450 | 126 | 2 peptides | 2 peptides | 2% |
| TRP_14 | 4 | Tb11.02.4700 | 104 | 3 peptides | 2 peptides | 7% |
| TRP_14 | 5 | Tb10.61.0540 | 98 | 3 peptides | 3 peptides | 13% |
| TRP_14 | 6 | Tb11.02.4870 | 94 | 3 peptides | 1 peptides | 12% |
| TRP_14 | 7 | Tb09.160.3820 | 76 | 2 peptides | 1 peptides | 4% |
| TRP_14 | 8 | Tb11.01.3110 | 55 | 1 peptides | 1 peptides | 1% |
| TRP_14 | 10 | Tb927.6.1570/Tb06.28P18.310 | 45 | 1 peptides | 1 peptides | 3% |
| TRP_14 | 11 | Tb11.01.5860 | 45 | 1 peptides | 1 peptides | 2% |
| TRP_14 | 12 | Tb927.7.1300/Tb07.27M11.560 | 43 | 1 peptides | 1 peptides | 2% |
| TRP_14 | 13 | Tb09.160.4250 | 43 | 1 peptides | 1 peptides | 5% |
| TRP_14 | 14 | Tb11.01.1290 | 43 | 2 peptides | 1 peptides | 6% |
| TRP_14 | 40 | Tb11.01.2270 | 22 | 1 peptides | 1 peptides | 2% |
| TRP_15 | 1 | Tb11.02.4700 | 178 | 5 peptides | 5 peptides | 20% |
| TRP_15 | 2 | Tb11.02.3210 | 172 | 2 peptides | 2 peptides | 11% |
| TRP_15 | 3 | Tb11.02.5450 | 168 | 3 peptides | 3 peptides | 4% |
| TRP_15 | 4 | Tb10.61.0540 | 130 | 5 peptides | 3 peptides | 6% |
| TRP_15 | 5 | Tb09.160.3820 | 92 | 2 peptides | 2 peptides | 3% |
| TRP_15 | 6 | Tb11.01.3110 | 60 | 1 peptides | 1 peptides | 1% |
| TRP_15 | 7 | Tb10.100.0120 | 52 | 1 peptides | 1 peptides | 4% |
| TRP_15 | 8 | Tb927.7.4520/Tb07.26A24.810 | 50 | 2 peptides | 2 peptides | 13% |
| TRP_15 | 9 | Tb927.6.1570/Tb06.28P18.310 | 46 | 1 peptides | 1 peptides | 3% |
| TRP_15 | 10 | Tb10.70.4740 | 46 | 1 peptides | 1 peptides | 4% |
| TRP_16 | 1 | Tb11.02.3210 | 191 | 4 peptides | 3 peptides | 20% |
| TRP_16 | 2 | Tb11.02.5450 | 157 | 5 peptides | 2 peptides | 8% |
| TRP_16 | 3 | Tb11.02.4700 | 150 | 3 peptides | 2 peptides | 14% |
| TRP_16 | 4 | Tb10.100.0170 | 111 | 2 peptides | 1 peptides | 10% |
| TRP_16 | 5 | Tb927.7.4520/Tb07.26A24.810 | 79 | 2 peptides | 2 peptides | 13% |
| TRP_16 | 6 | Tb927.8.1990/Tb08.26N11.460 | 77 | 2 peptides | 1 peptides | 8% |
| TRP_16 | 7 | Tb927.7.3550/Tb07.28B13.500 | 71 | 1 peptides | 1 peptides | 0% |
| TRP_16 | 8 | Tb10.70.2650 | 68 | 2 peptides | 1 peptides | 3% |
| TRP_16 | 9 | Tb10.26.1080 | 62 | 1 peptides | 1 peptides | 1% |

| | | | | | | |
|---|---|---|---|---|---|---|
| TRP_16 | 10 | Tb10.70.7730 | 57 | 1 peptides | 1 peptides | 2% |
| TRP_16 | 12 | Tb09.211.2590 | 54 | 1 peptides | 1 peptides | 3% |
| TRP_16 | 13 | Tb11.02.3390 | 49 | 1 peptides | 1 peptides | 5% |
| TRP_16 | 14 | Tb11.01.3110 | 43 | 1 peptides | 1 peptides | 1% |
| TRP_16 | 15 | Tb09.160.4250 | 43 | 1 peptides | 1 peptides | 5% |
| TRP_16 | 16 | Tb10.100.0120 | 41 | 1 peptides | 1 peptides | 4% |
| TRP_16 | 18 | Tb09.160.3820 | 36 | 1 peptides | 1 peptides | 1% |
| TRP_16 | 19 | Tb927.2.4680/30M24.240 | 32 | 1 peptides | 1 peptides | 2% |
| TRP_17 | 1 | Tb11.01.7800 | 161 | 2 peptides | 2 peptides | 16% |
| TRP_17 | 2 | Tb11.03.0410 | 97 | 3 peptides | 2 peptides | 19% |
| TRP_17 | 3 | Tb11.02.5450 | 87 | 2 peptides | 2 peptides | 2% |
| TRP_17 | 4 | Tb927.8.1990/Tb08.26N11.460 | 87 | 2 peptides | 2 peptides | 8% |
| TRP_17 | 5 | Tb09.160.3270 | 72 | 1 peptides | 1 peptides | 2% |
| TRP_17 | 7 | Tb10.70.7730 | 62 | 1 peptides | 1 peptides | 2% |
| TRP_17 | 8 | Tb927.7.7460/Tb07.30D13.320 | 60 | 1 peptides | 1 peptides | 5% |
| TRP_17 | 10 | Tb11.01.1190 | 36 | 1 peptides | 1 peptides | 4% |
| TRP_18 | 1 | Tb11.01.7800 | 153 | 2 peptides | 2 peptides | 16% |
| TRP_18 | 2 | Tb11.03.0410 | 145 | 2 peptides | 2 peptides | 17% |
| TRP_18 | 3 | Tb09.160.3270 | 127 | 4 peptides | 2 peptides | 6% |
| TRP_18 | 4 | Tb11.02.5450 | 113 | 2 peptides | 2 peptides | 2% |
| TRP_18 | 5 | Tb927.6.4280/Tb06.26G9.1050 | 87 | 3 peptides | 2 peptides | 9% |
| TRP_18 | 6 | Tb11.03.0250 | 85 | 2 peptides | 2 peptides | 11% |
| TRP_18 | 7 | Tb927.7.7460/Tb07.30D13.320 | 76 | 1 peptides | 1 peptides | 5% |
| TRP_18 | 9 | Tb10.70.7730 | 58 | 1 peptides | 1 peptides | 2% |
| TRP_18 | 10 | Tb11.01.1190 | 40 | 1 peptides | 1 peptides | 4% |
| TRP_18 | 11 | Tb927.8.1990/Tb08.26N11.460 | 39 | 2 peptides | 1 peptides | 9% |
| TRP_18 | 14 | Tb09.211.4210 | 32 | 2 peptides | 1 peptides | 1% |
| TRP_19 | 1 | Tb11.01.7800 | 197 | 5 peptides | 3 peptides | 30% |
| TRP_19 | 2 | Tb927.4.2080/Tb04.29M18.770 | 59 | 3 peptides | 1 peptides | 3% |
| TRP_19 | 7 | Tb927.3.5180/Tb03.5L5.210 | 40 | 1 peptides | 1 peptides | 10% |
| TRP_20 | 1 | Tb927.3.5180/Tb03.5L5.210 | 65 | 1 peptides | 1 peptides | 10% |
| TRP_20 | 2 | Tb10.70.7730 | 56 | 1 peptides | 1 peptides | 2% |
| TRP_20 | 4 | Tb10.26.0680 | 45 | 1 peptides | 1 peptides | 10% |
| TRP_20 | 6 | Tb927.7.4480/Tb07.26A24.920 | 35 | 1 peptides | 1 peptides | 8% |
| TRP_20 | 8 | Tb09.160.3590 | 31 | 1 peptides | 1 peptides | 1% |
| TRP_20 | 9 | Tb11.01.7830 | 31 | 1 peptides | 1 peptides | 3% |

| | | | | | | |
|---|---|---|---|---|---|---|
| TRP_20 | 14 | Tb927.4.2740/Tb04.2H8.1370 | 25 | 1 peptides | 1 peptides | 8% |
| TRP_21 | 1 | Tb10.26.1080 | 158 | 4 peptides | 2 peptides | 6% |
| TRP_21 | 2 | Tb09.211.3540 | 149 | 3 peptides | 1 peptides | 5% |
| TRP_21 | 3 | Tb10.70.2650 | 140 | 4 peptides | 2 peptides | 5% |
| TRP_21 | 4 | Tb11.01.1350 | 114 | 4 peptides | 1 peptides | 9% |
| TRP_21 | 5 | Tb11.01.7010 | 108 | 3 peptides | 2 peptides | 2% |
| TRP_21 | 6 | Tb10.70.1190 | 74 | 2 peptides | 2 peptides | 3% |
| TRP_21 | 8 | Tb927.6.4280/Tb06.26G9.1050 | 61 | 2 peptides | 1 peptides | 6% |
| TRP_21 | 11 | Tb11.01.3080 | 44 | 1 peptides | 1 peptides | 2% |
| TRP_21 | 12 | Tb927.4.3950/Tb04.1D20.740 | 43 | 2 peptides | 1 peptides | 1% |
| TRP_21 | 16 | Tb10.6k15.1220 | 32 | 2 peptides | 1 peptides | 1% |
| TRP_21 | 34 | Tb10.389.1430 | 24 | 2 peptides | 1 peptides | 0% |
| TRP_22 | 1 | Tb11.02.5450 | 401 | 6 peptides | 5 peptides | 10% |
| TRP_22 | 2 | Tb10.26.1080 | 342 | 9 peptides | 4 peptides | 13% |
| TRP_22 | 3 | Tb11.01.3110 | 261 | 9 peptides | 7 peptides | 10% |
| TRP_22 | 4 | Tb10.70.1190 | 256 | 6 peptides | 3 peptides | 7% |
| TRP_22 | 5 | Tb09.211.3540 | 211 | 5 peptides | 2 peptides | 8% |
| TRP_22 | 6 | Tb10.70.2650 | 114 | 6 peptides | 1 peptides | 5% |
| TRP_22 | 7 | Tb927.7.3550/Tb07.28B13.500 | 109 | 4 peptides | 2 peptides | 3% |
| TRP_22 | 8 | Tb927.3.4290/Tb03.26J7.510 | 99 | 6 peptides | 1 peptides | 10% |
| TRP_22 | 9 | Tb927.3.3580/Tb03.28C22.1060 | 73 | 5 peptides | 1 peptides | 5% |
| TRP_22 | 11 | Tb11.01.1350 | 44 | 2 peptides | 1 peptides | 3% |
| TRP_22 | 12 | Tb11.02.1120 | 44 | 1 peptides | 1 peptides | 2% |
| TRP_22 | 14 | Tb10.70.1370 | 41 | 2 peptides | 1 peptides | 6% |
| TRP_22 | 23 | Tb11.55.0006 | 30 | 2 peptides | 1 peptides | 2% |
| TRP_22 | 27 | Tb927.4.1060/Tb04.5E12.400 | 26 | 2 peptides | 1 peptides | 2% |
| TRP_23 | 1 | Tb10.26.1080 | 603 | 13 peptides | 8 peptides | 19% |
| TRP_23 | 2 | Tb927.3.4290/Tb03.26J7.510 | 382 | 6 peptides | 4 peptides | 11% |
| TRP_23 | 3 | Tb09.211.3540 | 274 | 5 peptides | 3 peptides | 11% |
| TRP_23 | 4 | Tb10.6k15.2620 | 251 | 6 peptides | 5 peptides | 11% |
| TRP_23 | 5 | Tb927.4.1080/Tb04.5E12.370 | 232 | 6 peptides | 2 peptides | 10% |
| TRP_23 | 6 | Tb10.70.2650 | 139 | 5 peptides | 1 peptides | 5% |
| TRP_23 | 7 | Tb927.8.4970/Tb08.5H5.920 | 127 | 4 peptides | 1 peptides | 9% |
| TRP_23 | 8 | Tb11.02.0070 | 122 | 2 peptides | 1 peptides | 3% |
| TRP_23 | 9 | Tb927.7.3550/Tb07.28B13.500 | 109 | 2 peptides | 2 peptides | 1% |
| TRP_23 | 10 | Tb11.02.1120 | 109 | 1 peptides | 1 peptides | 2% |

| | | | | | | |
|---|---|---|---|---|---|---|
| TRP_23 | 11 | Tb11.02.5450 | 107 | 2 peptides | 2 peptides | 3% |
| TRP_23 | 15 | Tb11.01.3110 | 36 | 2 peptides | 1 peptides | 4% |
| TRP_23 | 16 | Tb11.01.2530 | 36 | 1 peptides | 1 peptides | 1% |
| TRP_23 | 38 | Tb11.01.8380 | 21 | 1 peptides | 1 peptides | 1% |
| TRP_24 | 1 | Tb09.211.3550 | 539 | 11 peptides | 9 peptides | 20% |
| TRP_24 | 2 | Tb10.70.2650 | 176 | 5 peptides | 4 peptides | 7% |
| TRP_24 | 3 | Tb10.70.4740 | 169 | 3 peptides | 1 peptides | 9% |
| TRP_24 | 4 | Tb11.01.3560 | 85 | 1 peptides | 1 peptides | 4% |
| TRP_24 | 5 | Tb11.02.0070 | 85 | 4 peptides | 1 peptides | 8% |
| TRP_24 | 6 | Tb927.3.4290/Tb03.26J7.510 | 81 | 2 peptides | 1 peptides | 3% |
| TRP_24 | 7 | Tb11.02.4440 | 72 | 2 peptides | 1 peptides | 4% |
| TRP_24 | 8 | Tb10.406.0520 | 50 | 1 peptides | 1 peptides | 4% |
| TRP_24 | 12 | Tb927.4.5010/Tb04.3M17.390 | 39 | 1 peptides | 1 peptides | 2% |
| TRP_24 | 14 | Tb10.61.2130 | 34 | 1 peptides | 1 peptides | 1% |
| TRP_24 | 42 | Tb11.01.8380 | 22 | 1 peptides | 1 peptides | 1% |
| TRP_25 | 1 | Tb09.211.3540 | 373 | 10 peptides | 6 peptides | 15% |
| TRP_25 | 2 | Tb10.70.4740 | 267 | 4 peptides | 3 peptides | 13% |
| TRP_25 | 3 | Tb11.01.1350 | 201 | 5 peptides | 4 peptides | 10% |
| TRP_25 | 4 | Tb927.1.700 | 165 | 5 peptides | 4 peptides | 10% |
| TRP_25 | 5 | Tb10.70.2650 | 86 | 3 peptides | 2 peptides | 4% |
| TRP_25 | 6 | Tb11.01.3560 | 65 | 2 peptides | 2 peptides | 4% |
| TRP_25 | 7 | Tb11.01.8470 | 56 | 3 peptides | 1 peptides | 8% |
| TRP_25 | 8 | Tb927.6.4280/Tb06.26G9.1050 | 41 | 1 peptides | 1 peptides | 2% |
| TRP_25 | 35 | Tb11.01.8380 | 22 | 1 peptides | 1 peptides | 1% |
| TRP_26 | 1 | Tb10.70.4740 | 291 | 6 peptides | 5 peptides | 13% |
| TRP_26 | 2 | Tb927.1.700 | 249 | 6 peptides | 5 peptides | 11% |
| TRP_26 | 3 | Tb11.01.1350 | 208 | 6 peptides | 3 peptides | 9% |
| TRP_26 | 5 | Tb10.70.5800 | 99 | 1 peptides | 1 peptides | 3% |
| TRP_26 | 6 | Tb10.70.2650 | 69 | 3 peptides | 1 peptides | 3% |
| TRP_26 | 7 | Tb927.3.4680/Tb03.48K5.180 | 59 | 1 peptides | 1 peptides | 2% |
| TRP_26 | 9 | Tb10.6k15.3250 | 38 | 1 peptides | 1 peptides | 1% |
| TRP_26 | 10 | Tb11.01.8380 | 33 | 1 peptides | 1 peptides | 1% |
| TRP_26 | 20 | Tb09.160.0430 | 27 | 1 peptides | 1 peptides | 1% |
| TRP_27 | 1 | Tb10.70.1370 | 226 | 5 peptides | 2 peptides | 12% |
| TRP_27 | 2 | Tb11.01.1350 | 211 | 6 peptides | 4 peptides | 12% |
| TRP_27 | 3 | Tb927.8.3530/Tb08.28L1.740 | 155 | 3 peptides | 1 peptides | 11% |

| | | | | | | |
|---|---|---|---|---|---|---|
| TRP_27 | 4 | Tb927.1.700 | 146 | 5 peptides | 3 peptides | 8% |
| TRP_27 | 5 | Tb927.6.4280/Tb06.26G9.1050 | 139 | 6 peptides | 1 peptides | 15% |
| TRP_27 | 7 | Tb927.6.4840/Tb06.30P15.650 | 39 | 1 peptides | 1 peptides | 3% |
| TRP_27 | 10 | Tb11.01.1440 | 29 | 1 peptides | 1 peptides | 2% |
| TRP_28 | 1 | Tb10.70.1370 | 292 | 6 peptides | 4 peptides | 15% |
| TRP_28 | 2 | Tb927.6.4280/Tb06.26G9.1050 | 269 | 6 peptides | 4 peptides | 14% |
| TRP_28 | 3 | Tb927.8.3530/Tb08.28L1.740 | 199 | 3 peptides | 2 peptides | 11% |
| TRP_28 | 4 | Tb10.26.1080 | 120 | 4 peptides | 2 peptides | 4% |
| TRP_28 | 5 | Tb09.160.4560 | 109 | 3 peptides | 2 peptides | 6% |
| TRP_28 | 6 | Tb10.6k15.3250 | 108 | 2 peptides | 2 peptides | 3% |
| TRP_28 | 7 | Tb10.70.3710 | 99 | 1 peptides | 1 peptides | 2% |
| TRP_28 | 14 | Tb11.01.8380 | 36 | 2 peptides | 1 peptides | 2% |
| TRP_29 | 1 | Tb10.26.1080 | 423 | 11 peptides | 7 peptides | 11% |
| TRP_29 | 2 | Tb927.8.3530/Tb08.28L1.740 | 399 | 7 peptides | 6 peptides | 18% |
| TRP_29 | 3 | Tb10.70.1370 | 341 | 8 peptides | 4 peptides | 16% |
| TRP_29 | 4 | Tb927.6.4280/Tb06.26G9.1050 | 172 | 5 peptides | 4 peptides | 11% |
| TRP_29 | 5 | Tb11.01.3170 | 122 | 4 peptides | 2 peptides | 12% |
| TRP_29 | 6 | Tb10.6k15.3850 | 115 | 3 peptides | 1 peptides | 9% |
| TRP_29 | 7 | Tb09.211.3610 | 63 | 4 peptides | 1 peptides | 3% |
| TRP_29 | 9 | Tb927.7.6210/Tb07.2F2.530 | 36 | 3 peptides | 1 peptides | 5% |
| TRP_29 | 12 | Tb927.4.5460/Tb04.24M18.460 | 34 | 1 peptides | 1 peptides | 1% |
| TRP_29 | 15 | Tb10.70.2650 | 31 | 2 peptides | 1 peptides | 4% |
| TRP_29 | 16 | Tb927.4.4240/Tb04.1D20.250 | 30 | 1 peptides | 1 peptides | 2% |
| TRP_29 | 33 | Tb11.01.1440 | 25 | 1 peptides | 1 peptides | 2% |
| TRP_30 | 1 | Tb10.26.1080 | 564 | 12 peptides | 7 peptides | 19% |
| TRP_30 | 2 | Tb927.8.3530/Tb08.28L1.740 | 370 | 7 peptides | 6 peptides | 18% |
| TRP_30 | 3 | Tb10.70.2650 | 197 | 5 peptides | 4 peptides | 6% |
| TRP_30 | 4 | Tb10.6k15.3850 | 195 | 4 peptides | 3 peptides | 15% |
| TRP_30 | 5 | Tb11.01.3170 | 172 | 4 peptides | 2 peptides | 11% |
| TRP_30 | 6 | Tb927.6.4280/Tb06.26G9.1050 | 162 | 4 peptides | 2 peptides | 11% |
| TRP_30 | 7 | Tb09.160.3710 | 90 | 1 peptides | 1 peptides | 4% |
| TRP_30 | 8 | Tb09.211.3610 | 73 | 4 peptides | 2 peptides | 3% |
| TRP_30 | 9 | Tb927.7.1300/Tb07.27M11.560 | 48 | 1 peptides | 1 peptides | 2% |
| TRP_31 | 1 | Tb10.26.1080 | 563 | 14 peptides | 9 peptides | 14% |
| TRP_31 | 2 | Tb10.6k15.3850 | 310 | 5 peptides | 3 peptides | 19% |
| TRP_31 | 3 | Tb927.8.3530/Tb08.28L1.740 | 307 | 7 peptides | 6 peptides | 18% |

| | | | | | | |
|---|---|---|---|---|---|---|
| TRP_31 | 4 | Tb10.70.2650 | 256 | 7 peptides | 5 peptides | 8% |
| TRP_31 | 5 | Tb11.01.3170 | 180 | 6 peptides | 3 peptides | 18% |
| TRP_31 | 6 | Tb10.61.0540 | 94 | 2 peptides | 2 peptides | 6% |
| TRP_31 | 8 | Tb11.01.5860 | 55 | 1 peptides | 1 peptides | 2% |
| TRP_31 | 9 | Tb11.01.3040 | 54 | 1 peptides | 1 peptides | 3% |
| TRP_31 | 11 | Tb927.4.5460/Tb04.24M18.460 | 43 | 1 peptides | 1 peptides | 1% |
| TRP_31 | 17 | Tb09.160.3710 | 34 | 1 peptides | 1 peptides | 5% |
| TRP_31 | 19 | Tb927.6.1570/Tb06.28P18.310 | 34 | 1 peptides | 1 peptides | 3% |
| TRP_32 | 1 | Tb927.3.2230/Tb03.48O8.710 | 142 | 5 peptides | 4 peptides | 18% |
| TRP_32 | 2 | Tb11.02.5450 | 128 | 2 peptides | 2 peptides | 2% |
| TRP_32 | 3 | Tb11.02.4700 | 112 | 4 peptides | 2 peptides | 20% |
| TRP_32 | 4 | Tb11.01.3110 | 93 | 2 peptides | 1 peptides | 2% |
| TRP_32 | 5 | Tb11.01.1290 | 60 | 4 peptides | 1 peptides | 11% |
| TRP_32 | 6 | Tb927.1.2330 | 51 | 1 peptides | 1 peptides | 2% |
| TRP_32 | 7 | Tb10.70.2650 | 51 | 3 peptides | 1 peptides | 4% |
| TRP_32 | 8 | Tb09.v1.0380 | 46 | 3 peptides | 1 peptides | 5% |
| TRP_32 | 9 | Tb927.7.1300/Tb07.27M11.560 | 43 | 3 peptides | 1 peptides | 7% |
| TRP_32 | 10 | Tb10.61.0540 | 37 | 2 peptides | 1 peptides | 3% |
| TRP_33 | 1 | Tb11.02.3210 | 196 | 3 peptides | 3 peptides | 17% |
| TRP_33 | 2 | Tb11.02.4700 | 166 | 4 peptides | 4 peptides | 13% |
| TRP_33 | 3 | Tb11.02.5450 | 146 | 4 peptides | 2 peptides | 6% |
| TRP_33 | 4 | Tb09.160.3820 | 134 | 3 peptides | 2 peptides | 6% |
| TRP_33 | 5 | Tb10.26.1080 | 132 | 3 peptides | 2 peptides | 5% |
| TRP_33 | 6 | Tb11.01.1290 | 119 | 4 peptides | 2 peptides | 8% |
| TRP_33 | 7 | Tb927.1.2330 | 92 | 2 peptides | 1 peptides | 5% |
| TRP_33 | 8 | Tb927.6.4280/Tb06.26G9.1050 | 75 | 3 peptides | 1 peptides | 9% |
| TRP_33 | 9 | Tb10.6k15.3080 | 70 | 2 peptides | 2 peptides | 3% |
| TRP_33 | 10 | Tb11.02.4870 | 70 | 2 peptides | 1 peptides | 8% |
| TRP_33 | 11 | Tb927.7.710/Tb07.29K4.620 | 65 | 2 peptides | 1 peptides | 2% |
| TRP_33 | 12 | Tb10.70.3290 | 59 | 1 peptides | 1 peptides | 2% |
| TRP_33 | 13 | Tb927.8.1990/Tb08.26N11.460 | 55 | 1 peptides | 1 peptides | 4% |
| TRP_33 | 14 | Tb11.01.3110 | 52 | 1 peptides | 1 peptides | 1% |
| TRP_33 | 15 | Tb11.01.5680 | 46 | 1 peptides | 1 peptides | 2% |
| TRP_33 | 16 | Tb10.6k15.1160 | 43 | 1 peptides | 1 peptides | 3% |
| TRP_33 | 17 | Tb927.7.1300/Tb07.27M11.560 | 42 | 1 peptides | 1 peptides | 2% |
| TRP_33 | 18 | Tb927.7.3550/Tb07.28B13.500 | 42 | 2 peptides | 1 peptides | 1% |

| | | | | | | |
|---|---|---|---|---|---|---|
| TRP_33 | 19 | Tb09.160.3270 | 36 | 2 peptides | 1 peptides | 6% |
| TRP_34 | 1 | Tb11.02.3210 | 216 | 3 peptides | 3 peptides | 16% |
| TRP_34 | 2 | Tb927.8.1990/Tb08.26N11.460 | 136 | 3 peptides | 2 peptides | 12% |
| TRP_34 | 3 | Tb10.100.0170 | 117 | 1 peptides | 1 peptides | 5% |
| TRP_34 | 4 | Tb10.26.1080 | 80 | 1 peptides | 1 peptides | 1% |
| TRP_34 | 5 | Tb10.6k15.3080 | 80 | 3 peptides | 2 peptides | 3% |
| TRP_34 | 6 | Tb927.7.4520/Tb07.26A24.810 | 74 | 2 peptides | 2 peptides | 5% |
| TRP_34 | 7 | Tb09.211.2590 | 73 | 1 peptides | 1 peptides | 3% |
| TRP_34 | 8 | Tb11.01.4660 | 70 | 1 peptides | 1 peptides | 2% |
| TRP_34 | 9 | Tb11.01.2000 | 70 | 2 peptides | 2 peptides | 10% |
| TRP_34 | 10 | Tb09.160.4250 | 65 | 1 peptides | 1 peptides | 5% |
| TRP_34 | 11 | Tb927.1.2330 | 65 | 1 peptides | 1 peptides | 2% |
| TRP_34 | 12 | Tb11.02.5450 | 61 | 1 peptides | 1 peptides | 1% |
| TRP_34 | 13 | Tb927.5.1160/Tb05.30H13.70 | 59 | 1 peptides | 1 peptides | 3% |
| TRP_34 | 14 | Tb11.02.3390 | 58 | 2 peptides | 1 peptides | 11% |
| TRP_34 | 15 | Tb10.6k15.1160 | 58 | 1 peptides | 1 peptides | 3% |
| TRP_34 | 17 | Tb09.211.0110 | 46 | 1 peptides | 1 peptides | 6% |
| TRP_34 | 18 | Tb11.01.3110 | 45 | 1 peptides | 1 peptides | 1% |
| TRP_34 | 22 | Tb09.160.1160 | 37 | 1 peptides | 1 peptides | 1% |
| TRP_34 | 24 | Tb927.3.1990/Tb03.30P12.930 | 31 | 2 peptides | 1 peptides | 4% |
| TRP_35 | 1 | Tb11.03.0410 | 171 | 3 peptides | 3 peptides | 27% |
| TRP_35 | 2 | Tb927.8.1990/Tb08.26N11.460 | 137 | 3 peptides | 2 peptides | 12% |
| TRP_35 | 3 | Tb10.100.0170 | 118 | 2 peptides | 1 peptides | 10% |
| TRP_35 | 4 | Tb11.02.3210 | 85 | 1 peptides | 1 peptides | 5% |
| TRP_35 | 5 | Tb10.70.7730 | 82 | 1 peptides | 1 peptides | 2% |
| TRP_35 | 6 | Tb11.01.7800 | 82 | 1 peptides | 1 peptides | 11% |
| TRP_35 | 7 | Tb09.211.2590 | 71 | 2 peptides | 1 peptides | 9% |
| TRP_35 | 8 | Tb927.7.7460/Tb07.30D13.320 | 62 | 1 peptides | 1 peptides | 5% |
| TRP_35 | 9 | Tb927.3.780/Tb03.27F10.710 | 57 | 1 peptides | 1 peptides | 3% |
| TRP_35 | 10 | Tb11.01.4660 | 55 | 1 peptides | 1 peptides | 2% |
| TRP_35 | 11 | Tb10.70.4880 | 54 | 1 peptides | 1 peptides | 2% |
| TRP_35 | 12 | Tb09.160.3270 | 53 | 1 peptides | 1 peptides | 2% |
| TRP_35 | 13 | Tb927.5.1160/Tb05.30H13.70 | 51 | 1 peptides | 1 peptides | 3% |
| TRP_35 | 14 | Tb10.70.3290 | 50 | 1 peptides | 1 peptides | 2% |
| TRP_35 | 15 | Tb927.6.4280/Tb06.26G9.1050 | 48 | 2 peptides | 1 peptides | 6% |
| TRP_35 | 18 | Tb11.02.3390 | 39 | 1 peptides | 1 peptides | 5% |

| | | | | | | |
|---|---|---|---|---|---|---|
| TRP_35 | 19 | Tb927.2.1560/27H14.160 | 39 | 1 peptides | 1 peptides | 3% |
| TRP_35 | 21 | Tb10.6k15.1160 | 37 | 1 peptides | 1 peptides | 3% |
| TRP_35 | 23 | Tb10.26.1080 | 36 | 3 peptides | 1 peptides | 3% |
| TRP_36 | 1 | Tb927.8.1990/Tb08.26N11.460 | 223 | 6 peptides | 4 peptides | 25% |
| TRP_36 | 2 | Tb11.01.7800 | 149 | 2 peptides | 2 peptides | 16% |
| TRP_36 | 3 | Tb09.160.3270 | 122 | 4 peptides | 1 peptides | 7% |
| TRP_36 | 4 | Tb927.1.2330 | 100 | 3 peptides | 2 peptides | 7% |
| TRP_36 | 5 | Tb927.1.2340 | 99 | 3 peptides | 1 peptides | 7% |
| TRP_36 | 6 | Tb10.70.3290 | 91 | 2 peptides | 2 peptides | 5% |
| TRP_36 | 7 | Tb10.61.1750 | 80 | 2 peptides | 1 peptides | 2% |
| TRP_36 | 8 | Tb10.70.7730 | 77 | 2 peptides | 1 peptides | 5% |
| TRP_36 | 9 | Tb927.7.7460/Tb07.30D13.320 | 70 | 1 peptides | 1 peptides | 5% |
| TRP_36 | 11 | Tb11.03.0410 | 63 | 1 peptides | 1 peptides | 9% |
| TRP_36 | 12 | Tb11.01.4660 | 54 | 2 peptides | 1 peptides | 2% |
| TRP_36 | 13 | Tb927.6.4280/Tb06.26G9.1050 | 52 | 2 peptides | 1 peptides | 6% |
| TRP_36 | 14 | Tb927.5.1160/Tb05.30H13.70 | 50 | 1 peptides | 1 peptides | 3% |
| TRP_36 | 15 | Tb09.211.0740 | 48 | 1 peptides | 1 peptides | 5% |
| TRP_36 | 16 | Tb10.70.4880 | 47 | 1 peptides | 1 peptides | 2% |
| TRP_36 | 20 | Tb11.03.0250 | 36 | 1 peptides | 1 peptides | 5% |
| TRP_36 | 29 | Tb927.3.1990/Tb03.30P12.930 | 26 | 1 peptides | 1 peptides | 2% |
| TRP_36 | 30 | Tb927.7.4500/Tb07.26A24.860 | 25 | 1 peptides | 1 peptides | 1% |
| TRP_36 | 85 | Tb927.8.1050/Tb08.29O4.100 | 16 | 2 peptides | 1 peptides | 0% |
| TRP_37 | 1 | Tb11.03.0410 | 208 | 6 peptides | 4 peptides | 32% |
| TRP_37 | 2 | Tb09.160.3270 | 159 | 4 peptides | 2 peptides | 7% |
| TRP_37 | 3 | Tb11.01.7800 | 135 | 3 peptides | 2 peptides | 18% |
| TRP_37 | 4 | Tb927.2.1560/27H14.160 | 131 | 5 peptides | 1 peptides | 16% |
| TRP_37 | 5 | Tb11.03.0250 | 121 | 2 peptides | 2 peptides | 11% |
| TRP_37 | 6 | Tb927.6.4280/Tb06.26G9.1050 | 90 | 3 peptides | 2 peptides | 9% |
| TRP_37 | 7 | Tb10.70.7730 | 80 | 2 peptides | 1 peptides | 5% |
| TRP_37 | 9 | Tb927.1.2330 | 52 | 2 peptides | 1 peptides | 5% |
| TRP_37 | 10 | Tb927.1.2340 | 51 | 2 peptides | 1 peptides | 3% |
| TRP_37 | 11 | Tb927.4.2080/Tb04.29M18.770 | 46 | 1 peptides | 1 peptides | 1% |
| TRP_37 | 13 | Tb11.02.5450 | 43 | 1 peptides | 1 peptides | 1% |
| TRP_38 | 1 | Tb09.160.3270 | 237 | 5 peptides | 3 peptides | 10% |
| TRP_38 | 2 | Tb11.01.7800 | 163 | 4 peptides | 2 peptides | 24% |
| TRP_38 | 3 | Tb927.1.2330 | 100 | 2 peptides | 2 peptides | 5% |

| | | | | | | |
|---|---|---|---|---|---|---|
| TRP_38 | 4 | Tb10.70.2650 | 77 | 2 peptides | 1 peptides | 3% |
| TRP_38 | 5 | Tb10.6k15.2050 | 67 | 1 peptides | 1 peptides | 7% |
| TRP_38 | 6 | Tb10.70.7730 | 61 | 1 peptides | 1 peptides | 2% |
| TRP_38 | 9 | Tb11.02.0815 | 39 | 1 peptides | 1 peptides | 7% |
| TRP_38 | 35 | Tb11.03.0250 | 20 | 1 peptides | 1 peptides | 5% |
| TRP_39 | 1 | Tb11.01.7800 | 248 | 6 peptides | 5 peptides | 30% |
| TRP_39 | 2 | Tb927.1.2340 | 83 | 3 peptides | 1 peptides | 5% |
| TRP_39 | 3 | Tb927.1.2330 | 78 | 2 peptides | 1 peptides | 4% |
| TRP_39 | 4 | Tb927.3.5180/Tb03.5L5.210 | 75 | 1 peptides | 1 peptides | 10% |
| TRP_39 | 5 | Tb10.6k15.2050 | 71 | 1 peptides | 1 peptides | 7% |
| TRP_39 | 6 | Tb11.02.1085 | 58 | 1 peptides | 1 peptides | 3% |
| TRP_39 | 7 | Tb11.02.0815 | 49 | 1 peptides | 1 peptides | 7% |
| TRP_39 | 8 | Tb927.4.2080/Tb04.29M18.770 | 46 | 1 peptides | 1 peptides | 1% |
| TRP_39 | 11 | Tb09.160.4450 | 41 | 1 peptides | 1 peptides | 3% |
| TRP_39 | 12 | Tb927.5.1000/Tb05.28F8.340 | 40 | 1 peptides | 1 peptides | 7% |
| TRP_39 | 13 | Tb927.3.4000/Tb03.28C22.160 | 39 | 1 peptides | 1 peptides | 4% |
| TRP_39 | 17 | Tb927.4.2740/Tb04.2H8.1370 | 32 | 1 peptides | 1 peptides | 8% |
| TRP_40 | 1 | Tb927.1.2330 | 124 | 3 peptides | 2 peptides | 7% |
| TRP_40 | 2 | Tb927.1.2340 | 112 | 3 peptides | 1 peptides | 7% |
| TRP_40 | 3 | Tb10.70.2520 | 79 | 2 peptides | 1 peptides | 19% |
| TRP_40 | 4 | Tb927.8.6430/Tb08.11J15.110 | 78 | 2 peptides | 2 peptides | 22% |
| TRP_40 | 5 | Tb10.406.0615 | 75 | 1 peptides | 1 peptides | 8% |
| TRP_40 | 6 | Tb10.70.7730 | 71 | 1 peptides | 1 peptides | 2% |
| TRP_40 | 7 | Tb10.6k15.3340 | 58 | 1 peptides | 1 peptides | 9% |
| TRP_40 | 8 | Tb927.5.4170/Tb05.45E22.470 | 55 | 1 peptides | 1 peptides | 12% |
| TRP_40 | 9 | Tb11.01.7800 | 52 | 1 peptides | 1 peptides | 11% |
| TRP_40 | 10 | Tb10.26.0680 | 52 | 1 peptides | 1 peptides | 10% |
| TRP_40 | 11 | Tb10.70.7020 | 49 | 1 peptides | 1 peptides | 7% |
| TRP_40 | 12 | Tb11.50.0005 | 43 | 1 peptides | 1 peptides | 6% |
| TRP_40 | 13 | Tb09.211.4511 | 42 | 1 peptides | 1 peptides | 9% |
| TRP_40 | 14 | Tb927.7.3420/Tb07.28B13.650 | 39 | 1 peptides | 1 peptides | 7% |
| TRP_40 | 15 | Tb09.211.4460 | 37 | 1 peptides | 1 peptides | 6% |
| TRP_40 | 16 | Tb09.211.0560 | 33 | 1 peptides | 1 peptides | 3% |
| TRP_40 | 18 | Tb927.4.2740/Tb04.2H8.1370 | 29 | 1 peptides | 1 peptides | 8% |
| TRP_40 | 20 | Tb10.70.3160 | 28 | 1 peptides | 1 peptides | 10% |
| TRP_40 | 23 | Tb927.8.6440/Tb08.11J15.100 | 25 | 1 peptides | 1 peptides | 4% |

| | | | | | | |
|---|---|---|---|---|---|---|
| TRP_40_1 | 1 | Tb09.211.3540 | 490 | 9 peptides | 6 peptides | 16% |
| TRP_40_1 | 2 | Tb927.8.3530/Tb08.28L1.740 | 365 | 6 peptides | 6 peptides | 17% |
| TRP_40_1 | 3 | Tb11.01.1350 | 302 | 7 peptides | 4 peptides | 14% |
| TRP_40_1 | 4 | Tb10.26.1080 | 232 | 4 peptides | 4 peptides | 8% |
| TRP_40_1 | 5 | Tb927.6.4280/Tb06.26G9.1050 | 220 | 5 peptides | 4 peptides | 14% |
| TRP_40_1 | 6 | Tb10.70.2650 | 166 | 5 peptides | 1 peptides | 5% |
| TRP_40_1 | 7 | Tb10.6k15.3850 | 108 | 3 peptides | 1 peptides | 9% |
| TRP_40_1 | 8 | Tb11.02.0070 | 92 | 1 peptides | 1 peptides | 2% |
| TRP_40_1 | 9 | Tb11.01.8470 | 76 | 1 peptides | 1 peptides | 4% |
| TRP_40_1 | 10 | Tb11.01.3560 | 74 | 2 peptides | 2 peptides | 6% |
| TRP_40_1 | 12 | Tb11.02.0290 | 60 | 1 peptides | 1 peptides | 2% |
| TRP_40_1 | 13 | Tb11.02.1120 | 58 | 1 peptides | 1 peptides | 2% |
| TRP_40_1 | 14 | Tb927.4.1080/Tb04.5E12.370 | 58 | 2 peptides | 1 peptides | 2% |
| TRP_40_1 | 15 | Tb10.70.1370 | 50 | 1 peptides | 1 peptides | 3% |
| TRP_40_1 | 21 | Tb927.3.4290/Tb03.26J7.510 | 32 | 2 peptides | 1 peptides | 3% |
| TRP_40_1 | 23 | Tb11.02.0490 | 29 | 1 peptides | 1 peptides | 1% |
| TRP_40_1 | 32 | Tb11.01.2480 | 24 | 1 peptides | 1 peptides | 1% |
| TRP_40_2 | 1 | Tb09.211.3540 | 335 | 5 peptides | 5 peptides | 11% |
| TRP_40_2 | 2 | Tb927.8.3530/Tb08.28L1.740 | 270 | 5 peptides | 4 peptides | 16% |
| TRP_40_2 | 3 | Tb10.70.2650 | 251 | 7 peptides | 2 peptides | 7% |
| TRP_40_2 | 4 | Tb11.01.1350 | 148 | 4 peptides | 3 peptides | 7% |
| TRP_40_2 | 5 | Tb927.6.4280/Tb06.26G9.1050 | 134 | 3 peptides | 3 peptides | 8% |
| TRP_40_2 | 6 | Tb10.6k15.2620 | 112 | 4 peptides | 2 peptides | 9% |
| TRP_40_2 | 7 | Tb10.70.1370 | 102 | 2 peptides | 2 peptides | 7% |
| TRP_40_2 | 8 | Tb10.6k15.3850 | 101 | 2 peptides | 2 peptides | 8% |
| TRP_40_2 | 9 | Tb10.26.1080 | 91 | 1 peptides | 1 peptides | 1% |
| TRP_40_2 | 10 | Tb10.70.1190 | 82 | 1 peptides | 1 peptides | 1% |
| TRP_40_2 | 11 | Tb10.61.2680 | 71 | 2 peptides | 1 peptides | 7% |
| TRP_40_2 | 12 | Tb927.3.4290/Tb03.26J7.510 | 57 | 2 peptides | 1 peptides | 4% |
| TRP_40_2 | 13 | Tb11.01.3560 | 51 | 2 peptides | 1 peptides | 4% |
| TRP_40_2 | 14 | Tb927.4.3950/Tb04.1D20.740 | 45 | 2 peptides | 1 peptides | 2% |
| TRP_40_2 | 94 | Tb927.2.5150/30J2.25 | 16 | 1 peptides | 1 peptides | 2% |
| TRP_53 | 1 | Tb11.02.5170 | 278 | 5 peptides | 3 peptides | 22% |
| TRP_53 | 2 | Tb09.160.4250 | 216 | 4 peptides | 3 peptides | 19% |
| TRP_53 | 3 | Tb927.7.4790/Tb07.26A24.340 | 188 | 4 peptides | 4 peptides | 18% |
| TRP_53 | 4 | Tb10.100.0170 | 162 | 3 peptides | 2 peptides | 14% |

| | | | | | | |
|---|---|---|---|---|---|---|
| TRP_53 | 5 | Tb927.7.3440/Tb07.28B13.630 | 140 | 2 peptides | 2 peptides | 10% |
| TRP_53 | 6 | Tb10.70.2490 | 138 | 3 peptides | 2 peptides | 15% |
| TRP_53 | 7 | Tb11.01.3110 | 116 | 3 peptides | 1 peptides | 5% |
| TRP_53 | 8 | Tb927.8.5440/Tb08.5H5.50 | 83 | 1 peptides | 1 peptides | 6% |
| TRP_53 | 9 | Tb927.1.2340 | 79 | 2 peptides | 2 peptides | 3% |
| TRP_53 | 10 | Tb11.01.3020 | 74 | 1 peptides | 1 peptides | 6% |
| TRP_53 | 11 | Tb10.70.6540 | 74 | 1 peptides | 1 peptides | 5% |
| TRP_53 | 12 | Tb10.70.0850 | 48 | 1 peptides | 1 peptides | 4% |
| TRP_53 | 14 | Tb09.211.2590 | 33 | 1 peptides | 1 peptides | 3% |
| TRP_54 | 1 | Tb10.70.0790 | 215 | 5 peptides | 3 peptides | 14% |
| TRP_54 | 2 | Tb927.6.4990/Tb06.30P15.500 | 95 | 2 peptides | 1 peptides | 10% |
| TRP_54 | 3 | Tb11.02.5170 | 45 | 1 peptides | 1 peptides | 7% |
| TRP_54 | 4 | Tb11.01.3080 | 38 | 1 peptides | 1 peptides | 1% |
| TRP_54 | 17 | Tb927.1.2340 | 18 | 1 peptides | 1 peptides | 3% |
| TRP_55 | 1 | Tb09.211.4460 | 291 | 5 peptides | 4 peptides | 28% |
| TRP_55 | 2 | Tb11.03.0250 | 246 | 5 peptides | 4 peptides | 26% |
| TRP_55 | 3 | Tb927.1.2340 | 50 | 2 peptides | 1 peptides | 3% |
| TRP_56 | 1 | Tb09.211.4460 | 173 | 3 peptides | 3 peptides | 18% |
| TRP_56 | 2 | Tb11.02.4870 | 115 | 3 peptides | 2 peptides | 12% |
| TRP_56 | 3 | Tb927.1.2340 | 58 | 1 peptides | 1 peptides | 3% |
| TRP_56 | 4 | Tb11.03.0250 | 46 | 1 peptides | 1 peptides | 5% |
| TRP_56 | 6 | Tb927.3.3450/Tb03.25B21.123 | 29 | 1 peptides | 1 peptides | 5% |
| TRP_57 | 1 | Tb10.70.0850 | 721 | 16 peptides | 10 peptides | 37% |
| TRP_57 | 2 | Tb927.4.2030/Tb04.29M18.690 | 78 | 2 peptides | 2 peptides | 5% |
| TRP_57 | 3 | Tb11.02.4700 | 57 | 1 peptides | 1 peptides | 3% |
| TRP_58 | 1 | Tb11.02.4870 | 686 | 16 peptides | 11 peptides | 42% |
| TRP_58 | 2 | Tb10.100.0120 | 586 | 8 peptides | 7 peptides | 41% |
| TRP_58 | 3 | Tb927.3.780/Tb03.27F10.710 | 204 | 6 peptides | 3 peptides | 27% |
| TRP_58 | 13 | Tb11.03.0950 | 27 | 1 peptides | 1 peptides | 2% |
| TRP_59 | 1 | Tb11.02.4870 | 506 | 11 peptides | 6 peptides | 36% |
| TRP_59 | 2 | Tb927.3.780/Tb03.27F10.710 | 319 | 6 peptides | 4 peptides | 25% |
| TRP_59 | 3 | Tb10.100.0120 | 235 | 4 peptides | 4 peptides | 19% |
| TRP_59 | 4 | Tb09.211.1250 | 160 | 5 peptides | 2 peptides | 18% |
| TRP_59 | 5 | Tb927.6.1260/Tb06.3A7.810 | 73 | 1 peptides | 1 peptides | 3% |
| TRP_60 | 1 | Tb927.3.780/Tb03.27F10.710 | 180 | 4 peptides | 2 peptides | 16% |
| TRP_60 | 2 | Tb927.6.1260/Tb06.3A7.810 | 92 | 2 peptides | 2 peptides | 9% |

| | | | | | | |
|---|---|---|---|---|---|---|
| TRP_60 | 4 | Tb11.02.4870 | 56 | 1 peptides | 1 peptides | 4% |
| TRP_60 | 5 | Tb11.01.1190 | 41 | 2 peptides | 1 peptides | 8% |
| TRP_60 | 6 | Tb927.4.2030/Tb04.29M18.690 | 37 | 1 peptides | 1 peptides | 4% |
| TRP_61 | 1 | Tb09.211.2590 | 225 | 4 peptides | 4 peptides | 11% |
| TRP_61 | 2 | Tb927.4.430/Tb04.5D20.530 | 114 | 3 peptides | 2 peptides | 10% |
| TRP_61 | 3 | Tb10.70.3660 | 106 | 4 peptides | 2 peptides | 16% |
| TRP_61 | 4 | Tb927.6.1260/Tb06.3A7.810 | 101 | 2 peptides | 1 peptides | 7% |
| TRP_61 | 5 | Tb11.01.1190 | 86 | 4 peptides | 1 peptides | 12% |
| TRP_61 | 6 | Tb09.160.4250 | 63 | 2 peptides | 1 peptides | 9% |
| TRP_61 | 7 | Tb927.4.2030/Tb04.29M18.690 | 51 | 1 peptides | 1 peptides | 4% |
| TRP_62 | 1 | Tb09.211.2590 | 391 | 8 peptides | 8 peptides | 24% |
| TRP_62 | 2 | Tb10.100.0170 | 347 | 7 peptides | 7 peptides | 24% |
| TRP_62 | 3 | Tb09.160.4250 | 345 | 6 peptides | 5 peptides | 27% |
| TRP_62 | 4 | Tb10.70.3660 | 280 | 5 peptides | 5 peptides | 24% |
| TRP_62 | 5 | Tb11.02.3210 | 173 | 3 peptides | 3 peptides | 14% |
| TRP_62 | 6 | Tb927.6.1260/Tb06.3A7.810 | 118 | 2 peptides | 2 peptides | 9% |
| TRP_62 | 7 | Tb927.4.430/Tb04.5D20.530 | 113 | 4 peptides | 2 peptides | 13% |
| TRP_62 | 8 | Tb927.7.4790/Tb07.26A24.340 | 102 | 3 peptides | 2 peptides | 11% |
| TRP_62 | 9 | Tb09.160.1200 | 41 | 2 peptides | 1 peptides | 0% |
| TRP_63 | 1 | Tb10.100.0170 | 313 | 7 peptides | 4 peptides | 27% |
| TRP_63 | 2 | Tb09.160.4250 | 289 | 6 peptides | 5 peptides | 26% |
| TRP_63 | 3 | Tb927.7.4790/Tb07.26A24.340 | 258 | 5 peptides | 4 peptides | 18% |
| TRP_63 | 4 | Tb09.211.2590 | 222 | 5 peptides | 4 peptides | 16% |
| TRP_63 | 5 | Tb11.01.3110 | 200 | 3 peptides | 3 peptides | 5% |
| TRP_63 | 6 | Tb11.02.5170 | 190 | 3 peptides | 2 peptides | 16% |
| TRP_63 | 7 | Tb10.70.2490 | 139 | 5 peptides | 2 peptides | 15% |
| TRP_63 | 8 | Tb927.8.1990/Tb08.26N11.460 | 134 | 1 peptides | 1 peptides | 6% |
| TRP_63 | 9 | Tb927.7.3440/Tb07.28B13.630 | 91 | 1 peptides | 1 peptides | 7% |
| TRP_63 | 10 | Tb927.1.2340 | 75 | 1 peptides | 1 peptides | 3% |
| TRP_63 | 11 | Tb927.8.5470/Tb08.26E13.20 | 72 | 2 peptides | 1 peptides | 11% |
| TRP_63 | 12 | Tb11.02.4870 | 60 | 1 peptides | 1 peptides | 3% |
| TRP_63 | 13 | Tb11.01.3020 | 54 | 1 peptides | 1 peptides | 6% |
| TRP_63 | 15 | Tb10.70.0850 | 40 | 1 peptides | 1 peptides | 4% |
| TRP_63 | 18 | Tb09.160.1200 | 29 | 1 peptides | 1 peptides | 0% |
| TRP_63 | 21 | Tb927.7.3100/Tb07.13M20.220 | 26 | 1 peptides | 1 peptides | 1% |
| TRP_64 | 1 | Tb10.70.0790 | 261 | 5 peptides | 5 peptides | 19% |

| | | | | | | |
|---|---|---|---|---|---|---|
| TRP_64 | 2 | Tb927.6.4990/Tb06.30P15.500 | 78 | 1 peptides | 1 peptides | 7% |
| TRP_65 | 1 | Tb927.7.4420/Tb07.26A24.1040 | 279 | 5 peptides | 3 peptides | 16% |
| TRP_65 | 2 | Tb927.1.2340 | 117 | 2 peptides | 2 peptides | 5% |
| TRP_65 | 4 | Tb09.160.0580 | 58 | 1 peptides | 1 peptides | 3% |
| TRP_65 | 7 | Tb10.70.4080 | 30 | 1 peptides | 1 peptides | 0% |
| TRP_66 | 1 | Tb927.4.1080/Tb04.5E12.370 | 1037 | 19 peptides | 14 peptides | 30% |
| TRP_66 | 2 | Tb11.02.1120 | 508 | 12 peptides | 7 peptides | 17% |
| TRP_66 | 3 | Tb927.5.2940/Tb05.26K5.210 | 329 | 5 peptides | 5 peptides | 7% |
| TRP_66 | 4 | Tb927.8.1600/Tb08.29O9.340 | 310 | 7 peptides | 4 peptides | 12% |
| TRP_66 | 5 | Tb10.70.0280 | 202 | 3 peptides | 3 peptides | 6% |
| TRP_66 | 6 | Tb09.160.1180 | 179 | 7 peptides | 4 peptides | 12% |
| TRP_66 | 7 | Tb11.01.3110 | 172 | 3 peptides | 3 peptides | 6% |
| TRP_66 | 8 | Tb927.3.3560/Tb03.25B21.20 | 107 | 2 peptides | 1 peptides | 3% |
| TRP_66 | 9 | Tb927.1.2340 | 95 | 3 peptides | 1 peptides | 8% |
| TRP_66 | 10 | Tb11.01.8510 | 94 | 2 peptides | 2 peptides | 5% |
| TRP_66 | 11 | Tb11.02.5450 | 81 | 3 peptides | 1 peptides | 4% |
| TRP_66 | 12 | Tb10.389.0720 | 56 | 2 peptides | 1 peptides | 1% |
| TRP_66 | 13 | Tb10.406.0650 | 52 | 3 peptides | 2 peptides | 1% |
| TRP_66 | 17 | Tb10.70.1190 | 38 | 2 peptides | 1 peptides | 5% |
| TRP_66 | 26 | Tb11.02.0070 | 26 | 2 peptides | 1 peptides | 2% |
| TRP_66 | 27 | Tb10.61.2850 | 26 | 1 peptides | 1 peptides | 2% |
| TRP_66 | 52 | Tb927.4.1850/Tb04.29M18.150 | 20 | 1 peptides | 1 peptides | 1% |
| TRP_67 | 1 | Tb10.70.1190 | 1014 | 25 peptides | 15 peptides | 21% |
| TRP_67 | 2 | Tb10.389.0880 | 136 | 4 peptides | 3 peptides | 4% |
| TRP_67 | 3 | Tb10.26.1080 | 112 | 5 peptides | 2 peptides | 7% |
| TRP_67 | 5 | Tb927.3.1210/Tb03.1J15.730 | 34 | 1 peptides | 1 peptides | 0% |
| TRP_68 | 1 | Tb11.02.1120 | 689 | 14 peptides | 11 peptides | 21% |
| TRP_68 | 2 | Tb927.4.1080/Tb04.5E12.370 | 277 | 4 peptides | 4 peptides | 8% |
| TRP_68 | 3 | Tb927.5.2940/Tb05.26K5.210 | 240 | 5 peptides | 4 peptides | 8% |
| TRP_68 | 5 | Tb10.406.0560 | 35 | 1 peptides | 1 peptides | 0% |
| TRP_070330_97 | 1 | Tb11.01.8520 | 504 | 12 peptides | 7 peptides | 28% |
| TRP_070330_97 | 2 | Tb927.6.1570/Tb06.28P18.310 | 117 | 2 peptides | 2 peptides | 6% |
| TRP_070330_97 | 3 | Tb927.4.2030/Tb04.29M18.690 | 98 | 2 peptides | 1 peptides | 8% |
| TRP_070330_99 | 1 | Tb927.4.3590/Tb04.26G5.430 | 593 | 13 peptides | 10 peptides | 54% |
| TRP_070330_99 | 3 | Tb10.70.6610 | 118 | 3 peptides | 1 peptides | 10% |
| TRP_070330_99 | 4 | Tb927.4.2030/Tb04.29M18.690 | 99 | 2 peptides | 2 peptides | 8% |

| | | | | | | |
|---|---|---|---|---|---|---|
| TRP_070330_99 | 5 | Tb927.5.1700/Tb05.1P6.900 | 69 | 1 peptides | 1 peptides | 4% |
| TRP_070330_99 | 6 | Tb11.46.0001 | 61 | 1 peptides | 1 peptides | 3% |
| TRP_070330_99 | 11 | Tb10.70.2770 | 32 | 1 peptides | 1 peptides | 3% |
| TRP_070330_100 | 1 | Tb10.70.1100 | 252 | 5 peptides | 4 peptides | 34% |
| TRP_070330_100 | 2 | Tb927.7.570/Tb07.8P12.860 | 218 | 5 peptides | 5 peptides | 26% |
| TRP_070330_100 | 3 | Tb11.01.3110 | 173 | 3 peptides | 2 peptides | 5% |
| TRP_070330_100 | 4 | Tb10.70.6540 | 162 | 4 peptides | 2 peptides | 16% |
| TRP_070330_100 | 5 | Tb927.6.2170/Tb06.4M18.480 | 76 | 3 peptides | 1 peptides | 12% |
| TRP_070330_100 | 6 | Tb927.8.5880/Tb08.11J15.1060 | 74 | 2 peptides | 1 peptides | 13% |
| TRP_070330_100 | 7 | Tb927.7.3440/Tb07.28B13.630 | 67 | 1 peptides | 1 peptides | 3% |
| TRP_070330_100 | 8 | Tb11.01.6670 | 42 | 1 peptides | 1 peptides | 5% |
| TRP_070330_101 | 1 | Tb11.01.3110 | 66 | 2 peptides | 1 peptides | 3% |
| TRP_070330_101 | 2 | Tb11.01.7550 | 49 | 1 peptides | 1 peptides | 7% |
| TRP_070330_102 | 1 | Tb09.211.4460 | 332 | 5 peptides | 4 peptides | 28% |
| TRP_070330_102 | 2 | Tb11.03.0250 | 101 | 1 peptides | 1 peptides | 6% |
| TRP_070330_102 | 3 | Tb09.211.0740 | 84 | 2 peptides | 1 peptides | 10% |
| TRP_070330_102 | 5 | Tb927.3.3450/Tb03.25B21.123 | 55 | 2 peptides | 1 peptides | 15% |
| TRP_070330_102 | 6 | Tb927.6.2200/Tb06.4M18.560 | 55 | 1 peptides | 1 peptides | 5% |
| TRP_070330_102 | 7 | Tb10.61.1750 | 48 | 2 peptides | 1 peptides | 2% |
| TRP_070330_102 | 8 | Tb927.7.1130/Tb07.27M11.280 | 45 | 2 peptides | 1 peptides | 11% |
| TRP_070330_103 | 1 | Tb11.01.7800 | 134 | 5 peptides | 2 peptides | 28% |
| TRP_070330_103 | 3 | Tb11.02.0815 | 82 | 2 peptides | 1 peptides | 13% |
| TRP_070330_103 | 4 | Tb11.46.0001 | 65 | 1 peptides | 1 peptides | 3% |
| TRP_070330_104 | 1 | Tb927.3.5180/Tb03.5L5.210 | 204 | 5 peptides | 4 peptides | 26% |
| TRP_070330_104 | 3 | Tb10.70.0830 | 28 | 1 peptides | 1 peptides | 0% |
| TRP_070330_104 | 9 | Tb11.01.1100 | 20 | 1 peptides | 1 peptides | 1% |
| TRP_070330_105 | 1 | Tb927.7.1320/Tb07.27M11.580 | 236 | 6 peptides | 4 peptides | 50% |
| TRP_070330_105 | 2 | Tb09.160.0465 | 121 | 2 peptides | 1 peptides | 27% |
| TRP_070330_106 | 1 | Tb11.02.1070 | 2400 | 66 peptides | 39 peptides | 56% |
| TRP_070330_106 | 2 | Tb10.389.0880 | 813 | 15 peptides | 10 peptides | 20% |
| TRP_070330_106 | 3 | Tb10.70.2650 | 588 | 14 peptides | 8 peptides | 15% |
| TRP_070330_106 | 4 | Tb927.2.100/3B10.5 | 198 | 5 peptides | 3 peptides | 5% |
| TRP_070330_106 | 6 | Tb927.2.280/3B10.95 | 83 | 1 peptides | 1 peptides | 1% |
| TRP_070330_106 | 9 | Tb927.1.2340 | 36 | 1 peptides | 1 peptides | 3% |
| TRP_070330_106 | 18 | Tb10.05.0080 | 27 | 1 peptides | 1 peptides | 1% |
| TRP_070330_107 | 1 | Tb10.389.0880 | 1718 | 38 peptides | 27 peptides | 39% |

| | | | | | | |
|---|---|---|---|---|---|---|
| TRP_070330_107 | 2 | Tb10.70.2650 | 873 | 22 peptides | 11 peptides | 23% |
| TRP_070330_107 | 3 | Tb927.1.180 | 540 | 14 peptides | 8 peptides | 14% |
| TRP_070330_107 | 4 | Tb927.2.100/3B10.5 | 264 | 6 peptides | 5 peptides | 6% |
| TRP_070330_107 | 5 | Tb10.26.1080 | 185 | 6 peptides | 2 peptides | 7% |
| TRP_070330_107 | 6 | Tb927.6.1290/Tb06.3A7.840 | 169 | 5 peptides | 2 peptides | 4% |
| TRP_070330_107 | 7 | Tb11.02.1070 | 157 | 4 peptides | 3 peptides | 5% |
| TRP_070330_107 | 8 | Tb927.2.470/3B10.190 | 126 | 3 peptides | 1 peptides | 3% |
| TRP_070330_107 | 9 | Tb927.2.5980/1F7.360 | 91 | 3 peptides | 2 peptides | 3% |
| TRP_070330_107 | 11 | Tb927.2.1180/25N24.100 | 43 | 1 peptides | 1 peptides | 1% |
| TRP_070330_108 | 1 | Tb10.70.2650 | 1451 | 31 peptides | 20 peptides | 33% |
| TRP_070330_108 | 2 | Tb927.1.180 | 567 | 14 peptides | 8 peptides | 17% |
| TRP_070330_108 | 4 | Tb927.7.5210/Tb07.27E10.470 | 250 | 6 peptides | 4 peptides | 7% |
| TRP_070330_108 | 5 | Tb10.389.0880 | 187 | 4 peptides | 3 peptides | 5% |
| TRP_070330_108 | 6 | Tb10.70.4740 | 172 | 3 peptides | 2 peptides | 6% |
| TRP_070330_108 | 7 | Tb11.01.3110 | 146 | 3 peptides | 2 peptides | 5% |
| TRP_070330_108 | 8 | Tb927.3.4750/Tb03.48K5.320 | 138 | 4 peptides | 2 peptides | 4% |
| TRP_070330_108 | 9 | Tb11.02.1070 | 81 | 2 peptides | 2 peptides | 2% |
| TRP_070330_108 | 10 | Tb927.2.280/3B10.95 | 80 | 3 peptides | 1 peptides | 3% |
| TRP_070330_108 | 11 | Tb927.2.340/3B10.125 | 68 | 2 peptides | 1 peptides | 2% |
| TRP_070330_108 | 19 | Tb10.26.1080 | 27 | 2 peptides | 1 peptides | 2% |
| TRP_070330_109 | 1 | Tb11.01.7010 | 1128 | 26 peptides | 17 peptides | 24% |
| TRP_070330_109 | 2 | Tb10.70.4740 | 570 | 8 peptides | 5 peptides | 21% |
| TRP_070330_109 | 3 | Tb10.70.2650 | 510 | 12 peptides | 8 peptides | 13% |
| TRP_070330_109 | 4 | Tb927.1.2340 | 232 | 4 peptides | 3 peptides | 11% |
| TRP_070330_109 | 5 | Tb11.01.3110 | 137 | 3 peptides | 1 peptides | 4% |
| TRP_070330_109 | 6 | Tb927.7.5210/Tb07.27E10.470 | 94 | 3 peptides | 1 peptides | 3% |
| TRP_070330_109 | 7 | Tb927.6.700/Tb06.3D8.610 | 94 | 3 peptides | 1 peptides | 3% |
| TRP_070330_109 | 8 | Tb09.160.5550 | 78 | 1 peptides | 1 peptides | 1% |
| TRP_070330_109 | 10 | Tb927.8.7020/Tb08.10K10.800 | 54 | 1 peptides | 1 peptides | 1% |
| TRP_070330_109 | 16 | Tb927.3.4750/Tb03.48K5.320 | 34 | 1 peptides | 1 peptides | 1% |
| TRP_070330_110 | 1 | Tb11.02.1210 | 786 | 19 peptides | 12 peptides | 17% |
| TRP_070330_110 | 2 | Tb10.6k15.1220 | 660 | 17 peptides | 10 peptides | 14% |
| TRP_070330_110 | 3 | Tb10.389.0880 | 360 | 10 peptides | 6 peptides | 12% |
| TRP_070330_110 | 4 | Tb11.01.7010 | 314 | 11 peptides | 4 peptides | 8% |
| TRP_070330_110 | 5 | Tb927.6.4480/Tb06.26G9.740 | 181 | 5 peptides | 2 peptides | 5% |
| TRP_070330_110 | 6 | Tb927.8.2640/Tb08.26A17.680 | 178 | 5 peptides | 2 peptides | 3% |

| | | | | | | |
|---|---|---|---|---|---|---|
| TRP_070330_110 | 7 | Tb10.70.2650 | 137 | 4 peptides | 2 peptides | 4% |
| TRP_070330_110 | 8 | Tb927.1.2340 | 131 | 2 peptides | 2 peptides | 5% |
| TRP_070330_110 | 9 | Tb11.01.3110 | 55 | 4 peptides | 1 peptides | 5% |
| TRP_070330_110 | 57 | Tb927.8.7020/Tb08.10K10.800 | 16 | 1 peptides | 1 peptides | 1% |
| TRP_070330_111 | 1 | Tb10.26.1080 | 1509 | 38 peptides | 21 peptides | 37% |
| TRP_070330_111 | 2 | Tb10.389.0880 | 822 | 15 peptides | 10 peptides | 22% |
| TRP_070330_111 | 3 | Tb11.01.3110 | 327 | 7 peptides | 4 peptides | 12% |
| TRP_070330_111 | 4 | Tb10.70.6470 | 224 | 4 peptides | 4 peptides | 6% |
| TRP_070330_111 | 5 | Tb10.6k15.2290 | 139 | 4 peptides | 2 peptides | 7% |
| TRP_070330_111 | 9 | Tb927.8.3680/Tb08.10J17.750 | 36 | 1 peptides | 1 peptides | 1% |
| TRP_070330_111 | 14 | Tb11.52.0003 | 27 | 1 peptides | 1 peptides | 1% |
| TRP_070330_112 | 1 | Tb11.02.5450 | 1292 | 25 peptides | 19 peptides | 41% |
| TRP_070330_112 | 2 | Tb927.2.4370/30M24.395 | 1223 | 27 peptides | 23 peptides | 40% |
| TRP_070330_112 | 3 | Tb11.01.3110 | 1162 | 27 peptides | 18 peptides | 33% |
| TRP_070330_112 | 4 | Tb927.7.2100/Tb07.43M14.550 | 592 | 9 peptides | 8 peptides | 15% |
| TRP_070330_112 | 5 | Tb927.6.3740/Tb06.4F7.750 | 265 | 6 peptides | 4 peptides | 10% |
| TRP_070330_112 | 6 | Tb927.3.3560/Tb03.25B21.20 | 254 | 6 peptides | 4 peptides | 10% |
| TRP_070330_112 | 8 | Tb927.6.4590/Tb06.26G9.450 | 167 | 6 peptides | 2 peptides | 13% |
| TRP_070330_112 | 10 | Tb11.01.1400 | 37 | 1 peptides | 1 peptides | 2% |
| TRP_070330_112 | 36 | Tb927.7.4710/Tb07.26A24.460 | 21 | 1 peptides | 1 peptides | 3% |
| TRP_070330_113 | 1 | Tb10.26.1080 | 1407 | 40 peptides | 22 peptides | 39% |
| TRP_070330_113 | 2 | Tb11.01.3110 | 399 | 6 peptides | 5 peptides | 10% |
| TRP_070330_113 | 3 | Tb927.3.4840/Tb03.48K5.520 | 135 | 4 peptides | 2 peptides | 6% |
| TRP_070330_113 | 9 | Tb927.8.3680/Tb08.10J17.750 | 28 | 2 peptides | 1 peptides | 1% |
| TRP_070330_114 | 1 | Tb11.01.3110 | 1440 | 35 peptides | 23 peptides | 37% |
| TRP_070330_114 | 2 | Tb10.6k15.2520 | 517 | 14 peptides | 9 peptides | 17% |
| TRP_070330_114 | 3 | Tb11.46.0008 | 390 | 11 peptides | 8 peptides | 16% |
| TRP_070330_114 | 4 | Tb11.02.5450 | 311 | 7 peptides | 5 peptides | 14% |
| TRP_070330_114 | 5 | Tb927.7.3620/Tb07.28B13.380 | 187 | 3 peptides | 2 peptides | 5% |
| TRP_070330_114 | 6 | Tb927.7.190/Tb07.8P12.250 | 186 | 4 peptides | 4 peptides | 7% |
| TRP_070330_114 | 7 | Tb10.6k15.2290 | 153 | 6 peptides | 2 peptides | 11% |
| TRP_070330_114 | 8 | Tb927.3.3560/Tb03.25B21.20 | 85 | 2 peptides | 2 peptides | 4% |
| TRP_070330_114 | 10 | Tb927.1.2340 | 34 | 1 peptides | 1 peptides | 2% |
| TRP_070330_115 | 1 | Tb11.01.3110 | 914 | 18 peptides | 13 peptides | 27% |
| TRP_070330_115 | 2 | Tb10.70.0280 | 908 | 19 peptides | 12 peptides | 30% |
| TRP_070330_115 | 3 | Tb927.5.2940/Tb05.26K5.210 | 583 | 16 peptides | 7 peptides | 30% |

| | | | | | | |
|---|---|---|---|---|---|---|
| TRP_070330_115 | 4 | Tb927.3.3560/Tb03.25B21.20 | 306 | 8 peptides | 3 peptides | 13% |
| TRP_070330_115 | 5 | Tb10.6k15.2620 | 280 | 7 peptides | 5 peptides | 13% |
| TRP_070330_115 | 6 | Tb10.6k15.2290 | 239 | 5 peptides | 3 peptides | 10% |
| TRP_070330_115 | 7 | Tb927.4.1080/Tb04.5E12.370 | 207 | 5 peptides | 2 peptides | 8% |
| TRP_070330_115 | 8 | Tb11.02.5450 | 185 | 4 peptides | 3 peptides | 6% |
| TRP_070330_115 | 9 | Tb927.6.4590/Tb06.26G9.450 | 179 | 4 peptides | 3 peptides | 6% |
| TRP_070330_115 | 10 | Tb927.8.980/Tb08.25L8.80 | 137 | 3 peptides | 2 peptides | 6% |
| TRP_070330_115 | 11 | Tb10.26.1080 | 118 | 3 peptides | 1 peptides | 4% |
| TRP_070330_115 | 12 | Tb10.61.1750 | 68 | 1 peptides | 1 peptides | 1% |
| TRP_070330_115 | 14 | Tb09.160.5530 | 61 | 1 peptides | 1 peptides | 5% |
| TRP_070330_115 | 15 | Tb927.1.2340 | 35 | 1 peptides | 1 peptides | 3% |
| TRP_070330_115 | 18 | Tb10.70.0830 | 28 | 1 peptides | 1 peptides | 0% |
| TRP_070330_115 | 22 | Tb927.7.1310/Tb07.27M11.570 | 27 | 1 peptides | 1 peptides | 0% |
| TRP_070330_115 | 23 | Tb927.5.3830/Tb05.6E7.780 | 25 | 1 peptides | 1 peptides | 3% |
| TRP_070330_115 | 33 | Tb10.70.3070 | 22 | 1 peptides | 1 peptides | 3% |
| TRP_070330_116 | 1 | Tb10.6k15.2620 | 919 | 24 peptides | 14 peptides | 32% |
| TRP_070330_116 | 2 | Tb10.70.0280 | 704 | 14 peptides | 11 peptides | 25% |
| TRP_070330_116 | 3 | Tb11.01.3110 | 497 | 8 peptides | 5 peptides | 13% |
| TRP_070330_116 | 4 | Tb11.01.8510 | 124 | 3 peptides | 2 peptides | 7% |
| TRP_070330_116 | 5 | Tb927.8.980/Tb08.25L8.80 | 73 | 1 peptides | 1 peptides | 1% |
| TRP_070330_116 | 12 | Tb927.5.2940/Tb05.26K5.210 | 37 | 2 peptides | 1 peptides | 6% |
| TRP_070330_117 | 1 | Tb11.02.0100 | 1057 | 25 peptides | 19 peptides | 43% |
| TRP_070330_117 | 2 | Tb10.70.1130 | 566 | 9 peptides | 7 peptides | 22% |
| TRP_070330_117 | 3 | Tb11.02.2210 | 527 | 12 peptides | 6 peptides | 24% |
| TRP_070330_117 | 4 | Tb927.1.3950 | 426 | 8 peptides | 7 peptides | 13% |
| TRP_070330_117 | 5 | Tb10.70.4740 | 412 | 6 peptides | 4 peptides | 14% |
| TRP_070330_117 | 6 | Tb11.42.0003 | 301 | 6 peptides | 3 peptides | 13% |
| TRP_070330_117 | 7 | Tb927.7.2240/Tb07.22O10.180 | 209 | 5 peptides | 4 peptides | 10% |
| TRP_070330_117 | 8 | Tb927.1.2340 | 163 | 3 peptides | 2 peptides | 7% |
| TRP_070330_117 | 9 | Tb10.61.2680 | 129 | 4 peptides | 1 peptides | 10% |
| TRP_070330_117 | 10 | Tb11.02.5020 | 105 | 4 peptides | 2 peptides | 7% |
| TRP_070330_117 | 12 | Tb11.01.3110 | 61 | 3 peptides | 1 peptides | 5% |
| TRP_070330_117 | 13 | Tb11.01.8510 | 49 | 1 peptides | 1 peptides | 1% |
| TRP_070330_117 | 31 | Tb11.01.4830 | 28 | 1 peptides | 1 peptides | 2% |
| TRP_070330_117 | 41 | Tb10.70.5060 | 24 | 1 peptides | 1 peptides | 3% |
| TRP_070330_117 | 53 | Tb927.7.970/Tb07.29K4.160 | 22 | 1 peptides | 1 peptides | 1% |

| | | | | | | |
|---|---|---|---|---|---|---|
| TRP_070330_118 | 1 | Tb10.389.1480 | 524 | 11 peptides | 8 peptides | 24% |
| TRP_070330_118 | 2 | Tb10.70.1130 | 518 | 10 peptides | 8 peptides | 22% |
| TRP_070330_118 | 3 | Tb927.1.3950 | 395 | 6 peptides | 5 peptides | 11% |
| TRP_070330_118 | 4 | Tb09.211.3180 | 352 | 7 peptides | 7 peptides | 14% |
| TRP_070330_118 | 5 | Tb10.61.0180 | 327 | 10 peptides | 5 peptides | 25% |
| TRP_070330_118 | 6 | Tb927.1.2340 | 276 | 8 peptides | 3 peptides | 17% |
| TRP_070330_118 | 7 | Tb10.70.4740 | 244 | 5 peptides | 3 peptides | 15% |
| TRP_070330_118 | 8 | Tb11.01.0870 | 203 | 4 peptides | 3 peptides | 10% |
| TRP_070330_118 | 9 | Tb10.406.0520 | 152 | 3 peptides | 2 peptides | 8% |
| TRP_070330_118 | 10 | Tb927.1.2330 | 102 | 2 peptides | 2 peptides | 4% |
| TRP_070330_118 | 11 | Tb11.01.1350 | 96 | 2 peptides | 2 peptides | 3% |
| TRP_070330_118 | 13 | Tb10.70.7730 | 55 | 1 peptides | 1 peptides | 2% |
| TRP_070330_118 | 14 | Tb10.406.0650 | 53 | 2 peptides | 1 peptides | 1% |
| TRP_070330_119 | 1 | Tb10.70.1130 | 955 | 38 peptides | 15 peptides | 40% |
| TRP_070330_119 | 2 | Tb10.70.4740 | 229 | 5 peptides | 3 peptides | 14% |
| TRP_070330_119 | 3 | Tb927.1.2340 | 219 | 4 peptides | 2 peptides | 10% |
| TRP_070330_119 | 4 | Tb927.7.2240/Tb07.22O10.180 | 124 | 3 peptides | 3 peptides | 6% |
| TRP_070330_119 | 5 | Tb09.211.4330 | 116 | 2 peptides | 1 peptides | 4% |
| TRP_070330_119 | 6 | Tb10.61.2680 | 53 | 2 peptides | 1 peptides | 5% |
| TRP_070330_119 | 8 | Tb927.1.3950 | 41 | 3 peptides | 1 peptides | 7% |
| TRP_070330_119 | 14 | Tb10.70.7730 | 35 | 1 peptides | 1 peptides | 2% |
| TRP_070330_119 | 17 | Tb09.160.0810 | 29 | 1 peptides | 1 peptides | 3% |
| TRP_070330_119 | 50 | Tb10.70.5650 | 21 | 1 peptides | 1 peptides | 2% |
| TRP_070330_120 | 1 | Tb10.70.0280 | 1097 | 19 peptides | 15 peptides | 37% |
| TRP_070330_120 | 2 | Tb10.6k15.2620 | 313 | 10 peptides | 5 peptides | 19% |
| TRP_070330_120 | 3 | Tb10.70.4740 | 261 | 6 peptides | 4 peptides | 16% |
| TRP_070330_120 | 4 | Tb10.70.1130 | 153 | 3 peptides | 2 peptides | 9% |
| TRP_070330_120 | 5 | Tb10.61.2680 | 149 | 4 peptides | 2 peptides | 11% |
| TRP_070330_120 | 6 | Tb927.1.2340 | 117 | 3 peptides | 2 peptides | 7% |
| TRP_070330_120 | 7 | Tb10.6k15.2330 | 111 | 5 peptides | 2 peptides | 9% |
| TRP_070330_120 | 9 | Tb927.2.470/3B10.190 | 59 | 2 peptides | 1 peptides | 2% |
| TRP_070330_120 | 10 | Tb11.01.8510 | 52 | 1 peptides | 1 peptides | 1% |
| TRP_070330_120 | 11 | Tb11.42.0003 | 51 | 4 peptides | 1 peptides | 9% |
| TRP_070330_120 | 12 | Tb11.01.3110 | 50 | 1 peptides | 1 peptides | 1% |
| TRP_070330_120 | 13 | Tb11.01.8770 | 48 | 2 peptides | 1 peptides | 2% |
| TRP_070330_120 | 14 | Tb10.26.1080 | 41 | 1 peptides | 1 peptides | 1% |

| | | | | | | |
|---|---|---|---|---|---|---|
| TRP_070330_120 | 74 | Tb927.7.970/Tb07.29K4.160 | 17 | 1 peptides | 1 peptides | 1% |
| TRP_070330_121 | 1 | Tb10.70.4740 | 1325 | 29 peptides | 21 peptides | 53% |
| TRP_070330_121 | 2 | Tb927.1.700 | 163 | 3 peptides | 2 peptides | 9% |
| TRP_070330_121 | 3 | Tb11.01.4660 | 154 | 3 peptides | 3 peptides | 8% |
| TRP_070330_121 | 4 | Tb10.70.1130 | 114 | 2 peptides | 1 peptides | 7% |
| TRP_070330_121 | 5 | Tb10.389.1730 | 113 | 3 peptides | 2 peptides | 7% |
| TRP_070330_121 | 6 | Tb11.01.3110 | 79 | 3 peptides | 1 peptides | 4% |
| TRP_070330_121 | 33 | Tb927.8.2030/Tb08.26N11.500 | 24 | 2 peptides | 1 peptides | 4% |
| TRP_070330_121 | 35 | Tb927.1.2340 | 24 | 3 peptides | 1 peptides | 6% |
| TRP_070330_122 | 1 | Tb927.2.4590/30M24.285 | 284 | 10 peptides | 4 peptides | 17% |
| TRP_070330_122 | 2 | Tb927.7.1110/Tb07.27M11.260 | 248 | 9 peptides | 3 peptides | 22% |
| TRP_070330_122 | 3 | Tb11.46.0001 | 232 | 4 peptides | 2 peptides | 12% |
| TRP_070330_122 | 4 | Tb927.8.4430/Tb08.29H22.830 | 163 | 6 peptides | 2 peptides | 18% |
| TRP_070330_122 | 5 | Tb10.70.4740 | 152 | 2 peptides | 2 peptides | 8% |
| TRP_070330_122 | 6 | Tb927.7.4570/Tb07.26A24.720 | 82 | 3 peptides | 1 peptides | 6% |
| TRP_070330_122 | 8 | Tb927.4.1300/Tb04.2L9.440 | 46 | 1 peptides | 1 peptides | 2% |
| TRP_070330_122 | 29 | Tb11.55.0024 | 22 | 1 peptides | 1 peptides | 3% |
| TRP_070330_124 | 1 | Tb927.5.1460/Tb05.30H13.580 | 410 | 11 peptides | 5 peptides | 24% |
| TRP_070330_124 | 2 | Tb10.6k15.1160 | 277 | 7 peptides | 4 peptides | 22% |
| TRP_070330_124 | 3 | Tb11.01.3040 | 251 | 5 peptides | 3 peptides | 13% |
| TRP_070330_124 | 4 | Tb927.7.4290/Tb07.5F10.150 | 211 | 4 peptides | 2 peptides | 11% |
| TRP_070330_124 | 5 | Tb927.8.4430/Tb08.29H22.830 | 170 | 6 peptides | 4 peptides | 19% |
| TRP_070330_124 | 6 | Tb11.01.3170 | 164 | 4 peptides | 2 peptides | 10% |
| TRP_070330_124 | 7 | Tb927.7.4570/Tb07.26A24.720 | 163 | 4 peptides | 3 peptides | 10% |
| TRP_070330_124 | 8 | Tb09.160.3710 | 150 | 4 peptides | 2 peptides | 14% |
| TRP_070330_124 | 9 | Tb927.6.2790/Tb06.5F5.290 | 145 | 4 peptides | 2 peptides | 10% |
| TRP_070330_124 | 10 | Tb927.3.2960/Tb03.27C5.420 | 139 | 3 peptides | 2 peptides | 8% |
| TRP_070330_124 | 11 | Tb09.244.2730 | 110 | 4 peptides | 1 peptides | 9% |
| TRP_070330_124 | 12 | Tb10.70.1130 | 57 | 1 peptides | 1 peptides | 2% |
| TRP_070330_124 | 29 | Tb927.4.1300/Tb04.2L9.440 | 26 | 1 peptides | 1 peptides | 2% |
| TRP_070330_124 | 101 | NOT ASSIGNED | | | 4 peptides | |
| TRP_070330_125 | 1 | Tb09.160.3710 | 437 | 7 peptides | 5 peptides | 24% |
| TRP_070330_125 | 2 | Tb11.01.3040 | 389 | 9 peptides | 5 peptides | 21% |
| TRP_070330_125 | 3 | Tb927.5.1460/Tb05.30H13.580 | 322 | 8 peptides | 6 peptides | 28% |
| TRP_070330_125 | 4 | Tb927.3.2960/Tb03.27C5.420 | 210 | 5 peptides | 1 peptides | 17% |
| TRP_070330_125 | 5 | Tb11.01.3170 | 188 | 5 peptides | 1 peptides | 12% |

| | | | | | | |
|---|---|---|---|---|---|---|
| TRP_070330_125 | 6 | Tb09.244.2730 | 154 | 5 peptides | 3 peptides | 12% |
| TRP_070330_125 | 7 | Tb927.7.4290/Tb07.5F10.150 | 95 | 2 peptides | 2 peptides | 6% |
| TRP_070330_125 | 8 | Tb10.70.1130 | 88 | 2 peptides | 2 peptides | 4% |
| TRP_070330_125 | 9 | Tb927.6.2790/Tb06.5F5.290 | 77 | 2 peptides | 2 peptides | 6% |
| TRP_070330_125 | 10 | Tb10.6k15.1160 | 77 | 2 peptides | 1 peptides | 6% |
| TRP_070330_125 | 11 | Tb09.211.0560 | 67 | 2 peptides | 2 peptides | 5% |
| TRP_070330_125 | 12 | Tb09.160.5200 | 49 | 1 peptides | 1 peptides | 3% |
| TRP_070330_126 | 1 | Tb11.01.3040 | 659 | 14 peptides | 10 peptides | 33% |
| TRP_070330_126 | 2 | Tb927.3.2960/Tb03.27C5.420 | 341 | 10 peptides | 5 peptides | 24% |
| TRP_070330_126 | 3 | Tb09.244.2730 | 154 | 4 peptides | 2 peptides | 9% |
| TRP_070330_126 | 4 | Tb11.01.3170 | 110 | 4 peptides | 1 peptides | 14% |
| TRP_070330_126 | 5 | Tb927.6.2790/Tb06.5F5.290 | 100 | 3 peptides | 1 peptides | 8% |
| TRP_070330_126 | 6 | Tb10.70.2160 | 65 | 2 peptides | 1 peptides | 8% |
| TRP_070330_126 | 23 | Tb11.46.0001 | 24 | 1 peptides | 1 peptides | 3% |
| TRP_070330_126 | 65 | Tb927.8.5600/Tb08.26E13.220 | 17 | 1 peptides | 1 peptides | 2% |
| TRP_070330_127 | 1 | Tb10.70.4740 | 1170 | 22 peptides | 17 peptides | 49% |
| TRP_070330_127 | 2 | Tb927.8.7410/Tb08.10K10.160 | 965 | 24 peptides | 14 peptides | 41% |
| TRP_070330_127 | 3 | Tb10.70.1130 | 461 | 9 peptides | 7 peptides | 22% |
| TRP_070330_127 | 4 | Tb927.1.2340 | 371 | 7 peptides | 3 peptides | 16% |
| TRP_070330_127 | 5 | Tb10.70.7730 | 335 | 8 peptides | 5 peptides | 17% |
| TRP_070330_127 | 6 | Tb11.01.1350 | 157 | 5 peptides | 3 peptides | 10% |
| TRP_070330_127 | 7 | Tb11.01.3110 | 122 | 4 peptides | 1 peptides | 6% |
| TRP_070330_127 | 8 | Tb10.61.0180 | 71 | 2 peptides | 1 peptides | 4% |
| TRP_070330_127 | 9 | Tb10.389.1730 | 70 | 1 peptides | 1 peptides | 2% |
| TRP_070330_127 | 10 | Tb927.6.400/Tb06.28F21.350 | 69 | 3 peptides | 2 peptides | 6% |
| TRP_070330_128 | 1 | Tb10.70.4740 | 890 | 17 peptides | 12 peptides | 36% |
| TRP_070330_128 | 2 | Tb927.4.5010/Tb04.3M17.390 | 797 | 26 peptides | 14 peptides | 43% |
| TRP_070330_128 | 3 | Tb927.1.2340 | 498 | 11 peptides | 6 peptides | 21% |
| TRP_070330_128 | 4 | Tb11.01.1350 | 357 | 10 peptides | 5 peptides | 17% |
| TRP_070330_128 | 5 | Tb10.61.0180 | 341 | 11 peptides | 3 peptides | 25% |
| TRP_070330_128 | 6 | Tb927.1.2330 | 303 | 7 peptides | 5 peptides | 19% |
| TRP_070330_128 | 7 | Tb10.70.7730 | 268 | 5 peptides | 3 peptides | 11% |
| TRP_070330_128 | 8 | Tb10.70.0280 | 251 | 7 peptides | 5 peptides | 14% |
| TRP_070330_128 | 9 | Tb11.01.3110 | 157 | 4 peptides | 2 peptides | 9% |
| TRP_070330_128 | 10 | Tb927.3.5320/Tb03.5L5.500 | 133 | 5 peptides | 3 peptides | 10% |
| TRP_070330_128 | 11 | Tb927.6.400/Tb06.28F21.350 | 123 | 3 peptides | 3 peptides | 6% |

| | | | | | | |
|---|---|---|---|---|---|---|
| TRP_070330_128 | 14 | Tb10.70.1130 | 55 | 2 peptides | 1 peptides | 4% |
| TRP_070330_128 | 45 | Tb10.05.0110 | 22 | 2 peptides | 1 peptides | 4% |
| TRP_070330_129 | 1 | Tb10.70.4740 | 1294 | 30 peptides | 24 peptides | 52% |
| TRP_070330_129 | 2 | Tb11.01.4660 | 424 | 11 peptides | 7 peptides | 28% |
| TRP_070330_129 | 3 | Tb10.389.1730 | 395 | 9 peptides | 5 peptides | 17% |
| TRP_070330_129 | 4 | Tb09.160.4570 | 281 | 11 peptides | 3 peptides | 22% |
| TRP_070330_129 | 5 | Tb09.160.4560 | 269 | 10 peptides | 3 peptides | 17% |
| TRP_070330_129 | 6 | Tb11.01.3110 | 243 | 5 peptides | 4 peptides | 8% |
| TRP_070330_129 | 8 | Tb927.4.5010/Tb04.3M17.390 | 157 | 6 peptides | 2 peptides | 12% |
| TRP_070330_129 | 9 | Tb09.160.3270 | 138 | 2 peptides | 2 peptides | 5% |
| TRP_070330_129 | 10 | Tb927.8.1710/Tb08.29O9.490 | 93 | 4 peptides | 1 peptides | 8% |
| TRP_070330_129 | 11 | Tb11.02.5450 | 85 | 2 peptides | 1 peptides | 5% |
| TRP_070330_129 | 12 | Tb927.1.2340 | 67 | 2 peptides | 1 peptides | 6% |
| TRP_070330_129 | 13 | Tb10.70.7730 | 62 | 1 peptides | 1 peptides | 2% |
| TRP_070330_129 | 16 | Tb927.1.2330 | 47 | 1 peptides | 1 peptides | 2% |
| TRP_070330_129 | 52 | Tb927.8.2030/Tb08.26N11.500 | 27 | 2 peptides | 1 peptides | 3% |
| TRP_070330_130 | 1 | Tb10.70.4740 | 1209 | 27 peptides | 16 peptides | 42% |
| TRP_070330_130 | 2 | Tb11.01.3110 | 662 | 11 peptides | 9 peptides | 17% |
| TRP_070330_130 | 4 | Tb10.389.1730 | 296 | 5 peptides | 5 peptides | 13% |
| TRP_070330_130 | 5 | Tb09.160.4560 | 268 | 9 peptides | 2 peptides | 15% |
| TRP_070330_130 | 6 | Tb927.4.5010/Tb04.3M17.390 | 204 | 8 peptides | 4 peptides | 16% |
| TRP_070330_130 | 7 | Tb11.01.4660 | 119 | 2 peptides | 2 peptides | 5% |
| TRP_070330_130 | 8 | Tb11.01.1350 | 85 | 3 peptides | 1 peptides | 8% |
| TRP_070330_130 | 9 | Tb09.160.3270 | 77 | 2 peptides | 2 peptides | 4% |
| TRP_070330_130 | 10 | Tb927.1.2340 | 77 | 3 peptides | 1 peptides | 7% |
| TRP_070330_130 | 11 | Tb10.70.7730 | 76 | 1 peptides | 1 peptides | 2% |
| TRP_070330_131 | 1 | Tb10.70.3710 | 659 | 16 peptides | 9 peptides | 32% |
| TRP_070330_131 | 2 | Tb10.70.4740 | 456 | 8 peptides | 5 peptides | 23% |
| TRP_070330_131 | 3 | Tb11.01.3110 | 386 | 7 peptides | 6 peptides | 12% |
| TRP_070330_131 | 4 | Tb11.01.0320 | 242 | 7 peptides | 3 peptides | 14% |
| TRP_070330_131 | 5 | Tb10.6k15.3250 | 213 | 4 peptides | 4 peptides | 7% |
| TRP_070330_131 | 6 | Tb09.160.4570 | 212 | 7 peptides | 2 peptides | 11% |
| TRP_070330_131 | 7 | Tb09.160.4590 | 203 | 7 peptides | 2 peptides | 12% |
| TRP_070330_131 | 8 | Tb927.8.1990/Tb08.26N11.460 | 162 | 6 peptides | 2 peptides | 20% |
| TRP_070330_131 | 9 | Tb10.70.5360 | 155 | 4 peptides | 3 peptides | 11% |
| TRP_070330_131 | 10 | Tb09.211.1350 | 144 | 4 peptides | 2 peptides | 10% |

| | | | | | | |
|---|---|---|---|---|---|---|
| TRP_070330_131 | 11 | Tb927.8.3550/Tb08.10J17.940 | 119 | 3 peptides | 1 peptides | 7% |
| TRP_070330_131 | 12 | Tb927.4.1300/Tb04.2L9.440 | 113 | 3 peptides | 1 peptides | 7% |
| TRP_070330_131 | 13 | Tb09.160.3270 | 77 | 1 peptides | 1 peptides | 3% |
| TRP_070330_131 | 14 | Tb09.160.5530 | 60 | 1 peptides | 1 peptides | 5% |
| TRP_070330_131 | 15 | Tb927.7.1300/Tb07.27M11.560 | 59 | 1 peptides | 1 peptides | 2% |
| TRP_070330_131 | 16 | Tb927.7.1330/Tb07.27M11.600 | 56 | 2 peptides | 1 peptides | 5% |
| TRP_070330_131 | 17 | Tb927.3.3830/Tb03.28C22.630 | 46 | 1 peptides | 1 peptides | 2% |
| TRP_070330_131 | 19 | Tb09.160.4620 | 44 | 1 peptides | 1 peptides | 3% |
| TRP_070330_131 | 23 | Tb11.01.1670 | 36 | 3 peptides | 1 peptides | 8% |
| TRP_070330_131 | 24 | Tb11.01.5730 | 36 | 1 peptides | 1 peptides | 2% |
| TRP_070330_131 | 26 | Tb10.70.0280 | 34 | 2 peptides | 1 peptides | 3% |
| TRP_070330_132 | 1 | Tb927.8.5600/Tb08.26E13.220 | 622 | 16 peptides | 8 peptides | 36% |
| TRP_070330_132 | 2 | Tb09.160.3710 | 391 | 6 peptides | 5 peptides | 20% |
| TRP_070330_132 | 3 | Tb11.01.3170 | 359 | 10 peptides | 5 peptides | 22% |
| TRP_070330_132 | 4 | Tb927.3.2100/Tb03.30P12.1130 | 282 | 10 peptides | 3 peptides | 28% |
| TRP_070330_132 | 5 | Tb10.6k15.3850 | 266 | 9 peptides | 1 peptides | 25% |
| TRP_070330_132 | 6 | Tb09.211.0560 | 204 | 4 peptides | 4 peptides | 11% |
| TRP_070330_132 | 7 | Tb09.244.2730 | 182 | 7 peptides | 2 peptides | 13% |
| TRP_070330_132 | 8 | Tb11.01.3040 | 139 | 3 peptides | 3 peptides | 7% |
| TRP_070330_132 | 9 | Tb927.5.3830/Tb05.6E7.780 | 139 | 2 peptides | 2 peptides | 7% |
| TRP_070330_132 | 10 | Tb927.6.2740/Tb06.5F5.240 | 102 | 1 peptides | 1 peptides | 4% |
| TRP_070330_132 | 11 | Tb927.3.2960/Tb03.27C5.420 | 84 | 3 peptides | 2 peptides | 7% |
| TRP_070330_132 | 12 | Tb927.7.2640/Tb07.22O10.840 | 54 | 1 peptides | 1 peptides | 1% |
| TRP_070330_132 | 15 | Tb10.70.4740 | 45 | 3 peptides | 1 peptides | 5% |
| TRP_070330_132 | 16 | Tb10.70.4930 | 39 | 2 peptides | 1 peptides | 5% |
| TRP_070330_133 | 1 | Tb927.6.2740/Tb06.5F5.240 | 639 | 15 peptides | 11 peptides | 36% |
| TRP_070330_133 | 2 | Tb927.3.3490/Tb03.25B21.90 | 268 | 7 peptides | 2 peptides | 27% |
| TRP_070330_133 | 3 | Tb09.244.2730 | 245 | 8 peptides | 3 peptides | 18% |
| TRP_070330_133 | 4 | Tb927.5.3830/Tb05.6E7.780 | 199 | 4 peptides | 2 peptides | 11% |
| TRP_070330_133 | 5 | Tb11.01.3170 | 134 | 3 peptides | 2 peptides | 7% |
| TRP_070330_133 | 6 | Tb11.02.4250 | 131 | 2 peptides | 1 peptides | 7% |
| TRP_070330_133 | 7 | Tb09.211.0560 | 106 | 2 peptides | 2 peptides | 6% |
| TRP_070330_133 | 8 | Tb09.160.3710 | 106 | 3 peptides | 1 peptides | 10% |
| TRP_070330_133 | 13 | Tb10.70.7040 | 34 | 1 peptides | 1 peptides | 2% |
| TRP_070330_134 | 1 | Tb11.02.4200 | 261 | 6 peptides | 3 peptides | 25% |
| TRP_070330_134 | 2 | Tb927.4.2030/Tb04.29M18.690 | 252 | 5 peptides | 4 peptides | 13% |

| | | | | | | |
|---|---|---|---|---|---|---|
| TRP_070330_134 | 3 | Tb11.01.1290 | 219 | 4 peptides | 3 peptides | 16% |
| TRP_070330_134 | 4 | Tb11.01.5680 | 216 | 5 peptides | 1 peptides | 7% |
| TRP_070330_134 | 5 | Tb11.02.4700 | 147 | 4 peptides | 1 peptides | 12% |
| TRP_070330_134 | 6 | Tb927.8.2210/Tb08.26N11.790 | 70 | 1 peptides | 1 peptides | 2% |
| TRP_070330_134 | 7 | Tb09.244.2730 | 48 | 1 peptides | 1 peptides | 2% |
| TRP_070330_134 | 8 | Tb927.7.3440/Tb07.28B13.630 | 42 | 1 peptides | 1 peptides | 3% |
| TRP_070330_134 | 9 | Tb09.211.0120 | 37 | 2 peptides | 1 peptides | 5% |
| TRP_070330_134 | 13 | Tb09.v1.0660 | 29 | 1 peptides | 1 peptides | 22% |
| TRP_070330_134 | 15 | Tb10.70.2770 | 28 | 1 peptides | 1 peptides | 4% |
| TRP_070330_135 | 1 | Tb10.61.1880 | 314 | 7 peptides | 5 peptides | 18% |
| TRP_070330_135 | 2 | Tb927.5.1460/Tb05.30H13.580 | 125 | 5 peptides | 1 peptides | 16% |
| TRP_070330_135 | 3 | Tb927.4.2030/Tb04.29M18.690 | 123 | 3 peptides | 2 peptides | 13% |
| TRP_070330_135 | 4 | Tb927.3.2230/Tb03.48O8.710 | 99 | 5 peptides | 1 peptides | 18% |
| TRP_070330_135 | 5 | Tb927.7.5160/Tb07.27E10.390 | 76 | 3 peptides | 1 peptides | 8% |
| TRP_070330_135 | 7 | Tb09.244.2730 | 44 | 1 peptides | 1 peptides | 3% |
| TRP_070330_135 | 13 | Tb927.6.2740/Tb06.5F5.240 | 24 | 1 peptides | 1 peptides | 4% |
| TRP_070330_136 | 1 | Tb927.4.2030/Tb04.29M18.690 | 345 | 6 peptides | 6 peptides | 18% |
| TRP_070330_136 | 2 | Tb10.70.6610 | 173 | 5 peptides | 2 peptides | 22% |
| TRP_070330_136 | 3 | Tb927.4.3590/Tb04.26G5.430 | 147 | 4 peptides | 2 peptides | 24% |
| TRP_070330_136 | 4 | Tb10.70.6540 | 83 | 2 peptides | 1 peptides | 10% |
| TRP_070330_136 | 5 | Tb10.70.4740 | 58 | 2 peptides | 1 peptides | 6% |
| TRP_070330_136 | 6 | Tb10.70.3070 | 42 | 1 peptides | 1 peptides | 3% |
| TRP_070330_136 | 8 | Tb927.4.1300/Tb04.2L9.440 | 31 | 1 peptides | 1 peptides | 2% |
| TRP_070330_136 | 29 | Tb10.70.2770 | 20 | 1 peptides | 1 peptides | 4% |
| TRP_070330_137 | 1 | Tb11.01.1290 | 442 | 11 peptides | 5 peptides | 37% |
| TRP_070330_137 | 2 | Tb11.02.4700 | 413 | 8 peptides | 5 peptides | 35% |
| TRP_070330_137 | 3 | Tb09.160.0770 | 305 | 6 peptides | 5 peptides | 21% |
| TRP_070330_137 | 4 | Tb11.01.5680 | 235 | 6 peptides | 4 peptides | 8% |
| TRP_070330_137 | 5 | Tb09.244.2730 | 166 | 5 peptides | 2 peptides | 12% |
| TRP_070330_137 | 6 | Tb927.1.4830 | 154 | 4 peptides | 1 peptides | 14% |
| TRP_070330_137 | 7 | Tb927.4.2030/Tb04.29M18.690 | 126 | 4 peptides | 2 peptides | 12% |
| TRP_070330_137 | 9 | Tb10.70.4740 | 118 | 2 peptides | 1 peptides | 6% |
| TRP_070330_137 | 10 | Tb09.211.0120 | 106 | 3 peptides | 1 peptides | 19% |
| TRP_070330_137 | 11 | Tb927.7.3440/Tb07.28B13.630 | 68 | 1 peptides | 1 peptides | 3% |
| TRP_070330_137 | 12 | Tb11.01.7120 | 52 | 1 peptides | 1 peptides | 4% |
| TRP_070330_137 | 15 | Tb11.01.1570 | 32 | 1 peptides | 1 peptides | 3% |

| | | | | | | |
|---|---|---|---|---|---|---|
| TRP_070330_138 | 1 | Tb927.7.570/Tb07.8P12.860 | 270 | 5 peptides | 4 peptides | 26% |
| TRP_070330_138 | 2 | Tb11.01.3110 | 268 | 5 peptides | 4 peptides | 9% |
| TRP_070330_138 | 3 | Tb10.70.6540 | 127 | 5 peptides | 1 peptides | 23% |
| TRP_070330_138 | 4 | Tb927.8.5880/Tb08.11J15.1060 | 81 | 3 peptides | 1 peptides | 9% |
| TRP_070330_138 | 5 | Tb10.70.1100 | 69 | 1 peptides | 1 peptides | 6% |
| TRP_070330_138 | 6 | Tb11.01.4660 | 58 | 2 peptides | 1 peptides | 4% |
| TRP_070330_138 | 14 | Tb09.211.4460 | 27 | 1 peptides | 1 peptides | 6% |
| TRP_070330_139 | 1 | Tb11.01.3110 | 481 | 10 peptides | 8 peptides | 13% |
| TRP_070330_139 | 2 | Tb927.8.1990/Tb08.26N11.460 | 414 | 12 peptides | 6 peptides | 37% |
| TRP_070330_139 | 3 | Tb10.26.0200 | 198 | 4 peptides | 2 peptides | 19% |
| TRP_070330_139 | 4 | Tb10.70.6540 | 173 | 4 peptides | 2 peptides | 13% |
| TRP_070330_139 | 5 | Tb927.7.3450/Tb07.28B13.620 | 132 | 4 peptides | 2 peptides | 16% |
| TRP_070330_139 | 6 | Tb927.1.3200 | 123 | 3 peptides | 2 peptides | 12% |
| TRP_070330_139 | 7 | Tb927.2.2770/10C8.185 | 108 | 3 peptides | 2 peptides | 17% |
| TRP_070330_139 | 8 | Tb09.211.3210 | 73 | 2 peptides | 1 peptides | 11% |
| TRP_070330_139 | 9 | Tb927.6.2130/Tb06.4M18.380 | 67 | 3 peptides | 1 peptides | 18% |
| TRP_070330_139 | 10 | Tb11.03.0410 | 65 | 1 peptides | 1 peptides | 7% |
| TRP_070330_139 | 11 | Tb11.01.6670 | 63 | 4 peptides | 1 peptides | 11% |
| TRP_070330_139 | 13 | Tb11.02.5450 | 52 | 1 peptides | 1 peptides | 1% |
| TRP_070330_139 | 14 | Tb09.211.4550 | 52 | 1 peptides | 1 peptides | 6% |
| TRP_070330_139 | 16 | Tb927.4.3570/Tb04.26G5.400 | 44 | 2 peptides | 1 peptides | 7% |
| TRP_070330_140 | 1 | Tb09.211.4460 | 366 | 6 peptides | 4 peptides | 31% |
| TRP_070330_140 | 2 | Tb11.03.0250 | 117 | 2 peptides | 2 peptides | 16% |
| TRP_070330_140 | 4 | Tb09.211.0740 | 69 | 2 peptides | 1 peptides | 8% |
| TRP_070330_140 | 5 | Tb927.3.3450/Tb03.25B21.123 | 68 | 3 peptides | 2 peptides | 19% |
| TRP_070330_140 | 6 | Tb09.211.4550 | 65 | 1 peptides | 1 peptides | 6% |
| TRP_070330_140 | 7 | Tb11.01.7550 | 64 | 2 peptides | 1 peptides | 10% |
| TRP_070330_140 | 8 | Tb11.03.0530 | 63 | 1 peptides | 1 peptides | 3% |
| TRP_070330_140 | 10 | Tb09.160.3270 | 40 | 1 peptides | 1 peptides | 2% |
| TRP_070330_140 | 14 | Tb10.61.1750 | 31 | 3 peptides | 1 peptides | 2% |
| TRP_070330_142 | 1 | Tb11.02.2040 | 107 | 3 peptides | 2 peptides | 18% |
| TRP_070330_142 | 2 | Tb11.02.0815 | 92 | 3 peptides | 1 peptides | 13% |
| TRP_070330_142 | 9 | Tb11.46.0001 | 21 | 1 peptides | 1 peptides | 3% |
| TRP_070330_145 | 1 | Tb11.01.1680 | 139 | 5 peptides | 3 peptides | 6% |
| TRP_070330_146 | 1 | Tb927.7.5790/Tb07.10C21.170 | 142 | 3 peptides | 3 peptides | 22% |
| TRP_070330_146 | 2 | Tb11.02.2030 | 113 | 3 peptides | 1 peptides | 31% |

| | | | | | | |
|---|---|---|---|---|---|---|
| TRP_070330_147b | 1 | Tb927.5.1360/Tb05.30H13.400 | 145 | 5 peptides | 3 peptides | 22% |
| TRP_070330_147b | 2 | Tb10.6k15.2050 | 98 | 2 peptides | 2 peptides | 11% |
| TRP_070330_147b | 3 | Tb11.02.0210 | 77 | 3 peptides | 1 peptides | 4% |
| TRP_070330_147b | 4 | Tb11.02.0815 | 62 | 2 peptides | 1 peptides | 13% |
| TRP_070330_148 | 1 | Tb927.8.890/Tb08.12O16.530 | 208 | 4 peptides | 3 peptides | 19% |
| TRP_070330_148 | 2 | Tb11.03.0410 | 194 | 3 peptides | 2 peptides | 20% |
| TRP_070330_148 | 3 | Tb09.211.4460 | 134 | 2 peptides | 2 peptides | 11% |
| TRP_070330_148 | 4 | Tb11.01.7550 | 101 | 2 peptides | 1 peptides | 10% |
| TRP_070330_148 | 5 | Tb927.3.3450/Tb03.25B21.123 | 87 | 2 peptides | 2 peptides | 11% |
| TRP_070330_148 | 7 | Tb09.211.1690 | 55 | 1 peptides | 1 peptides | 5% |
| TRP_070330_149 | 1 | Tb11.02.4700 | 363 | 11 peptides | 5 peptides | 40% |
| TRP_070330_149 | 2 | Tb11.01.1290 | 361 | 10 peptides | 4 peptides | 30% |
| TRP_070330_149 | 3 | Tb927.1.4830 | 157 | 3 peptides | 2 peptides | 8% |
| TRP_070330_149 | 4 | Tb09.v1.0380 | 70 | 3 peptides | 1 peptides | 7% |
| TRP_070330_149 | 5 | Tb09.211.0120 | 60 | 2 peptides | 1 peptides | 13% |
| TRP_070330_150 | 1 | Tb09.v1.0380 | 249 | 6 peptides | 4 peptides | 18% |
| TRP_070330_150 | 2 | Tb11.02.2310 | 207 | 6 peptides | 3 peptides | 14% |
| TRP_070330_150 | 3 | Tb927.3.2100/Tb03.30P12.1130 | 82 | 3 peptides | 2 peptides | 11% |
| TRP_070330_150 | 4 | Tb11.02.4700 | 81 | 2 peptides | 2 peptides | 7% |
| TRP_070330_150 | 5 | Tb11.01.0700 | 80 | 1 peptides | 1 peptides | 7% |
| TRP_070330_150 | 6 | Tb09.244.2730 | 80 | 3 peptides | 1 peptides | 7% |
| TRP_070330_150 | 7 | Tb11.01.1290 | 63 | 1 peptides | 1 peptides | 3% |
| TRP_070330_150 | 8 | Tb927.7.3440/Tb07.28B13.630 | 60 | 1 peptides | 1 peptides | 3% |
| TRP_070330_150 | 9 | Tb927.7.2640/Tb07.22O10.840 | 52 | 1 peptides | 1 peptides | 1% |
| TRP_BN_151 | 1 | Tb09.v1.0380 | 236 | 5 peptides | 3 peptides | 15% |
| TRP_BN_151 | 2 | Tb927.3.2100/Tb03.30P12.1130 | 192 | 5 peptides | 2 peptides | 14% |
| TRP_BN_151 | 3 | Tb927.3.3490/Tb03.25B21.90 | 162 | 3 peptides | 2 peptides | 9% |
| TRP_BN_151 | 4 | Tb10.61.0540 | 133 | 3 peptides | 2 peptides | 7% |
| TRP_BN_151 | 5 | Tb10.70.4930 | 73 | 2 peptides | 1 peptides | 5% |
| TRP_BN_151 | 6 | Tb11.02.4700 | 55 | 1 peptides | 1 peptides | 3% |
| TRP_BN_151 | 7 | Tb927.7.3440/Tb07.28B13.630 | 52 | 2 peptides | 1 peptides | 7% |
| TRP_BN_151 | 8 | Tb11.02.2310 | 46 | 1 peptides | 1 peptides | 3% |
| TRP_BN_151 | 10 | Tb11.01.3170 | 39 | 1 peptides | 1 peptides | 2% |
| TRP_BN_151 | 11 | Tb09.211.0560 | 38 | 1 peptides | 1 peptides | 3% |
| TRP_BN_151 | 12 | Tb11.01.0700 | 38 | 1 peptides | 1 peptides | 5% |
| TRP_BN_151 | 14 | Tb927.6.2740/Tb06.5F5.240 | 37 | 1 peptides | 1 peptides | 3% |

| | | | | | | |
|---|---|---|---|---|---|---|
| TRP_BN_152 | 1 | Tb09.160.4590 | 398 | 12 peptides | 6 peptides | 26% |
| TRP_BN_152 | 3 | Tb10.70.5360 | 216 | 7 peptides | 3 peptides | 15% |
| TRP_BN_152 | 4 | Tb10.70.3710 | 180 | 5 peptides | 3 peptides | 12% |
| TRP_BN_152 | 5 | Tb927.7.1300/Tb07.27M11.560 | 180 | 5 peptides | 2 peptides | 12% |
| TRP_BN_152 | 6 | Tb09.160.5530 | 169 | 6 peptides | 3 peptides | 13% |
| TRP_BN_152 | 7 | Tb09.211.1350 | 109 | 2 peptides | 2 peptides | 5% |
| TRP_BN_152 | 8 | Tb10.61.0540 | 100 | 4 peptides | 1 peptides | 8% |
| TRP_BN_152 | 9 | Tb09.160.4620 | 85 | 2 peptides | 2 peptides | 5% |
| TRP_BN_152 | 11 | Tb11.55.0024 | 76 | 2 peptides | 1 peptides | 5% |
| TRP_BN_152 | 14 | Tb09.160.1950 | 59 | 1 peptides | 1 peptides | 2% |
| TRP_BN_152 | 15 | Tb927.7.1330/Tb07.27M11.600 | 47 | 1 peptides | 1 peptides | 1% |
| TRP_BN_152 | 16 | Tb10.389.0570 | 45 | 1 peptides | 1 peptides | 3% |
| TRP_BN_152 | 22 | Tb927.8.810/Tb08.12O16.380 | 28 | 1 peptides | 1 peptides | 3% |
| TRP_BN_152 | 29 | Tb927.7.1110/Tb07.27M11.260 | 23 | 1 peptides | 1 peptides | 1% |
| TRP_BN_152 | 44 | Tb927.4.1690/Tb04.2L9.1190 | 17 | 1 peptides | 1 peptides | 3% |
| TRP_BN_153 | 1 | Tb927.8.6060/Tb08.11J15.760 | 610 | 11 peptides | 9 peptides | 28% |
| TRP_BN_153 | 2 | Tb10.70.3710 | 433 | 10 peptides | 5 peptides | 16% |
| TRP_BN_153 | 3 | Tb927.4.1300/Tb04.2L9.440 | 216 | 5 peptides | 4 peptides | 11% |
| TRP_BN_153 | 4 | Tb11.01.3110 | 186 | 4 peptides | 4 peptides | 6% |
| TRP_BN_153 | 5 | Tb927.6.1990/Tb06.4M18.80 | 129 | 3 peptides | 2 peptides | 8% |
| TRP_BN_153 | 6 | Tb10.70.4740 | 128 | 2 peptides | 1 peptides | 4% |
| TRP_BN_153 | 7 | Tb09.211.1350 | 121 | 3 peptides | 2 peptides | 8% |
| TRP_BN_153 | 8 | Tb10.70.1130 | 119 | 2 peptides | 1 peptides | 5% |
| TRP_BN_153 | 9 | Tb09.160.4590 | 114 | 3 peptides | 3 peptides | 8% |
| TRP_BN_153 | 10 | Tb927.6.1800/Tb06.28P18.780 | 103 | 2 peptides | 2 peptides | 5% |
| TRP_BN_153 | 11 | Tb10.6k15.0990 | 84 | 2 peptides | 2 peptides | 4% |
| TRP_BN_153 | 12 | Tb927.7.1330/Tb07.27M11.600 | 78 | 2 peptides | 1 peptides | 4% |
| TRP_BN_153 | 13 | Tb10.70.5360 | 77 | 3 peptides | 1 peptides | 8% |
| TRP_BN_153 | 14 | Tb927.6.4670/Tb06.26G9.330 | 73 | 1 peptides | 1 peptides | 2% |
| TRP_BN_153 | 15 | Tb927.7.1300/Tb07.27M11.560 | 72 | 2 peptides | 1 peptides | 6% |
| TRP_BN_153 | 16 | Tb927.3.2960/Tb03.27C5.420 | 59 | 2 peptides | 1 peptides | 4% |
| TRP_BN_153 | 17 | Tb10.6k15.3970 | 48 | 2 peptides | 2 peptides | 4% |
| TRP_BN_153 | 18 | Tb10.6k15.2930 | 46 | 1 peptides | 1 peptides | 2% |
| TRP_BN_153 | 20 | Tb927.4.2450/Tb04.1H19.870 | 32 | 1 peptides | 1 peptides | 1% |
| TRP_BN_154 | 1 | Tb11.02.1690 | 423 | 10 peptides | 7 peptides | 13% |
| TRP_BN_154 | 2 | Tb10.6k15.2330 | 142 | 4 peptides | 2 peptides | 7% |

| | | | | | | |
|---|---|---|---|---|---|---|
| TRP_BN_154 | 3 | Tb10.70.0280 | 58 | 1 peptides | 1 peptides | 2% |
| TRP_BN_154 | 4 | Tb927.6.2740/Tb06.5F5.240 | 57 | 1 peptides | 1 peptides | 3% |
| TRP_BN_155 | 1 | Tb927.6.4840/Tb06.30P15.650 | 730 | 18 peptides | 11 peptides | 32% |
| TRP_BN_155 | 2 | Tb10.70.4740 | 625 | 13 peptides | 9 peptides | 25% |
| TRP_BN_155 | 3 | Tb09.160.3270 | 457 | 10 peptides | 6 peptides | 22% |
| TRP_BN_155 | 4 | Tb11.52.0013 | 247 | 5 peptides | 4 peptides | 11% |
| TRP_BN_155 | 5 | Tb927.8.3690/Tb08.10J17.710 | 162 | 4 peptides | 3 peptides | 10% |
| TRP_BN_155 | 6 | Tb10.70.1130 | 71 | 1 peptides | 1 peptides | 2% |
| TRP_BN_155 | 7 | Tb10.70.3710 | 69 | 2 peptides | 1 peptides | 6% |
| TRP_BN_155 | 8 | Tb927.5.1460/Tb05.30H13.580 | 68 | 1 peptides | 1 peptides | 3% |
| TRP_BN_155 | 9 | Tb11.01.5730 | 68 | 1 peptides | 1 peptides | 2% |
| TRP_BN_155 | 10 | Tb11.01.3080 | 50 | 1 peptides | 1 peptides | 1% |
| TRP_BN_155 | 12 | Tb11.02.0490 | 45 | 3 peptides | 1 peptides | 4% |
| TRP_BN_155 | 39 | Tb09.211.2420 | 21 | 1 peptides | 1 peptides | 0% |
| TRP_BN_156 | 1 | Tb09.160.5530 | 366 | 10 peptides | 4 peptides | 25% |
| TRP_BN_156 | 2 | Tb11.02.1690 | 169 | 4 peptides | 3 peptides | 5% |
| TRP_BN_156 | 3 | Tb10.70.3070 | 94 | 2 peptides | 2 peptides | 8% |
| TRP_BN_156 | 4 | Tb927.6.4590/Tb06.26G9.450 | 63 | 7 peptides | 1 peptides | 5% |
| TRP_BN_157 | 1 | Tb10.70.4740 | 636 | 13 peptides | 10 peptides | 24% |
| TRP_BN_157 | 2 | Tb09.160.3270 | 371 | 7 peptides | 5 peptides | 20% |
| TRP_BN_157 | 3 | Tb11.01.3110 | 202 | 4 peptides | 4 peptides | 7% |
| TRP_BN_157 | 4 | Tb11.01.4660 | 197 | 4 peptides | 3 peptides | 9% |
| TRP_BN_157 | 5 | Tb10.61.1870 | 68 | 3 peptides | 1 peptides | 7% |
| TRP_BN_157 | 6 | Tb927.1.2330 | 48 | 1 peptides | 1 peptides | 2% |
| TRP_BN_157 | 17 | Tb11.02.0070 | 30 | 1 peptides | 1 peptides | 2% |
| TRP_BN_158 | 1 | Tb10.6k15.1220 | 650 | 14 peptides | 9 peptides | 11% |
| TRP_BN_158 | 2 | Tb11.02.1210 | 630 | 14 peptides | 12 peptides | 11% |
| TRP_BN_158 | 3 | Tb11.02.5550 | 235 | 5 peptides | 2 peptides | 7% |
| TRP_BN_158 | 4 | Tb10.389.0880 | 222 | 5 peptides | 3 peptides | 6% |
| TRP_BN_158 | 5 | Tb927.1.3950 | 207 | 4 peptides | 3 peptides | 7% |
| TRP_BN_158 | 6 | Tb11.01.3110 | 165 | 3 peptides | 3 peptides | 5% |
| TRP_BN_158 | 7 | Tb10.26.1080 | 128 | 5 peptides | 2 peptides | 6% |
| TRP_BN_158 | 8 | Tb927.1.2340 | 90 | 2 peptides | 1 peptides | 3% |
| TRP_BN_159 | 1 | Tb09.211.3610 | 446 | 11 peptides | 6 peptides | 8% |
| TRP_BN_159 | 2 | Tb11.01.8770 | 121 | 4 peptides | 1 peptides | 3% |
| TRP_BN_159 | 6 | Tb927.5.1810/Tb05.1P6.730 | 35 | 2 peptides | 1 peptides | 3% |

| | | | | | | |
|---|---|---|---|---|---|---|
| TRP_BN_159 | 10 | Tb10.61.2680 | 26 | 1 peptides | 1 peptides | 3% |
| TRP_BN_160 | 1 | Tb10.389.0720 | 735 | 21 peptides | 8 peptides | 16% |
| TRP_BN_160 | 2 | Tb927.8.8330/Tb08.28A12.350 | 57 | 2 peptides | 1 peptides | 1% |
| TRP_BN_160 | 3 | Tb927.1.2330 | 41 | 1 peptides | 1 peptides | 2% |
| TRP_BN_161 | 1 | Tb10.26.1080 | 1096 | 27 peptides | 18 peptides | 31% |
| TRP_BN_161 | 3 | Tb927.4.760/Tb04.5E12.910 | 48 | 1 peptides | 1 peptides | 1% |
| TRP_BN_161 | 4 | Tb927.8.3680/Tb08.10J17.750 | 40 | 3 peptides | 1 peptides | 1% |
| TRP_BN_162 | 1 | Tb11.22.0005 | 649 | 15 peptides | 7 peptides | 20% |
| TRP_BN_162 | 2 | Tb11.01.3110 | 578 | 13 peptides | 8 peptides | 20% |
| TRP_BN_162 | 3 | Tb927.3.4290/Tb03.26J7.510 | 567 | 11 peptides | 10 peptides | 14% |
| TRP_BN_162 | 4 | Tb927.8.4970/Tb08.5H5.920 | 356 | 10 peptides | 6 peptides | 13% |
| TRP_BN_162 | 6 | Tb11.02.0070 | 38 | 1 peptides | 1 peptides | 2% |
| TRP_BN_162 | 27 | Tb11.03.0670 | 22 | 1 peptides | 1 peptides | 1% |
| TRP_BN_163 | 1 | Tb10.70.4740 | 212 | 4 peptides | 2 peptides | 9% |
| TRP_BN_163 | 2 | Tb927.1.2340 | 91 | 2 peptides | 1 peptides | 3% |
| TRP_BN_163 | 3 | Tb927.2.5160/30J2.30 | 88 | 4 peptides | 1 peptides | 7% |
| TRP_BN_163 | 4 | Tb927.3.5340/Tb03.5L5.520 | 82 | 2 peptides | 1 peptides | 4% |
| TRP_BN_163 | 5 | Tb927.1.700 | 59 | 1 peptides | 1 peptides | 2% |
| TRP_BN_163 | 6 | Tb10.70.7480 | 39 | 1 peptides | 1 peptides | 1% |
| TRP_BN_163 | 7 | Tb927.1.2330 | 36 | 1 peptides | 1 peptides | 2% |
| TRP_BN_164 | 1 | Tb927.7.7420/Tb07.30D13.360 | 320 | 7 peptides | 4 peptides | 10% |
| TRP_BN_164 | 2 | Tb09.160.4250 | 186 | 4 peptides | 2 peptides | 13% |
| TRP_BN_165 | 1 | Tb11.02.4440 | 1052 | 23 peptides | 17 peptides | 41% |
| TRP_BN_165 | 2 | Tb11.01.5710 | 453 | 9 peptides | 7 peptides | 16% |
| TRP_BN_165 | 3 | Tb10.61.2680 | 261 | 6 peptides | 3 peptides | 17% |
| TRP_BN_165 | 4 | Tb09.211.3540 | 74 | 2 peptides | 1 peptides | 4% |
| TRP_BN_165 | 5 | Tb927.1.2340 | 70 | 1 peptides | 1 peptides | 3% |
| TRP_BN_166 | 1 | Tb11.01.4660 | 150 | 3 peptides | 2 peptides | 7% |
| TRP_BN_166 | 2 | Tb11.02.4440 | 87 | 2 peptides | 1 peptides | 4% |
| TRP_BN_167 | 1 | Tb09.160.4250 | 269 | 6 peptides | 5 peptides | 26% |
| TRP_BN_167 | 2 | Tb10.70.3710 | 78 | 2 peptides | 1 peptides | 6% |
| TRP_BN_169 | 1 | Tb927.5.1460/Tb05.30H13.580 | 147 | 3 peptides | 2 peptides | 7% |
| TRP_BN_169 | 2 | Tb10.70.1490 | 96 | 2 peptides | 1 peptides | 7% |
| TRP_BN_170 | 1 | Tb10.70.1370 | 385 | 6 peptides | 6 peptides | 18% |
| TRP_BN_170 | 2 | Tb09.160.4250 | 103 | 3 peptides | 2 peptides | 16% |
| TRP_BN_170 | 3 | Tb927.4.1300/Tb04.2L9.440 | 52 | 1 peptides | 1 peptides | 2% |

| | | | | | | |
|---|---|---|---|---|---|---|
| TRP_BN_171 | 1 | Tb10.26.1080 | 783 | 18 peptides | 11 peptides | 20% |
| TRP_BN_171 | 2 | Tb11.01.3110 | 268 | 7 peptides | 4 peptides | 10% |
| TRP_BN_171 | 3 | Tb10.70.2650 | 222 | 5 peptides | 3 peptides | 6% |
| TRP_BN_171 | 4 | Tb09.211.3610 | 206 | 6 peptides | 3 peptides | 4% |
| TRP_BN_171 | 5 | Tb10.6k15.3600 | 153 | 4 peptides | 3 peptides | 5% |
| TRP_BN_171 | 6 | Tb10.389.0880 | 153 | 5 peptides | 3 peptides | 6% |
| TRP_BN_171 | 7 | Tb10.389.0630 | 106 | 3 peptides | 1 peptides | 2% |
| TRP_BN_171 | 8 | Tb927.1.2340 | 93 | 2 peptides | 1 peptides | 3% |
| TRP_BN_171 | 9 | Tb927.8.4970/Tb08.5H5.920 | 68 | 1 peptides | 1 peptides | 1% |
| TRP_BN_171 | 20 | Tb11.02.0070 | 25 | 1 peptides | 1 peptides | 2% |
| TRP_BN_172 | 1 | Tb10.26.1080 | 755 | 18 peptides | 11 peptides | 22% |
| TRP_BN_172 | 2 | Tb927.5.2940/Tb05.26K5.210 | 340 | 9 peptides | 6 peptides | 13% |
| TRP_BN_172 | 3 | Tb10.61.1750 | 270 | 6 peptides | 3 peptides | 7% |
| TRP_BN_172 | 4 | Tb927.8.4970/Tb08.5H5.920 | 244 | 7 peptides | 4 peptides | 10% |
| TRP_BN_172 | 5 | Tb10.389.0880 | 155 | 5 peptides | 2 peptides | 7% |
| TRP_BN_172 | 6 | Tb11.01.3110 | 145 | 3 peptides | 2 peptides | 5% |
| TRP_BN_172 | 7 | Tb10.70.2650 | 132 | 4 peptides | 2 peptides | 5% |
| TRP_BN_172 | 8 | Tb927.1.2340 | 130 | 3 peptides | 2 peptides | 6% |
| TRP_BN_172 | 9 | Tb09.211.3610 | 130 | 4 peptides | 2 peptides | 3% |
| TRP_BN_172 | 10 | Tb927.3.4290/Tb03.26J7.510 | 109 | 2 peptides | 2 peptides | 2% |
| TRP_BN_172 | 12 | Tb09.160.3630 | 49 | 1 peptides | 1 peptides | 1% |
| TRP_BN_172 | 13 | Tb927.1.2330 | 48 | 1 peptides | 1 peptides | 2% |
| TRP_BN_172 | 14 | Tb11.02.1120 | 48 | 1 peptides | 1 peptides | 1% |
| TRP_BN_172 | 15 | Tb927.4.3740/Tb04.26G5.680 | 44 | 2 peptides | 1 peptides | 0% |
| TRP_BN_173 | 1 | Tb10.61.1750 | 369 | 9 peptides | 5 peptides | 12% |
| TRP_BN_173 | 2 | Tb927.3.4290/Tb03.26J7.510 | 331 | 8 peptides | 5 peptides | 14% |
| TRP_BN_173 | 3 | Tb10.389.0720 | 234 | 4 peptides | 2 peptides | 4% |
| TRP_BN_173 | 4 | Tb10.26.1080 | 121 | 3 peptides | 2 peptides | 6% |
| TRP_BN_173 | 6 | Tb927.1.2340 | 78 | 2 peptides | 1 peptides | 3% |
| TRP_BN_173 | 7 | Tb927.8.4970/Tb08.5H5.920 | 60 | 1 peptides | 1 peptides | 1% |
| TRP_BN_173 | 8 | Tb927.1.2330 | 44 | 1 peptides | 1 peptides | 2% |
| TRP_BN_173 | 12 | Tb11.02.4830 | 39 | 1 peptides | 1 peptides | 1% |
| TRP_BN_173 | 14 | Tb11.01.3110 | 34 | 1 peptides | 1 peptides | 1% |
| TRP_BN_173 | 19 | Tb927.4.3740/Tb04.26G5.680 | 30 | 1 peptides | 1 peptides | 0% |
| TRP_BN_173 | 28 | Tb10.406.0560 | 26 | 2 peptides | 1 peptides | 1% |
